# Facile triflic acid-catalyzed α-1,2-*cis*-thio glycosylations: scope and application to the synthesis of *S*-linked oligosaccharides, glycolipids, sublancin glycopeptides, and T_N_/T_F_ antigens†
†Electronic supplementary information (ESI) available: Procedures, analytical data, and copties of ^1^H and ^13^C NMR spectra. See DOI: 10.1039/c9sc04079j


**DOI:** 10.1039/c9sc04079j

**Published:** 2019-10-01

**Authors:** Sanyong Zhu, Ganesh Samala, Eric T. Sletten, Jennifer L. Stockdill, Hien M. Nguyen

**Affiliations:** a Department of Chemistry , Wayne State University , Detroit , Michigan 48202 , USA . Email: hmnguyen@wayne.edu ; Email: stockdill@wayne.edu; b Department of Chemistry , University of Iowa , Iowa City , Iowa 52242 , USA

## Abstract

Studies of *S*-linked glycoconjugates have attracted growing interest because of their enhanced chemical stability and enzymatic resistance over *O*-glycoside counterparts. We here report a facile approach to access α-1,2-*cis-S*-linked glycosides using triflic acid as a catalyst to promote the glycosylation of a series of thiols with d-glucosamine, galactosamine, glucose, and galactose electrophiles. This method is broadly applicable for the stereoselective synthesis of *S*-linked glycopeptides, oligosaccharides and glycolipids in high yield and excellent α-selectivity. Many of the synthetic limitations associated with the preparation of these *S*-linked products are overcome by this catalytic method.

## Abstract 2

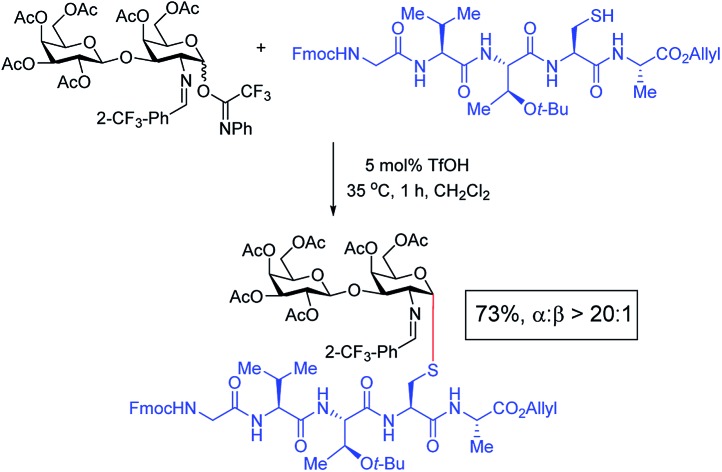
Studies of *S*-linked glycoconjugates have attracted growing interest because of their enhanced chemical stability and enzymatic resistance over *O*-glycoside counterparts.

## Introduction

Protein glycosylation, one of the most ubiquitous post-translational modifications, typically involves the attachment of carbohydrate chains to proteins through the hydroxyl group of serine or threonine (*O*-glycans) or the amido group of asparagine (*N*-glycans).^1^–^5^ The resulting glycoproteins exhibit a diverse array of biological functions such as cell adhesion, protein folding, signal transduction, and immune response.^1^–^5^ However, naturally occurring glycoproteins exist as mixtures of glycoforms, and their isolation as homogeneous species is a complicated process.^6^ As such, there is a great demand for methods to efficiently access structurally defined glycoproteins. Recently, replacement of the anomeric oxygen of *O*-linked glycosides with a sulfur atom to generate *S*-linked glycosides has attracted considerable attention because of the enhanced resistance of the latter to chemical and enzymatic hydrolysis.^7^,^8^ In addition, *S*-linked glycosides exhibit similar conformational preferences and equal or even improved biological activities compared to their native *O*-glycoside counterparts.^9^–^11^ In this context, *S*-linked glycan analogs could be utilized as structural mimetics and serve as powerful tools for the biological study of the natural *O*-linked substrates. The recent discovery of *S*-glycosylation, the addition of carbohydrate residues to the sulfur atom of cysteine on bacterial peptides,^12^–^14^ suggests that naturally existing *S*-linked glycoproteins may be more widespread than was previously thought and may lead to the development of new therapeutics.

Given the significant importance of thiol-containing carbohydrate molecules, methods that enable access to the challenging α-1,2-*cis* thiol glycosidic linkages are of high synthetic value. Formation of β-1,2-*trans* glycosides can be readily accomplished by employing electrophiles with C(2)-participatory groups.^15^ The stereoselective formation of α-1,2-*cis* glycosides, however, has proven challenging, and a mixture of α- and β-glycosides is often obtained.^15^ A variety of strategies have been reported for the synthesis of mucin-related α-thiol-containing GalNAc glycopeptide mimetics.^16^–^24^ Representative methods include stereoselective preparation of α-GalNAc thiols followed by (1) an S_N_2 displacement with β-bromoalanine-containing peptides,^16^,^17^ (2) site-selective conjugation with aziridine-containing peptides,^18^,^19^ or (3) conjugate addition to dehydroalanine-containing peptides.^20^ S_N_2 reaction of α-glycosyl thiols with 4-axial triflate glycosides^21^,^22^ 6-iodinated glycosides,^23^–^25^ enzymatic glycosylation,^26^–^29^ and metal-catalyzed cross coupling,^30^,^31^ have also been reported to generate α-1,2 *cis S*-linked oligosaccharides and glycopeptides. Despite these important advances over the past decades, numerous challenges remain, including the need for highly specialized coupling partners and the multistep preparation of bromoalanine-, aziridine-, and dehydroalanine-containing amino acid residues or peptides.^32^ In addition, most of the current methods are limited to specific classes of thiol nucleophiles. To date, there is only one reported method that is applicable for a variety of sulfur nucleophiles, and it uses glycosyl stannane to promote *S*-linked glycoside formation.^31^

We recently found that triflic acid, released from nickel triflate, can effectively promote the glycosylations of serine/threonine amino acids and hydroxyl groups of carbohydrates with the C(2)-*N-ortho*-(trifluoromethyl)benzylidenamino *N*-phenyl trifluoroacetimidates.^33^–^36^ This catalytic system features several advantages such as mild conditions, short reaction time, good yields and excellent levels of α-selectivity. However, the question remains whether it will be suited for *S*-glycosylations as Lewis acid-promoted reactions are known to be incompatible with thiol nucleophiles.^37^–^40^ Lewis acid-promoted *S*-glycosylations were underutilized with a few limited examples.^37^–^40^ The reactions generally proceed to provide the coupling products with low yields and require stoichiometric amount of promoter. An inherent issue with the *S*-nucleophiles is their tendency to undergo oxidation to form disulfide and other side products.^38^ They also react with the TMSOTf catalyst during the glycosylation process.^40^ All those factors make it more challenging to handle *S*-nucleophiles than their *O*-counterparts.

Herein we report a distinct approach to *S*-linked glycosylations: a triflic acid catalyst is shown to enable the facile α-1,2-*cis* synthesis of thiol-oligosaccharides, glycopeptide of antimicrobial sublancin, *S*-linked tumor-associated T_N_/T_F_ antigens, and thiol-glycolipids. This strategy obviates the need for substrate prefunctionalization and can proceed by direct coupling of thiol-containing molecules or cysteine-containing peptides with *N*-phenyl trifluoroacetimidates.

## Results and discussions

To test our hypothesis, we initiated our study by examining the glycosylation of *N*-Cbz cysteine methyl ester **2** with C(2)-*N-ortho*-(trifluoromethyl)benzylidene glucosamine *N*-phenyl trifluoroacetimidate **1** under previously established conditions (Table 1).^36^ To our delight, the coupling of **2** with **1** was successful using 15 mol% nickel triflate, Ni(OTf)_2_, (entry 1). As we expected based on the previously reported conditions,^36^ the reaction with 5 mol% triflic acid, TfOH, proceeded to completion within 1 h at 35 °C (entry 2) to afford the desired thiol-linked glycoconjugate **4** in 68% yield with excellent selectivity (α : β > 20 : 1). This result is consistent with our recently reported mechanism of the triflic acid-catalyzed α-selective 1,2-*cis* glycosylation with *N*-phenyl trifluoroacetimidate electrophiles.^36^ Specifically, triflic acid engages in the activation of electrophile **1** to generate a glycosyl triflate intermediate, which then undergoes equilibration from the stable α-anomer to the more reactive β-anomer. Subsequent S_N_2-like displacement of the reactive β-anomer of glycosyl triflate by **2** results in the formation of **4** with exclusive α-configuration.

**Table 1:** Optimization of the reaction conditions^*a*^

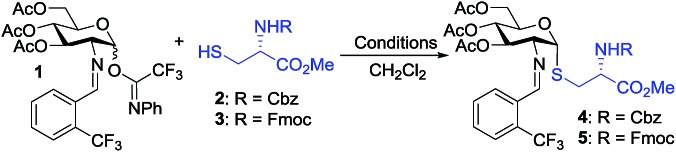						
Entry	**1** (equiv.)	**2** or **3** (equiv.)	Catalyst	Temp. (°C)	Time (h)	**4** or **5** yield (α : β)
1	1	**2** (1.5)	15 mol% Ni(OTf)_2_	35	16	**4**: 66% (>20 : 1)
2	1	**2** (1.5)	5 mol% TfOH	35	1	**4**: 64% (>20 : 1)
3	1	**2** (1.5)	5 mol% TfOH	25	2	**4**: 68% (>20 : 1)
4	1	**2** (1.5)	1 mol% TfOH	25	20	**4**: 67% (>20 : 1)
5	1.5	**2** (1.0)	3 mol% TfOH	25	3	**4**: 76% (>20 : 1)
6	2	**2** (1.0)	3 mol% TfOH	25	3	**4**: 81% (>20 : 1)
7	2	**2** (1.0)	5 mol% TfOH	25	1	**4**: 80% (>20 : 1)
8	2	**3** (1.0)	5 mol% TfOH	25	1	**5**: 78% (>20 : 1)

^*a*^The reaction was conducted with 0.1–0.2 mmol of donor **1**. Yields of the isolated product averaged two runs. The (α/β) ratios were determined by ^1^H NMR analysis.

We observed that while the reaction proceeded to completion faster with use of triflic acid, the glycal elimination product derived from donor **1** was also detected (see Fig. S1† for details). Lowering the reaction temperature (entry 3) and catalyst loading (entry 4) provided a similar outcome (Table 1). To address the problem of **1** from undergoing elimination, the cysteine residue **2** was utilized as the limiting reagent in the presence of 1.5 equiv. of donor **1**. This modification improved the yield to 76% (entry 5). Further increasing the donor **1** to two equivalents gave the desired coupling product **4** in an enhanced 81% yield while maintaining high levels of selectivity (entry 6). Most notably, the glycosylation reached completion within 1 h at 25 °C with use of 5 mol% TfOH to provide **4** with comparable yield and α-selectivity (entry 7). The cysteine residue **3** with *N*-Fmoc protection, commonly utilized in the solid-phase peptide synthesis (SPPS), also displayed good efficiency (entry 8) to provide the desired glycoconjugate **5** (Table 1) in good yield and α-selectivity.

**Fig. 1:**
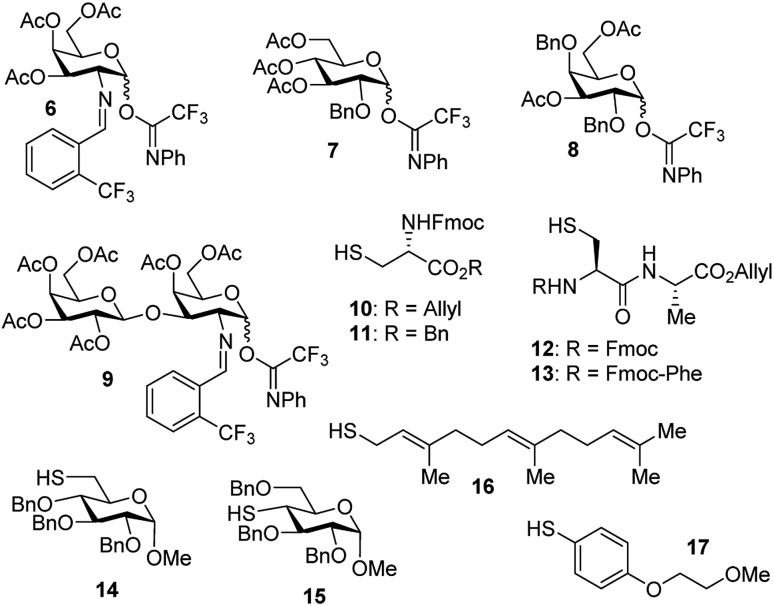
Carbohydrate donors and thiol-containing acceptors.

**Scheme 1:**
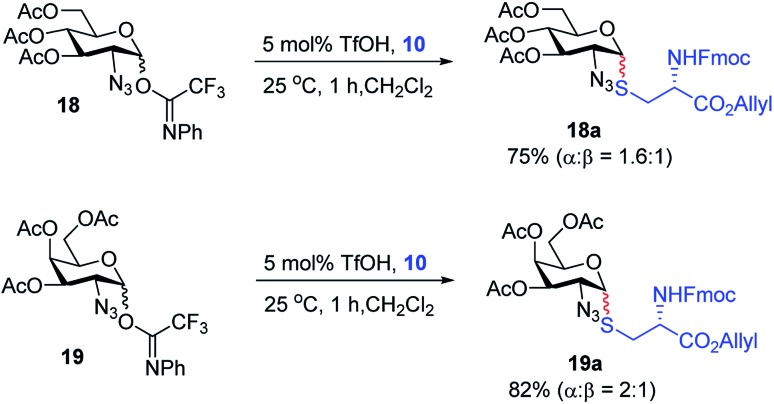
Glycosylation with C(2)-azido donors **18** and **19**.

With the optimized conditions in hand, we evaluated the scope of the triflic acid-catalyzed *S*-glycosylations (Fig. 1) using glycosyl *N*-phenyl trifluoroacetimidate donors (**1**, **6–9**) and thiol-containing molecules (**10–17**). Based on our previous study,^36^ we anticipated that *N*-phenyl trifluoroacetimidates **1**, **6** and **7** would exhibit high α-selectivity because their C(2)-*N*-benzylidene and C(2)-benzyl ether can modulate the electronic properties of the anomeric carbon, facilitating isomerization of the glycosyl triflate intermediate. Indeed, both donors **1** and **6** were effectively glycosylated to cysteine amino acids **10** and **11** to provide **1a**, **1b**, and **6a**, respectively, in good yield and with excellent α-selectivity (Table 2). There are several underlying factors that could influence the stereochemical outcome of the coupling event. To confirm that the α-selectivity arise from the directing ability of the C(2)-*N*-benzylidene group, we also coupled C(2)-azido donors **18** and **19** with cysteine residue **10** under standard conditions (Scheme 1). The desired products **18a** and **19a** were obtained with poor levels of α-selectivity (α : β = 1.6 : 1 to 2 : 1).

**Table 2:** Scope with respect to cysteine-containing peptides^*a*^

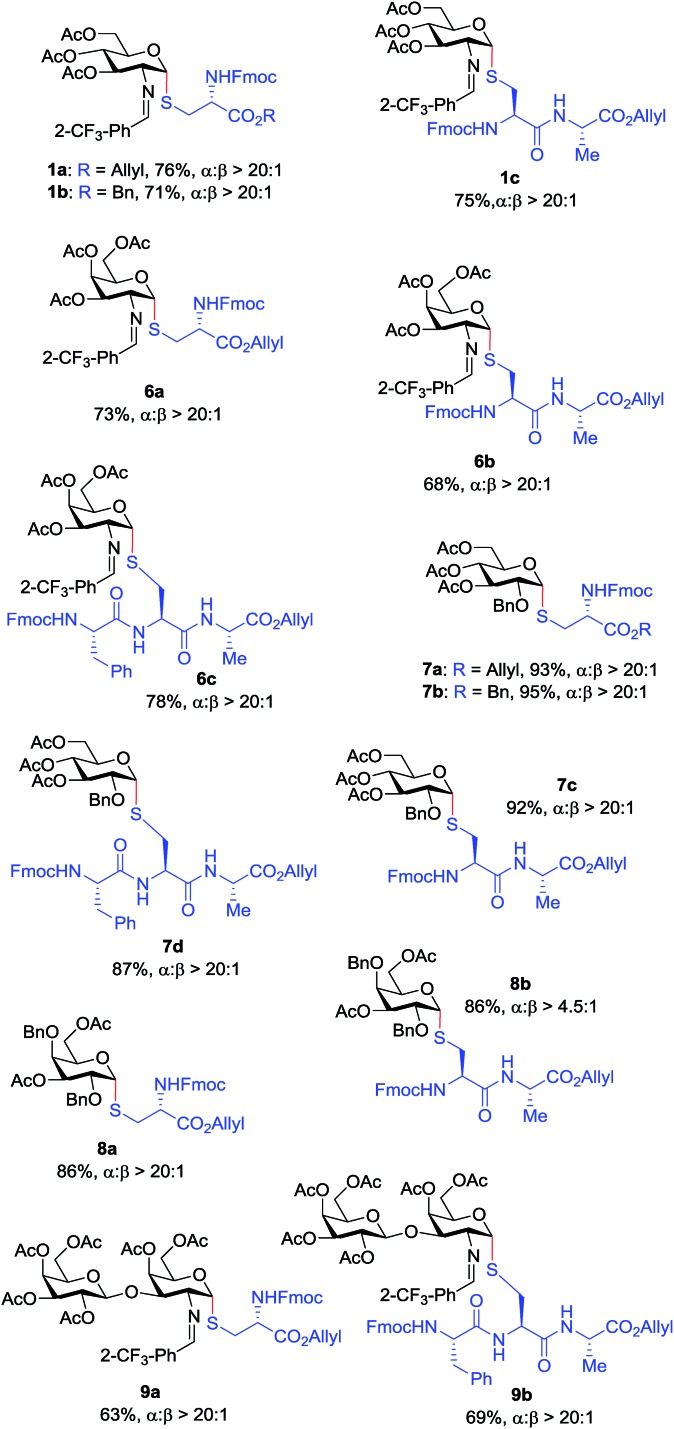

^*a*^All reactions were conducted with donor (2 equiv.), acceptor (1 equiv.) and 5 mol% TfOH in CH_2_Cl_2_ at 25 °C for 1 h. Yields of isolated *S*-linked glycopeptides averaged two runs. The (α/β) ratios were determined by ^1^H NMR analysis.

These results in Scheme 1 support the importance of the C(2)-*N*-benzylidene group in the triflic acid-catalyzed stereoselective α-1,2-*cis S*-linked glycosylation. Next, we expanded nucleophilic scope from single cysteine amino acid to more complex peptides. Both dipeptide **12** and tripeptide **13** (Fig. 1) performed well under the standard conditions with **1** and **6** to afford glycopeptides **1c**, **6b**, and **6c** (Table 2), presaging the potential utility of this transformation for access *S*-linked glycopeptides of tumor-associated mucin T_N_ and T_F_ antigens (*vide infra*).^11^,^44^

In addition to C(2)-*N*-benzylidene electrophiles **1** and **6**, the C(2)-*O*-benzyl protecting group is tolerated. Glucose donor **7** (Table 2) was an effective electrophilic partner, providing the coupling products **7a–7d** in excellent yields (87–95%) with exclusive α-configuration. More importantly, donor **7** does not generate a glycal elimination product. Employing the axial 4-*O*-benzyl protected donor **8** in place of the equatorial 4-*O*-acetyl group (**7**) slightly diminished the α-selectivity (**8a**: α : β > 20 : 1, **8b**: α : β = 4.5 : 1). Nevertheless, this result illustrates the ability of this catalytic system to overturn the inherent bias of d-galactose donors whose axial C(4)-*O*-benzyl protecting group has been reported to favor β-products.^41^ The difference in α-selectivity between the coupling products **8a** and **8b** could be explained by the relative rate for anomerization of the α-to the β-glycosyl triflate intermediate generated from the reaction of glycoyl electrophile **8** with triflic acid. Since a dipeptide is a more reactive nucleophile than a cysteine amino acid residue, less time is allowed for anomerization of the α-triflate to the more reactive β-triflate, resulting in decreasing the α-selectivity observed in the coupling product **8b**. Finally, disaccharide donor **9**, a carbohydrate motif of T_F_ antigen,^42^ was readily coupled to provide the corresponding 1,2-*cis S*-glycoconjugates **9a** and **9b** (63–69%, α : β > 20 : 1).

Inspired by the above discovery, we next examined the scope with respect to nucleophilic coupling partners **14–17**. As illustrated in Table 3, the glycosylations of primary (**14**) and secondary (**15**) thiol acceptors with donors **1**, **6**, and **7** proceeded smoothly to produce the desired *S*-linked disaccharides **14a–c** and **15a–c** in good yields (84–89%) with exclusive α-selectivity. The *trans*,*trans*-farnesyl mercaptan **16** was also a suitable nucleophile (**16a**, **b**), revealing the potential utility of this method for preparation of *S*-linked glycolipids. Recently, Messaoudi and co-workers reported the Pd-mediated cross coupling of aryl iodides with β-glycosyl thiols to generate *S*-linked glycosides with exclusive β-configuration.^30^ For comparison, we examined the coupling efficiency of our method with aryl thiol **17**. To our delight, the reaction compared favorably with excellent α-stereoselectivity and yield (**17a–c**).

**Table 3:** Scope with respect to thiol-containing acceptors^*a*^

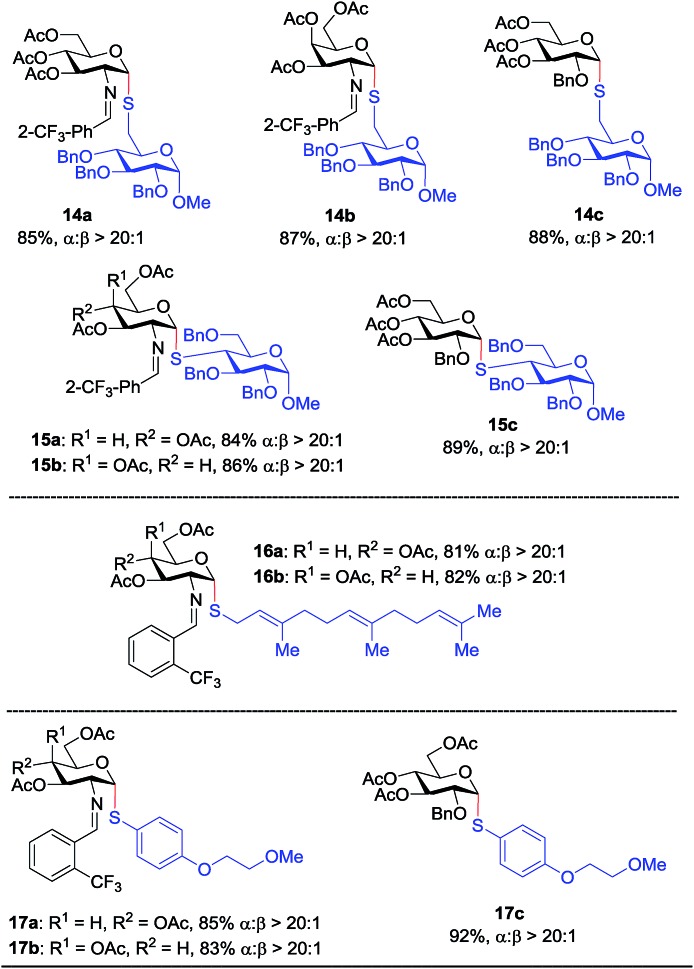

^*a*^See Table 2.

**Scheme 2:**
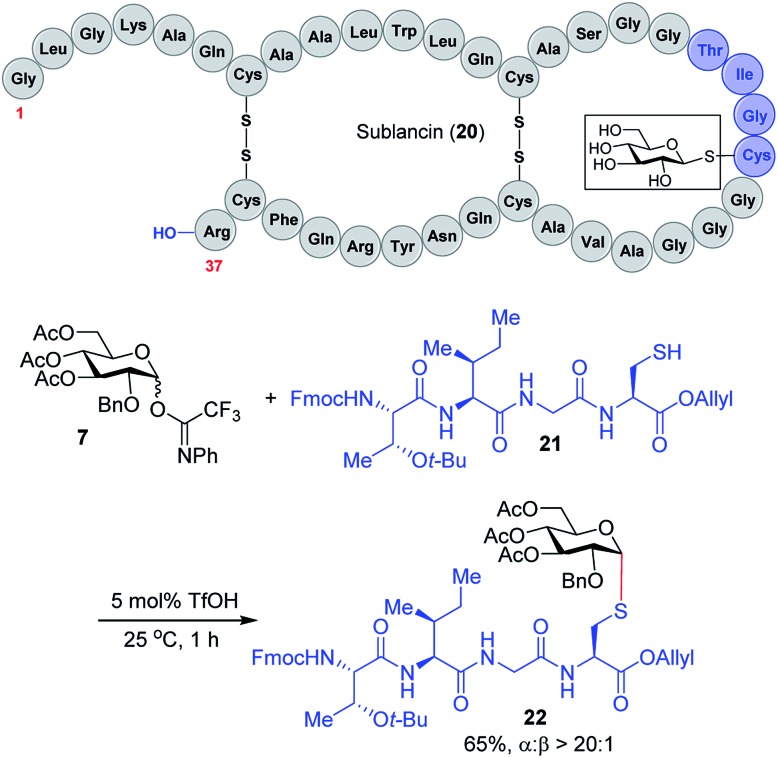
Synthesis of the sublancin glycopeptide fragment.

We expect that the triflic acid-catalyzed α-1,2-*cis S*-linked glycosylation method will be particularly useful when applied to the synthesis of bioactive glycopeptides. To establish the potential of a late-stage glycosylation approach, we prepared the tetrapeptide sequence surrounding the β-linked d-glucose unit in sublancin **20** (Scheme 2). This *S*-linked glycosyl unit is essential for antimicrobial activity.^12^ SunS, the recently discovered *S*-glycosyl transferase enzyme, is responsible for the unusual glycosylation of cysteine residues with carbohydrates.^12^ It has a relaxed substrate specificity and is able to glycosylate other hexose sugars, which has allowed its use in the preparation of sublancin analogs bearing other β-linked glycans.^12^,^13^ However, to the best of our knowledge, the activity of α-1,2-*cis S*-linked sublancin analogs has not been investigated,^10^ presumably because of the difficulty of α-1,2-*cis S*-glycosylations. Our current catalytic method would provide an ideal approach for the assembly of such analogs. Accordingly, we investigated the coupling of tetrapeptide **21** with glucose donor **7** in the presence of 5 mol% triflic acid. The glycosylation proceeded to completion within an hour to provide glycopeptide **22** in 65% yield with exclusive α-selectivity (Scheme 2).

**Scheme 3:**
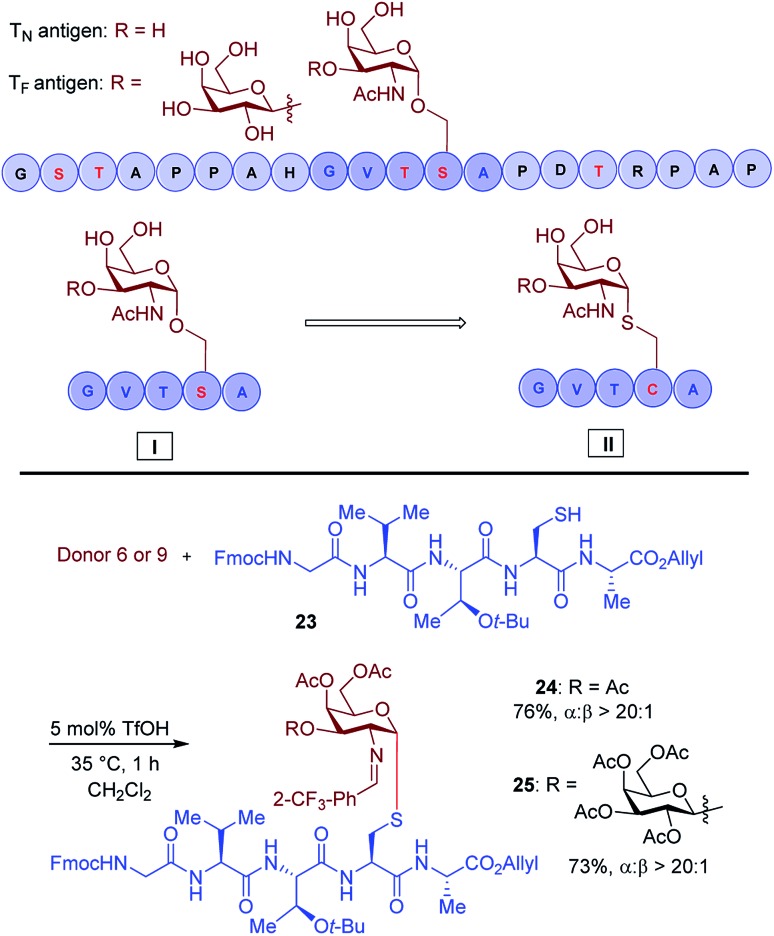
Substrate synthesis of *S*-linked T_N_ and T_F_ glycopeptide fragments.

Another important application of this method is the preparation of *S*-linked glycopeptides as analogs of MUC1 type tumor-associated T_N_/T_F_ antigens. MUC1 is a glycoprotein that consists of a tandem 20 amino acids repeating unit, with five possible *O*-glycosylation sites (serine or threonine) (Scheme 3).^43^ In normal cells, the protein backbone is decorated with complex oligosaccharides, while the glycosylation is incomplete in cancer cells. As such, multiple epitopes such as T_N_/T_F_ antigens are exposed to the immune system and can be targeted for the development of antitumor vaccines. A therapeutic vaccine employing multivalent T_N_-antigen clusters and CS4+ T-cell epitopes (MAG-Tn3) has entered to clinical trials.^44^ It has been reported that anti-T_N_ monoclonal antibodies (mAbs) have a binding preference for T_N_-Ser antigen, and the short MUC1 pentapeptide, GVTSA, is a suitable binding motif for mAbs.^43^ Since the *S*-linked T_N_ antigen has been proved to enhance the immunogenicity,^11^ we sought to prepare *S*-linked T_N_/T_F_ antigen mimetics by exchanging the 
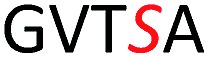
 sequence (I) for the 
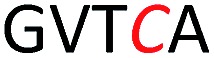
 sequence (II) (Scheme 3). Accordingly, we investigated the coupling of GVTCA pentapeptide **23** with both monosaccharide donor **6** and disaccharide donor **9** in the presence of 5 mol% TfOH. The coupling proceeded smoothly at 35 °C for 1 h to provide the corresponding 1,2-*cis S*-linked glycopeptides **24** (T_N_) and **25** (T_F_) in 76% and 73% yield, respectively, with exclusive α-selectivity (Scheme 3).

## Conclusions

In conclusion, we have illustrated the utility of the triflic acid-catalyzed α-1,2-*cis* thiol glycosylation reaction using stable glycosyl *N*-phenyl trifluoroacetimidates and thiol-containing molecules. This catalytic system obviates the need for substrate prefunctionalization and furnishes a diverse collection of synthetically valuable *S*-linked glycopeptides, oligosaccharides, and glycolipids with excellent α-selectivity under mild and operationally simple conditions. Notably, the facile nature of this reaction have also successfully applied to the synthesis of *S*-linked glycopeptides of antimicrobial sublancin and tumor-associated mucin T_N_/T_F_ antigens. Our future investigations will focus on transforming the current method to automated synthesis for the synthesis of the α-1,2-*cis S*-linked glycosides.

## Conflicts of interest

There are no conflicts to declare.

## Supplementary Material

Supplementary information
