# Supplementary material for: Facile triflic acid-catalyzed α-1,2-cis-thio glycosylations: scope and application to the synthesis of S-linked oligosaccharides, glycolipids, sublancin glycopeptides, and TN/TF antigens
Source: Chem Sci. 2019 Oct 1;10(45):10475–80. doi: 10.1039/c9sc04079j (PMC7020787; doi:10.1039/c9sc04079j)
Supplement: Supplementary file 1 [file SC-010-C9SC04079J-s001.pdf]

Electronic Supplementary Material (ESI) for Chemical Science.
This journal is © The Royal Society of Chemistry 2019

Facile Triflic Acid-Catalyzed α-1,2-*Cis*-Thiol Glycosylations: Scope and Applications to the Synthesis of *S*-Linked Oligosaccharides, Glycolipids, Sublancin Glycopeptide and T_N/T_F Antigens

Authors: Sanyong Zhu,[1] Ganesh Samala,[1] Eric T. Sletten,[2] Jennifer L. Stockdill[1]* and Hien M. Nguyen[1]*

Affiliations:

[1] Department of Chemistry, Wayne State University, Detroit, Michigan 48202, United States

[2] Department of Chemistry, University of Iowa, Iowa City, Iowa 52242, United States

*Correspondence to: stockdill@wayne.edu and hmnguyen@wayne.edu

Contents

1. Supporting Figure S1 ..... S2

2. General Information S3

3. Optimization Studies S4

4. Preparation of Glycosyl Donors S6

5. Preparation of Thiol Nucleophiles S15

6. Scope of Glycosylation S21

7. Synthesis of Sublancin Glycopeptide Fragment **22** S36

8. Synthesis of S-linked TN and TF Glycopeptide Fragments 24 and 25 ..... S37

9. References S38

10. Spectral Data S39

1. Supporting Figure

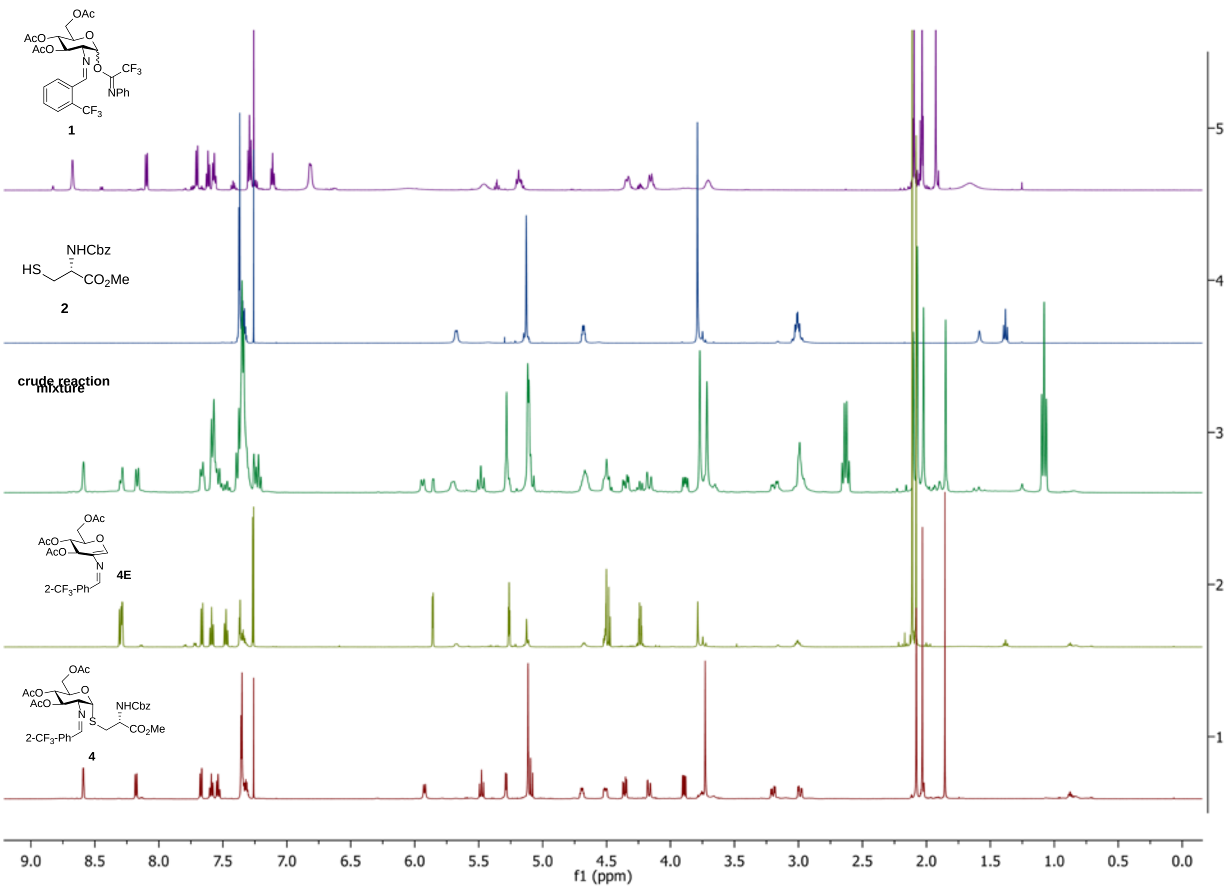

Figure S1. ^{1}H NMR Analysis of Glycosylation of Cysteine Amino acid Nucleophile 2 with *N*-Phenyl Trifluoroacetimidate Electrophile 1

The crude mixture resulted from the coupling of cysteine amino acid nucleophile **2** with *N*-phenyl trichloroacetimidate electrophile **1** was first analyzed by ^{1}H NMR spectroscopy. The crude mixture was purified by silica gel flash chromatography to separate the desired product **4** from the undesired elimination product **4E**. The result indicated the electrophile **1** was fully converted to the desired product **4** as exclusive α-isomer accompanied with the elimination product **4E**.

2. General Information

Methods and Reagents. All reactions were performed in oven-dried flasks fitted with septa under a positive pressure of nitrogen atmosphere. Organic solutions were concentrated using a Buchi rotary evaporator below 40 °C at 25 torr. Analytical thin-layer chromatography was routinely utilized to monitor the progress of the reactions and performed using pre-coated glass plates with 230-400 mesh silica gel impregnated with a fluorescent indicator (250 nm). Visualization was then achieved using UV light, iodine, or ceric ammonium molybdate. Flash column chromatography was performed using 40-63 μm silica gel (SiliaFlash F60 from Silicycle). Dry solvents were obtained from a SG Waters solvent system utilizing activated alumina columns under an argon pressure. All other commercial reagents were used as received from Sigma Aldrich, Alfa Aesar, Acros Organics, TCI, and Combi-Blocks, unless otherwise noted.

Instrumentation. All new compounds were characterized by Nuclear Magnetic Resonance (NMR) spectroscopy and High-Resolution Mass spectrometry (HRMS). All ^{1}H NMR spectra were recorded on either Agilent 400 or 600 MHz spectrometers. All ^{13}C NMR spectra were recorded on either Agilent 100 or 150 MHz spectrometer. Chemical shifts are expressed in parts per million (δ scale) referenced to the residual proton in the NMR solvent ($CDCl_3$: δ 7.26 ppm, δ 77.16 ppm). Data are presented as follows: chemical shift, multiplicity (s = singlet, d = doublet, t = triplet, q = quartet, m = multiplet, and bs = broad singlet), integration, and coupling constant in hertz (Hz).

High resolution mass spectra (HRMS) were recorded using a Micromass LCT Premier XE instrument (Waters) and were determined by electrospray ionization (ESI).

3. Optimization Studies

Table S1. Reaction Development[a]

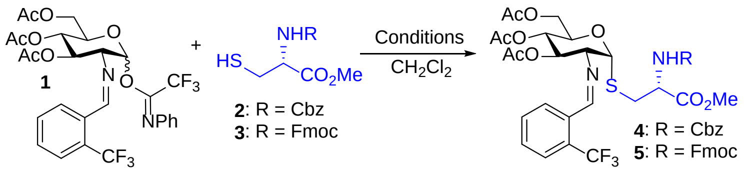

entry	1 (equiv.)	2 or 3 (equiv.)	catalyst	temp (°C)	time (h)	4 or 5 yield (α:β)
1	1	**2** (1.5)	15 mol% $Ni(OTf)_2$	35	16	**4**: 66% (>20:1)
2	1	**2** (1.5)	5 mol% TfOH	35	1	**4**: 64% (>20:1)
3	1	**2** (1.5)	5 mol% TfOH	25	2	**4**: 68% (>20:1)
4	1	**2** (1.5)	1 mol% TfOH	25	20	**4**: 67% (>20:1)
5	1.5	**2** (1.0)	3 mol% TfOH	25	3	**4**: 76% (>20:1)
6	2	**2** (1.0)	3 mol% TfOH	25	3	**4**: 81% (>20:1)
7	2	**2** (1.0)	5 mol% TfOH	25	1	**4**: 80% (>20:1)
8	2	**3** (1.0)	5 mol% TfOH	25	1	**5**: 78% (>20:1)

[a] The reaction was conducted with 0.1 – 0.2 mmol of 1. Yields of isolated product averaged two runs. The (α/β) ratios were determined by ^{1}H NMR analysis.

Procedure for Thiol Glycosylations Opitimization:

A 10 mL Schlenk flask was charged with *N*-phenyl trifluoroacetimidate glycosyl donor **1** (0.1 – 0.2 mmol, 1 – 2 equiv.), cysteine acceptor **2** or **3** (0.1 – 0.15 mmol, 1 – 1.5 equiv.) and dichloromethane (1 mL). The resulting solution was stirred at room temperature for 5 min under a nitrogen atmosphere before the catalyst (1 – 15 mol% with respect to donor **1**) was added. After the TLC shows completion of the reaction, it was quenched with 1 drop of Et_3N and concentrated. A crude ^{1}H NMR was taken to determine the (α/β) ratio. Further purification by silica gel column chromatography (ethyl acetate/hexane: 1/4→1/2) was performed to give the desired product.

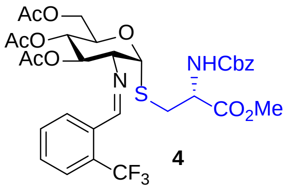

4

64 - 81%, α:β > 20:1

^{1}H NMR (CDCl$_3$, 600 MHz): δ 8.59 (d, *J* = 2.1 Hz, 1H), 8.18 (d, *J* = 7.7 Hz, 1H), 7.67 (d, *J* = 7.7 Hz, 1H), 7.59 (t, *J* = 7.5 Hz, 1H), 7.54 (t, *J* = 7.6 Hz, 1H), 7.40 – 7.29 (m, 5H), 5.92 (d, *J* = 8.5 Hz, 1H), 5.48 (t, *J* = 9.7 Hz, 1H), 5.29 (d, *J* = 5.6 Hz, 1H), 5.10 (dd, *J* = 15.9, 6.0 Hz, 3H), 4.73 – 4.64 (m, 1H), 4.51 (dd, *J* = 10.1, 3.1 Hz, 1H), 4.36 (dd, *J* = 12.4, 4.9 Hz, 1H), 4.17 (d, *J* = 10.9 Hz, 1H), 3.89 (dd, *J* = 10.0, 5.6 Hz, 1H), 3.73 (s, 3H), 3.20 (dd, *J* = 14.2, 5.4 Hz, 1H), 2.99 (dd, *J* = 14.2, 4.2 Hz, 1H), 2.08 (s, 3H), 2.03 (s, 3H), 1.85 (s, 3H).

^{13}C NMR (CDCl3, 150 MHz): δ = 171.02, 170.59, 169.85, 169.59, 160.37, 155.74, 136.09, 133.03, 132.27, 130.90, 129.07, 128.50, 128.20, 128.11, 125.48, 125.45, 85.18, 71.97, 71.33, 68.56, 68.52, 67.11, 62.09, 53.93, 53.75, 52.63, 33.42, 20.66, 20.64, 20.34.

HRMS (ESI): calc. for $C_{32}H_{35}F_3N_2O_{11}SNa$ $(M+Na)^+$: 735.1806; found: 735.1818.

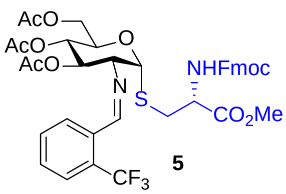

5

78%, α:β > 20:1

^{1}H NMR (CDCl$_3$, 600 MHz): δ 8.62 (d, *J* = 1.9 Hz, 1H), 8.19 (d, *J* = 7.7 Hz, 1H), 7.75 (t, *J* = 7.8 Hz, 2H), 7.67 (d, *J* = 7.6 Hz, 1H), 7.63 – 7.56 (m, 3H), 7.53 (t, *J* = 7.5 Hz, 1H), 7.43 – 7.36 (m, 2H), 7.34 – 7.27 (m, 2H), 6.00 (d, *J* = 8.6 Hz, 1H), 5.49 (t, *J* = 9.7 Hz, 1H), 5.27 (d, *J* = 5.6 Hz, 1H), 5.14 – 5.06 (m, 1H), 4.74 – 4.69 (m, 1H), 4.52 (dd, *J* = 10.1, 3.0 Hz, 1H), 4.42 (qd, *J* = 10.6, 7.3 Hz, 2H), 4.28 (dd, *J* = 12.3, 5.1 Hz, 1H), 4.24 – 4.19 (m, 2H), 3.92 (dd, *J* = 10.0, 5.6 Hz, 1H), 3.74 (s, 3H), 3.20 (dd, *J* = 14.3, 5.5 Hz, 1H), 2.99 (dd, *J* = 14.3, 4.1 Hz, 1H), 2.06 (s, 3H), 2.03 (s, 3H), 1.87 (s, 3H).

^{13}C NMR (CDCl3, 150 MHz): δ 170.96, 170.55, 169.85, 169.57, 160.36, 155.72, 143.64, 141.31, 141.28, 132.26, 130.90, 129.05, 127.71, 127.69, 127.06, 127.03, 125.49, 125.46, 125.03, 119.97, 119.94, 85.43, 72.03, 71.33, 68.64, 68.63, 67.03, 62.21, 54.05, 52.63, 47.11, 33.77, 20.65, 20.60, 20.33.

HRMS (ESI): calc. for $C_{39}H_{39}F_3N_2O_{11}SNa$ $(M+Na)^+$: 823.2119; found: 823.2116.

4. Preparation of Glycosyl Donors

Glycosyl donors **1**, **6** and **7** were synthesis according to our former literature.[1]

4.1 Preparation of glycosyl donor 8

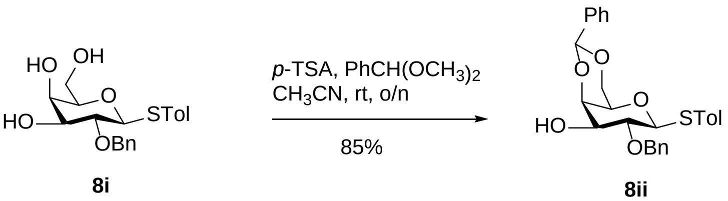

To a solution of **8i** (3.18 g, 8.46 mmol) in CH_3CN (80 mL) were added benzaldehyde dimethyl acetal (1.9 mL, 12.67 mmol) and *p*-TSA (161 mg, 0.846 mmol). After the reaction mixture was stirred at room temperature for overnight, it was quenched with Et_3N and concentrated. The residue was subjected to flush silica gel column chromatography (1:1.5, EtOAc–hexane) to give **8ii** (3.34 g, 85%) as a white foam. R_f 0.30 (1:1.5, EtOAc–hexane); ^{1}H NMR (600 MHz, $CDCl_3$) δ: 7.62 (d, J = 8.1 Hz, 2H), 7.54 – 7.48 (m, 2H), 7.46 – 7.39 (m, 5H), 7.36 (t, J = 7.4 Hz, 2H), 7.30 (d, J = 7.3 Hz, 1H), 7.07 (d, J = 7.9 Hz, 2H), 5.54 (s, 1H), 4.79 (d, J = 10.6 Hz, 1H), 4.70 (d, J = 10.6 Hz, 1H), 4.57 (d, J = 9.5 Hz, 1H), 4.38 (dd, J = 12.4, 1.3 Hz, 1H), 4.17 (d, J = 3.6 Hz, 1H), 4.00 (dd, J = 12.4, 1.4 Hz, 1H), 3.78 (dd, J = 9.0, 3.6 Hz, 1H), 3.62 (t, J = 9.3 Hz, 1H), 3.45 (s, 1H), 2.35 (s, 3H). ^{13}C NMR (150 MHz, $CDCl_3$) δ: 138.38, 137.71, 133.26, 129.65, 129.28, 128.88, 128.38, 128.20, 128.11, 127.78, 126.61, 101.37, 86.34, 77.10, 75.76, 75.23, 74.38, 69.75, 69.28, 21.19. HR ESI-TOF MS (m/z): calcd for $C_{27}H_{28}O_5SNa$ $[M + Na]^+$, 487.1550; found, 487.1555.

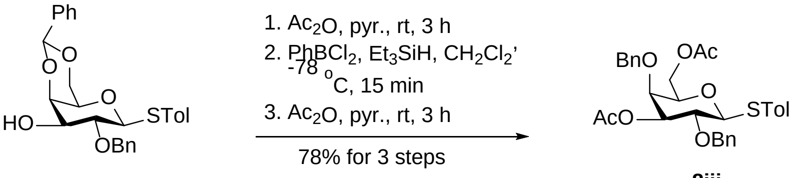

A solution of **8ii** (2.0 g, 4.31 mmol) in 50 mL anhydrous pyridine was added acetic anhydride (814 µL, 8.62 mmol) and stirred at room temperature for 3 h. The reaction mixture was concentrated under reduced pressure, and the residue was subjected to flush silica gel column chromatography (1:3, EtOAc–hexane). The obtained intermediate was dissolved in 50 mL anhydrous CH_2Cl_2, $PhBCl_2$ (1.12 mL, 8.62 mmol) and Et3SiH (1.37 mL, 8.62 mmol) were added at -78 ºC under a N_2

atmosphere. 15 min later, the reaction was quenched by addition of CH_3OH and Et_3N. The mixture was concentrated and purified by silica gel column chromatograpgy (1:2, EtOAc–hexane) to afford the intermediate product as syrup. It was dissolved in pyridine (50 mL) again, followed by addition of acetic anhydride (814 µL, 2.21 mmol). After the reaction mixture was stirred at room temperature for 3 h, it was concentrated and the residue was subjected to flush silica gel column chromatography (1:4, EtOAc–hexane) to give **8iii** (1.85 g, 78% for 3 steps) as a serup. R_f 0.50 (1:3, EtOAc–hexane); ^{1}H NMR (600 MHz, $CDCl_3$) δ: 7.48 (d, J = 8.1 Hz, 2H), 7.39 – 7.28 (m, 10H), 7.06 (d, J = 7.9 Hz, 2H), 4.96 (dd, J = 9.7, 2.9 Hz, 1H), 4.87 (d, J = 11.0 Hz, 1H), 4.68 (d, J = 11.5 Hz, 1H), 4.65 – 4.52 (m, 3H), 4.30 (dd, J = 11.1, 6.7 Hz, 1H), 4.10 (dd, J = 11.2, 6.3 Hz, 1H), 3.98 – 3.89 (m, 2H), 3.71 (t, J = 6.5 Hz, 1H), 2.33 (s, 3H), 2.02 (s, 3H), 1.94 (s, 3H). ^{13}C NMR (150 MHz, $CDCl_3$) δ: 170.40, 170.29, 138.06, 137.69, 137.63, 132.46, 129.71, 129.60, 128.38, 128.34, 127.99, 127.96, 127.88, 127.77, 88.05, 77.00, 75.59, 75.38, 75.36, 74.85, 74.13, 62.60, 21.12, 20.90, 20.78. HR ESI-TOF MS (m/z): calcd for $C_{31}H_{34}O_7SNa$ [M + Na]$^+$, 573.1917; found, 573.1911.

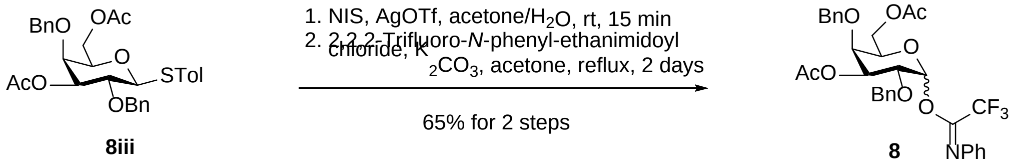

To a solution of **8iii** (1.5 g, 2.73 mmol) in acetone-H_2O (v/v, 50/1, 30 mL) were added NIS (1.23 g, 5.45 mmol) and AgOTf (140 mg, 0.545 mmol). After the reaction mixture was stirred at room temperature for 15 min, it was quenched with Et_3N, and concentrated. The residue was subjected to flush silica gel column chromatography (1:2, EtOAc–hexane) to give a hemiacetal. To a solution of this hemiacetal in anhydrous acetone (30 mL) was added 2,2,2-Trifluoro-*N*-phenyl-ethanimidoyl chloride (1.31 mL, 8.19 mmol) and K_2CO_3 (752 mg, 5.45 mmol) under N_2 protection. After 2 days of stirring under reflux, the mixture was filtered and concentrated in vacuum, and the residue was purified by Et_3N-neutralized silica gel column with EtOAc and hexanes (1:4) as the eluent to afford compound **8** (1.09 g, 65% for 2 steps) as a syrup.

For α anomer: R_f 0.55 (1:3, EtOAc–hexane); ^{1}H NMR (600 MHz, $CDCl_3$) δ: 7.39 – 7.22 (m, 12H), 7.09 (t, J = 7.5 Hz, 1H), 6.74 (s, 2H), 6.54 (s, 1H), 5.30 (d, J = 10.0 Hz, 1H), 4.70 (dd, J = 26.1, 11.6 Hz, 3H), 4.53 (d, J = 11.3 Hz, 1H), 4.20 (d, J = 7.4 Hz, 3H), 4.10 (d, J = 8.1 Hz, 2H), 2.05 (s, 3H), 2.04 (s, 3H). ^{13}C NMR (150 MHz, $CDCl_3$) δ: 170.32, 170.30, 137.68, 137.31, 128.70, 128.51,

128.43, 128.11, 127.90, 127.60, 119.34, 75.29, 74.51, 73.27, 72.91, 72.11, 70.65, 62.38, 20.96, 20.72. HR ESI-TOF MS (m/z): calcd for $C_{31}H_{35}N_3O_7SNa$ [M + Na]$^+$, 616.2093; found, 616.2088. For β anomer: R_f 0.50 (1:3, EtOAc–hexane); ^{1}H NMR (600 MHz, $CDCl_3$) δ: 7.42 – 7.27 (m, 12H), 7.11 (t, J = 7.5 Hz, 1H), 6.82 (d, J = 7.6 Hz, 2H), 5.70 (s, 1H), 4.97 (s, 1H), 4.84 (d, J = 11.5 Hz, 1H), 4.69 (d, J = 11.4 Hz, 2H), 4.55 (d, J = 11.5 Hz, 1H), 4.27 (dd, J = 11.0, 6.6 Hz, 1H), 4.16 – 4.02 (m, 2H), 3.94 (s, 1H), 3.75 (s, 1H), 1.99 (s, 3H), 1.98 (s, 3H). ^{13}C NMR (150 MHz, $CDCl_3$) δ: 170.25, 170.24, 143.33, 137.67, 137.31, 128.70, 128.50, 128.42, 128.25, 128.11, 128.02, 127.96, 124.30, 119.14, 97.03, 75.48, 75.04, 75.02, 74.61, 73.47, 72.89, 61.99, 20.83, 20.68. HR ESI-TOF MS (m/z): calcd for $C_{32}H_{32}F_3NO_8Na$ [M + Na]$^+$, 638.1972; found, 638.1976.

4.2 Preparation of glycosyl donor 9

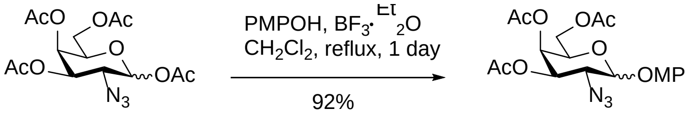

To a solution of **9i** (4.5 g, 12.03 mmol) in CH_2Cl_2 (70 mL) was added 4-Methoxyphenol (4.475 g, 36.09 mmol) and boron trifluoride diethyl etherate (2.97 mL, 24.06 mmol) at room temperature. After the reaction mixture was stirred under reflux for 1 day, it was diluted with CH_2Cl_2 and washed with 1 N NaOH and brine, dried over anhydrous Na_2SO_4, and concentrated. The residue was subjected to flush silica gel column chromatography (1:3, EtOAc–hexane) to give **9ii** (4.85 g, 92%) as an inseparable α/β mixture. R_f 0.50 (1:2, EtOAc–hexane); ^{1}H NMR (600 MHz, $CDCl_3$) δ: 7.04 (dd, J = 9.0, 1.4 Hz, 5H), 6.92 – 6.76 (m, 5H), 5.57 (dd, J = 11.1, 3.2 Hz, 1.7H), 5.51 (dd, J = 4.6, 2.2 Hz, 3H), 5.37 (d, J = 3.3 Hz, 1H), 4.84 (dd, J = 10.9, 3.4 Hz, 1H), 4.79 (d, J = 8.1 Hz, 1H), 4.39 (t, J = 6.6 Hz, 1.7H), 4.21 (dd, J = 11.3, 6.9 Hz, 1H), 4.17 – 4.04 (m, 5.5H), 4.00 – 3.90 (m, 1.7H), 3.84 – 3.68 (m, 8.5H), 2.17 (s, 3H), 2.16 (s, 5H), 2.08 (s, 5H), 2.07 (s, 3H), 2.04 (s, 3H), 1.98 (s, 5H). ^{13}C NMR (150 MHz, $CDCl_3$) δ: 170.26, 170.01, 169.96, 169.78, 169.73, 155.91, 155.63, 150.78, 150.19, 118.71, 118.21, 114.67, 114.58, 101.89, 97.94, 70.92, 68.13, 67.48, 67.37, 66.18, 61.49, 61.25, 60.62, 60.34, 57.35, 55.62, 20.63, 20.58, 20.57. HR ESI-TOF MS (m/z): calcd for $C_{19}H_{23}N_3O_9Na$ [M + Na]$^+$, 460.1327; found, 460.1328.

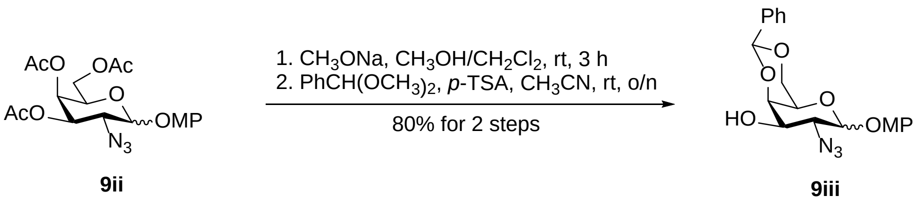

To a solution of **9ii** (4.2 g, 9.59 mmol) in CH_3OH/CH_2Cl_2 (v/v, 3/1, 80 mL) was added CH_3ONa (259 mg, 4.79 mmol). After the reaction mixture was stirred at room temperature for 3 h, it was quenched with Amberlyst 15 hydrogen resin, and concentrated. The obtained residue was dissolved in CH_3CN (80 mL), benzaldehyde dimethyl acetal (2.16 mL, 14.39 mmol) and *p*-TSA (182 mg, 0.959 mmol) were added. After the reaction mixture was stirred at room temperature for overnight, it was quenched with Et_3N and concentrated. The residue was subjected to flush silica gel column chromatography (1:2, EtOAc–hexane) to give **9iii** (3.07 g, 80% for 2 steps) as a white solid.

For α anomer: R_f 0.55 (1:2, EtOAc–hexane); ^{1}H NMR (600 MHz, $CDCl_3$) δ: 7.55 – 7.48 (m, 2H), 7.44 – 7.35 (m, 3H), 7.12 – 7.02 (m, 2H), 6.88 – 6.81 (m, 2H), 5.60 (s, 1H), 5.58 (d, *J* = 3.3 Hz, 1H), 4.39 (dd, *J* = 10.5, 3.7 Hz, 1H), 4.37 – 4.33 (m, 1H), 4.27 (dd, *J* = 12.7, 1.4 Hz, 1H), 4.06 (dd, *J* = 12.7, 1.7 Hz, 1H), 3.88 (d, *J* = 0.9 Hz, 1H), 3.78 (s, 3H), 3.70 (dd, *J* = 10.5, 3.3 Hz, 1H), 2.60 (s, 1H). ^{13}C NMR (150 MHz, $CDCl_3$) δ: 155.28, 150.52, 137.21, 129.40, 128.35, 126.20, 117.56, 114.72, 101.27, 98.14, 75.35, 69.14, 67.39, 63.36, 60.49, 55.65. HR ESI-TOF MS (m/z): calcd for $C_{31}H_{35}N_3O_7SNa$ [M + Na]$^+$, 616.2093; found, 616.2088.

For β anomer: R_f 0.20 (1:2, EtOAc–hexane); ^{1}H NMR (600 MHz, $CDCl_3$) δ: 7.52 (dd, *J* = 6.6, 3.0 Hz, 2H), 7.42 – 7.34 (m, 3H), 7.11 – 7.04 (m, 2H), 6.87 – 6.79 (m, 2H), 5.57 (s, 1H), 4.74 (d, *J* = 8.1 Hz, 1H), 4.36 (dd, *J* = 12.5, 1.4 Hz, 1H), 4.22 – 4.17 (m, 1H), 4.08 (dd, *J* = 12.6, 1.7 Hz, 1H), 3.87 (dd, *J* = 10.2, 8.1 Hz, 1H), 3.78 (s, 3H), 3.61 (dd, *J* = 10.2, 3.8 Hz, 1H), 3.50 (d, *J* = 1.1 Hz, 1H), 2.66 (s, 1H). ^{13}C NMR (150 MHz, $CDCl_3$) δ: 155.68, 151.06, 137.24, 129.41, 128.31, 126.41, 118.90, 114.53, 101.79, 101.44, 74.33, 71.34, 68.92, 66.63, 63.74, 55.63. HR ESI-TOF MS (m/z): calcd for $C_{20}H_{21}N_3O_6Na$ [M + Na]$^+$, 422.1323; found, 422.1313.

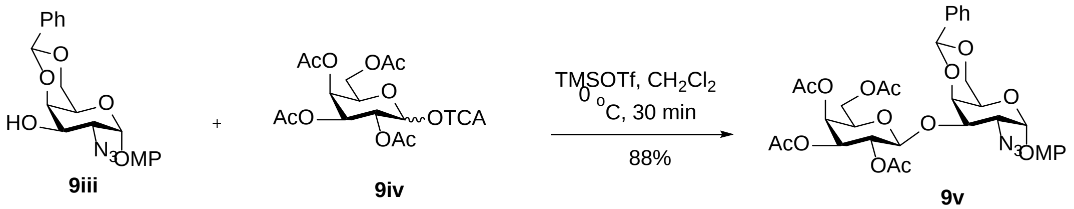

To a stirred mixture of donor **9iv** (4.06 g, 8.25 mmol), acceptor **9iii** (2.2 g, 5.5 mmol), and freshly activated MS 4Å (5 g), in anhydrous CH_2Cl_2 (100 mL) was added TMSOTf (99.4 μL, 0.55 mmol) under N_2 protection at 0 °C. After the reaction mixture was stirred for another 30 min, it was neutralized with Et_3N, filtered, and concentrated. The residue was subjected to silica gel column chromatography with EtOAc and hexanes (1:2) as the eluent to afford the **9v** (3.53 g, 88%) as a syrup. R_f 0.45 (1:1, EtOAc–hexane); ^{1}H NMR (600 MHz, $CDCl_3$) δ: 7.59 – 7.52 (m, 2H), 7.42 – 7.31 (m, 3H), 7.10 – 7.01 (m, 2H), 6.88 – 6.80 (m, 2H), 5.60 (d, J = 3.3 Hz, 1H), 5.58 (s, 1H), 5.44 – 5.40 (m, 1H), 5.32 (dd, J = 10.4, 7.9 Hz, 1H), 5.06 (dd, J = 10.4, 3.5 Hz, 1H), 4.86 (d, J = 7.9 Hz, 1H), 4.46 (d, J = 3.1 Hz, 1H), 4.32 (dd, J = 10.8, 3.3 Hz, 1H), 4.24 (ddd, J = 17.8, 11.9, 3.9 Hz, 2H), 4.14 (dd, J = 10.0, 5.3 Hz, 1H), 4.04 (dd, J = 12.5, 1.2 Hz, 1H), 3.97 (ddd, J = 14.4, 9.1, 5.0 Hz, 2H), 3.84 (s, 1H), 3.77 (s, 3H), 2.17 (s, 3H), 2.07 (s, 3H), 2.04 (s, 3H), 1.99 (s, 3H). ^{13}C NMR (150 MHz, $CDCl_3$) δ: 170.27, 170.24, 170.12, 169.41, 155.33, 150.46, 137.54, 128.89, 128.13, 126.09, 117.61, 114.72, 102.52, 100.60, 98.32, 75.79, 75.67, 71.03, 70.91, 69.07, 68.68, 66.97, 63.67, 61.40, 58.74, 55.63, 20.71, 20.70, 20.68, 20.54. HR ESI-TOF MS (m/z): calcd for $C_{34}H_{39}N_3O_{15}Na$ $[M + Na]^+$, 752.2273; found, 752.2278.

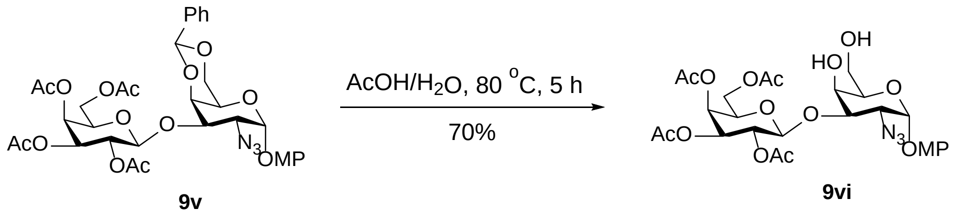

Compound **9v** (3.3 g, 4.52 mmol) was dissolved in mixed solvent of acetic acid/H_2O (4:1, 50 mL). After the reaction mixture was stirred at 80 °C for 5 h, it was concentrated and the residue was subjected to silica gel column chromatography with acetone and hexanes (1:2) as the eluent to afford the **9vi** (2.03 g, 70%) as a syrup. R_f 0.50 (1:1, acetone–hexane); ^{1}H NMR (600 MHz, $CDCl_3$) δ: 7.06 – 6.98 (m, 2H), 6.85 – 6.78 (m, 2H), 5.46 (d, J = 3.5 Hz, 1H), 5.40 (d, J = 2.7 Hz, 1H), 5.29 (dd, J = 10.5, 8.0 Hz, 1H), 5.05 (dd, J = 10.5, 3.4 Hz, 1H), 4.78 (d, J = 8.0 Hz, 1H), 4.27 (d, J = 1.7 Hz, 1H), 4.22 (dd, J = 10.5, 3.1 Hz, 1H), 4.17 (dd, J = 11.5, 7.3 Hz, 1H), 4.10 (dd, J = 11.5, 5.5 Hz, 1H), 4.03 (t, J = 5.6 Hz, 1H), 3.98 (t, J = 6.6 Hz, 1H), 3.88 (dd, J = 11.7, 5.6 Hz, 1H), 3.80 (dd, J = 11.7, 5.1 Hz, 1H), 3.77 – 3.73 (m, 4H), 2.15 (s, 3H), 2.09 (s, 3H), 2.04 (s, 3H), 1.98 (s, 3H). ^{13}C NMR (150 MHz, $CDCl_3$) δ: 170.44, 170.11, 170.04, 169.63, 155.50, 150.40, 118.28, 114.72, 101.98, 98.40, 78.16, 71.29, 70.66, 69.93, 69.23, 68.39, 66.99, 62.44, 61.55, 58.42, 55.61,

20.63, 20.58, 20.56, 20.50. HR ESI-TOF MS (m/z): calcd for $C_{27}H_{35}N_3O_{15}Na$ $[M + Na]^+$, 664.1960; found, 664.1969.

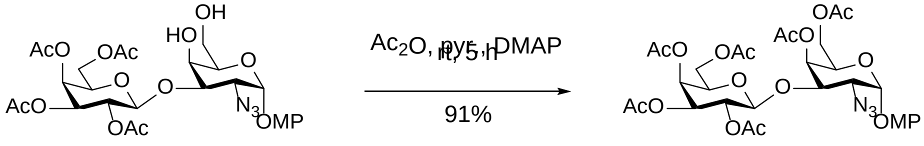

9vi **9vii**

To a solution of **9vi** (1.8 g, 2.80 mmol) in pyridine (30 mL) was added acetic anhydride (1.06 mL, 11.21 mmol) and DMAP (34.2 mg, 0.28 mmol). After the reaction mixture was stirred at room temperature for 5 h, it was concentrated and the residue was subjected to flush silica gel column chromatography (1:2, EtOAc–hexane) to give **9vii** (1.85 g, 91%) as a serup. R_f 0.50 (1:1, EtOAc–hexane); ^{1}H NMR (600 MHz, $CDCl_3$) δ: 7.04 – 6.97 (m, 2H), 6.83 – 6.78 (m, 2H), 5.51 (d, J = 3.2 Hz, 1H), 5.44 (d, J = 3.5 Hz, 1H), 5.36 – 5.34 (m, 1H), 5.19 (dd, J = 10.5, 7.8 Hz, 1H), 5.01 (dd, J = 10.5, 3.4 Hz, 1H), 4.75 (d, J = 7.8 Hz, 1H), 4.30 – 4.26 (m, 2H), 4.15 (dt, J = 11.0, 5.2 Hz, 2H), 4.08 (dd, J = 7.0, 4.2 Hz, 1H), 3.99 – 3.90 (m, 2H), 3.78 – 3.72 (m, 4H), 2.13 (s, 3H), 2.12 (s, 3H), 2.07 (s, 3H), 2.03 (s, 3H), 1.96 (s, 3H), 1.93 (s, 3H). ^{13}C NMR (150 MHz, $CDCl_3$) δ: 170.36, 170.33, 170.19, 170.02, 169.64, 169.47, 155.62, 150.20, 149.05, 136.57, 118.43, 114.61, 101.56, 98.02, 74.73, 70.84, 70.77, 69.32, 68.77, 68.13, 66.78, 62.54, 61.06, 59.40, 55.59, 20.69, 20.62, 20.60, 20.59, 20.50. HR ESI-TOF MS (m/z): calcd for $C_{31}H_{39}N_3O_{17}Na$ $[M + Na]^+$, 748.2172; found, 748.2162.

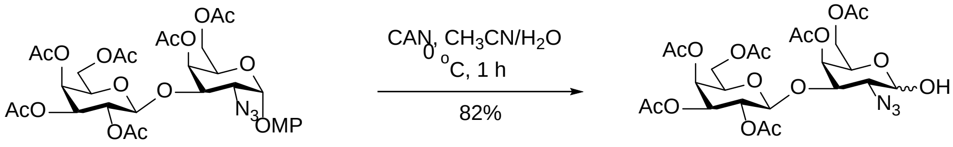

9vii **9ix**

To a solution of **9vii** (2.5 g, 3.44 mmol) in acetonitrile/H_2O (1:1, 60 mL) was added cerium ammonium nitrate (5.66 g, 10.33 mmol) at 0 °C. After the reaction mixture was stirred at the same temperature for 1 h, it was diluted with ethyl acetate and washed with aqueous $NaHCO_3$ and brine, dried over anhydrous Na_2SO_4, and concentrated. The residue was subjected to flush silica gel column chromatography (1.5:1, EtOAc–hexane) to give **9ix** (1.75 g, 82%) as a α/β mixture. R_f 0.30 (1.5:1, EtOAc–hexane); ^{1}H NMR (600 MHz, $CDCl_3$) δ: 5.48 (d, J = 2.9 Hz, 1.1H), 5.40 (d, J = 3.4 Hz, 1.1H), 5.37 – 5.32 (m, 2.3H), 5.16 (ddd, J = 15.2, 10.5, 7.8 Hz, 1.8H), 5.00 (dd, J = 10.4, 3.4 Hz, 1.7H), 4.71 (dd, J = 12.0, 7.8 Hz, 1.8H), 4.60 (d, J = 6.9 Hz, 1H), 4.37 – 4.32 (m, 1.1H), 4.22 – 4.05 (m, 7.4H), 3.98 (ddd, J = 20.8, 11.7, 7.4 Hz, 2.1H), 3.89 (dt, J = 16.9, 6.5 Hz, 1.9H),

3.81 – 3.76 (m, 1.3H), 3.73 (dd, *J* = 10.6, 3.5 Hz, 1.2H), 3.55 (p, *J* = 10.3 Hz, 1.7H), 3.24 (s, 1.3H), 2.15 (d, *J* = 0.4 Hz, 4.6H), 2.13 (d, *J* = 3.2 Hz, 4.4H), 2.08 (s, 2.2H), 2.06 (d, *J* = 0.4 Hz, 6.7H), 2.05 (d, *J* = 1.3 Hz, 4.4H), 2.04 (s, 1.9H), 1.97 (d, *J* = 2.3 Hz, 4.5H). ^{13}C NMR (150 MHz, $CDCl_3$) δ: 101.45, 101.42, 96.35, 92.32, 77.35, 74.40, 71.70, 70.84, 70.81, 70.77, 70.63, 69.52, 68.81, 68.77, 68.12, 67.47, 66.80, 66.75, 64.49, 62.76, 62.58, 61.06, 60.97, 60.39, 60.16, 20.77, 20.73, 20.66, 20.62, 20.60, 20.52. HR ESI-TOF MS (m/z): calcd for $C_{24}H_{33}N_3O_{16}Na$ [M + Na]$^+$, 642.1753; found, 642.1759.

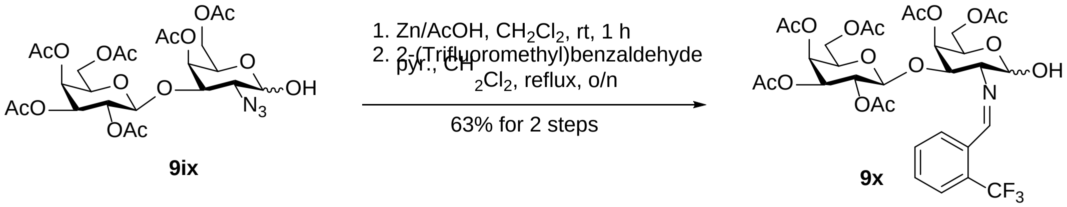

To a solution of **9ix** (1.7 g, 2.74 mmol) in CH_2Cl_2 (30 mL) was added zinc powder (5.35 g, 82.25 mmol) and acetic acid (4.7 mL, 82.25 mmol). After the reaction mixture was stirred at room temperature for 1 h, it was filtered and concentrated. The obtained residue was dissolved in pyridine/CH_2Cl_2 (1:10, 30 mL), 2-(Trifluoromethyl) benzaldehyde (433 µL, 3.29 mmol) was added. After the reaction mixture was stirred under reflux for overnight, it was concentrated. The residue was subjected to flush silica gel column chromatography (2:1, EtOAc–hexane) to give **9x** (1.3 g, 63% for 2 steps) as a α/β mixture. R_f 0.30 (2:1, EtOAc–hexane); ^{1}H NMR (600 MHz, $CDCl_3$) δ: 8.83 (d, *J* = 16.1 Hz, 2.2H), 8.32 (dd, *J* = 21.5, 7.4 Hz, 2.2H), 7.86 (d, *J* = 7.2 Hz, 2.3H), 7.77 (t, *J* = 7.3 Hz, 1H), 7.75 – 7.65 (m, 3.5H), 5.66 (s, 1H), 5.58 (s, 1.6H), 5.44 (s, 2.2H), 5.34 (s, 1H), 5.17 (dd, *J* = 12.3, 5.9 Hz, 2.2H), 5.05 – 4.96 (m, 3.6H), 4.73 (d, *J* = 7.4 Hz, 2.2H), 4.67 (s, 1H), 4.58 (d, *J* = 10.3 Hz, 1H), 4.44 – 4.37 (m, 2.5H), 4.34 – 4.13 (m, 11H), 4.09 (d, *J* = 3.6 Hz, 1.7H), 4.04 – 3.96 (m, 3H), 3.80 (s, 1H), 3.69 (t, *J* = 8.3 Hz, 1.6H), 2.28 (d, *J* = 13.7 Hz, 13H), 2.25 – 2.16 (m, 15H), 2.03 (d, *J* = 1.3 Hz, 5.4H), 1.62 (d, *J* = 11.0 Hz, 5.4H). ^{13}C NMR (150 MHz, $CDCl_3$) δ: 170.66, 170.39, 170.38, 170.28, 170.06, 170.02, 168.77, 161.72, 161.48, 133.49, 132.11, 131.99, 131.00, 130.75, 129.27, 128.46, 128.23, 125.84, 124.80, 122.98, 100.68, 95.41, 94.16, 77.43, 74.07, 73.72, 71.84, 70.83, 70.77, 70.63, 70.61, 70.25, 68.93, 68.85, 68.69, 67.95, 67.83, 66.72, 66.68, 63.03, 63.01, 60.98, 60.94, 60.37, 20.98, 20.81, 20.77, 20.76, 20.72, 20.64, 20.59, 20.43, 19.87. HR ESI-TOF MS (m/z): calcd for $C_{32}H_{38}F_3NO_{16}Na$ [M + Na]$^+$, 772.2035; found, 772.2041.

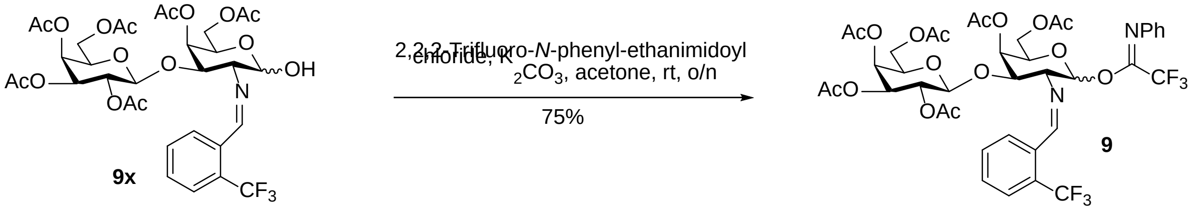

To a solution of **9x** (1.04 g, 1.39 mmol) in anhydrous acetone (12 mL) was added 2,2,2-Trifluoro-*N*-phenyl-ethanimidoyl chloride (443 μL, 2.77 mmol) and K_2CO_3 (382 mg, 2.77 mmol) under N_2 protection. After overnight stirring at room temperature, the mixture was filtered and concentrated in vacuum, and the residue was purified by Et_3N-neutralized silica gel column with EtOAc and hexanes (1:1) as the eluent to afford compound **9** (0.96 g, 75%) as a yellow solid.

For α anomer: R_f 0.65 (1.5:1, EtOAc–hexane); ^{1}H NMR (400 MHz, $CDCl_3$) δ: 8.69 (s, 1H), 8.26 (d, J = 7.7 Hz, 1H), 7.74 (d, J = 7.7 Hz, 1H), 7.67 (t, J = 7.5 Hz, 1H), 7.60 (t, J = 7.4 Hz, 1H), 7.14 (t, J = 7.7 Hz, 2H), 7.02 (t, J = 7.4 Hz, 1H), 6.59 (d, J = 6.9 Hz, 2H), 6.25 (s, 1H), 5.61 (d, J = 2.3 Hz, 1H), 5.32 (d, J = 3.3 Hz, 1H), 5.04 (dd, J = 10.4, 7.9 Hz, 1H), 4.87 (dd, J = 10.4, 3.4 Hz, 1H), 4.61 (d, J = 7.8 Hz, 1H), 4.52 – 4.40 (m, 2H), 4.28 (dd, J = 11.7, 4.7 Hz, 1H), 4.19 – 3.98 (m, 4H), 3.89 (t, J = 6.6 Hz, 1H), 2.16 (s, 3H), 2.13 (s, 3H), 2.10 (s, 3H), 2.06 (s, 3H), 1.89 (s, 3H), 1.52 (s, 3H). ^{13}C NMR (100 MHz, $CDCl_3$) δ: 170.47, 170.40, 170.23, 170.01, 169.80, 168.79, 161.70, 133.39, 132.07, 131.02, 128.96, 128.61, 128.35, 125.83, 124.23, 119.76, 119.27, 100.60, 70.88, 70.83, 70.37, 68.98, 68.51, 68.04, 66.80, 62.62, 61.14, 20.72, 20.59, 20.58, 20.45, 19.90. HR ESI-TOF MS (m/z): calcd for $C_{40}H_{42}F_6N_2O_{16}Na$ $[M + Na]^+$, 943.2331; found, 943.2338.

For β anomer: R_f 0.55 (1.5:1, EtOAc–hexane); ^{1}H NMR (400 MHz, $CDCl_3$) δ: 8.71 (d, J = 1.4 Hz, 1H), 8.17 (d, J = 7.7 Hz, 1H), 7.64 (ddd, J = 28.0, 21.1, 7.6 Hz, 3H), 7.23 (dd, J = 14.8, 7.0 Hz, 2H), 7.05 (t, J = 7.4 Hz, 1H), 6.70 (d, J = 7.7 Hz, 2H), 5.83 (s, 1H), 5.46 (d, J = 2.9 Hz, 1H), 5.30 (d, J = 3.2 Hz, 1H), 5.02 (dd, J = 10.4, 7.9 Hz, 1H), 4.83 (dd, J = 10.5, 3.4 Hz, 1H), 4.59 (d, J = 7.8 Hz, 1H), 4.28 – 3.93 (m, 6H), 3.85 (t, J = 6.7 Hz, 2H), 2.16 (s, 3H), 2.11 (s, 3H), 2.03 (s, 3H), 2.01 (s, 3H), 1.87 (s, 3H), 1.50 (s, 3H). ^{13}C NMR (100 MHz, $CDCl_3$) δ: 170.46, 170.32, 170.21, 169.99, 169.88, 168.73, 162.52, 143.25, 133.28, 132.06, 131.03, 129.73, 129.42, 128.65, 128.22, 125.97, 125.91, 125.19, 124.29, 122.46, 119.05, 100.78, 95.27, 77.16, 72.64, 71.48, 70.74, 70.70, 68.82, 67.65, 66.70, 62.26, 61.03, 20.67, 20.63, 20.62, 20.56, 20.40, 19.86. HR ESI-TOF MS (m/z): calcd for $C_{40}H_{42}F_6N_2O_{16}Na$ $[M + Na]^+$, 943.2331; found, 943.2336.

4.3 Preparation of glycosyl donor 18

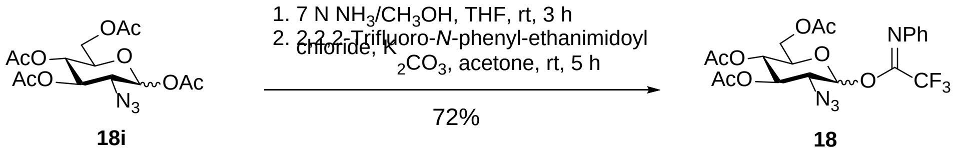

To a solution of **18i** (1.3 g, 3.48 mmol) in THF (20 mL) was added 7 N NH_3/CH_3OH (3 mL). After the reaction mixture was stirred at room temperature for 1 h, it was diluted with ethyl acetate and washed with 1 N HCl and brine. The organic layer was dried over Na_2SO_4, filtered and concentrated. The obtained residue was dissolved in anhydrous acetone (20 mL), 2,2,2-Trifluoro-*N*-phenyl-ethanimidoyl chloride (1.11 mL, 6.96 mmol) and K_2CO_3 (960 mg, 6.96 mmol) were added under N_2 protection. After 5 h stirring at room temperature, the mixture was filtered and concentrated in vacuum, and the residue was purified by Et_3N-neutralized silica gel column with EtOAc and hexanes (1:4) as the eluent to afford **18** (1.26 g, 72%) as a α/β mixture. R_f 0.50 (1:2, EtOAc–hexane); ^{1}H NMR (600 MHz, $CDCl_3$) δ: 7.32 (td, J = 7.9, 4.2 Hz, 2.5H), 7.14 (dt, J = 11.4, 5.7 Hz, 1.3H), 6.84 (t, J = 8.2 Hz, 2.6H), 6.48 (s, 1H), 5.62 (s, 1H), 5.50 (t, J = 9.9 Hz, 0.6H), 5.14 (t, J = 9.7 Hz, 1H), 5.06 (s, 1H), 4.36 – 4.22 (m, 1H), 4.20 – 4.03 (m, 1.5H), 3.87 – 3.65 (m, 2.3H), 2.11 (s, 3H), 2.10 (s, 2H), 2.09 (s, 3H), 2.07 (s, 2H), 2.06 (s, 3H), 2.02 (s, 2H). ^{13}C NMR (150 MHz, $CDCl_3$) δ: 170.44, 170.39, 169.77, 169.56, 169.48, 142.79, 128.83, 124.70, 119.19, 119.09, 95.23, 92.95, 72.70, 72.49, 70.59, 70.03, 67.74, 62.78, 61.37, 60.44, 20.61, 20.59, 20.52, 20.50. HR ESI-TOF MS (m/z): calcd for $C_{20}H_{21}F_3N_4O_8Na$ $[M + Na]^+$, 525.1204; found, 525.1213.

4.4 Preparation of glycosyl donor 19

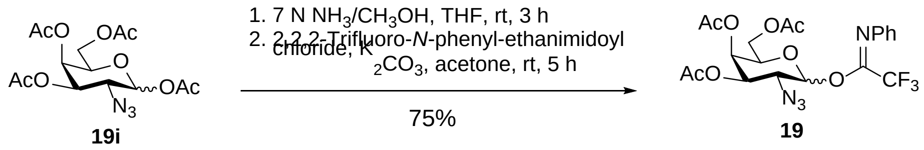

Compound **19** (678 mg, 75%) was prepared from **19i** (672 mg, 1.8 mmol) by the same procedure as the synthesis of **18**. R_f 0.55 (1:2, EtOAc–hexane); ^{1}H NMR (400 MHz, $CDCl_3$) of α/β anomers δ: 7.36 – 7.27 (m, 4.2H), 7.13 (t, J = 7.5 Hz, 2H), 6.84 (d, J = 7.8 Hz, 4H), 6.47 (s, 0.5H), 5.60 (s, 0.5H), 5.53 (s, 1H), 5.41 – 5.30 (m, 2H), 4.87 (d, J = 8.9 Hz, 1H), 4.31 (d, J = 6.0 Hz, 0.8H), 4.19 – 4.05 (m, 5H), 3.96 (dd, J = 24.4, 10.4 Hz, 3H), 2.18 (s, 3.4H), 2.16 (s, 2.7H), 2.08 (s, 2.7H), 2.07 (s, 3.4H), 2.05 (s, 2.7H), 2.00 (s, 3.4H). ^{13}C NMR (100 MHz, $CDCl_3$) δ: 170.21, 169.91, 169.58, 142.87, 128.83, 124.66, 119.23, 119.07, 95.47, 93.32, 71.75, 71.13, 69.10, 68.61, 66.83, 66.03,

61.20, 60.90, 59.86, 56.90, 20.58, 20.54, 20.52. HR ESI-TOF MS (m/z): calcd for $C_{20}H_{21}F_3N_4O_8Na$ [M + Na]$^+$, 525.1204; found, 525.1217.

5. Preparation of Thiol Nucleophiles

5.1 Preparation of cysteine-containing acceptors

General procedure for peptide coupling:

To a stirred solution of *N*-protected amino acid in dry DMF (0.5 M) was added HATU (1.1 eq), DIPEA (3.0 eq) followed by *C*-protected amino acid (1.05 eq) at room temperature under nitrogen atmosphere. The reaction mixture was stirred at same temperature for 3 to 6 h (TLC control). The reaction mixture was quenched with water and extracted with EtOAc (3×). The combined organic layer was dried over anhydrous Na_2SO_4 and evaporated to dryness. The obtained crude product was purified by flash chromatography.

General procedure for the removal of trityl protection:

The peptide was dissolved in dry dichloromethane (0.1 M) and purged with was argon gas for few minutes, then was added TIPS (2.0 eq) followed TFA (10.0 eq) dropwise under argon atmosphere at room temperature. The reaction mixture was allowed to stir at same temperature for 1-2 hours (TLC control). The reaction mixture was evaporated, to the obtained crude gummy crude product was added diethyl ether (10 vol). The solids precipitated were filtered using sintered funnel and washed again with diethyl ether to get pure product (no column purification required).

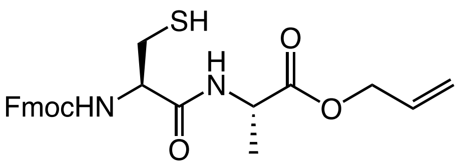

12

^{1}H NMR (CDCl$_3$, 400 MHz): δ 7.77 (d, *J* = 7.5 Hz, 2H), 7.59 (d, *J* = 7.3 Hz, 2H), 7.41 (t, *J* = 7.4 Hz, 2H), 7.32 (t, *J* = 7.4 Hz, 2H), 6.71 (d, *J* = 5.2 Hz, NH), 5.90 (ddt, *J* = 16.0, 10.6, 5.6 Hz, 1H), 5.72 (s, NH), 5.30 (dd, *J* = 26.5, 13.7 Hz, 2H), 4.70 – 4.53 (m, 3H), 4.53 – 4.32 (m, 3H), 4.23 (t, *J*

= 6.7 Hz, 1H), 3.12 – 2.98 (m, 1H), 2.74 (bs, 1H), 1.69 (t, *J* = 7.2 Hz, SH), 1.45 (d, *J* = 7.2 Hz, 3H).

^{13}C NMR ($CDCl_3$, 100 MHz): δ 172.1, 169.1, 143.6, 141.3, 141.30, 131.3, 127.8, 127.1, 125.0, 120.0, 120.0, 119.1, 67.2, 66.2, 56.0, 48.5, 47.1, 27.1, 18.2.

HRMS (ESI): calc. for $C_{24}H_{26}N_2O_5SNa$ $[M+Na]^+$: 477.1455; found: 477.1454.

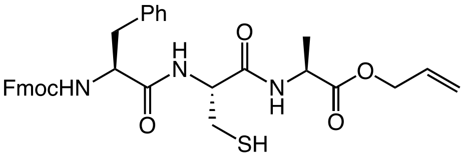

13

^{1}H NMR ($CDCl_3$, 400 MHz): δ 7.74 (d, *J* = 7.5 Hz, 2H), 7.50 (t, *J* = 7.8 Hz, 2H), 7.38 (t, *J* = 7.4 Hz, 2H), 7.34 – 7.12 (m, *7*H), 6.89 (d, *J* = 26.1 Hz, NH), 5.87 (ddt, *J* = 16.4, 10.9, 5.8 Hz, 1H), 5.42 – 5.19 (m, 2H, NH), 4.68 – 4.45 (m, 5H), 4.43 – 4.27 (m, 2H), 4.17 (t, *J* = 6.9 Hz, 1H), 3.21 – 2.95 (m, 3H), 2.73 – 2.61 (m, 1H), 1.63 (t, *J* = 7.2 Hz, SH), 1.38 (d, *J* = 6.6 Hz, 3H).

^{13}C NMR ($CDCl_3$, 100 MHz): δ 171.9, 170.9, 168.7, 143.6, 141.3, 135.9, 131.4, 129.2, 128.9, 128.8, 127.8, 127.3, 127.1, 124.9, 120.0, 118.8, 67.2, 66.1, 56.3, 54.2, 48.4, 47.0, 38.1, 26.6, 17.9.

HRMS (ESI): calc. for $C_{33}H_{35}N_3O_6SNa$ $[M+Na]^+$: 624.2139; found: 624.2134.

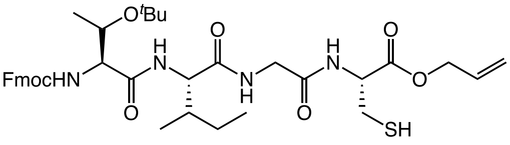

21

^{1}H NMR ($CDCl_3$, 400 MHz): δ 7.76 (d, *J* = 7.5 Hz, 2H), 7.61 (d, *J* = 7.4 Hz, 2H), 7.54 (d, *J* = 6.7 Hz, NH), 7.39 (t, *J* = 7.5 Hz, 2H), 7.30 (t, *J* = 7.4 Hz, 2H), 6.92 (bd, NH), 6.12 (m, NH), 5.94 – 5.80 (m, 1H), 5.36 – 5.18 (m, 2H), 4.87 (td, *J* = 12.5, 4.9 Hz, 1H), 4.64 (d, *J* = 4.9 Hz, 2H), 4.44 – 4.34 (m, 2H), 4.33 – 4.06 (m, 5H), 3.88 (ddd, *J* = 34.4, 16.8, 5.3 Hz, 1H), 3.08 – 2.98 (m, 2H), 2.05 – 1.93 (m, 1H), 1.84 – 1.69 (m, 1H), 1.65 – 1.50 (m, 2H), 1.33 (s, 9H), 1.21 – 1.04 (m, 4H), 1.04 – 0.86 (m, 6H).

^{13}C NMR ($CDCl_3$, 100 MHz): δ 171.7, 170.4, 170.1, 169.5, 156.1, 143.8, 143.62, 141.3, 131.3, 131.3, 127.7, 127.0, 125.1, 120.1, 119.2, 76.0, 67.2, 66.9, 66.4, 59.3, 58.4, 54.1, 47.2, 43.2, 36.3, 28.2, 26.6, 25.04, 16.8, 15.8, 11.4.

HRMS (ESI): calc. for $C_{37}H_{51}N_4O_8S$ $[M+H]^+$: 711.3432; found: 711.3435.

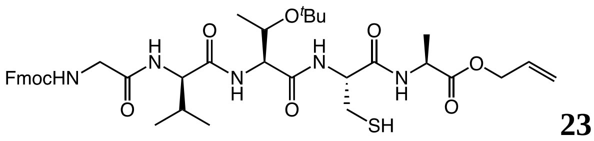

23

^{1}H NMR ($CDCl_3$, 400 MHz): δ 8.22 (s, NH), 7.94 (s, NH), 7.74 (d, *J* = 7.4 Hz, 2H), 7.57 (d, *J* = 7.2 Hz, 2H), 7.37 (t, *J* = 7.3 Hz, 2H), 7.27 (d, *J* = 9.6 Hz, 2H), 7.05 (s, NH), 6.12 (s, NH), 5.90 – 5.73 (m, 1H), 5.21 (dd, *J* = 26.3, 13.8 Hz, 2H), 5.06 (s, 1H), 4.83 – 4.73 (m, 1H), 4.65 – 4.51 (m, 3H), 4.45 – 4.15 (m, 3H), 4.08 – 3.87 (m, 3H), 3.09 – 2.98 (m, 1H), 2.87 – 2.72 (m, 1H), 2.02 (dd, *J* = 12.9, 6.6 Hz, 1H), 1.65 (t, *J* = 7.4 Hz, SH), 1.37 (d, *J* = 7.1 Hz, 3H), 1.25 (s, 9H), 1.05 (d, *J* = 5.5 Hz, 3H), 0.93 (d, *J* = 6.3 Hz, 6H).

^{13}C NMR ($CDCl_3$, 100 MHz): δ 172.2, 170.8, 169.6, 169.3, 168.9, 156.7, 143.8, 141.2, 131.5, 127.7, 127.0, 125.1, 119.9, 118.7, 75.4, 67.3, 66.1, 61.5, 58.1, 57.6, 53.7, 48.2, 47.0, 44.4, 32.1, 29.3, 28.3, 19.0, 18.5, 18.1.

HRMS (ESI): calc. for $C_{39}H_{54}N_5O_9S$ $[M+H]^+$: 768.5.

5.2 Preparation of glucosyl acceptor 14

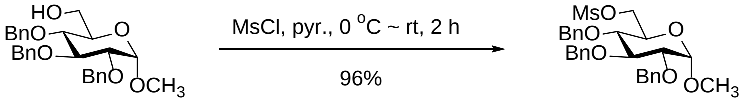

14i **14ii**

To a solution of **14i** (2.7 g, 5.82 mmol) in 30 mL of anhydrous pyridine was added MsCl (901 µL, 11.64 mmol) under N_2 protection at 0 °C. After 2 hours of stirring at the same temperature, the reaction mixture was quenched with methanol and concentrated. The residue was subjected to silica gel column chromatography with EtOAc and hexanes (1:3) as the eluent to afford compound **26** (3.03 g, 96%) as a syrup. R_f 0.45 (1:2, EtOAc–hexane); ^{1}H NMR (600 MHz, $CDCl_3$) *δ*: 7.43 – 7.21 (m, 1H), 5.01 (d, *J* = 10.9 Hz, 1H), 4.91 (d, *J* = 10.8 Hz, 1H), 4.81 (dd, *J* = 21.3, 11.5 Hz, 1H), 4.70 – 4.54 (m, 1H), 4.36 (qd, *J* = 11.0, 3.2 Hz, 1H), 4.02 (t, *J* = 9.2 Hz, 1H), 3.84 (ddd, *J* = 10.1, 4.1, 2.2 Hz, 1H), 3.56 – 3.45 (m, 1H), 3.38 (s, 1H), 2.97 (s, 1H). ^{13}C NMR (150 MHz, $CDCl_3$) *δ*: 138.48, 137.92, 137.73, 128.51, 128.50, 128.43, 128.07, 128.06, 128.00, 127.97, 127.89, 127.69, 98.16, 81.79, 79.76, 76.93, 75.75, 75.11, 73.46, 68.62, 68.39, 55.45, 37.51. HR ESI-TOF MS (m/z): calcd for $C_{29}H_{34}SO_8Na$ $[M + Na]^+$, 565.1867; found, 565.1878.

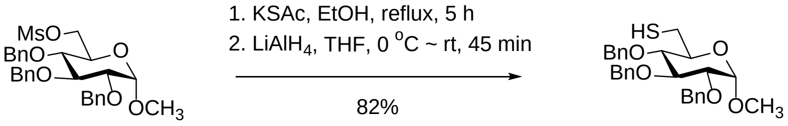

14ii **14**

To a solution of **14ii** (3.3 g, 6.09 mmol) in ethanol (60 mL) was added KSAc (2.08 g, 18.27 mmol). After it was stirred under reflux for 5 h, it was diluted with ethyl acetate and washed with aqueous $NaHCO_3$ and brine. The organic layer was dried over Na_2SO_4, filtered and concentrated. The residue was subjected to flush silica gel column chromatography (1:6, EtOAc–hexane) to give an intermediate. To the obtained syrup in THF (60 mL) was added $LiAlH_4$ (1.85 g, 48.7 mmol) at 0 ºC. After it was stirred at rt for 45 min, it was quenched with addition of ethyl acetate. The mixture was diluted with CH_2Cl_2 and washed with 1 N HCl and brine. The organic layer was dried over Na_2SO_4, filtered and concentrated. The residue was subjected to flush silica gel column chromatography (1:8, EtOAc–hexane) to give **14** (2.4 g, 82%) as a syrup. R_f 0.65 (1:4, EtOAc–hexane); ^{1}H NMR (600 MHz, $CDCl_3$) δ: 7.44 – 7.16 (m, 1H), 5.01 (d, J = 10.8 Hz, 1H), 4.92 (d, J = 11.1 Hz, 1H), 4.86 – 4.77 (m, 1H), 4.71 – 4.56 (m, 1H), 4.00 (t, J = 9.2 Hz, 1H), 3.73 (ddd, J = 9.7, 7.1, 2.6 Hz, 1H), 3.53 (dd, J = 9.6, 3.6 Hz, 1H), 3.45 (t, J = 9.3 Hz, 1H), 3.41 (s, 1H), 2.87 (ddd, J = 13.8, 8.7, 2.7 Hz, 1H), 2.65 – 2.57 (m, 1H), 1.52 (t, J = 8.3 Hz, 1H).
^{13}C NMR (150 MHz, $CDCl_3$) δ: 138.64, 138.08, 128.47, 128.46, 128.40, 128.08, 127.96, 127.93, 127.86, 127.63, 97.93, 81.99, 80.13, 79.67, 75.72, 75.07, 73.37, 70.69, 55.21, 26.39. HR ESI-TOF MS (m/z): calcd for $C_{28}H_{32}SO_5Na$ $[M + Na]^+$, 503.1863; found, 503.1859.

5.3 Preparation of glucosyl acceptor 15

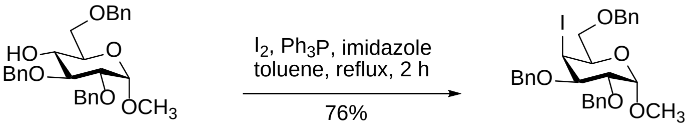

15i **15ii**

To a solution of **15i** (1.6 g, 3.45 mmol) in toluene (60 mL) were added I_2 (1.75 g, 6.9 mmol), imidazole (703 mg, 10.35 mmol) and Ph_3P (2.71 g, 10.35 mmol). After it was stirred under reflux for 2 h, it was filtered and concentrated. The residue was subjected to flush silica gel column chromatography (1:10, EtOAc–hexane) to give **15ii** (3.34 g, 76%) as a syrup. R_f 0.70 (1:3, EtOAc–hexane); ^{1}H NMR (400 MHz, $CDCl_3$) δ: 7.54 – 7.12 (m, 1H), 4.86 (d, J = 12.0 Hz, 1H), 4.75 (d, J = 11.6 Hz, 1H), 4.71 – 4.52 (m, 1H), 3.85 (dd, J = 9.5, 3.8 Hz, 1H), 3.64 (dd, J = 9.6, 6.0 Hz, 1H),

3.52 (dd, J = 9.6, 6.4 Hz, 1H), 3.39 (s, 1H), 3.31 (t, J = 6.1 Hz, 1H), 3.21 (dd, J = 9.5, 4.0 Hz, 1H). ^{13}C NMR (100 MHz, $CDCl_3$) δ: 138.34, 137.93, 137.80, 128.43, 128.39, 128.35, 128.12, 127.81, 127.78, 127.75, 127.68, 99.04, 77.84, 75.71, 74.18, 73.96, 73.74, 71.24, 67.08, 55.34, 41.23. HR ESI-TOF MS (m/z): calcd for $C_{28}H_{31}IO_5Na$ $[M + Na]^+$, 597.1108; found, 597.1106.

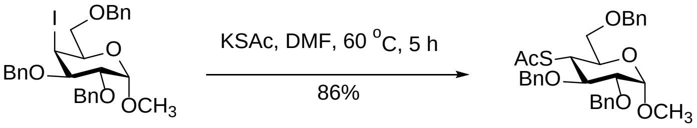

15ii **15iii**

To a solution of **15ii** (1.5 g, 2.61 mmol) in DMF (30 mL) was added KSAc (1.19 g, 10.45 mmol). After it was stirred at 60 °C for 5 h, it was diluted with ethyl acetate and washed with H_2O and brine. The organic layer was dried over Na_2SO_4, filtered and concentrated. The residue was subjected to flush silica gel column chromatography (1:6, EtOAc–hexane) to give **15iii** (1.17 g, 86%) as a syrup. R_f 0.40 (1:4, EtOAc–hexane); ^{1}H NMR (600 MHz, $CDCl_3$) δ: 7.39 – 7.17 (m, 1H), 4.93 (d, J = 11.1 Hz, 1H), 4.79 (d, J = 12.0 Hz, 1H), 4.73 – 4.62 (m, 1H), 4.52 (s, 1H), 3.93 – 3.88 (m, 1H), 3.84 (dd, J = 10.6, 9.5 Hz, 1H), 3.68 (t, J = 11.1 Hz, 1H), 3.65 – 3.58 (m, 1H), 3.41 (s, 1H), 2.24 (s, 1H). ^{13}C NMR (150 MHz, $CDCl_3$) δ: 193.42, 138.63, 138.07, 138.03, 128.44, 128.23, 128.19, 128.10, 127.91, 127.71, 127.63, 127.50, 127.42, 98.33, 97.44, 80.93, 78.34, 75.93, 73.45, 73.35, 69.87, 69.82, 55.39, 45.80, 30.61. HR ESI-TOF MS (m/z): calcd for $C_{30}H_{34}SO_6Na$ $[M + Na]^+$, 545.1968; found, 545.1963.

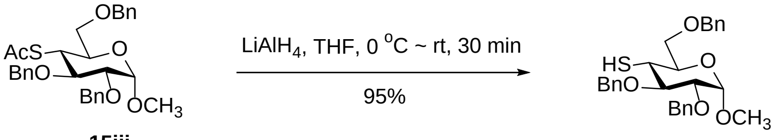

15iii **15**

To a solution of **15iii** (590 mg, 1.13 mmol) in THF (20 mL) was added $LiAlH_4$ (344 mg, 9.04 mmol) at 0 °C. After it was stirred at rt for 30 min, it was quenched with addition of ethyl acetate. The mixture was diluted with CH_2Cl_2 and washed with 1 N HCl and brine. The organic layer was dried over Na_2SO_4, filtered and concentrated. The residue was subjected to flush silica gel column chromatography (1:7, EtOAc–hexane) to give **15** (515 mg, 95%) as a syrup. R_f 0.45 (1:4, EtOAc–hexane); ^{1}H NMR (600 MHz, $CDCl_3$) δ: 7.54 – 7.21 (m, 1H), 4.99 (d, J = 10.6 Hz, 1H), 4.82 (dd, J = 24.7, 11.3 Hz, 1H), 4.72 – 4.61 (m, 1H), 4.52 (d, J = 12.1 Hz, 1H), 3.80 (dd, J = 10.7, 3.9 Hz, 1H), 3.72 (ddd, J = 19.2, 12.0, 5.9 Hz, 1H), 3.55 (dd, J = 9.3, 3.5 Hz, 1H), 3.41 (s, 1H), 3.09 (td, J = 10.5, 6.7 Hz, 1H), 1.67 (d, J = 6.7 Hz, 1H). ^{13}C NMR (150 MHz, $CDCl_3$) δ: 138.41, 138.05,

137.95, 128.46, 128.40, 128.34, 128.19, 128.10, 127.94, 127.76, 127.73, 127.65, 98.53, 81.81, 80.64, 76.29, 73.56, 73.20, 72.30, 69.49, 55.39, 41.89. HR ESI-TOF MS (m/z): calcd for $C_{28}H_{32}SO_5Na$ $[M + Na]^+$, 503.1863; found, 503.1857.

5.4 Preparation of thiol-Farnesol acceptor 16

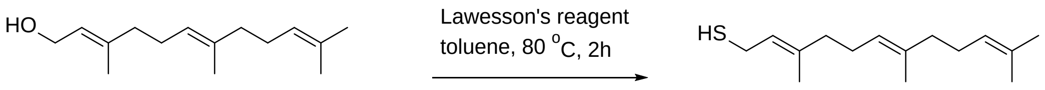

trans,trans-Farnesol **16**

To a solution of **trans,trans-Farnesol** (0.4 mL, 1.58 mmol) in dry toluene (5 mL) was added Lawesson's reagent (0.42 g, 0.95 mmol). After it was stirred at 80 ºC for 2 h, it was cooled down, filtered and concentrated. The residue was subjected to flush silica gel column chromatography (hexane) to give **16** (316 mg, 84%) as a colorless oil. R_f 0.25 (hexane); ^{1}H NMR (600 MHz, $CDCl_3$) δ: 5.34 (td, J = 7.8, 1.1 Hz, 1H), 5.12 – 5.06 (m, 2H), 3.16 (t, J = 7.4 Hz, 2H), 2.12 – 1.94 (m, 8H), 1.68 (s, 3H), 1.66 (s, 2H), 1.60 (s, 5H), 1.39 (t, J = 7.1 Hz, 1H). ^{13}C NMR (150 MHz, $CDCl_3$) δ: 137.47, 135.27, 131.26, 124.31, 124.25, 124.09, 123.72, 123.61, 123.28, 39.67, 39.37, 26.70, 26.27, 25.67, 22.09, 17.66, 16.00, 15.75. HR ESI-TOF MS (m/z): calcd for $C_{15}H_{26}SNa$ $[M + Na]^+$, 261.1647; found, 261.1648.

6. Scope of Glycosylation

General Procedure for Triflic Acid-Catalyzed Thiol Glycosylation:

A 10 mL Schlenk flask was charged with *N*-phenyl trifluoroacetimidate glycosyl donor **1, 6 – 9, 18, 19** (0.2 mmol, 1 – 2 equiv.), thiol acceptor **10-17** (0.1 mmol, 1 equiv.) and dichloromethane (1 mL). The resulting solution was stirred at room temperature for 2 min under a nitrogen atmosphere before the TfOH (5 mol%) was added. After 1 h of stirring at room temperature, the reaction was quenched with 1 drop of Et_3N and concentrated. A crude ^{1}H NMR was taken to determine the (α/β) ratio. Further purification by silica gel column chromatography (ethyl acetate/hexane: 1/4→1/2) was performed to give the desired product.

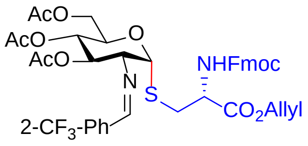

1a: 76%, α:β > 20:1

^{1}H NMR ($CDCl_3$, 600 MHz): δ 8.61 (d, *J* = 2.0 Hz, 1H), 8.20 (d, *J* = 7.7 Hz, 1H), 7.76 (d, *J* = 7.6 Hz, 2H), 7.68 (d, *J* = 7.6 Hz, 1H), 7.64 – 7.51 (m, 4H), 7.40 (dd, *J* = 14.0, 7.0 Hz, 2H), 7.31 (dd, *J* = 15.2, 7.6 Hz, 2H), 6.01 (d, *J* = 8.6 Hz, 1H), 5.87 (ddd, *J* = 16.5, 11.2, 5.9 Hz, 1H), 5.49 (t, *J* = 9.7 Hz, 1H), 5.25 (ddd, *J* = 17.4, 11.5, 9.1 Hz, 3H), 5.10 (dd, *J* = 10.1, 9.6 Hz, 1H), 4.78 – 4.70 (m, 1H), 4.64 (d, *J* = 5.8 Hz, 2H), 4.52 (dd, *J* = 10.2, 3.2 Hz, 1H), 4.42 (p, *J* = 10.6 Hz, 2H), 4.25 (ddd, *J* = 12.5, 11.2, 6.2 Hz, 3H), 3.92 (dd, *J* = 10.0, 5.6 Hz, 1H), 3.23 (dd, *J* = 14.2, 5.4 Hz, 1H), 3.01 (dd, *J* = 14.3, 4.0 Hz, 1H), 2.05 (s, 3H), 2.03 (s, 3H), 1.87 (s, 3H).

^{13}C NMR (CDCl3, 150 MHz): δ 170.50, 169.87, 169.73, 155.69, 143.85, 143.62, 141.31, 137.07, 131.26, 128.51, 128.14, 127.79, 127.73, 127.71, 127.04, 125.07, 124.98, 119.99, 119.97, 119.34, 85.32, 76.11, 72.50, 71.86, 68.41, 67.07, 66.43, 62.07, 54.18, 47.05, 34.58, 20.73, 20.61, 20.58.

HRMS (ESI): calc. for $C_{41}H_{41}F_3N_2O_{11}SNa$ $[M + Na]^+$, 849.2275; found: 849.2278.

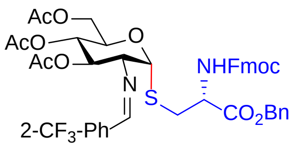

1b: 71%, α:β > 20:1

^{1}H NMR (CDCl$_3$, 600 MHz): δ 8.59 (d, *J* = 1.9 Hz, 1H), 8.18 (d, *J* = 7.7 Hz, 1H), 7.76 (d, *J* = 7.5 Hz, 2H), 7.68 (d, *J* = 7.6 Hz, 1H), 7.63 – 7.51 (m, 4H), 7.40 (dd, *J* = 13.5, 7.0 Hz, 2H), 7.36 – 7.27 (m, 8H), 6.05 (d, *J* = 8.6 Hz, 1H), 5.46 (t, *J* = 9.7 Hz, 1H), 5.16 (dd, *J* = 12.4, 3.4 Hz, 3H), 5.10 – 5.04 (m, 1H), 4.80 – 4.73 (m, 1H), 4.43 (ddt, *J* = 17.3, 13.7, 6.8 Hz, 3H), 4.28 – 4.16 (m, 3H), 3.85 (dd, *J* = 10.0, 5.6 Hz, 1H), 3.23 (dd, *J* = 14.3, 5.2 Hz, 1H), 2.99 (dd, *J* = 14.3, 4.0 Hz, 1H), 2.04 (s, 3H), 2.01 (s, 3H), 1.87 (s, 3H).

^{13}C NMR (CDCl3, 150 MHz): δ 170.55, 169.84, 169.56, 160.34, 155.73, 143.63, 141.27, 134.86, 132.28, 130.90, 129.09, 128.62, 128.59, 128.57, 127.71, 127.69, 127.06, 127.03, 125.49, 125.04, 119.97, 119.94, 85.35, 72.02, 71.29, 68.63, 67.65, 67.07, 62.18, 54.15, 53.77, 47.10, 33.77, 20.66, 20.59, 20.33.

HRMS (ESI): calc. for $C_{45}H_{43}F_3N_2O_{11}SNa$ [M + Na]$^+$, 899.2432; found: 899.2433.

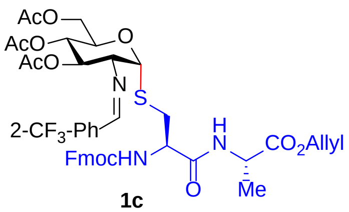

1c

75%, α:β > 20:1

^{1}H NMR (CDCl$_3$, 600 MHz): δ 8.62 (d, *J* = 1.7 Hz, 1H), 8.18 (d, *J* = 7.6 Hz, 1H), 7.75 (d, *J* = 7.5 Hz, 2H), 7.66 (d, *J* = 7.6 Hz, 1H), 7.54 (ddd, *J* = 24.2, 15.3, 7.5 Hz, 4H), 7.38 (dd, *J* = 12.8, 7.2 Hz, 2H), 7.30 (ddd, *J* = 16.4, 7.6, 1.0 Hz, 2H), 6.84 (d, *J* = 5.7 Hz, 1H), 5.88 (ddd, *J* = 16.3, 11.0, 5.7 Hz, 1H), 5.71 (s, 1H), 5.52 (t, *J* = 9.7 Hz, 1H), 5.41 (s, 1H), 5.36 – 5.29 (m, 1H), 5.24 (dd, *J* = 10.5, 1.1 Hz, 1H), 5.11 (t, *J* = 9.8 Hz, 1H), 4.67 – 4.50 (m, 4H), 4.43 (dt, *J* = 17.0, 10.2 Hz, 3H), 4.25 (ddd, *J* = 24.6, 13.0, 5.8 Hz, 3H), 3.91 (dd, *J* = 9.9, 5.6 Hz, 1H), 3.14 – 3.06 (m, 1H), 2.93 – 2.85 (m, 1H), 2.06 (s, 3H), 2.02 (s, 3H), 1.86 (s, 3H), 1.38 (d, *J* = 7.2 Hz, 3H).

^{13}C NMR (CDCl3, 150 MHz): δ 171.96, 170.69, 169.87, 169.58, 160.45, 155.90, 143.69, 143.59, 141.29, 141.26, 133.05, 132.21, 131.44, 130.89, 129.09, 129.04, 127.75, 127.72, 127.10, 127.06, 125.50, 125.46, 125.00, 124.91, 119.98, 119.96, 118.81, 85.36, 71.99, 71.47, 68.82, 68.45, 67.19, 66.01, 62.16, 55.00, 48.39, 47.07, 33.38, 20.72, 20.65, 20.34, 18.00.

HRMS (ESI): calc. for $C_{44}H_{46}F_3N_3O_{12}SNa$ [M + Na]$^+$, 920.2647; found: 920.2643.

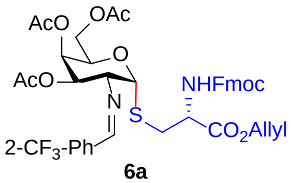

6a

73%, α:β > 20:1

^{1}H NMR (CDCl$_3$, 600 MHz): δ 8.63 (s, 1H), 8.19 (d, *J* = 7.7 Hz, 1H), 7.76 (d, *J* = 7.6 Hz, 2H), 7.68 (d, *J* = 7.7 Hz, 1H), 7.58 (ddd, *J* = 33.7, 14.2, 7.5 Hz, 4H), 7.40 (dd, *J* = 13.5, 7.0 Hz, 2H), 7.32 (dt, *J* = 15.0, 7.6 Hz, 2H), 5.96 (d, *J* = 8.5 Hz, 1H), 5.92 – 5.82 (m, 1H), 5.48 (s, 1H), 5.40 – 5.27 (m, 3H), 5.20 (d, *J* = 10.5 Hz, 1H), 4.72 – 4.60 (m, 3H), 4.40 (d, *J* = 7.1 Hz, 2H), 4.26 – 4.06 (m, 4H), 3.22 (dd, *J* = 14.0, 5.4 Hz, 1H), 3.00 (dd, *J* = 14.0, 4.0 Hz, 1H), 2.21 (s, 3H), 2.04 (s, 3H), 1.87 (s, 3H).

^{13}C NMR (CDCl3, 150 MHz): δ 170.46, 170.03, 169.62, 160.33, 155.67, 143.67, 141.29, 132.22, 131.30, 130.77, 129.03, 127.69, 127.04, 125.04, 119.96, 119.11, 85.43, 69.13, 67.62, 67.12, 66.79, 66.36, 62.18, 54.10, 47.07, 32.99, 29.67, 20.75, 20.61, 20.33.

HRMS (ESI): calc. for $C_{41}H_{41}F_3N_2O_{11}SNa$ [M + Na]$^+$, 849.2275; found: 849.2273.

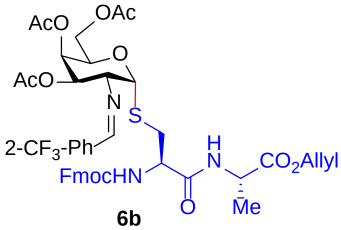

6b

68%, α:β > 20:1

^{1}H NMR (CDCl$_3$, 600 MHz): δ 8.63 (d, *J* = 1.9 Hz, 1H), 8.18 (d, *J* = 7.7 Hz, 1H), 7.75 (d, *J* = 7.5 Hz, 2H), 7.66 (d, *J* = 7.6 Hz, 1H), 7.62 – 7.48 (m, 4H), 7.39 (dd, *J* = 15.2, 7.6 Hz, 2H), 7.36 – 7.28 (m, 2H), 6.97 (d, *J* = 6.0 Hz, 1H), 5.94 – 5.76 (m, 2H), 5.48 (d, *J* = 2.4 Hz, 2H), 5.31 (ddd, *J* = 56.3, 24.8, 6.8 Hz, 3H), 4.67 – 4.50 (m, 4H), 4.40 (dd, *J* = 31.9, 5.9 Hz, 3H), 4.21 (t, *J* = 6.9 Hz, 1H), 4.16 (d, *J* = 6.0 Hz, 2H), 4.10 (dd, *J* = 10.4, 5.5 Hz, 1H), 3.15 (dd, *J* = 13.5, 5.7 Hz, 1H), 2.93 – 2.85 (m, 1H), 2.19 (s, 3H), 2.00 (s, 3H), 1.87 (s, 3H), 1.38 (d, *J* = 7.0 Hz, 3H).

^{13}C NMR (CDCl3, 150 MHz): δ 172.00, 170.56, 170.08, 169.66, 169.60, 160.40, 155.90, 143.73, 143.61, 141.29, 141.27, 133.32, 132.18, 131.44, 130.74, 129.15, 129.04, 128.94, 127.74, 127.72, 127.09, 127.05, 125.48, 125.44, 125.01, 124.96, 123.15, 119.98, 119.97, 118.79, 85.31, 69.47,

69.24, 67.44, 67.11, 67.03, 66.84, 65.98, 62.22, 53.78, 48.35, 47.06, 32.54, 20.76, 20.63, 20.34, 18.08.

HRMS (ESI): calc. for $C_{44}H_{46}F_3N_3O_{12}SNa$ [M + Na]$^+$, 920.2647; found: 920.2641.

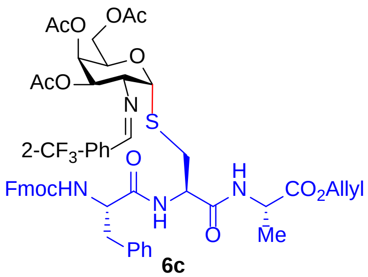

6c

78%, α:β > 20:1

^{1}H NMR ($CDCl_3$, 600 MHz): δ 8.63 (d, *J* = 1.7 Hz, 1H), 8.16 (d, *J* = 7.6 Hz, 1H), 7.74 (dd, *J* = 17.5, 5.8 Hz, 2H), 7.66 (d, *J* = 7.5 Hz, 1H), 7.55 – 7.46 (m, 4H), 7.39 (dd, *J* = 13.4, 6.9 Hz, 2H), 7.33 – 7.23 (m, 5H), 7.18 (s, 2H), 6.92 (s, 1H), 6.81 (s, 1H), 5.93 – 5.83 (m, 1H), 5.46 (dd, *J* = 9.3, 3.8 Hz, 2H), 5.32 (ddd, *J* = 9.6, 9.0, 2.2 Hz, 3H), 5.24 (dd, *J* = 10.5, 1.1 Hz, 1H), 4.66 – 4.41 (m, 7H), 4.31 – 4.12 (m, 4H), 4.05 (dd, *J* = 10.5, 5.5 Hz, 1H), 3.07 (d, *J* = 36.9 Hz, 3H), 2.87 (d, *J* = 13.3 Hz, 1H), 2.16 (s, 3H), 2.04 (s, 3H), 1.85 (s, 3H), 1.35 (d, *J* = 7.0 Hz, 3H).

^{13}C NMR (CDCl3, 150 MHz): δ 171.90, 170.68, 170.09, 169.59, 169.07, 160.44, 143.72, 141.25, 133.56, 132.18, 131.49, 130.74, 129.30, 129.18, 129.03, 128.78, 127.72, 127.21, 127.05, 124.97, 119.97, 119.94, 118.76, 84.73, 69.23, 67.46, 67.15, 67.02, 66.72, 65.96, 62.11, 53.77, 48.38, 47.04, 31.47, 29.25, 20.73, 20.71, 20.32, 17.89.

HRMS (ESI): calc. for $C_{53}H_{55}F_3N_4O_{13}SNa$ [M + Na]$^+$, 1067.3331; found: 1067.3337.

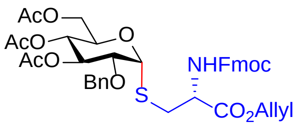

7a: 93%, α:β > 20:1

^{1}H NMR ($CDCl_3$, 600 MHz): δ 7.76 (dd, *J* = 7.5, 0.7 Hz, 2H), 7.59 (dd, *J* = 19.0, 7.5 Hz, 2H), 7.40 (q, *J* = 7.4 Hz, 2H), 7.35 – 7.26 (m, 5H), 6.06 (d, *J* = 8.7 Hz, 1H), 5.91 (ddd, *J* = 16.5, 11.2, 5.9 Hz, 1H), 5.34 (d, *J* = 17.2 Hz, 1H), 5.24 (ddd, *J* = 19.2, 9.9, 2.1 Hz, 3H), 4.98 – 4.90 (m, 1H), 4.78 – 4.70 (m, 1H), 4.64 (dd, *J* = 22.9, 9.0 Hz, 3H), 4.55 (d, *J* = 12.2 Hz, 1H), 4.40 (dd, *J* = 7.0,

1.7 Hz, 2H), 4.35 – 4.26 (m, 1H), 4.24 – 4.12 (m, 3H), 3.80 (dd, *J* = 10.0, 5.5 Hz, 1H), 3.25 (dd, *J* = 14.5, 5.0 Hz, 1H), 2.90 (dd, *J* = 14.5, 3.8 Hz, 1H), 2.02 (s, 3H), 2.01 (s, 3H), 2.00 (s, 3H).

^{13}C NMR (CDCl3, 150 MHz): δ 170.50, 169.94, 169.87, 169.73, 155.69, 143.85, 143.62, 141.31, 137.07, 131.26, 128.51, 128.14, 127.79, 127.73, 127.71, 127.04, 125.07, 124.98, 119.99, 119.97, 119.34, 85.32, 76.11, 72.50, 71.86, 68.41, 67.07, 66.43, 62.07, 54.18, 47.05, 34.58, 20.73, 20.61, 20.58.

HRMS (ESI): calc. for $C_{40}H_{43}NO_{12}SNa$ $[M + Na]^+$, 784.2398; found: 784.2392.

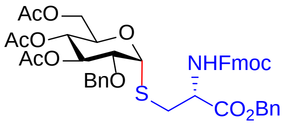

7b: 95%, α:β > 20:1

^{1}H NMR (CDCl$_3$, 600 MHz): δ 7.76 (d, *J* = 7.5 Hz, 2H), 7.59 (dd, *J* = 18.7, 7.5 Hz, 2H), 7.46 – 7.23 (m, 14H), 6.12 (d, *J* = 8.8 Hz, 1H), 5.17 (dt, *J* = 23.4, 5.9 Hz, 4H), 4.92 (t, *J* = 9.8 Hz, 1H), 4.76 (dt, *J* = 8.6, 4.2 Hz, 1H), 4.59 (d, *J* = 12.3 Hz, 1H), 4.51 (d, *J* = 12.3 Hz, 1H), 4.40 (dd, *J* = 6.9, 4.2 Hz, 2H), 4.31 – 4.08 (m, 4H), 3.76 (dd, *J* = 10.0, 5.6 Hz, 1H), 3.26 (dd, *J* = 14.5, 4.9 Hz, 1H), 2.88 (dd, *J* = 14.5, 3.7 Hz, 1H), 2.03 (s, 3H), 2.01 (s, 3H), 1.97 (s, 3H).

^{13}C NMR (CDCl3, 150 MHz): δ 170.50, 170.11, 169.86, 169.72, 155.71, 143.87, 143.62, 141.31, 141.27, 137.08, 134.84, 128.70, 128.65, 128.64, 128.51, 128.14, 127.80, 127.73, 127.71, 127.05, 125.08, 124.98, 119.99, 119.97, 85.30, 72.50, 71.82, 68.42, 68.39, 67.72, 67.08, 62.06, 54.20, 47.05, 34.68, 20.73, 20.63, 20.54.

HRMS (ESI): calc. for $C_{44}H_{45}NO_{12}SNa$ $[M + Na]^+$, 834.2555; found: 834.2565.

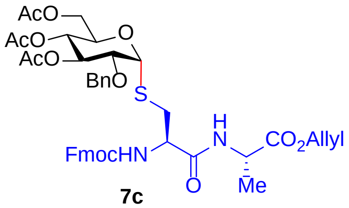

7c

92%, α:β > 20:1

^{1}H NMR (CDCl$_3$, 600 MHz): δ 7.76 (dd, *J* = 7.5, 2.2 Hz, 2H), 7.59 (t, *J* = 8.0 Hz, 2H), 7.40 (dd, *J* = 13.1, 7.3 Hz, 2H), 7.30 (qdd, *J* = 20.3, 10.7, 6.6 Hz, 7H), 6.86 (d, *J* = 6.4 Hz, 1H), 5.96 – 5.79 (m, 2H), 5.47 (d, *J* = 5.3 Hz, 1H), 5.39 – 5.20 (m, 3H), 4.95 (t, *J* = 9.8 Hz, 1H), 4.73 – 4.48 (m, 5H), 4.47 – 4.34 (m, 3H), 4.34 – 4.26 (m, 1H), 4.26 – 4.17 (m, 2H), 4.12 (dd, *J* = 14.2, 7.1 Hz,

1H), 3.83 (dd, *J* = 9.0, 5.2 Hz, 1H), 3.11 (d, *J* = 9.6 Hz, 1H), 2.81 (dd, *J* = 13.6, 4.9 Hz, 1H), 2.02 (s, 3H), 2.01 (s, 3H), 2.00 (s, 3H), 1.37 (d, *J* = 7.1 Hz, 3H).

^{13}C NMR (CDCl3, 150 MHz): δ 171.92, 170.58, 169.85, 169.73, 169.40, 155.86, 143.69, 143.58, 141.31, 141.28, 136.96, 131.49, 128.57, 128.51, 128.26, 128.17, 128.01, 127.91, 127.77, 127.75, 127.65, 127.59, 127.11, 127.08, 125.00, 120.01, 119.99, 118.81, 85.20, 76.21, 72.70, 72.14, 68.52, 68.43, 67.16, 66.01, 62.06, 54.94, 48.42, 47.06, 33.95, 20.75, 20.68, 20.61, 17.92.

HRMS (ESI): calc. for $C_{43}H_{48}N_2O_{13}SNa$ [M + Na]$^+$, 855.2769; found: 855.2764.

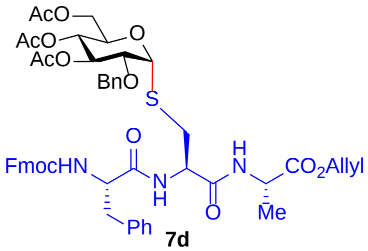

7d

87%, α:β > 20:1

^{1}H NMR ($CDCl_3$, 600 MHz): δ 7.75 (d, *J* = 7.5 Hz, 2H), 7.50 (dd, *J* = 13.9, 7.5 Hz, 2H), 7.42 – 7.36 (m, 2H), 7.36 – 7.22 (m, 10H), 7.18 (s, 2H), 6.85 (d, *J* = 38.5 Hz, 2H), 5.94 – 5.84 (m, 1H), 5.56 (d, *J* = 4.3 Hz, 1H), 5.27 (ddd, *J* = 13.8, 12.1, 1.3 Hz, 4H), 4.95 (t, *J* = 9.7 Hz, 1H), 4.71 – 4.38 (m, 8H), 4.36 – 4.11 (m, 5H), 3.81 (dd, *J* = 9.9, 5.5 Hz, 1H), 3.18 – 2.96 (m, 3H), 2.88 – 2.77 (m, 1H), 2.05 (s, 3H), 2.01 (s, 3H), 1.99 (s, 3H), 1.34 (d, *J* = 6.7 Hz, 3H).

^{13}C NMR (CDCl3, 150 MHz): δ 171.79, 170.87, 170.68, 169.88, 169.73, 168.83, 143.68, 141.26, 136.97, 135.96, 131.53, 129.22, 128.82, 128.50, 128.14, 127.87, 127.72, 127.26, 127.08, 124.96, 119.97, 118.77, 84.81, 76.25, 72.66, 72.19, 68.61, 68.45, 67.11, 65.98, 61.92, 53.43, 48.45, 47.05, 38.05, 33.11, 20.74, 20.62, 17.70.

HRMS (ESI): calc. for $C_{38}H$ $C_{52}H_{57}N_3O_{14}SNa$ [M + Na]$^+$, 1002.3453; found: 1002.3458.

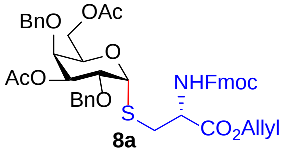

8a

86%, α:β > 20:1

^{1}H NMR ($CDCl_3$, 600 MHz): δ 7.73 (d, *J* = 7.5 Hz, 2H), 7.60 (dd, *J* = 17.0, 7.5 Hz, 2H), 7.44 – 7.21 (m, 14H), 6.16 (d, *J* = 8.8 Hz, 1H), 5.97 – 5.83 (m, 1H), 5.40 – 5.20 (m, 3H), 5.07 (dd, *J* = 10.5, 2.9 Hz, 1H), 4.77 – 4.61 (m, 5H), 4.58 (d, *J* = 12.0 Hz, 1H), 4.52 (d, *J* = 11.2 Hz, 1H), 4.39

(td, *J* = 17.9, 10.5 Hz, 2H), 4.33 – 4.16 (m, 4H), 4.12 (dd, *J* = 11.3, 4.9 Hz, 1H), 3.98 (d, *J* = 1.6 Hz, 1H), 3.29 (dd, *J* = 14.5, 5.1 Hz, 1H), 2.88 (dd, *J* = 14.5, 3.7 Hz, 1H), 2.04 (s, 3H), 2.00 (s, 3H).

^{13}C NMR (CDCl3, 150 MHz): δ 170.54, 170.20, 169.98, 155.75, 143.84, 143.71, 141.27, 141.24, 137.55, 137.41, 131.39, 128.52, 128.41, 128.18, 128.12, 127.93, 127.79, 127.65, 127.10, 127.05, 125.17, 125.08, 119.90, 119.11, 85.71, 75.29, 74.89, 73.35, 72.58, 72.49, 69.30, 67.06, 66.29, 62.88, 54.27, 47.06, 34.20, 20.95, 20.66.

HRMS (ESI): calc. for C_{38}H $C_{45}H_{47}NO_{11}SNa$ [M + Na]$^+$, 832.2762; found: 832.2761.

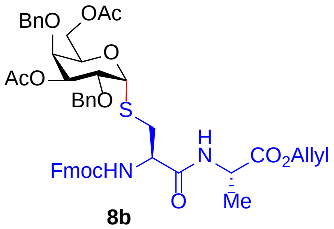

8b

86%, α:β > 4.5:1

^{1}H NMR (CDCl$_3$, 600 MHz): α/β mixture, inseparable, δ 7.79 – 7.70 (m, 2H), 7.64 – 7.54 (m, 2H), 7.44 – 7.25 (m, 14H), 7.17 (d, *J* = 20.4 Hz, 0.2H), 6.97 (d, *J* = 6.8 Hz, 1H), 6.09 (d, *J* = 5.7 Hz, 0.2H), 5.97 (d, *J* = 6.7 Hz, 1H), 5.93 – 5.82 (m, 1H), 5.45 (s, 1H), 5.31 (d, *J* = 17.3 Hz, 1H), 5.23 (d, *J* = 10.4 Hz, 1H), 5.16 – 5.05 (m, 1H), 4.97 (d, *J* = 9.0 Hz, 0.2H), 4.86 (d, *J* = 10.8 Hz, 0.2H), 4.74 – 4.48 (m, 7H), 4.44 (d, *J* = 5.8 Hz, 2H), 4.37 (d, *J* = 5.3 Hz, 1H), 4.21 (dd, *J* = 18.3, 11.6 Hz, 4H), 4.12 – 3.94 (m, 3H), 3.84 (d, *J* = 10.1 Hz, 1H), 3.19 (dd, *J* = 32.9, 11.6 Hz, 1.2H), 2.97 (d, *J* = 8.1 Hz, 0.2H), 2.80 (d, *J* = 10.3 Hz, 1H), 2.04 (s, 3H), 1.95 (s, 3H), 1.38 (d, *J* = 6.9 Hz, 3H).

^{13}C NMR (CDCl3, 150 MHz): δ 171.99, 170.51, 170.17, 169.61, 155.98, 146.78, 143.73, 143.66, 141.31, 141.26, 137.48, 137.43, 135.83, 131.66, 131.53, 128.51, 128.45, 128.40, 128.38, 128.14, 128.09, 128.04, 127.98, 127.96, 127.90, 127.71, 127.70, 127.14, 127.08, 125.02, 124.96, 121.19, 119.95, 119.92, 118.71, 118.57, 85.41, 75.56, 75.12, 75.04, 74.56, 74.41, 73.50, 72.76, 72.52, 69.30, 66.98, 65.93, 65.84, 63.01, 62.72, 55.23, 54.62, 48.54, 48.35, 47.09, 33.68, 29.67, 20.97, 20.87, 20.66, 18.08, 17.93.

HRMS (ESI): calc. for C_{38}H $C_{48}H_{52}N_2O_{12}SNa$ [M + Na]$^+$, 903.3133; found: 903.3136.

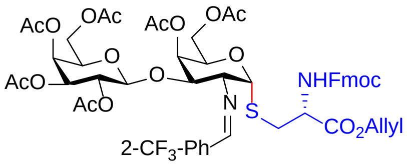

9a

63%, α:β > 20:1

^{1}H NMR ($CDCl_3$, 600 MHz): δ 8.61 (s, 1H), 8.24 (d, *J* = 7.7 Hz, 1H), 7.81 – 7.68 (m, 3H), 7.65 – 7.52 (m, 4H), 7.40 (dd, *J* = 13.1, 7.2 Hz, 2H), 7.31 (dd, *J* = 16.3, 7.9 Hz, 2H), 5.90 (d, *J* = 8.5 Hz, 1H), 5.87 – 5.77 (m, 1H), 5.51 – 5.48 (m, 1H), 5.23 (ddd, *J* = 21.7, 14.4, 6.8 Hz, 4H), 5.01 (dd, *J* = 10.4, 7.9 Hz, 1H), 4.83 (dd, *J* = 10.5, 3.5 Hz, 1H), 4.60 (ddd, *J* = 30.9, 23.7, 6.4 Hz, 4H), 4.42 – 4.19 (m, 5H), 4.17 – 4.01 (m, 5H), 3.84 (t, *J* = 6.6 Hz, 1H), 3.16 (dd, *J* = 14.1, 5.7 Hz, 1H), 2.95 (dd, *J* = 14.1, 4.0 Hz, 1H), 2.17 (s, 3H), 2.12 (s, 3H), 2.05 (d, *J* = 6.8 Hz, 6H), 1.89 (s, 3H), 1.48 (s, 3H).

^{13}C NMR (CDCl3, 150 MHz): δ 171.10, 170.53, 170.35, 170.25, 170.15, 170.04, 169.86, 168.76, 160.10, 155.66, 143.81, 143.64, 141.28, 133.56, 133.30, 132.28, 132.14, 131.23, 130.90, 129.04, 128.72, 127.70, 127.00, 125.73, 125.07, 119.99, 119.06, 100.65, 85.64, 75.21, 70.75, 70.70, 69.08, 68.84, 68.69, 68.47, 67.16, 67.11, 66.70, 66.31, 63.02, 60.98, 60.36, 54.06, 47.06, 32.95, 21.02, 20.76, 20.69, 20.67, 20.61, 20.46, 19.92.

HRMS (ESI): calc. for $C_{53}H_{57}F_3N_2O_{19}SNa$ $[M + Na]^+$, 1137.3121; found: 1137.3125.

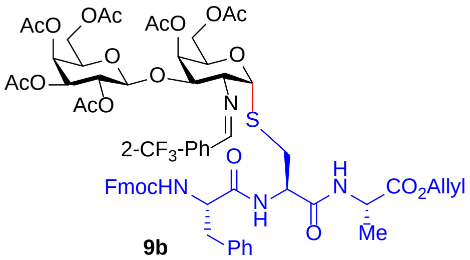

9b

69%, α:β > 20:1

^{1}H NMR ($CDCl_3$, 600 MHz): δ 8.61 (s, 1H), 8.23 (d, *J* = 7.7 Hz, 1H), 7.80 – 7.66 (m, 3H), 7.52 (ddt, *J* = 30.9, 15.4, 7.6 Hz, 4H), 7.39 (dd, *J* = 14.6, 7.2 Hz, 2H), 7.34 – 7.20 (m, 6H), 7.15 (s, 2H), 6.93 (d, *J* = 6.7 Hz, 1H), 6.77 (s, 1H), 5.87 (ddd, *J* = 16.2, 11.0, 5.7 Hz, 1H), 5.46 (d, *J* = 3.0 Hz, 1H), 5.37 – 5.22 (m, 5H), 4.99 (dd, *J* = 10.4, 7.9 Hz, 2H), 4.80 (dd, *J* = 10.5, 3.3 Hz, 1H), 4.64 – 4.41 (m, 6H), 4.34 – 4.26 (m, 2H), 4.19 – 4.02 (m, 5H), 3.76 (d, *J* = 6.9 Hz, 1H), 3.07 (s, 2H), 2.96 (d, *J* = 6.8 Hz, 1H), 2.82 (d, *J* = 10.5 Hz, 1H), 2.13 (s, 3H), 2.10 (s, 3H), 2.04 (s, 3H), 2.01 (s, 3H), 1.88 (s, 3H), 1.43 (s, 3H), 1.33 (d, *J* = 7.1 Hz, 3H).

^{13}C NMR (CDCl3, 150 MHz): δ 171.99, 170.85, 170.82, 170.34, 170.24, 170.01, 169.96, 169.07, 168.74, 160.09, 143.65, 141.27, 141.23, 136.02, 133.34, 132.07, 131.46, 130.84, 129.26, 128.79, 128.72, 127.79, 127.75, 127.14, 127.08, 124.94, 120.02, 120.00, 118.71, 100.65, 84.97, 75.54, 70.75, 70.60, 68.92, 68.78, 68.49, 68.45, 67.11, 66.70, 65.92, 62.95, 60.93, 60.35, 55.94, 53.78, 48.33, 47.06, 30.89, 29.66, 29.24, 20.82, 20.77, 20.67, 20.63, 20.60, 20.45, 19.86, 17.86.

HRMS (ESI): calc. for $C_{65}H_{71}F_3N_4O_{21}SNa$ $[M + Na]^+$, 1355.4176; found: 1355.4182.

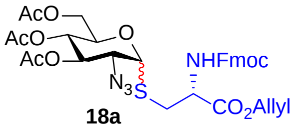

18a

75% (α:β = 1.6:1)

^{1}H NMR (CDCl$_3$, 600 MHz): α/β mixture, inseparable, δ 7.76 (d, *J* = 7.3 Hz, 4.4H), 7.60 (d, *J* = 5.5 Hz, 5H), 7.33 (dd, *J* = 33.0, 25.9 Hz, 9H), 6.04 (d, *J* = 8.4 Hz, 1.3H), 5.91 (d, *J* = 5.9 Hz, 2.6H), 5.69 – 5.50 (m, 3.6H), 5.48 – 5.09 (m, 9.2H), 5.00 (t, *J* = 9.6 Hz, 1.6H), 4.94 (t, *J* = 9.7 Hz, 1H), 4.70 (d, *J* = 41.2 Hz, 7H), 4.55 – 4.00 (m, 17H), 3.98 – 3.92 (m, 1H), 3.51 (d, *J* = 8.8 Hz, 1.6H), 3.35 – 3.21 (m, 2.6H), 3.04 (dd, *J* = 20.4, 9.2 Hz, 2.6H), 2.08 (s, 3H), 2.05 (s, 4.8H), 2.03 (s, 4.8H), 1.99 (s, 4.8H), 1.65 (s, 3H), 1.56 (s, 3H).

^{13}C NMR (CDCl3, 150 MHz): δ 170.48, 170.30, 170.01, 169.69, 155.68, 143.84, 143.74, 141.26, 131.27, 131.18, 127.73, 127.69, 127.05, 125.14, 125.08, 124.91, 119.97, 119.47, 119.12, 92.28, 85.39, 71.64, 71.25, 68.80, 68.56, 67.97, 67.31, 66.75, 66.54, 66.44, 62.04, 61.95, 61.53, 61.14, 60.35, 54.22, 53.40, 47.17, 47.04, 35.49, 30.43, 29.78, 29.66, 29.24, 28.79, 20.67, 20.64, 20.60, 20.58, 20.55.

HRMS (ESI): calc. for $C_{33}H_{36}N_4O_{11}SNa$ $[M + Na]^+$, 719.1993; found: 719.1998.

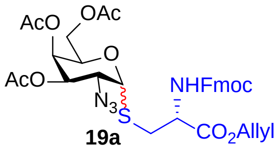

19a

82% (α:β = 2:1)

^{1}H NMR (CDCl$_3$, 600 MHz): α/β mixture, inseparable, δ 7.75 (t, *J* = 10.1 Hz, 5.2H), 7.61 (t, *J* = 8.8 Hz, 5.2H), 7.40 (dd, *J* = 12.1, 7.2 Hz, 5.5H), 7.36 – 7.29 (m, 5.4H), 6.07 (d, *J* = 8.5 Hz, 2H), 5.90 (ddd, *J* = 16.3, 10.5, 5.8 Hz, 2.8H), 5.66 (d, *J* = 8.3 Hz, 1H), 5.59 (d, *J* = 3.2 Hz, 1H), 5.46 –

5.23 (m, 11.2H), 5.03 (dd, *J* = 11.1, 2.8 Hz, 2H), 4.76 – 4.62 (m, 8H), 4.52 – 4.32 (m, 8H), 4.31 – 4.18 (m, 7H), 4.08 (dddd, *J* = 22.7, 18.2, 11.4, 6.4 Hz, 8H), 3.87 – 3.79 (m, 1H), 3.36 – 3.25 (m, 3H), 3.04 (td, *J* = 14.0, 5.3 Hz, 3H), 2.18 (s, 6H), 2.13 (s, 3H), 2.05 (d, *J* = 9.0 Hz, 6H), 1.99 (s, 6H), 1.64 (s, 3H), 1.56 (s, 3H).

^{13}C NMR (CDCl3, 150 MHz): δ 170.34, 170.29, 170.04, 169.82, 169.70, 169.46, 155.66, 143.83, 143.76, 143.63, 141.35, 141.30, 141.26, 131.31, 131.23, 127.70, 127.68, 127.03, 127.00, 125.12, 125.03, 125.00, 119.95, 119.36, 119.07, 92.75, 85.63, 69.67, 69.17, 67.83, 67.50, 67.35, 66.97, 66.88, 66.49, 66.42, 61.76, 61.73, 60.35, 57.98, 57.75, 54.15, 47.12, 47.06, 34.90, 29.85, 21.01, 20.61, 20.55, 20.49.

HRMS (ESI): calc. for $C_{33}H_{36}N_4O_{11}SNa$ $[M + Na]^+$, 719.1993; found: 719.1999.

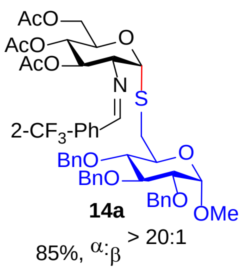

14a

85%, α:β > 20:1

^{1}H NMR (CDCl$_3$, 600 MHz): δ 8.60 (d, *J* = 1.9 Hz, 1H), 8.21 (d, *J* = 7.6 Hz, 1H), 7.66 (d, *J* = 7.5 Hz, 1H), 7.53 (dd, *J* = 14.6, 7.4 Hz, 2H), 7.36 – 7.22 (m, 15H), 5.57 (t, *J* = 9.7 Hz, 1H), 5.42 (d, *J* = 5.6 Hz, 1H), 5.13 (t, *J* = 9.8 Hz, 1H), 4.93 (dd, *J* = 18.2, 10.9 Hz, 2H), 4.78 (d, *J* = 10.8 Hz, 1H), 4.70 (d, *J* = 12.0 Hz, 1H), 4.63 (d, *J* = 11.0 Hz, 1H), 4.59 – 4.53 (m, 2H), 4.51 (d, *J* = 3.5 Hz, 1H), 4.33 (dd, *J* = 12.3, 4.3 Hz, 1H), 4.02 (dd, *J* = 12.3, 2.0 Hz, 1H), 3.97 – 3.88 (m, 2H), 3.85 – 3.79 (m, 1H), 3.46 – 3.40 (m, 2H), 3.31 (s, 3H), 2.99 (dd, *J* = 13.7, 2.3 Hz, 1H), 2.63 (dd, *J* = 13.1, 8.2 Hz, 1H), 2.06 (s, 3H), 2.03 (s, 3H), 1.86 (s, 3H).

^{13}C NMR (CDCl3, 150 MHz): δ 170.56, 169.89, 169.68, 169.63, 159.89, 138.61, 138.05, 138.01, 132.23, 130.92, 130.78, 129.09, 128.72, 128.59, 128.41, 128.40, 128.37, 127.99, 127.96, 127.88, 127.79, 127.74, 127.62, 97.76, 84.21, 81.88, 80.42, 80.17, 75.69, 75.14, 73.33, 73.22, 72.10, 71.75, 69.70, 68.70, 67.90, 62.10, 55.07, 30.53, 20.70, 20.66, 20.37.

HRMS (ESI): calc. for $C_{48}H_{52}F_3NO_{12}SNa$ $[M + Na]^+$, 946.3055; found: 946.3063.

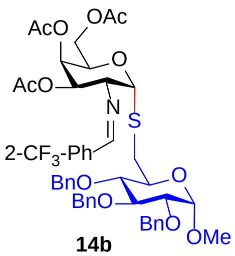

14b

87%, α:β > 20:1

^{1}H NMR (CDCl$_3$, 600 MHz): δ 8.62 (d, *J* = 2.0 Hz, 1H), 8.21 (d, *J* = 7.6 Hz, 1H), 7.67 (d, *J* = 7.6 Hz, 1H), 7.54 (dt, *J* = 24.8, 7.4 Hz, 2H), 7.39 – 7.21 (m, 15H), 5.48 (dd, *J* = 8.1, 4.0 Hz, 2H), 5.42 (dd, *J* = 10.5, 3.3 Hz, 1H), 4.93 (dd, *J* = 24.2, 10.9 Hz, 2H), 4.81 – 4.56 (m, 5H), 4.52 (d, *J* = 3.5 Hz, 1H), 4.18 – 4.05 (m, 3H), 3.94 (t, *J* = 9.2 Hz, 1H), 3.86 – 3.78 (m, 1H), 3.46 – 3.40 (m, 2H), 3.29 (s, 3H), 3.00 (dd, *J* = 13.8, 2.3 Hz, 1H), 2.64 (dd, *J* = 13.8, 7.6 Hz, 1H), 2.18 (s, 3H), 1.98 (s, 3H), 1.86 (s, 3H).

^{13}C NMR (CDCl3, 150 MHz): δ 170.30, 170.11, 169.68, 159.89, 138.61, 138.06, 138.02, 132.18, 130.65, 129.07, 128.42, 128.40, 128.37, 127.99, 127.97, 127.88, 127.81, 127.76, 127.62, 125.44, 97.72, 84.47, 81.91, 80.50, 80.18, 75.70, 75.18, 73.32, 69.74, 69.54, 67.19, 66.85, 61.97, 54.99, 30.23, 20.75, 20.66, 20.37.

HRMS (ESI): calc. for $C_{48}H_{52}F_3NO_{12}SNa$ $[M + Na]^+$, 946.3055; found: 946.3061.

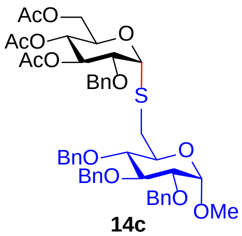

14c

88%, α:β > 20:1

^{1}H NMR (CDCl$_3$, 600 MHz): δ 7.42 – 7.13 (m, 20H), 5.58 (d, *J* = 5.6 Hz, 1H), 5.30 (dd, *J* = 15.4, 5.7 Hz, 1H), 5.03 – 4.88 (m, 3H), 4.80 (dd, *J* = 30.1, 11.5 Hz, 2H), 4.68 – 4.58 (m, 3H), 4.53 (d, *J* = 3.5 Hz, 1H), 4.44 (d, *J* = 12.2 Hz, 1H), 4.39 (ddd, *J* = 10.2, 4.0, 2.1 Hz, 1H), 4.29 (dd, *J* = 12.4, 4.2 Hz, 1H), 4.00 (t, *J* = 9.2 Hz, 1H), 3.95 (dd, *J* = 12.4, 2.0 Hz, 1H), 3.90 – 3.85 (m, 1H), 3.78 (dd, *J* = 9.9, 5.6 Hz, 1H), 3.54 (t, *J* = 9.3 Hz, 1H), 3.48 (dd, *J* = 9.6, 3.6 Hz, 1H), 3.38 (s, 3H), 2.94 (dd, *J* = 13.9, 2.5 Hz, 1H), 2.67 (dd, *J* = 13.9, 6.7 Hz, 1H), 2.03 (s, 3H), 2.01 (s, 3H), 1.99 (s, 3H).

^{13}C NMR (CDCl3, 150 MHz): δ ^{z}170.49, 169.90, 169.76, 138.64, 138.03, 137.97, 137.12, 128.45, 128.39, 128.08, 128.03, 127.96, 127.86, 127.83, 127.81, 127.77, 127.60, 98.05, 83.62, 81.88, 80.10,

79.94, 75.66, 75.55, 75.26, 73.41, 72.09, 71.62, 70.20, 68.40, 67.56, 61.93, 55.34, 30.39, 20.75, 20.68, 20.62.

HRMS (ESI): calc. for $C_{47}H_{54}O_{13}SNa$ $[M + Na]^+$, 881.3177; found: 881.3178.

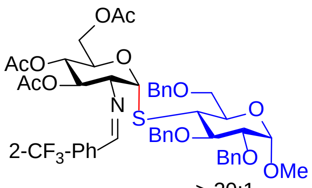

15a: 84% α:β > 20:1

^{1}H NMR (CDCl$_3$, 600 MHz): δ 8.32 (d, *J* = 1.8 Hz, 1H), 8.23 (d, *J* = 7.8 Hz, 1H), 7.66 (d, *J* = 7.7 Hz, 1H), 7.59 (t, *J* = 7.4 Hz, 1H), 7.54 (t, *J* = 7.6 Hz, 1H), 7.40 – 7.23 (m, 10H), 7.10 (t, *J* = 7.3 Hz, 1H), 7.04 (t, *J* = 7.5 Hz, 2H), 6.96 (d, *J* = 7.3 Hz, 2H), 5.82 (d, *J* = 5.8 Hz, 1H), 5.40 (t, *J* = 9.7 Hz, 1H), 5.06 (t, *J* = 9.8 Hz, 1H), 4.95 (d, *J* = 11.0 Hz, 1H), 4.74 (d, *J* = 12.1 Hz, 1H), 4.64 (ddd, *J* = 22.5, 16.1, 7.7 Hz, 5H), 4.40 – 4.33 (m, 1H), 4.21 (dd, *J* = 12.3, 3.9 Hz, 1H), 4.02 (t, *J* = 9.9 Hz, 1H), 3.90 – 3.76 (m, 4H), 3.71 (dd, *J* = 9.9, 5.7 Hz, 1H), 3.51 (dd, *J* = 9.5, 3.4 Hz, 1H), 3.41 (s, 3H), 3.02 (t, *J* = 10.5 Hz, 1H), 2.02 (d, *J* = 6.2 Hz, 6H), 1.82 (s, 3H).

^{13}C NMR (CDCl3, 150 MHz): δ 170.49, 169.71, 169.68, 159.57, 138.21, 137.99, 137.96, 133.19, 132.16, 130.77, 128.92, 128.42, 128.40, 128.04, 127.92, 127.88, 127.69, 127.66, 127.23, 126.94, 125.43, 98.24, 84.83, 83.43, 80.72, 75.87, 73.65, 73.12, 72.01, 71.53, 70.05, 69.84, 68.41, 68.35, 61.82, 55.36, 43.99, 20.71, 20.66, 20.27.

HRMS (ESI): calc. for $C_{48}H_{52}F_3NO_{12}SNa$ $[M + Na]^+$, 946.3055; found: 946.3052.

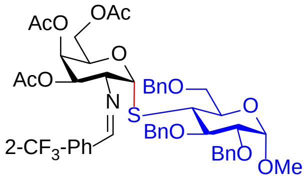

15b: 86% α:β > 20:1

^{1}H NMR (CDCl$_3$, 600 MHz): δ 8.36 (d, *J* = 1.9 Hz, 1H), 8.23 (d, *J* = 7.8 Hz, 1H), 7.67 (d, *J* = 7.7 Hz, 1H), 7.61 (t, *J* = 7.6 Hz, 1H), 7.55 (t, *J* = 7.6 Hz, 1H), 7.41 – 7.21 (m, 10H), 7.17 – 6.91 (m, 5H), 5.81 (d, *J* = 5.7 Hz, 1H), 5.36 (d, *J* = 2.4 Hz, 1H), 5.22 (dd, *J* = 10.6, 3.3 Hz, 1H), 4.95 (d, *J* = 11.1 Hz, 1H), 4.76 – 4.54 (m, 6H), 4.36 (t, *J* = 6.6 Hz, 1H), 4.06 – 3.73 (m, 7H), 3.51 (dd, *J* = 9.5, 3.4 Hz, 1H), 3.42 (s, 3H), 3.01 – 2.89 (m, 1H), 2.14 (s, 3H), 1.96 (s, 3H), 1.83 (s, 3H).

^{13}C NMR (CDCl3, 150 MHz): δ 170.15, 170.12, 169.66, 159.60, 138.34, 138.10, 138.00, 133.46, 132.17, 130.67, 128.98, 128.41, 128.38, 128.03, 127.98, 127.88, 127.72, 127.64, 127.19, 127.00, 125.41, 98.15, 85.13, 83.56, 80.77, 75.85, 73.33, 73.07, 70.34, 69.79, 69.12, 67.14, 66.52, 61.92, 55.32, 43.94, 29.67, 20.74, 20.69, 20.29.

HRMS (ESI): calc. for $C_{48}H_{52}F_3NO_{12}SNa$ $[M + Na]^+$, 946.3055; found: 946.3058.

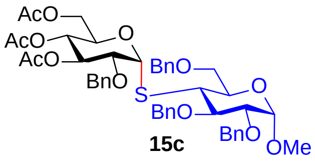

15c

89%, α:β > 20:1

^{1}H NMR (CDCl$_3$, 600 MHz): δ 7.40 – 7.13 (m, 18H), 6.94 (dd, *J* = 6.4, 2.7 Hz, 2H), 6.02 (d, *J* = 5.6 Hz, 1H), 5.20 (dd, *J* = 20.3, 10.8 Hz, 2H), 4.89 (dd, *J* = 10.7, 8.6 Hz, 2H), 4.75 – 4.66 (m, 2H), 4.64 – 4.55 (m, 3H), 4.40 (d, *J* = 12.3 Hz, 1H), 4.18 (ddd, *J* = 16.4, 11.2, 2.9 Hz, 2zH), 4.02 (t, *J* = 9.9 Hz, 1H), 3.97 (d, *J* = 12.3 Hz, 1H), 3.81 (ddd, *J* = 21.8, 10.2, 5.8 Hz, 4H), 3.63 (dd, *J* = 9.8, 5.6 Hz, 1H), 3.57 (dd, *J* = 9.4, 3.4 Hz, 1H), 3.40 (s, 3H), 3.03 (t, *J* = 10.8 Hz, 1H), 2.00 (d, *J* = 5.7 Hz, 6H), 1.91 (s, 3H).

^{13}C NMR (CDCl3, 150 MHz): δ 170.44, 169.92, 169.59, 139.00, 137.96, 137.87, 137.07, 128.42, 128.39, 128.33, 128.17, 128.07, 127.91, 127.69, 127.67, 127.46, 127.11, 126.26, 98.10, 83.98, 82.41, 81.00, 75.51, 75.25, 73.65, 73.07, 71.89, 71.37, 70.04, 69.93, 68.19, 68.14, 61.75, 55.40, 44.21, 20.72, 20.71, 20.63.

HRMS (ESI): calc. for $C_{47}H_{54}O_{13}SNa$ $[M + Na]^+$, 881.3177; found: 881.3173.

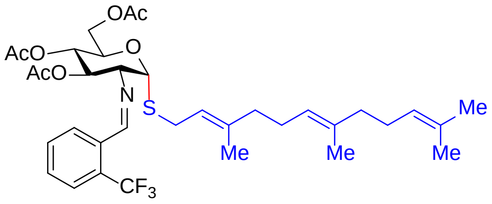

16a: 81% α:β > 20:1

^{1}H NMR (CDCl$_3$, 600 MHz): δ 8.59 (d, *J* = 2.0 Hz, 1H), 8.21 (d, *J* = 7.8 Hz, 1H), 7.66 (d, *J* = 7.7 Hz, 1H), 7.59 (t, *J* = 7.6 Hz, 1H), 7.52 (t, *J* = 7.6 Hz, 1H), 5.58 (t, *J* = 9.6 Hz, 1H), 5.32 (d, *J* = 5.7 Hz, 1H), 5.23 (t, *J* = 7.7 Hz, 1H), 5.08 (ddd, *J* = 13.2, 8.3, 7.6 Hz, 3H), 4.63 (ddd, *J* = 10.2, 4.9, 2.0 Hz, 1H), 4.36 (dd, *J* = 12.2, 5.0 Hz, 1H), 4.14 (dd, *J* = 12.2, 2.0 Hz, 1H), 3.93 (dd, *J* = 9.9, 5.6

Hz, 1H), 3.29 (dd, J = 13.2, 8.9 Hz, 1H), 3.08 (dd, J = 13.2, 6.6 Hz, 1H), 2.13 – 1.99 (m, 12H), 1.97 – 1.91 (m, 2H), 1.86 (s, 3H), 1.67 (d, J = 6.0 Hz, 6H), 1.57 (d, J = 10.6 Hz, 6H).

^{13}C NMR (CDCl3, 150 MHz): δ 170.62, 169.95, 169.62, 159.82, 139.93, 135.30, 133.26, 132.22, 131.27, 130.69, 129.15, 129.00, 125.39, 125.35, 124.24, 123.72, 119.12, 82.87, 72.15, 71.95, 69.00, 67.79, 62.47, 39.66, 39.60, 29.67, 26.78, 26.67, 26.38, 25.64, 20.74, 20.69, 20.37, 17.65, 15.95.

HRMS (ESI): calc. for $C_{35}H_{46}F_3NO_7SNa$ [M + Na]$^+$, 704.2839; found: 704.2833.

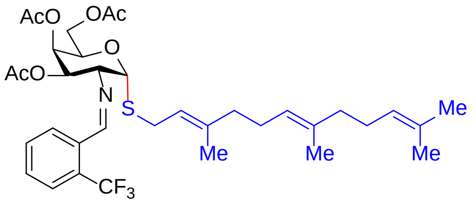

16b: 82% α:β > 20:1

^{1}H NMR (CDCl$_3$, 600 MHz): δ 8.62 (d, J = 2.0 Hz, 1H), 8.20 (d, J = 7.8 Hz, 1H), 7.67 (d, J = 7.7 Hz, 1H), 7.59 (t, J = 7.6 Hz, 1H), 7.52 (t, J = 7.6 Hz, 1H), 5.50 (d, J = 2.4 Hz, 1H), 5.42 (dd, J = 10.5, 3.3 Hz, 1H), 5.36 (d, J = 5.7 Hz, 1H), 5.22 (t, J = 7.7 Hz, 1H), 5.06 (dt, J = 7.1, 3.8 Hz, 2H), 4.77 (t, J = 6.4 Hz, 1H), 4.18 (d, J = 6.4 Hz, 2H), 4.09 (dd, J = 10.4, 5.7 Hz, 1H), 3.31 (dd, J = 13.2, 9.1 Hz, 1H), 3.06 (dd, J = 13.2, 6.5 Hz, 1H), 2.19 (s, 3H), 2.12 – 1.99 (m, 9H), 1.98 – 1.91 (m, 2H), 1.86 (s, 3H), 1.67 (d, J = 9.1 Hz, 6H), 1.58 (t, J = 7.6 Hz, 6H).

^{13}C NMR (CDCl3, 150 MHz): δ 170.43, 170.13, 169.66, 159.83, 139.81, 135.26, 133.52, 132.18, 131.25, 130.55, 129.13, 125.39, 125.35, 124.25, 123.76, 119.24, 83.07, 69.76, 67.22, 67.05, 66.80, 62.43, 39.65, 39.61, 29.67, 26.67, 26.54, 26.42, 25.64, 20.75, 20.72, 20.36, 17.64, 15.94, 15.91.

HRMS (ESI): calc. for $C_{35}H_{46}F_3NO_7SNa$ [M + Na]$^+$, 704.2839; found: 704.2836.

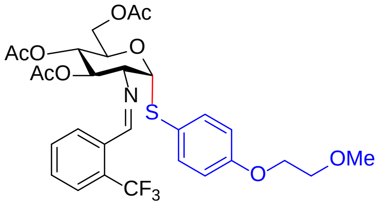

17a: 85% α:β > 20:1

^{1}H NMR (CDCl$_3$, 600 MHz): δ 8.65 (d, J = 1.7 Hz, 1H), 8.28 (d, J = 7.8 Hz, 1H), 7.68 (d, J = 7.8 Hz, 1H), 7.62 (t, J = 7.6 Hz, 1H), 7.55 (d, J = 7.6 Hz, 1H), 7.40 (d, J = 8.8 Hz, 2H), 6.86 (d, J = 8.8 Hz, 2H), 5.64 (t, J = 9.7 Hz, 1H), 5.51 (d, J = 5.6 Hz, 1H), 5.13 (t, J = 9.8 Hz, 1H), 4.74 (ddd,

J = 10.2, 4.8, 1.8 Hz, 1H), 4.32 (dd, *J* = 12.3, 5.0 Hz, 1H), 4.14 – 4.06 (m, 2H), 4.02 – 3.93 (m, 2H), 3.79 – 3.68 (m, 2H), 3.43 (s, 3H), 2.05 (s, 3H), 2.03 (s, 3H), 1.88 (s, 3H).

^{13}C NMR (CDCl3, 150 MHz): δ 170.57, 169.90, 169.65, 160.17, 158.83, 134.76, 133.23, 132.27, 130.81, 129.19, 129.05, 128.99, 128.18, 125.45, 125.41, 124.96, 123.46, 115.28, 87.38, 72.22, 71.71, 70.87, 68.87, 68.16, 67.32, 62.20, 59.18, 20.68, 20.36.

HRMS (ESI): calc. for $C_{29}H_{32}F_3NO_9SNa$ $[M + Na]^+$, 650.1642; found: 650.1637.

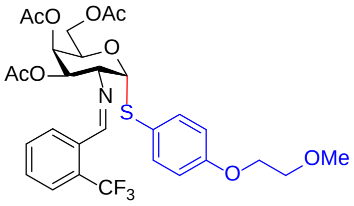

17b: 83% α:β > 20:1

^{1}H NMR (CDCl$_3$, 600 MHz): δ ^{1}H NMR (600 MHz, cdcl$_3$) δ 8.67 (d, *J* = 1.9 Hz, 1H), 8.27 (d, *J* = 7.8 Hz, 1H), 7.69 (d, *J* = 7.8 Hz, 1H), 7.62 (t, *J* = 7.6 Hz, 1H), 7.55 (t, *J* = 7.7 Hz, 1H), 7.42 (d, *J* = 8.8 Hz, 2H), 6.86 (d, *J* = 8.8 Hz, 2H), 5.51 (ddd, *J* = 13.8, 10.7, 4.5 Hz, 3H), 4.87 (t, *J* = 6.5 Hz, 1H), 4.18 – 4.00 (m, 5H), 3.79 – 3.68 (m, 2H), 3.43 (s, 3H), 2.17 (s, 3H), 1.99 (s, 3H), 1.88 (s, 3H).

^{13}C NMR (CDCl3, 150 MHz): δ 170.39, 170.10, 169.69, 160.17, 158.79, 135.00, 133.49, 132.24, 130.67, 129.17, 125.45, 125.41, 123.58, 115.18, 87.91, 70.88, 69.53, 67.37, 67.30, 67.16, 66.93, 62.02, 59.18, 20.72, 20.68, 20.36.

HRMS (ESI): calc. for $C_{29}H_{32}F_3NO_9SNa$ $[M + Na]^+$, 650.1642; found: 650.1635.

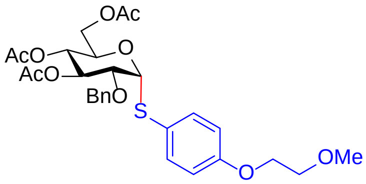

17c

92%, α:β > 20:1

^{1}H NMR (CDCl$_3$, 600 MHz): δ 7.42 – 7.26 (m, 7H), 6.86 (d, *J* = 8.7 Hz, 2H), 5.45 (d, *J* = 5.5 Hz, 1H), 5.35 (t, *J* = 9.6 Hz, 1H), 4.97 (t, *J* = 9.8 Hz, 1H), 4.71 (d, *J* = 12.2 Hz, 1H), 4.63 – 4.55 (m, 2H), 4.27 (dd, *J* = 12.3, 5.2 Hz, 1H), 4.14 – 4.07 (m, 2H), 3.98 (dd, *J* = 12.3, 2.0 Hz, 1H), 3.85 (dd, *J* = 9.9, 5.5 Hz, 1H), 3.76 – 3.70 (m, 2H), 3.44 (s, 3H), 2.04 – 2.01 (m, 9H).

^{13}C NMR (CDCl3, 150 MHz): δ 170.50, 169.92, 169.79, 158.95, 137.20, 134.67, 128.47, 128.07, 127.88, 123.60, 115.33, 87.42, 76.29, 72.39, 72.14, 70.88, 68.69, 67.91, 67.36, 62.14, 59.20, 20.75, 20.67, 20.64.

HRMS (ESI): calc. for $C_{28}H_{34}O_{10}SNa$ $[M + Na]^+$, 585.1765; found: 585.1768.

7. Synthesis of Sublancin Glycopeptide Fragment 22

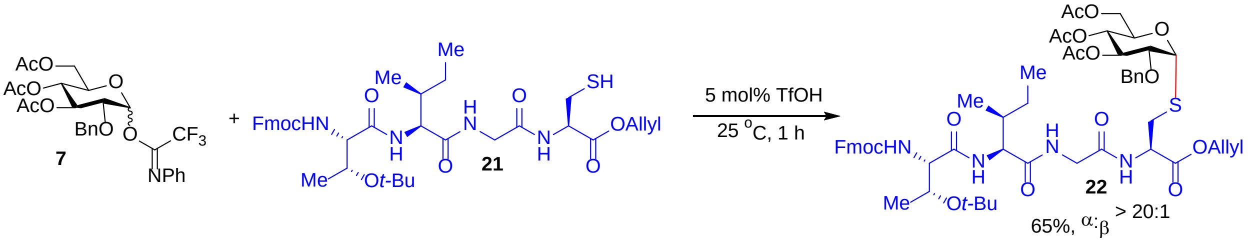

^{1}H NMR (CDCl$_3$, 600 MHz): δ 7.81 (d, *J* = 6.6 Hz, 1H), 7.76 (d, *J* = 7.5 Hz, 2H), 7.60 (d, *J* = 7.3 Hz, 2H), 7.52 (d, *J* = 8.7 Hz, 1H), 7.39 (t, *J* = 7.4 Hz, 2H), 7.36 – 7.26 (m, 5H), 6.46 (dd, *J* = 7.4, 4.8 Hz, 1H), 6.35 (d, *J* = 6.5 Hz, 1H), 5.81 (ddd, *J* = 25.2, 15.0, 5.5 Hz, 2H), 5.37 – 5.09 (m, 4H), 5.03 – 4.91 (m, 2H), 4.69 (d, *J* = 12.3 Hz, 1H), 4.61 (dd, *J* = 12.5, 6.6 Hz, 1H), 4.47 (d, *J* = 12.3 Hz, 1H), 4.40 – 4.16 (m, 7H), 4.13 – 4.00 (m, 2H), 3.91 (dd, *J* = 13.0, 6.3 Hz, 1H), 3.83 (dd, *J* = 9.8, 5.6 Hz, 1H), 3.63 (dd, *J* = 17.0, 4.4 Hz, 1H), 3.14 (dd, *J* = 14.0, 4.3 Hz, 1H), 2.93 (dd, *J* = 14.0, 8.8 Hz, 1H), 2.02 (s, 3H), 2.01 (s, 3H), 1.98 (s, 3H), 1.31 (s, 9H), 1.14 (d, *J* = 6.4 Hz, 3H), 0.99 – 0.92 (m, 5H), 0.90 – 0.84 (m, 4H).

^{13}C NMR (CDCl3, 150 MHz): δ 171.46, 170.81, 170.62, 170.49, 170.00, 169.76, 169.49, 168.97, 156.06, 143.84, 143.64, 141.24, 137.40, 131.08, 128.39, 128.37, 127.96, 127.88, 127.72, 127.56, 126.99, 125.16, 125.12, 120.01, 119.99, 119.27, 83.12, 81.74, 81.45, 75.55, 71.96, 71.36, 68.37, 67.91, 67.39, 67.01, 66.44, 61.89, 59.92, 58.13, 55.97, 53.72, 50.85, 47.09, 42.95, 36.59, 35.97, 31.91, 30.77, 29.69, 29.65, 29.35, 29.24, 28.12, 26.67, 25.25, 22.68, 20.77, 20.68, 20.65, 16.40, 15.58, 14.12, 11.12.

HRMS (ESI): calc. for $C_{56}H_{72}N_4O_{16}SNa$ $[M + Na]^+$, 1111.4556; found: 1111.4562.

8. Synthesis of S-linked TN and TF Glycopeptide Fragments 24 and 25

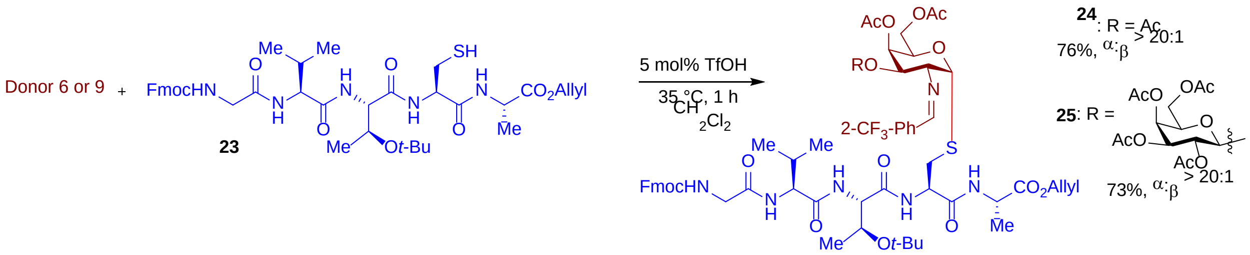

24:

^{1}H NMR (CDCl$_3$, 600 MHz): δ 8.60 (d, *J* = 1.7 Hz, 1H), 8.15 (d, *J* = 7.6 Hz, 1H), 7.75 (d, *J* = 7.5 Hz, 3H), 7.66 (d, *J* = 7.3 Hz, 1H), 7.62 – 7.49 (m, 3H), 7.39 (t, *J* = 7.4 Hz, 2H), 7.29 (t, *J* = 7.4 Hz, 2H), 6.69 (s, 1H), 5.84 (ddd, *J* = 22.8, 10.8, 5.7 Hz, 1H), 5.74 (s, 1H), 5.52 – 5.17 (m, 5H), 4.81 (s, 1H), 4.65 – 4.46 (m, 5H), 4.40 (dd, *J* = 19.9, 8.3 Hz, 3H), 4.26 – 3.87 (m, 8H), 3.11 (dd, *J* = 13.6, 6.6 Hz, 1H), 2.91 (dd, *J* = 13.6, 5.3 Hz, 1H), 2.17 (s, 3H), 2.10 – 2.00 (m, 4H), 1.84 (s, 3H), 1.31 (d, *J* = 7.2 Hz, 3H), 1.25 (s, 9H), 1.01 (d, *J* = 5.6 Hz, 3H), 0.92 (dd, *J* = 14.0, 6.8 Hz, 6H).

^{13}C NMR (CDCl3, 150 MHz): δ 171.97, 170.61, 170.10, 169.62, 169.45, 169.29, 160.13, 156.68, 143.73, 141.24, 133.57, 133.31, 132.06, 131.53, 130.72, 128.99, 127.71, 127.05, 125.44, 125.02, 123.12, 119.98, 118.60, 83.95, 75.64, 69.27, 67.27, 67.21, 67.18, 66.69, 66.22, 65.84, 61.99, 57.57, 55.95, 53.77, 52.97, 48.26, 47.04, 44.50, 29.67, 29.25, 28.07, 20.74, 20.68, 20.34, 19.14, 18.03, 18.00.

HRMS (ESI): calc. for $C_{59}H_{73}F_3N_6O_{16}SNa$ $[M + Na]^+$, 1233.4648; found: 1233.4656.

25:

^{1}H NMR (CDCl$_3$, 600 MHz): δ 8.58 (s, 1H), 8.23 (d, *J* = 7.7 Hz, 1H), 7.76 (d, *J* = 7.5 Hz, 2H), 7.69 (d, *J* = 7.8 Hz, 1H), 7.62 – 7.51 (m, 3H), 7.39 (t, *J* = 7.4 Hz, 2H), 7.30 (t, *J* = 7.3 Hz, 2H), 7.23 (s, 1H), 6.67 (s, 1H), 5.83 (ddd, *J* = 16.2, 10.9, 5.7 Hz, 1H), 5.76 (s, 1H), 5.47 (s, 1H), 5.35 – 5.18 (m, 4H), 5.11 (s, 1H), 5.00 (dd, *J* = 10.4, 7.9 Hz, 1H), 4.81 (dd, *J* = 10.5, 3.4 Hz, 1H), 4.73 (d, *J* = 4.9 Hz, 1H), 4.60 – 4.46 (m, 5H), 4.45 – 4.34 (m, 3H), 4.34 – 4.20 (m, 3H), 4.17 – 4.01 (m, 5H), 3.93 (d, *J* = 4.7 Hz, 2H), 3.82 (t, *J* = 6.8 Hz, 1H), 3.02 (dd, *J* = 13.6, 6.7 Hz, 1H), 2.87 (dd, *J* = 13.5, 5.2 Hz, 1H), 2.18 – 2.15 (m, 1H), 2.14 (s, 3H), 2.11 (s, 3H), 2.04 (d, *J* = 3.0 Hz, 6H), 1.88 (s, 3H), 1.45 (s, 3H), 1.27 (d, *J* = 7.2 Hz, 3H), 1.25 (s, 9H), 0.98 (s, 3H), 0.91 (d, *J* = 6.7 Hz, 3H), 0.89 (d, *J* = 6.7 Hz, 3H).

^{13}C NMR (CDCl3, 150 MHz): δ 171.99, 170.72, 170.65, 170.37, 170.25, 170.04, 169.96, 169.36, 169.21, 168.73, 159.88, 143.73, 141.24, 133.33, 132.03, 131.48, 130.83, 128.71, 127.73, 127.07, 125.03, 120.00, 118.61, 100.65, 84.10, 75.59, 75.57, 70.77, 70.58, 69.14, 68.81, 68.39, 68.31, 67.28, 66.64, 66.22, 65.82, 62.87, 60.87, 58.27, 57.41, 55.94, 53.76, 52.95, 48.23, 47.04, 44.52, 29.66, 29.24, 27.96, 20.77, 20.67, 20.61, 20.46, 19.90, 19.15, 17.99, 17.91.

HRMS (ESI): calc. for $C_{71}H_{89}F_3N_6O_{24}SNa$ [M + Na]$^+$, 1521.5493; found: 1521.5498.

9. References

[1] E. T. Sletten, Y.-J. Tu, H. B. Schlegel, H. M. Nguyen, *ACS Catal.* **2019,** *9*, 2110.

10. Spectral Data

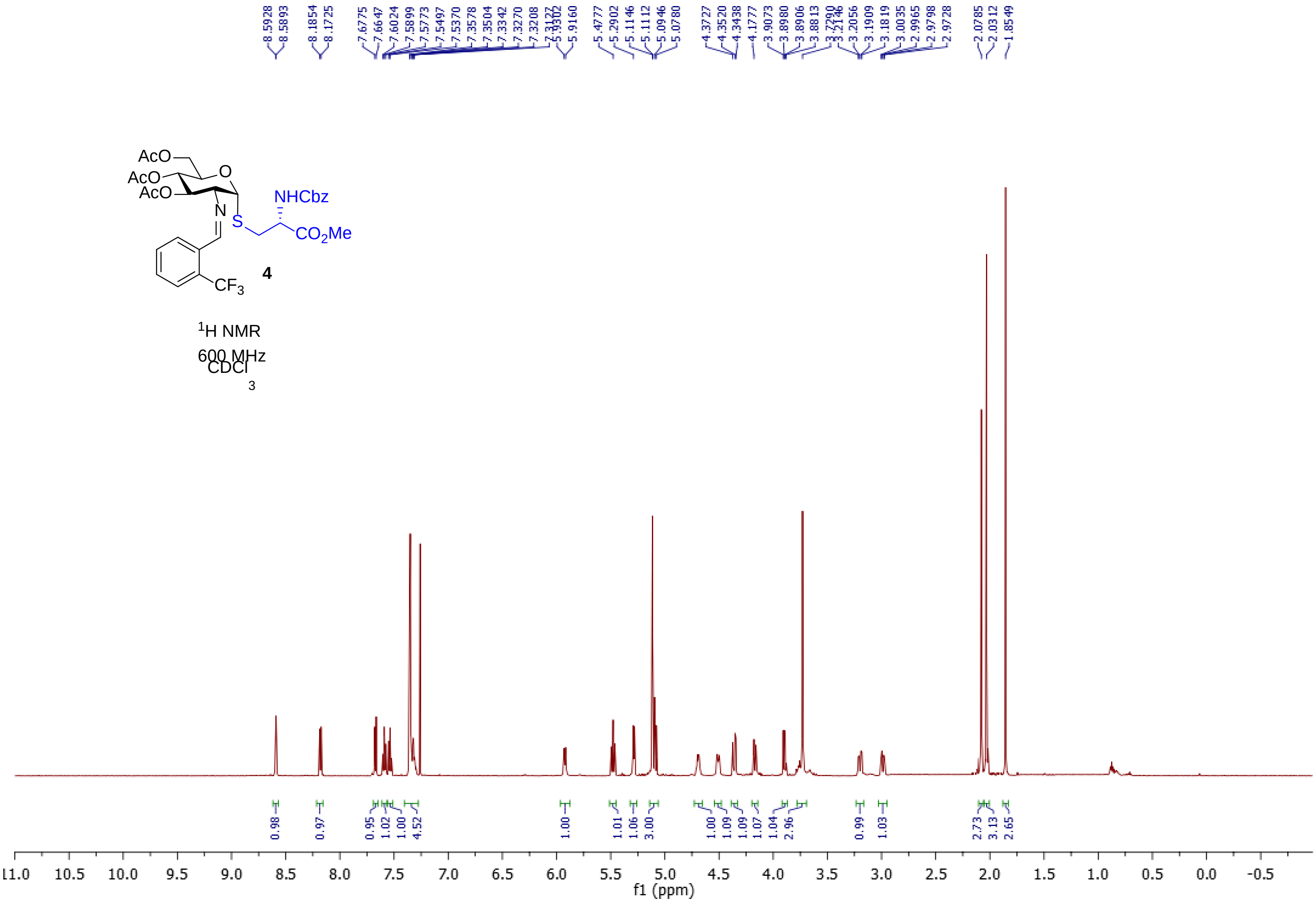

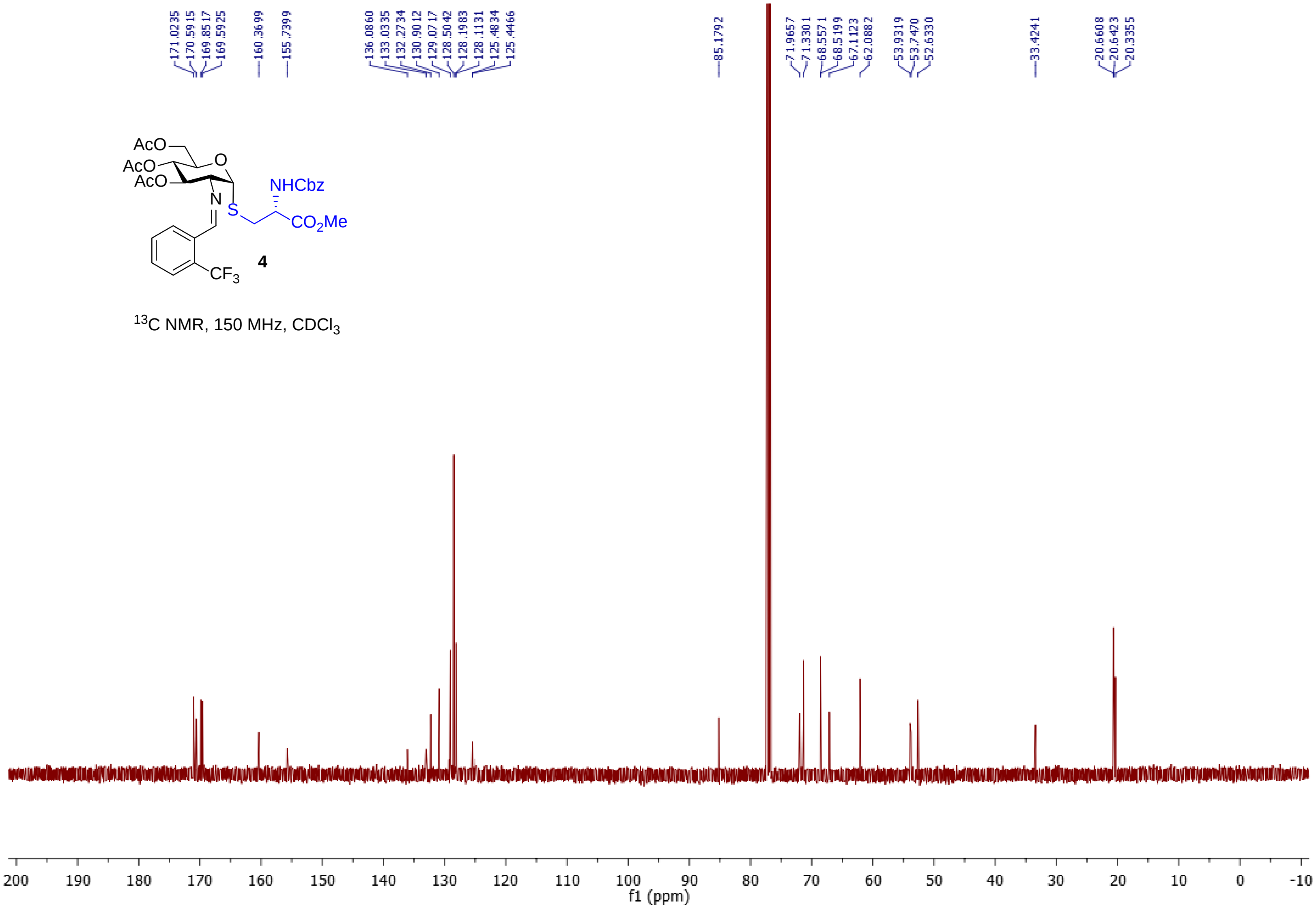

171.0235
170.5915
169.8517
169.5925
160.3699
155.7399
136.0860
133.0335
132.2734
130.9012
129.0717
128.5042
128.1983
128.1131
125.4834
125.4466
85.1792
71.9657
71.3301
68.5571
68.5199
67.1123
62.0882
53.9319
53.7470
52.6330
33.4241
20.6608
20.6423
20.3355
AcO
AcO
AcO
O
N
S
NHCbz
CO_2Me
CF_3
4
^{13}C NMR, 150 MHz, $CDCl_3$
200 190 180 170 160 150 140 130 120 110 100 90 80 70 60 50 40 30 20 10 0 -10
f1 (ppm)

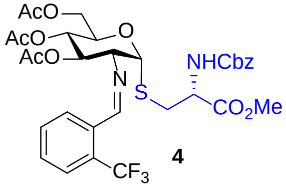

4

coupled ^{13}C NMR, 150 MHz, $CDCl_3$

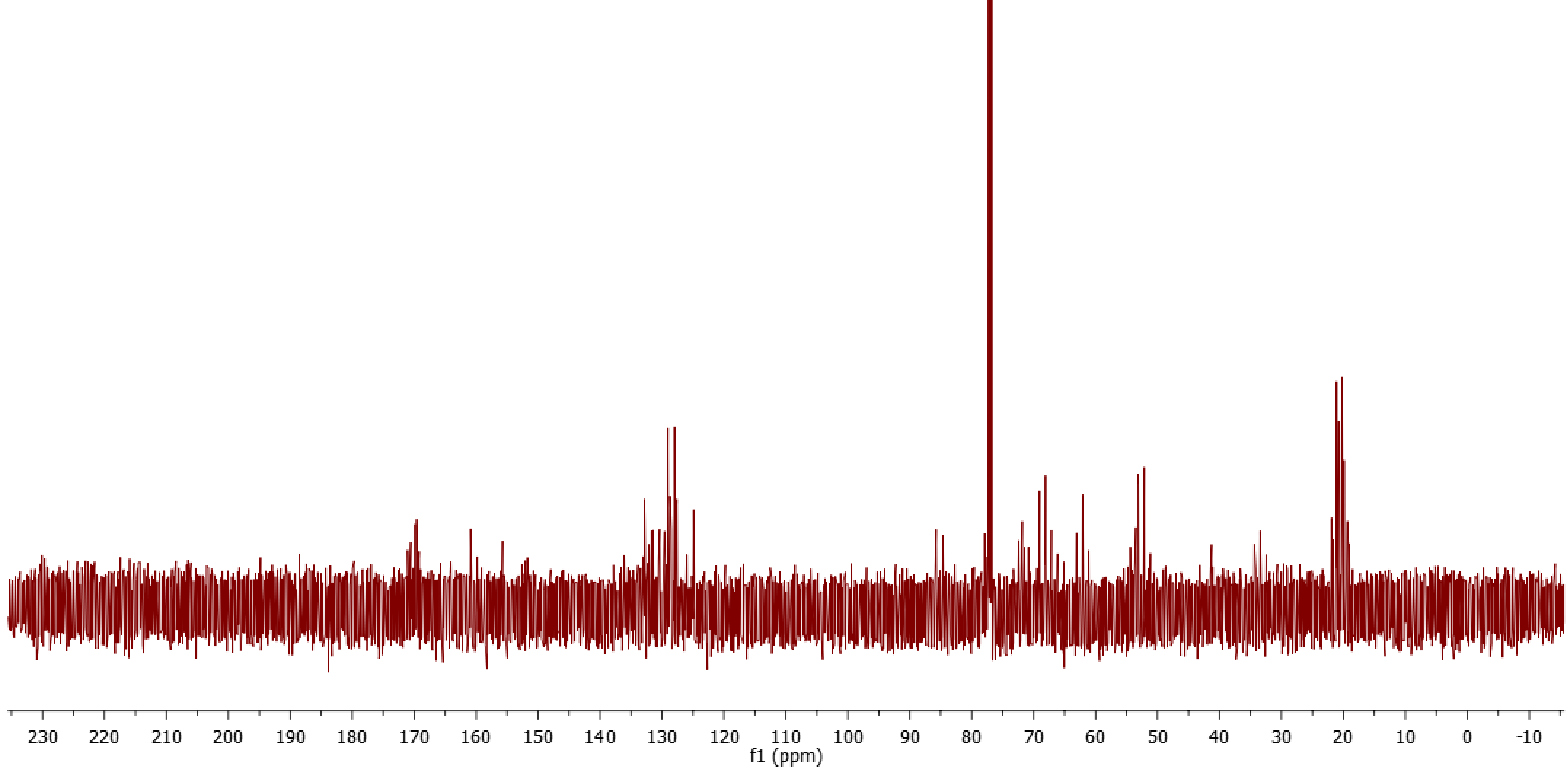

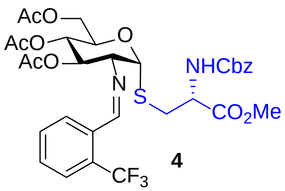

4

^{1}H-^{13}C HSQC, 600/150MHz, $CDCl_3$

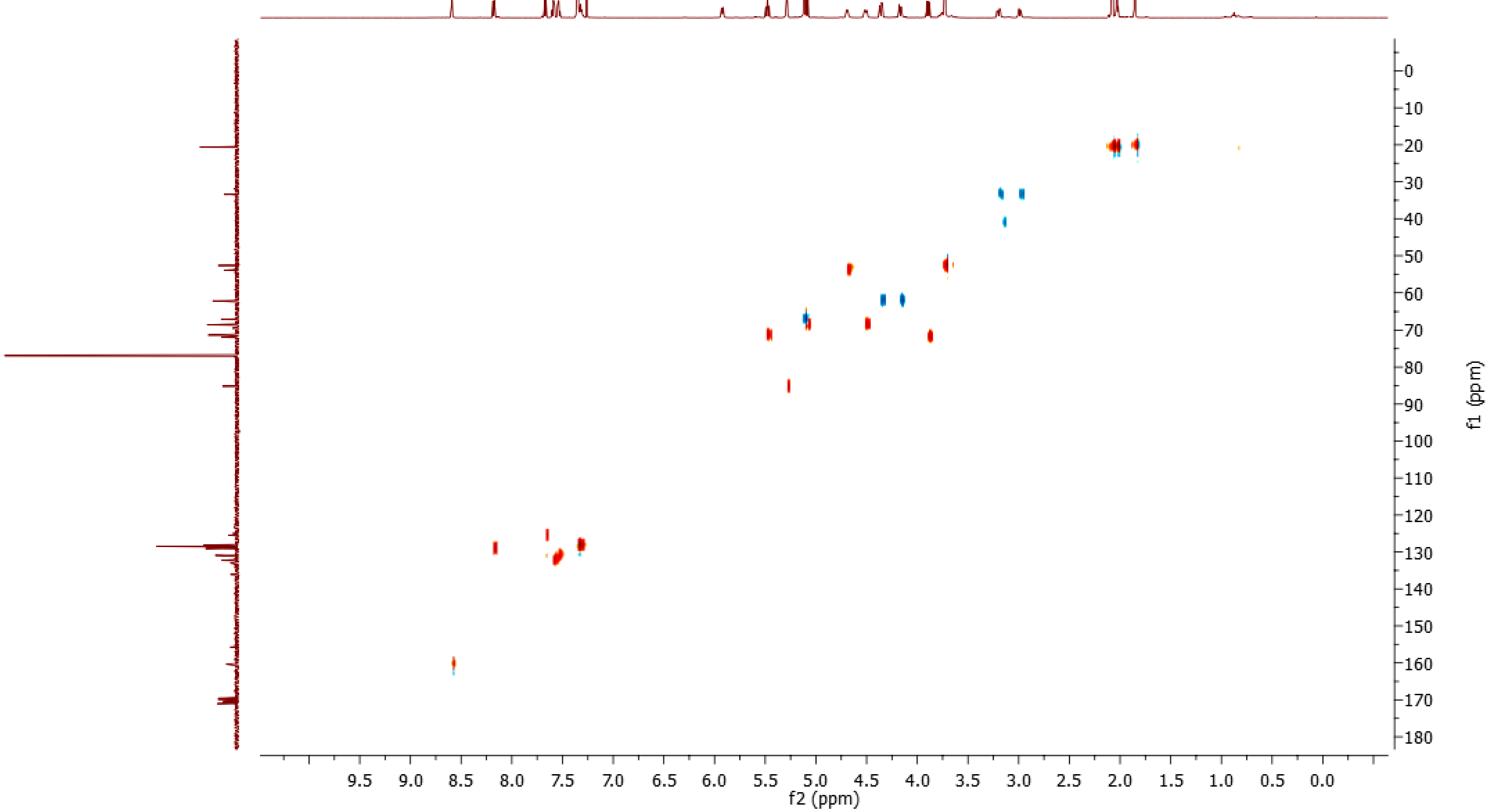

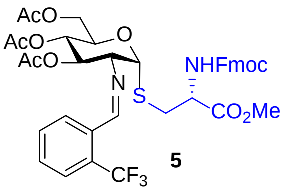

5

^{1}H NMR
600 MHz
$CDCl_3$

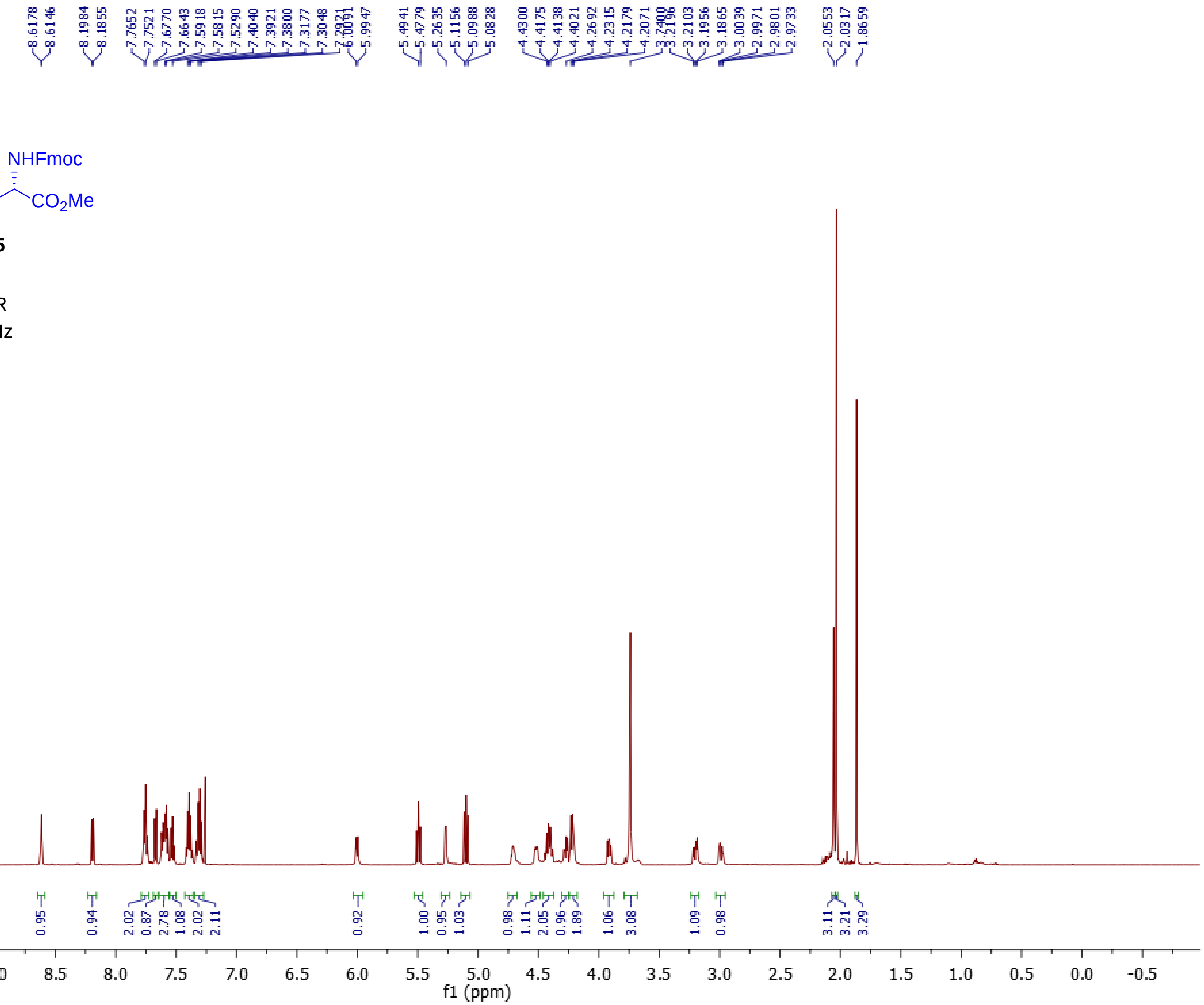

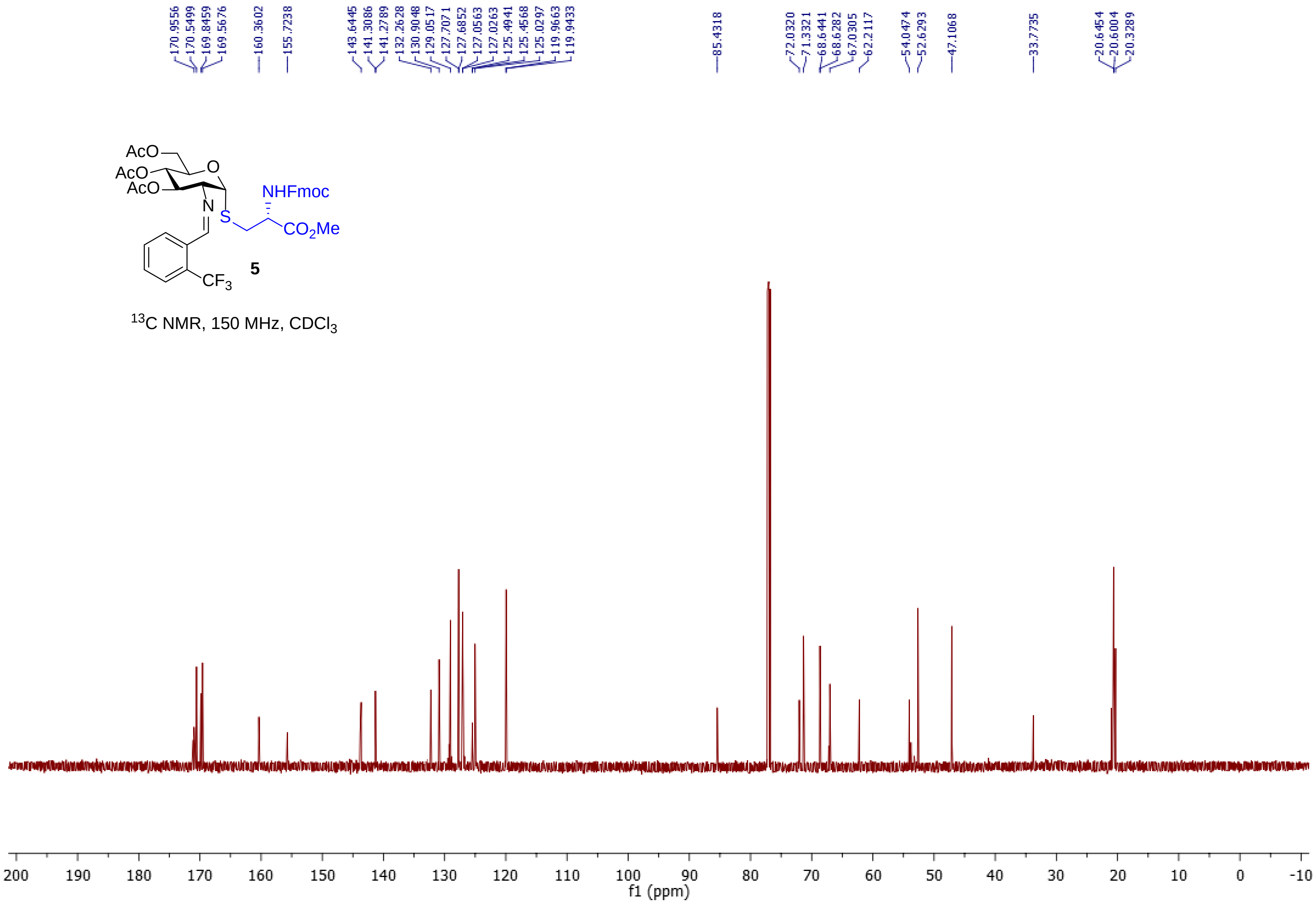

170.9556
170.5499
169.8459
169.5676
160.3602
155.7238
143.6445
141.3086
141.2789
132.2628
130.9048
129.0517
127.7071
127.6852
127.0563
127.0263
125.4941
125.4568
125.0297
119.9663
119.9433
85.4318
72.0320
71.3321
68.6441
68.6282
67.0305
62.2117
54.0474
52.6293
47.1068
33.7735
20.6454
20.6004
20.3289
AcO
AcO
AcO
O
N
S
NHFmoc
CO_2Me
CF_3
5
^{13}C NMR, 150 MHz, $CDCl_3$
200 190 180 170 160 150 140 130 120 110 100 90 80 70 60 50 40 30 20 10 0 -10
f1 (ppm)

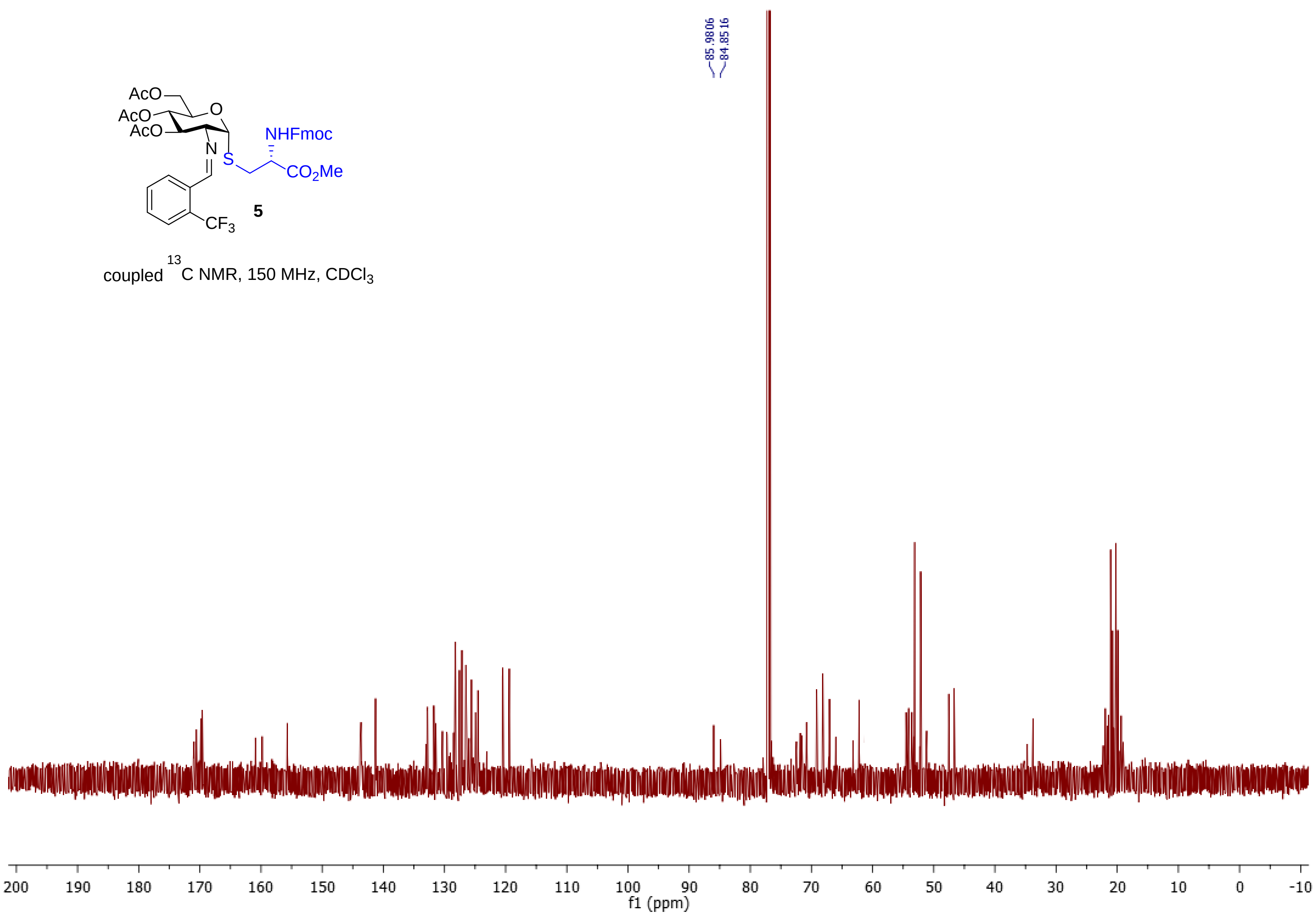
AcO
AcO
AcO
O
N
S
NHFmoc
CO_2Me
CF_3
5
coupled ^{13}C NMR, 150 MHz, $CDCl_3$
85.9806
84.8516
200 190 180 170 160 150 140 130 120 110 100 90 80 70 60 50 40 30 20 10 0 -10
f1 (ppm)

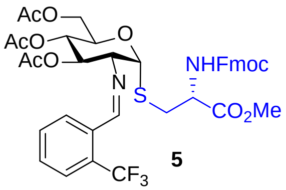

5

^1H-^1H COSY, 600 MHz, $CDCl_3$

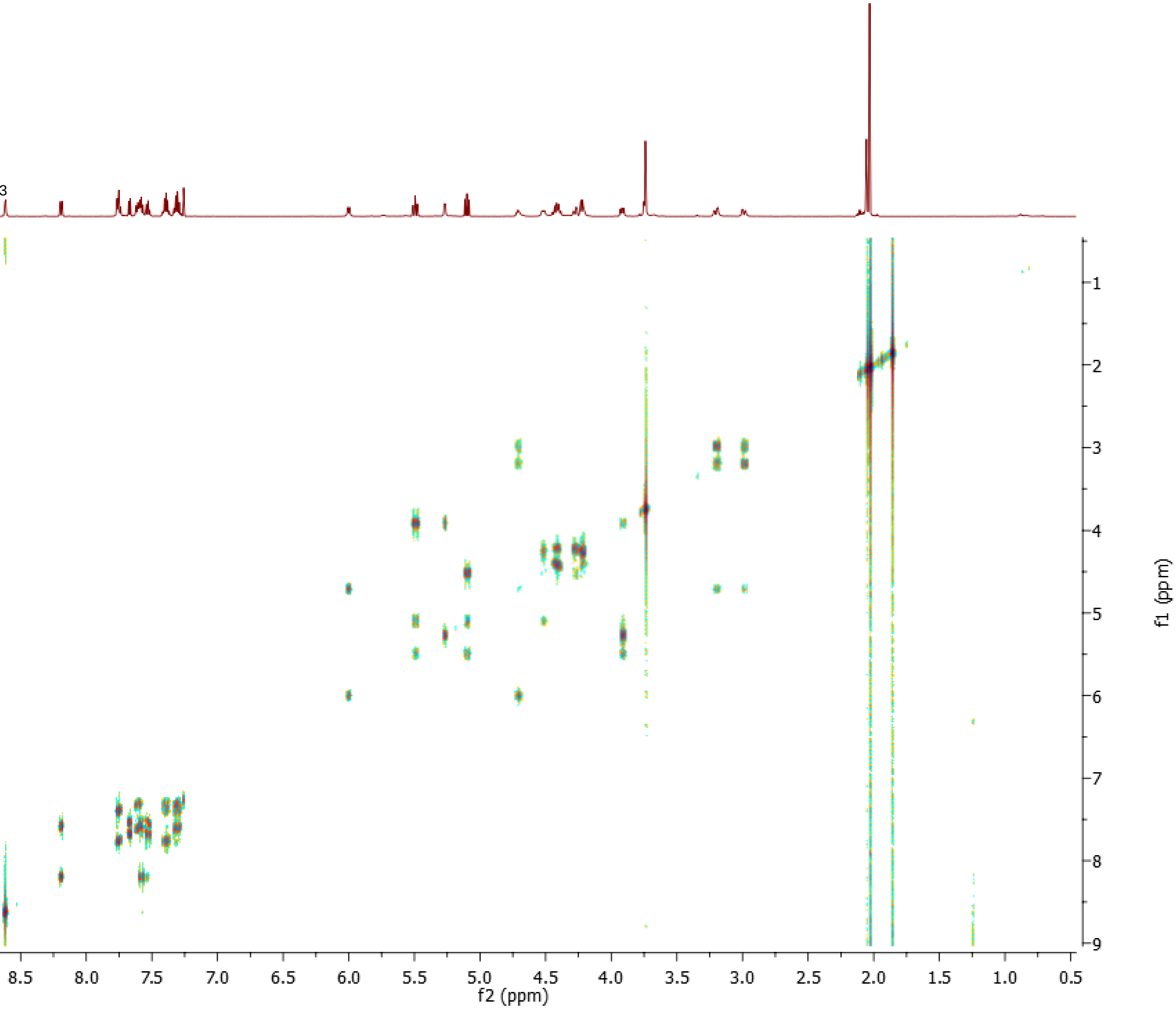

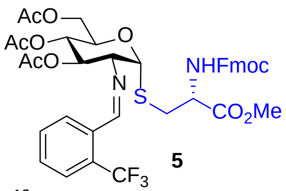

5

^1H-^{13}C HSQC, 600/150MHz, $CDCl_3$

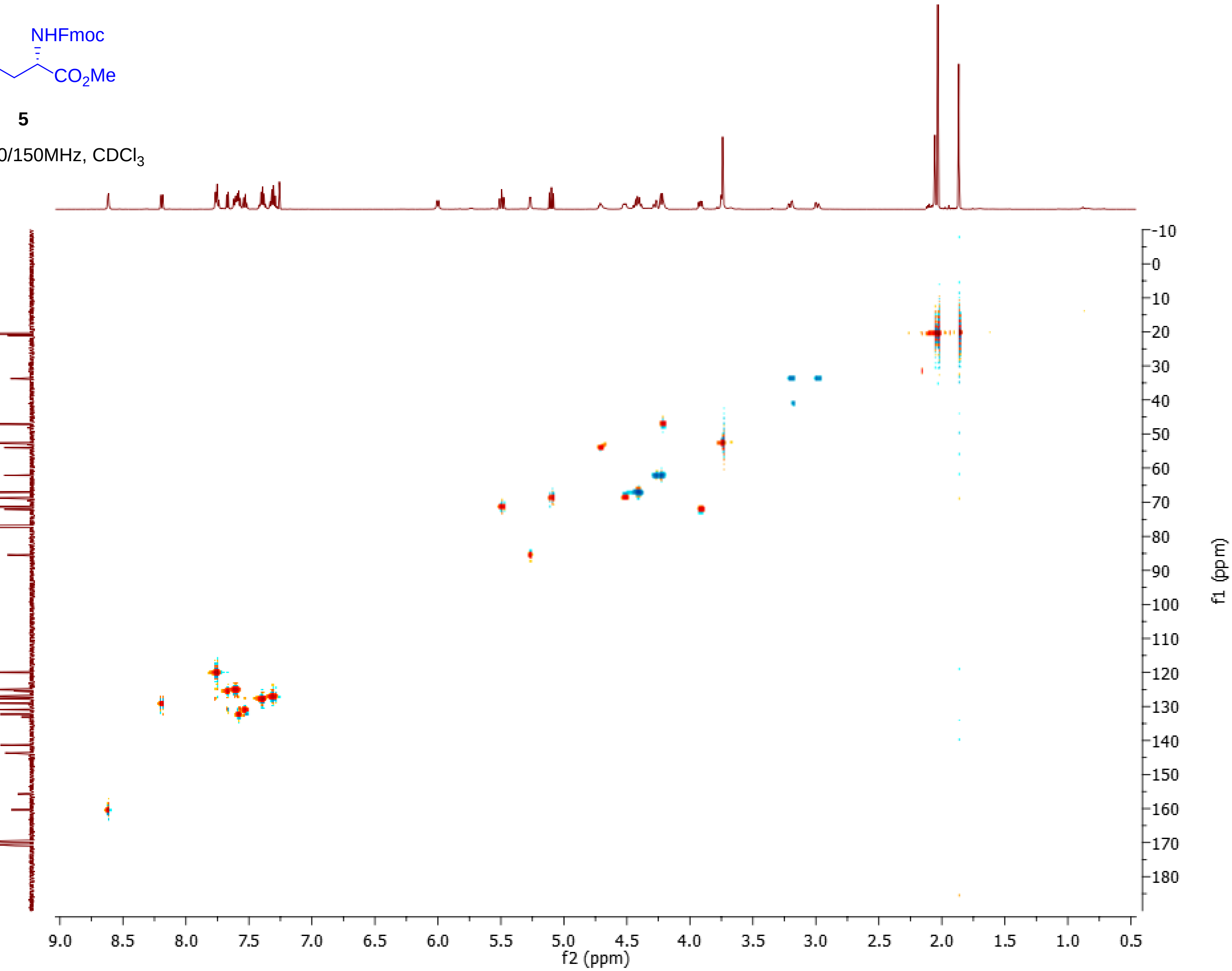

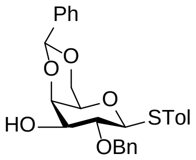

8ii

^{1}H NMR
600 MHz
$CDCl_3$

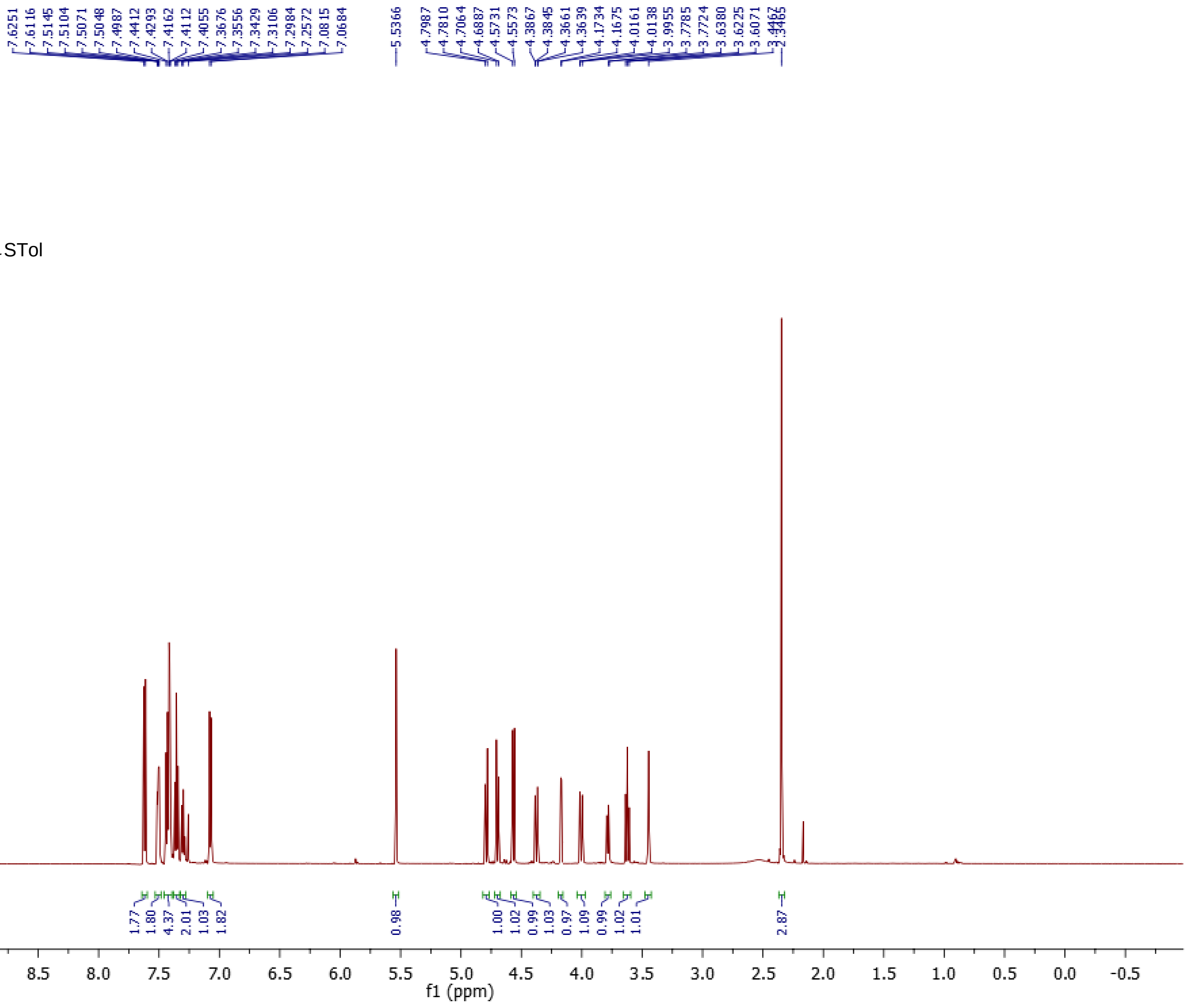

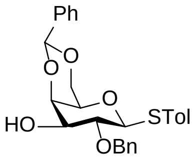

8ii

^{13}C NMR, 150 MHz, $CDCl_3$

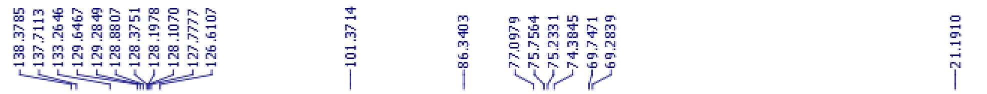

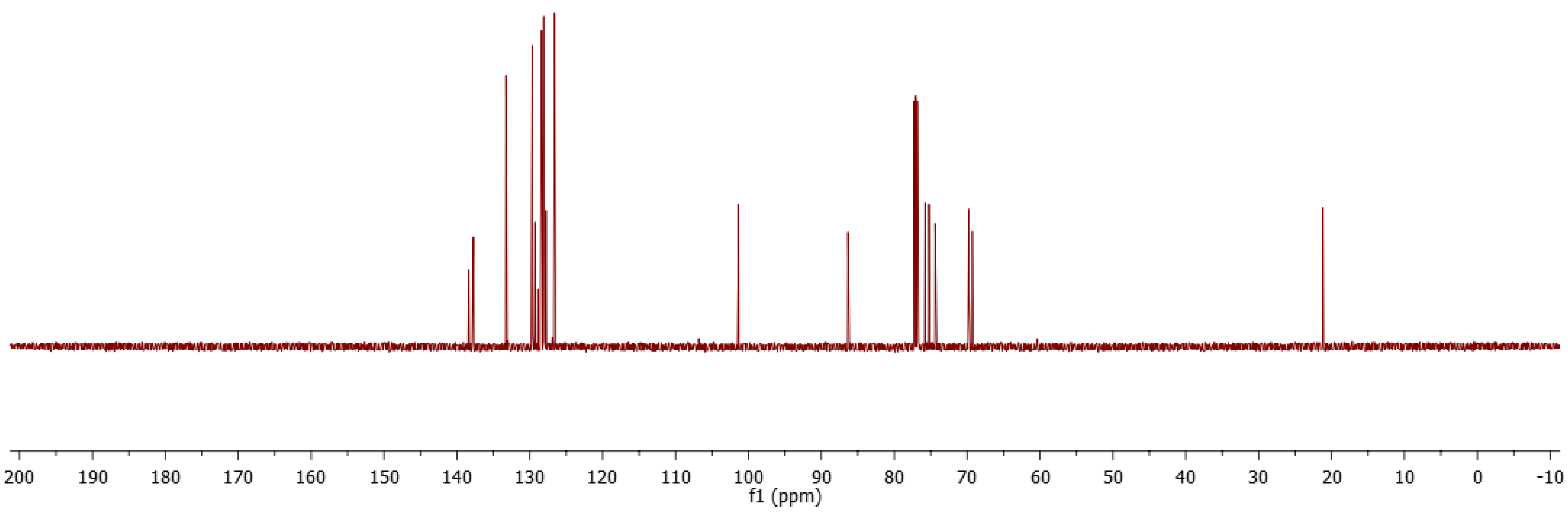

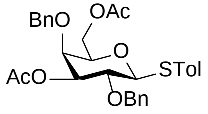

8iii

^{1}H NMR
600 MHz
$CDCl_3$

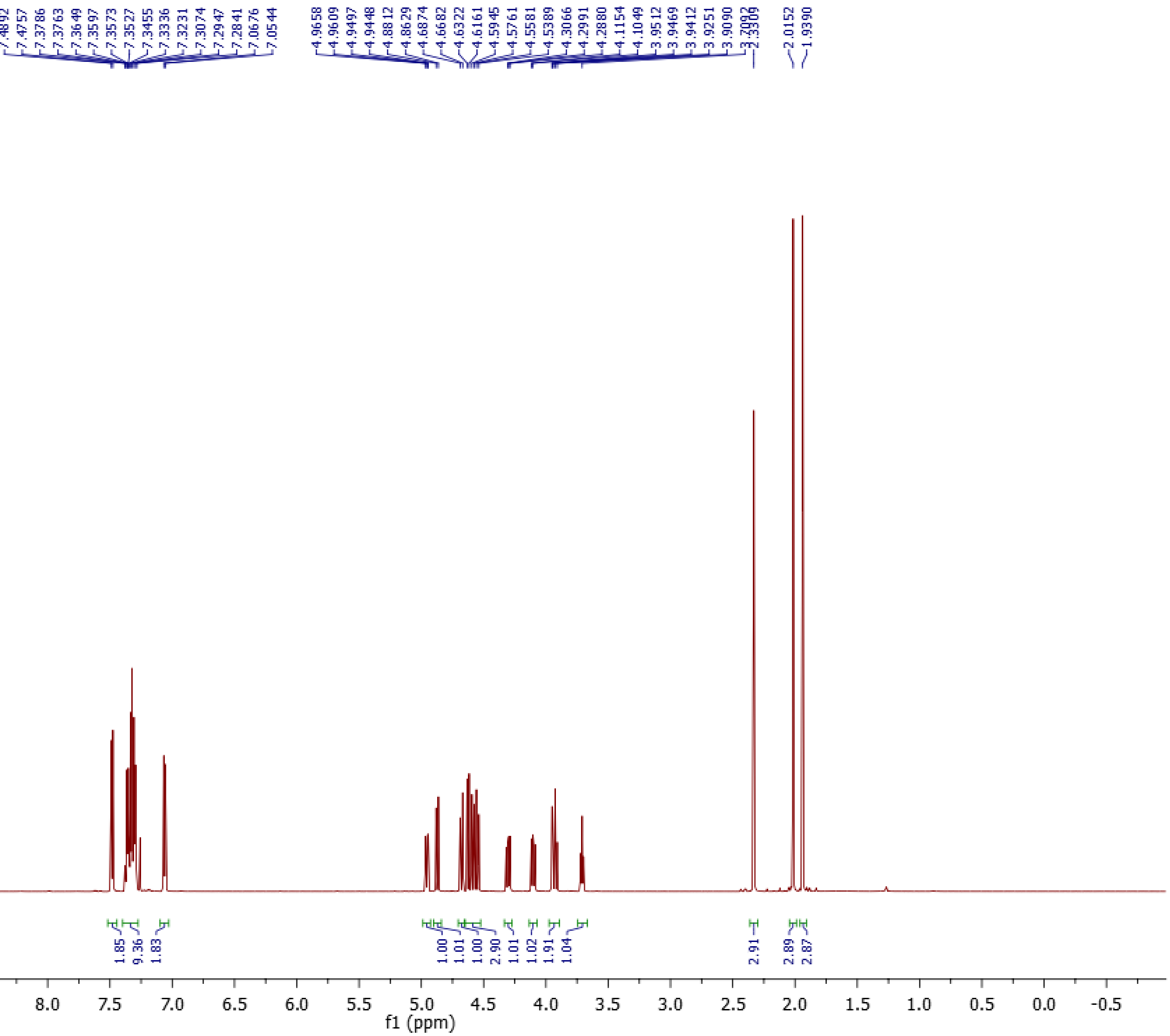

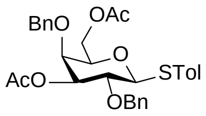

8iii

^{13}C NMR, 150 MHz, $CDCl_3$

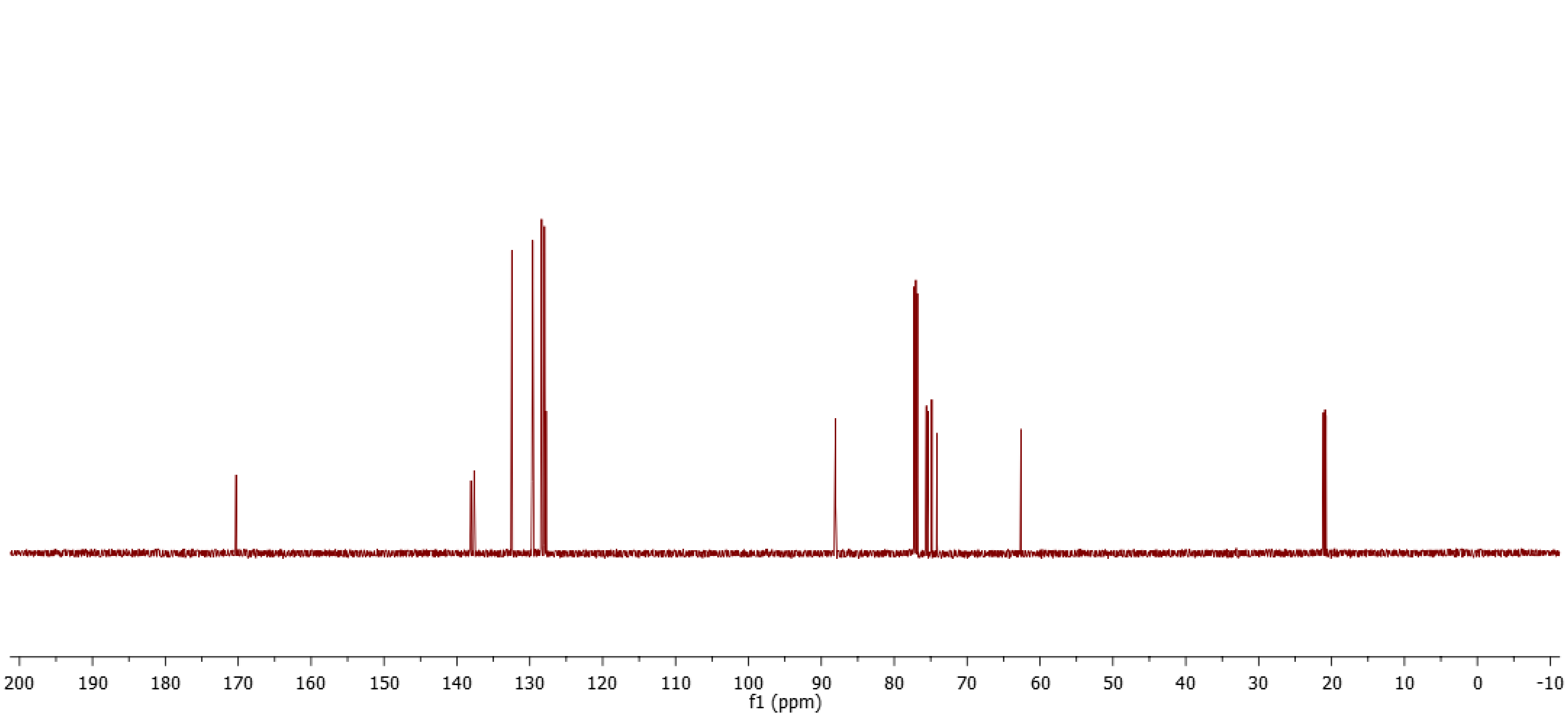

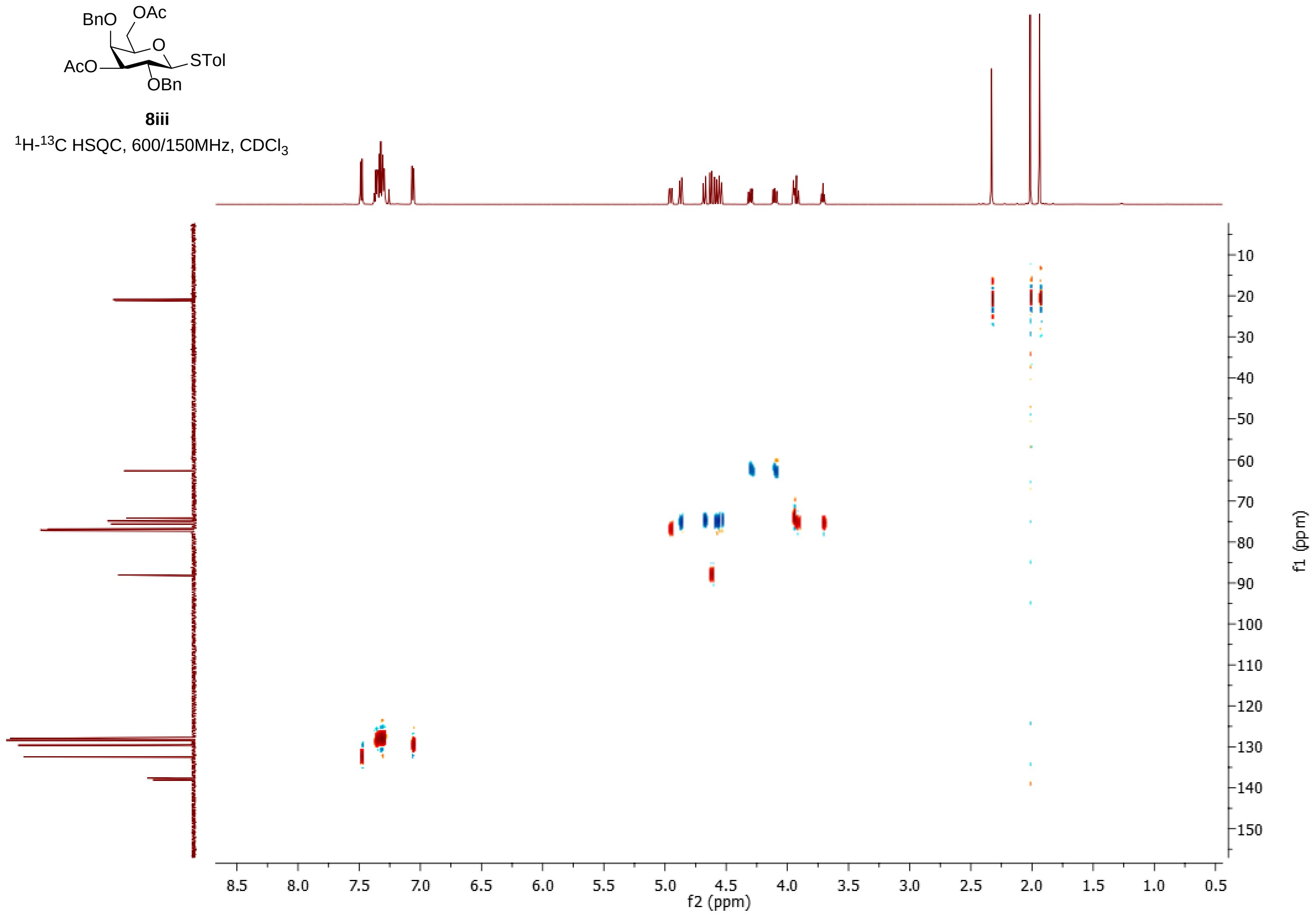
BnO
OAc
O
AcO
STol
OBn
8iii
^{1}H-^{13}C HSQC, 600/150MHz, CDCl$_3$
f2 (ppm)
f1 (ppm)

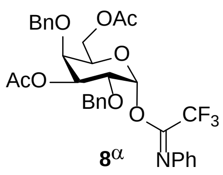

8α

¹H NMR
600 MHz
CDCl3

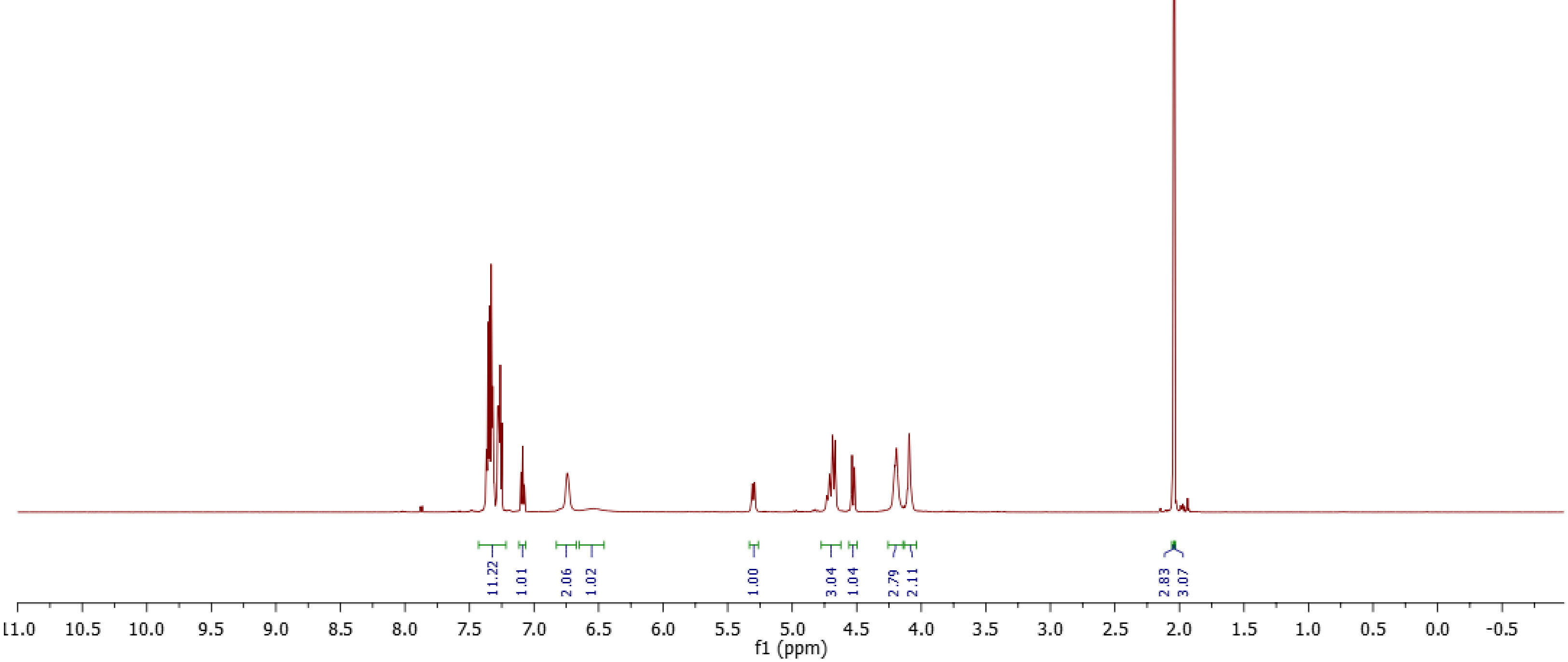

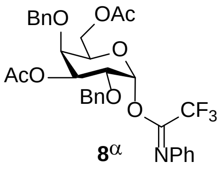

8α

^{13}C NMR, 150 MHz, $CDCl_3$

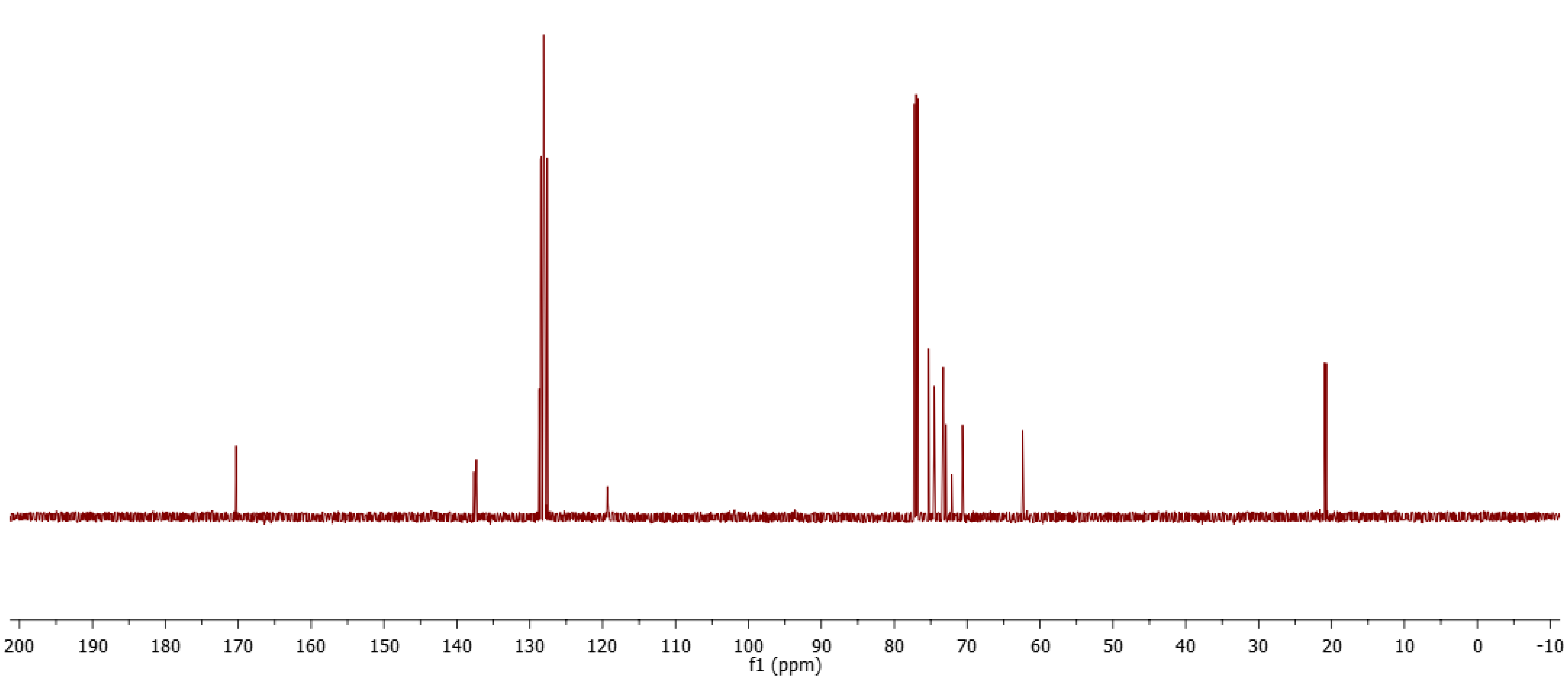

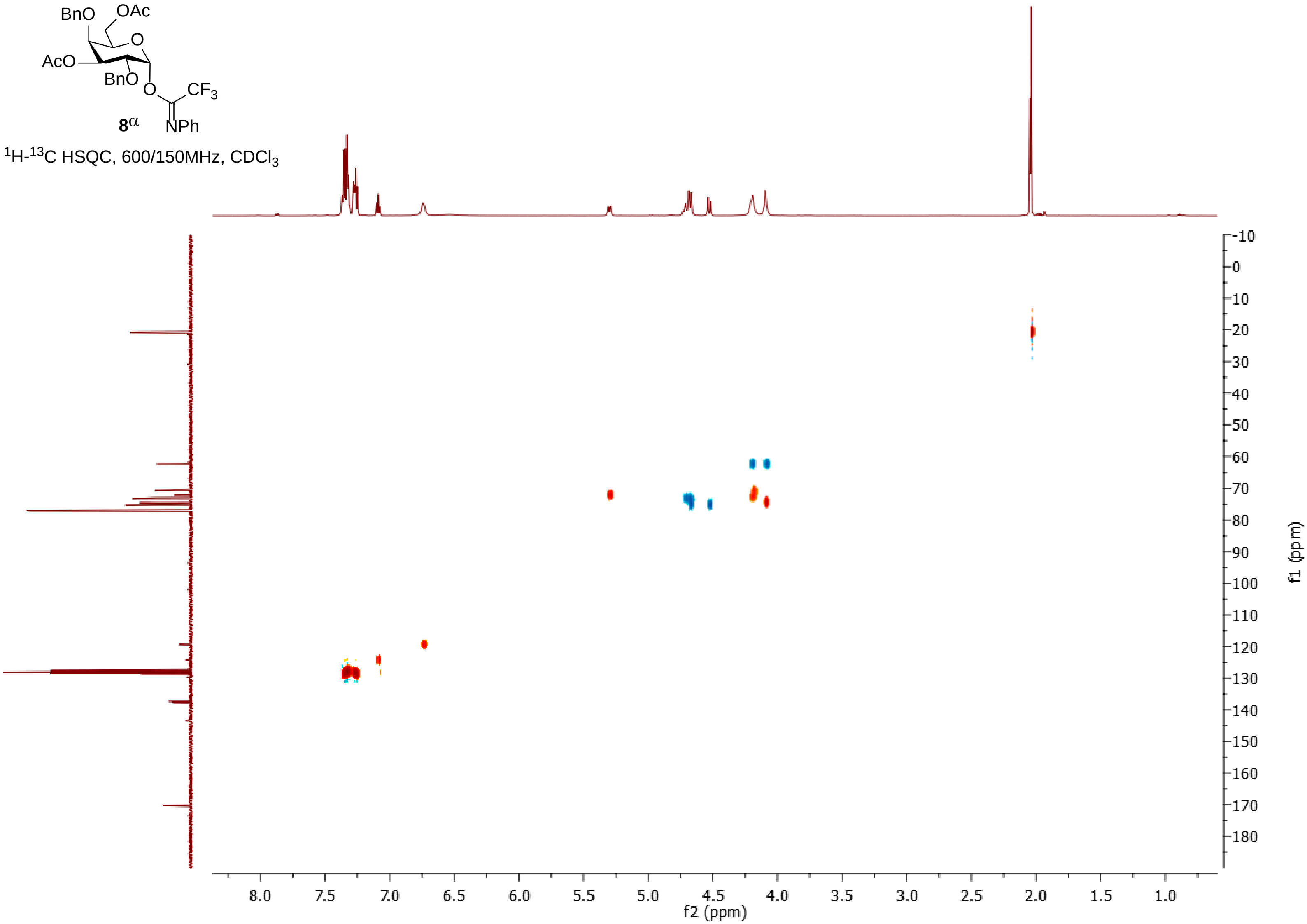
BnO
OAc
O
AcO
BnO
O
CF3
8α
NPh
1H-13C HSQC, 600/150MHz, CDCl3
f2 (ppm)
f1 (ppm)

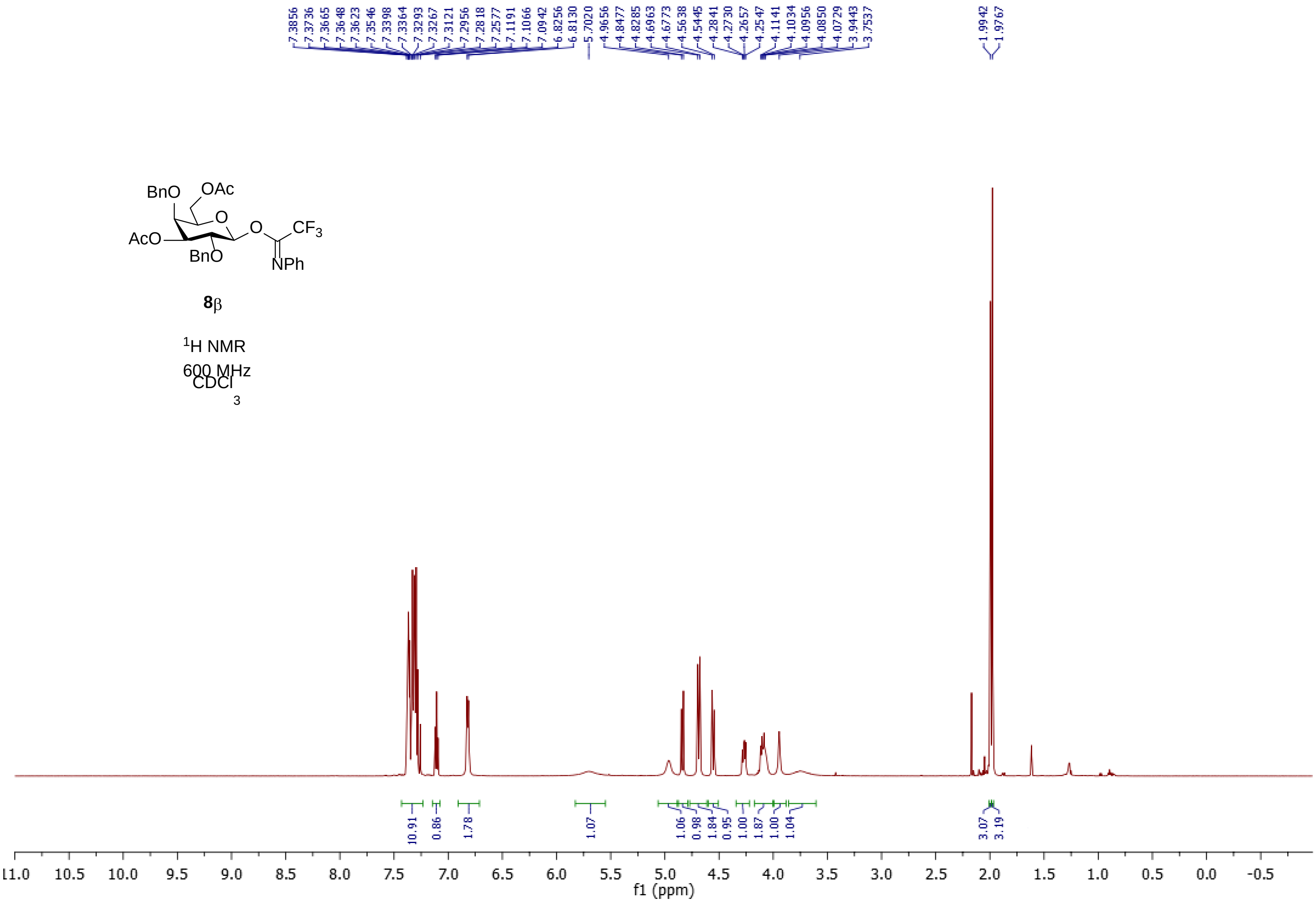

8β

^{1}H NMR
600 MHz
$CDCl_3$

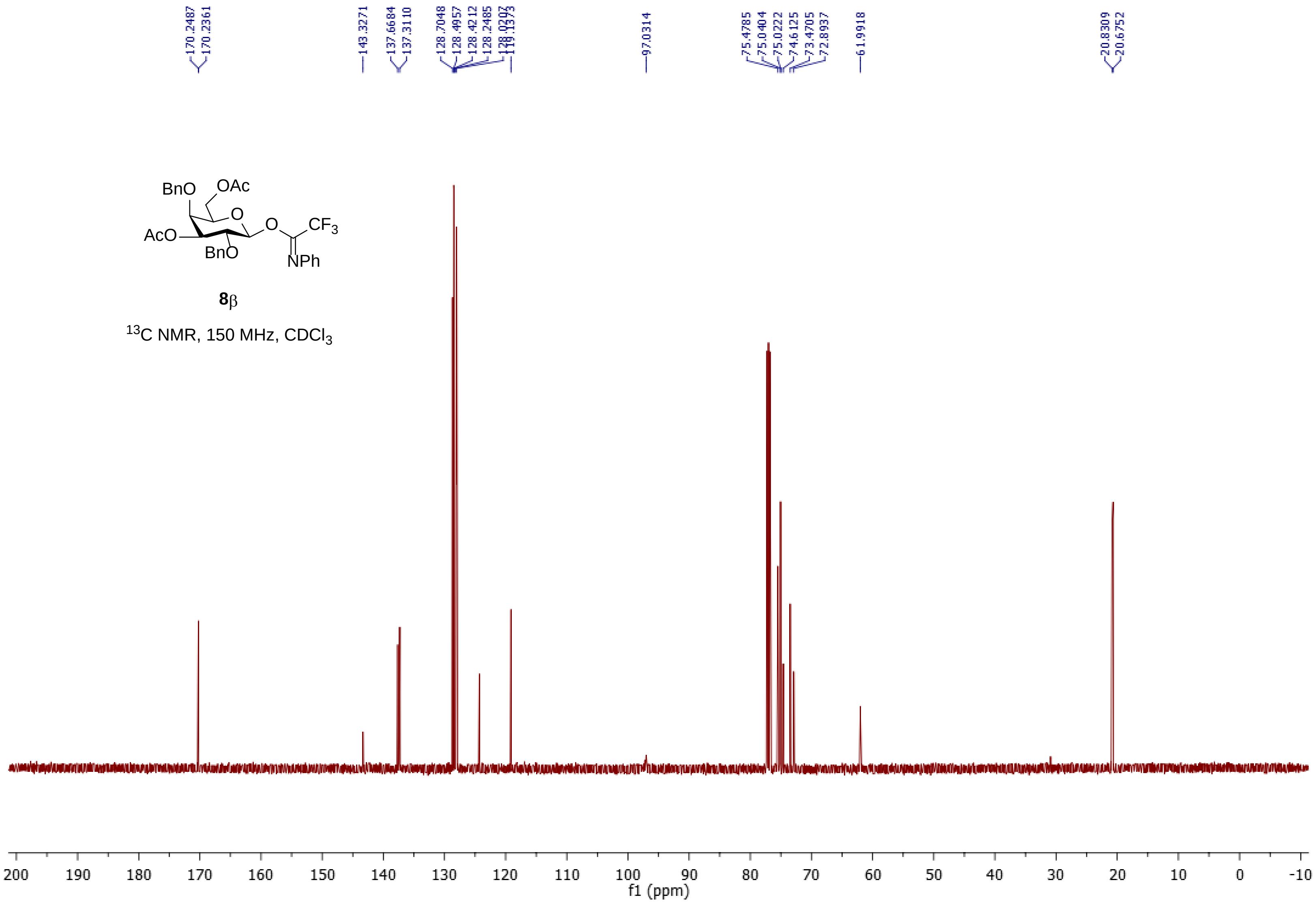
170.2487
170.2361
143.3271
137.6684
137.3110
128.7048
128.4957
128.4212
128.2485
128.0292
119.1373
97.0314
75.4785
75.0404
75.0222
74.6125
73.4705
72.8937
61.9918
20.8309
20.6752
BnO
OAc
O
O
CF3
AcO
BnO
NPh
8β
13C NMR, 150 MHz, CDCl3
200
190
180
170
160
150
140
130
120
110
100
90
80
70
60
50
40
30
20
10
0
-10
f1 (ppm)

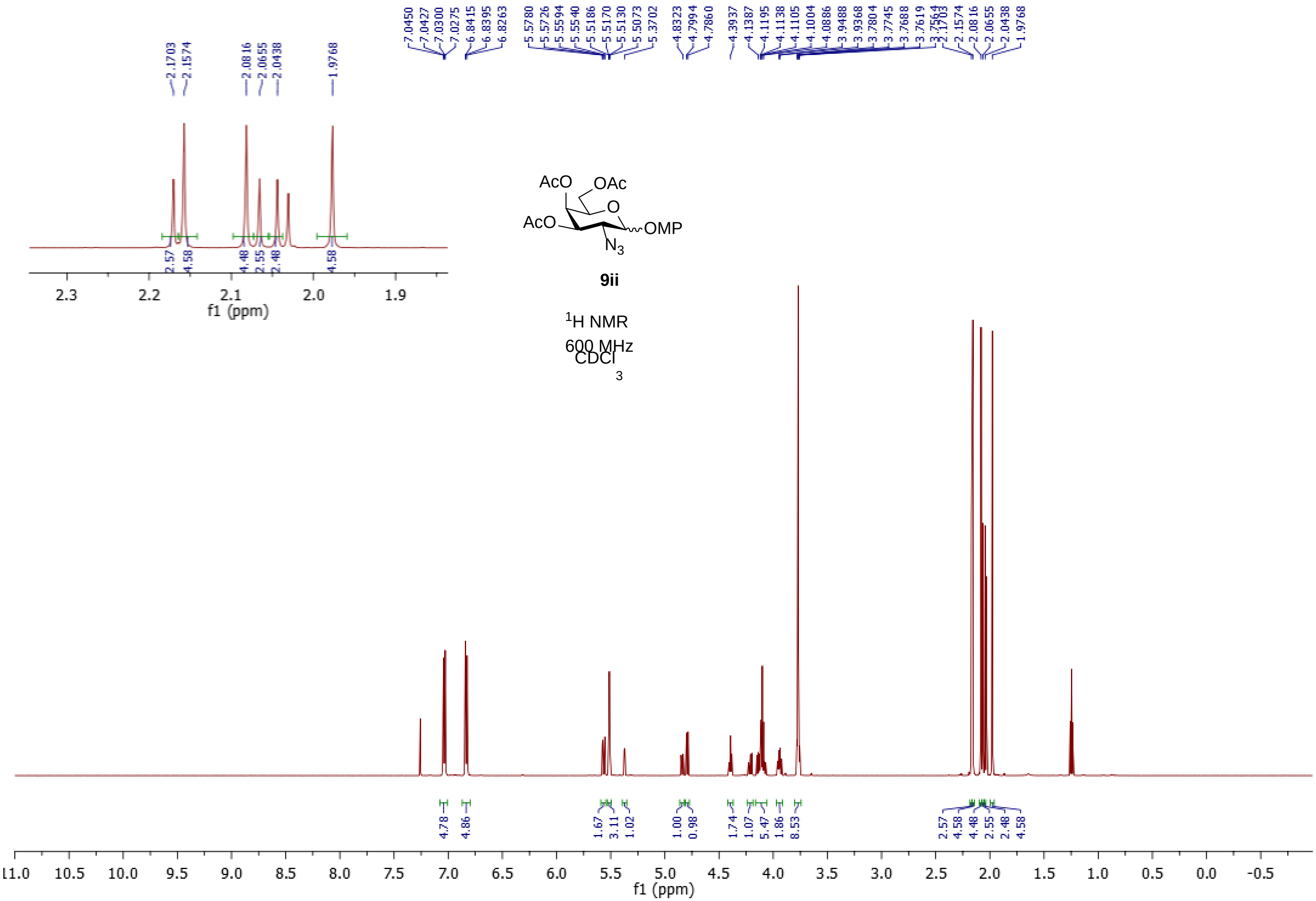

9ii

^{1}H NMR
600 MHz
$CDCl_3$

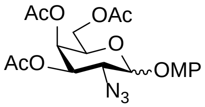

9ii

^{13}C NMR, 150 MHz, $CDCl_3$

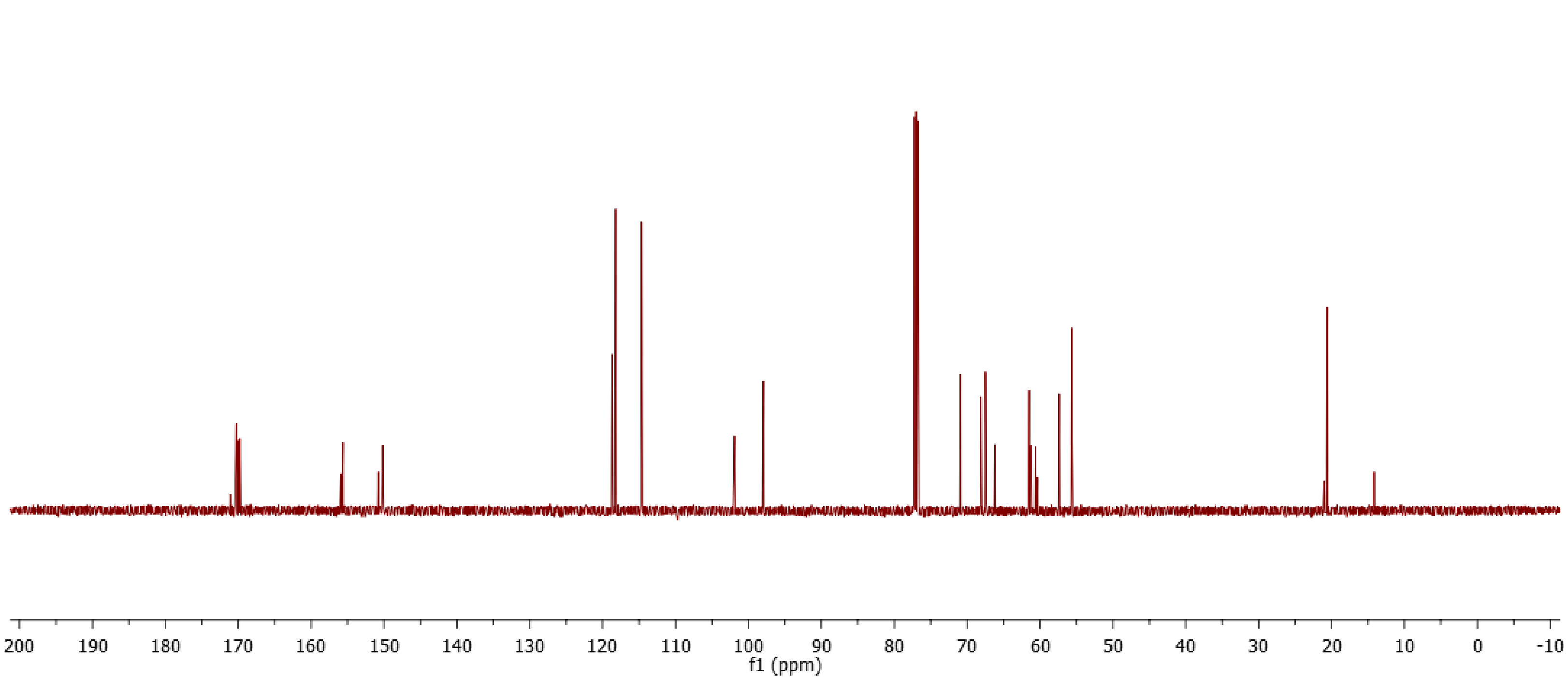

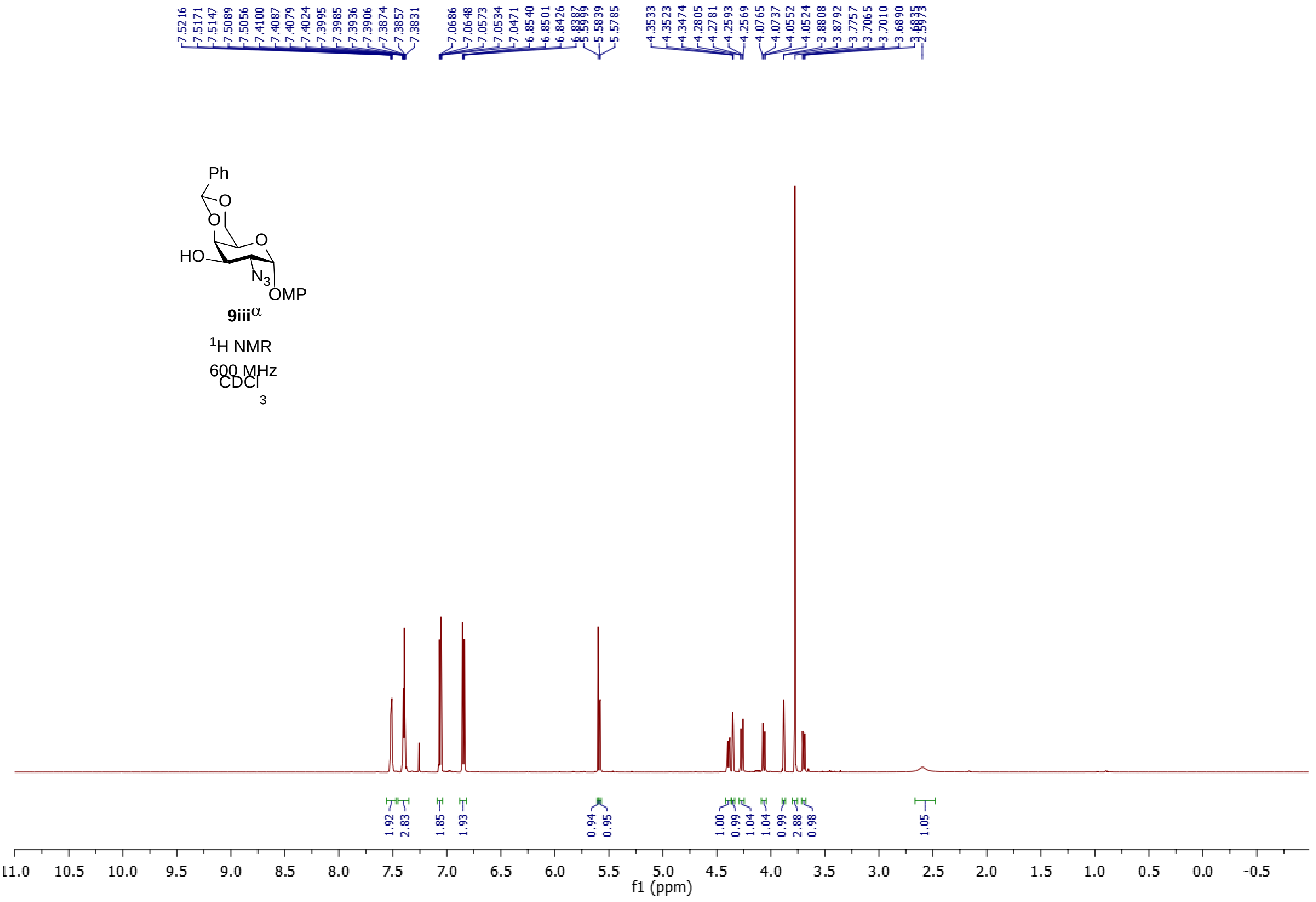
Ph
O
O
O
HO
N3
OMP
9iii α
1H NMR
600 MHz
CDCl3
f1 (ppm)

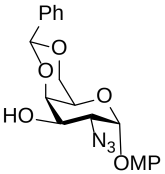

9iii$^{\alpha}$

^{13}C NMR, 150 MHz, $CDCl_3$

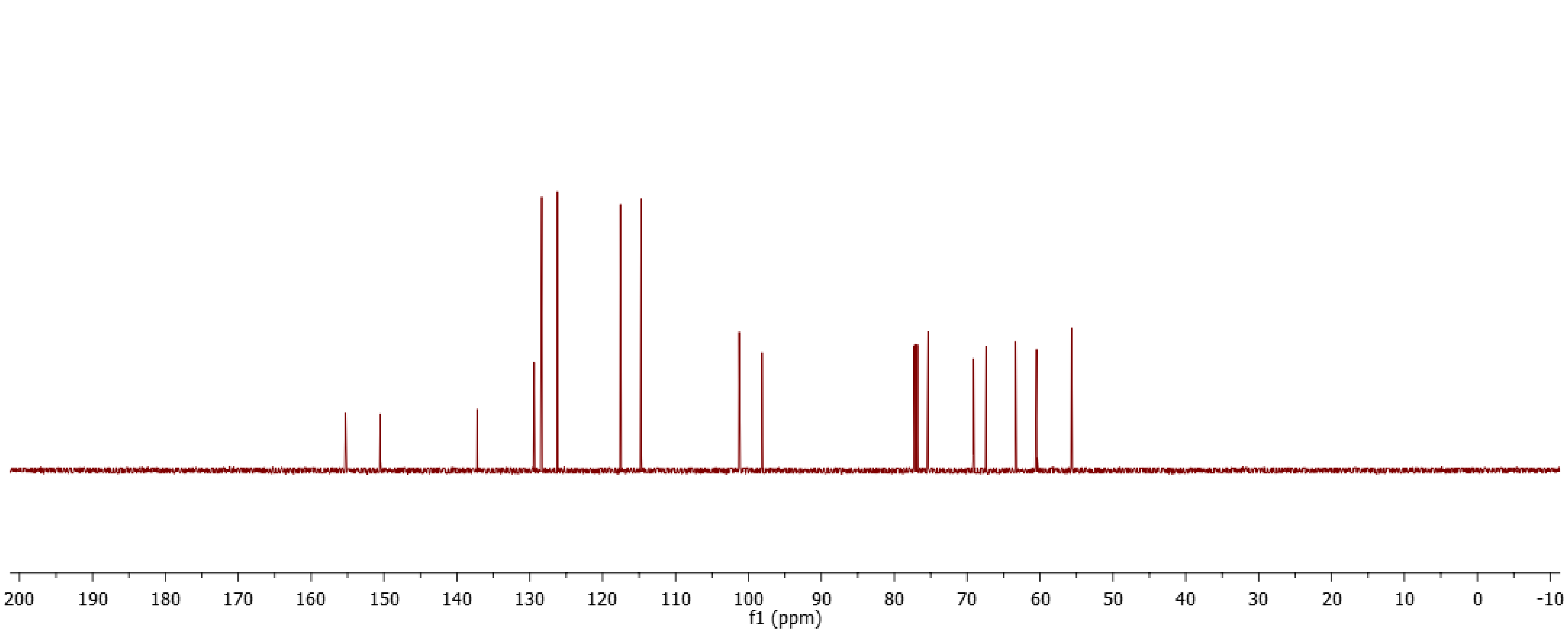

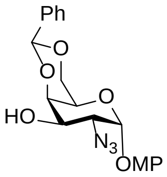

9iii$^{\alpha}$

^{1}H-^{13}C HSQC, 600/150MHz, $CDCl_3$

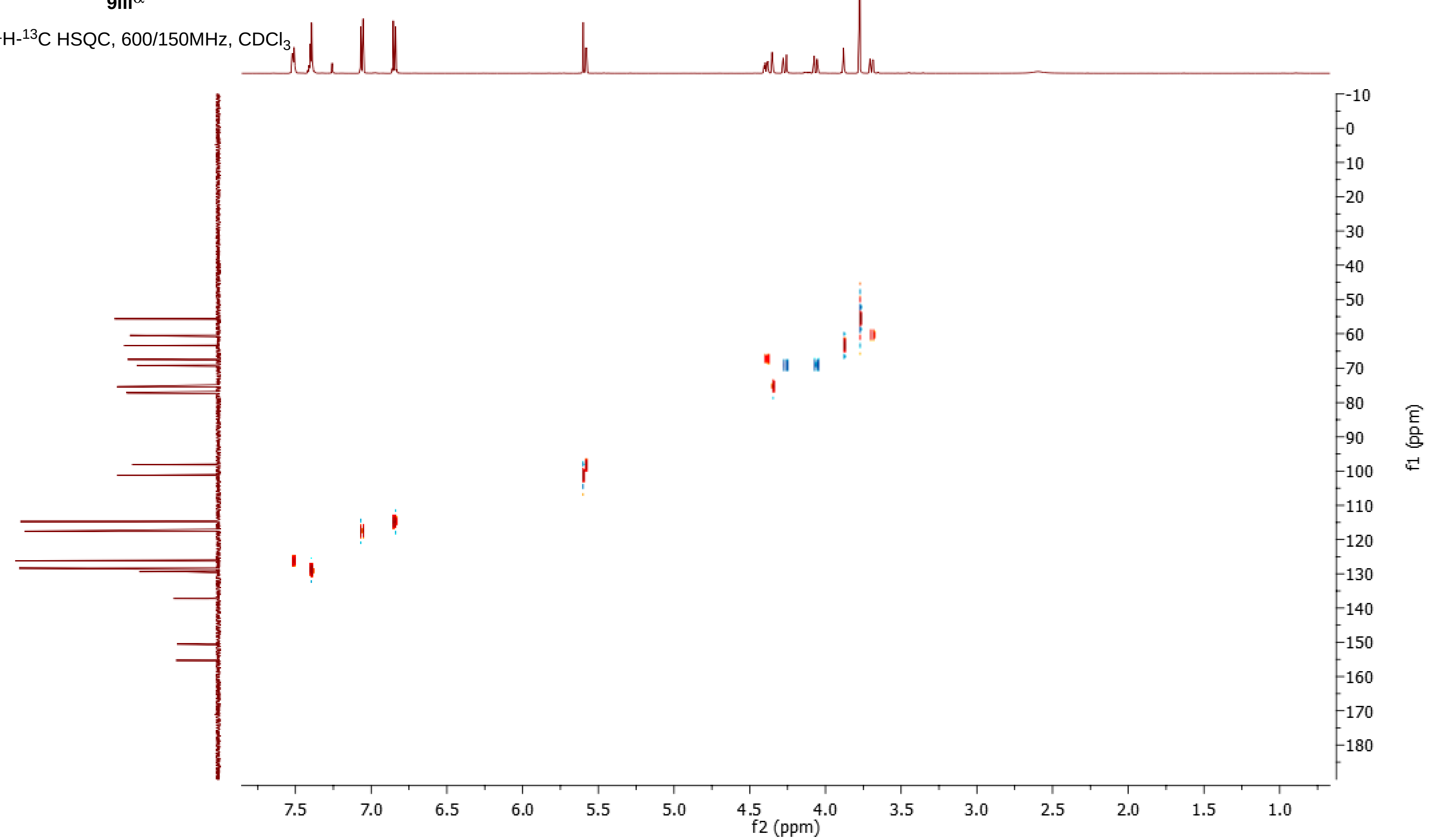

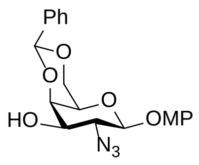

9iiiβ

^{1}H NMR

600 MHz

$CDCl_3$

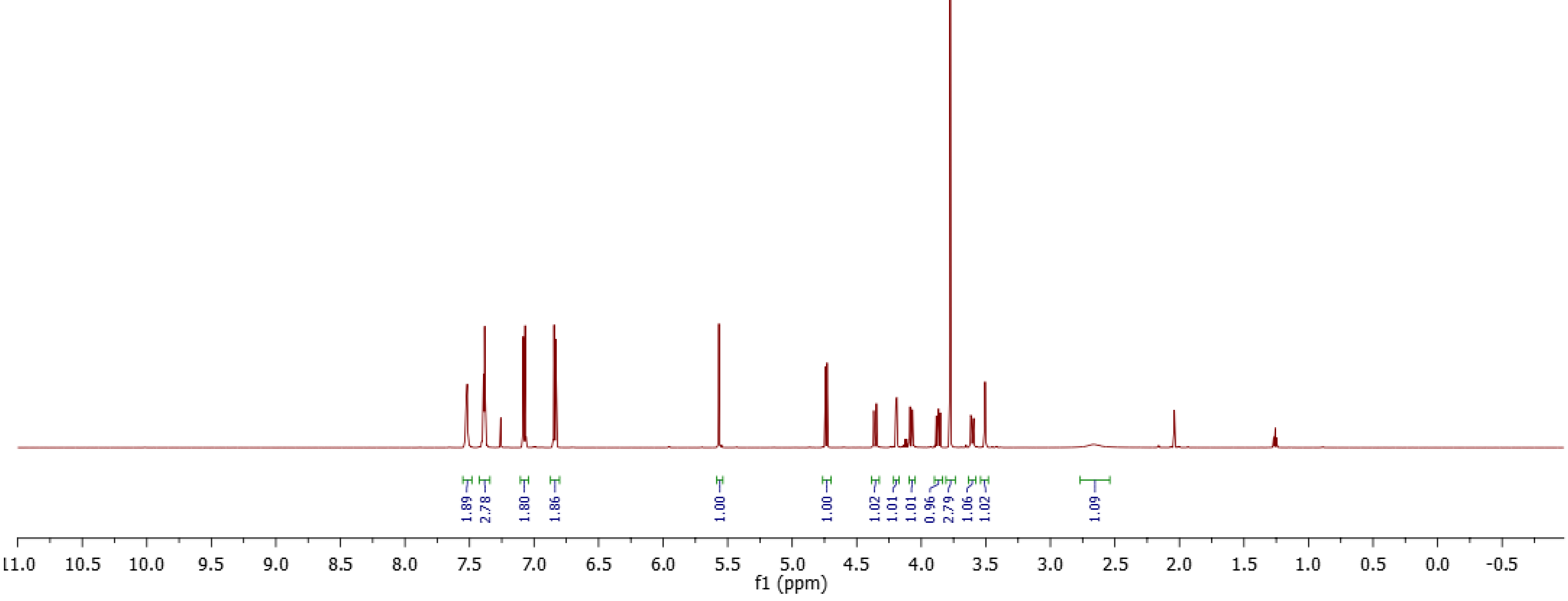

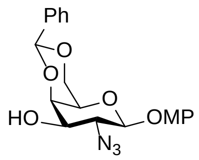

9iiiβ

^{13}C NMR, 150 MHz, $CDCl_3$

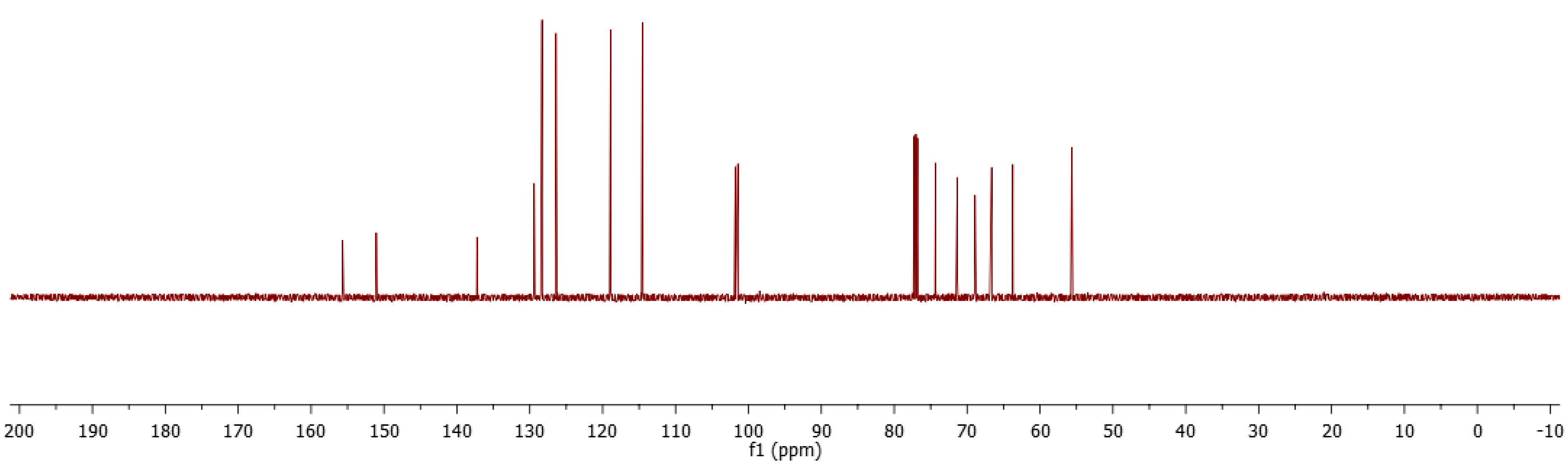

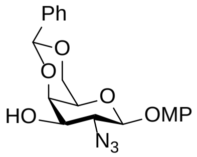

9iiiβ

^{1}H-^{13}C HSQC, 600/150MHz, $CDCl_3$

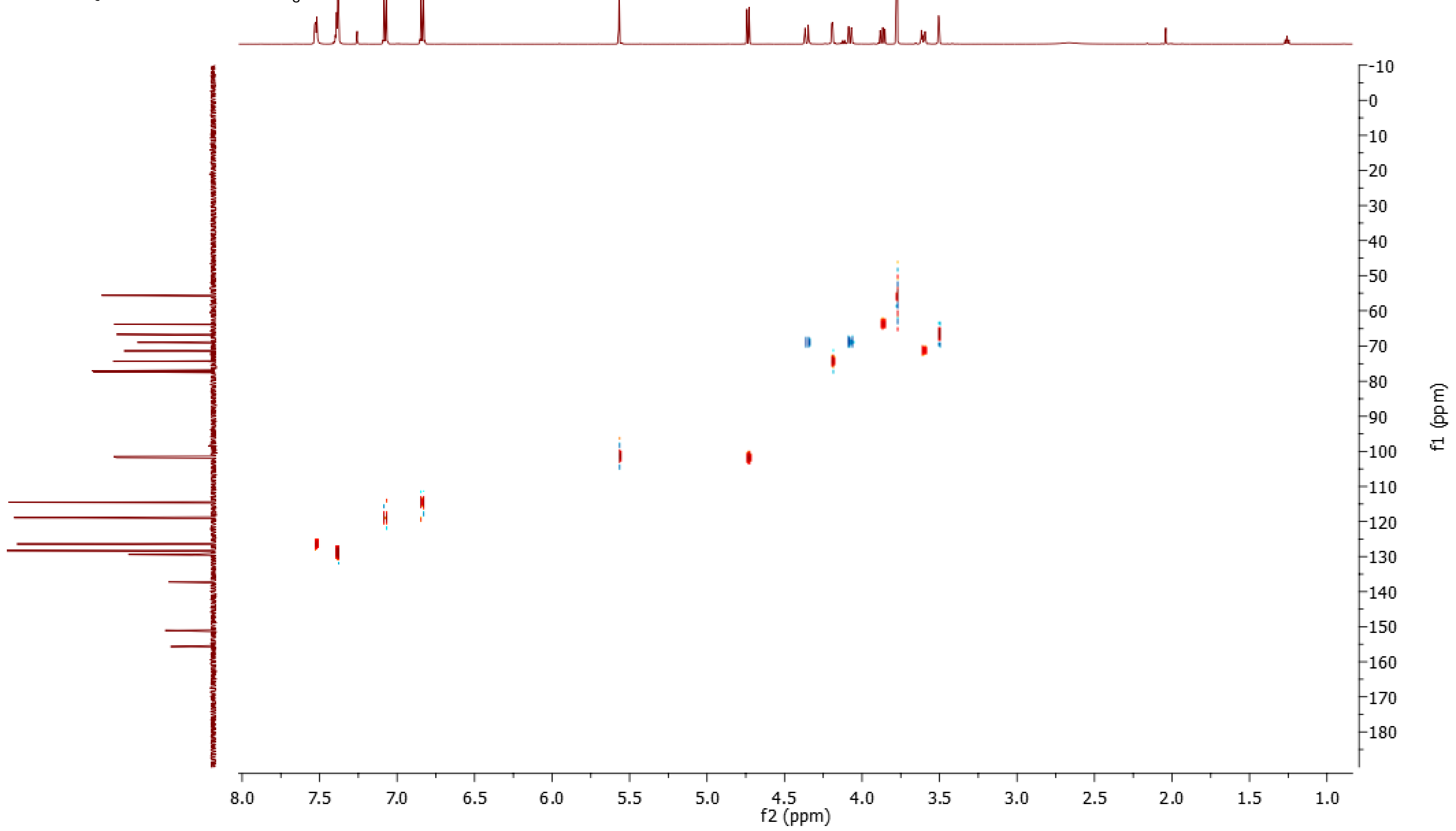

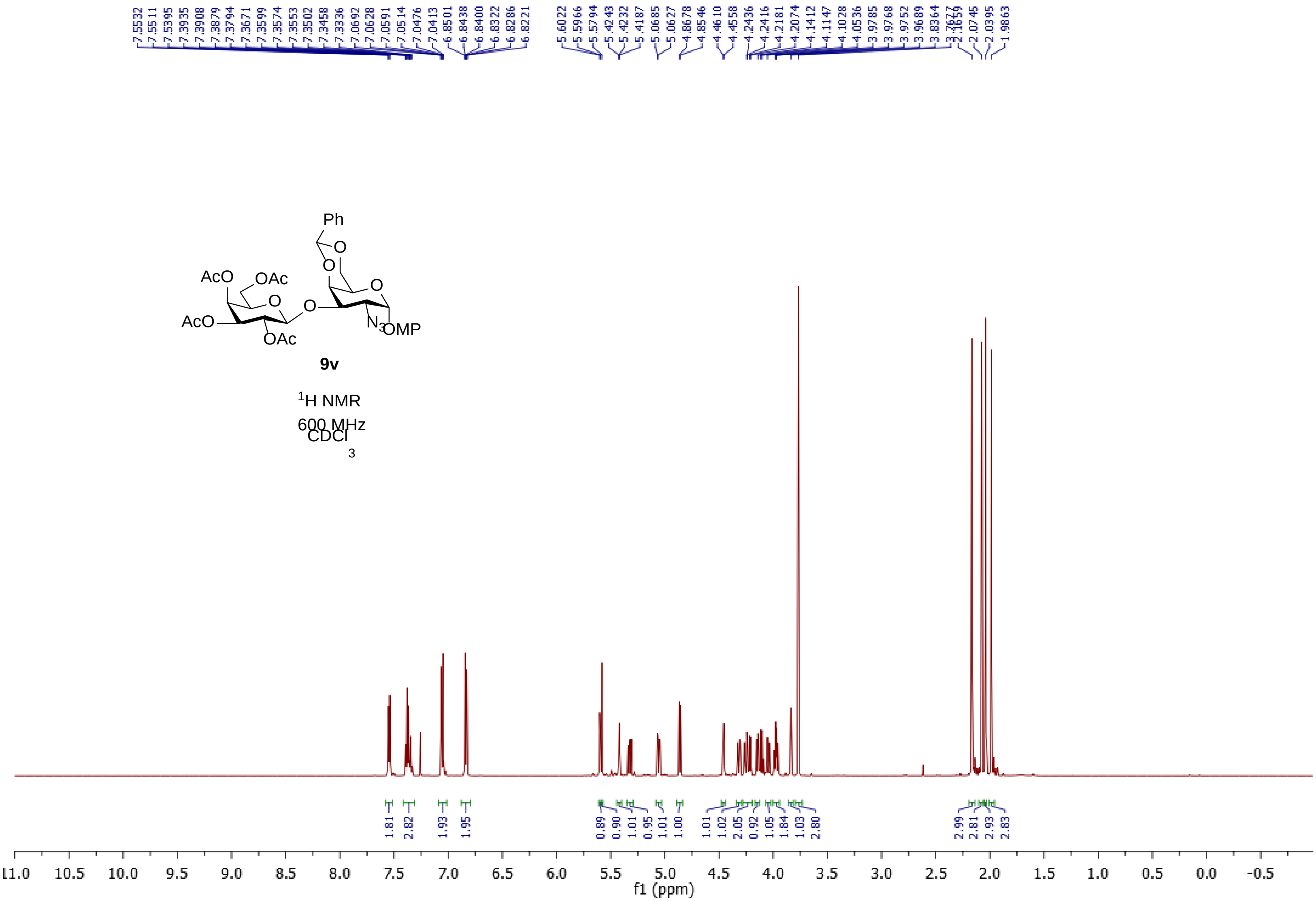
7.5532
7.5511
7.5395
7.3935
7.3908
7.3879
7.3794
7.3671
7.3599
7.3574
7.3553
7.3502
7.3458
7.3336
7.0692
7.0628
7.0591
7.0514
7.0476
7.0413
6.8501
6.8438
6.8400
6.8322
6.8286
6.8221
5.6022
5.5966
5.5794
5.4243
5.4232
5.4187
5.0685
5.0627
4.8678
4.8546
4.4610
4.4558
4.2436
4.2416
4.2181
4.2074
4.1412
4.1147
4.1028
4.0536
3.9785
3.9768
3.9752
3.9689
3.8364
3.7677
2.1659
2.0745
2.0395
1.9863
Ph
O
O
AcO
OAc
O
O
AcO
O
OAc
N3
OMP
9v
1H NMR
600 MHz
CDCl3
1.81
2.82
1.93
1.95
0.89
0.90
1.01
0.95
1.01
1.00
1.01
1.02
2.05
0.92
1.05
1.84
1.03
2.80
2.99
2.81
2.93
2.83
11.0
10.5
10.0
9.5
9.0
8.5
8.0
7.5
7.0
6.5
6.0
5.5
5.0
4.5
4.0
3.5
3.0
2.5
2.0
1.5
1.0
0.5
0.0
-0.5
f1 (ppm)

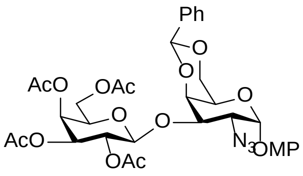

9v

^{13}C NMR, 150 MHz, $CDCl_3$

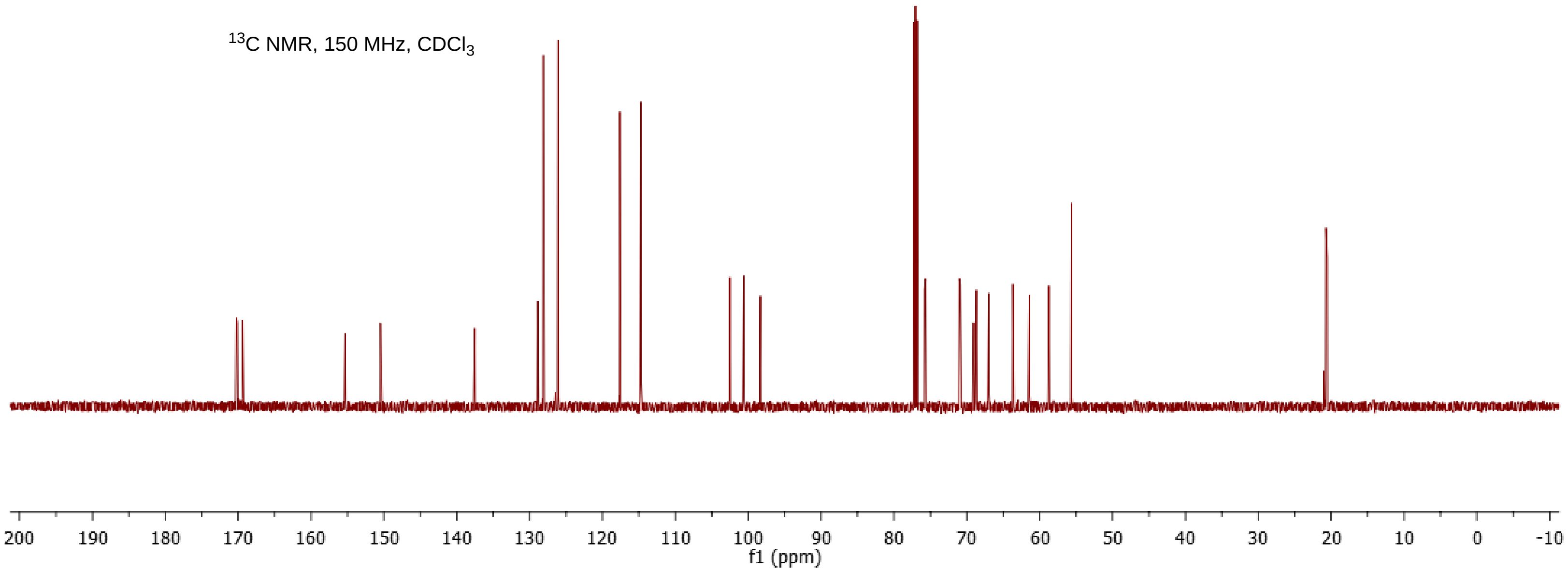

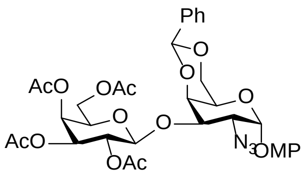

9v

^{1}H-^{13}C HSQC, 600/150MHz, $CDCl_3$

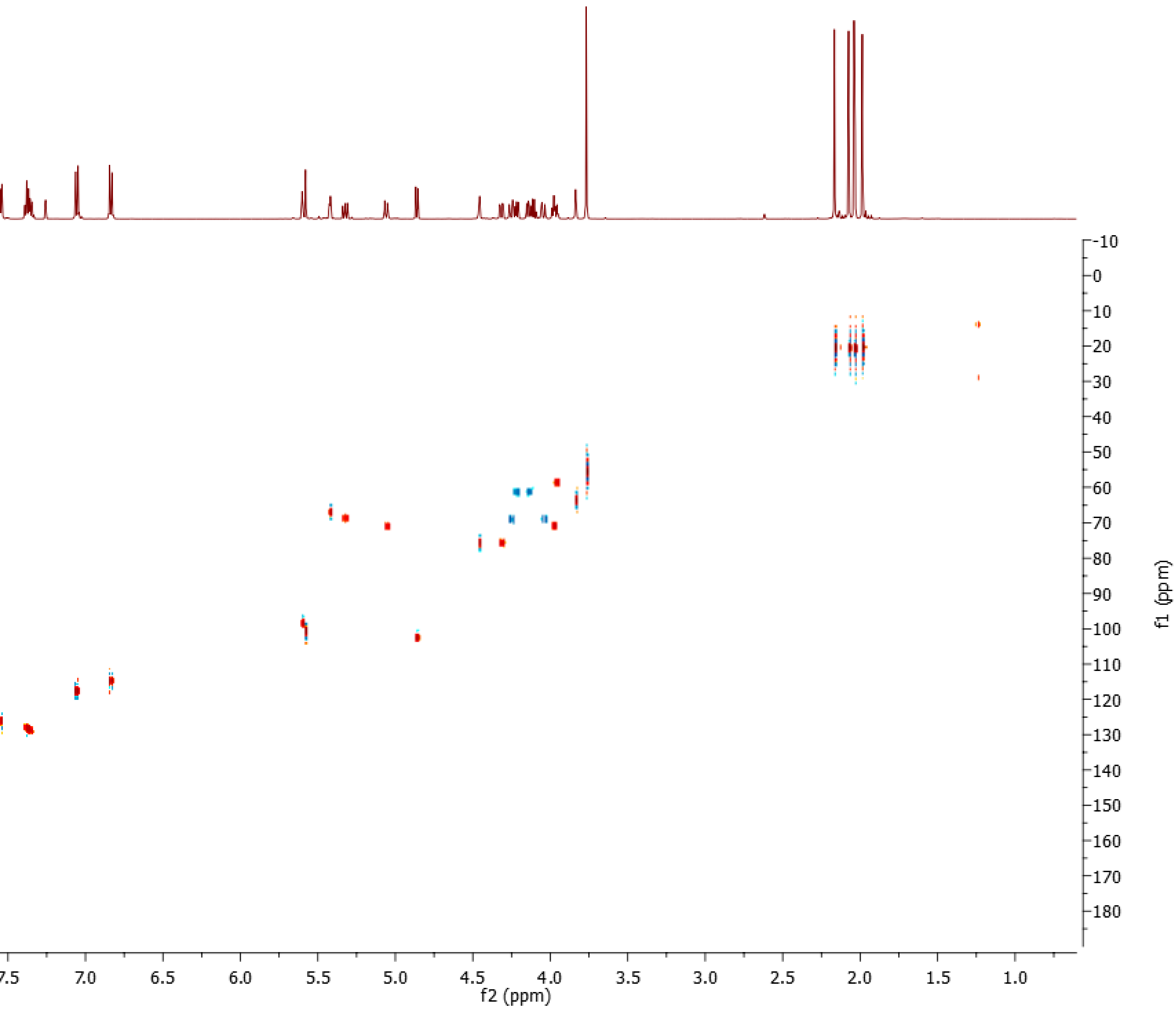

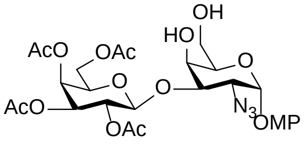

9vi

^{1}H NMR
600 MHz
$CDCl_3$

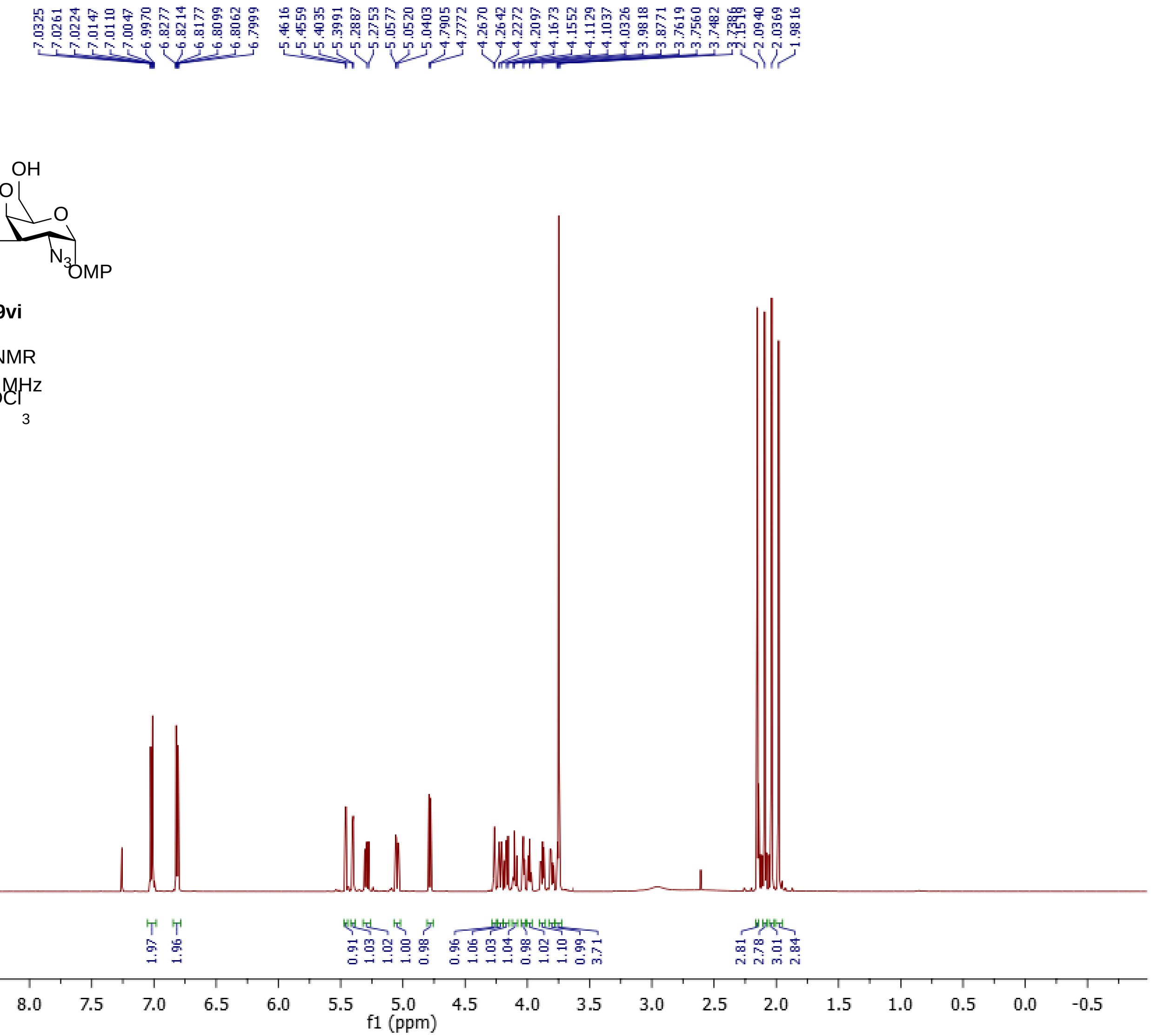

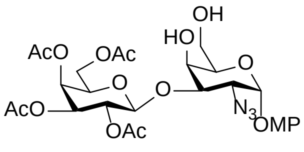

9vi

^{13}C NMR, 150 MHz, $CDCl_3$

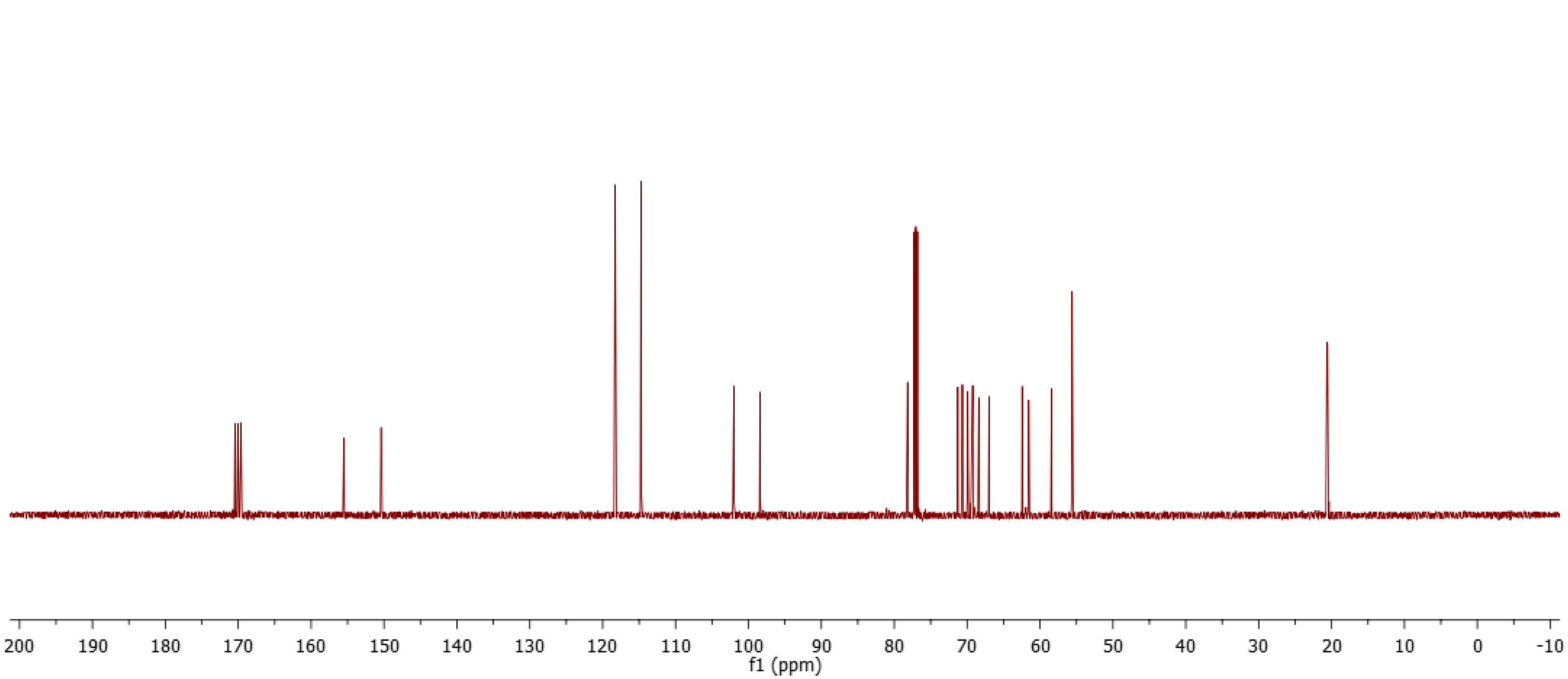

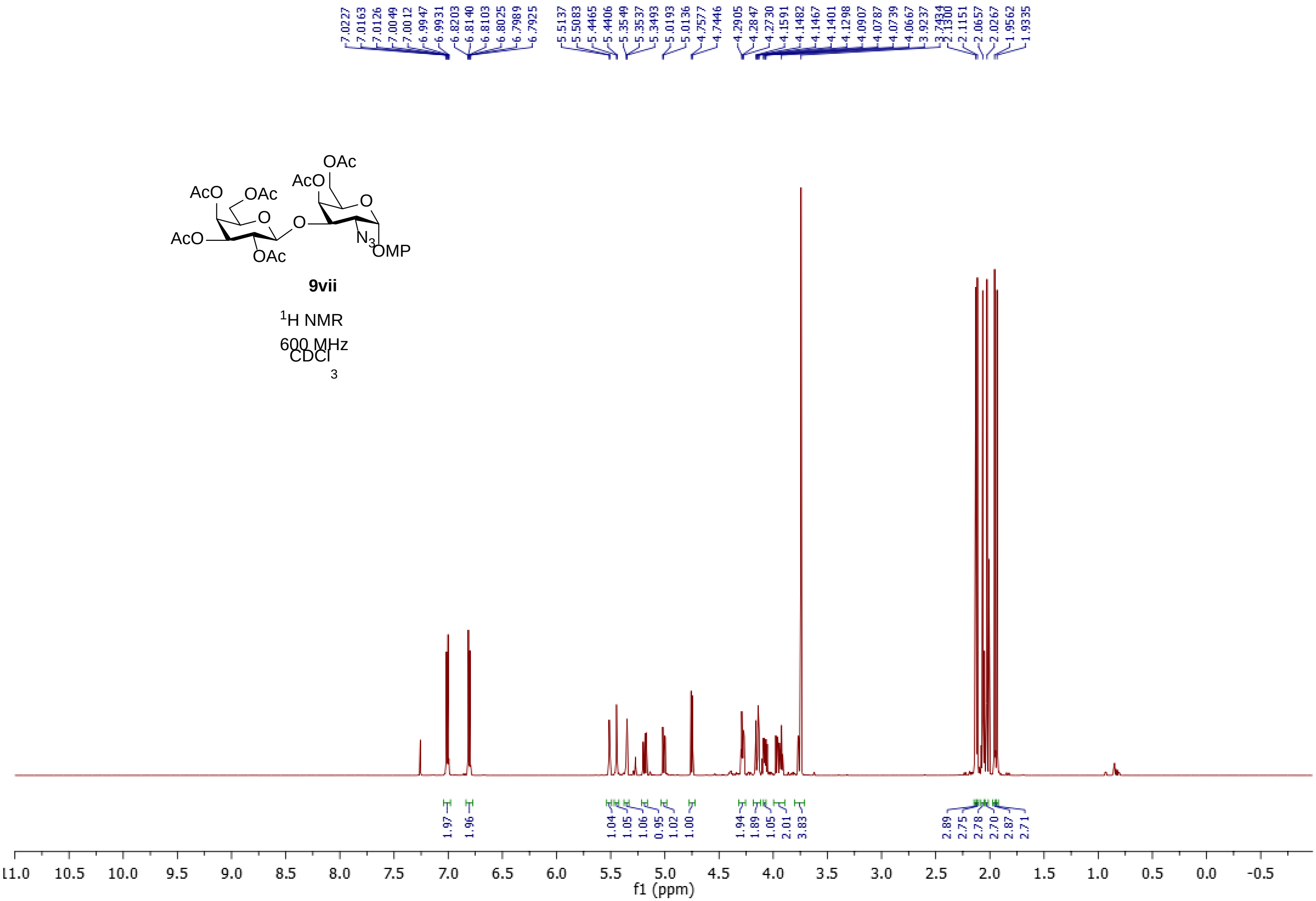

OAc
AcO
AcO
OAc
O
O
AcO
OAc
O
N3
OMP
9vii
1H NMR
600 MHz
CDCl3
7.0227
7.0163
7.0126
7.0049
7.0012
6.9947
6.9931
6.8203
6.8140
6.8103
6.8025
6.7989
6.7925
5.5137
5.5083
5.4465
5.4406
5.3549
5.3537
5.3493
5.0193
5.0136
4.7577
4.7446
4.2905
4.2847
4.2730
4.1591
4.1482
4.1467
4.1401
4.1298
4.0907
4.0787
4.0739
4.0667
3.9237
3.7434
2.1300
2.1151
2.0657
2.0267
1.9562
1.9335
1.97
1.96
1.04
1.05
1.06
0.95
1.02
1.00
1.94
1.89
1.05
2.01
3.83
2.89
2.75
2.78
2.70
2.87
2.71
11.0
10.5
10.0
9.5
9.0
8.5
8.0
7.5
7.0
6.5
6.0
5.5
5.0
4.5
4.0
3.5
3.0
2.5
2.0
1.5
1.0
0.5
0.0
-0.5
f1 (ppm)

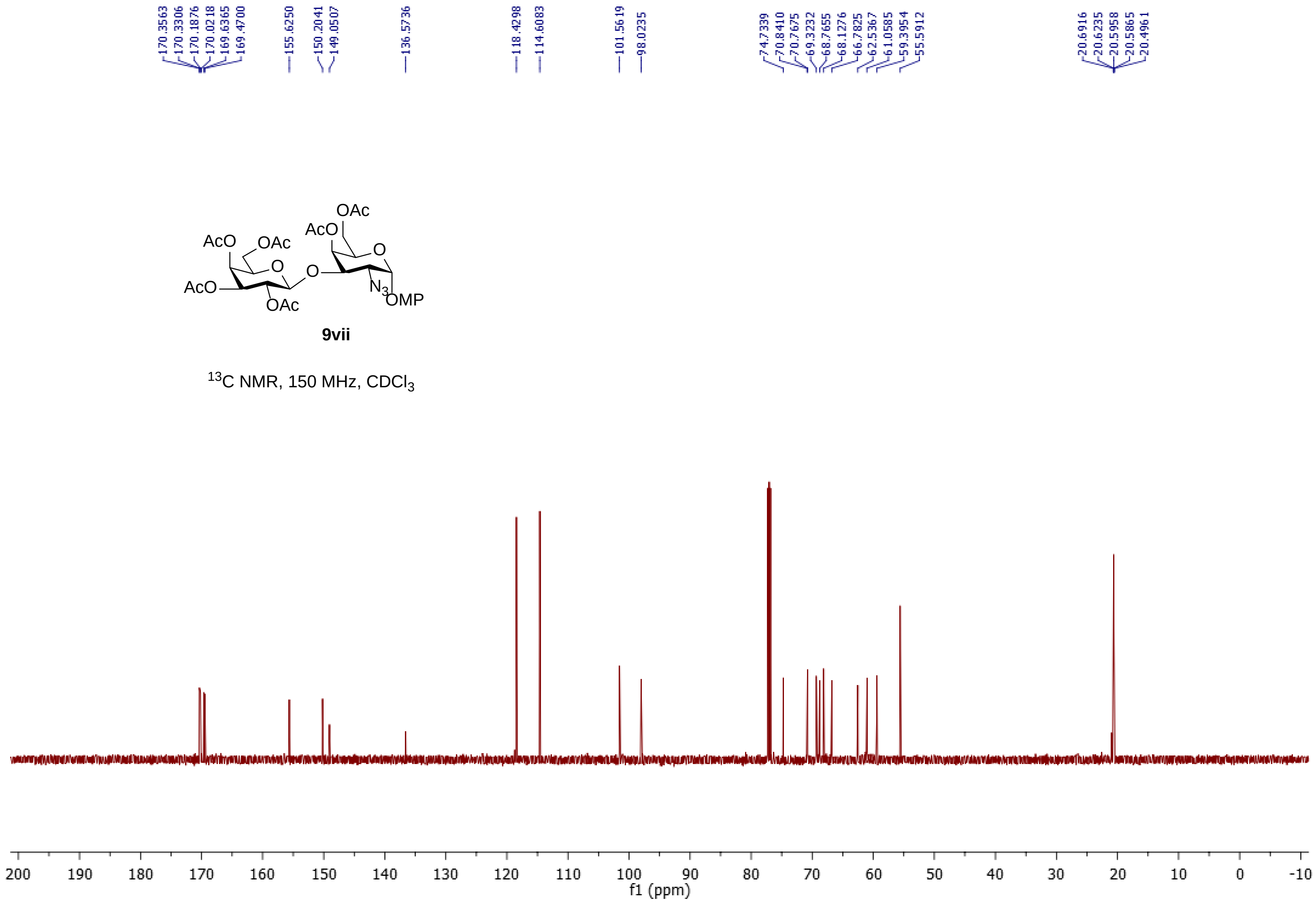

170.3563
170.3306
170.1876
170.0218
169.6365
169.4700
155.6250
150.2041
149.0507
136.5736
118.4298
114.6083
101.5619
98.0235
74.7339
70.8410
70.7675
69.3232
68.7655
68.1276
66.7825
62.5367
61.0585
59.3954
55.5912
20.6916
20.6235
20.5958
20.5865
20.4961
AcO
OAc
AcO
OAc
AcO
O
O
O
OAc
N3
OMP
9vii
^{13}C NMR, 150 MHz, $CDCl_3$
f1 (ppm)

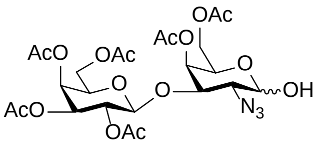

9ix

^{1}H NMR
600 MHz
$CDCl_3$

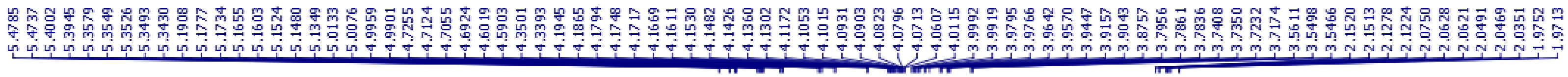

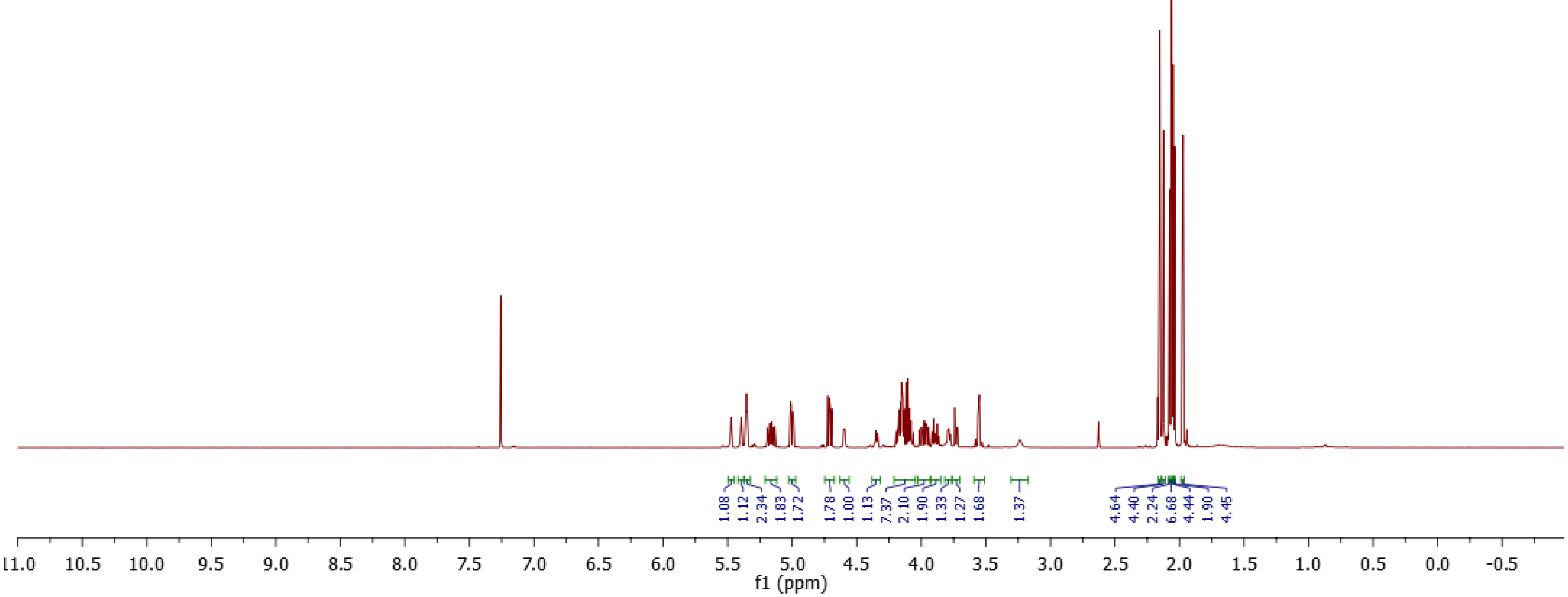

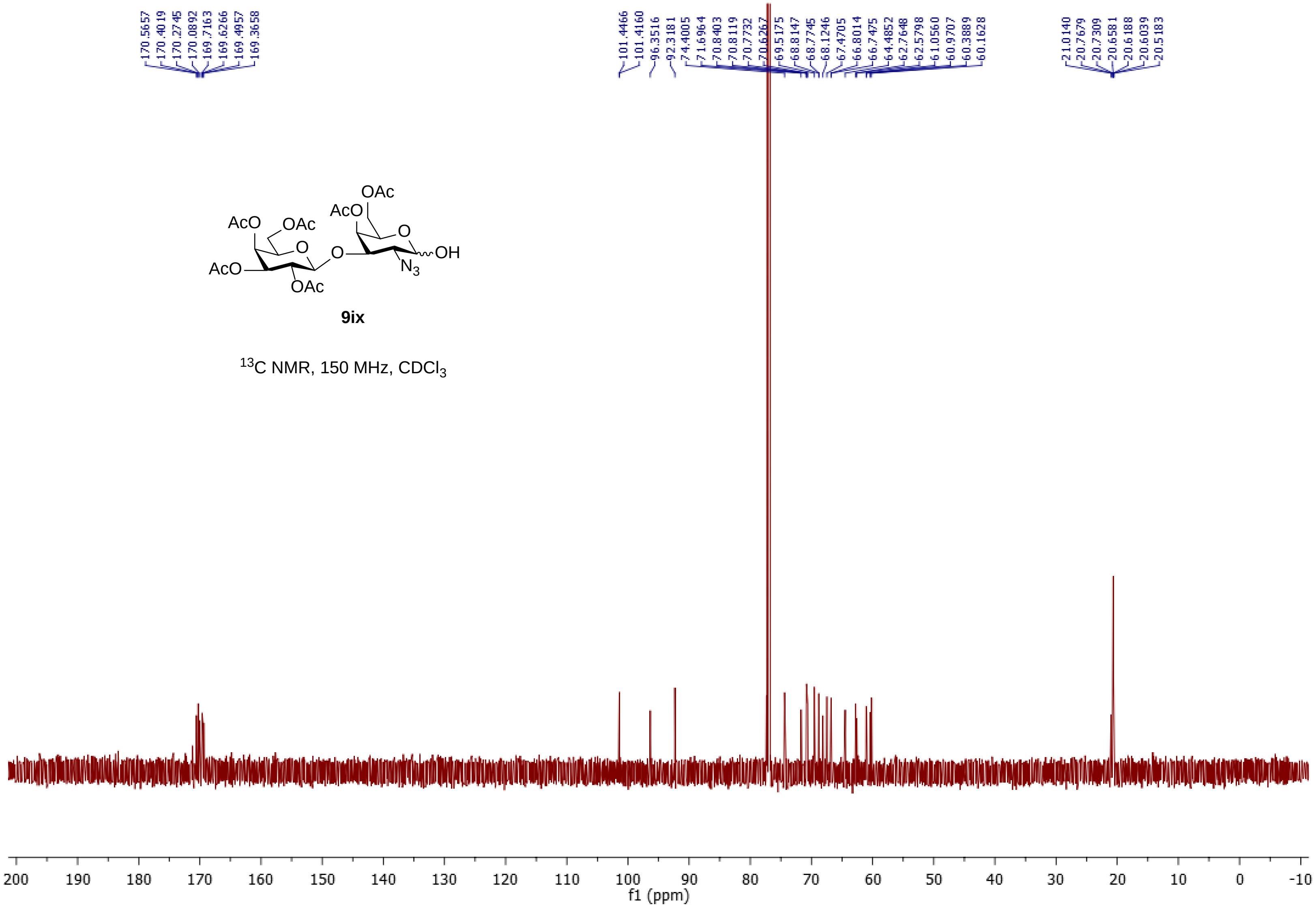

170.5657
170.4019
170.2745
170.0892
169.7163
169.6266
169.4957
169.3658
101.4466
101.4160
96.3516
92.3181
74.4005
71.6964
70.8403
70.8119
70.7732
70.6267
69.5175
68.8147
68.7745
68.1246
67.4705
66.8014
66.7475
64.4852
62.7648
62.5798
61.0560
60.9707
60.3889
60.1628
21.0140
20.7679
20.7309
20.6581
20.6188
20.6039
20.5183
AcO
OAc
AcO
OAc
O
O
O
OH
AcO
N3
OAc
9ix
13C NMR, 150 MHz, CDCl3
200
190
180
170
160
150
140
130
120
110
100
90
80
70
60
50
40
30
20
10
0
-10
f1 (ppm)

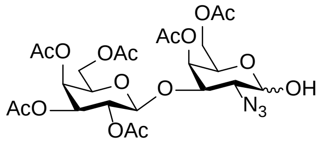

9ix

^{1}H-^{13}C HSQC, 600/150MHz, $CDCl_3$

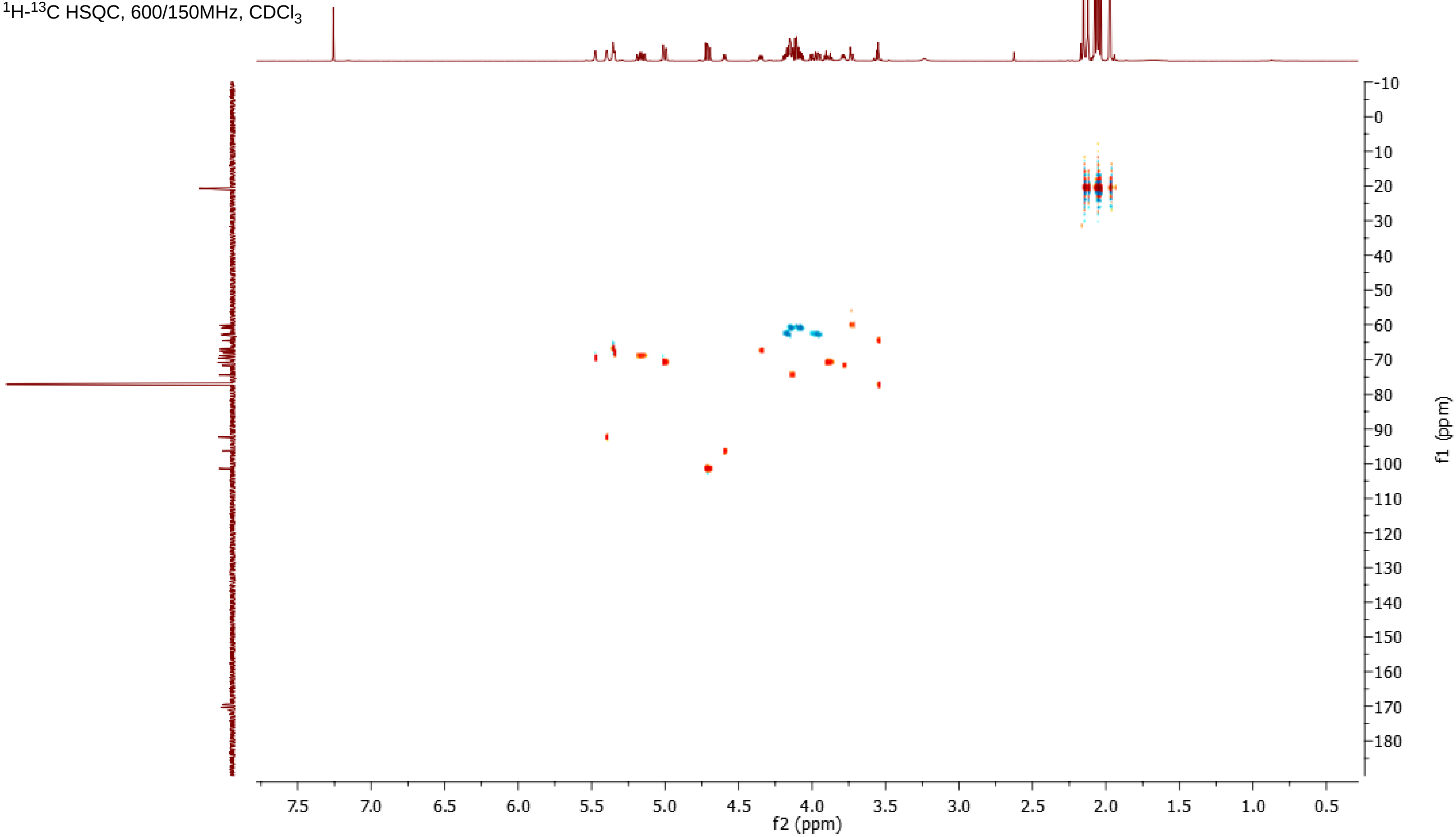

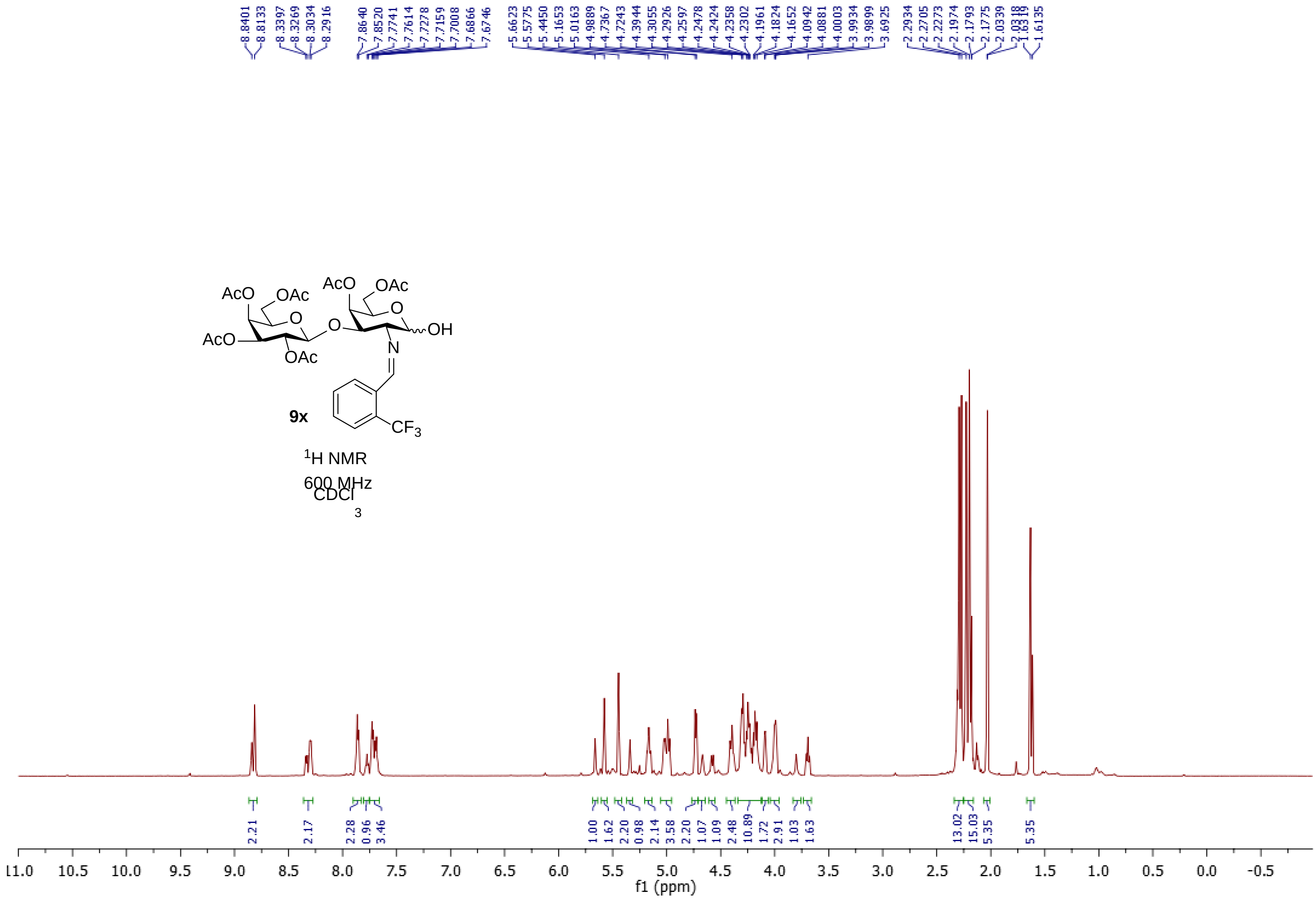
AcO
OAc
AcO
OAc
O
O
O
AcO
OAc
OH
N
9x
CF3
1H NMR
600 MHz
CDCl3
f1 (ppm)

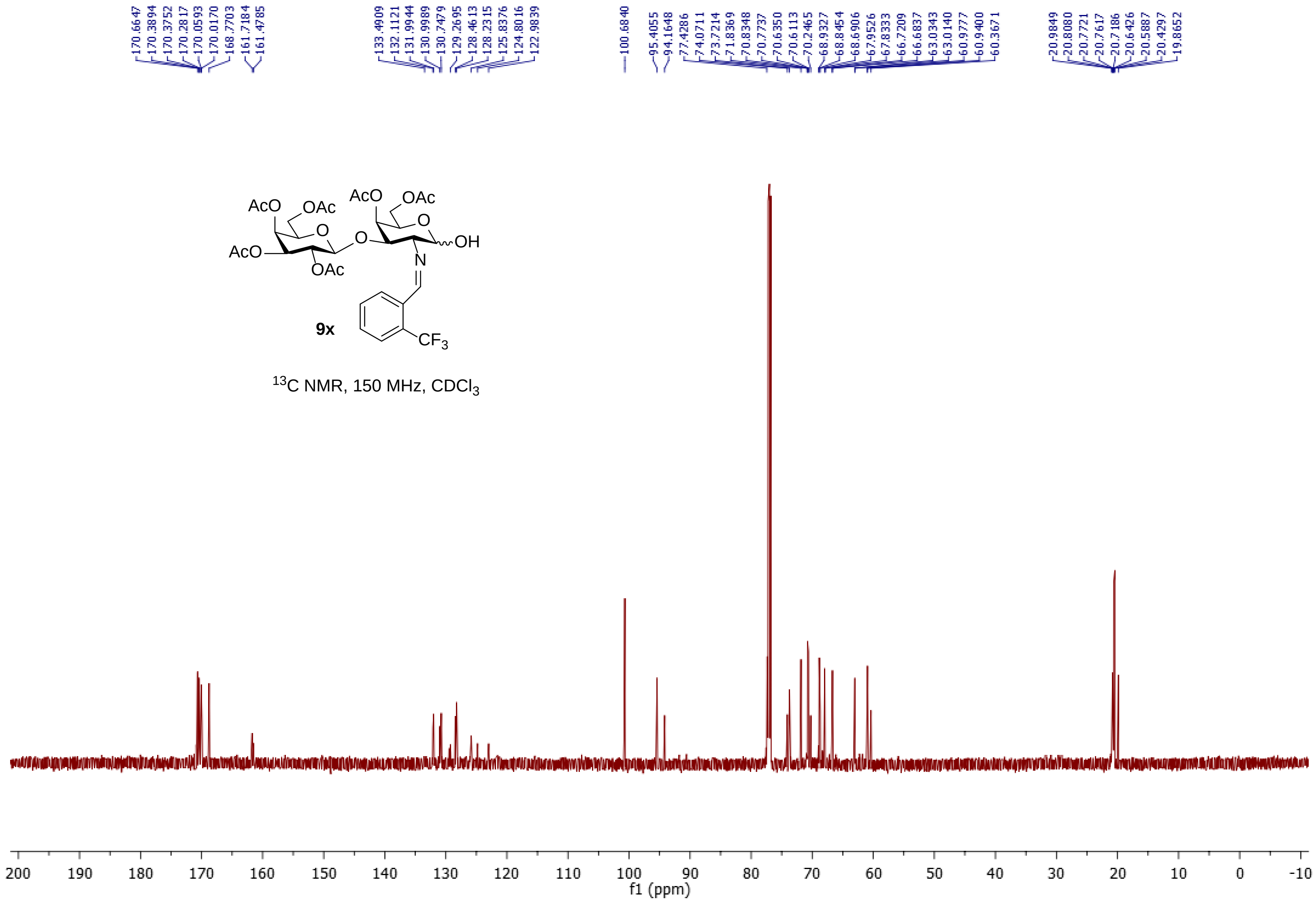

170.6647
170.3894
170.3752
170.2817
170.0593
170.0170
168.7703
161.7184
161.4785
133.4909
132.1121
131.9944
130.9989
130.7479
129.2695
128.4613
128.2315
125.8376
124.8016
122.9839
100.6840
95.4055
94.1648
77.4286
74.0711
73.7214
71.8369
70.8348
70.7737
70.6350
70.6113
70.2465
68.9327
68.8454
68.6906
67.9526
67.8333
66.7209
66.6837
63.0343
63.0140
60.9777
60.9400
60.3671
20.9849
20.8080
20.7721
20.7617
20.7186
20.6426
20.5887
20.4297
19.8652
AcO
OAc
O
AcO
OAc
O
O
OAc
AcO
OH
N
CF3
9x
13C NMR, 150 MHz, CDCl3
200 190 180 170 160 150 140 130 120 110 100 90 80 70 60 50 40 30 20 10 0 -10
f1 (ppm)

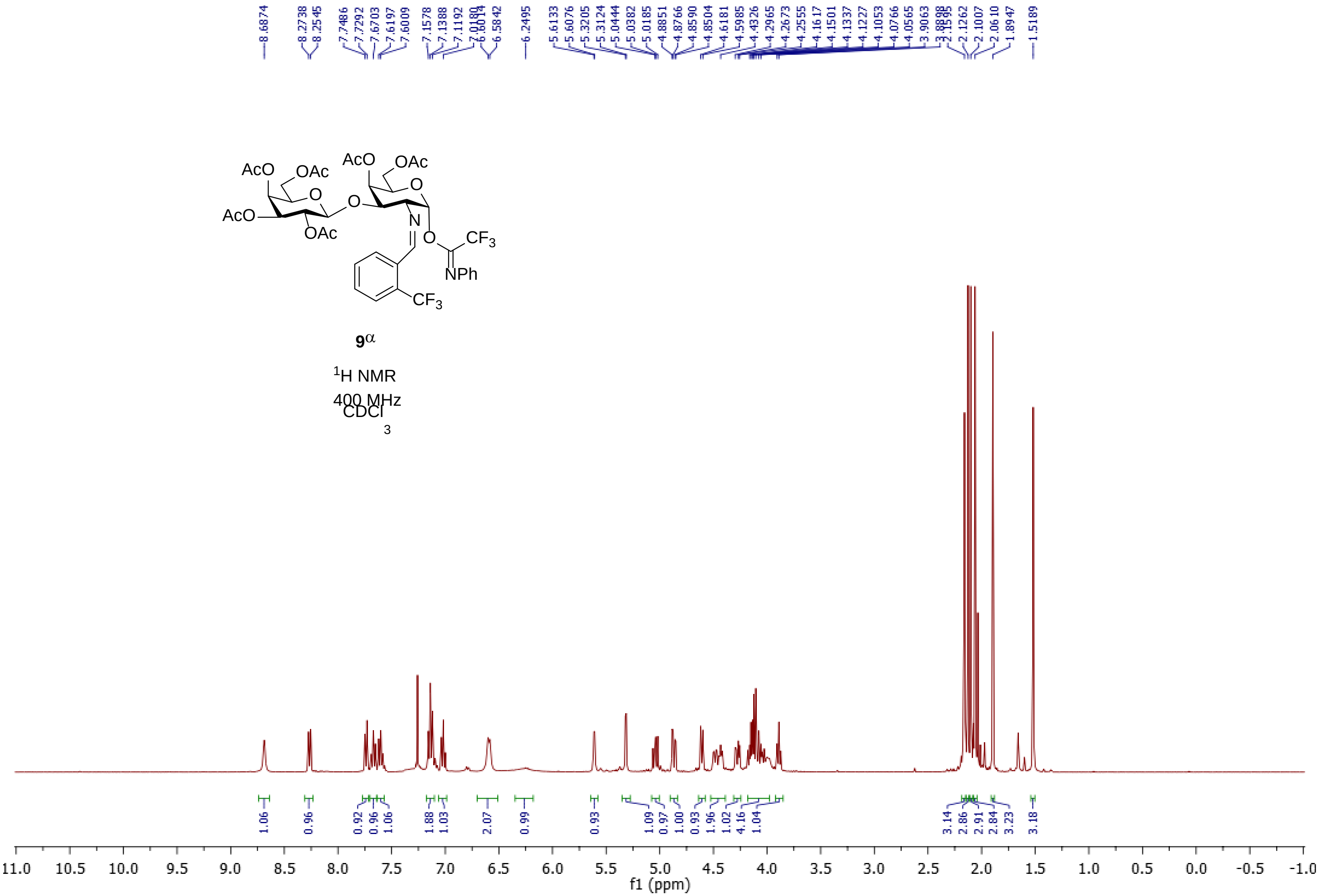
AcO
OAc
AcO
OAc
AcO
OAc
CF3
NPh
CF3
9α
1H NMR
400 MHz
CDCl3
f1 (ppm)

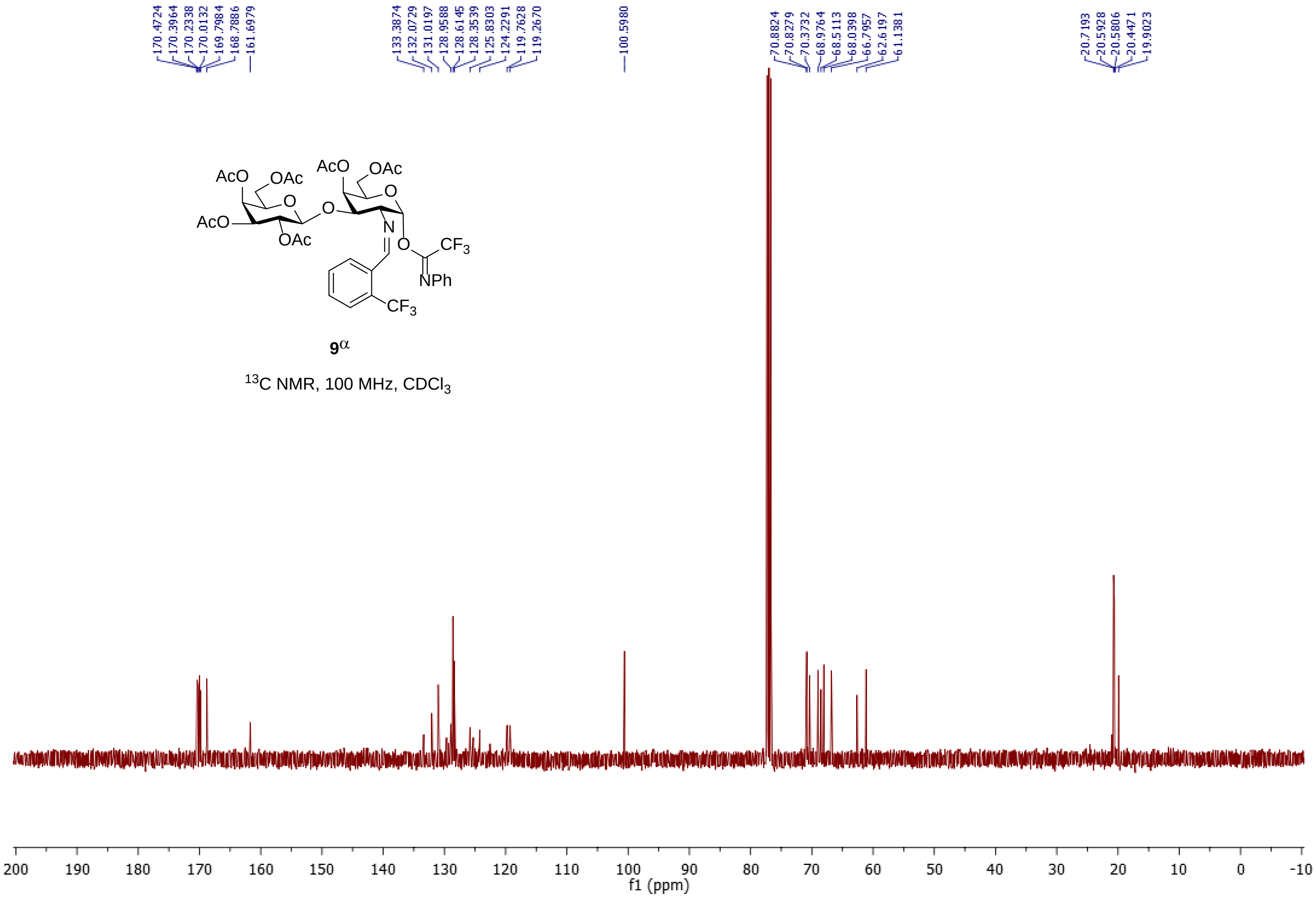

170.4724
170.3964
170.2338
170.0132
169.7984
168.7886
161.6979
133.3874
132.0729
131.0197
128.9588
128.6145
128.3539
125.8303
124.2291
119.7628
119.2670
100.5980
70.8824
70.8279
70.3732
68.9764
68.5113
68.0398
66.7957
62.6197
61.1381
20.7193
20.5928
20.5806
20.4471
19.9023
AcO
OAc
O
AcO
OAc
O
AcO
OAc
O
O
N
O
CF3
NPh
CF3
9α
13C NMR, 100 MHz, CDCl3
200
190
180
170
160
150
140
130
120
110
100
90
80
70
60
50
40
30
20
10
0
-10
f1 (ppm)

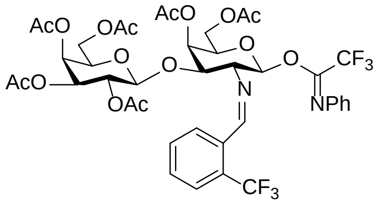

9β

^{1}H NMR
400 MHz
$CDCl_3$

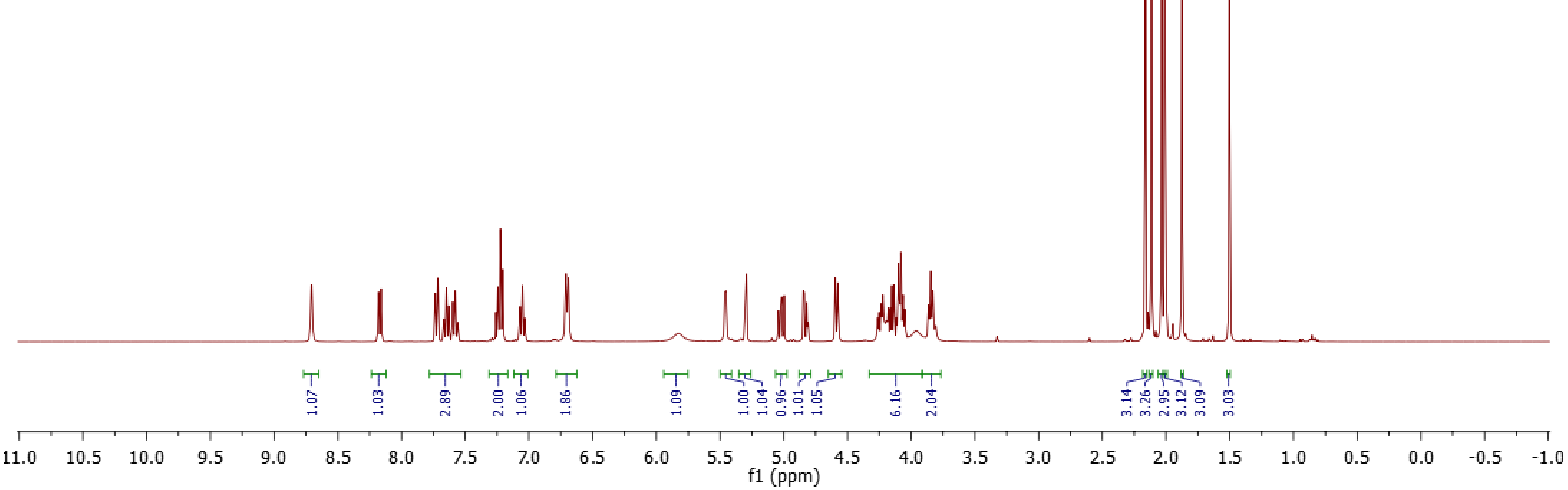

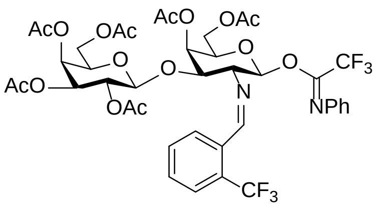

9β

^{13}C NMR, 100 MHz, $CDCl_3$

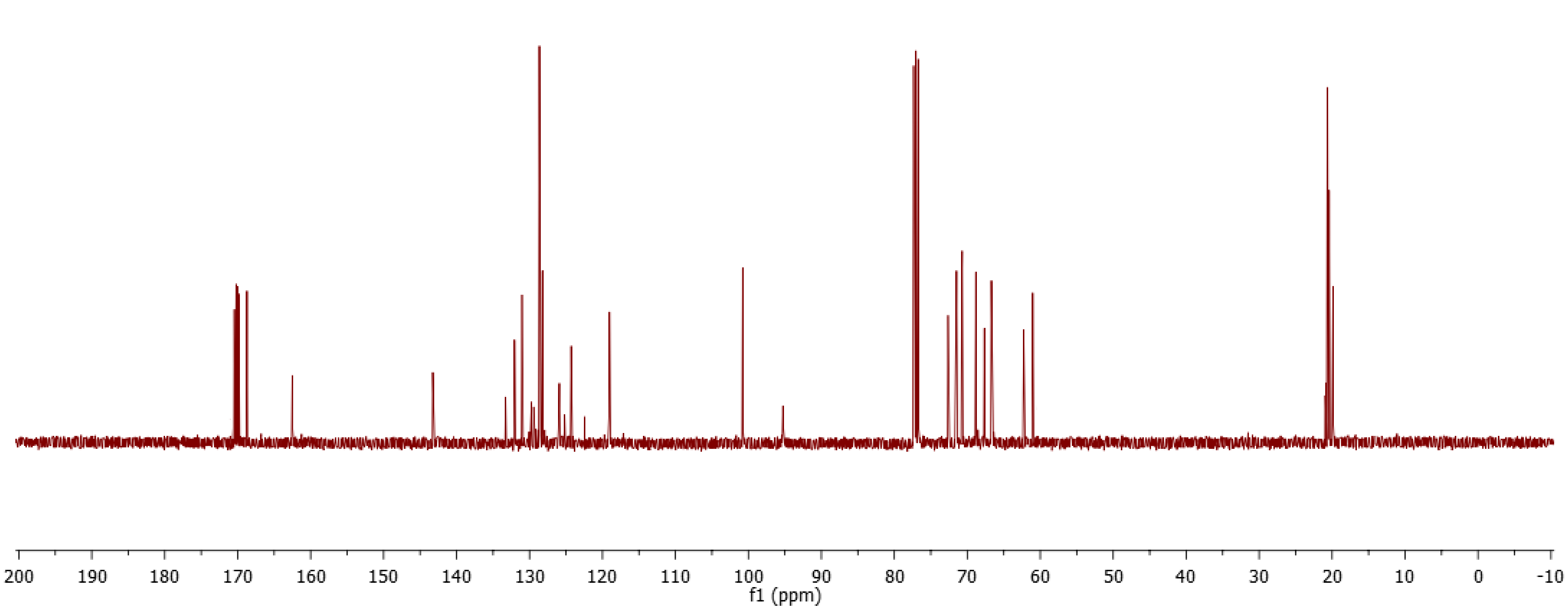

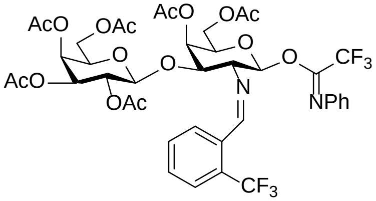

9β

^{1}H-^{13}C HSQC, 400/100MHz, $CDCl_3$

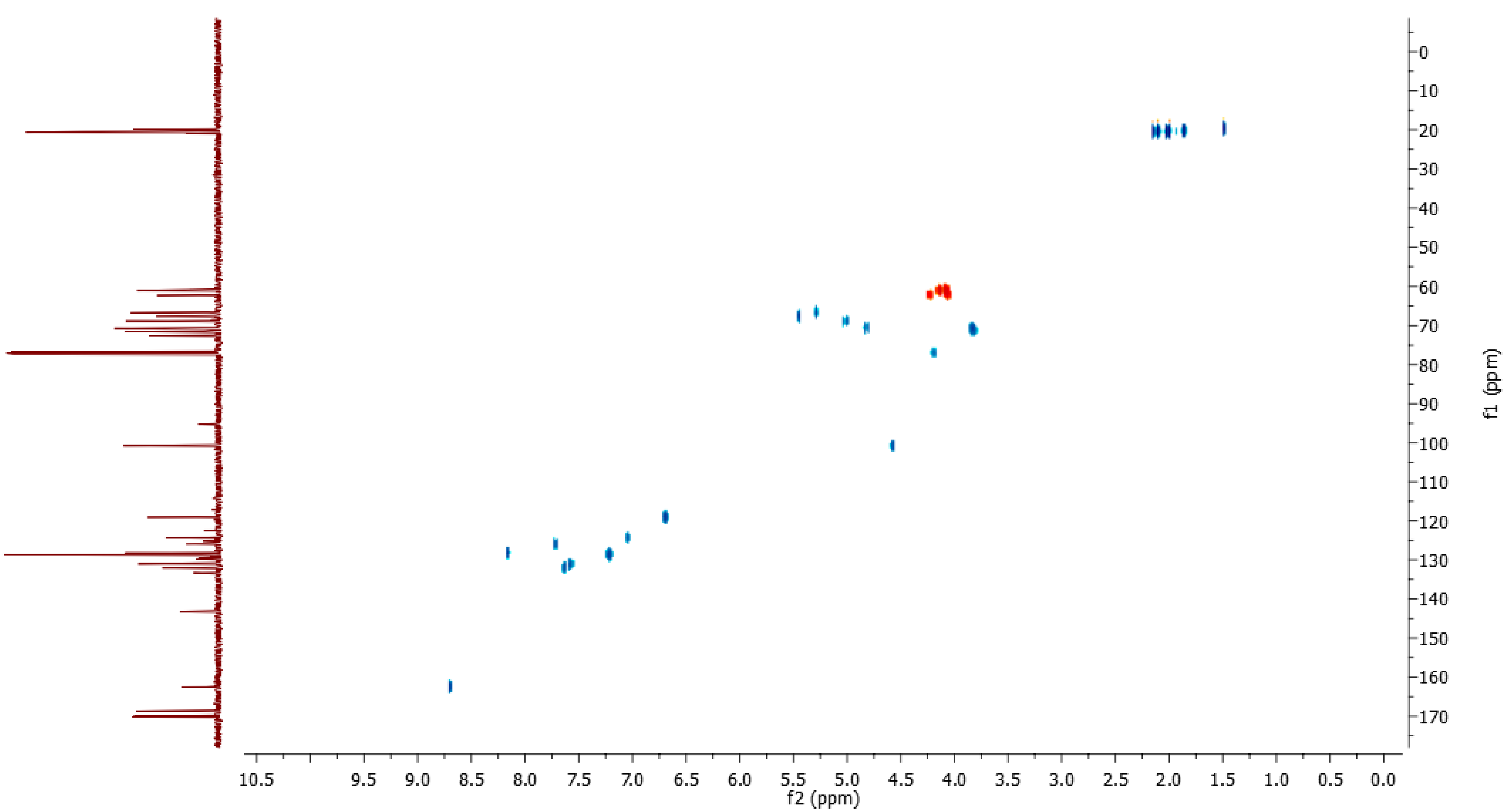

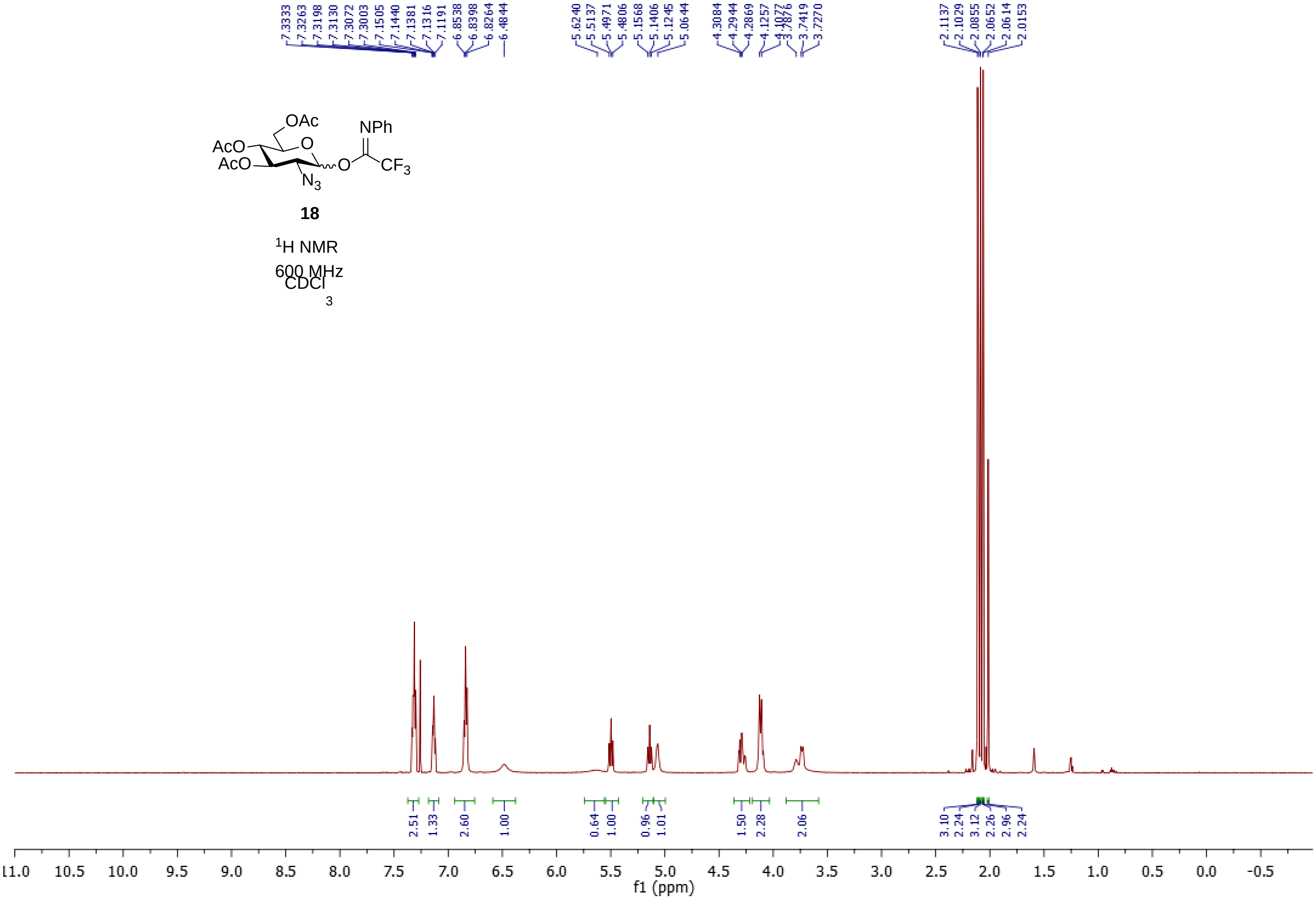

18

^{1}H NMR
600 MHz
$CDCl_3$

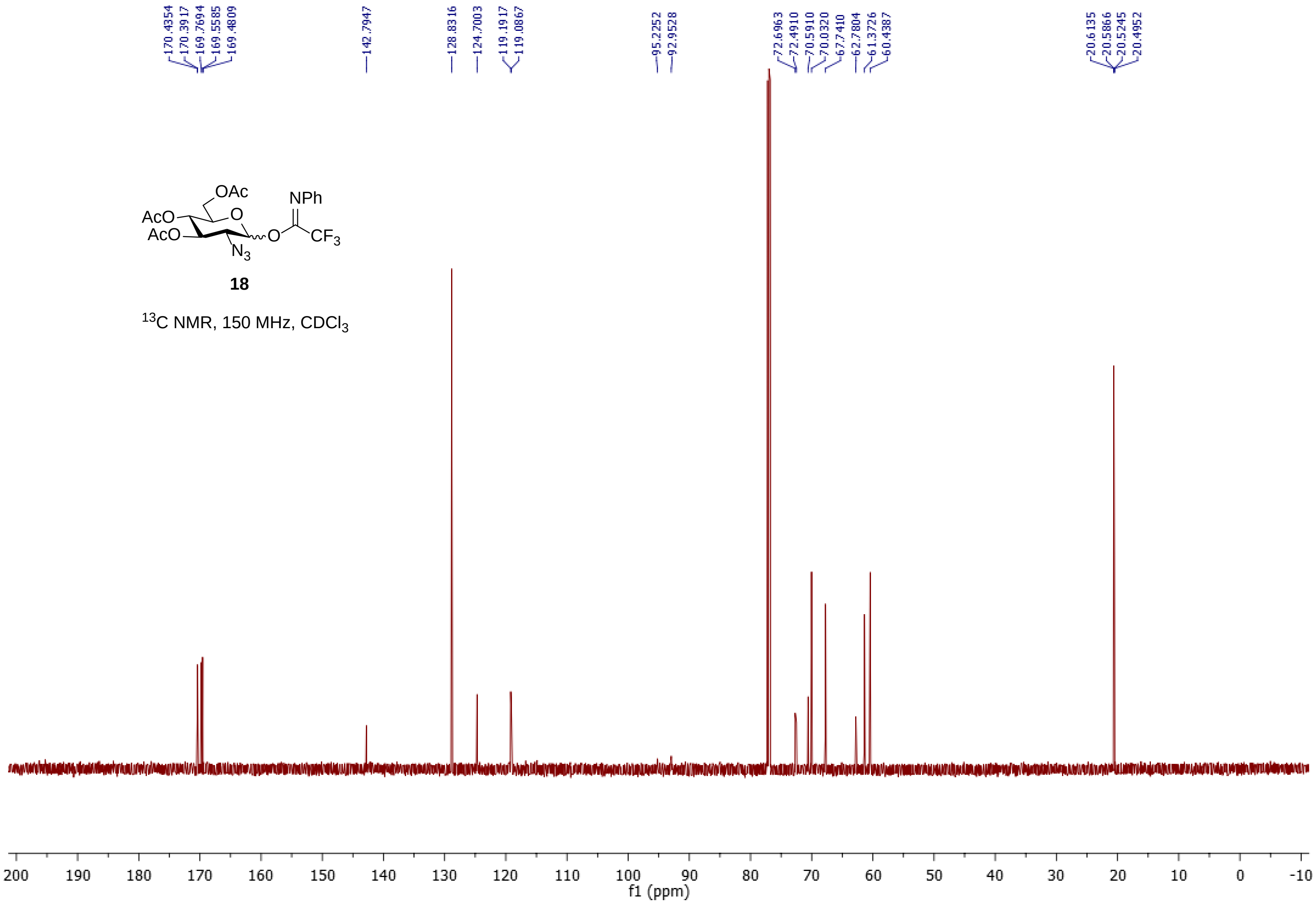

OAc
AcO
AcO
O
N3
O
NPh
CF3
18
13C NMR, 150 MHz, CDCl3
170.4354
170.3917
169.7694
169.5585
169.4809
142.7947
128.8316
124.7003
119.1917
119.0867
95.2252
92.9528
72.6963
72.4910
70.5910
70.0320
67.7410
62.7804
61.3726
60.4387
20.6135
20.5866
20.5245
20.4952
200
190
180
170
160
150
140
130
120
110
100
90
80
70
60
50
40
30
20
10
0
-10
f1 (ppm)

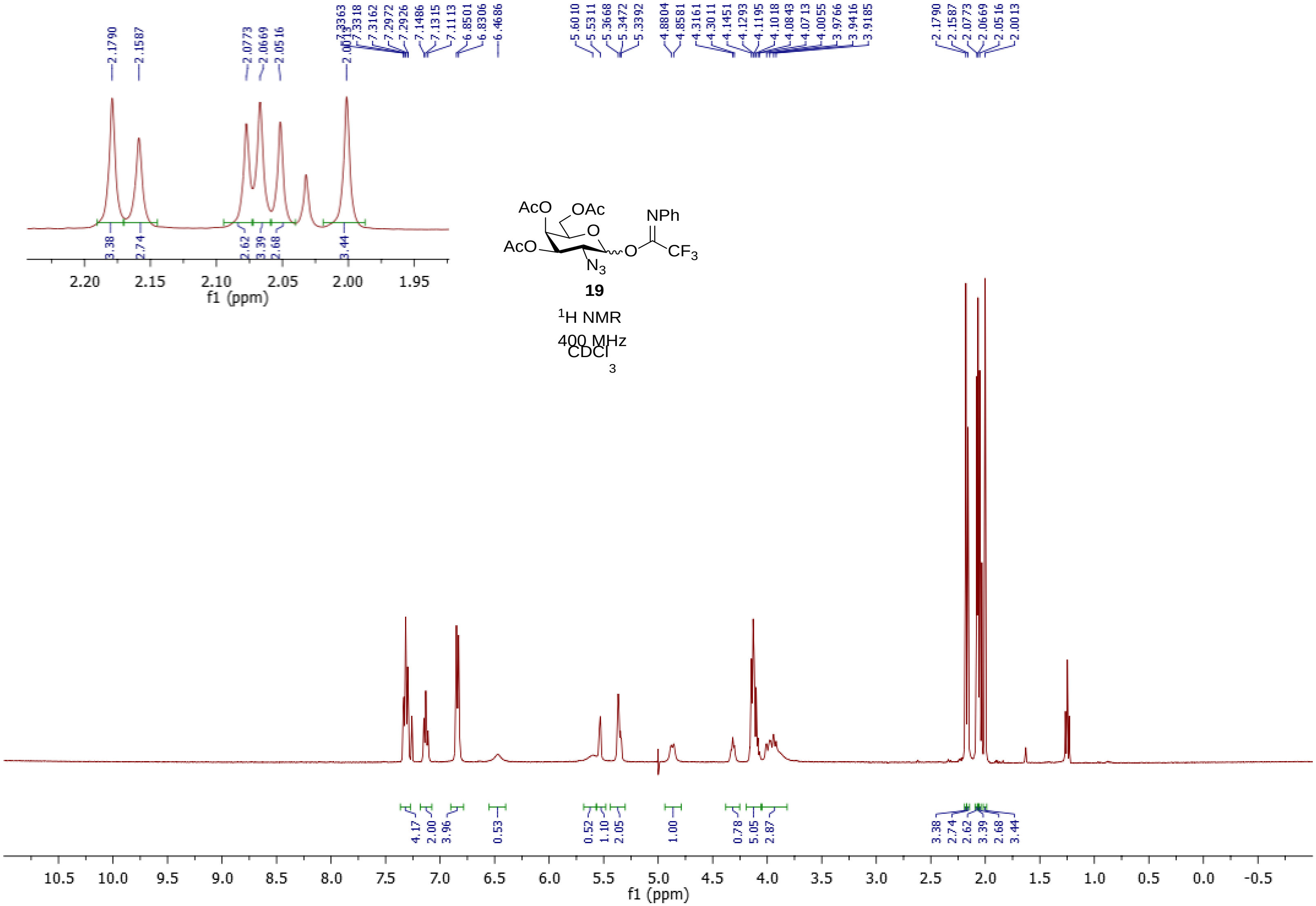

19

^{1}H NMR

400 MHz

$CDCl_3$

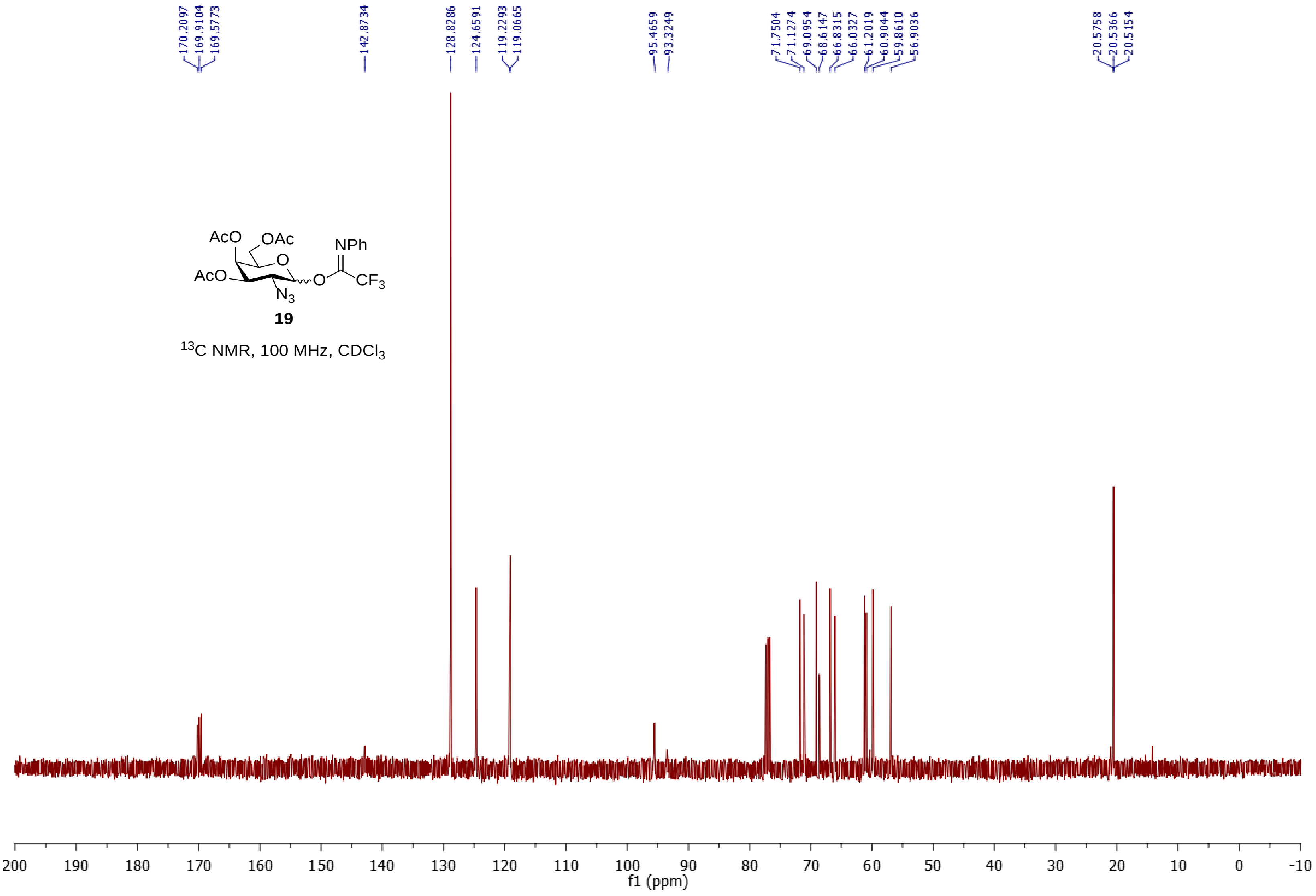
170.2097
169.9104
169.5773
142.8734
128.8286
124.6591
119.2293
119.0665
95.4659
93.3249
71.7504
71.1274
69.0954
68.6147
66.8315
66.0327
61.2019
60.9044
59.8610
56.9036
20.5758
20.5366
20.5154
AcO
OAc
O
NPh
AcO
O
CF3
N3
19
13C NMR, 100 MHz, CDCl3
200 190 180 170 160 150 140 130 120 110 100 90 80 70 60 50 40 30 20 10 0 -10
f1 (ppm)

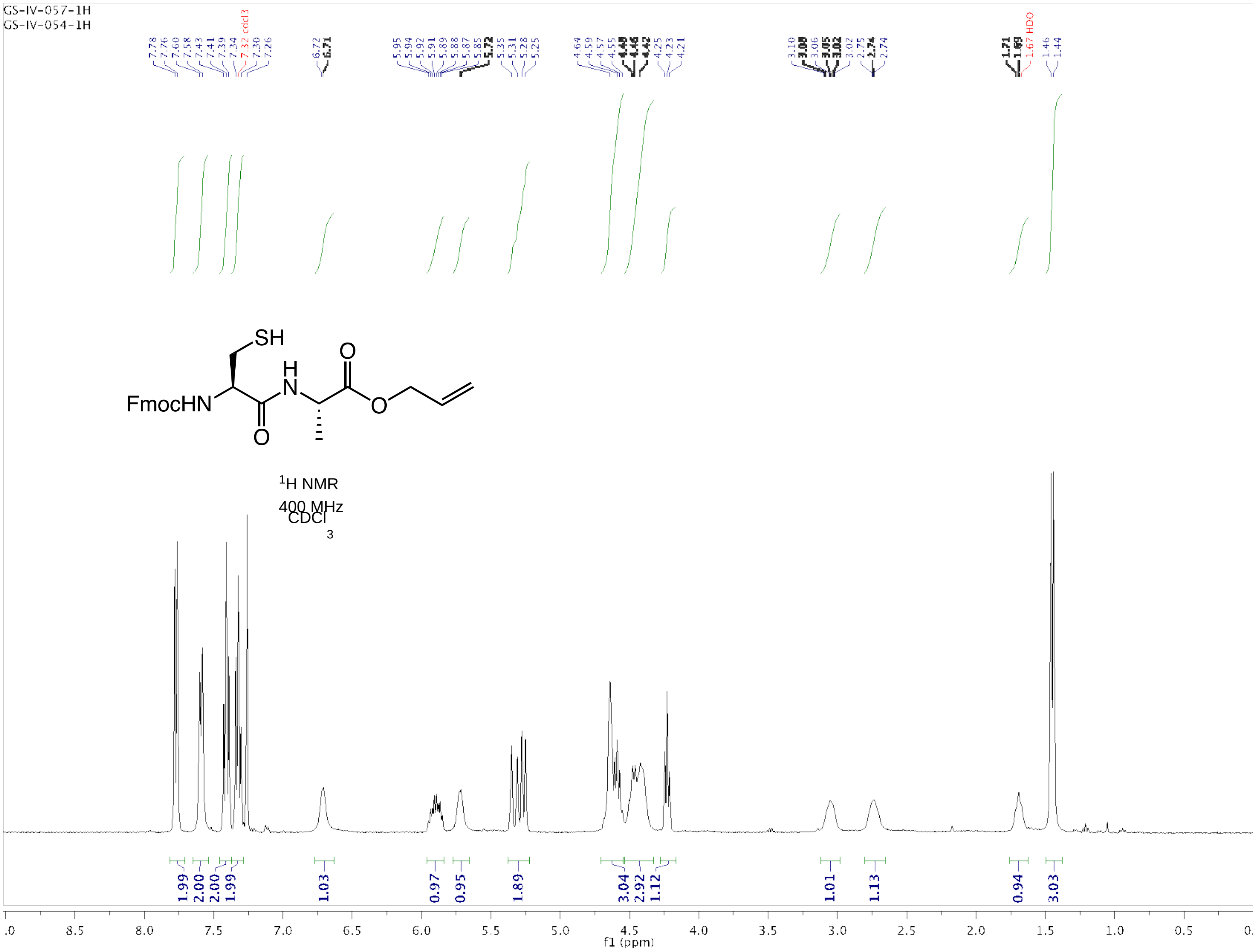
GS-IV-057-1H
GS-IV-054-1H
FmocHN
SH
H
N
O
O
O
1H NMR
400 MHz
CDCl3
f1 (ppm)
1.99
2.00
2.00
1.99
1.03
0.97
0.95
1.89
3.04
2.92
1.12
1.01
1.13
0.94
3.03

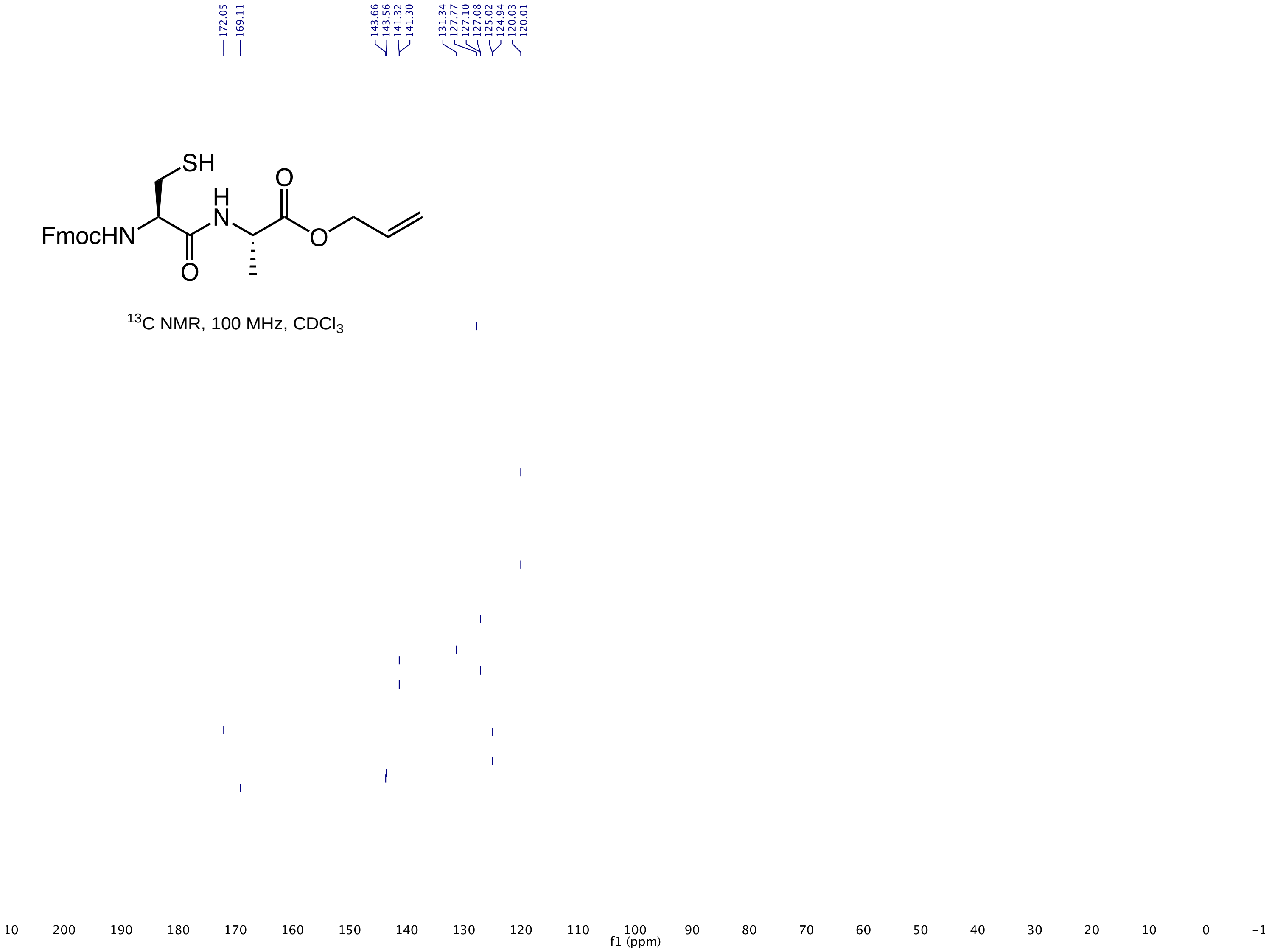

172.05
169.11
143.66
143.56
141.32
141.30
131.34
127.77
127.10
127.08
125.02
124.94
120.03
120.01
SH
H
N
O
FmocHN
O
O
^{13}C NMR, 100 MHz, $CDCl_3$
10
200
190
180
170
160
150
140
130
120
110
100
90
80
70
60
50
40
30
20
10
0
-1
f1 (ppm)

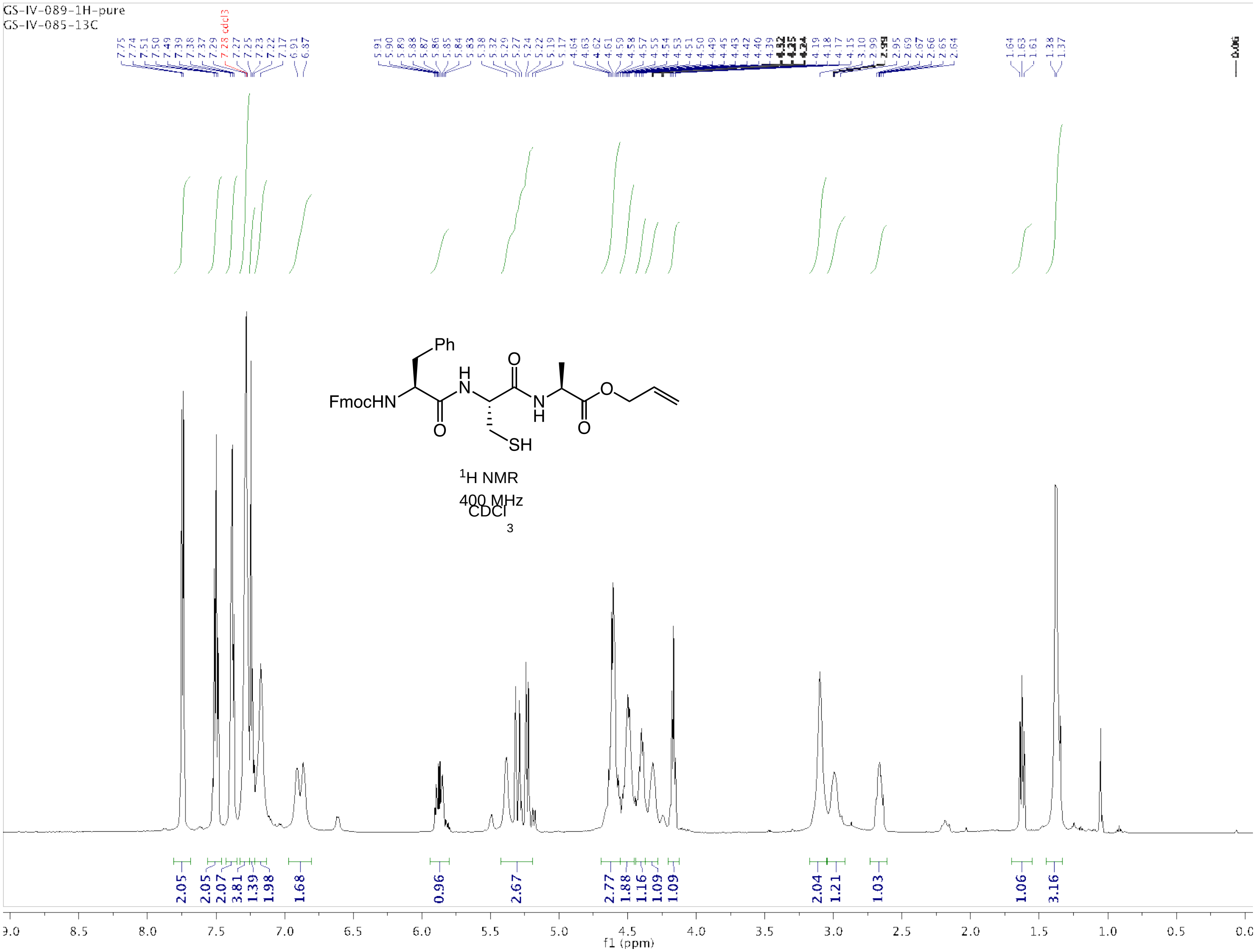

GS-IV-089-1H-pure
GS-IV-085-13C
FmocHN
Ph
SH
O
H
N
¹H NMR
400 MHz
CDCl3
f1 (ppm)

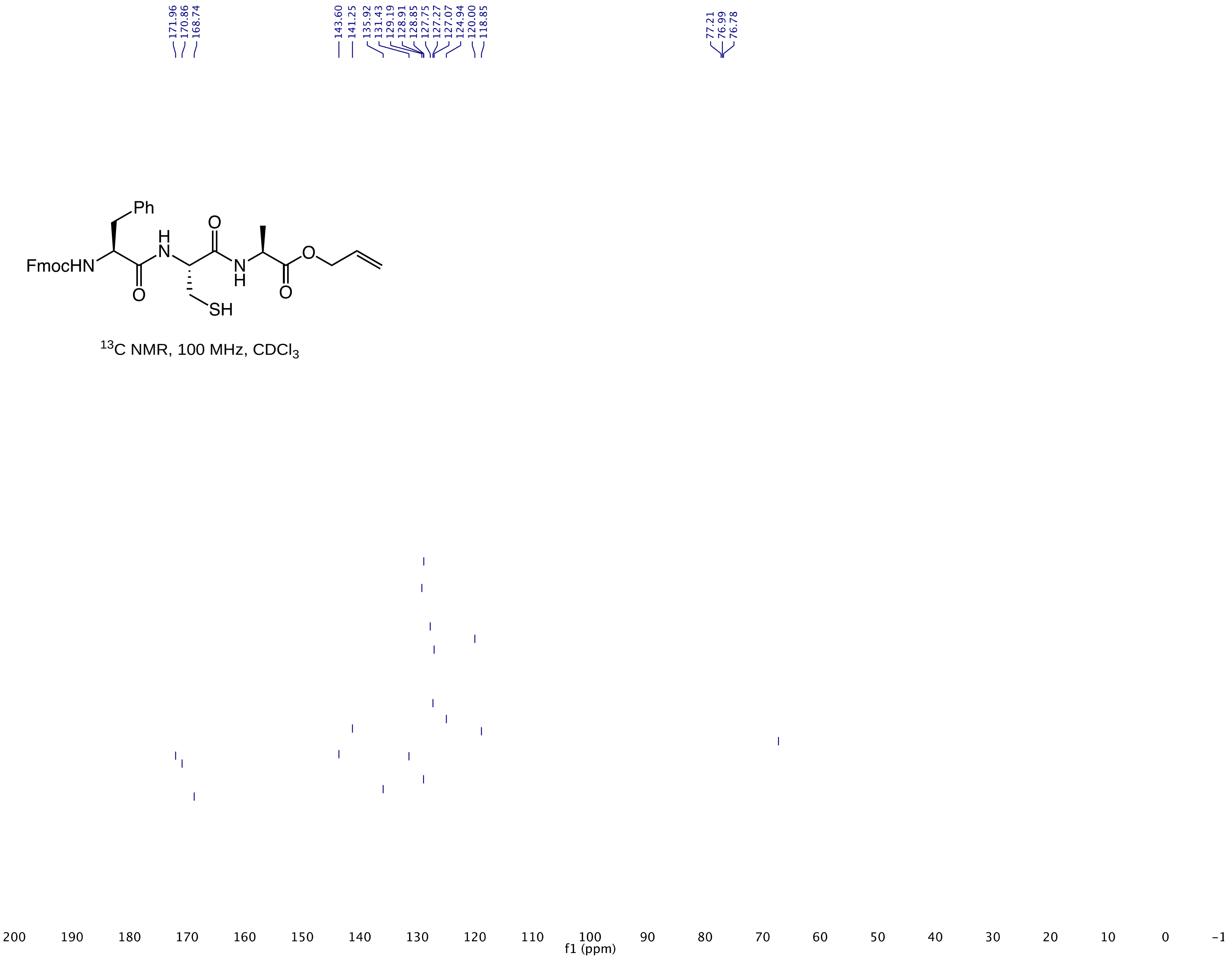
171.96
170.86
168.74
143.60
141.25
135.92
131.43
129.19
128.91
128.85
127.75
127.27
127.07
124.94
120.00
118.85
77.21
76.99
76.78
Ph
H
N
O
FmocHN
O
N
H
O
O
SH
13C NMR, 100 MHz, CDCl3
200 190 180 170 160 150 140 130 120 110 100 90 80 70 60 50 40 30 20 10 0 -1
f1 (ppm)

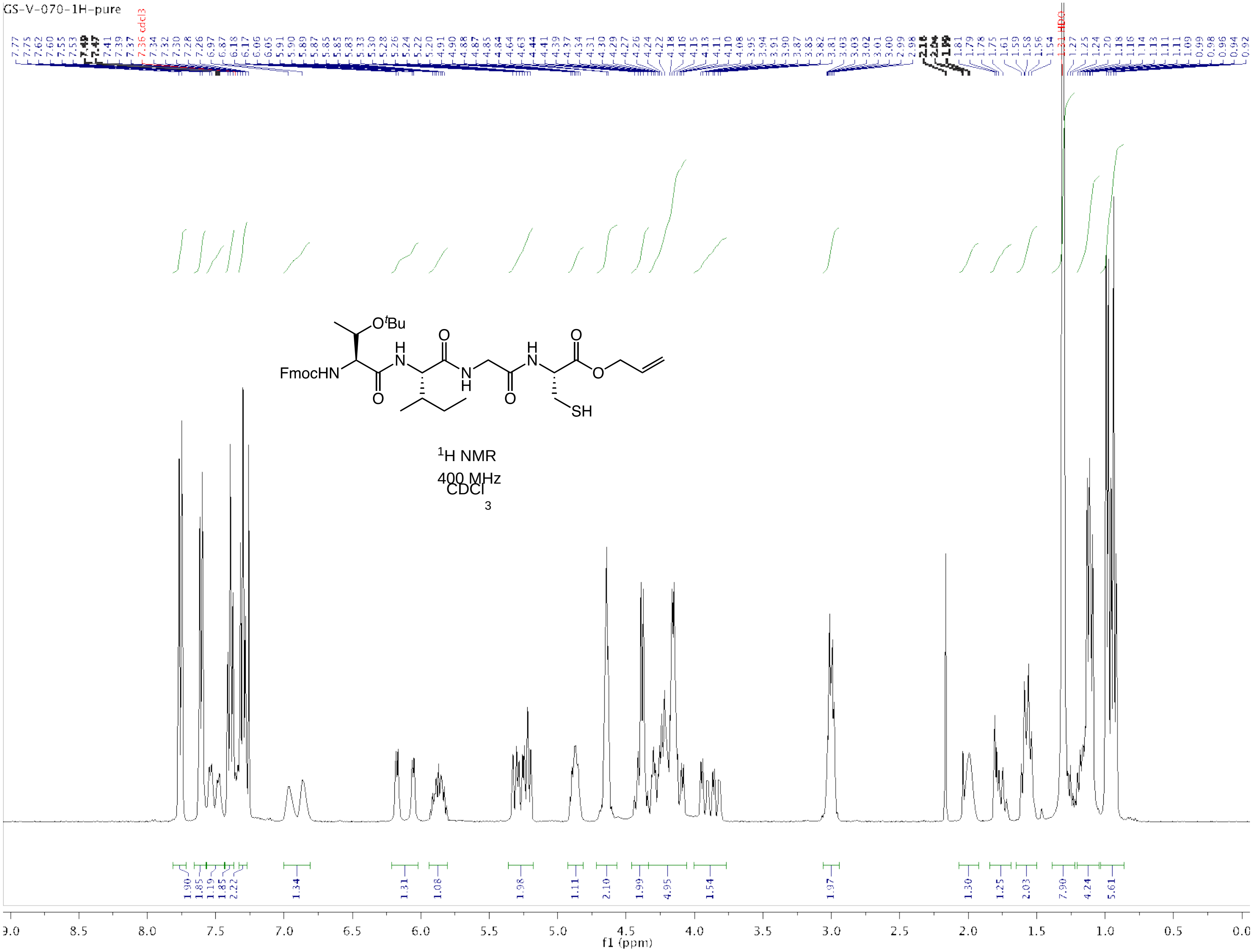

GS-V-070-1H-pure
O^{t}Bu
FmocHN
H
N
O
N
H
SH
O
^{1}H NMR
400 MHz
CDCl$_3$
1.90
1.85
1.19
1.85
2.22
1.34
1.31
1.08
1.98
1.11
2.10
1.99
4.95
1.54
1.97
1.30
1.25
2.03
7.90
4.24
5.61
9.0 8.5 8.0 7.5 7.0 6.5 6.0 5.5 5.0 4.5 4.0 3.5 3.0 2.5 2.0 1.5 1.0 0.5 0.0
f1 (ppm)

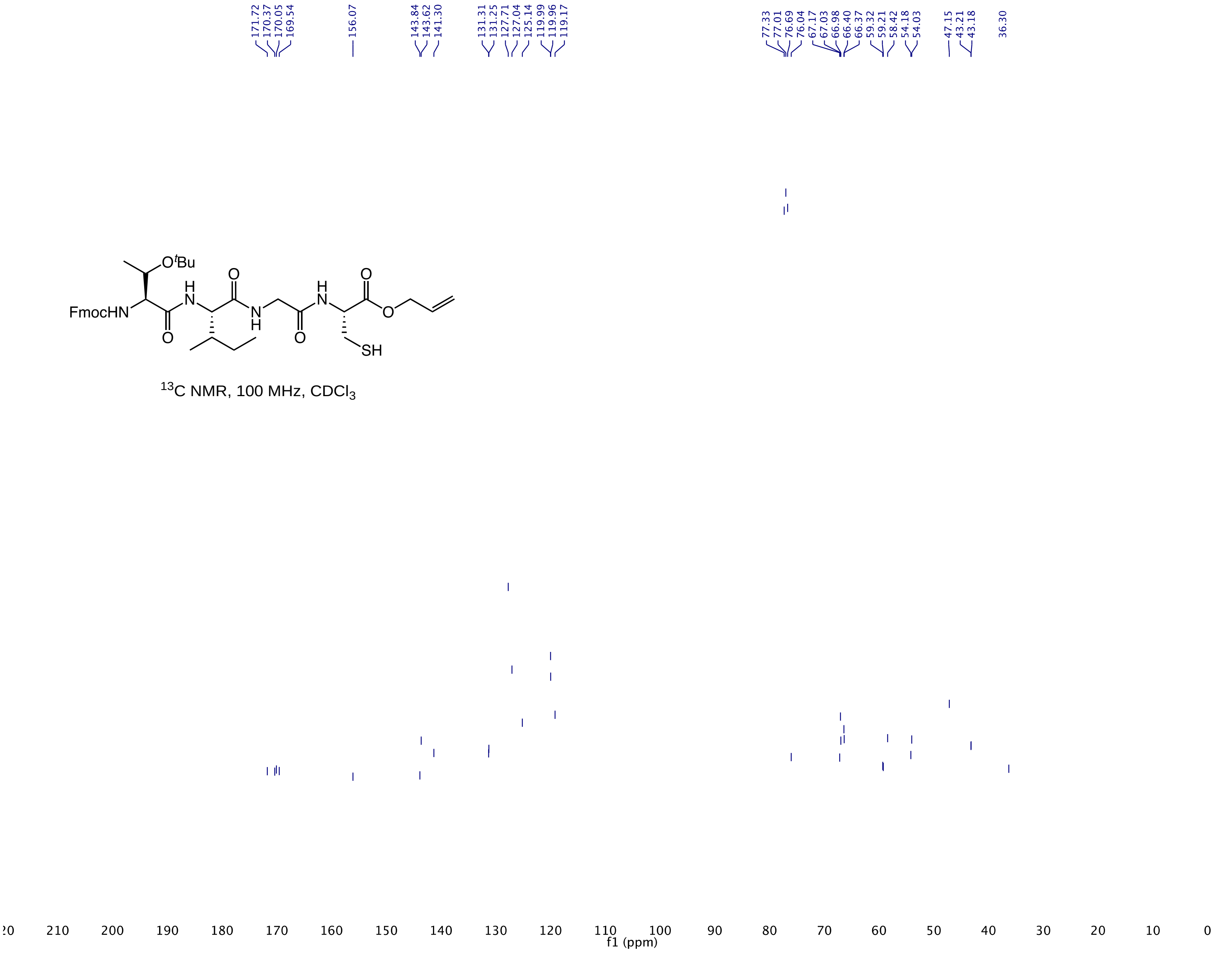

^{13}C NMR, 100 MHz, $CDCl_3$

8.22

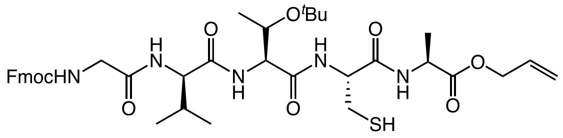

^{1}H NMR
400 MHz
$CDCl_3$

9.0 8.5 8.0 7.5 7.0 6.5 6.0 5.5 5.0 4.5 4.0 3.5 3.0 2.5 2.0 1.5 1.0 0.5 0.0
f1 (ppm)

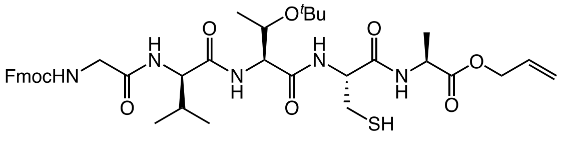

^{13}C NMR, 100 MHz, $CDCl_3$

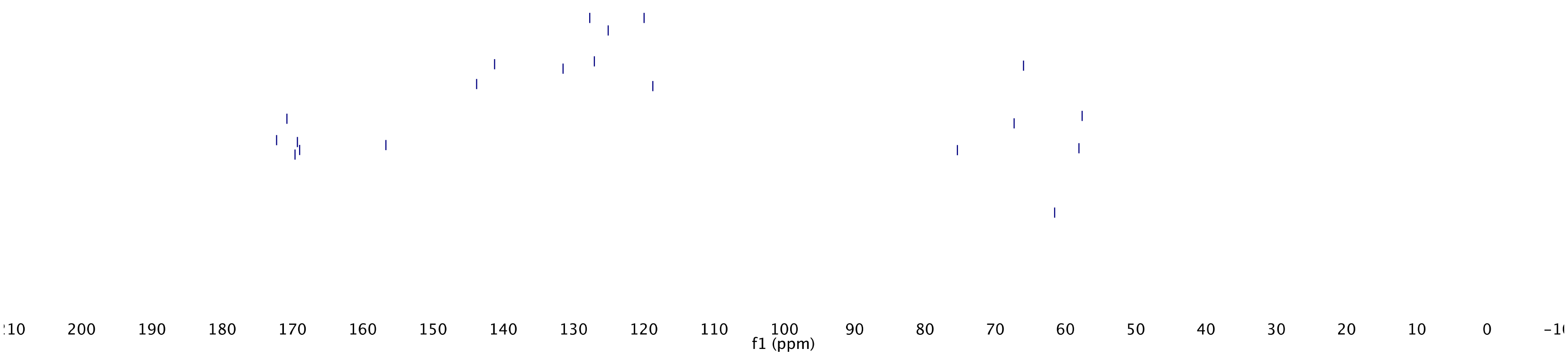

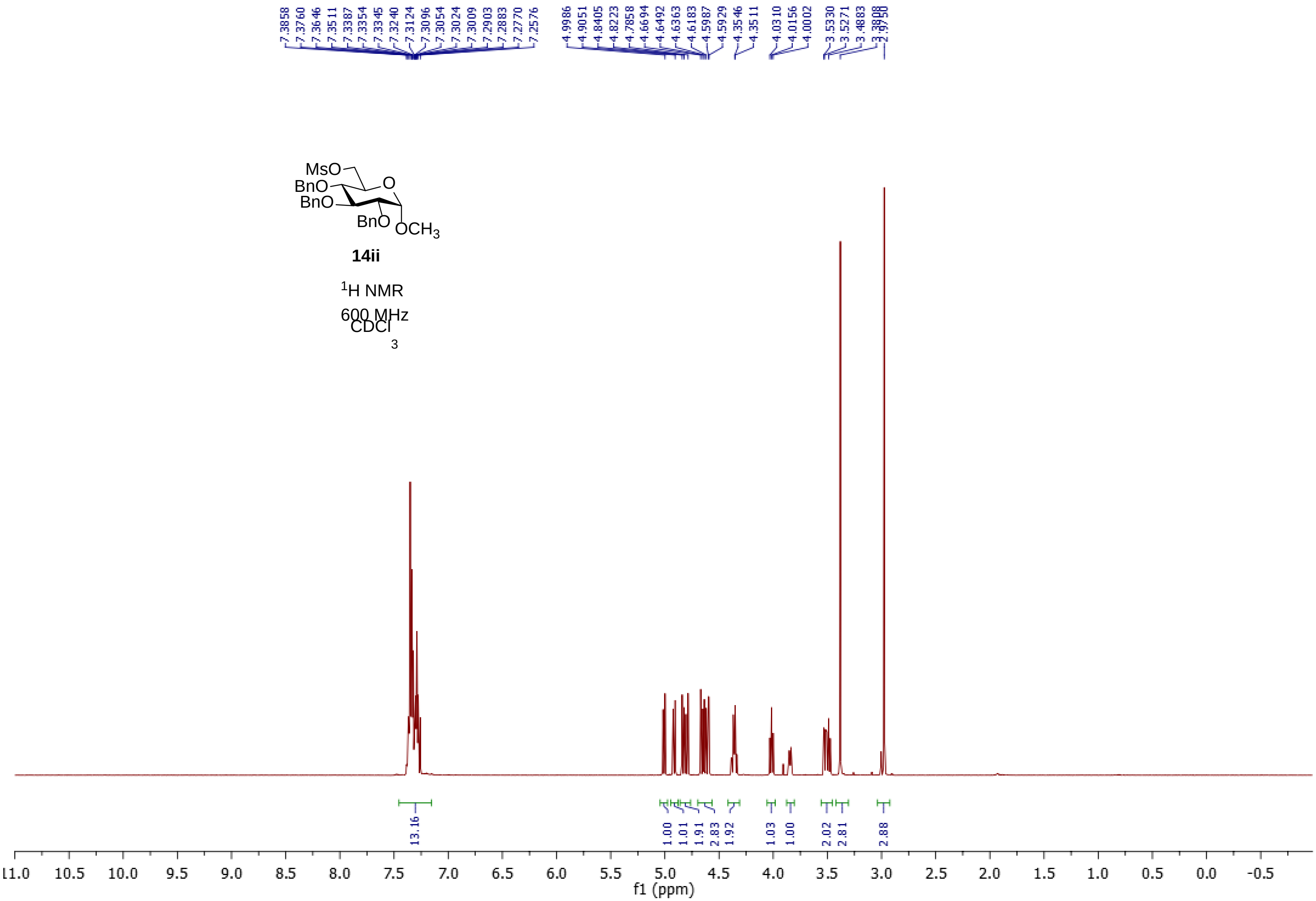
7.3858
7.3760
7.3646
7.3511
7.3387
7.3354
7.3345
7.3240
7.3124
7.3096
7.3054
7.3024
7.3009
7.2903
7.2883
7.2770
7.2576
4.9986
4.9051
4.8405
4.8223
4.7858
4.6694
4.6492
4.6363
4.6183
4.5987
4.5929
4.3546
4.3511
4.0310
4.0156
4.0002
3.5330
3.5271
3.4883
3.3808
2.9750
MsO
BnO
BnO
O
BnO
OCH3
14ii
1H NMR
600 MHz
CDCl3
13.16
1.00
1.01
1.91
2.83
1.92
1.03
1.00
2.02
2.81
2.88
11.0
10.5
10.0
9.5
9.0
8.5
8.0
7.5
7.0
6.5
6.0
5.5
5.0
4.5
4.0
3.5
3.0
2.5
2.0
1.5
1.0
0.5
0.0
-0.5
f1 (ppm)

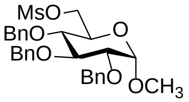

14ii

^{13}C NMR, 150 MHz, $CDCl_3$

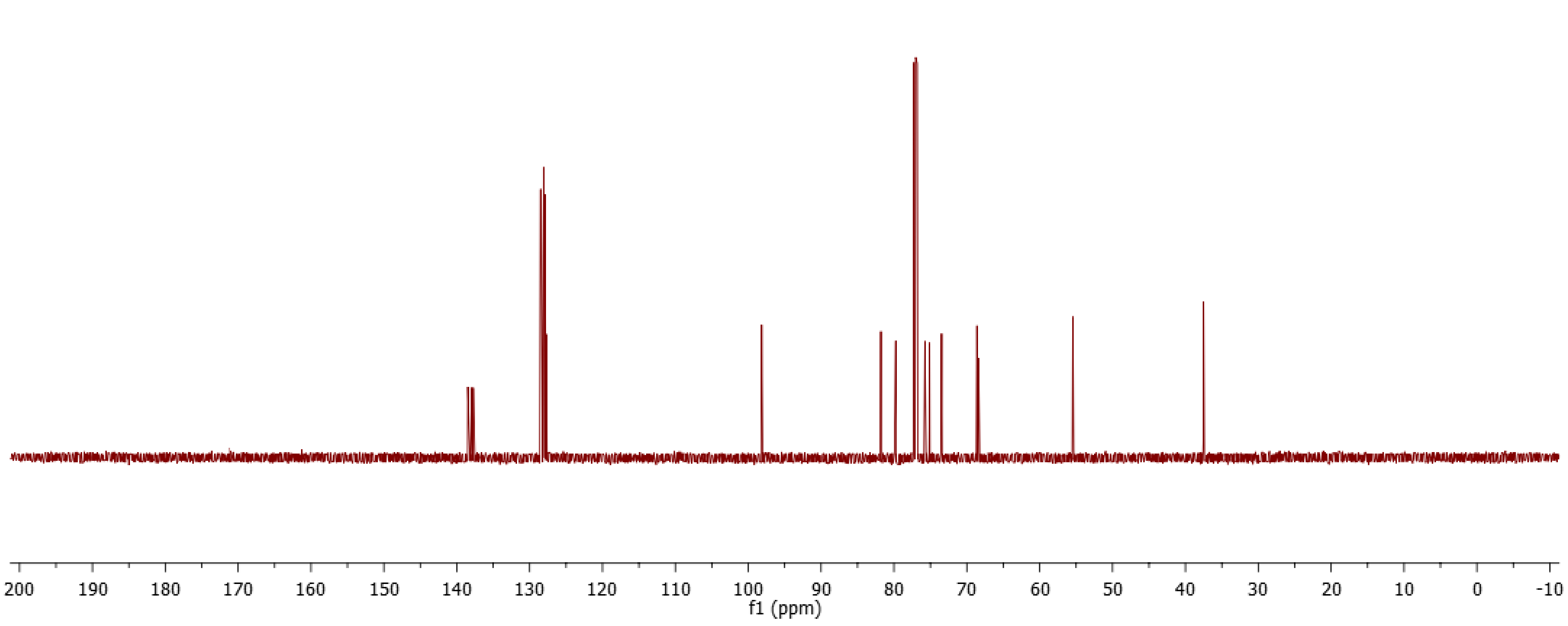

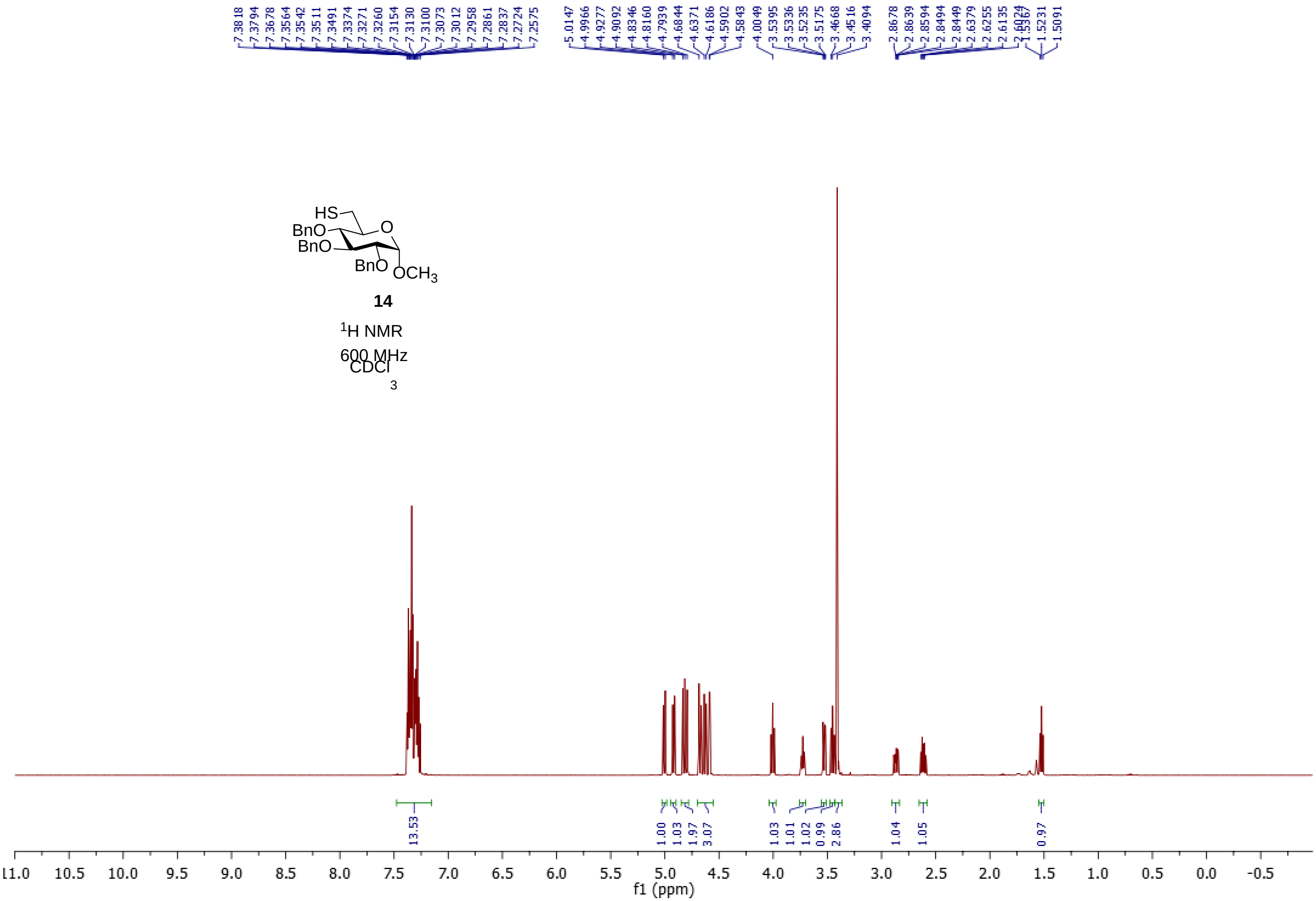

7.3818 7.3794 7.3678 7.3564 7.3542 7.3511 7.3491 7.3374 7.3271 7.3260 7.3154 7.3130 7.3100 7.3073 7.3012 7.2958 7.2861 7.2837 7.2724 7.2575
5.0147 4.9966 4.9277 4.9092 4.8346 4.8160 4.7939 4.6844 4.6371 4.6186 4.5902 4.5843 4.0049 3.5395 3.5336 3.5235 3.5175 3.4668 3.4516 3.4094
2.8678 2.8639 2.8594 2.8494 2.8449 2.6379 2.6255 2.6135 2.6024 1.5367 1.5231 1.5091
HS
BnO
BnO
BnO
O
OCH3
14
1H NMR
600 MHz
CDCl3
13.53
1.00 1.03 1.97 3.07
1.03 1.01 1.02 0.99 2.86
1.04 1.05 0.97
11.0 10.5 10.0 9.5 9.0 8.5 8.0 7.5 7.0 6.5 6.0 5.5 5.0 4.5 4.0 3.5 3.0 2.5 2.0 1.5 1.0 0.5 0.0 -0.5
f1 (ppm)

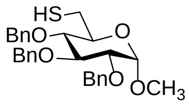

14

^{13}C NMR, 150 MHz, $CDCl_3$

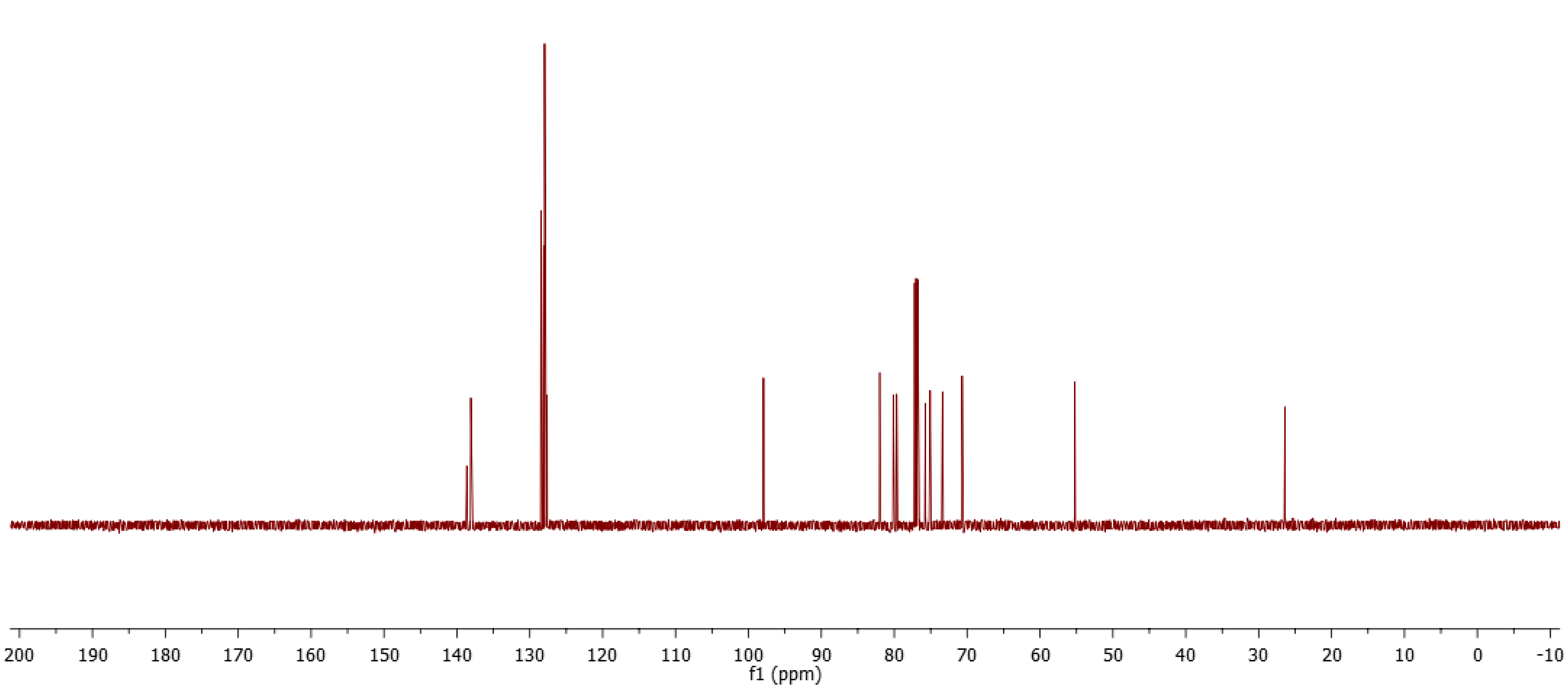

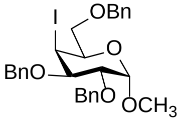

15ii

^{1}H NMR
400 MHz
$CDCl_3$

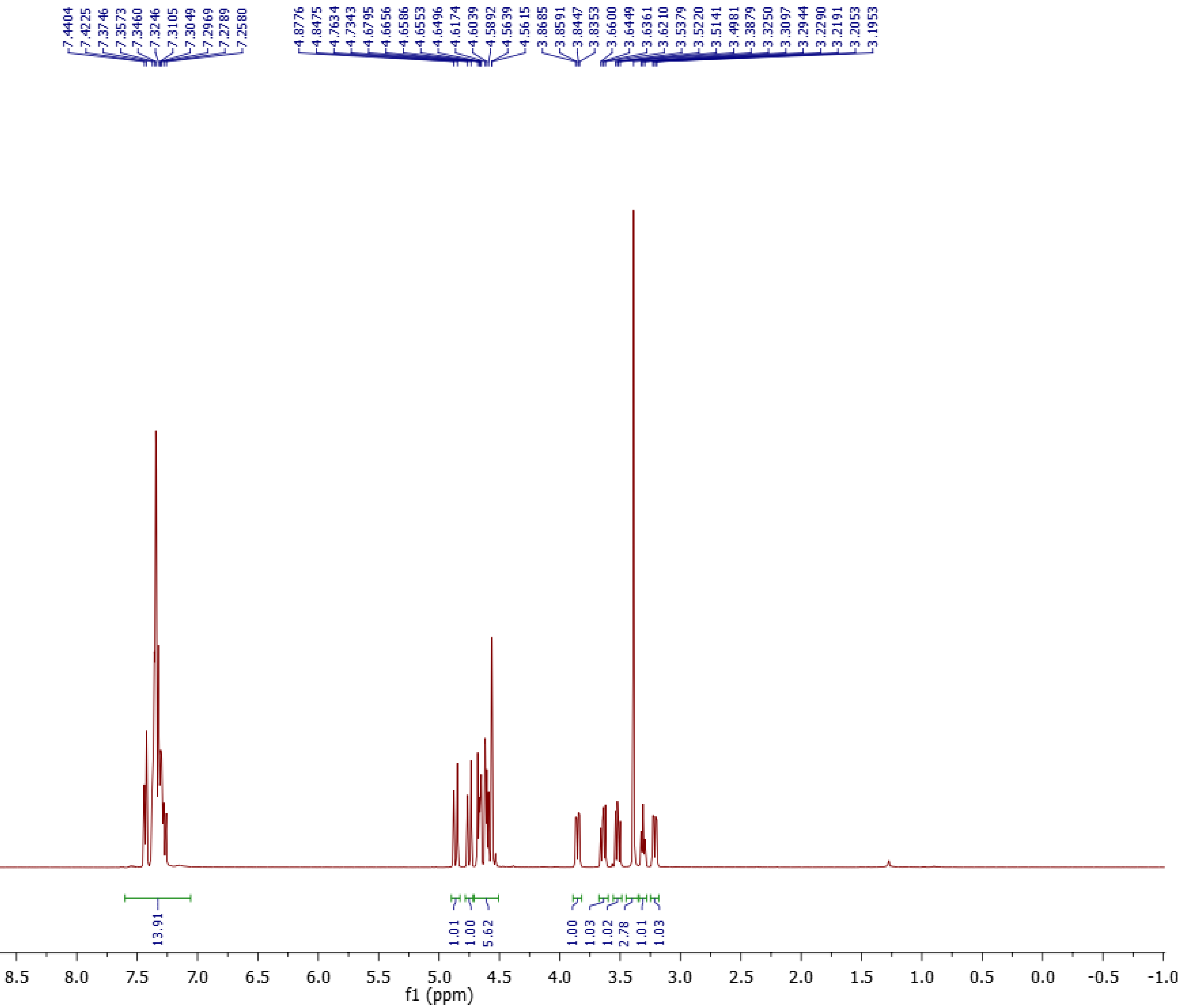

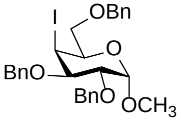

15ii

^{13}C NMR, 100 MHz, $CDCl_3$

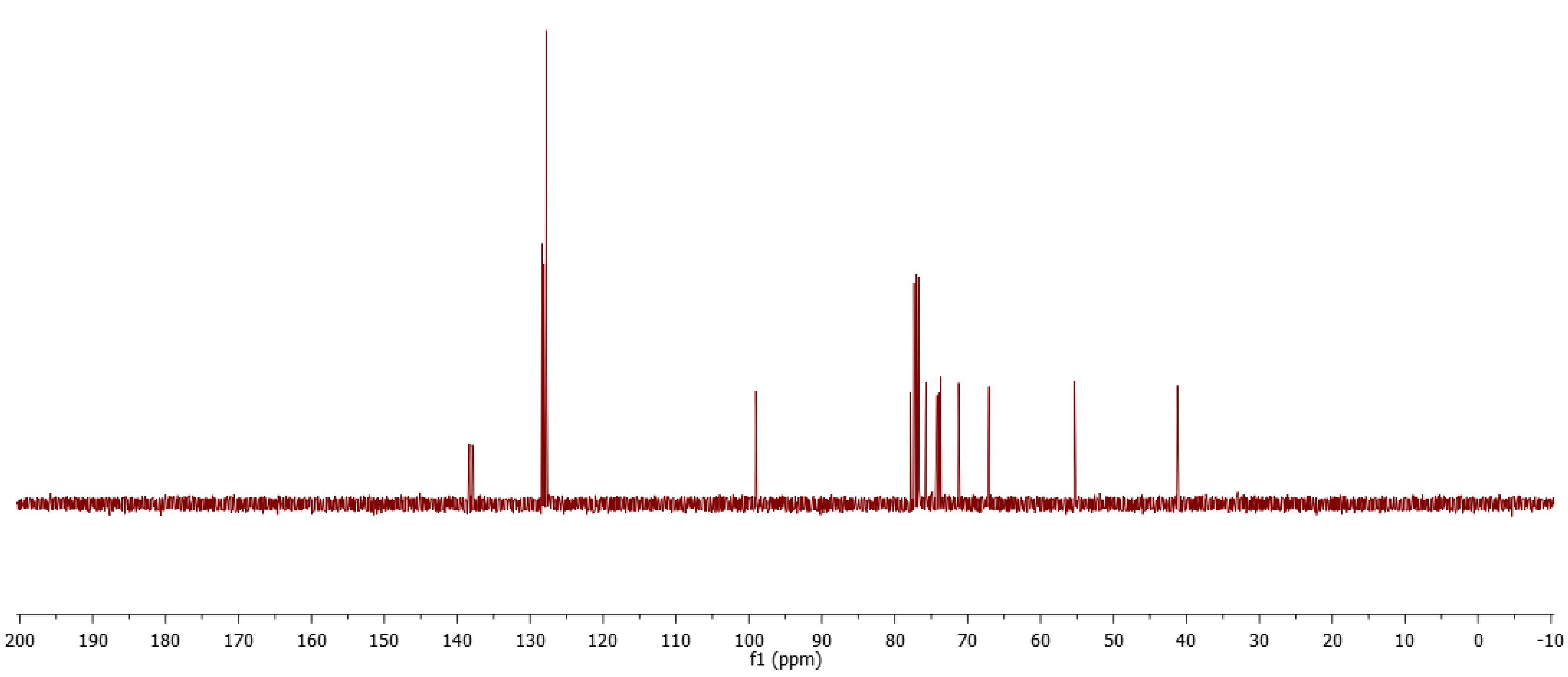

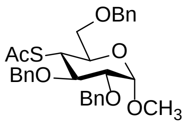

15iii

^{1}H NMR
600 MHz
$CDCl_3$

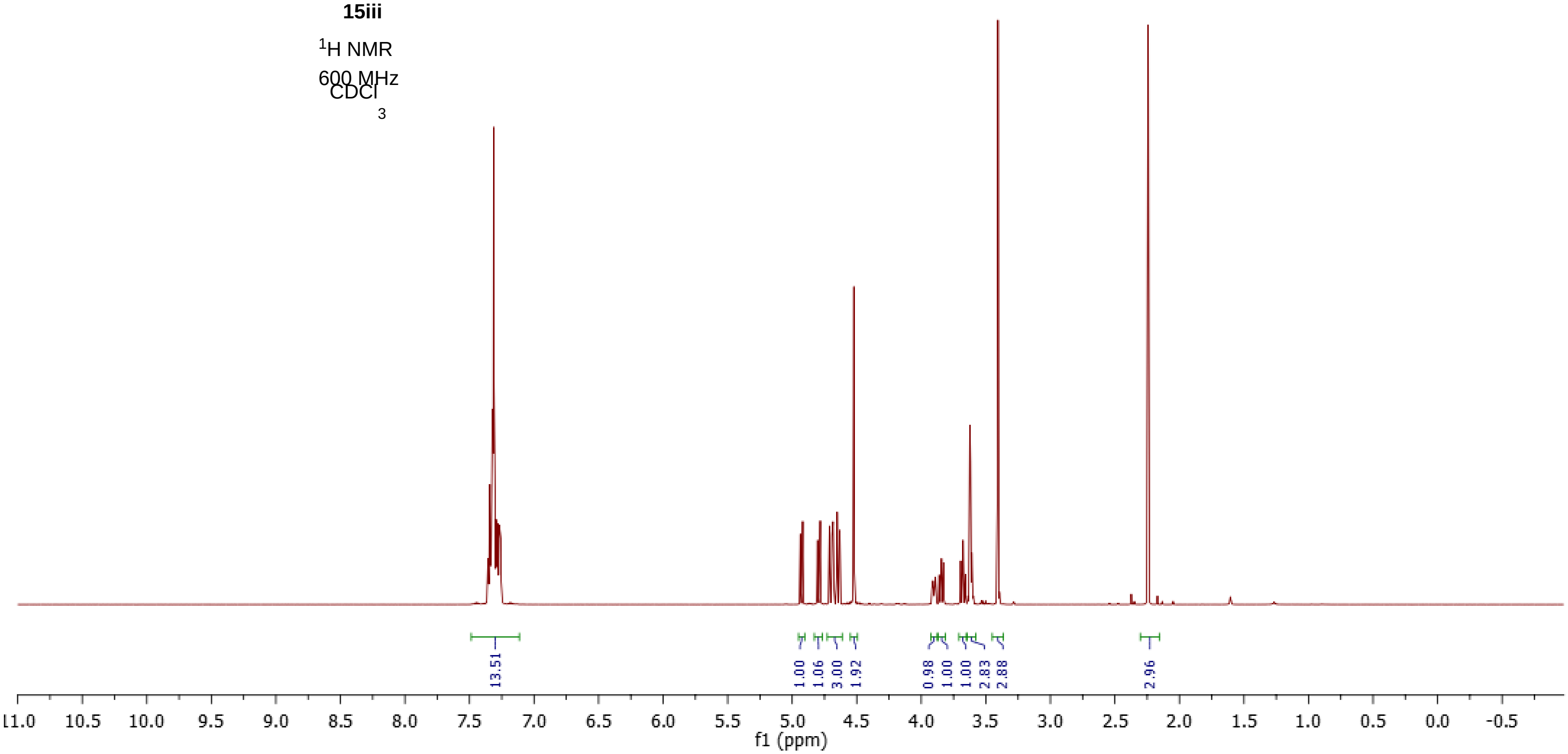

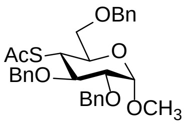

15iii

^{13}C NMR, 150 MHz, $CDCl_3$

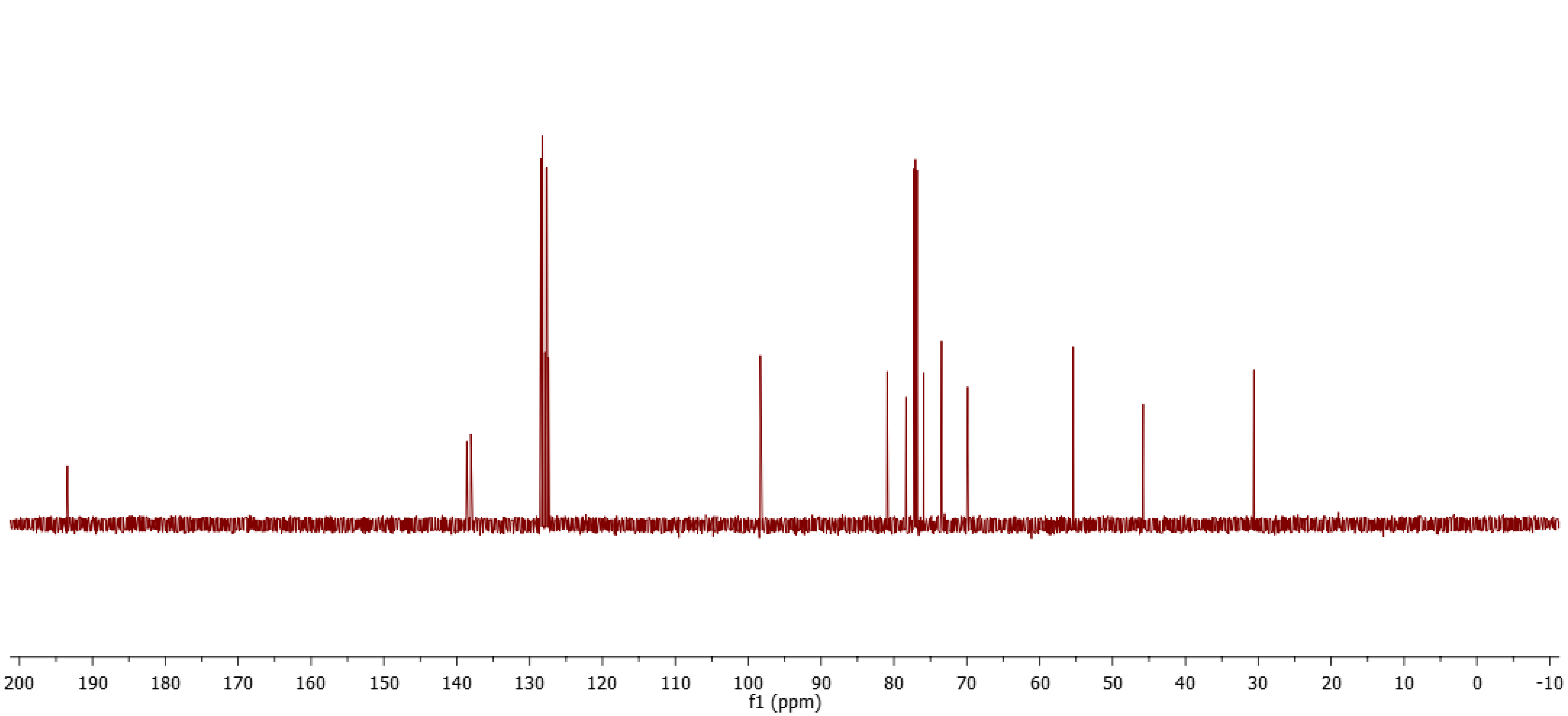

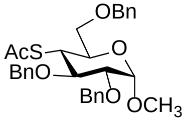

15iii

^1H-^1H COSY, 600 MHz, $CDCl_3$

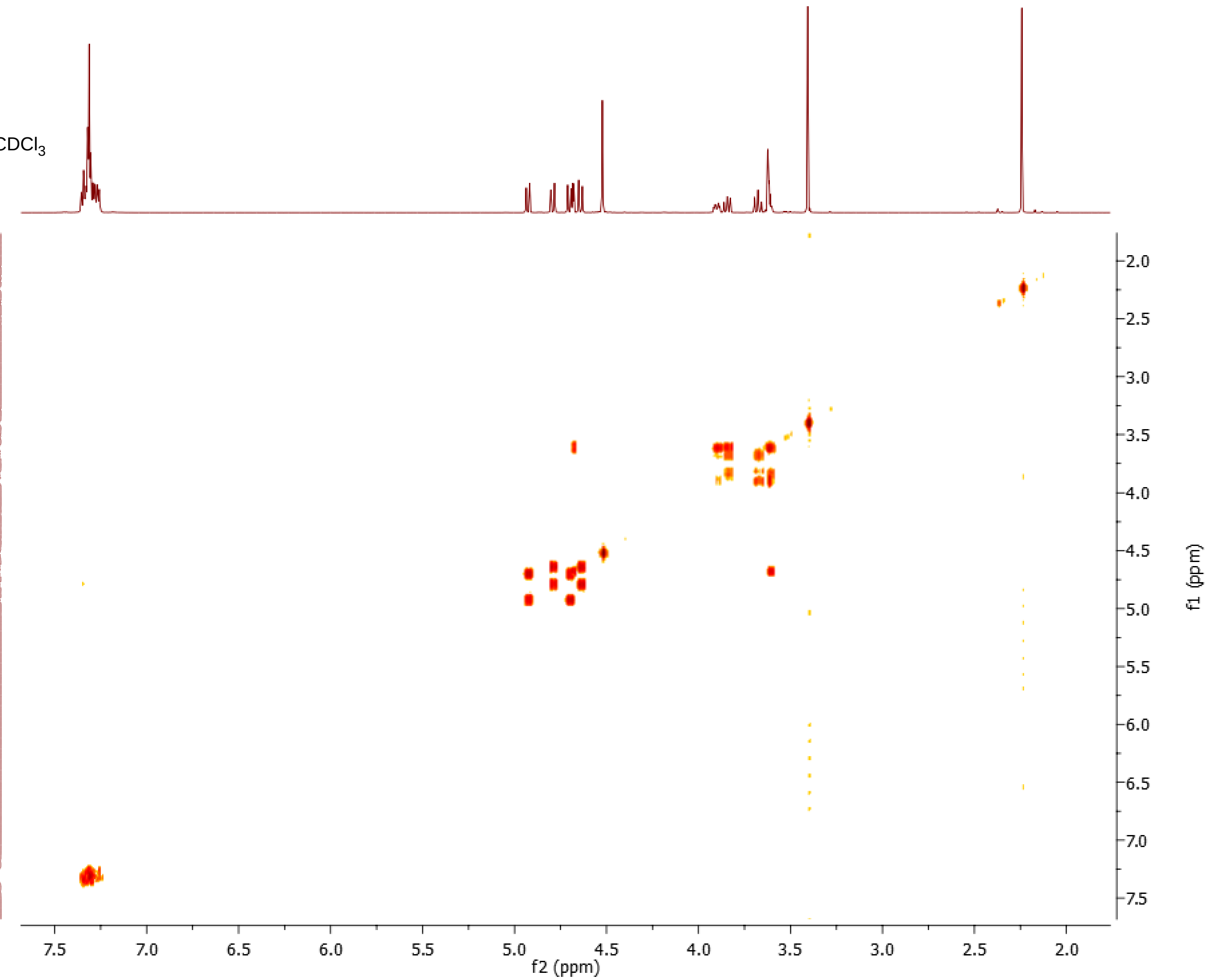

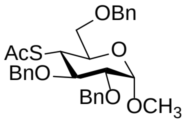

15iii

^1H-^{13}C HSQC, 600/150MHz, $CDCl_3$

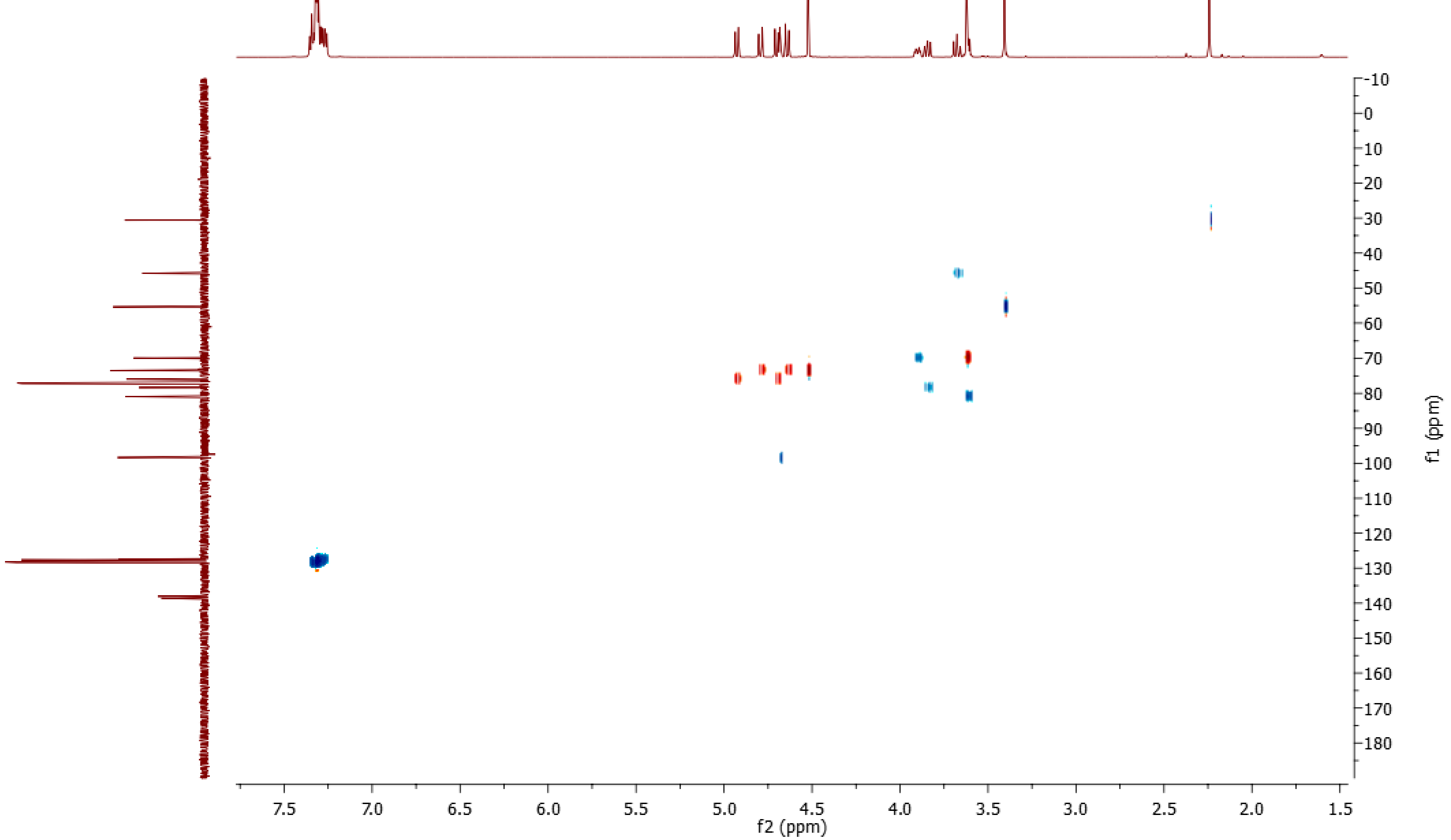

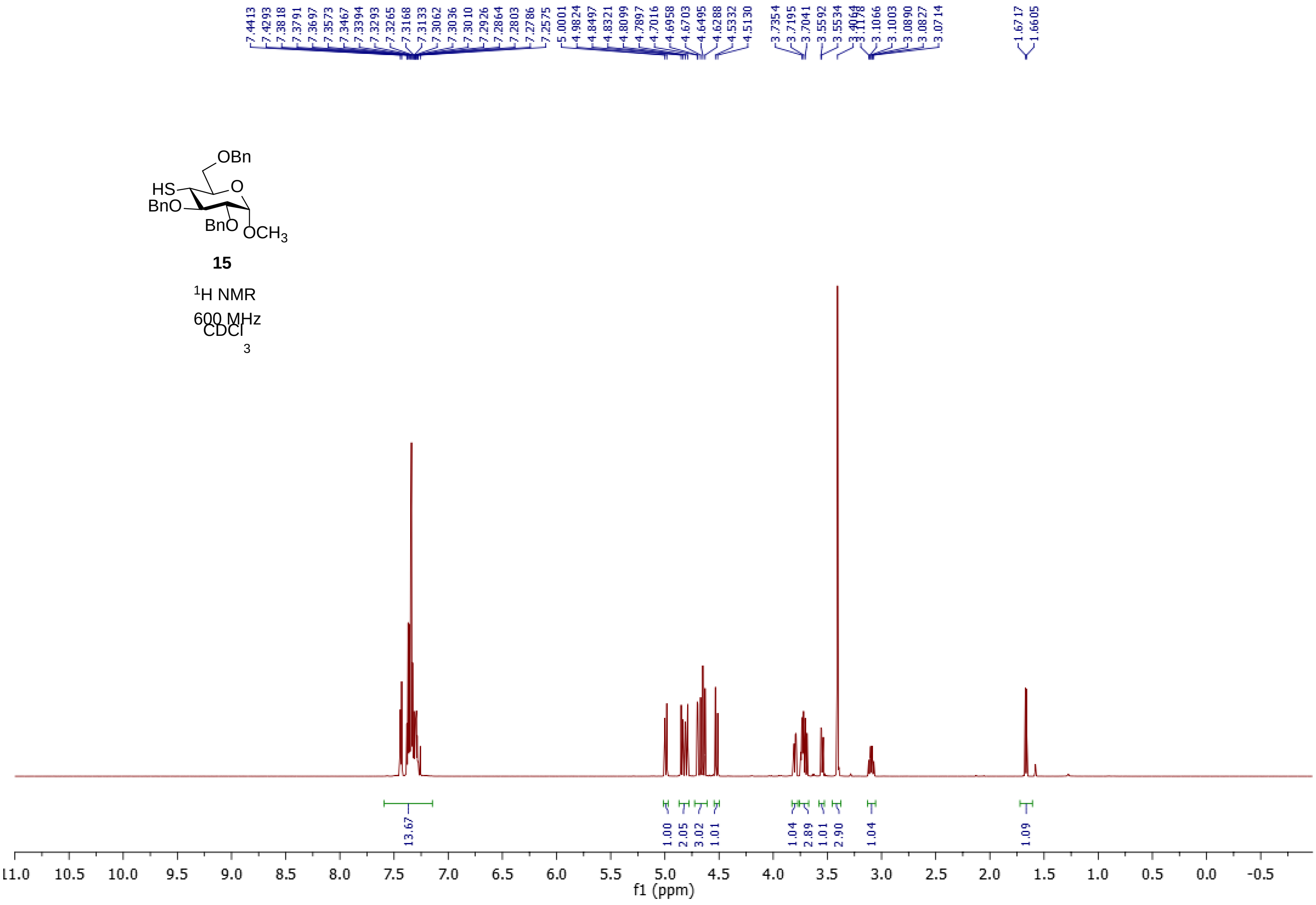

OBn
HS
O
BnO
BnO
OCH3
15
1H NMR
600 MHz
CDCl3
7.4413
7.4293
7.3818
7.3791
7.3697
7.3573
7.3467
7.3394
7.3293
7.3265
7.3168
7.3133
7.3062
7.3036
7.3010
7.2926
7.2864
7.2803
7.2786
7.2575
5.0001
4.9824
4.8497
4.8321
4.8099
4.7897
4.7016
4.6958
4.6703
4.6495
4.6288
4.5332
4.5130
3.7354
3.7195
3.7041
3.5592
3.5534
3.4064
3.1178
3.1066
3.1003
3.0890
3.0827
3.0714
1.6717
1.6605
13.67
1.00
2.05
3.02
1.01
1.04
2.89
1.01
2.90
1.04
1.09
11.0
10.5
10.0
9.5
9.0
8.5
8.0
7.5
7.0
6.5
6.0
5.5
5.0
4.5
4.0
3.5
3.0
2.5
2.0
1.5
1.0
0.5
0.0
-0.5
f1 (ppm)

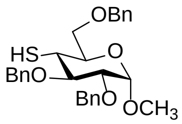

15

^{13}C NMR, 150 MHz, $CDCl_3$

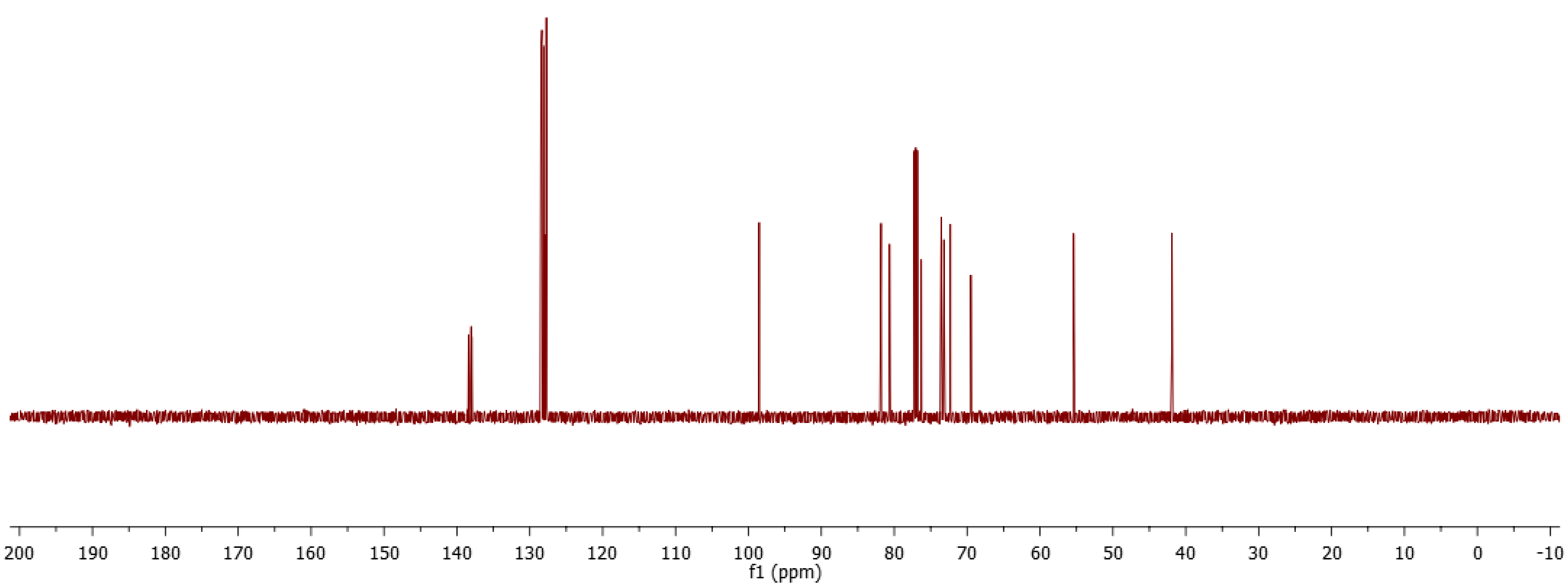

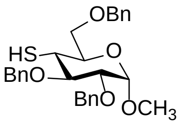

15

^{1}H-^{13}C HSQC, 600/150MHz, $CDCl_3$

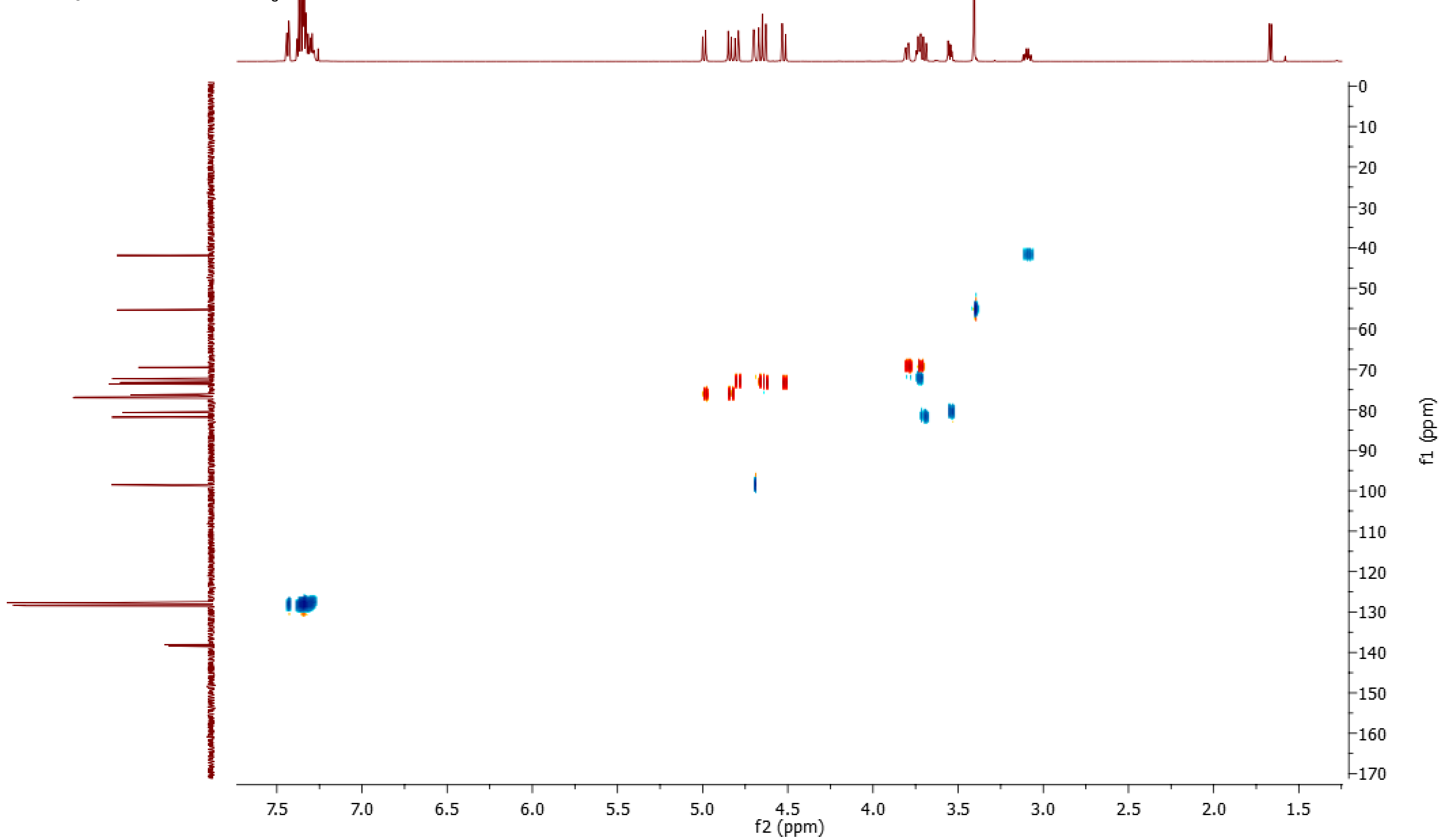

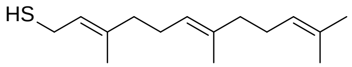

16

^{1}H NMR

600 MHz

$CDCl_3$

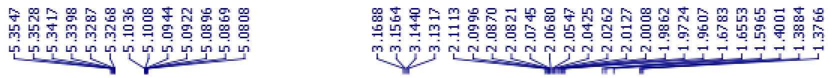

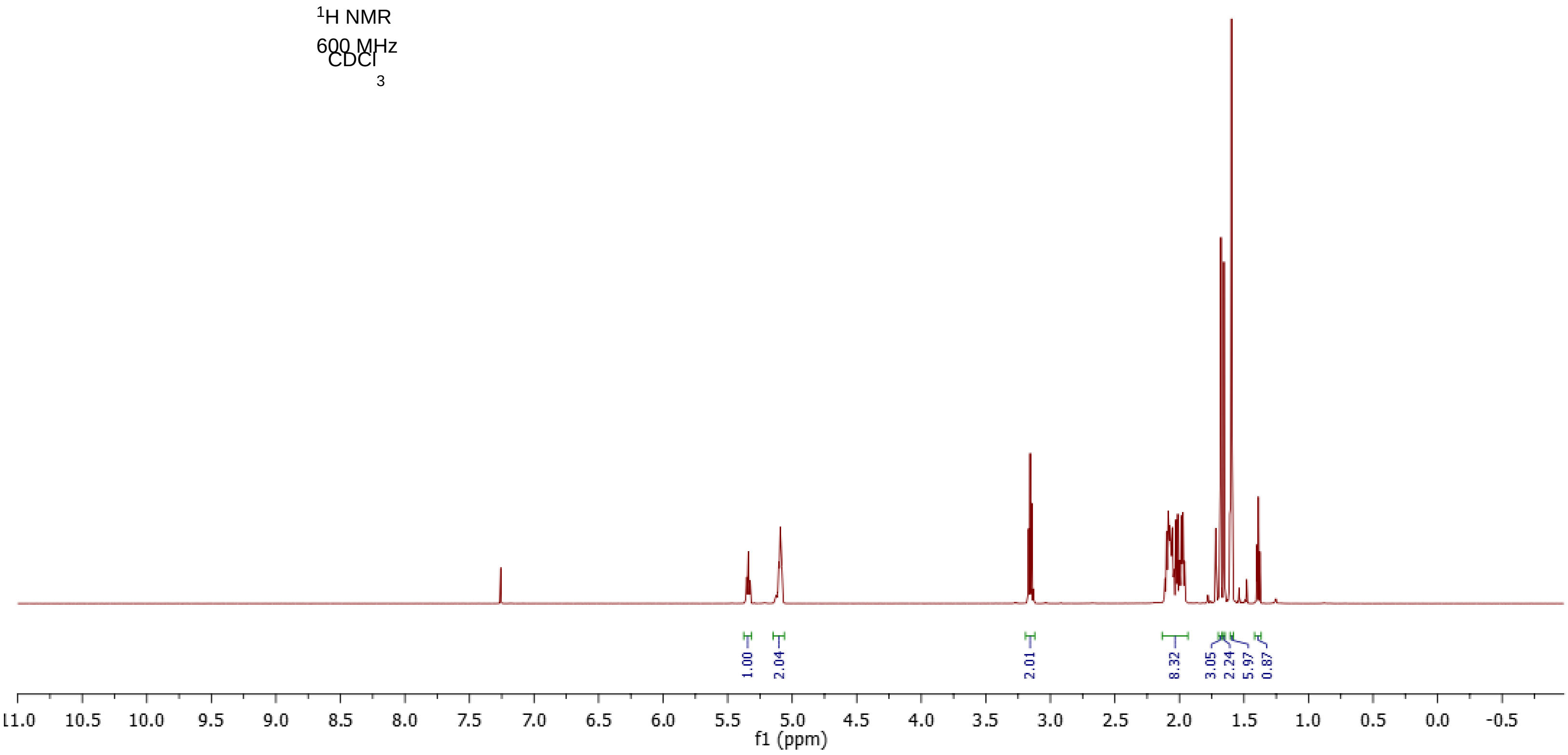

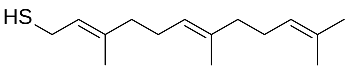

16

^{13}C NMR, 150 MHz, $CDCl_3$

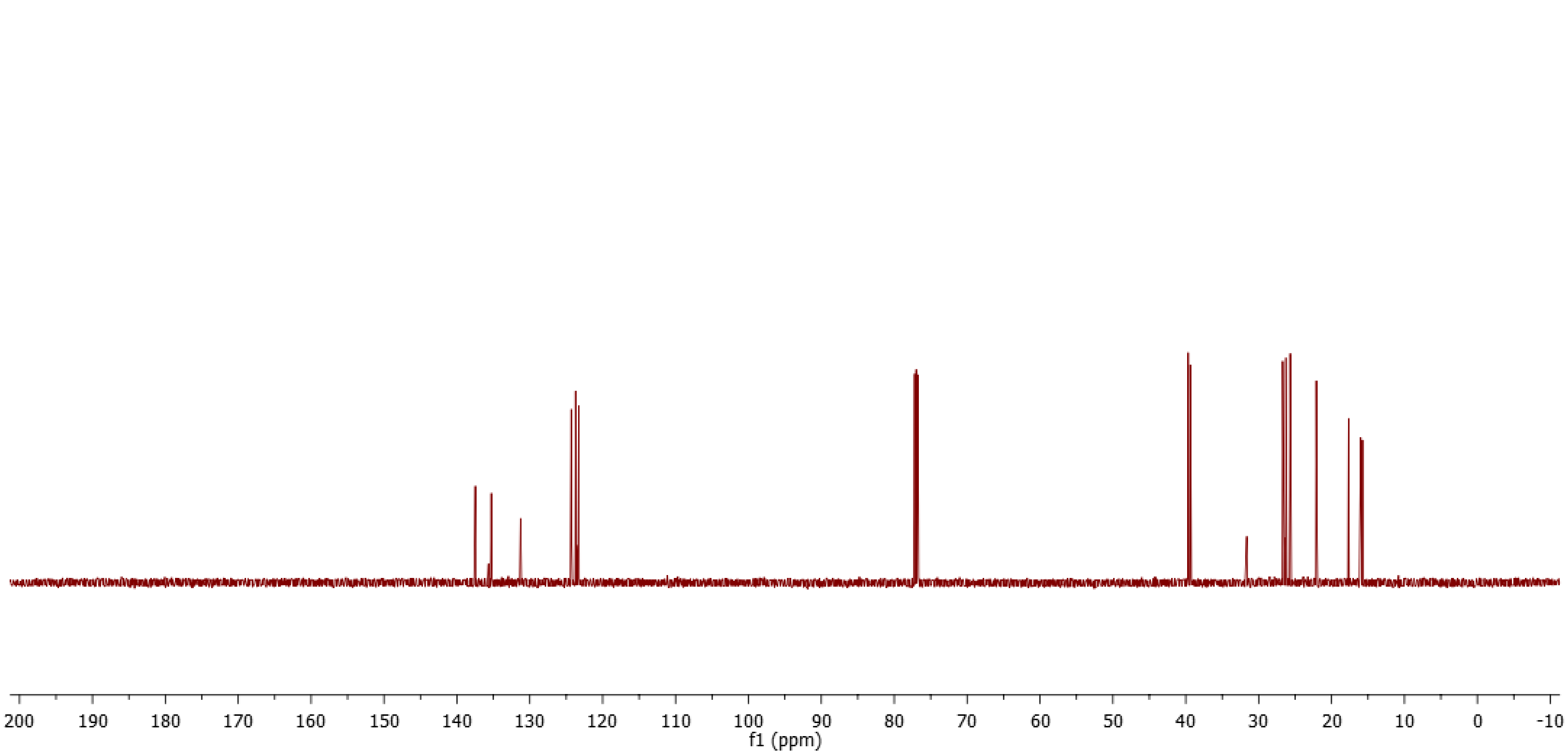

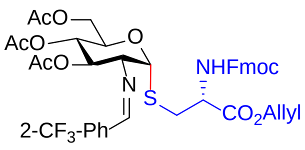

1a

^{1}H NMR
600 MHz
$CDCl_3$

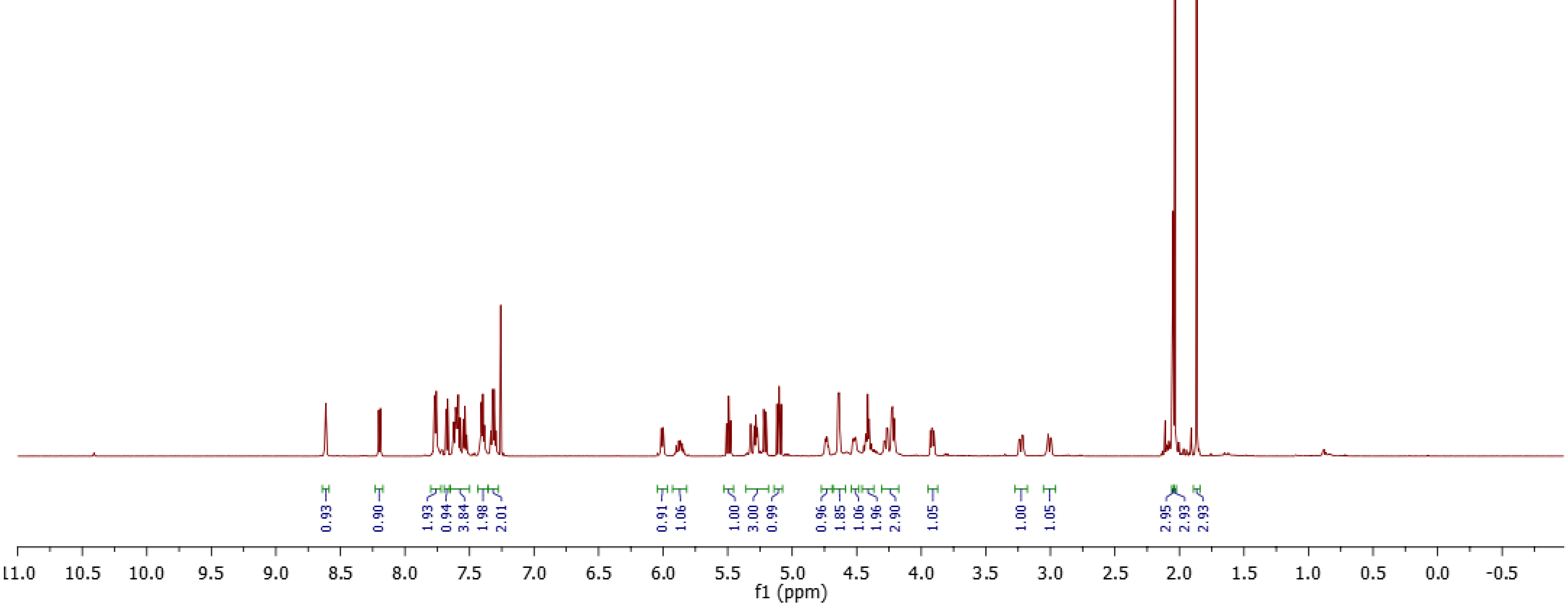

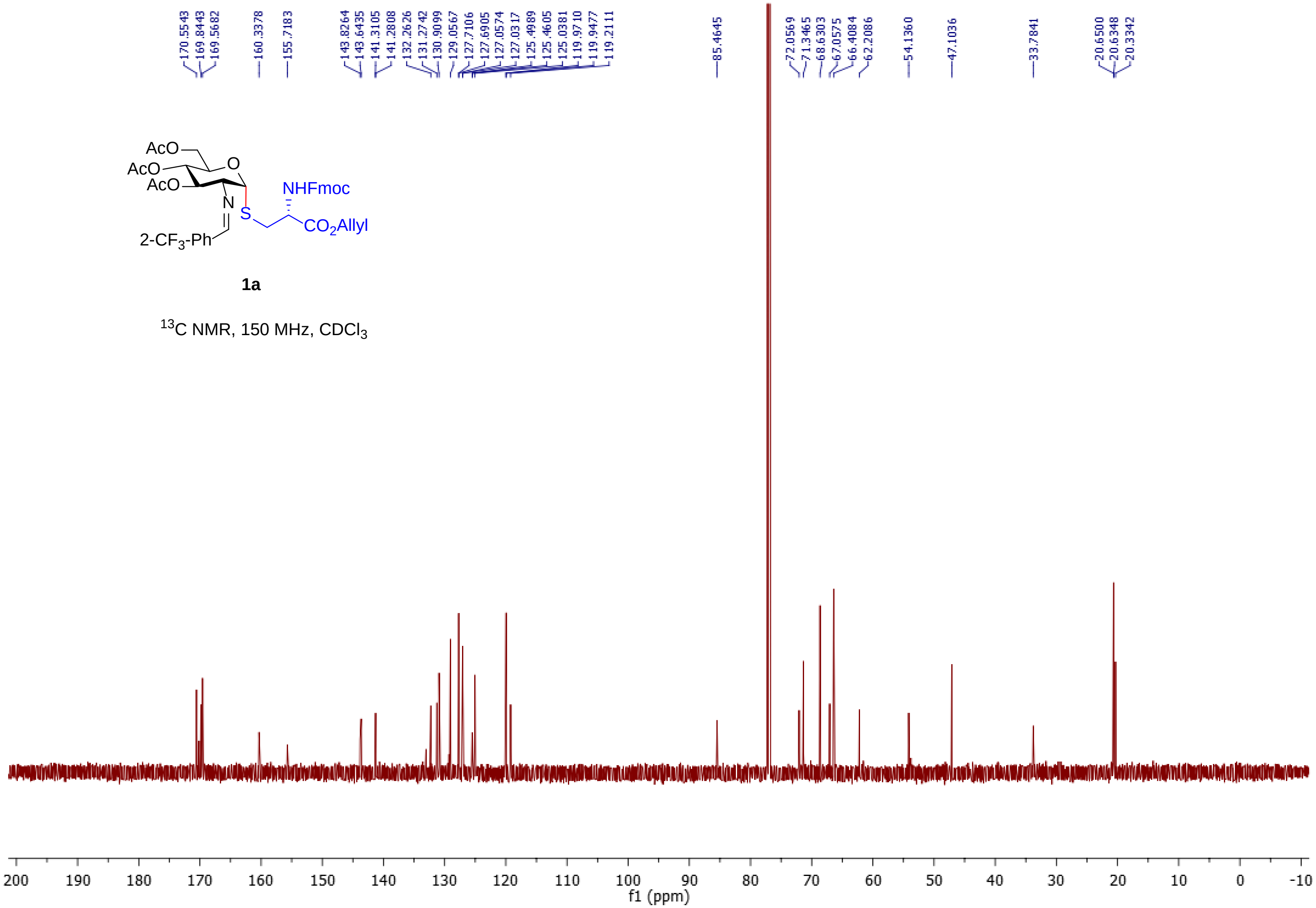
170.5543
169.8443
169.5682
160.3378
155.7183
143.8264
143.6435
141.3105
141.2808
132.2626
131.2742
130.9099
129.0567
127.7106
127.6905
127.0574
127.0317
125.4989
125.4605
125.0381
119.9710
119.9477
119.2111
85.4645
72.0569
71.3465
68.6303
67.0575
66.4084
62.2086
54.1360
47.1036
33.7841
20.6500
20.6348
20.3342
AcO
AcO
AcO
O
N
S
NHFmoc
CO_2Allyl
2-CF_3-Ph
1a
^{13}C NMR, 150 MHz, $CDCl_3$
200 190 180 170 160 150 140 130 120 110 100 90 80 70 60 50 40 30 20 10 0 -10
f1 (ppm)

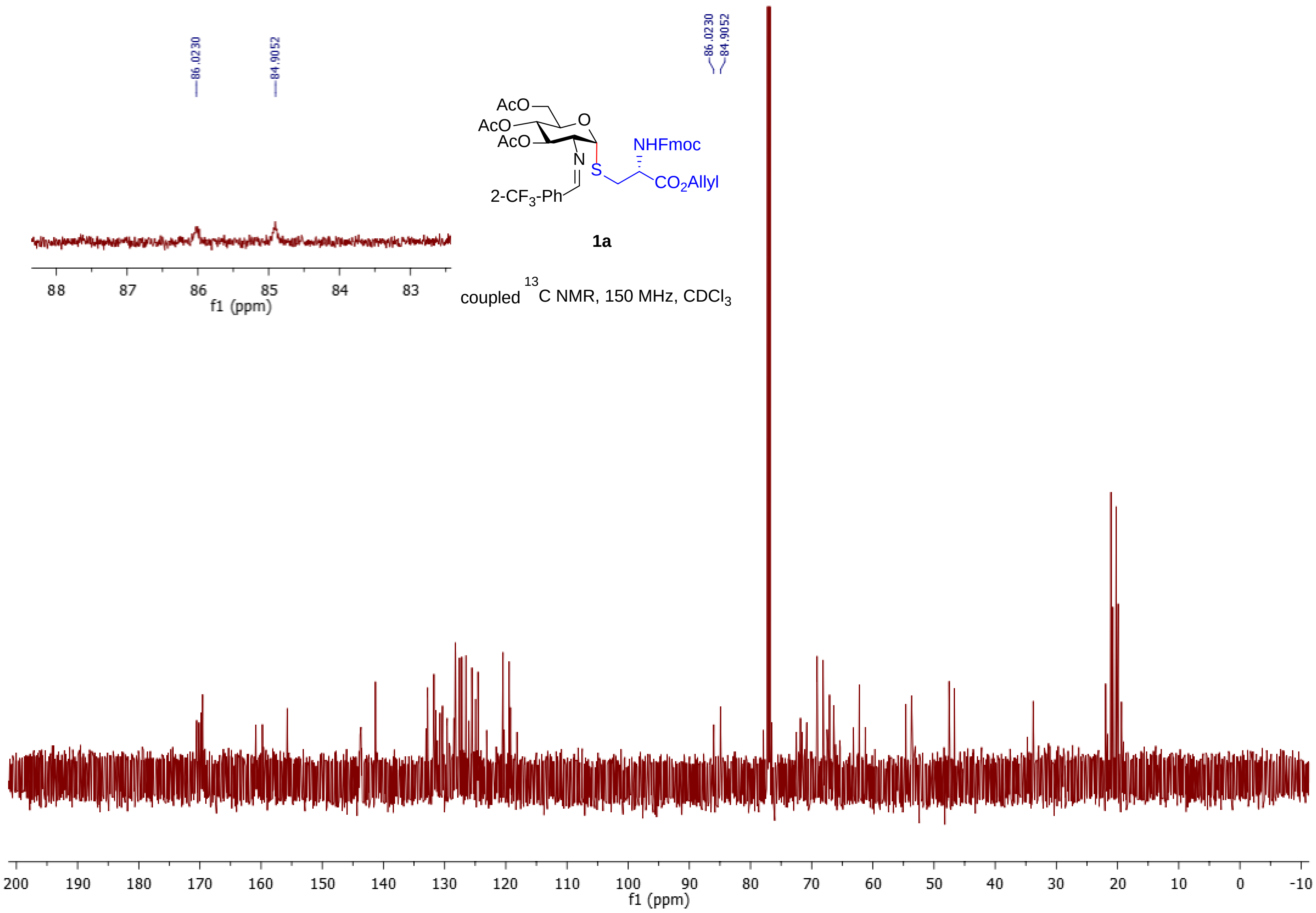

86.0230
84.9052
AcO
AcO
AcO
O
N
S
NHFmoc
CO_2Allyl
2-CF_3-Ph
1a
coupled ^{13}C NMR, 150 MHz, $CDCl_3$
88 87 86 85 84 83
f1 (ppm)
200 190 180 170 160 150 140 130 120 110 100 90 80 70 60 50 40 30 20 10 0 -10
f1 (ppm)

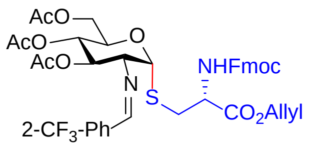

1a

^{1}H-^{1}H COSY, 600 MHz, $CDCl_3$

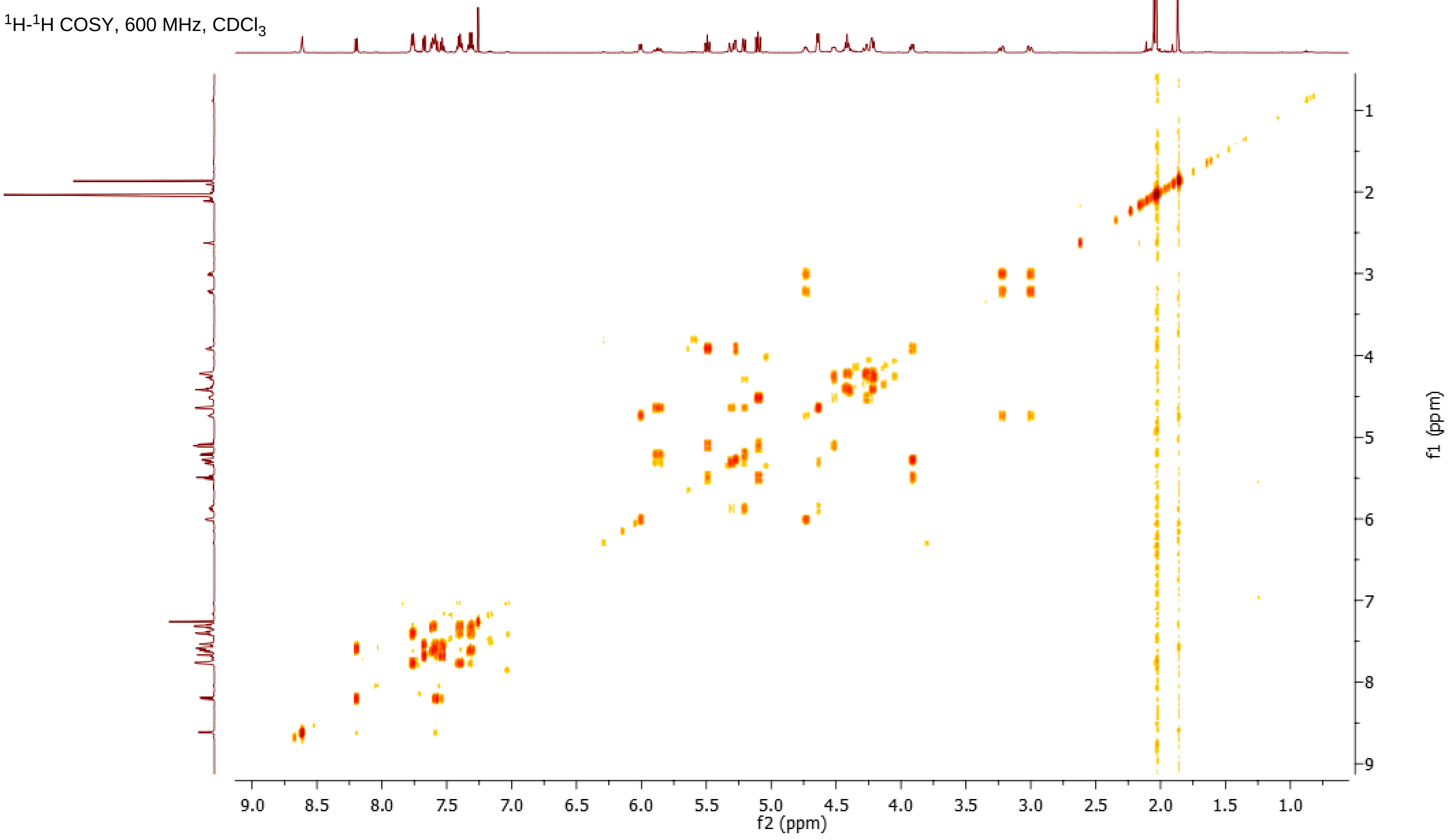

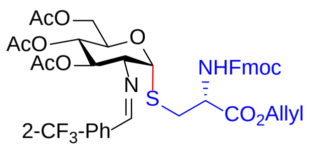

1a

^{1}H-^{13}C HSQC, 600/150MHz, $CDCl_3$

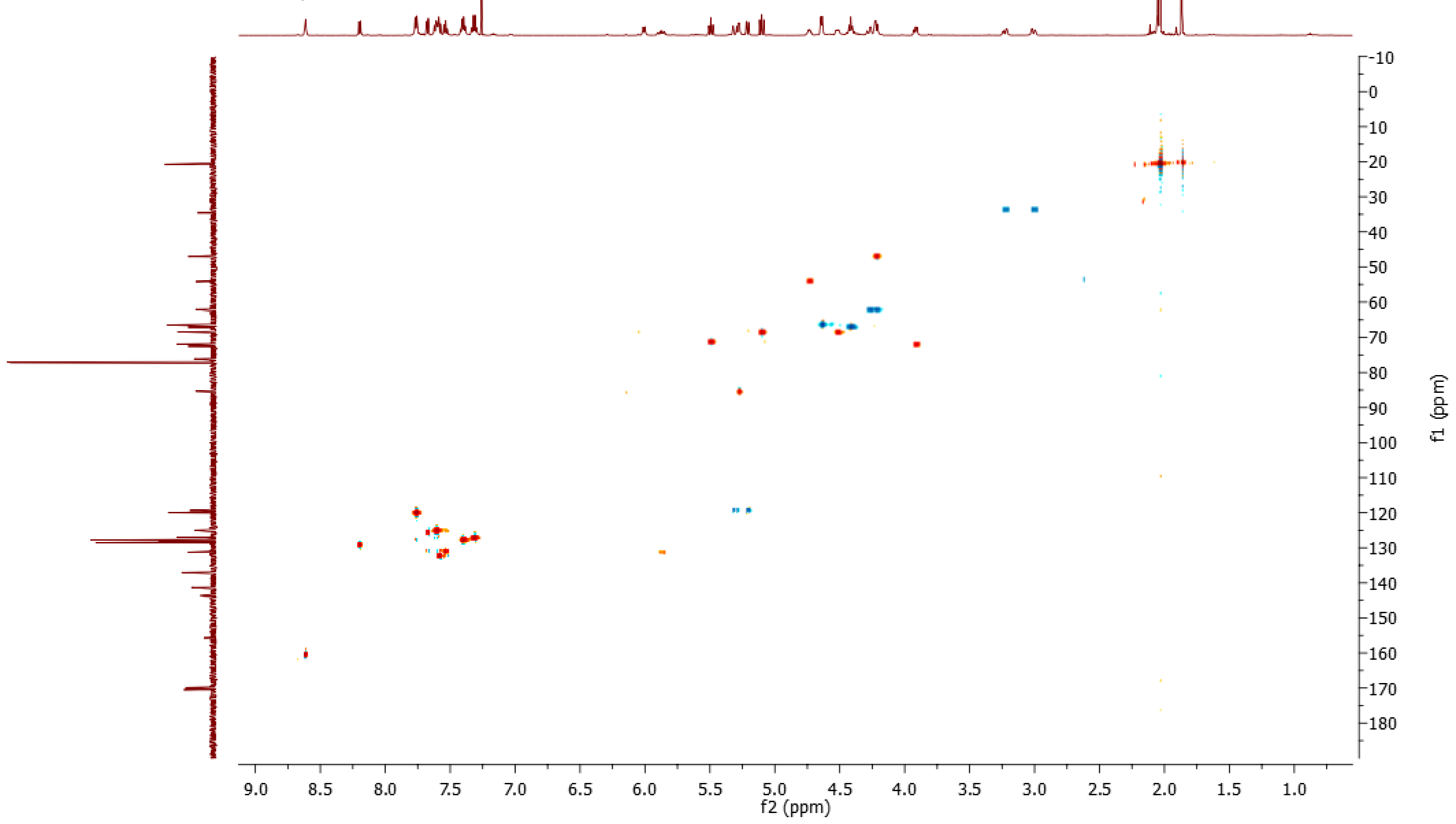

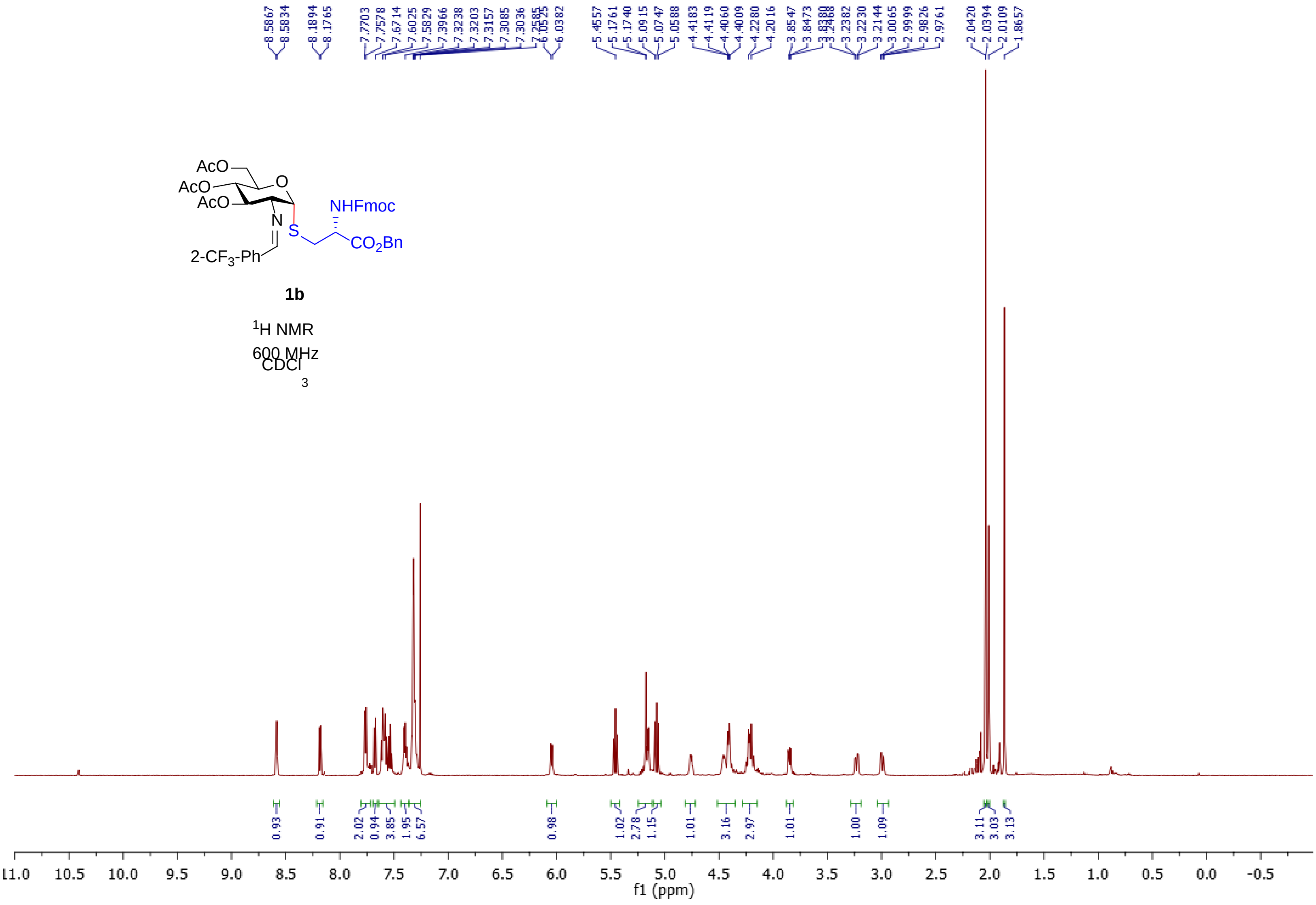
AcO
AcO
AcO
O
N
S
NHFmoc
CO2Bn
2-CF3-Ph
1b
1H NMR
600 MHz
CDCl3
f1 (ppm)

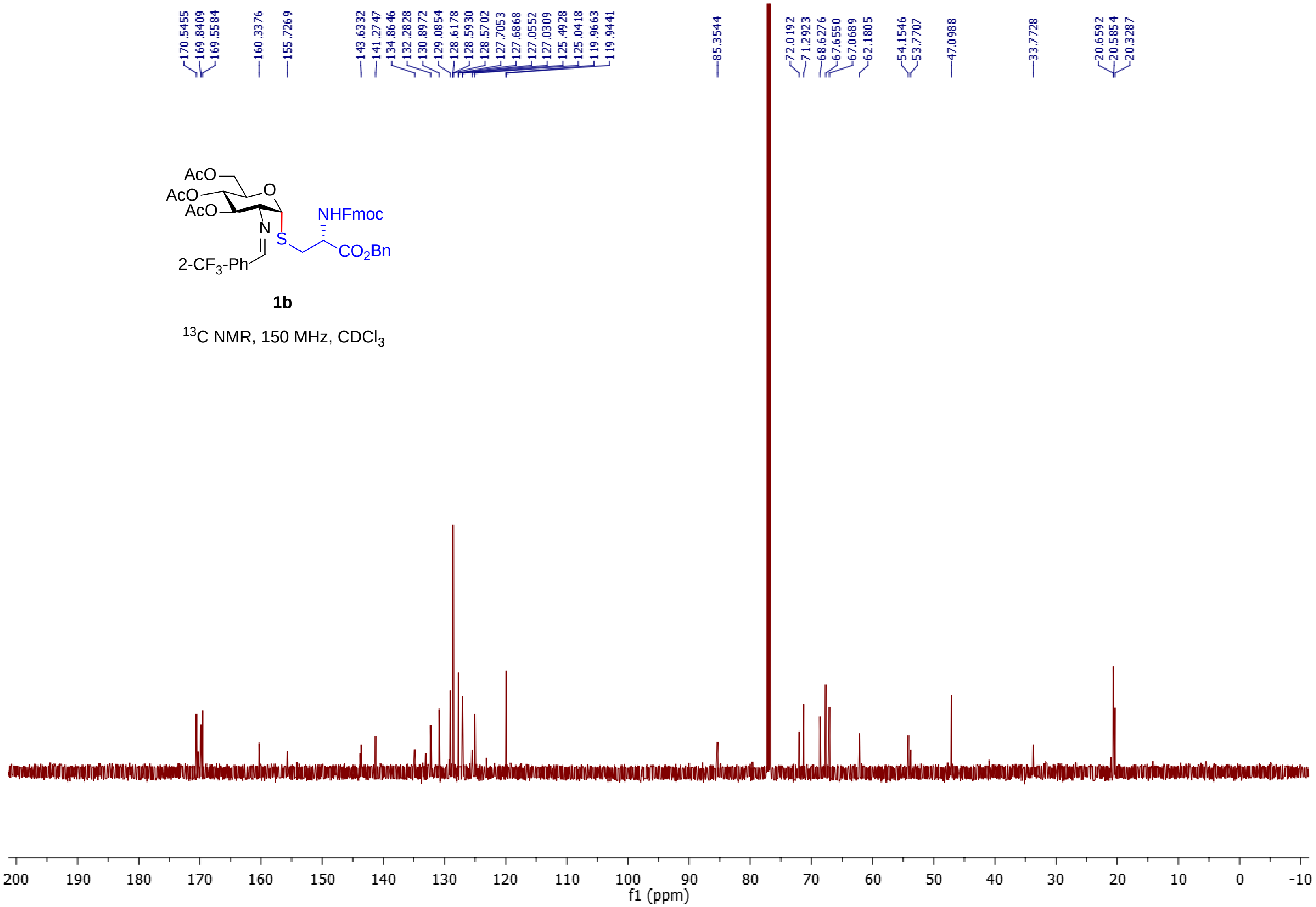

170.5455
169.8409
169.5584
160.3376
155.7269
143.6332
141.2747
134.8646
132.2828
130.8972
129.0854
128.6178
128.5930
128.5702
127.7053
127.6868
127.0552
127.0309
125.4928
125.0418
119.9663
119.9441
85.3544
72.0192
71.2923
68.6276
67.6550
67.0689
62.1805
54.1546
53.7707
47.0988
33.7728
20.6592
20.5854
20.3287
AcO
AcO
AcO
O
N
S
NHFmoc
CO_2Bn
2-CF_3-Ph
1b
^{13}C NMR, 150 MHz, $CDCl_3$
200 190 180 170 160 150 140 130 120 110 100 90 80 70 60 50 40 30 20 10 0 -10
f1 (ppm)

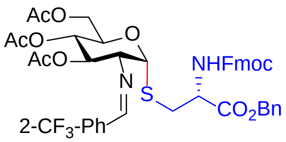

1b

^{1}H-^{1}H COSY, 600 MHz, $CDCl_3$

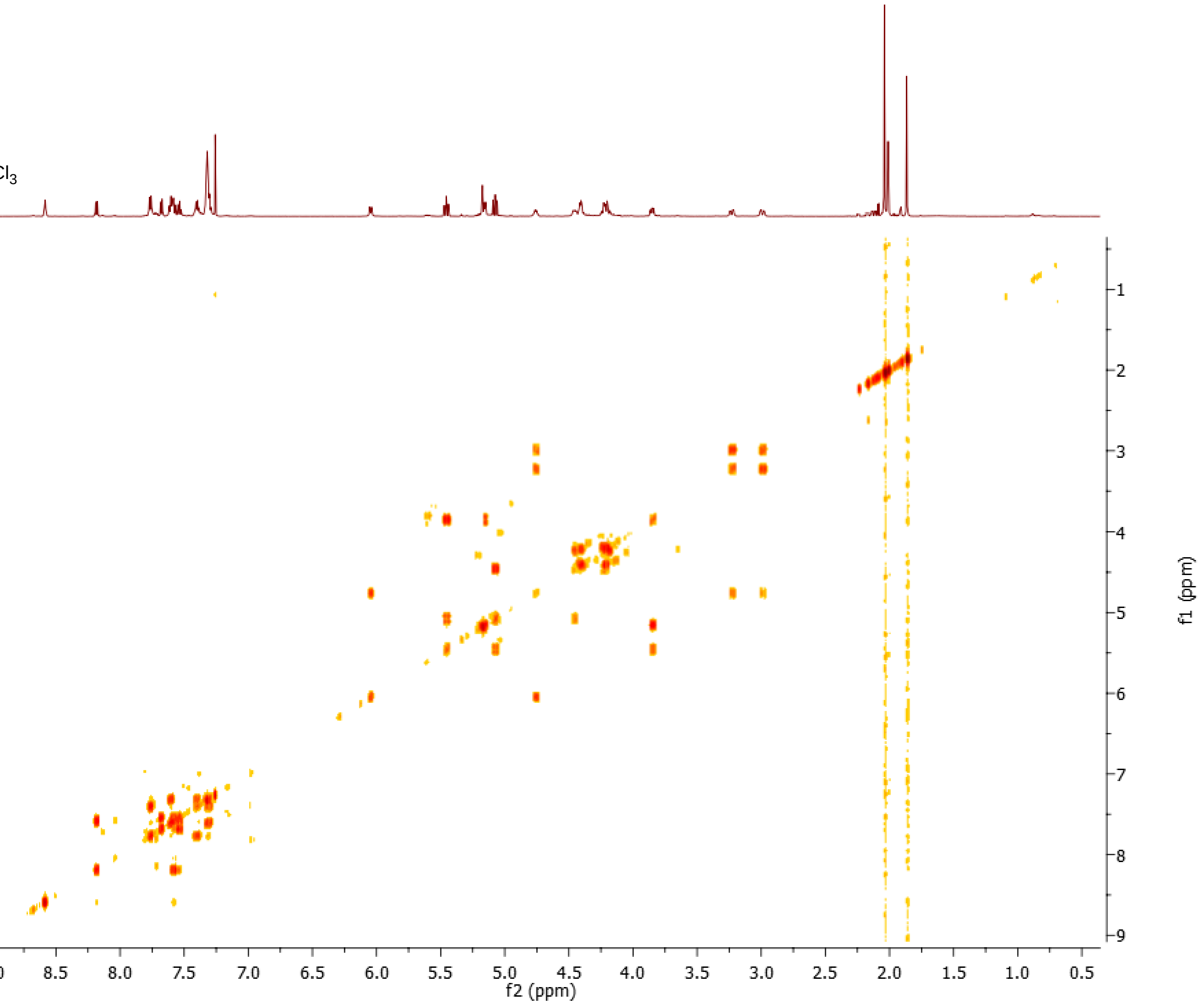

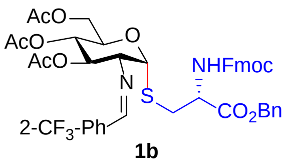

1b

^{1}H-^{13}C HSQC, 600/150MHz, $CDCl_3$

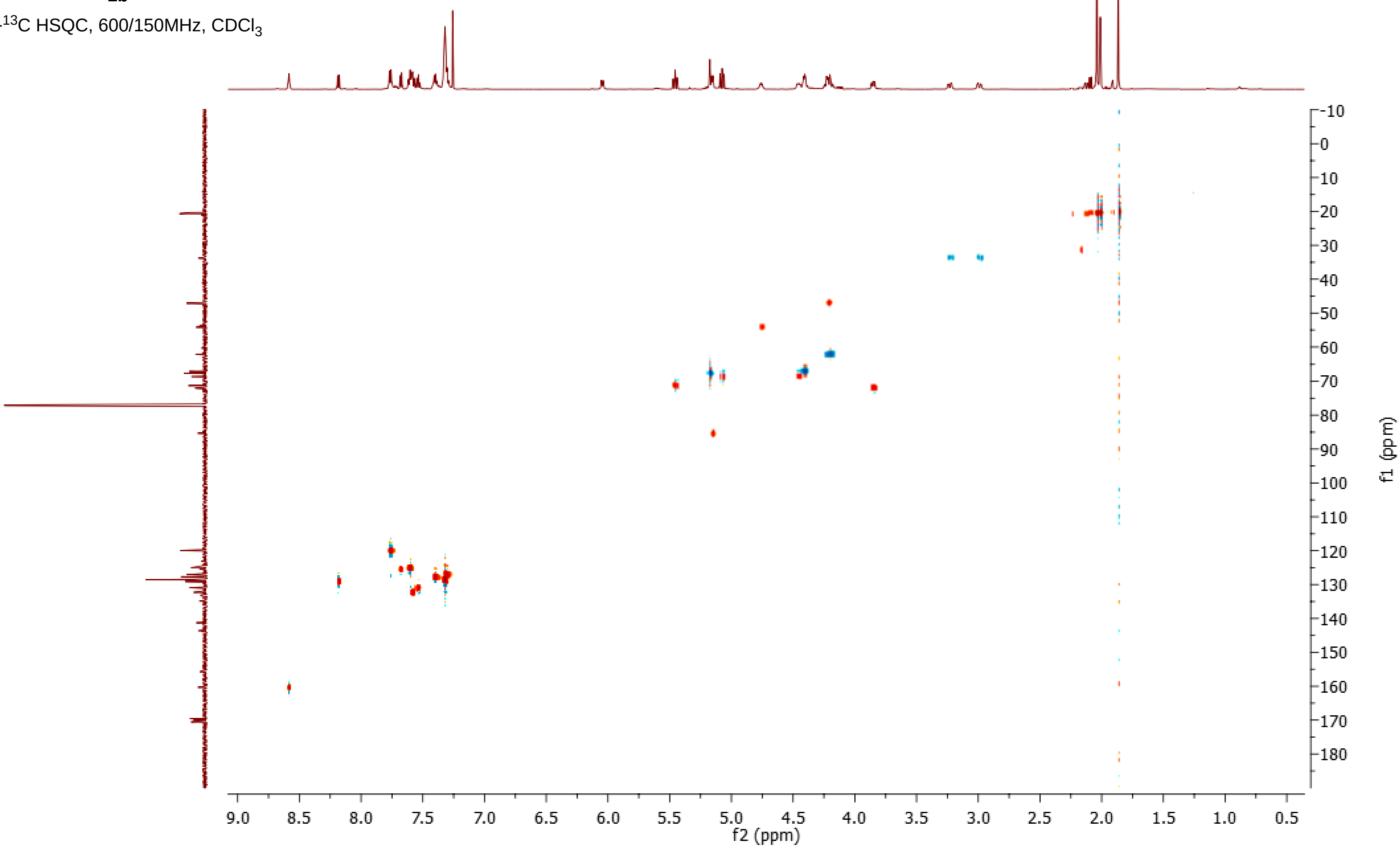

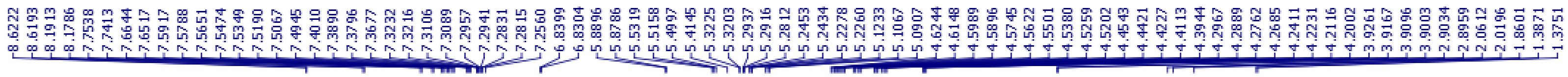

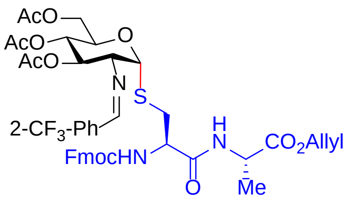

1c

^{1}H NMR
600 MHz
$CDCl_3$

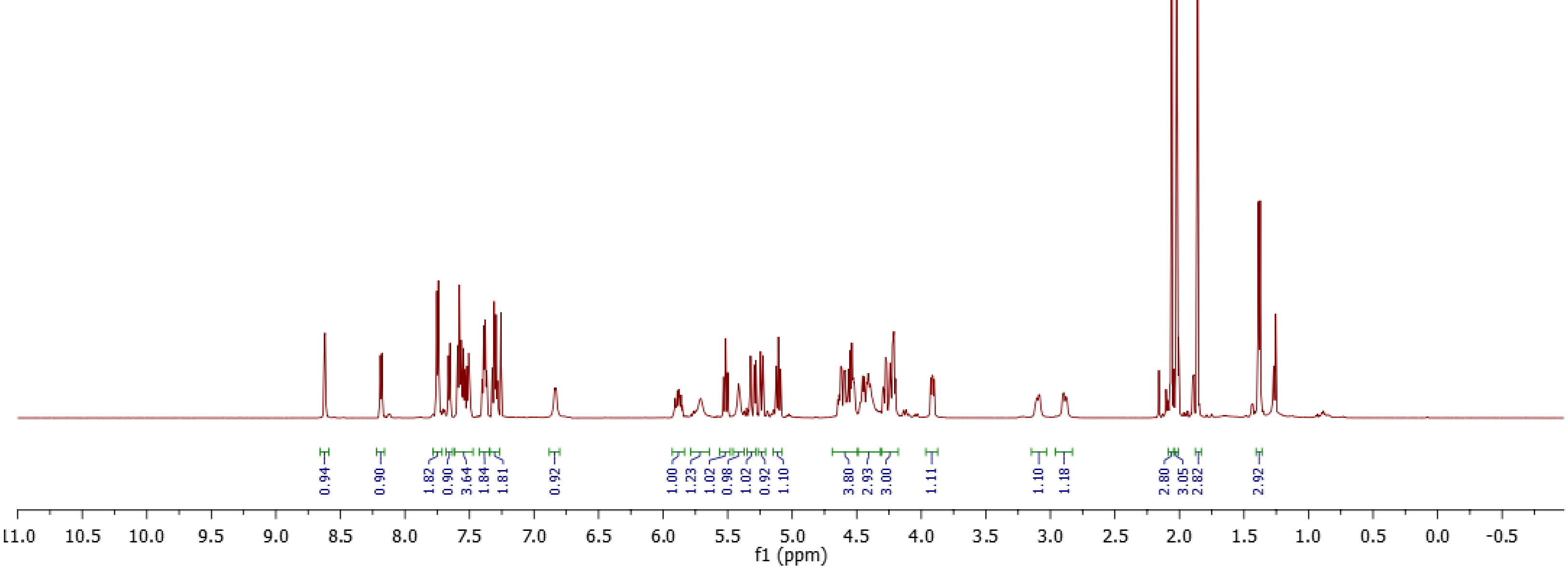

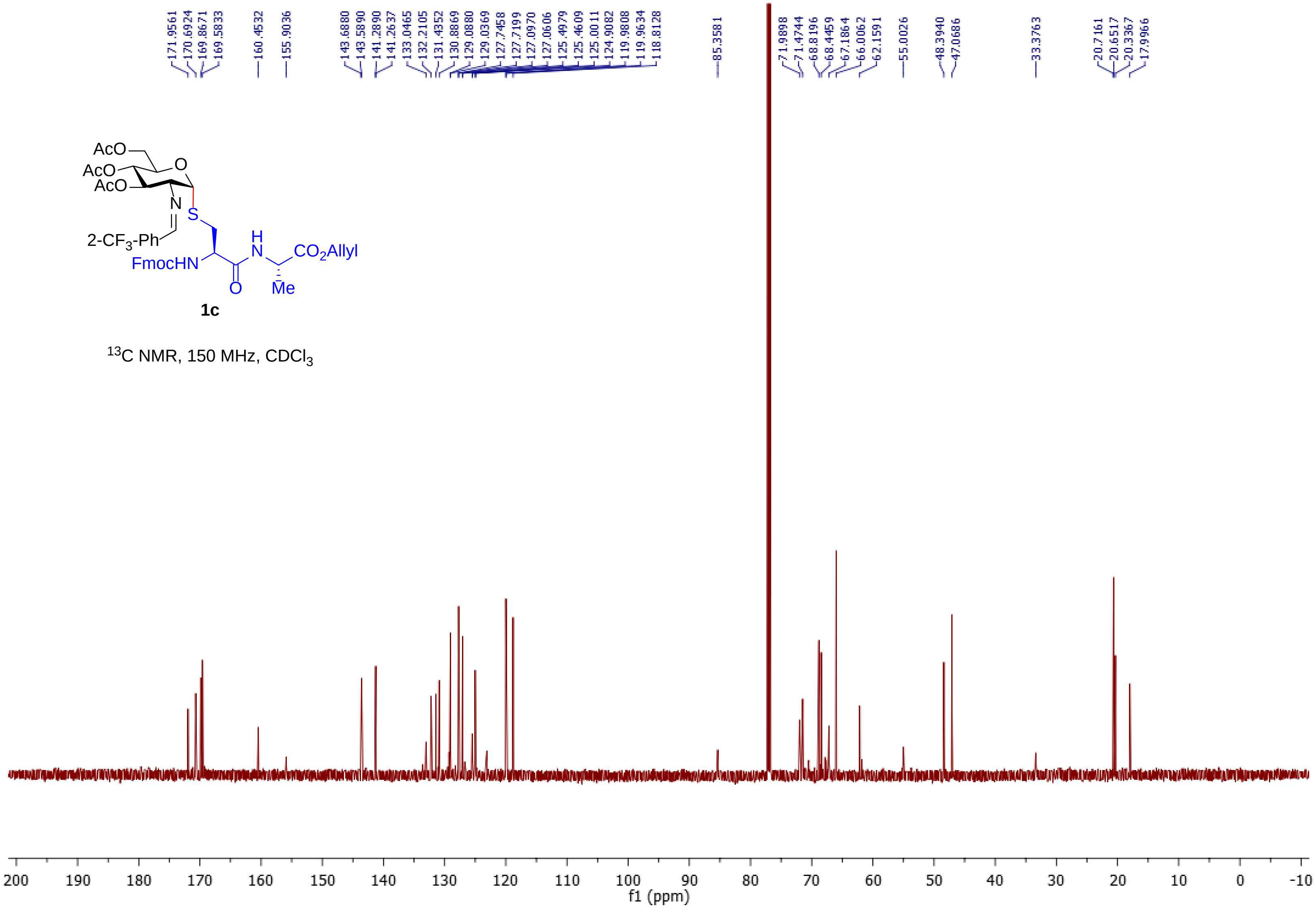

1c

^{13}C NMR, 150 MHz, $CDCl_3$

1c

coupled ^{13}C NMR, 150 MHz, $CDCl_3$

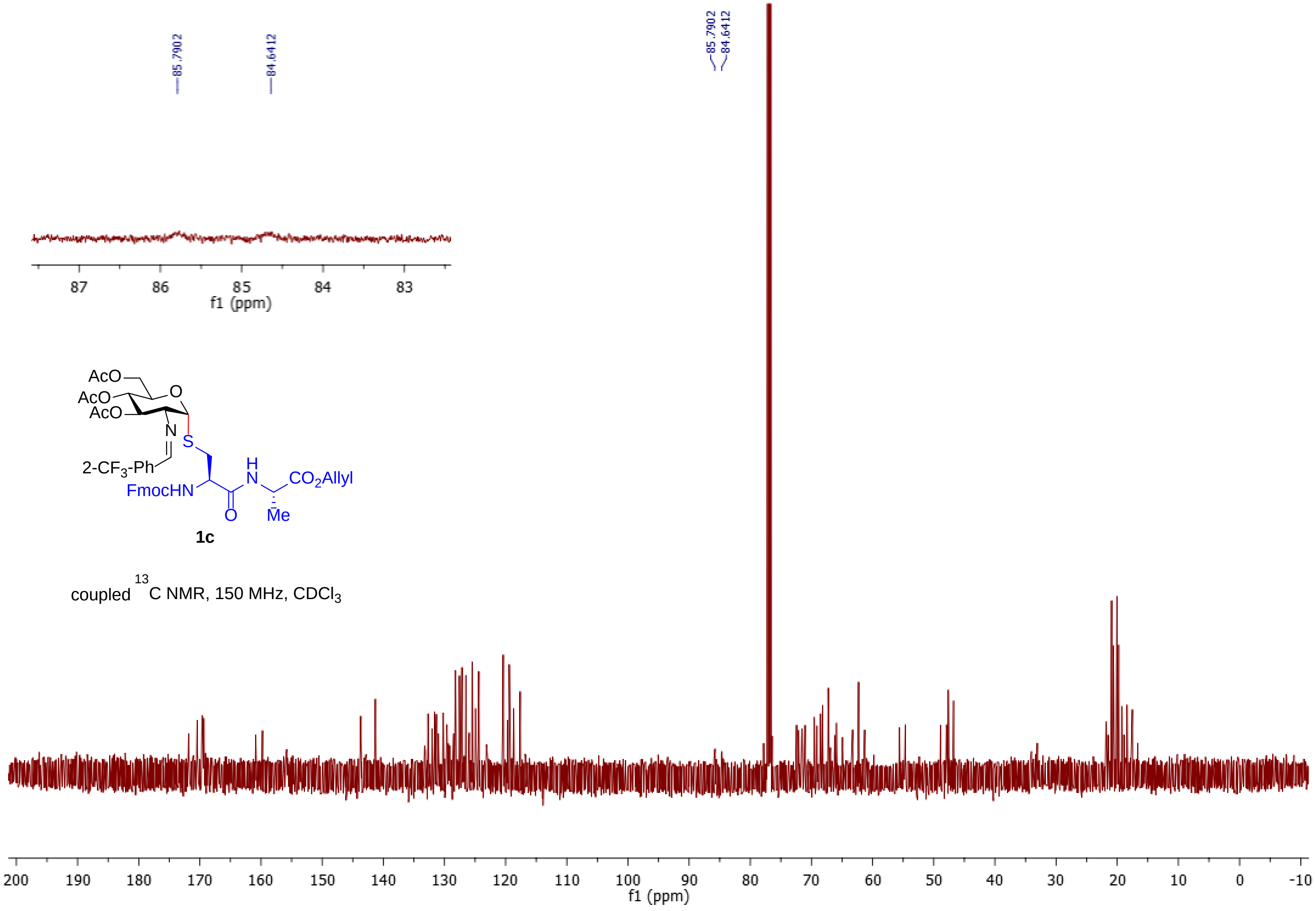

1c

^{1}H-^{13}C HSQC, 600/150MHz, $CDCl_3$

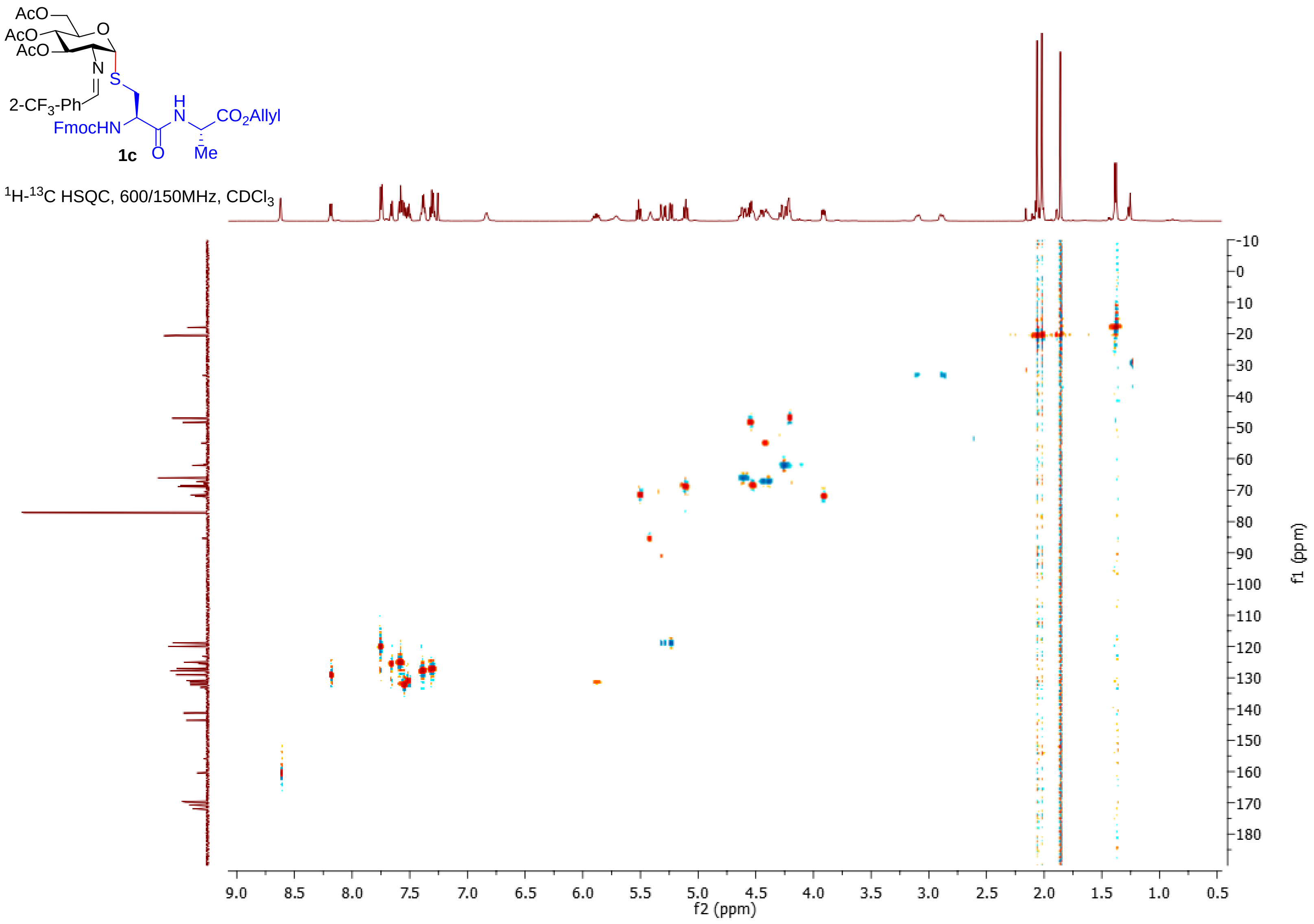

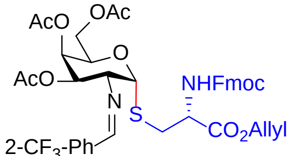

6a

^1H NMR
600 MHz
$CDCl_3$

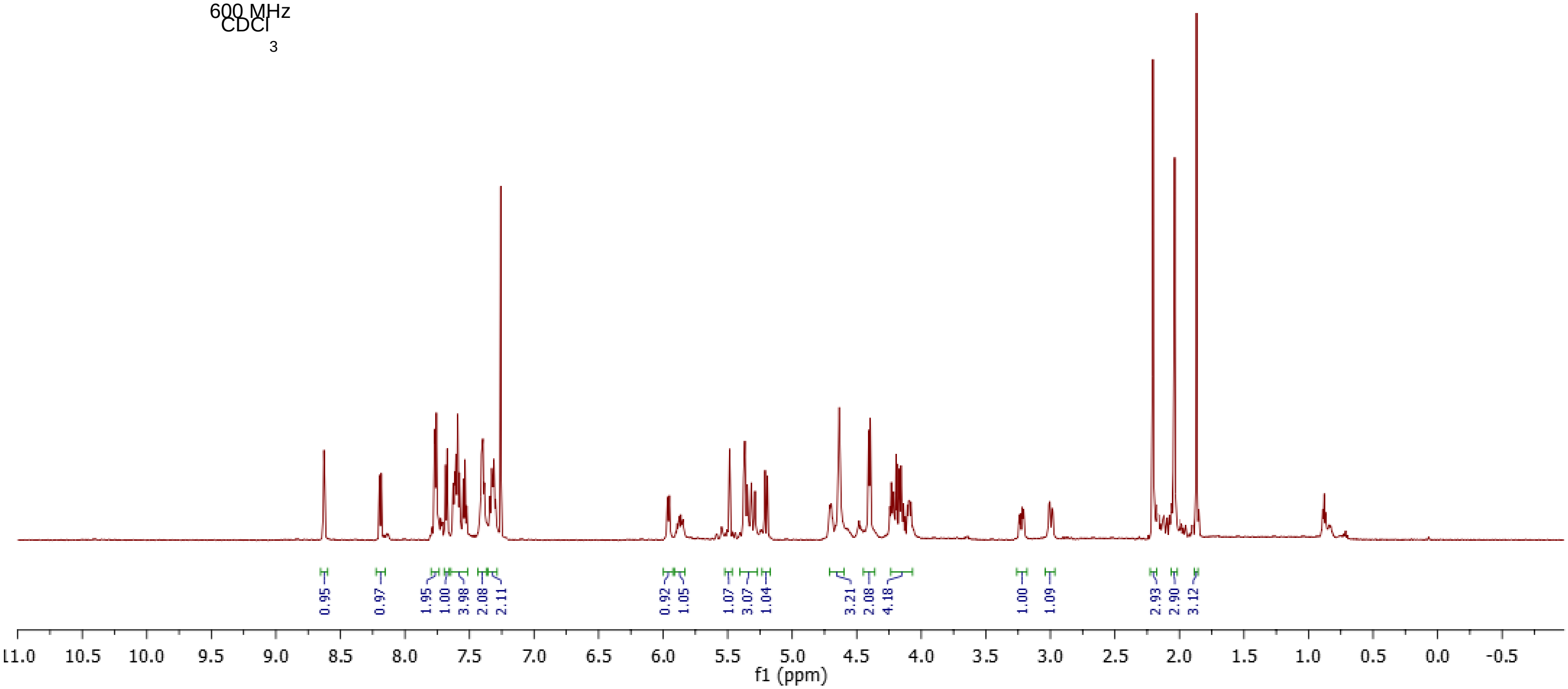

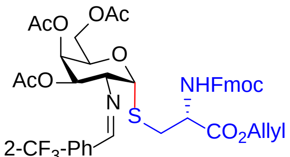

6a

^{13}C NMR, 150 MHz, $CDCl_3$

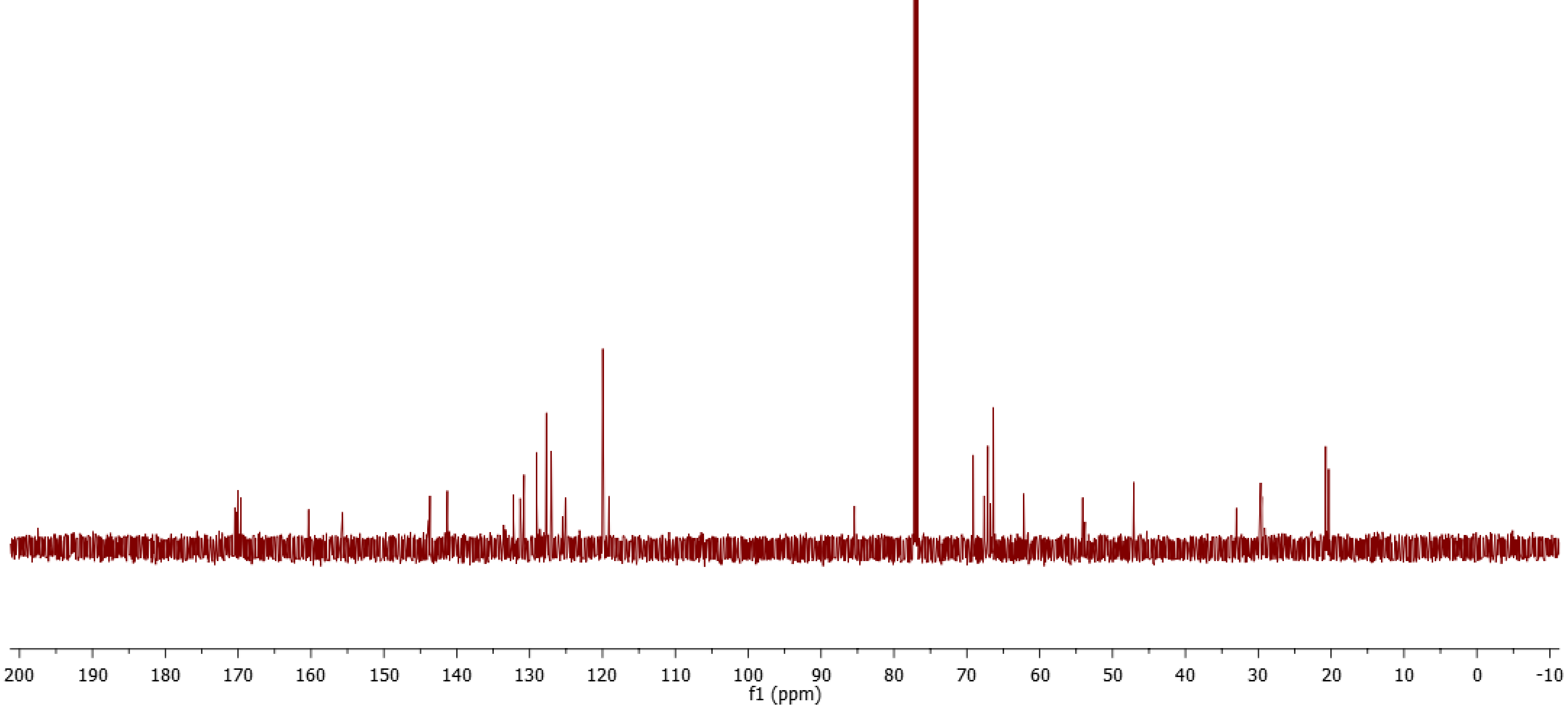

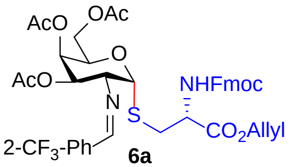

6a

^{1}H-^{13}C HSQC, 600/150MHz, $CDCl_3$

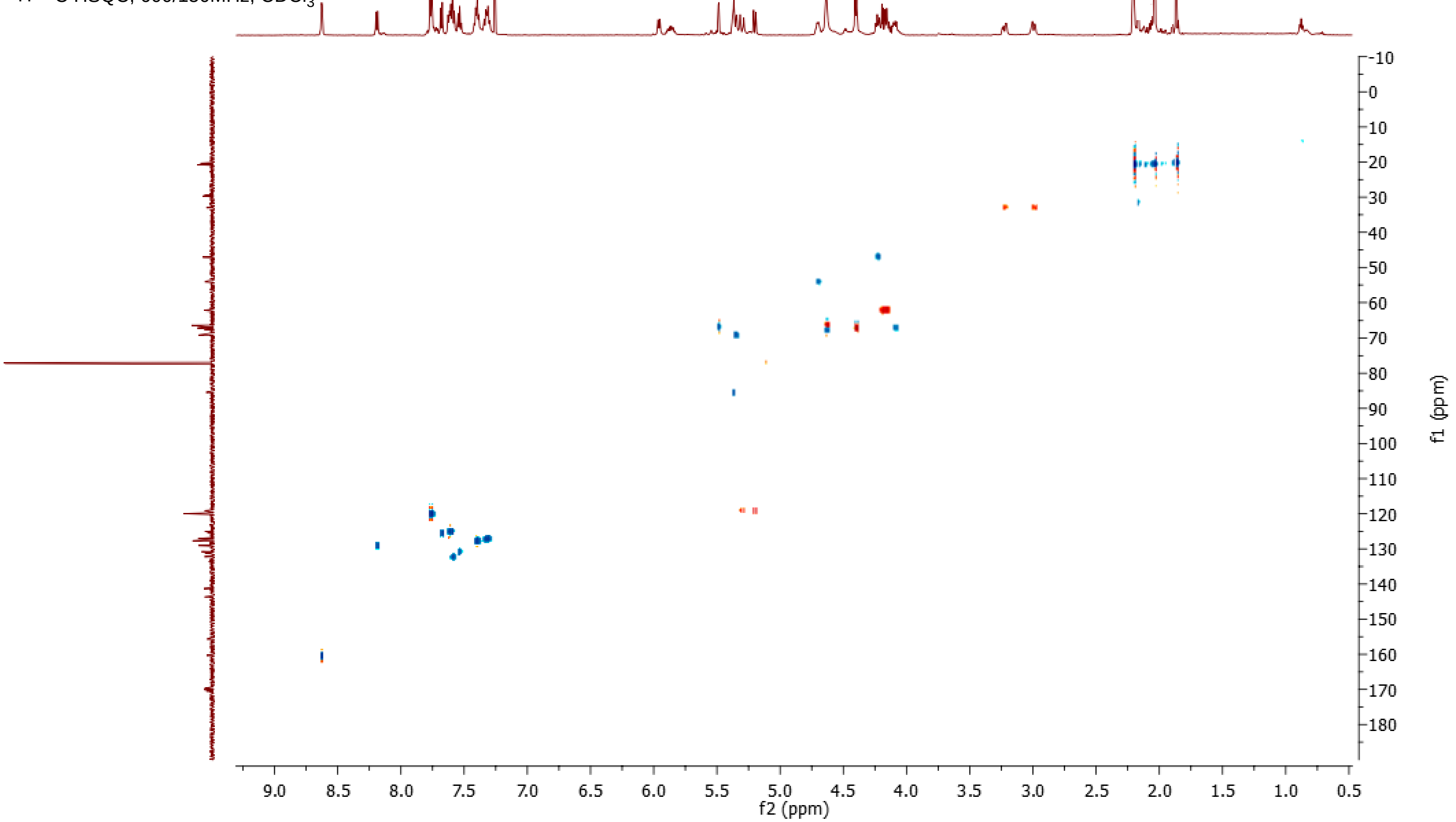

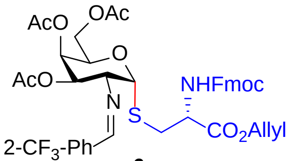

6a

^{1}H-^{13}C Coupled HSQC, 600/150MHz, $CDCl_3$

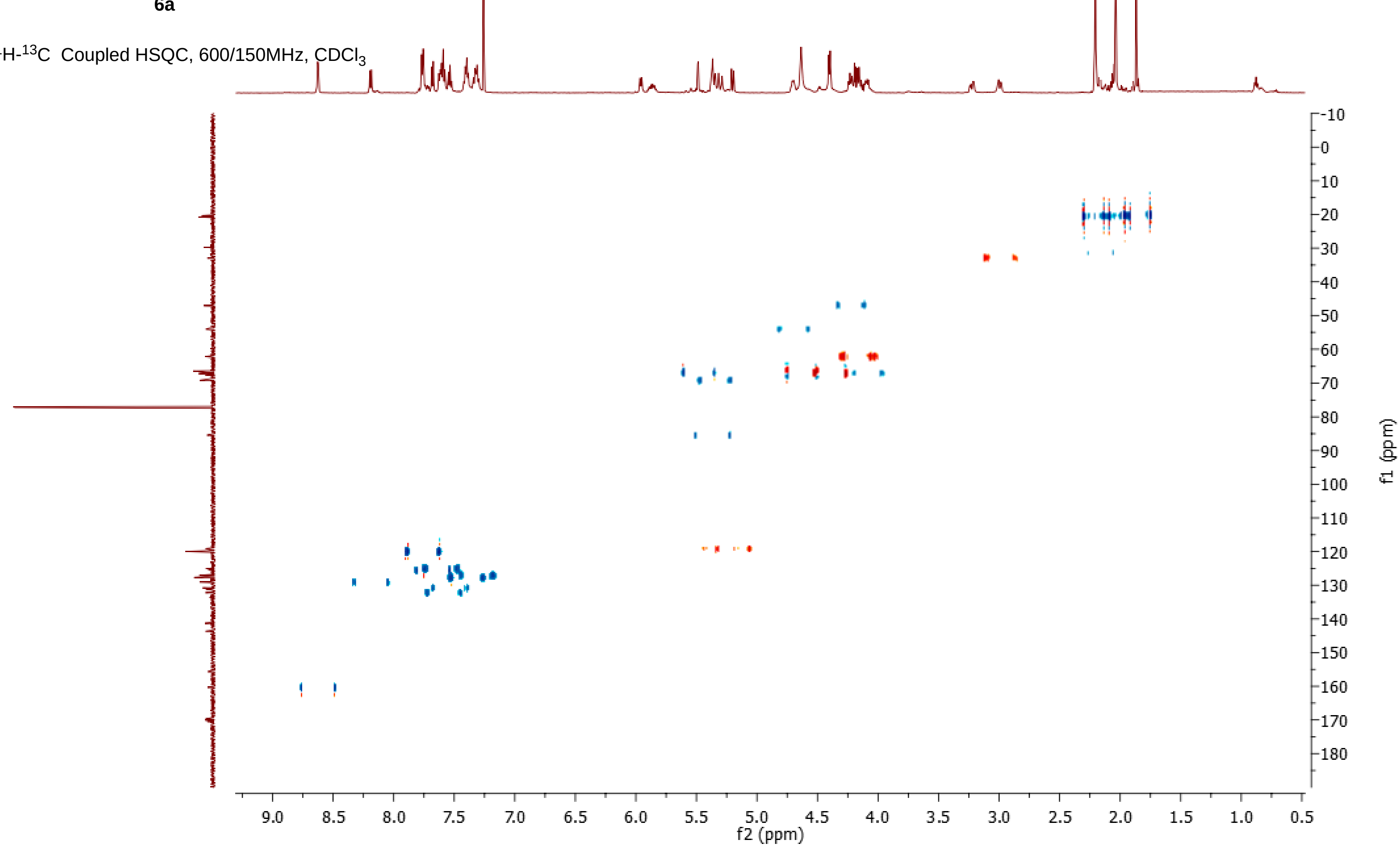

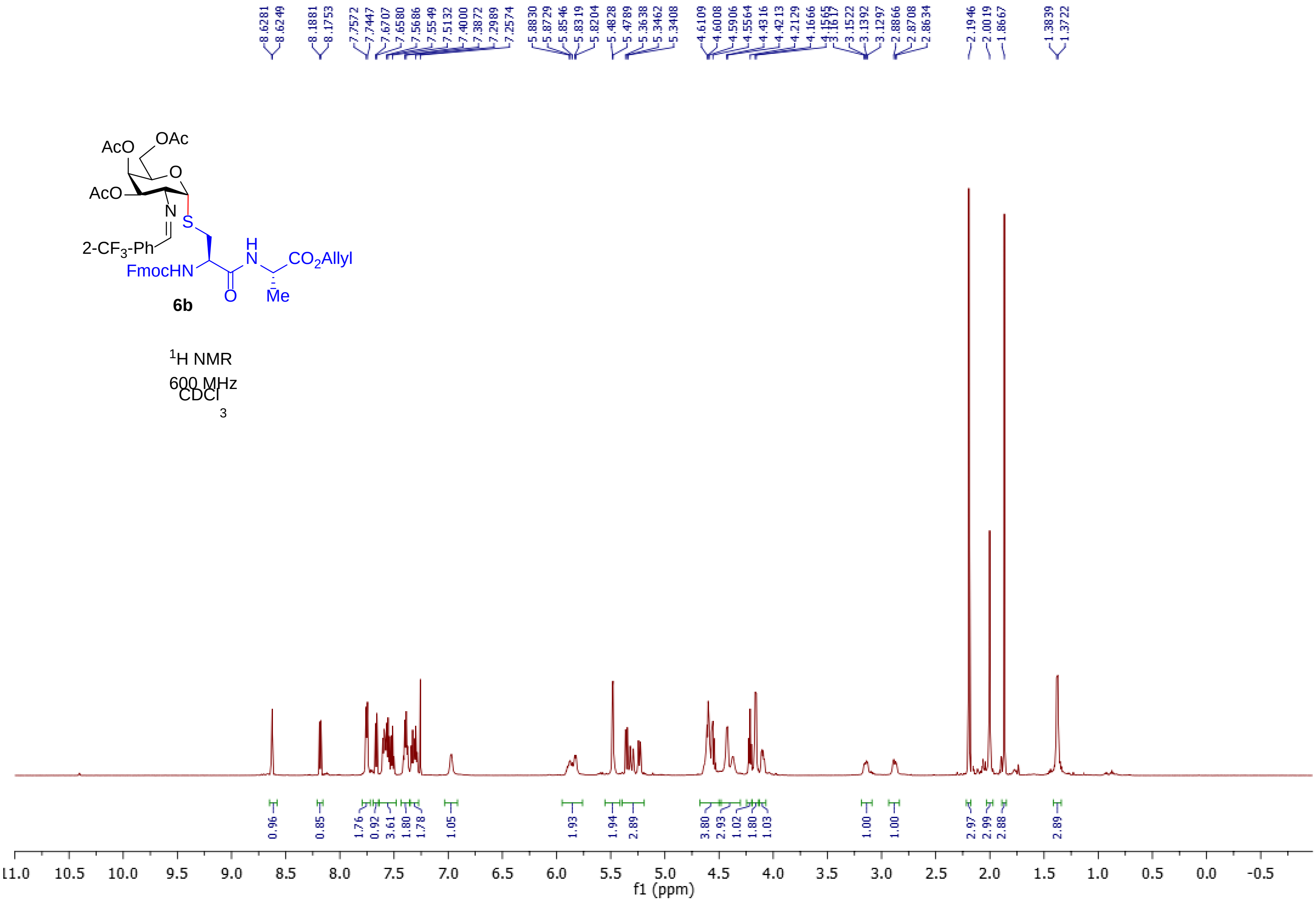
AcO
OAc
O
AcO
N
S
2-CF3-Ph
H
N
CO2Allyl
FmocHN
O
Me
6b
1H NMR
600 MHz
CDCl3
f1 (ppm)

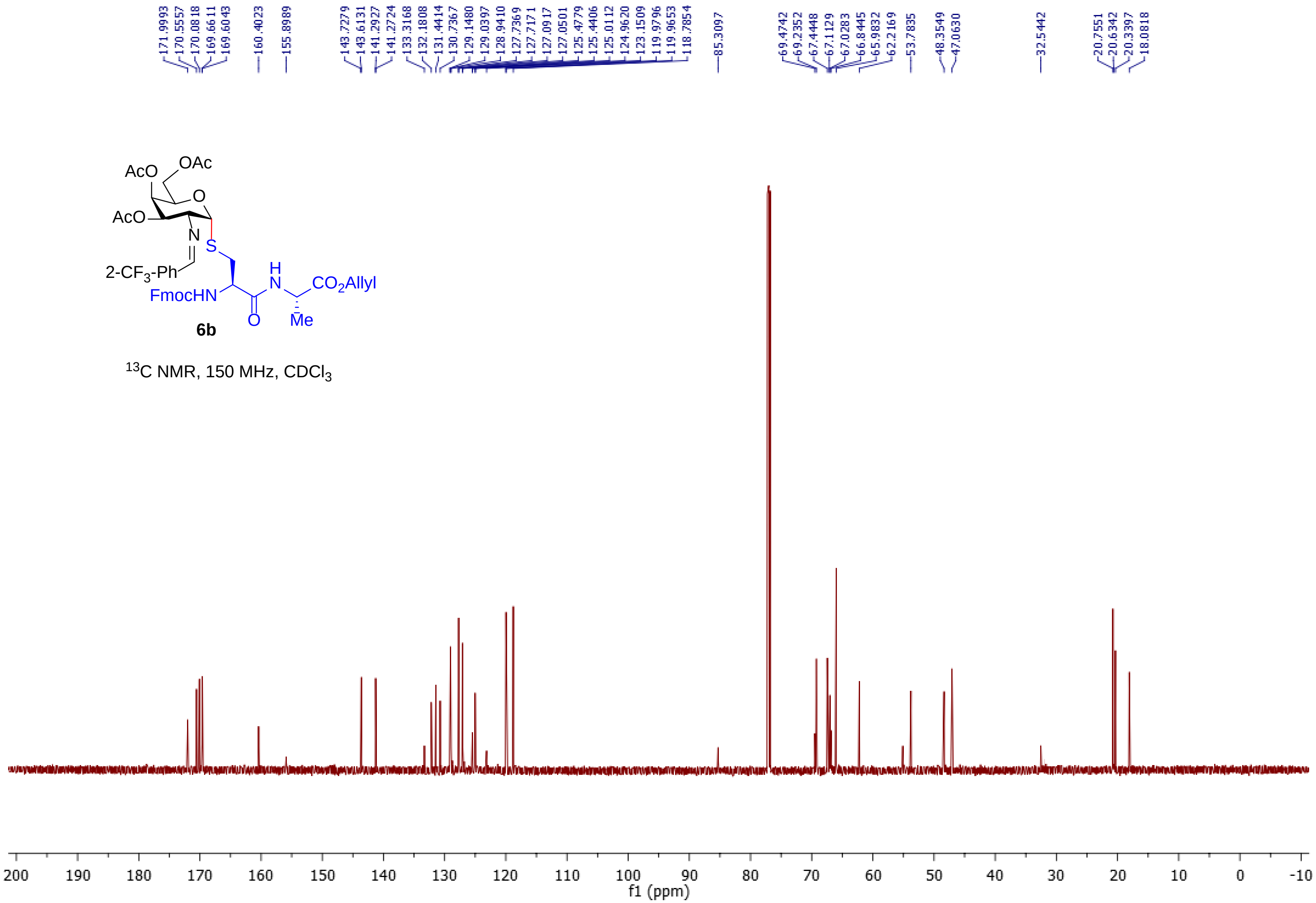

6b

^{13}C NMR, 150 MHz, $CDCl_3$

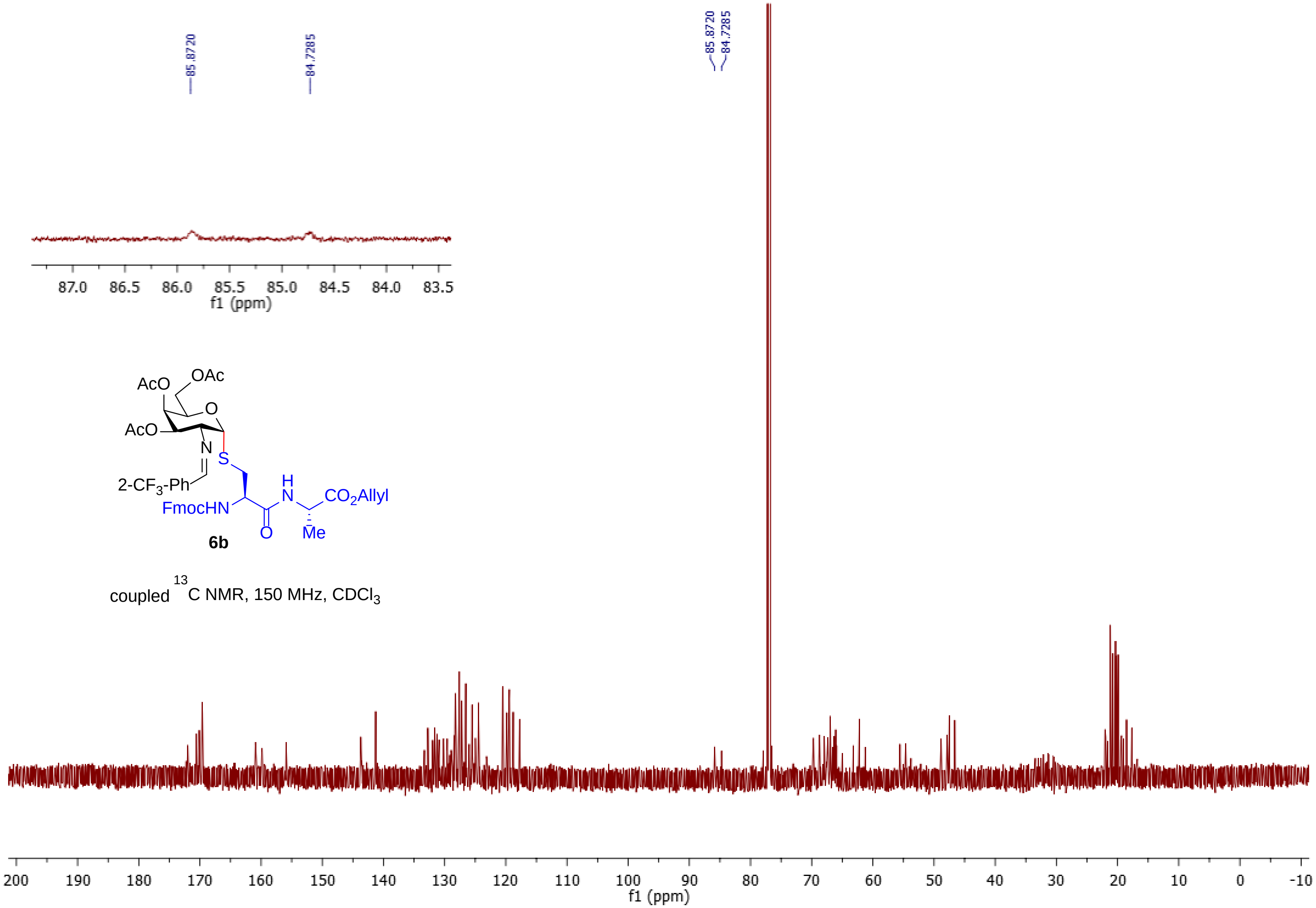

coupled ^{13}C NMR, 150 MHz, $CDCl_3$

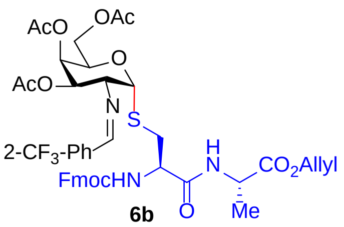

^{1}H-^{13}C HSQC, 600/150MHz, $CDCl_3$

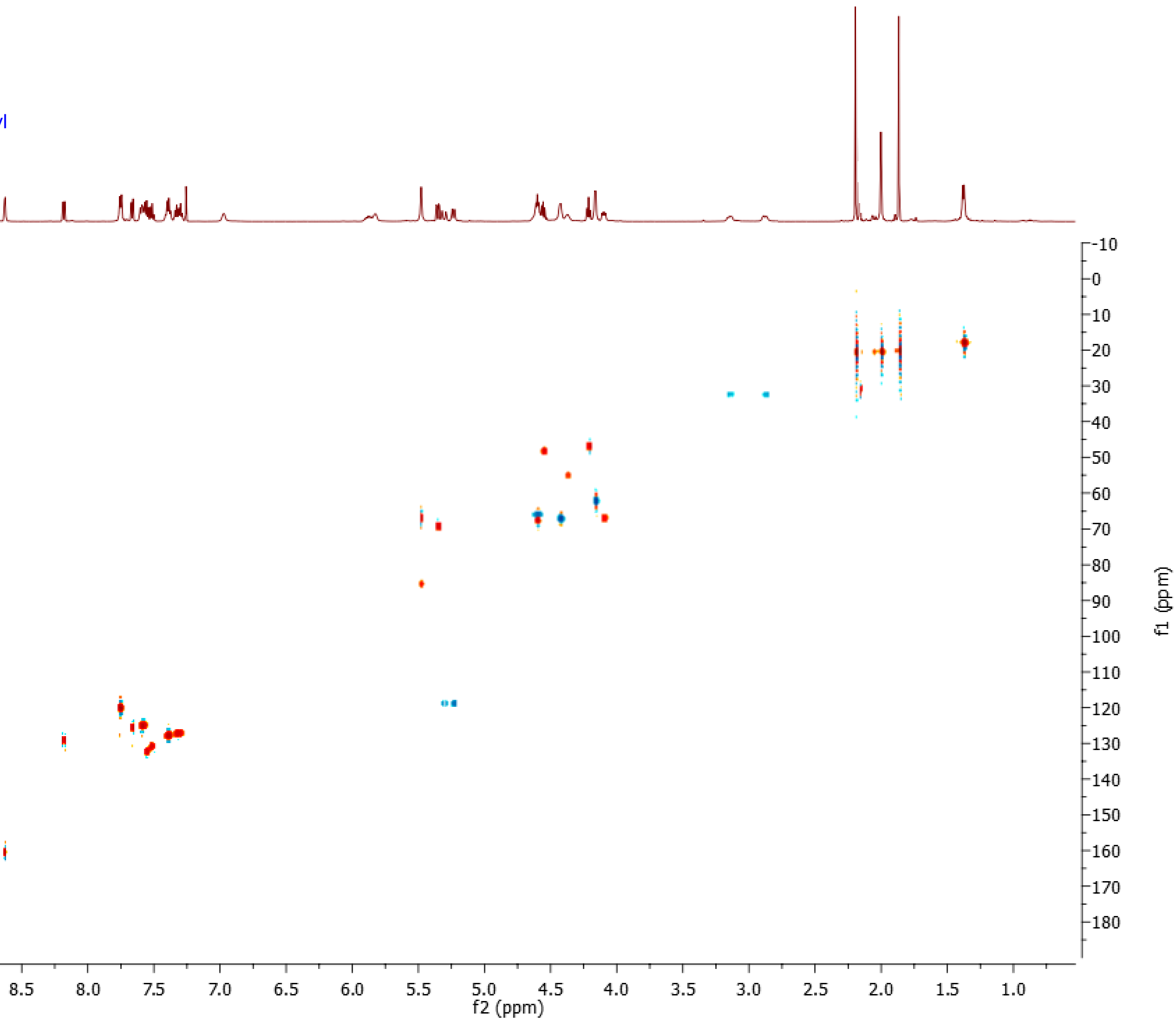

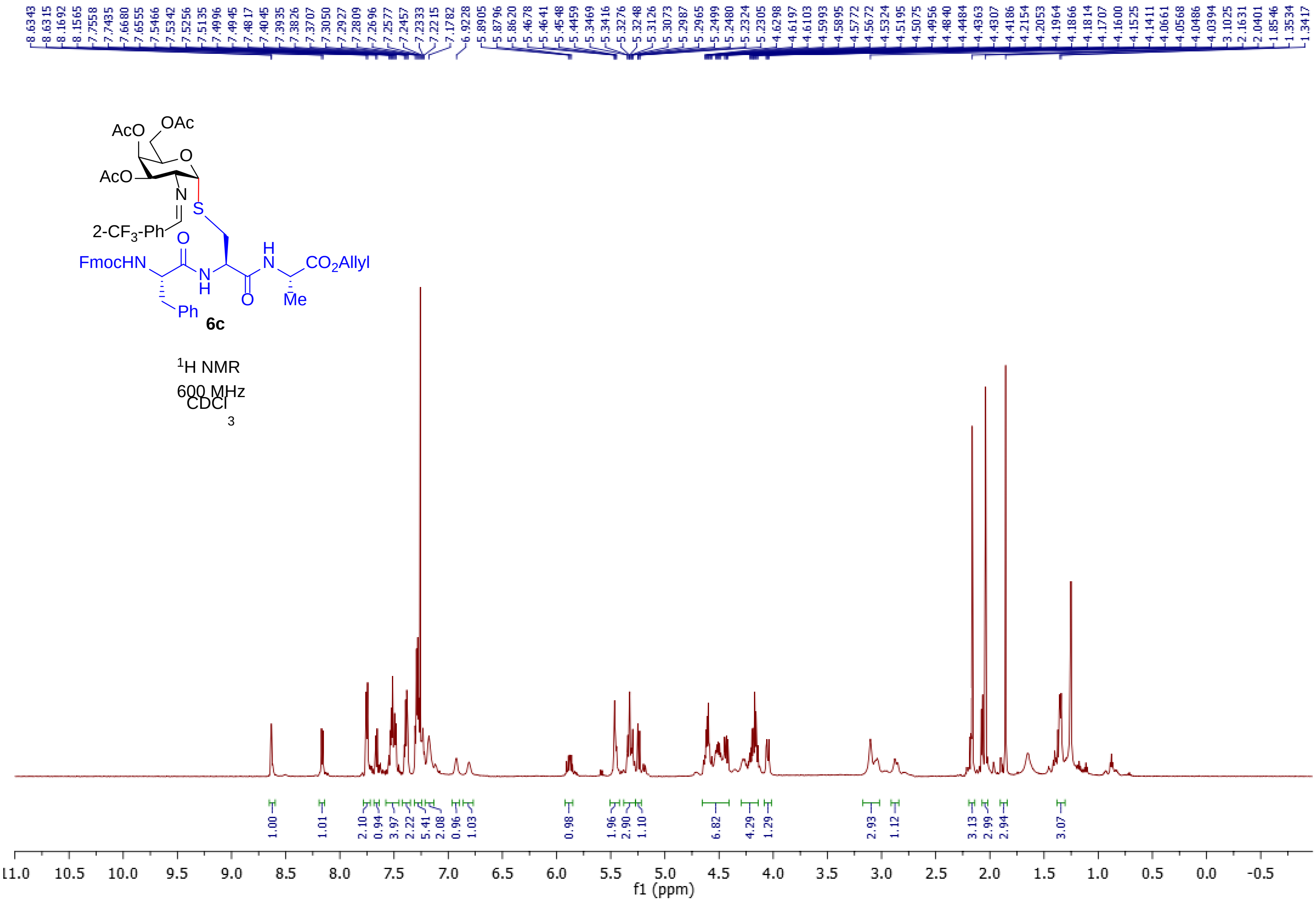
8.6343
8.6315
8.1692
8.1565
7.7558
7.7435
7.6680
7.6555
7.5466
7.5342
7.5256
7.5135
7.4996
7.4945
7.4817
7.4045
7.3935
7.3826
7.3707
7.3050
7.2927
7.2809
7.2696
7.2577
7.2457
7.2333
7.2215
7.1782
6.9228
5.8905
5.8796
5.8620
5.4678
5.4641
5.4548
5.4459
5.3469
5.3416
5.3276
5.3248
5.3126
5.3073
5.2987
5.2965
5.2499
5.2480
5.2324
5.2305
4.6298
4.6197
4.6103
4.5993
4.5895
4.5772
4.5672
4.5324
4.5195
4.5075
4.4956
4.4840
4.4484
4.4363
4.4307
4.4186
4.2154
4.2053
4.1964
4.1866
4.1814
4.1707
4.1600
4.1525
4.1411
4.0661
4.0568
4.0486
4.0394
3.1025
2.1631
2.0401
1.8546
1.3534
1.3417
AcO
OAc
O
AcO
N
S
2-CF3-Ph
O
FmocHN
N
H
H
N
CO2Allyl
O
Me
Ph
6c
1H NMR
600 MHz
CDCl3
1.00
1.01
2.10
0.94
3.97
2.22
5.41
2.08
0.96
1.03
0.98
1.96
2.90
1.10
6.82
4.29
1.29
2.93
1.12
3.13
2.99
2.94
3.07
11.0
10.5
10.0
9.5
9.0
8.5
8.0
7.5
7.0
6.5
6.0
5.5
5.0
4.5
4.0
3.5
3.0
2.5
2.0
1.5
1.0
0.5
0.0
-0.5
f1 (ppm)

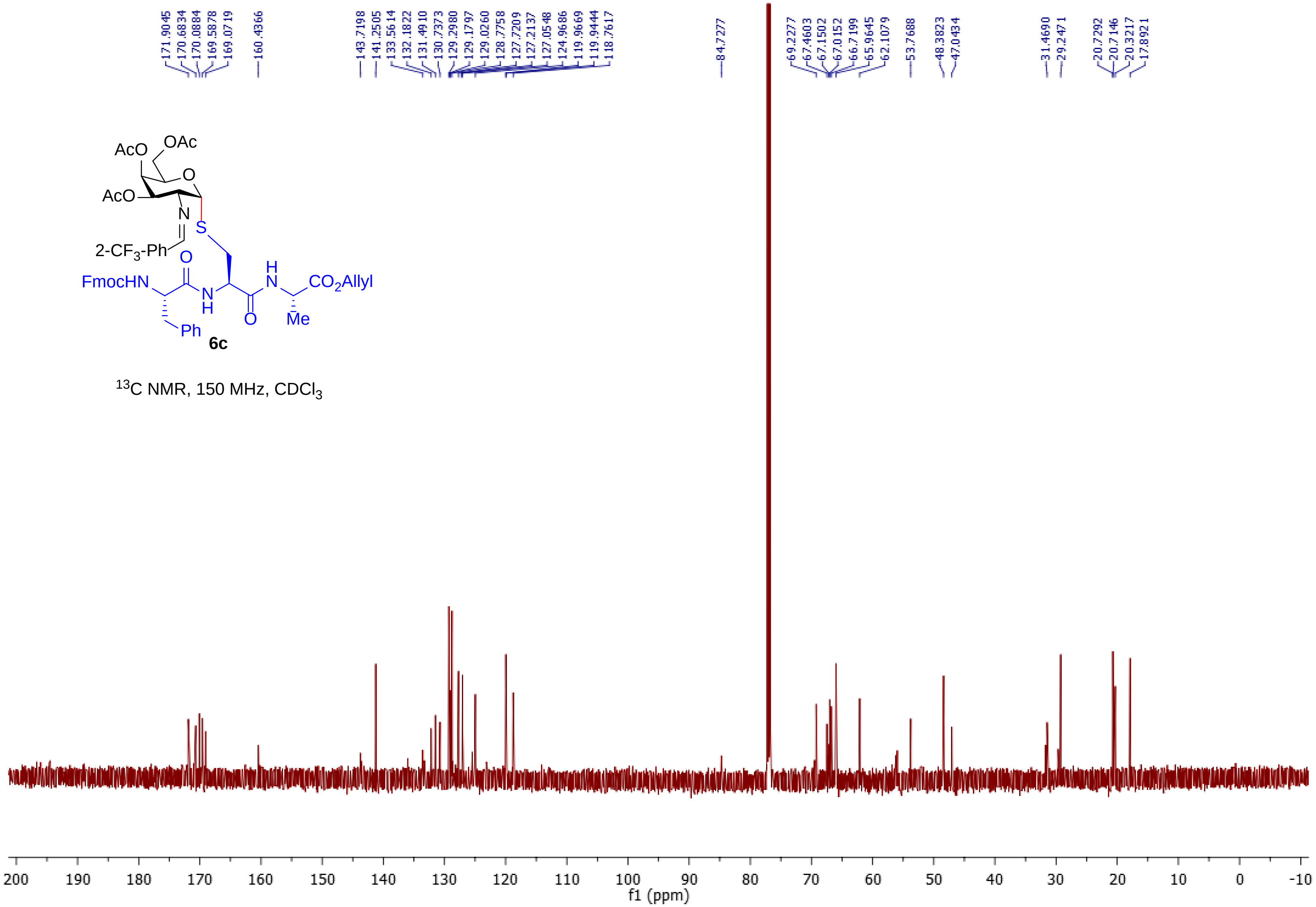

6c

^{13}C NMR, 150 MHz, $CDCl_3$

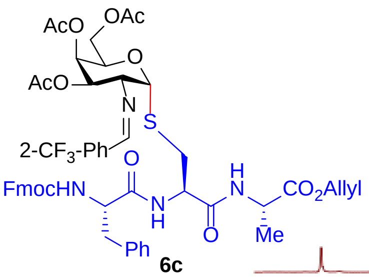
AcO
OAc
O
AcO
N
S
2-CF3-Ph
O
FmocHN
N
H
H
N
CO2Allyl
O
Me
Ph

6c

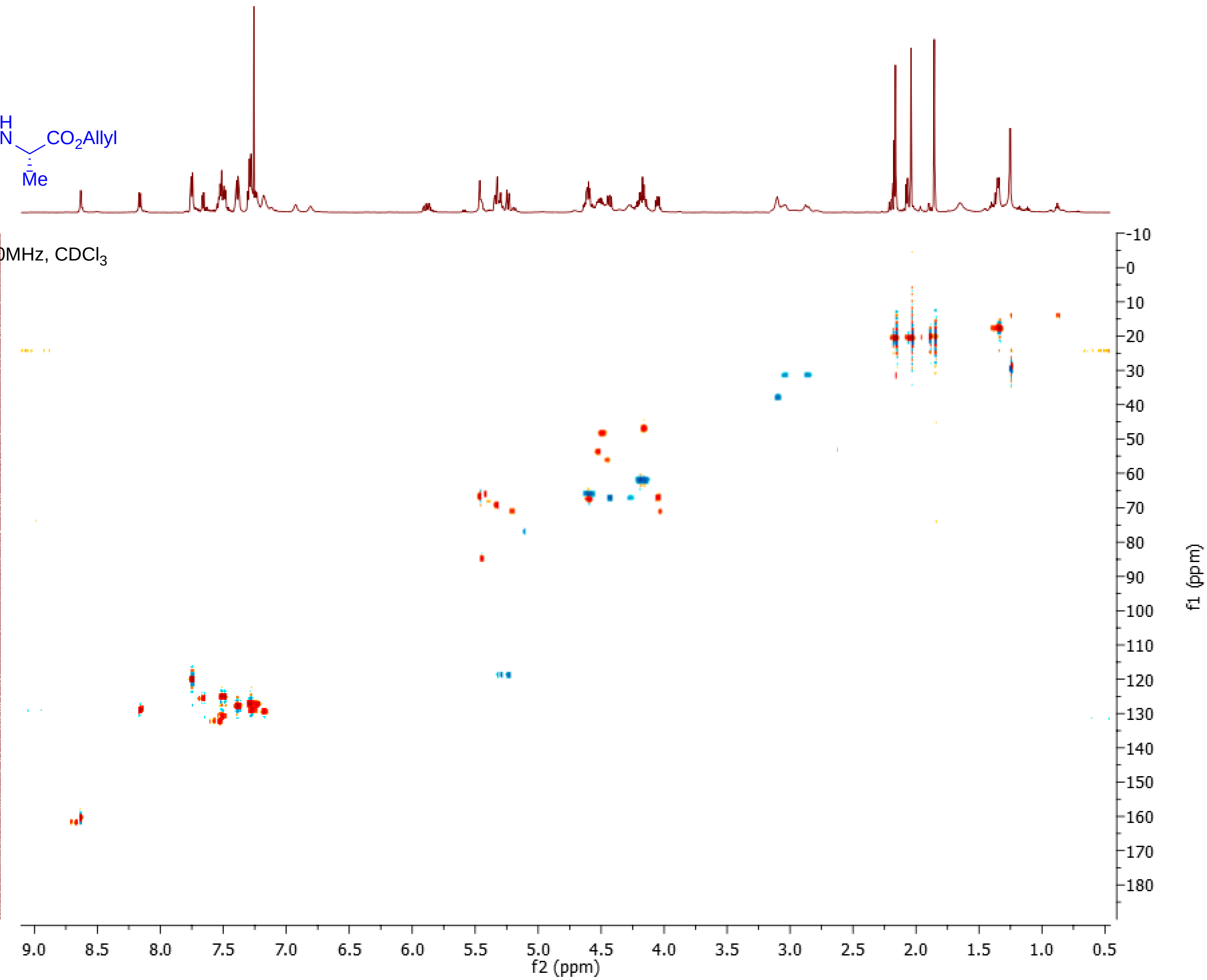
1H-13C HSQC, 600/150MHz, CDCl3
f2 (ppm)
f1 (ppm)

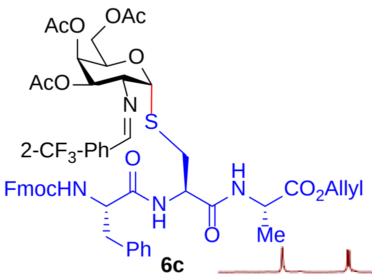
AcO
OAc
O
AcO
N
S
2-CF3-Ph
O
FmocHN
N
H
O
H
N
CO2Allyl
Me
Ph

6c

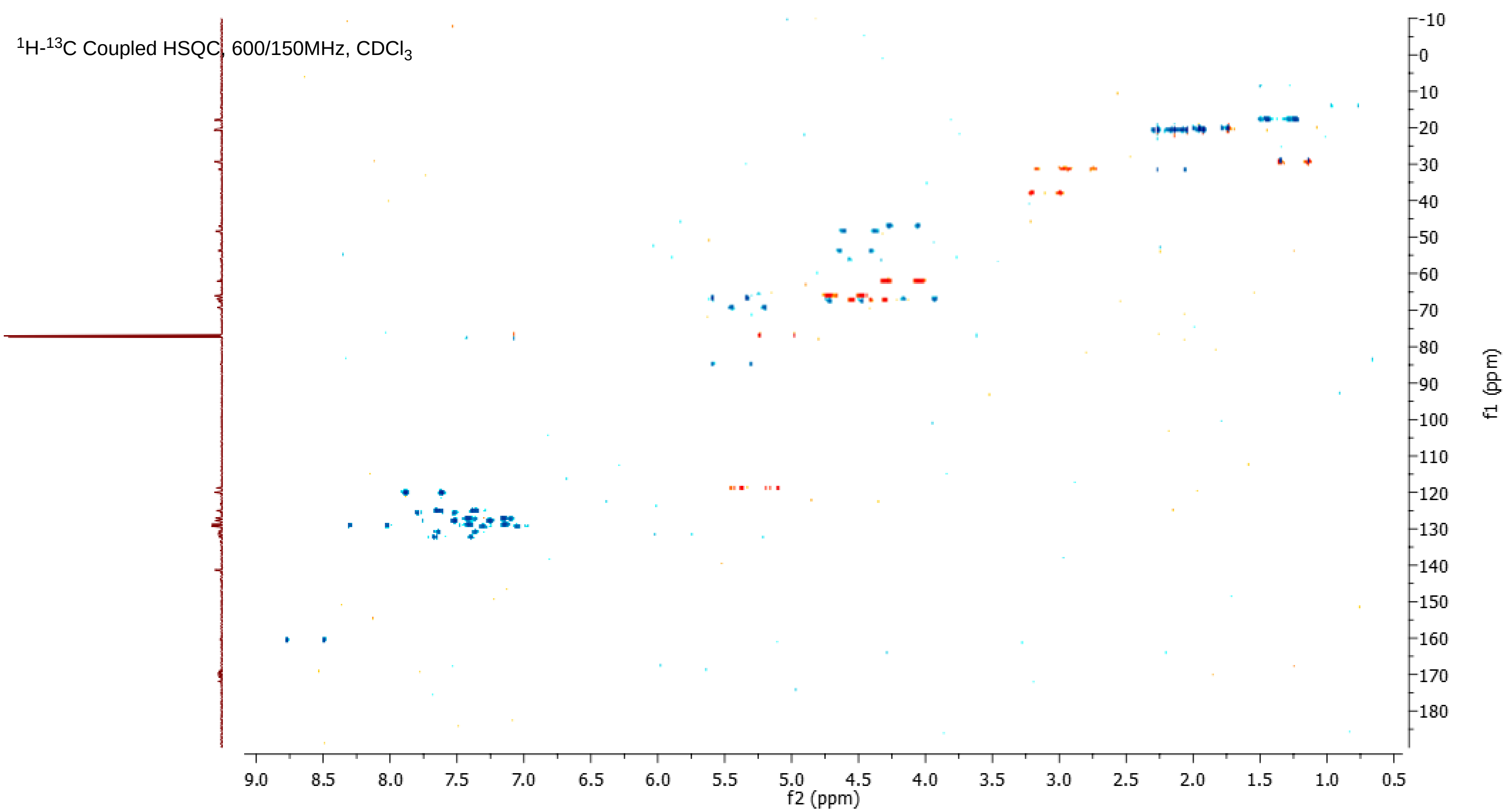
1H-13C Coupled HSQC, 600/150MHz, CDCl3
f2 (ppm)
f1 (ppm)
9.0
8.5
8.0
7.5
7.0
6.5
6.0
5.5
5.0
4.5
4.0
3.5
3.0
2.5
2.0
1.5
1.0
0.5
-10
0
10
20
30
40
50
60
70
80
90
100
110
120
130
140
150
160
170
180

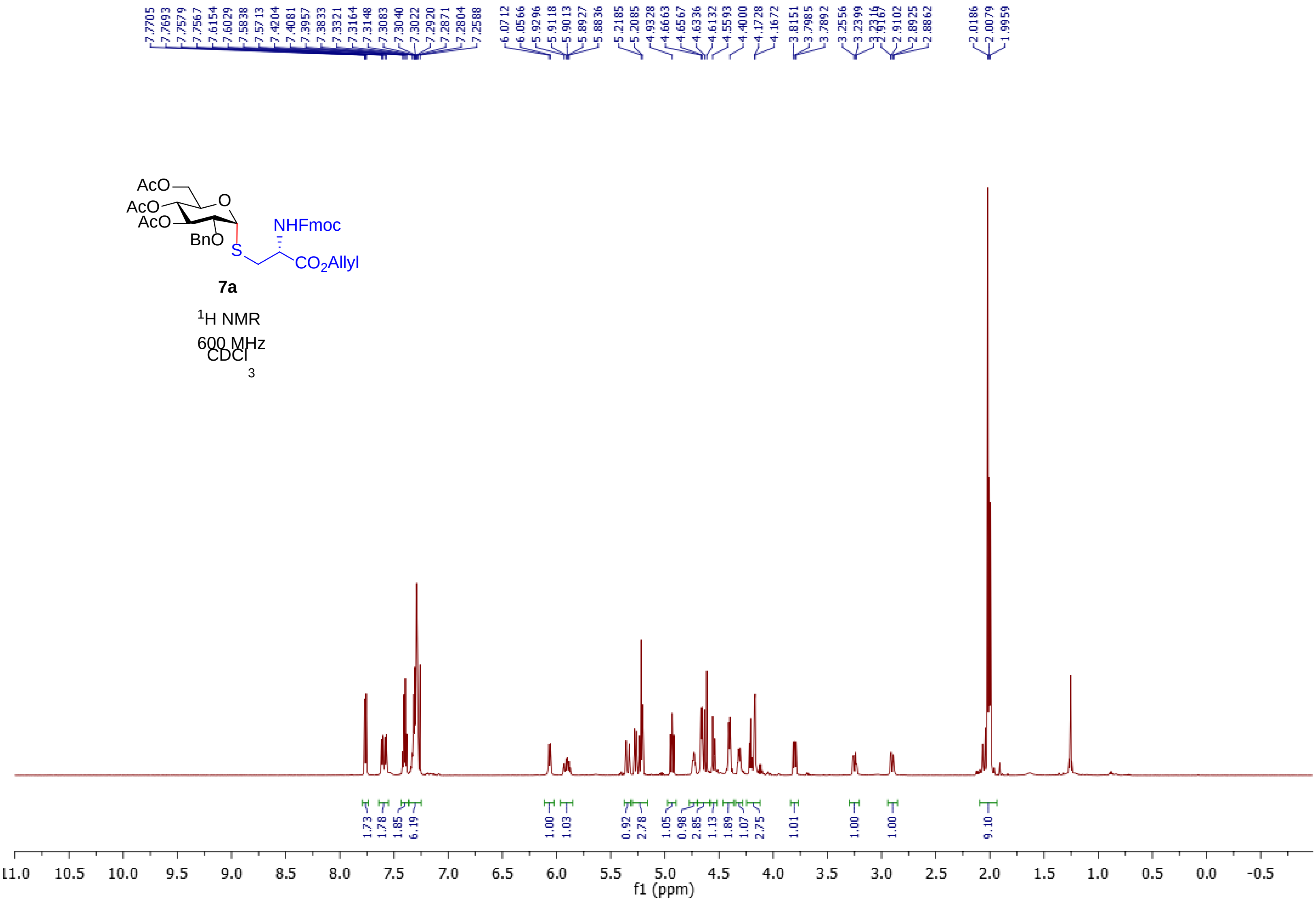

7a

^{1}H NMR
600 MHz
$CDCl_3$

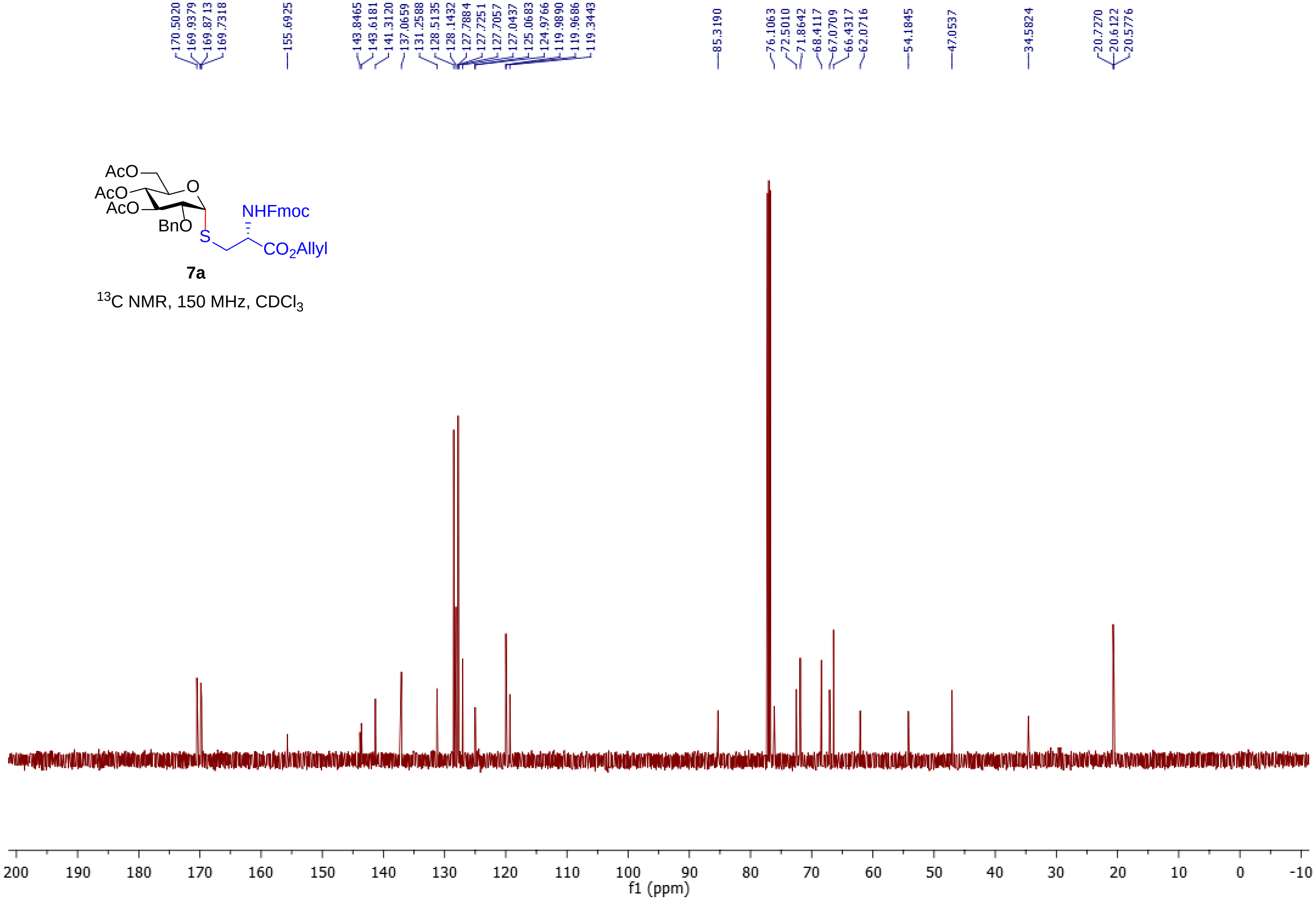
170.5020
169.9379
169.8713
169.7318
155.6925
143.8465
143.6181
141.3120
137.0659
131.2588
128.5135
128.1432
127.7884
127.7251
127.7057
127.0437
125.0683
124.9766
119.9890
119.9686
119.3443
85.3190
76.1063
72.5010
71.8642
68.4117
67.0709
66.4317
62.0716
54.1845
47.0537
34.5824
20.7270
20.6122
20.5776
AcO
AcO
AcO
O
BnO
S
NHFmoc
CO_2Allyl
7a
^{13}C NMR, 150 MHz, $CDCl_3$
200 190 180 170 160 150 140 130 120 110 100 90 80 70 60 50 40 30 20 10 0 -10
f1 (ppm)

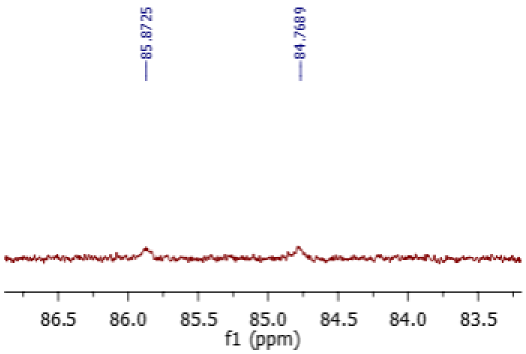

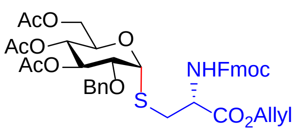

7a

coupled ^{13}C NMR, 150 MHz, $CDCl_3$

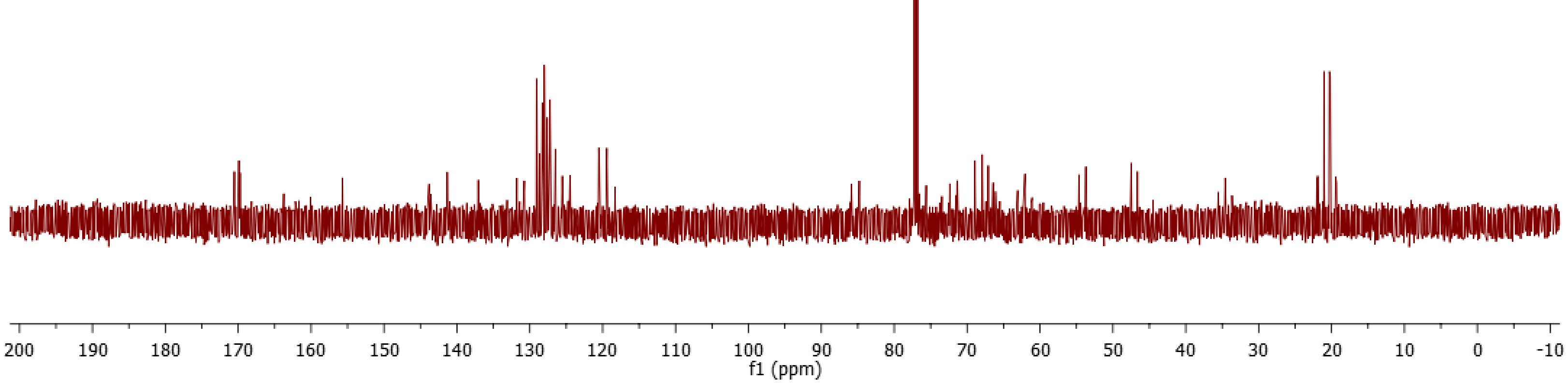

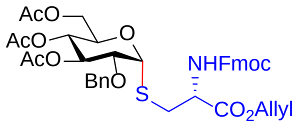

7a

^{1}H-^{1}H COSY, 600 MHz, $CDCl_3$

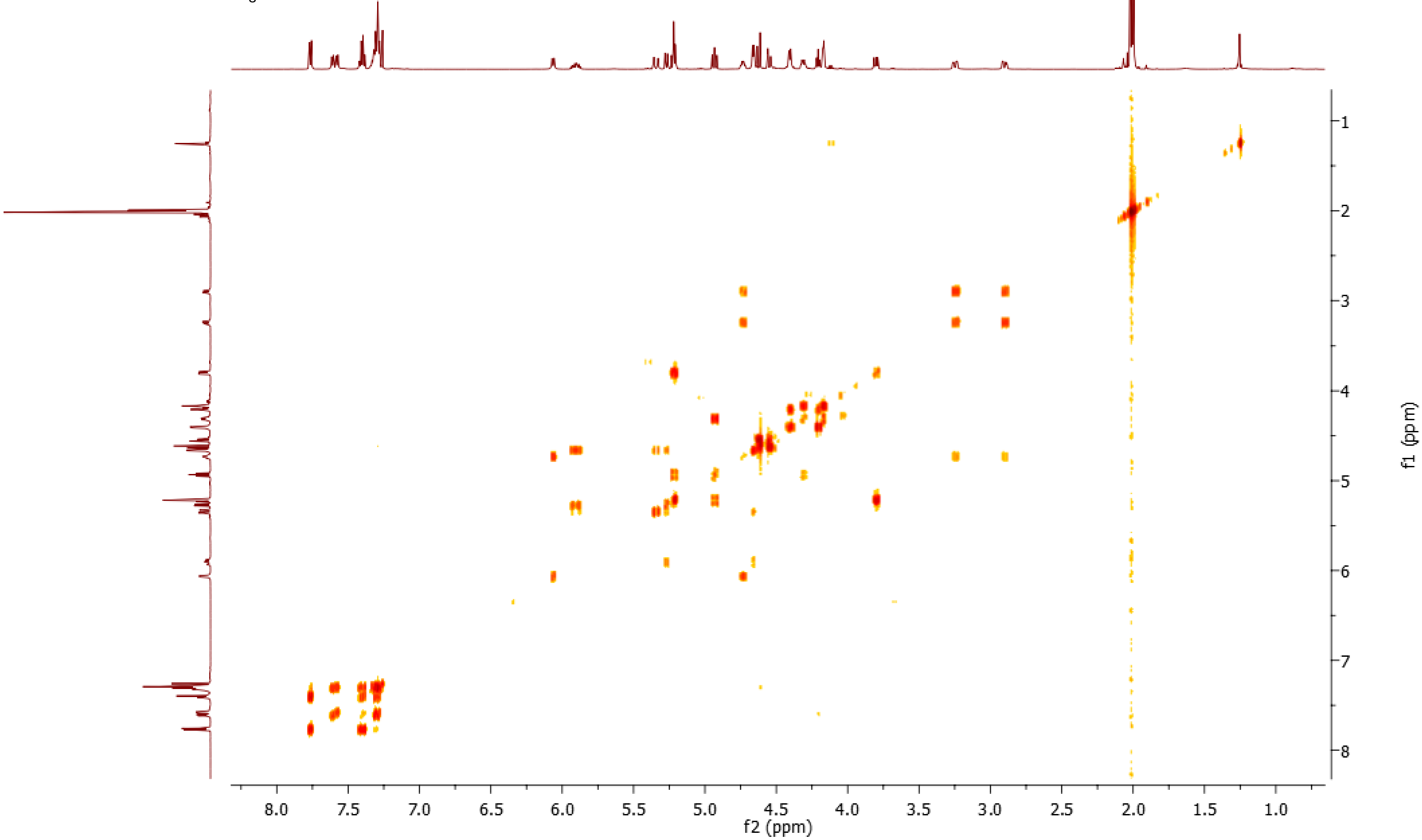

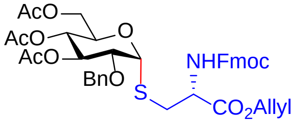

7a

^{1}H-^{13}C HSQC, 600/150MHz, $CDCl_3$

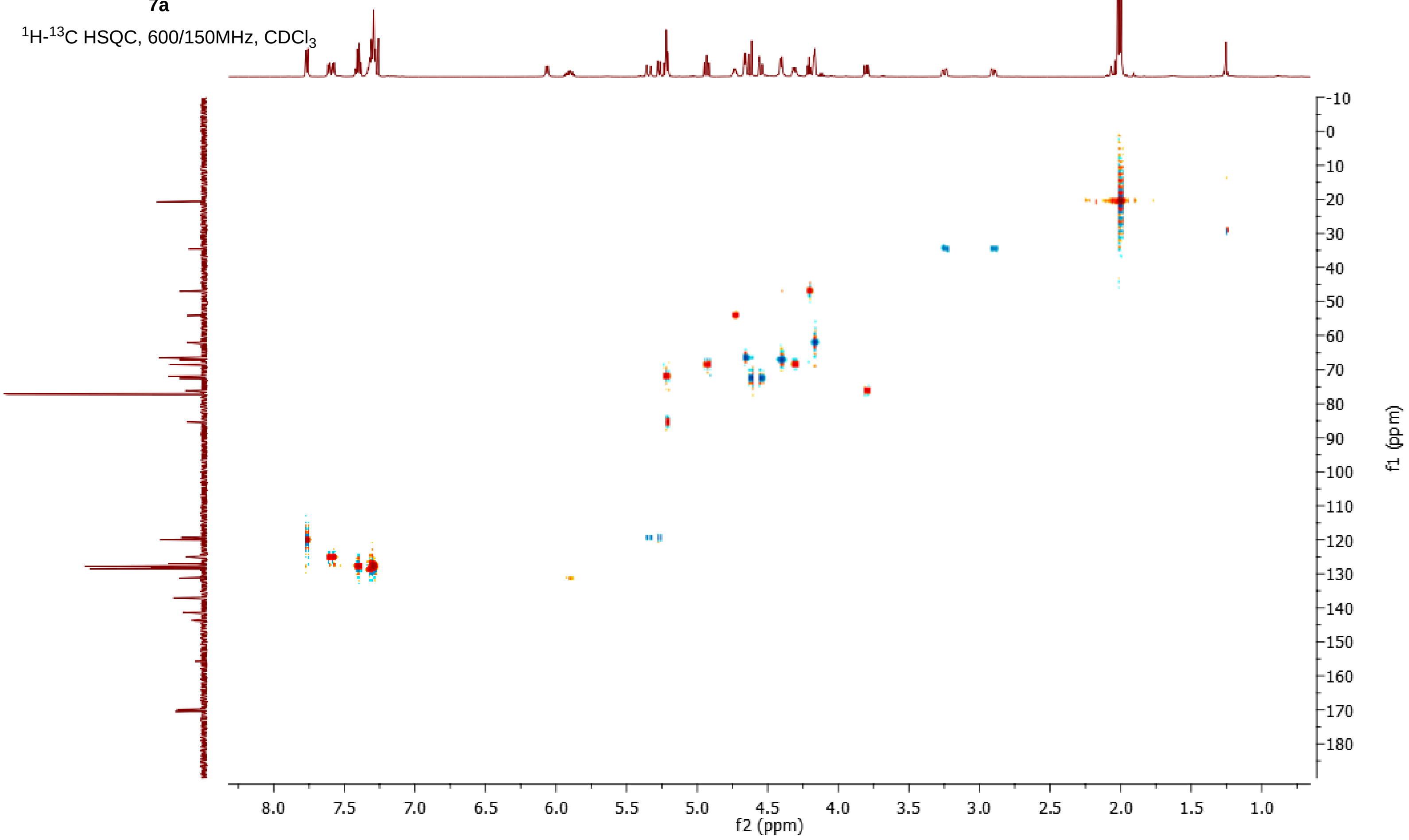

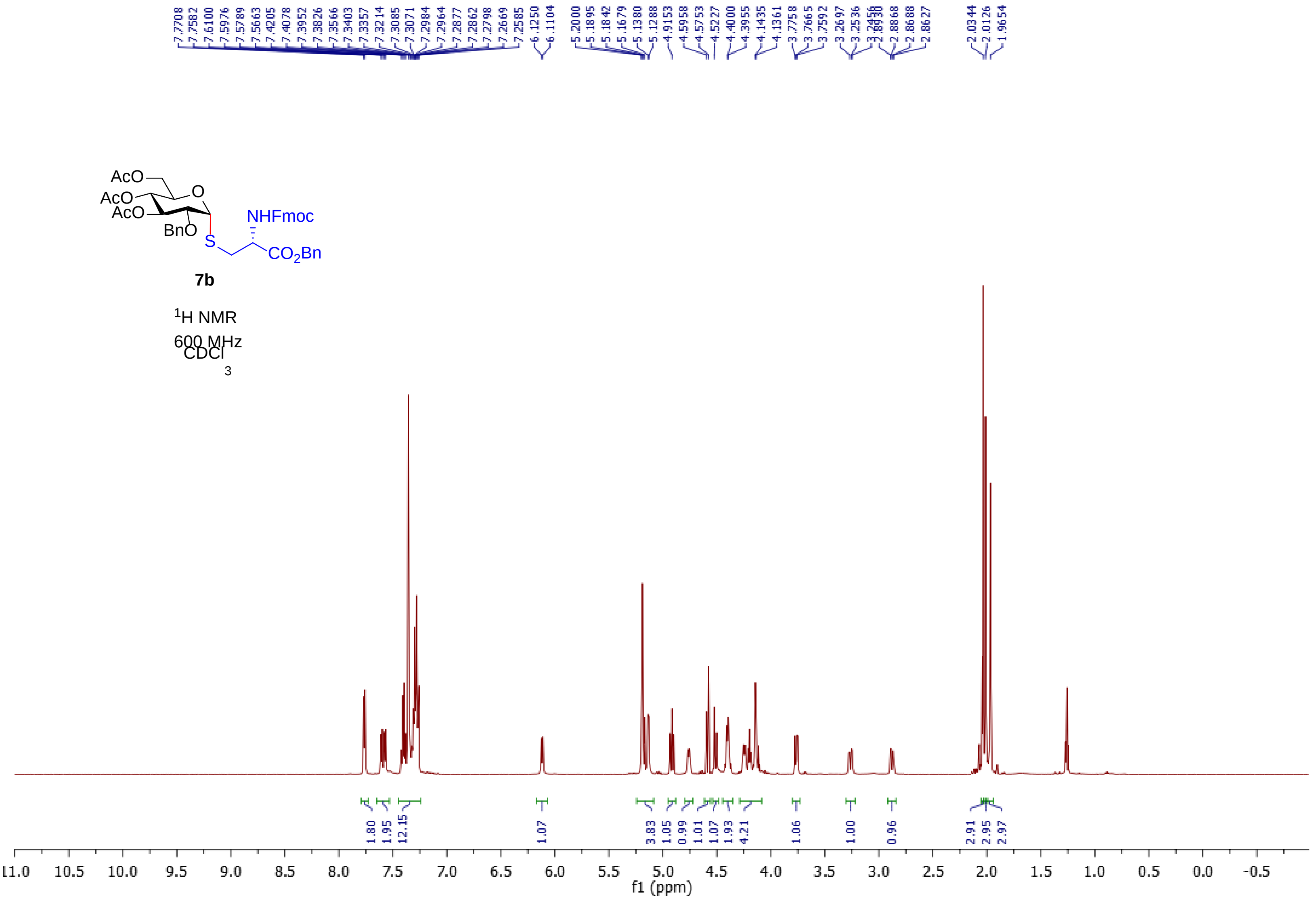
AcO
AcO
AcO
O
BnO
S
NHFmoc
CO2Bn
7b
1H NMR
600 MHz
CDCl3
1.80
1.95
12.15
1.07
3.83
1.05
0.99
1.01
1.07
1.93
4.21
1.06
1.00
0.96
2.91
2.95
2.97
f1 (ppm)

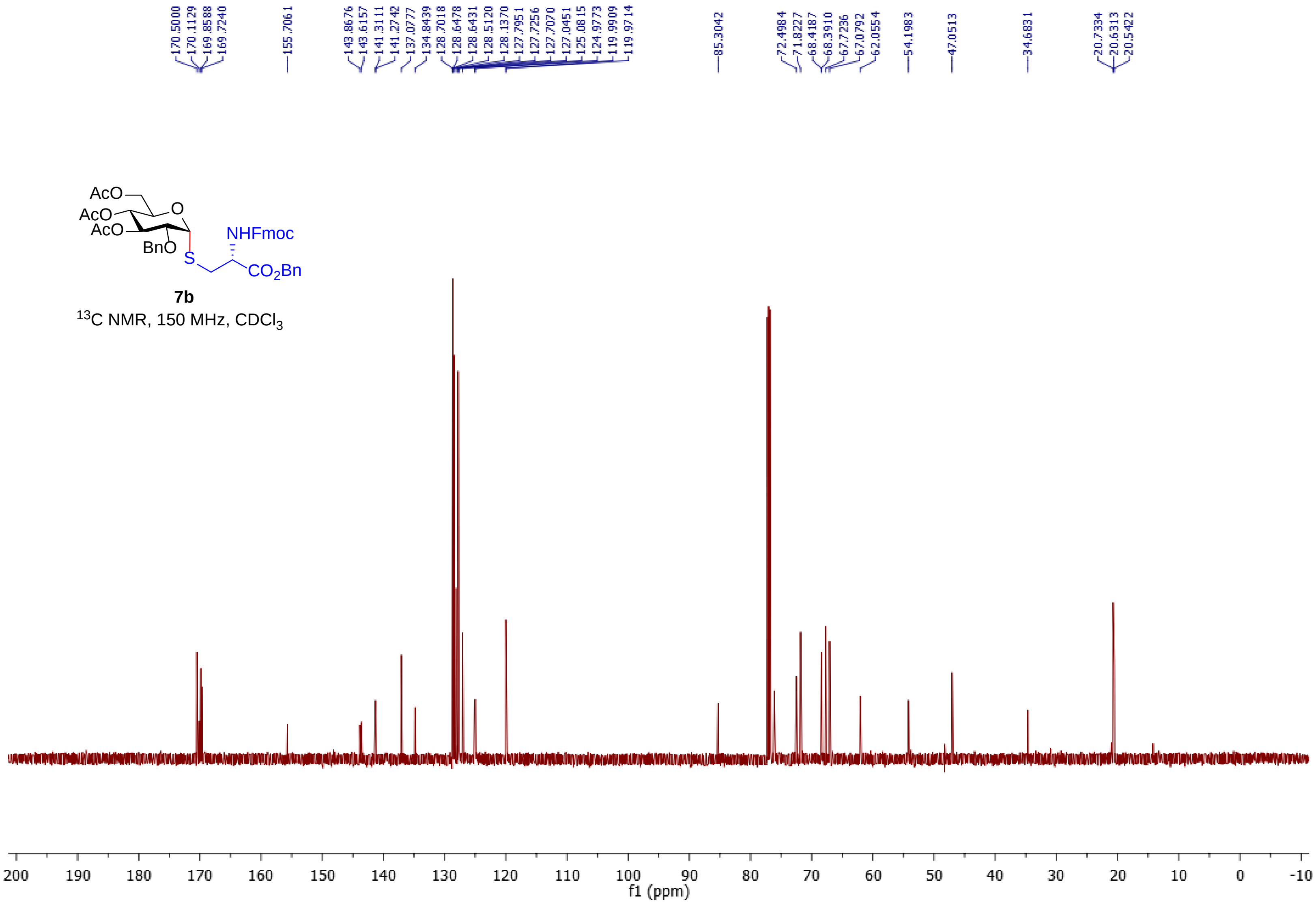

7b

^{13}C NMR, 150 MHz, $CDCl_3$

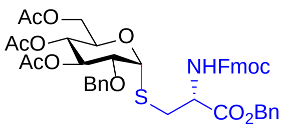

7b

^1H-^1H COSY, 600 MHz, $CDCl_3$

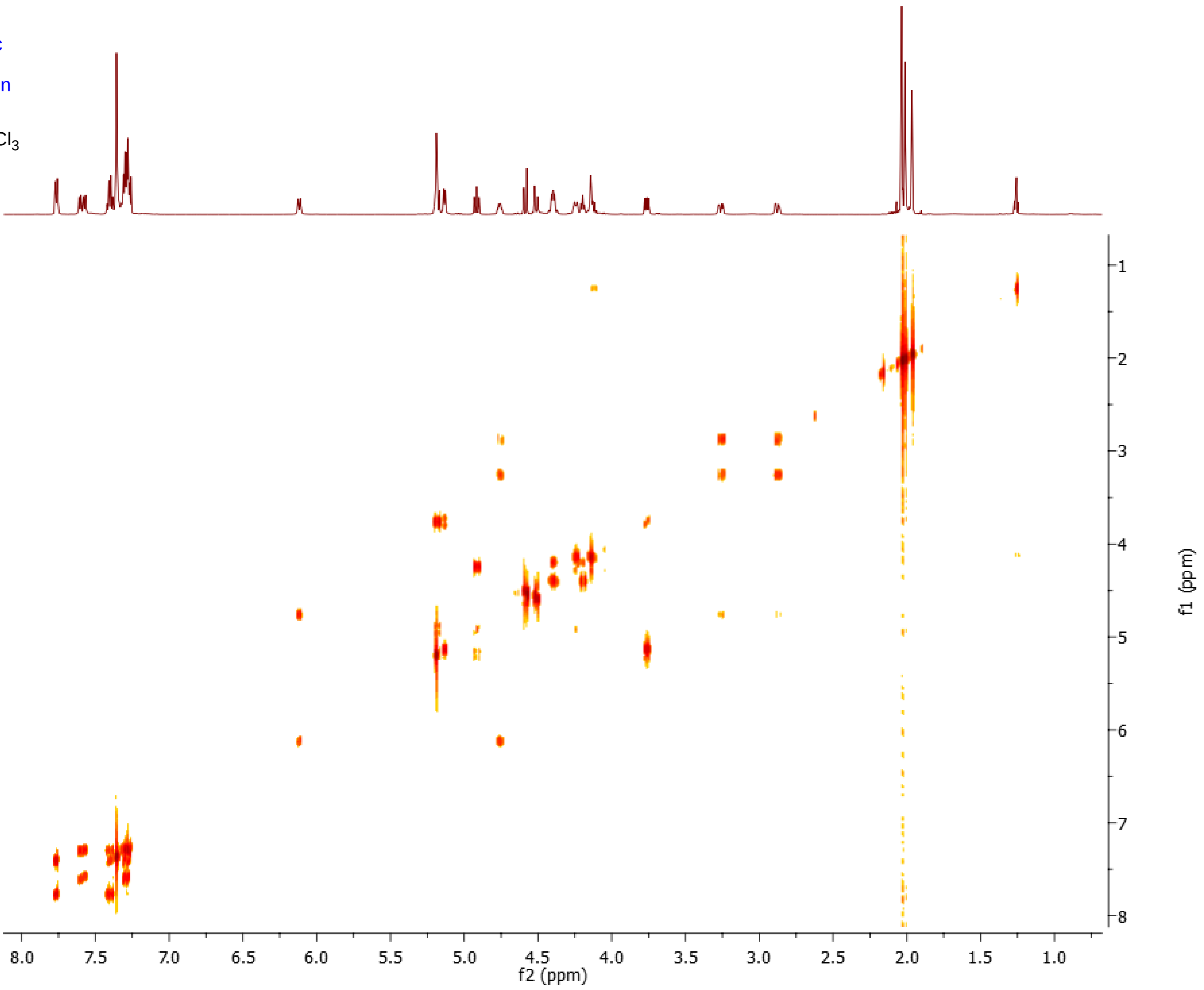

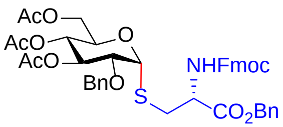

7b

^{1}H-^{13}C HSQC, 600/150MHz, $CDCl_3$

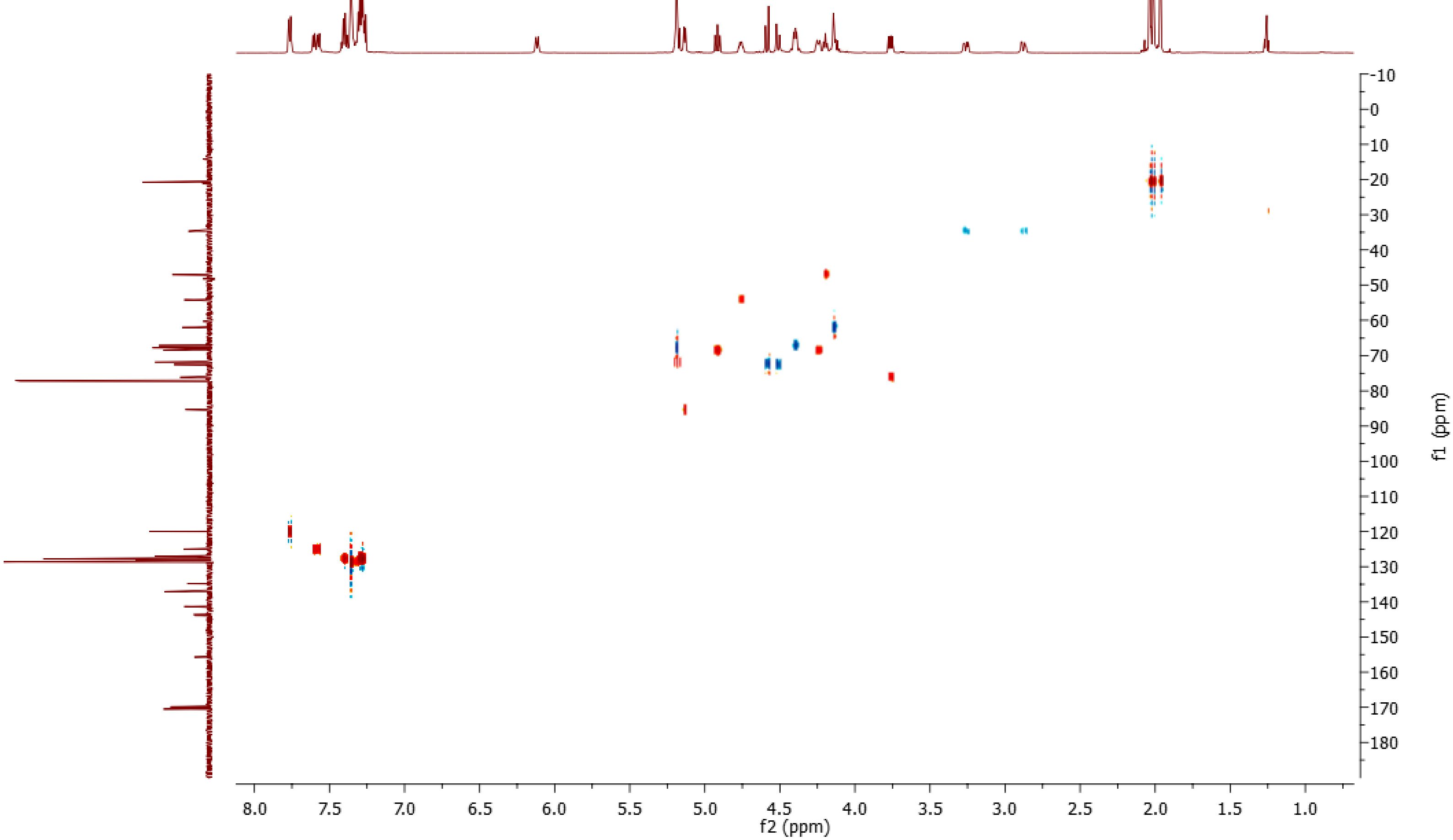

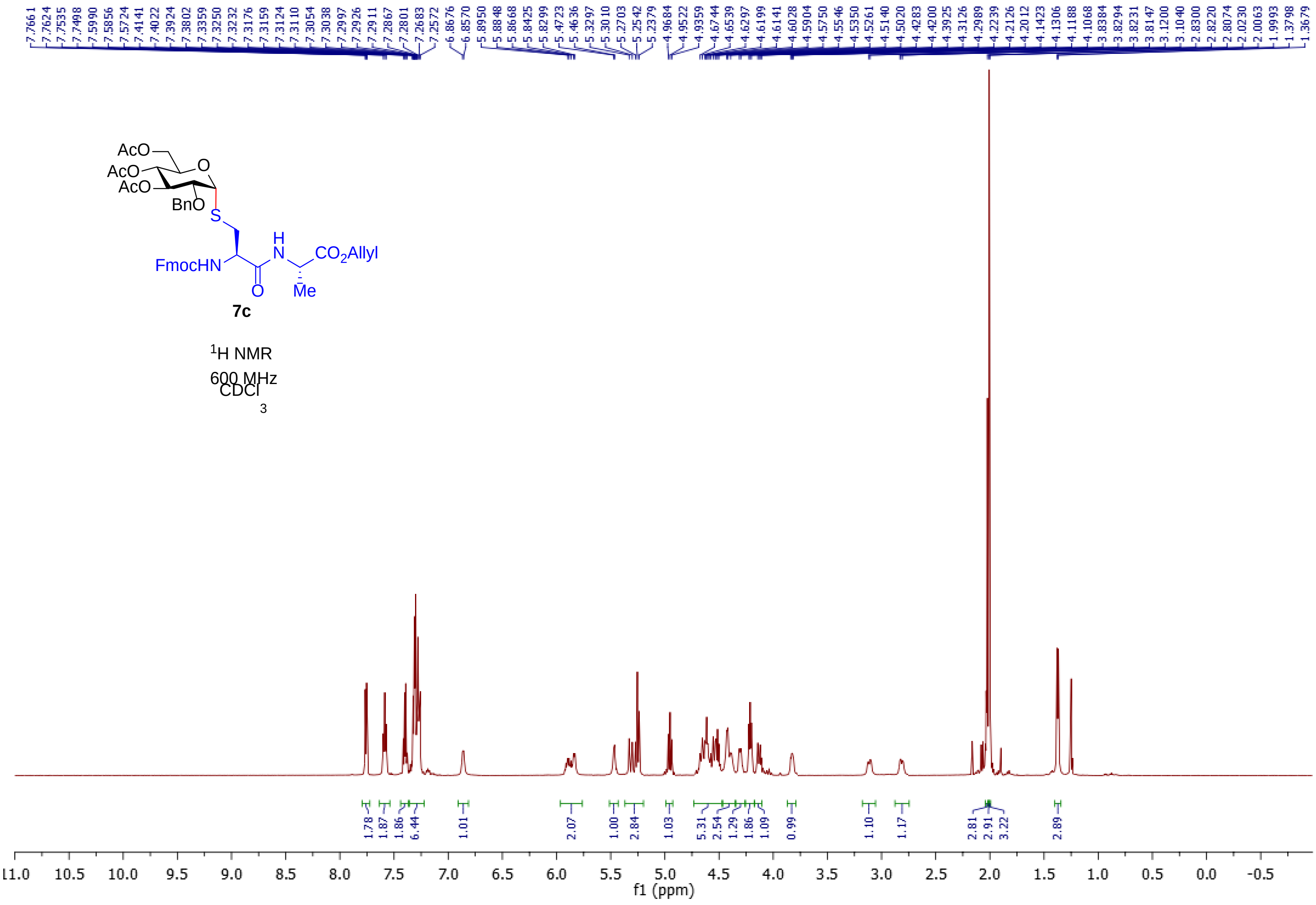
AcO
AcO
AcO
O
BnO
S
H
N
FmocHN
O
CO2Allyl
Me
7c
1H NMR
600 MHz
CDCl3
f1 (ppm)

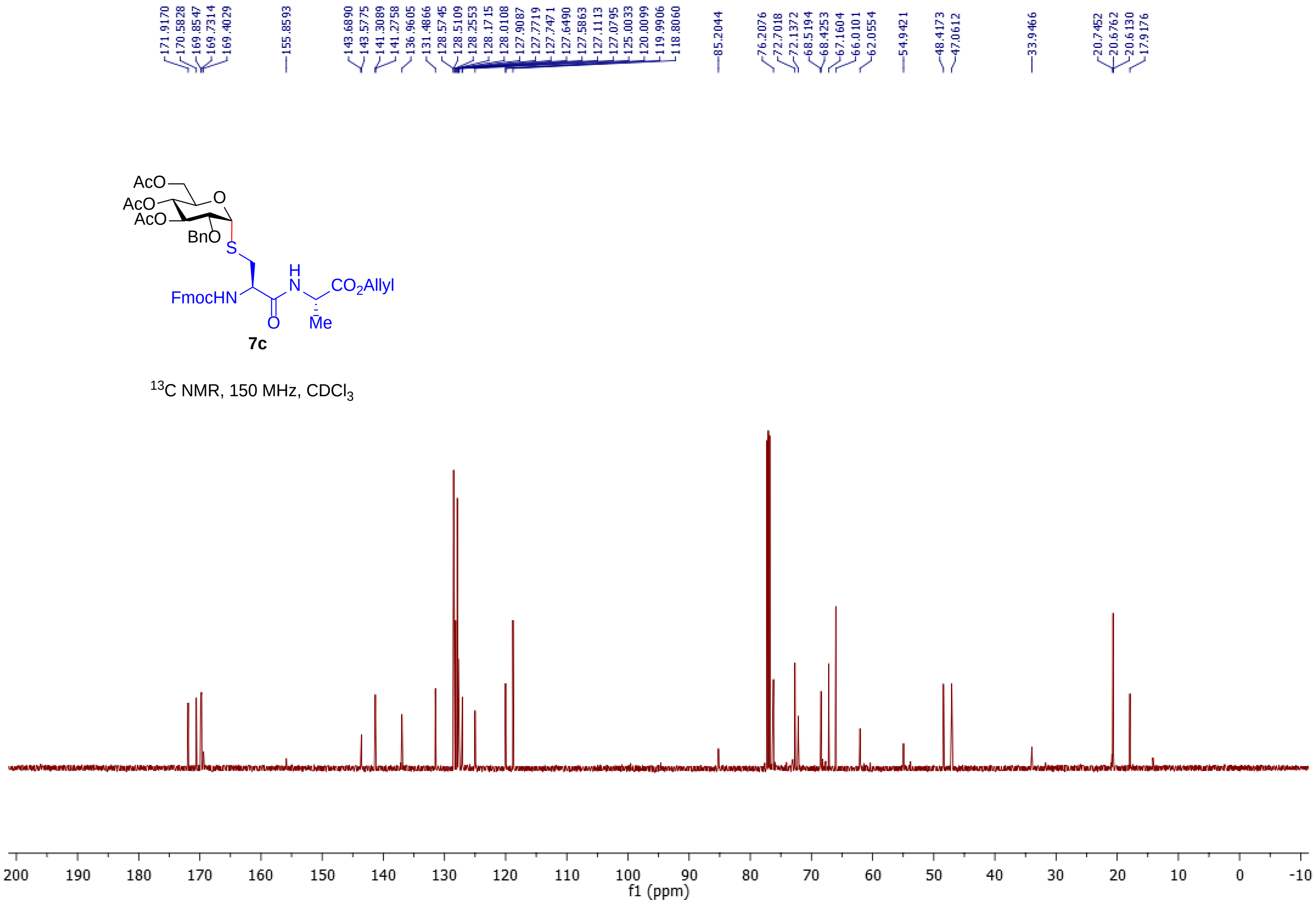

7c

^{13}C NMR, 150 MHz, $CDCl_3$

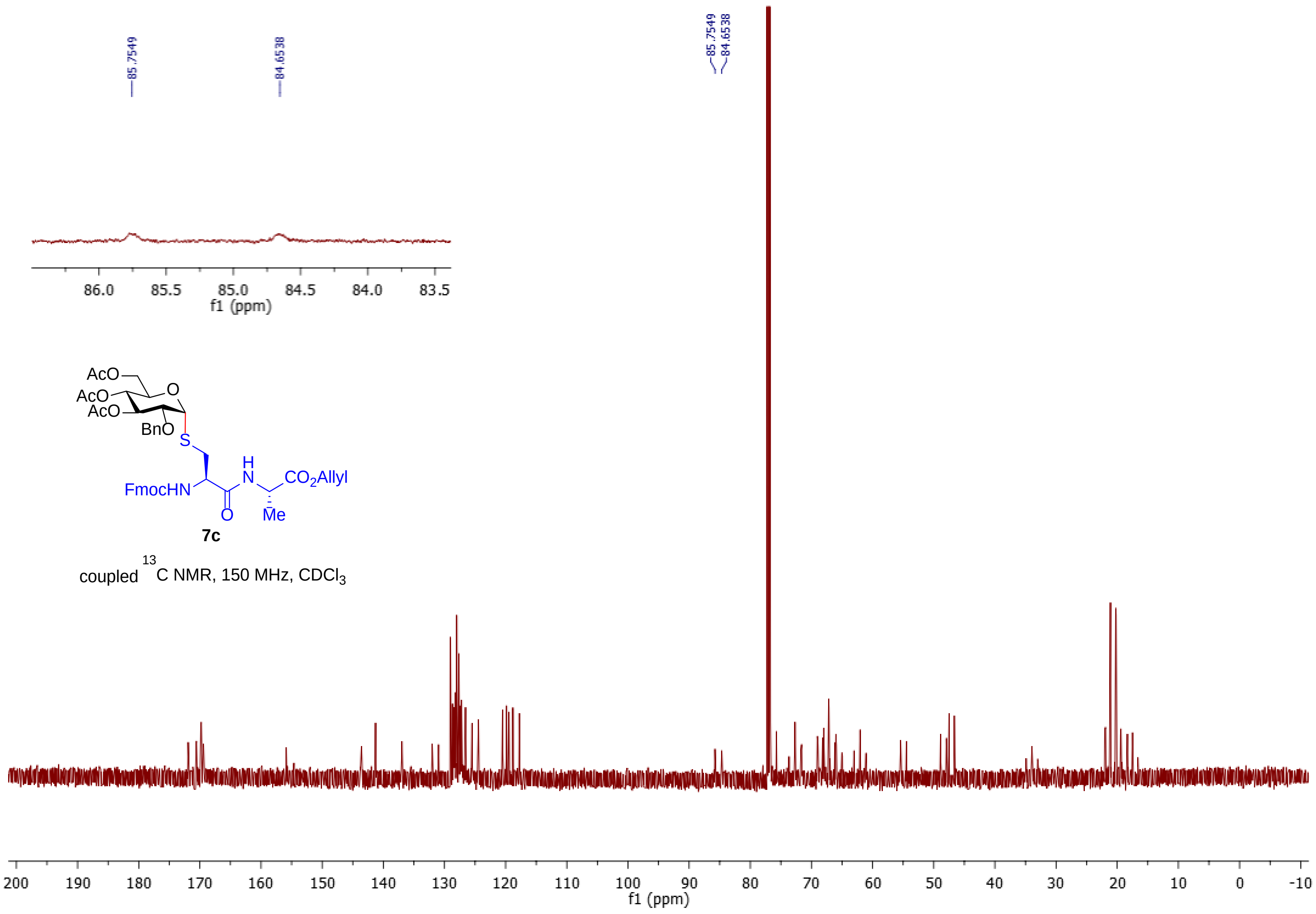

7c

coupled ^{13}C NMR, 150 MHz, $CDCl_3$

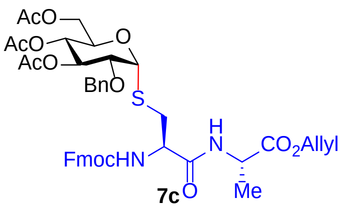

7c

^{1}H-^{13}C HSQC, 600/150MHz, $CDCl_3$

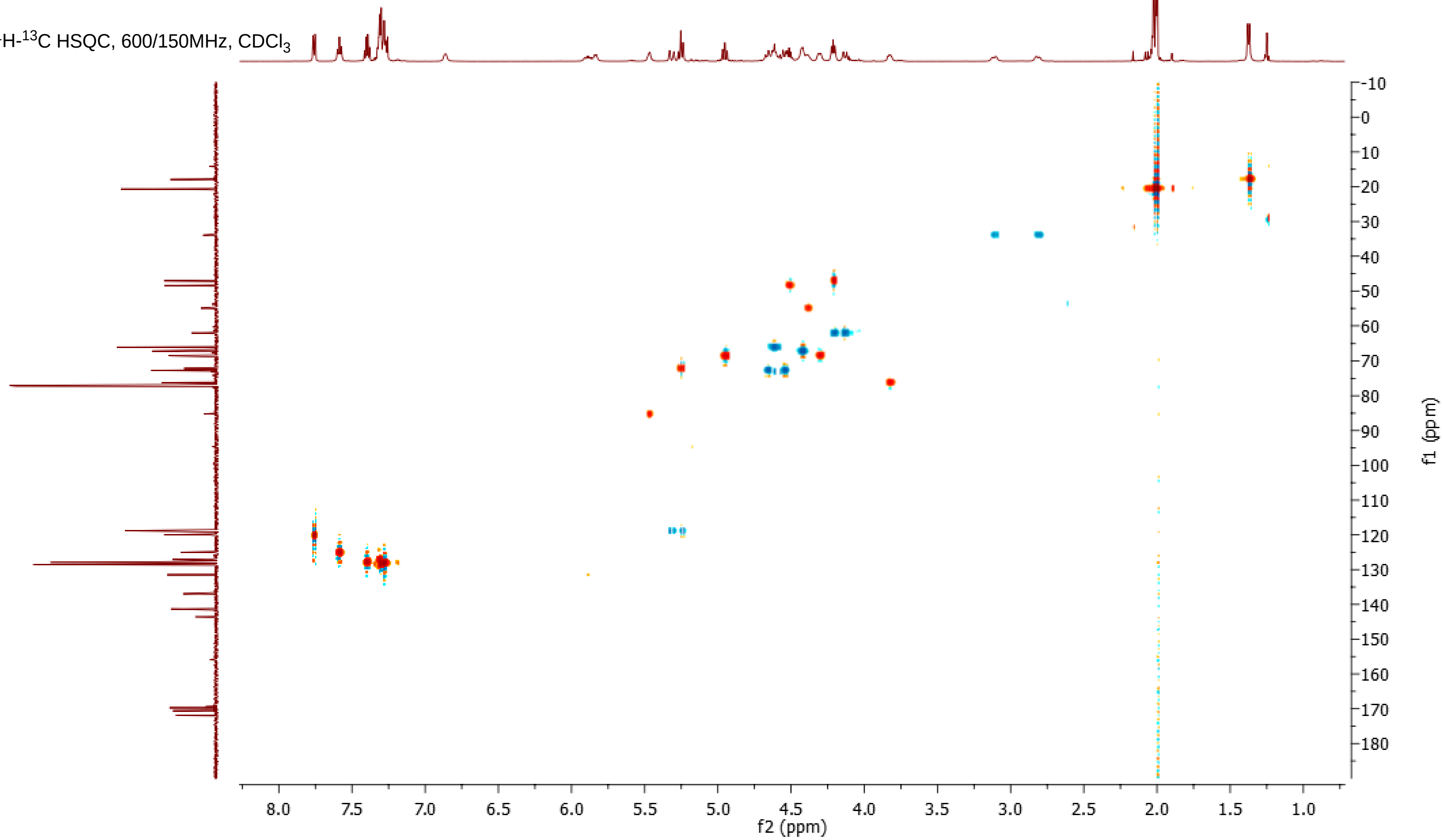

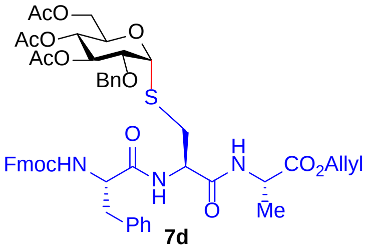

7d

^{1}H NMR

600 MHz

$CDCl_3$

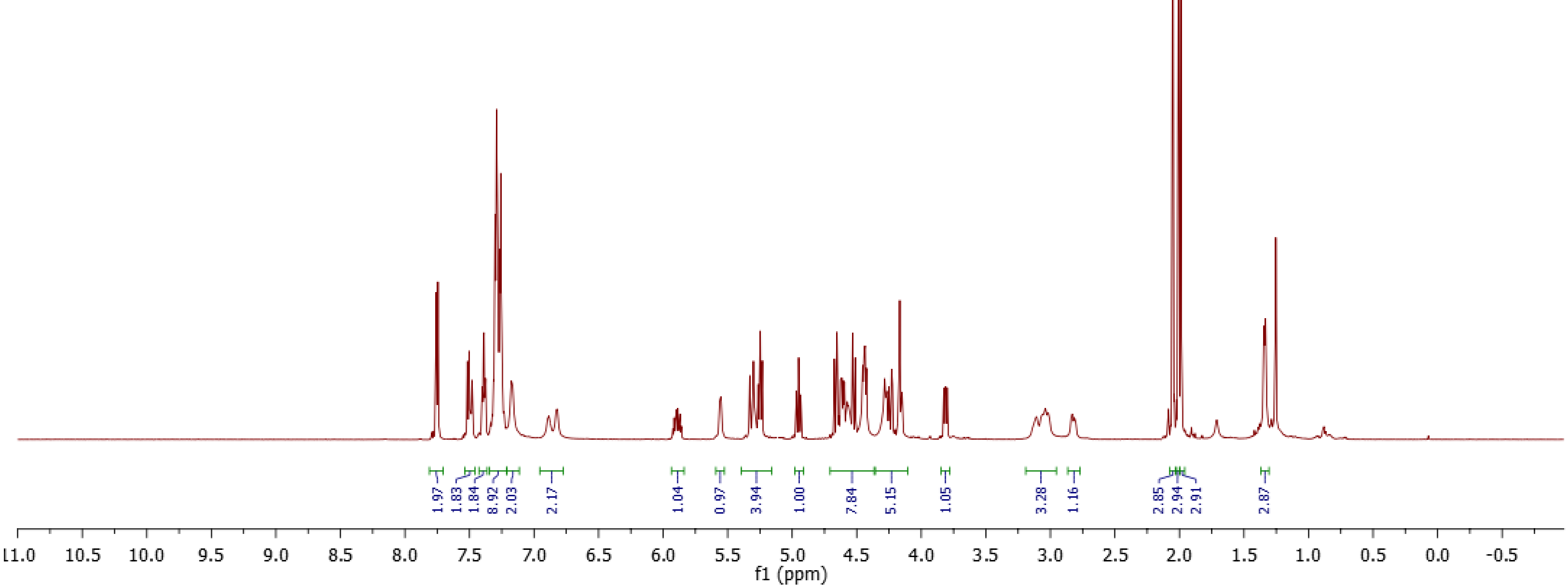

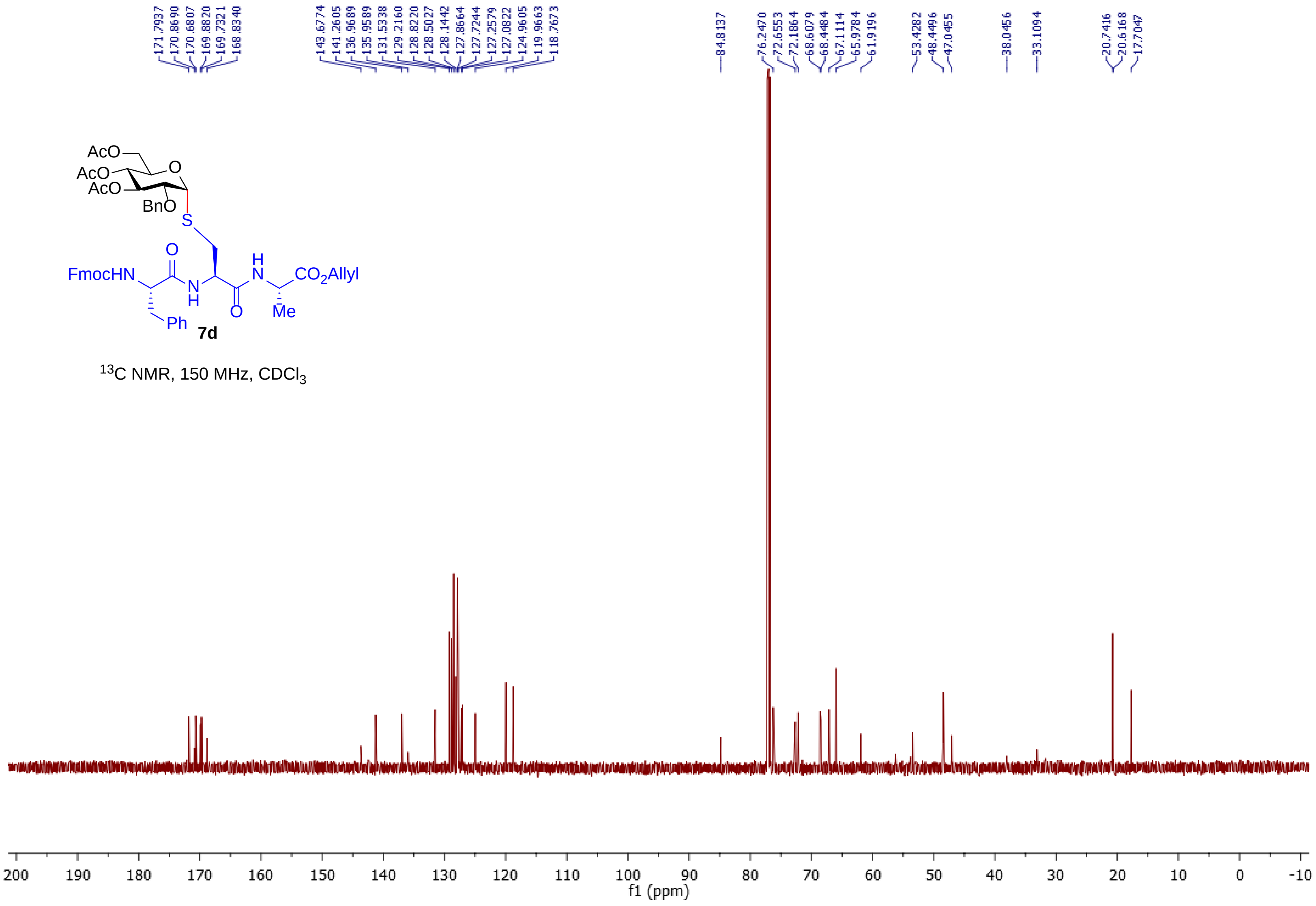

AcO
AcO
AcO
O
BnO
S
FmocHN
O
N
H
H
N
O
CO2Allyl
Me
Ph
7d
13C NMR, 150 MHz, CDCl3
171.7937
170.8690
170.6807
169.8820
169.7321
168.8340
143.6774
141.2605
136.9689
135.9589
131.5338
129.2160
128.8220
128.5027
128.1442
127.8664
127.7244
127.2579
127.0822
124.9605
119.9663
118.7673
84.8137
76.2470
72.6553
72.1864
68.6079
68.4484
67.1114
65.9784
61.9196
53.4282
48.4496
47.0455
38.0456
33.1094
20.7416
20.6168
17.7047
200 190 180 170 160 150 140 130 120 110 100 90 80 70 60 50 40 30 20 10 0 -10
f1 (ppm)

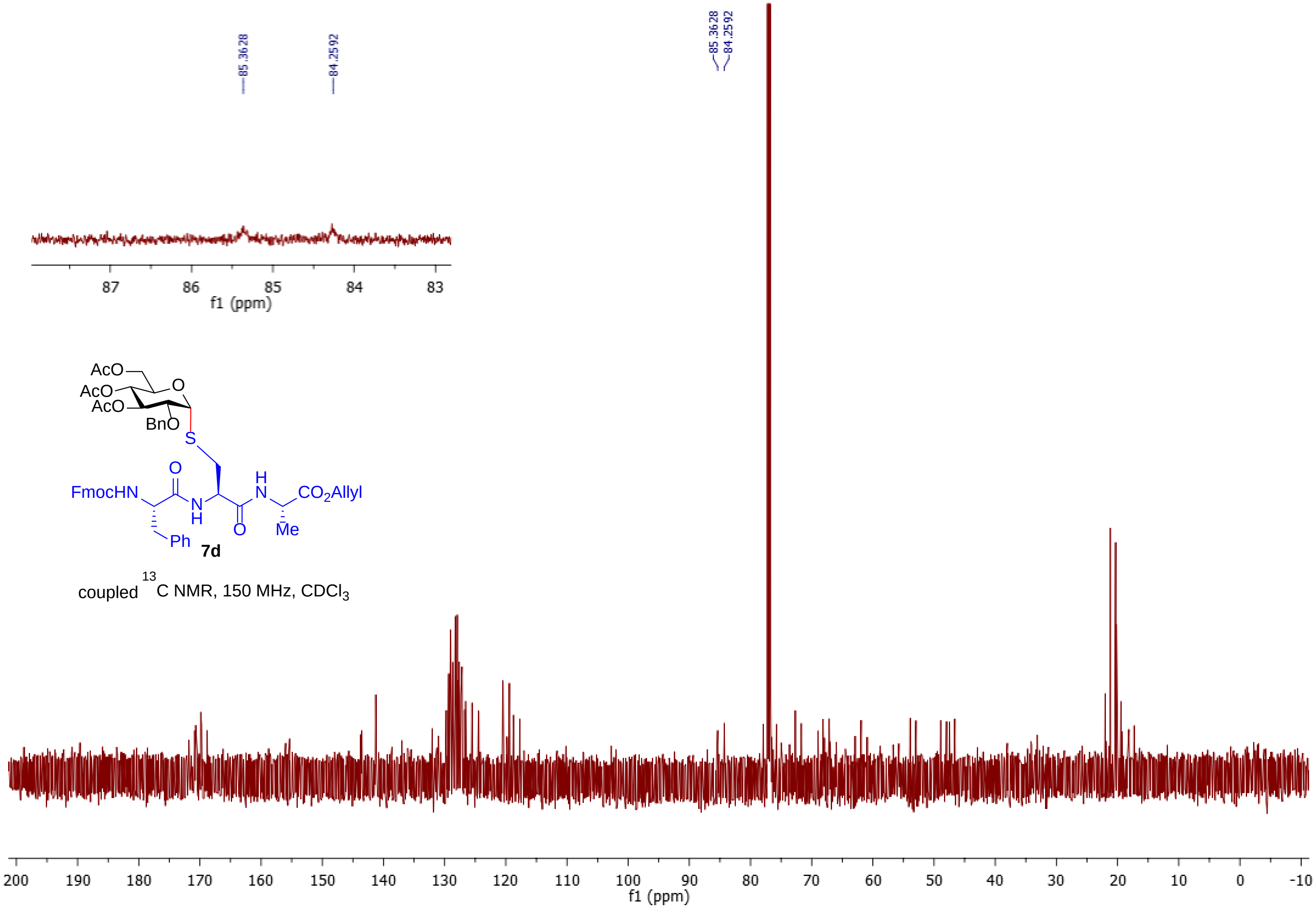

85.3628
84.2592
87
86
85
84
83
f1 (ppm)
AcO
AcO
AcO
O
BnO
S
FmocHN
O
N
H
H
N
O
CO2Allyl
Me
Ph
7d
coupled 13C NMR, 150 MHz, CDCl3
200
190
180
170
160
150
140
130
120
110
100
90
80
70
60
50
40
30
20
10
0
-10
f1 (ppm)

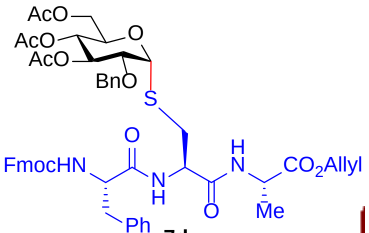

7d

^{1}H-^{13}C HSQC, 600/150MHz, $CDCl_3$

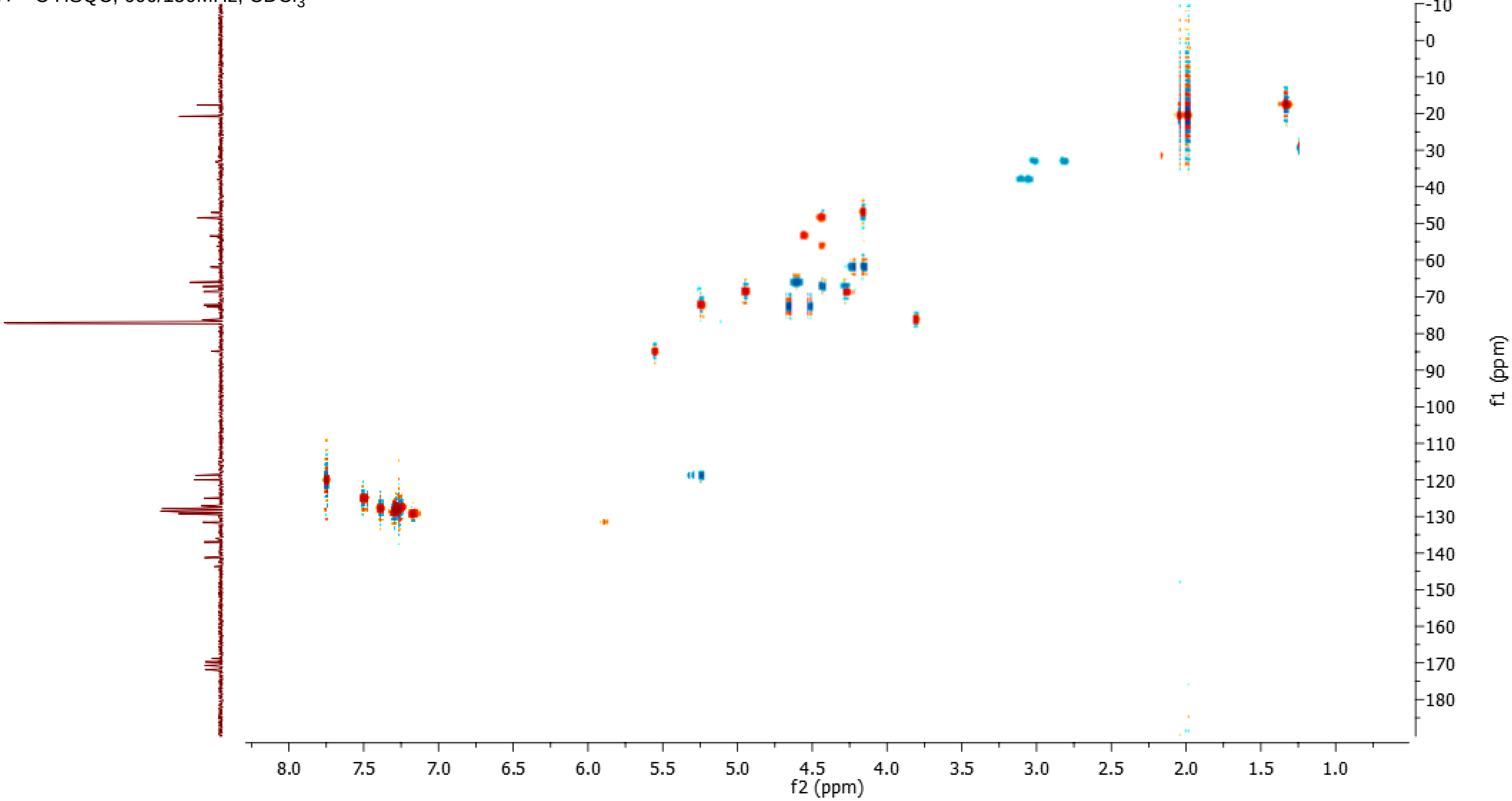

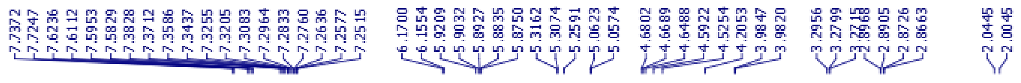

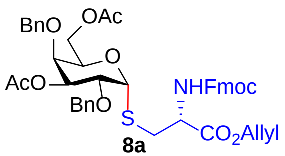

8a

^{1}H NMR
600 MHz
$CDCl_3$

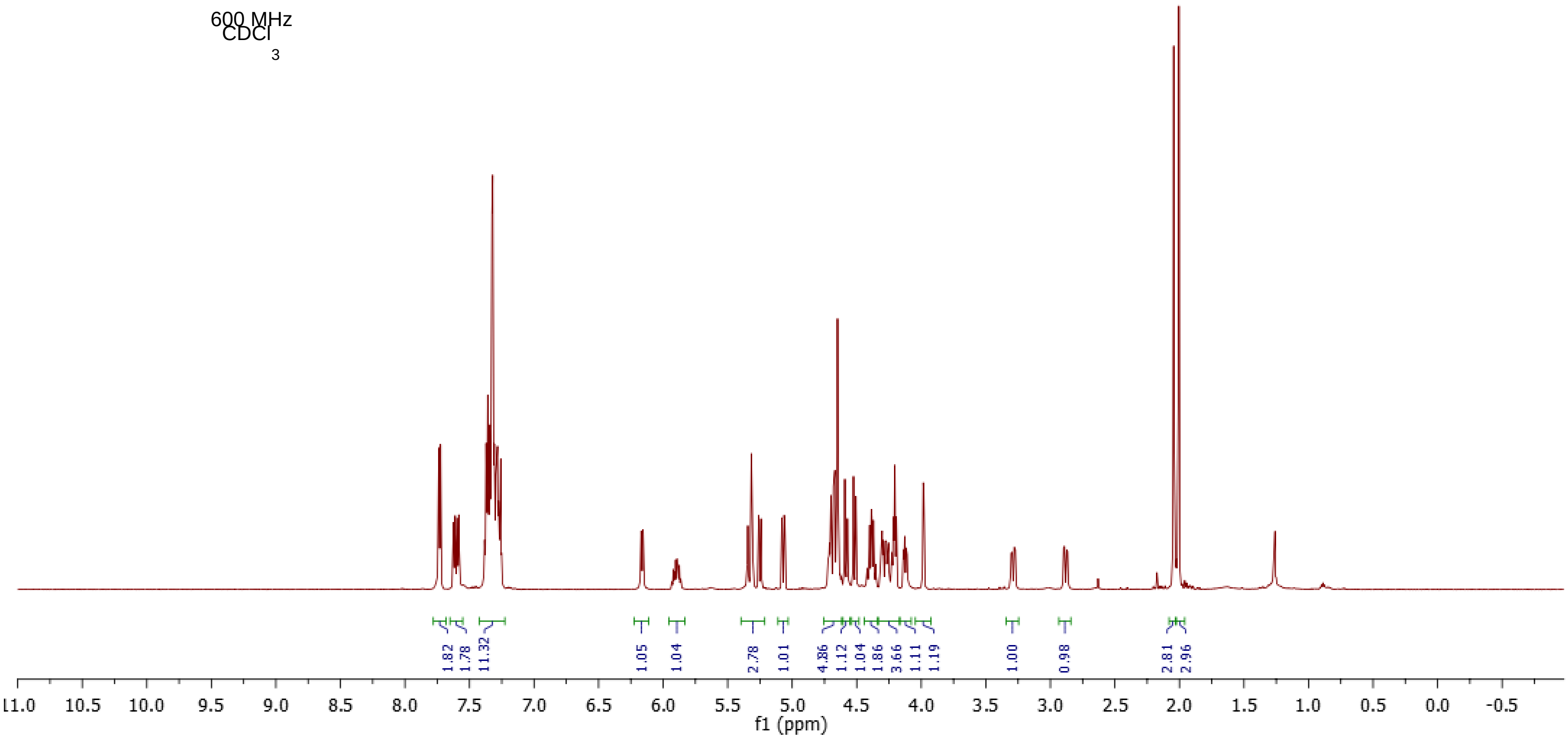

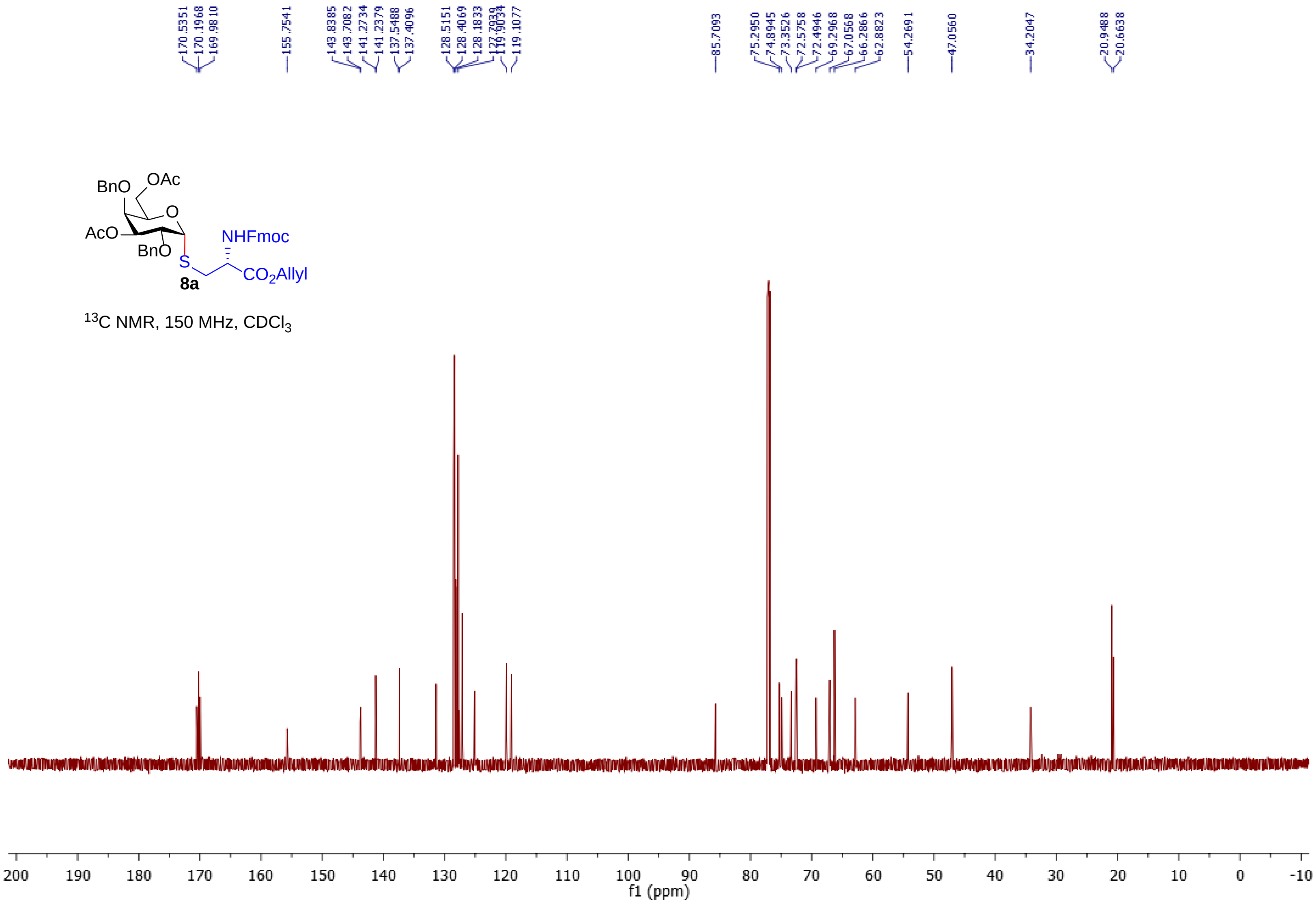

^{13}C NMR, 150 MHz, $CDCl_3$

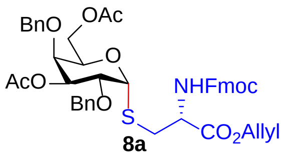

8a

^{1}H-^{13}C HSQC, 600/150MHz, $CDCl_3$

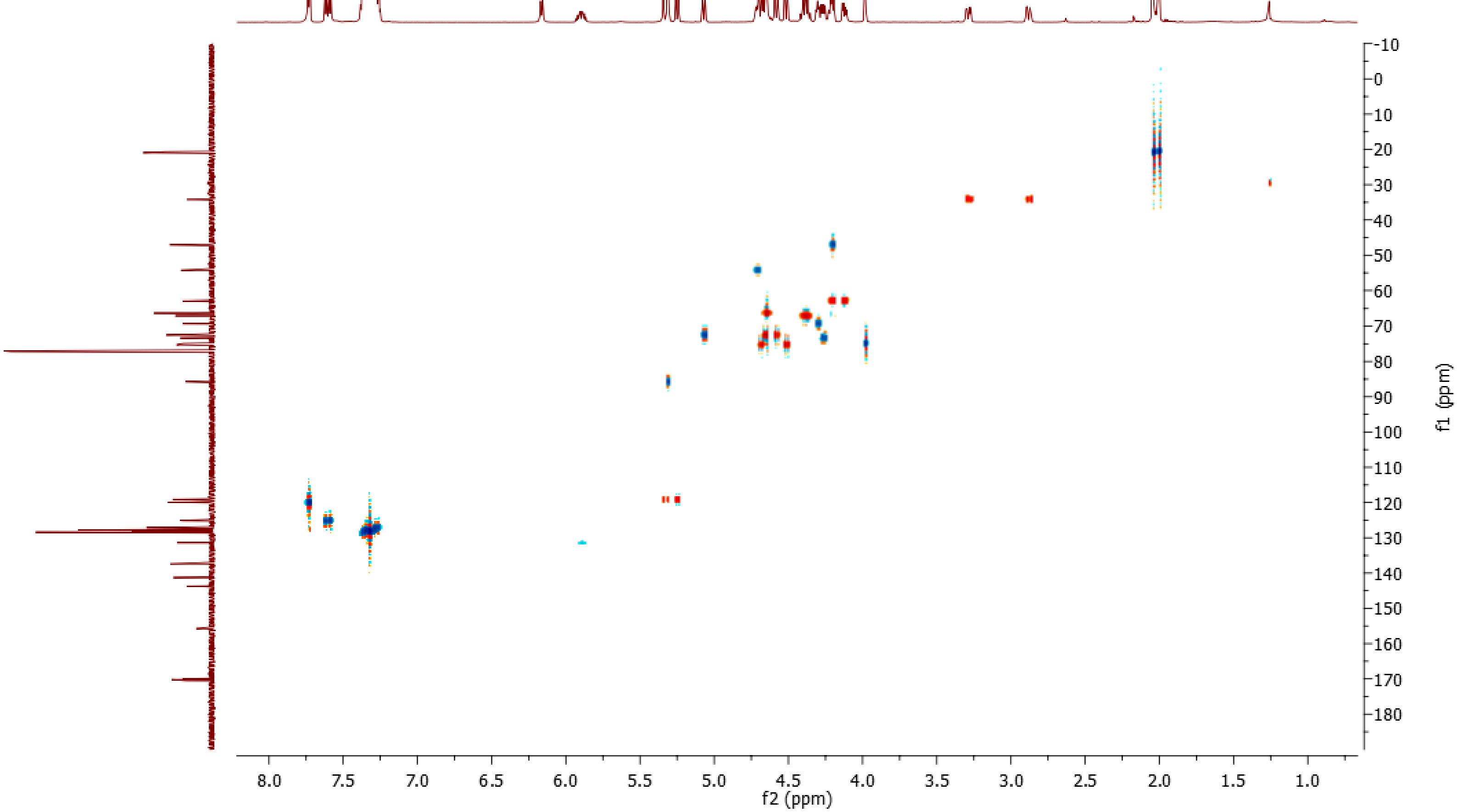

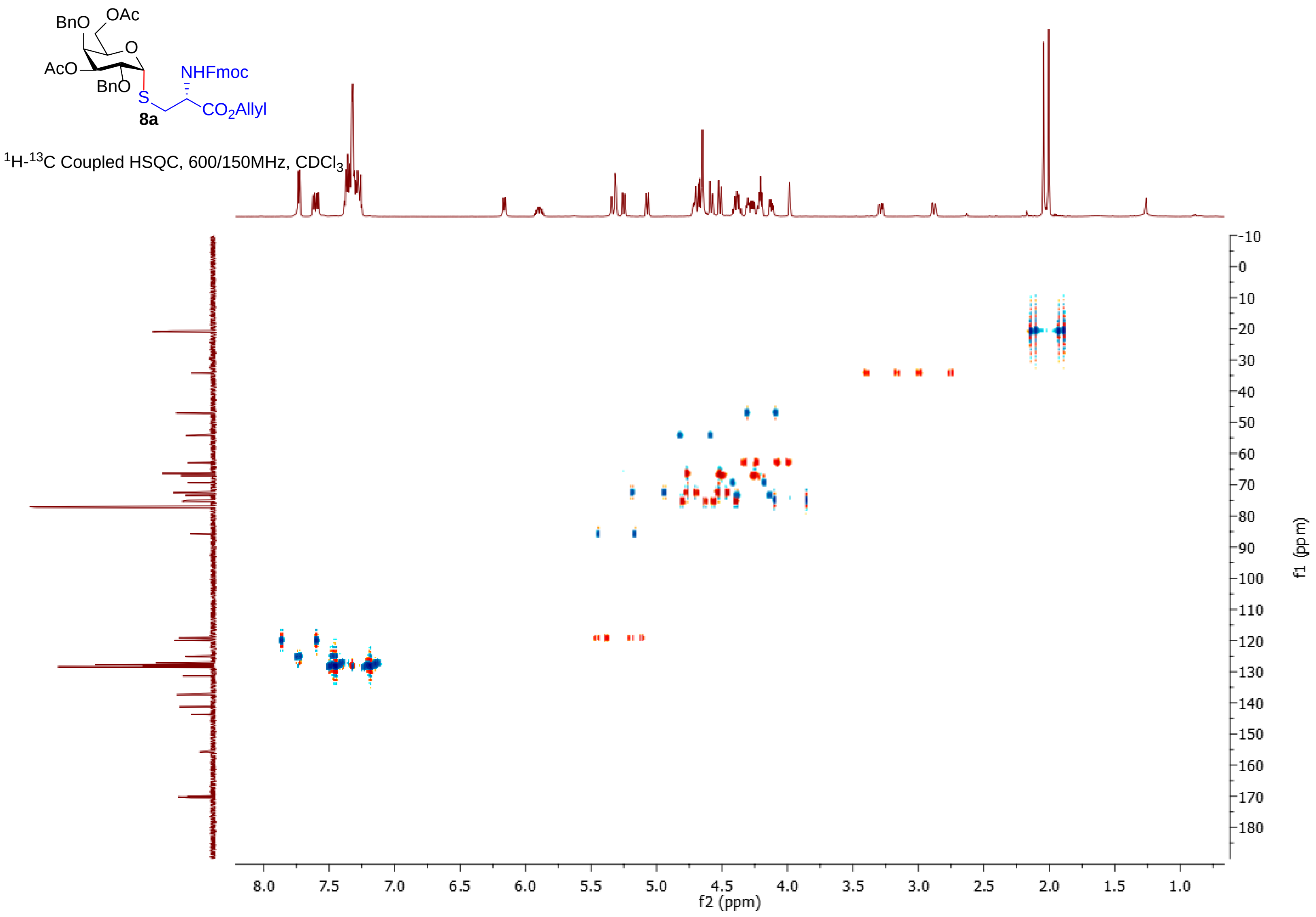

BnO
OAc
O
AcO
BnO
S
NHFmoc
CO2Allyl
8a
1H-13C Coupled HSQC, 600/150MHz, CDCl3
f2 (ppm)
f1 (ppm)

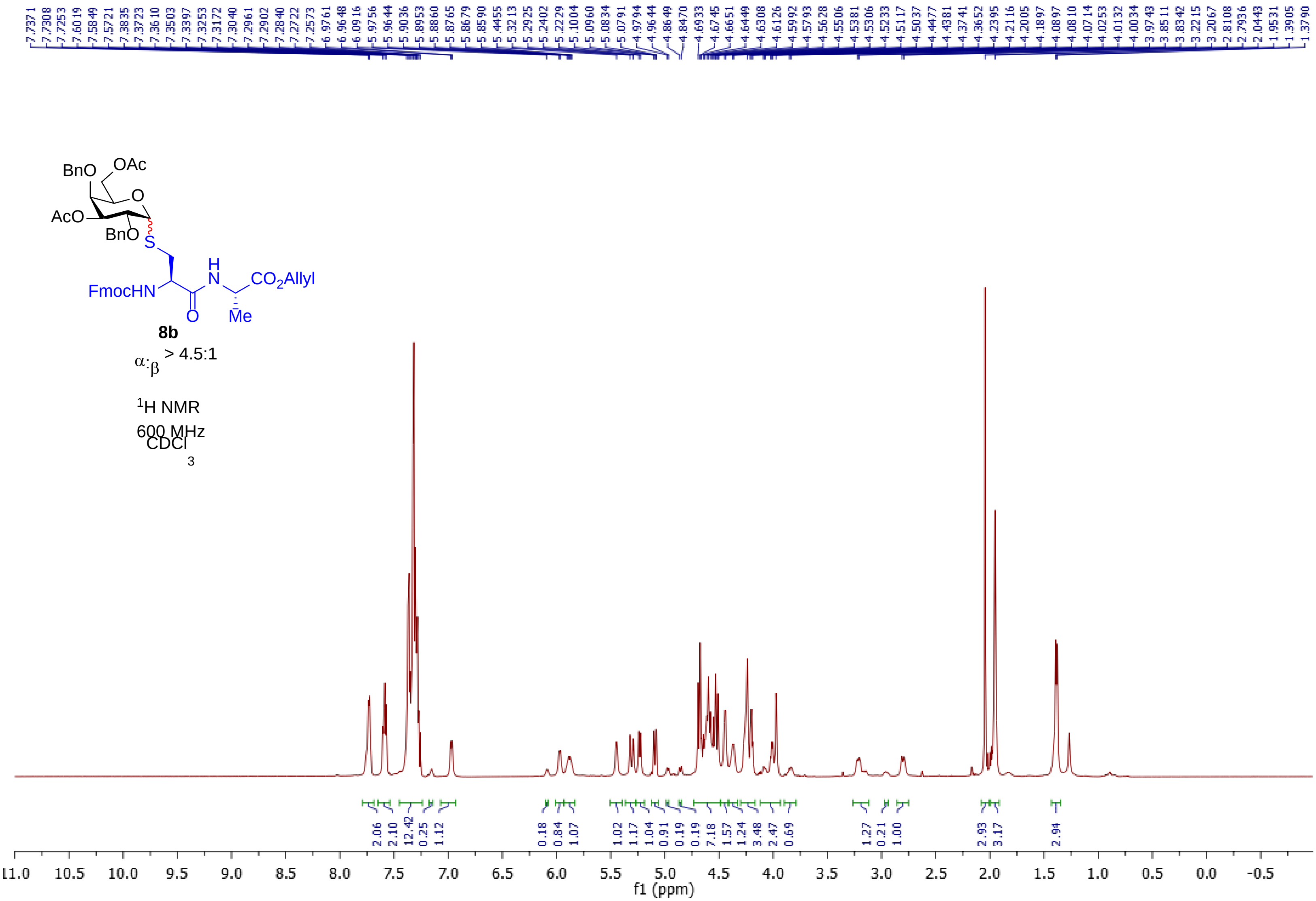

8b

α:β > 4.5:1

^{1}H NMR

600 MHz

$CDCl_3$

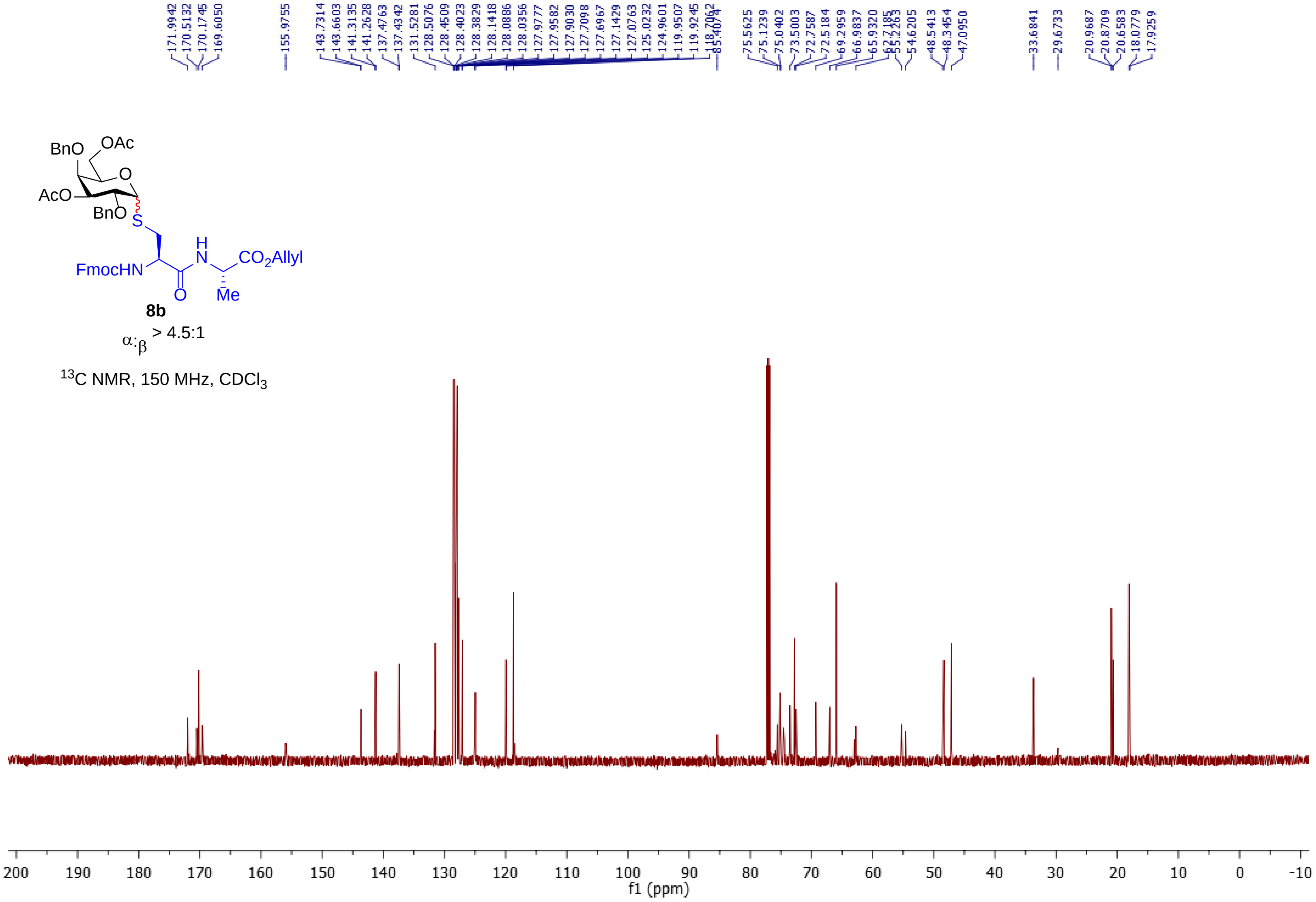

8b

α:β > 4.5:1

^{13}C NMR, 150 MHz, $CDCl_3$

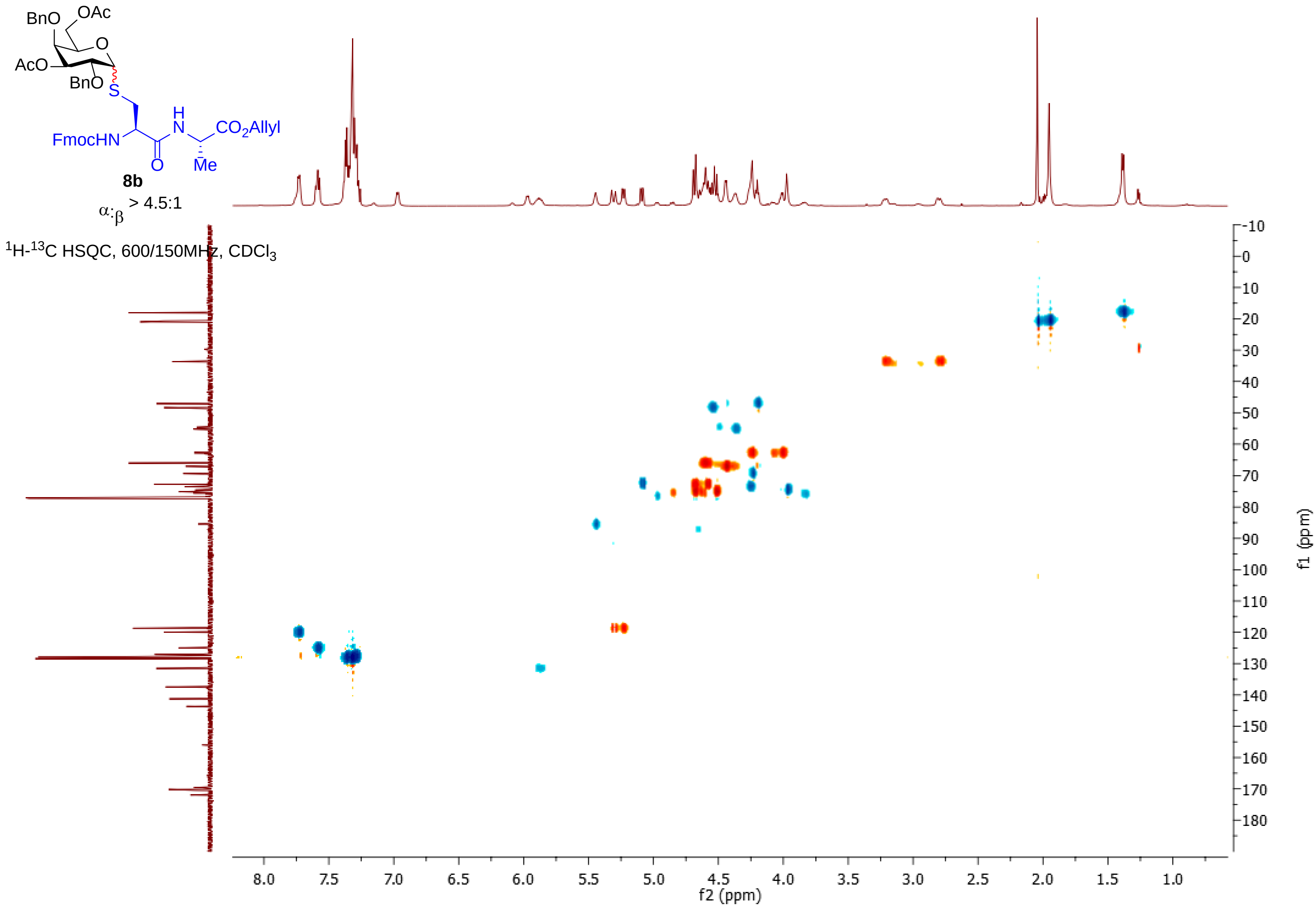
BnO
OAc
O
AcO
BnO
S
H
N
CO$_2$Allyl
FmocHN
O
Me
8b
α:β > 4.5:1
^{1}H-^{13}C HSQC, 600/150MHz, CDCl$_3$
f2 (ppm)
f1 (ppm)

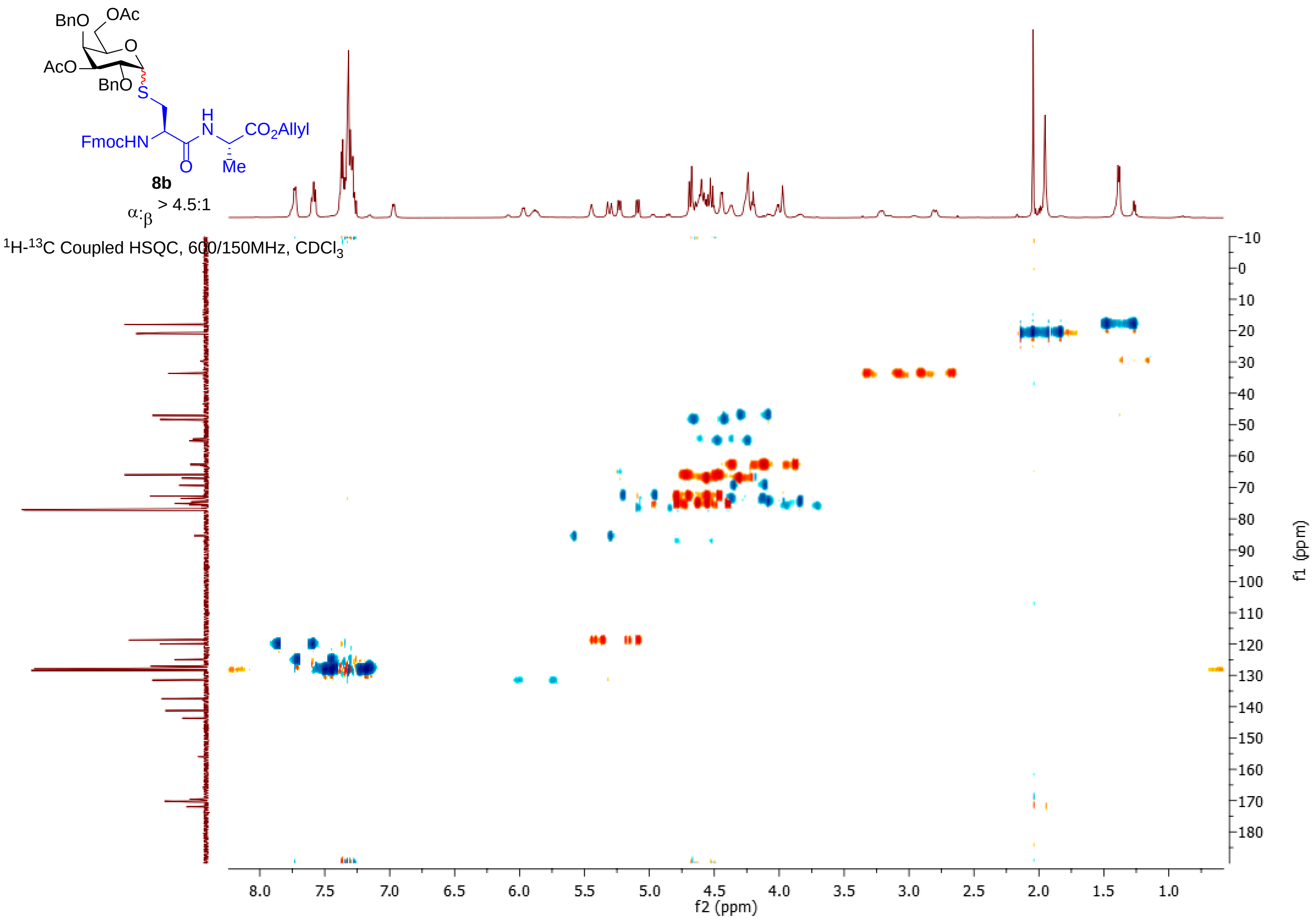
BnO
OAc
O
AcO
BnO
S
H
N
CO2Allyl
FmocHN
O
Me
8b
α:β > 4.5:1
1H-13C Coupled HSQC, 600/150MHz, CDCl3
f2 (ppm)
f1 (ppm)

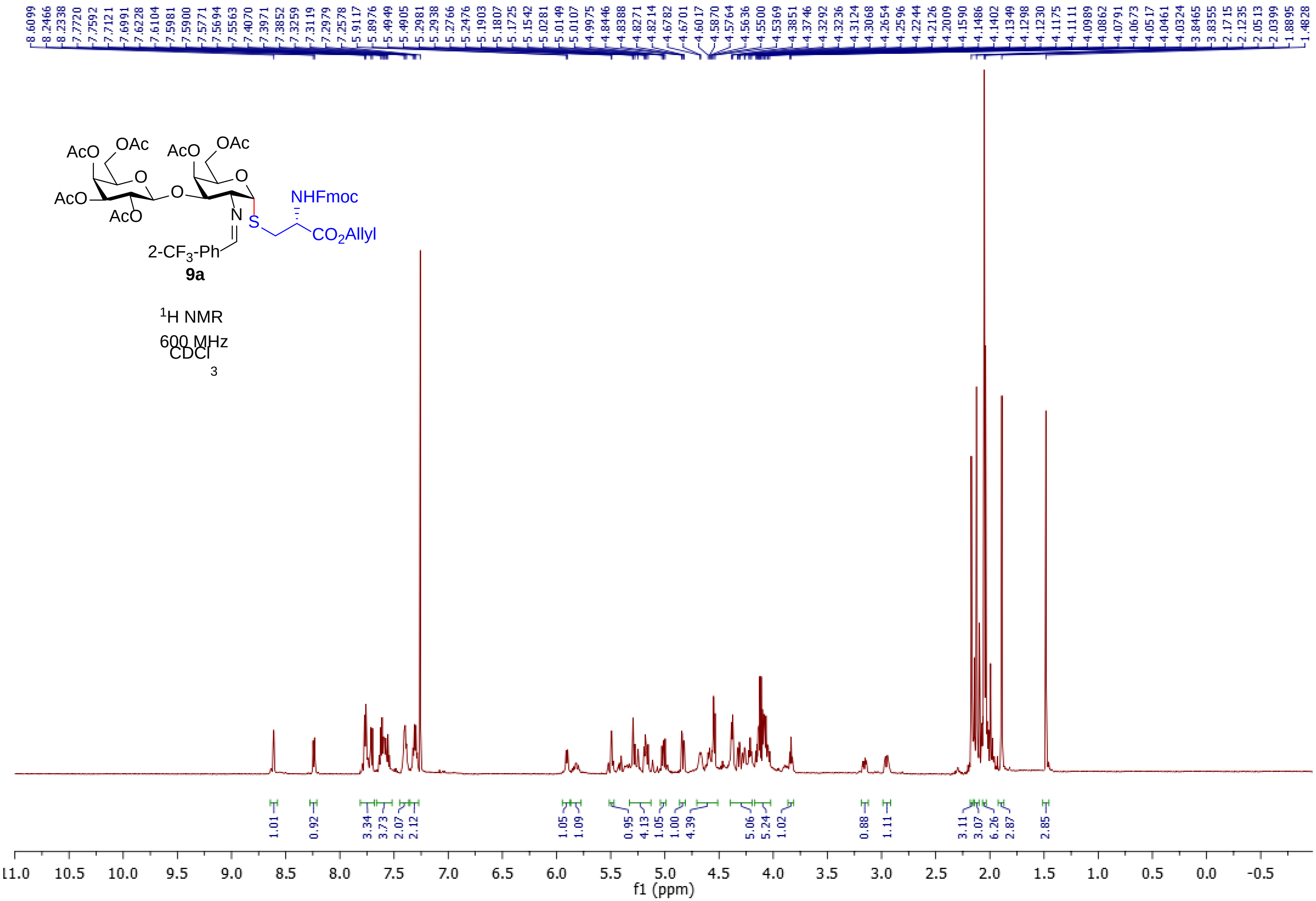

AcO
OAc
O
AcO
O
AcO
OAc
O
AcO
N
S
NHFmoc
CO_2Allyl
2-CF_3-Ph
9a
^{1}H NMR
600 MHz
$CDCl_3$
8.6099 8.2466 8.2338 7.7720 7.7592 7.7121 7.6991 7.6228 7.6104 7.5981 7.5900 7.5771 7.5694 7.5563 7.4070 7.3971 7.3852 7.3259 7.3119 7.2979 7.2578 5.9117 5.8976 5.4949 5.4905 5.2981 5.2938 5.2766 5.2476 5.1903 5.1807 5.1725 5.1542 5.0281 5.0149 5.0107 4.9975 4.8446 4.8388 4.8271 4.8214 4.6782 4.6701 4.6017 4.5870 4.5764 4.5636 4.5500 4.5369 4.3851 4.3746 4.3292 4.3236 4.3124 4.3068 4.2654 4.2596 4.2244 4.2126 4.2009 4.1590 4.1486 4.1402 4.1349 4.1298 4.1230 4.1175 4.1111 4.0989 4.0862 4.0791 4.0673 4.0517 4.0461 4.0324 3.8465 3.8355 2.1715 2.1235 2.0513 2.0399 1.8895 1.4828
1.01 0.92 3.34 3.73 2.07 2.12 1.05 1.09 0.95 4.13 1.05 1.00 4.39 5.06 5.24 1.02 0.88 1.11 3.11 3.07 6.26 2.87 2.85
11.0 10.5 10.0 9.5 9.0 8.5 8.0 7.5 7.0 6.5 6.0 5.5 5.0 4.5 4.0 3.5 3.0 2.5 2.0 1.5 1.0 0.5 0.0 -0.5
f1 (ppm)

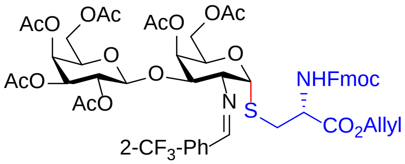

9a

^{13}C NMR, 150 MHz, $CDCl_3$

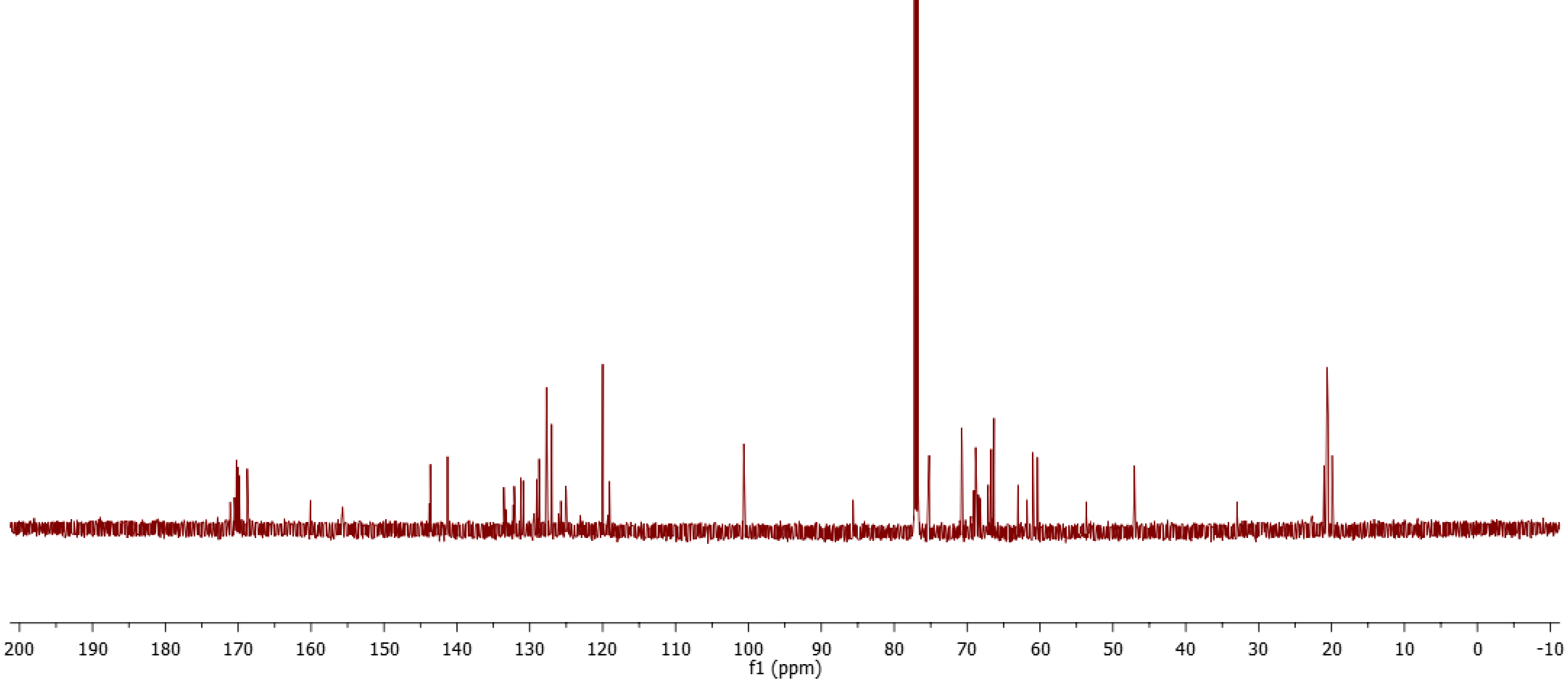

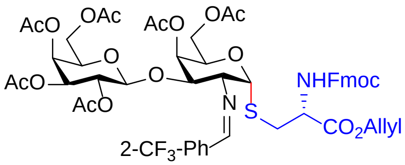

9a

^{1}H-^{13}C HSQC, 600/150MHz, $CDCl_3$

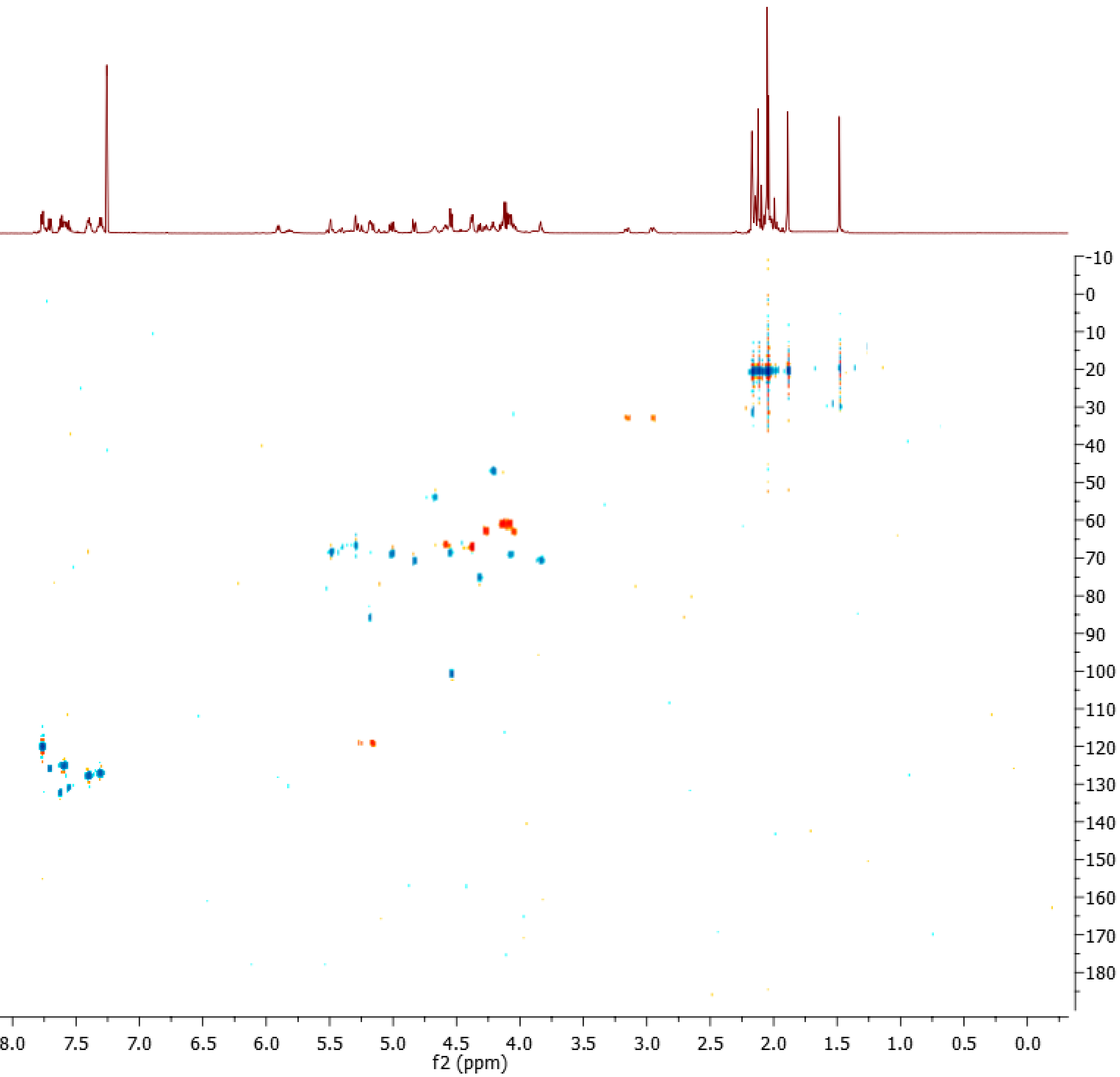

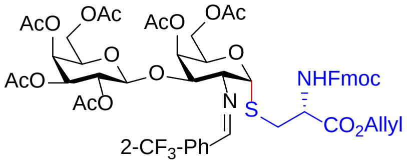

9a

^{1}H-^{13}C Coupled HSQC, 600/150MHz, $CDCl_3$

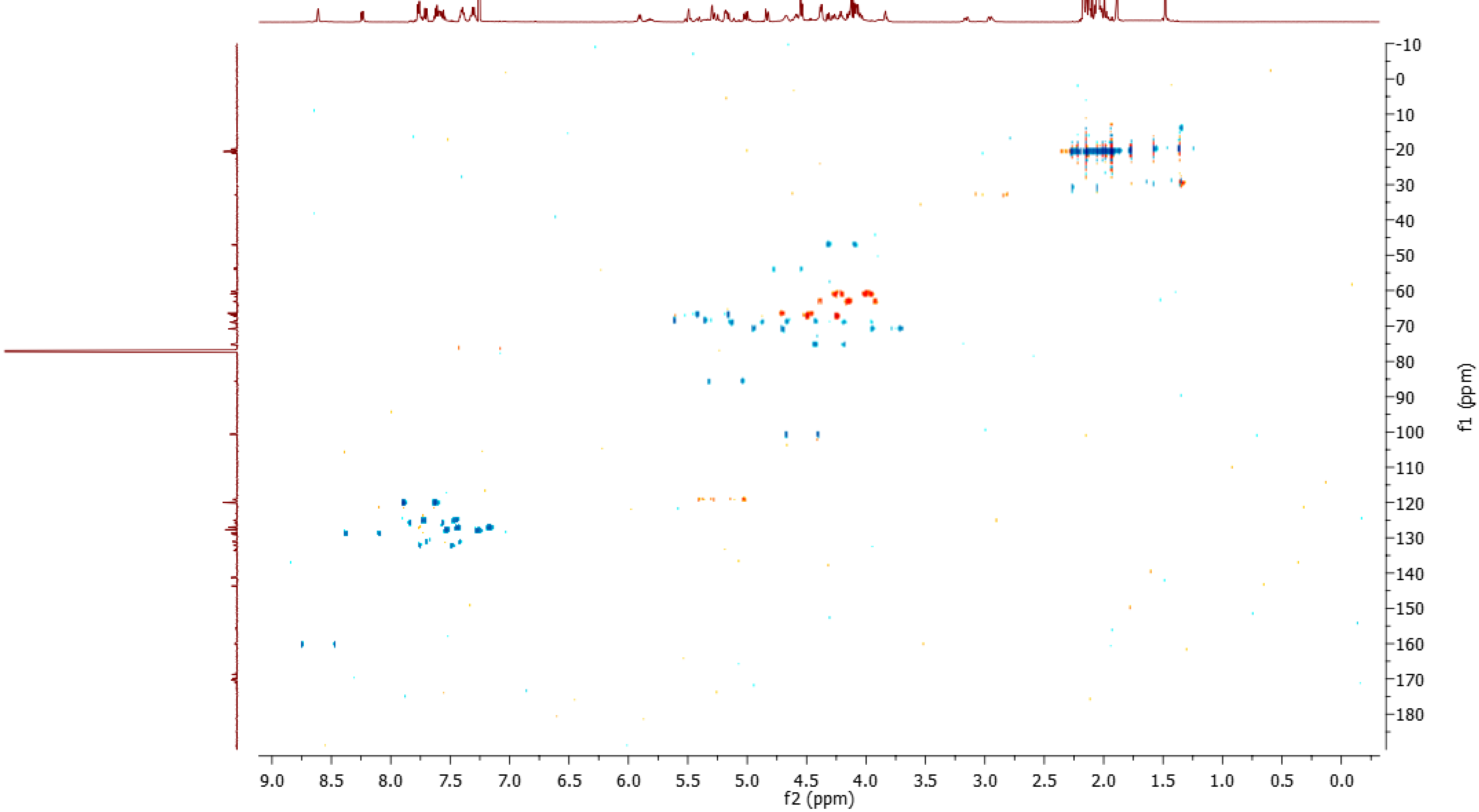

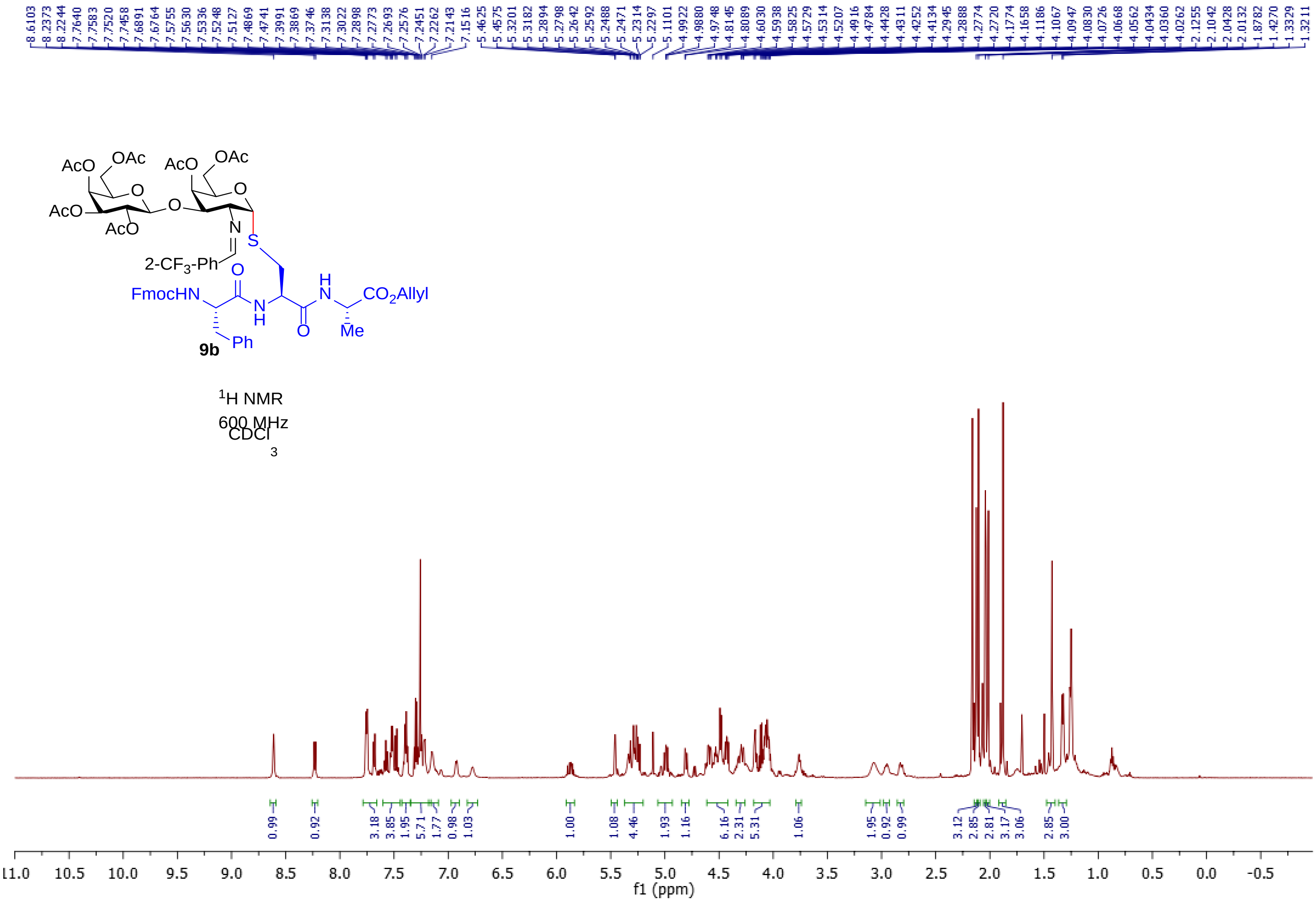

9b
^{1}H NMR
600 MHz
$CDCl_3$
f1 (ppm)

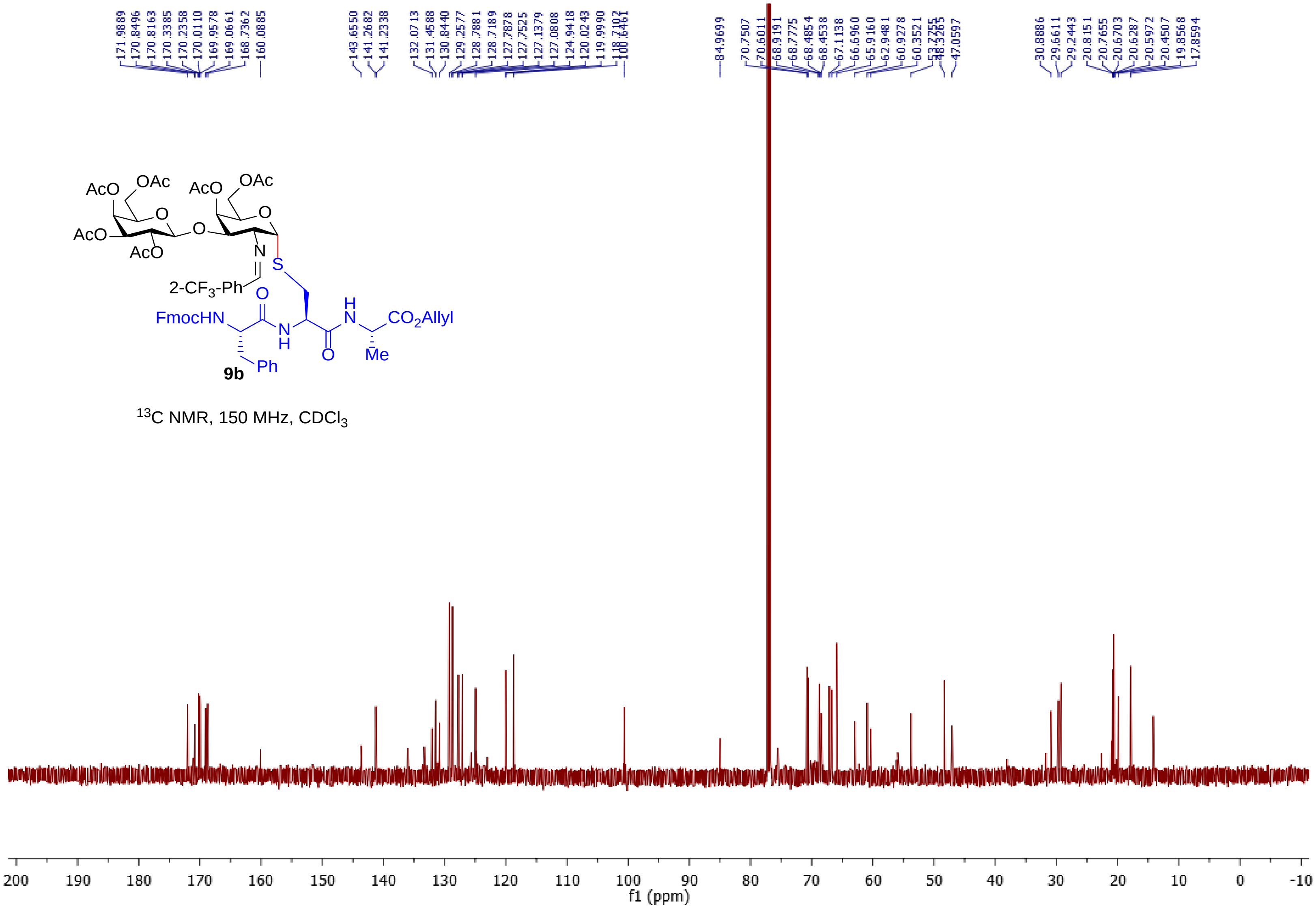
AcO
OAc
AcO
OAc
AcO
AcO
O
N
S
2-CF3-Ph
FmocHN
N
H
O
CO2Allyl
Me
Ph
9b
13C NMR, 150 MHz, CDCl3
171.9889
170.8496
170.8163
170.3385
170.2358
170.0110
169.9578
169.0661
168.7362
160.0885
143.6550
141.2682
141.2338
132.0713
131.4588
130.8440
129.2577
128.7881
128.7189
127.7878
127.7525
127.1379
127.0808
124.9418
120.0243
119.9990
100.6461
84.9699
70.7507
70.6011
68.9191
68.7775
68.4854
68.4538
67.1138
66.6960
65.9160
62.9481
60.9278
60.3521
47.0597
30.8886
29.6611
29.2443
20.8151
20.7655
20.6703
20.6287
20.5972
20.4507
19.8568
17.8594
200 190 180 170 160 150 140 130 120 110 100 90 80 70 60 50 40 30 20 10 0 -10
f1 (ppm)

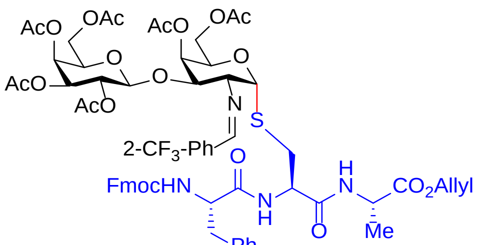

9b

^{1}H-^{13}C HSQC, 600/150MHz, $CDCl_3$

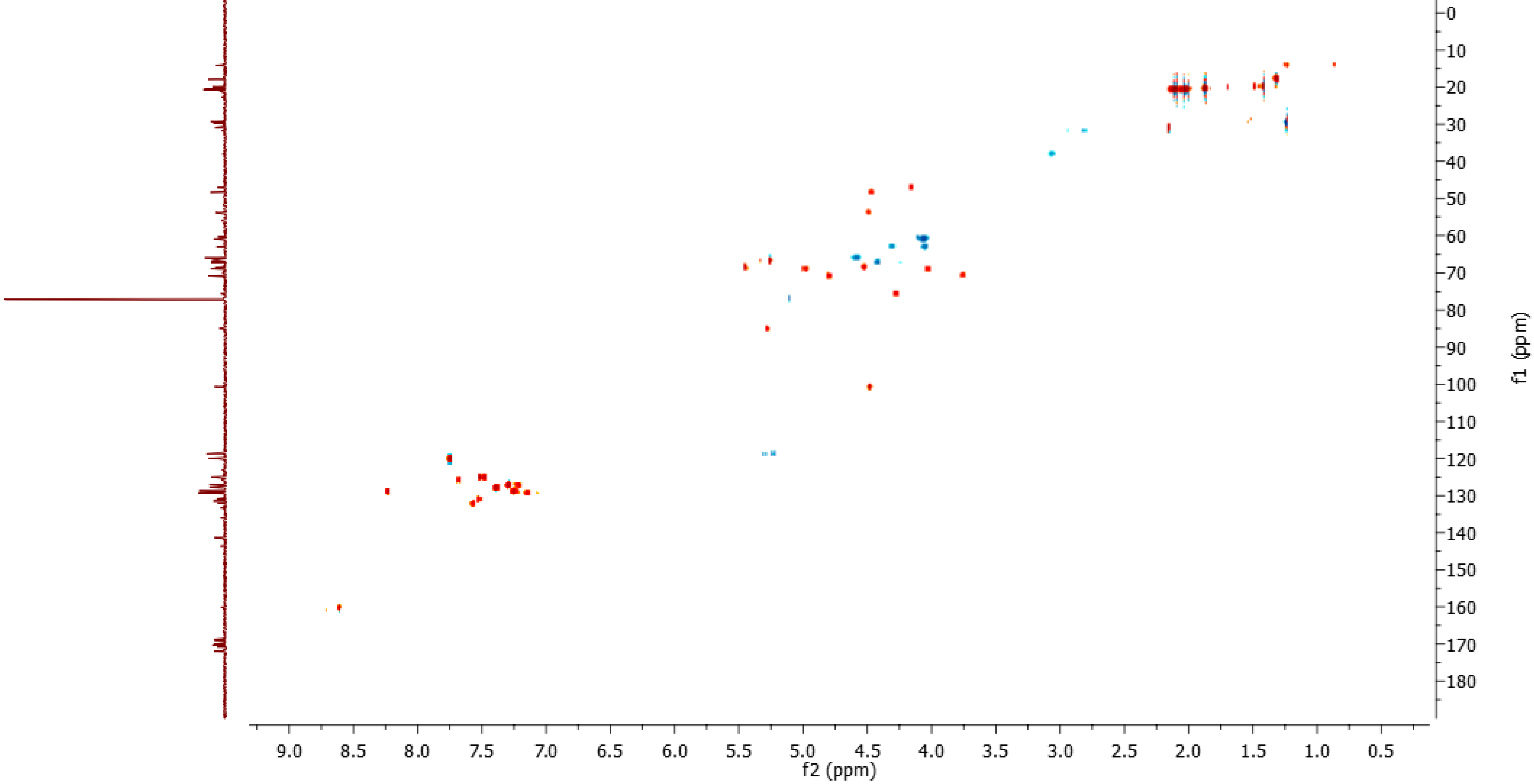

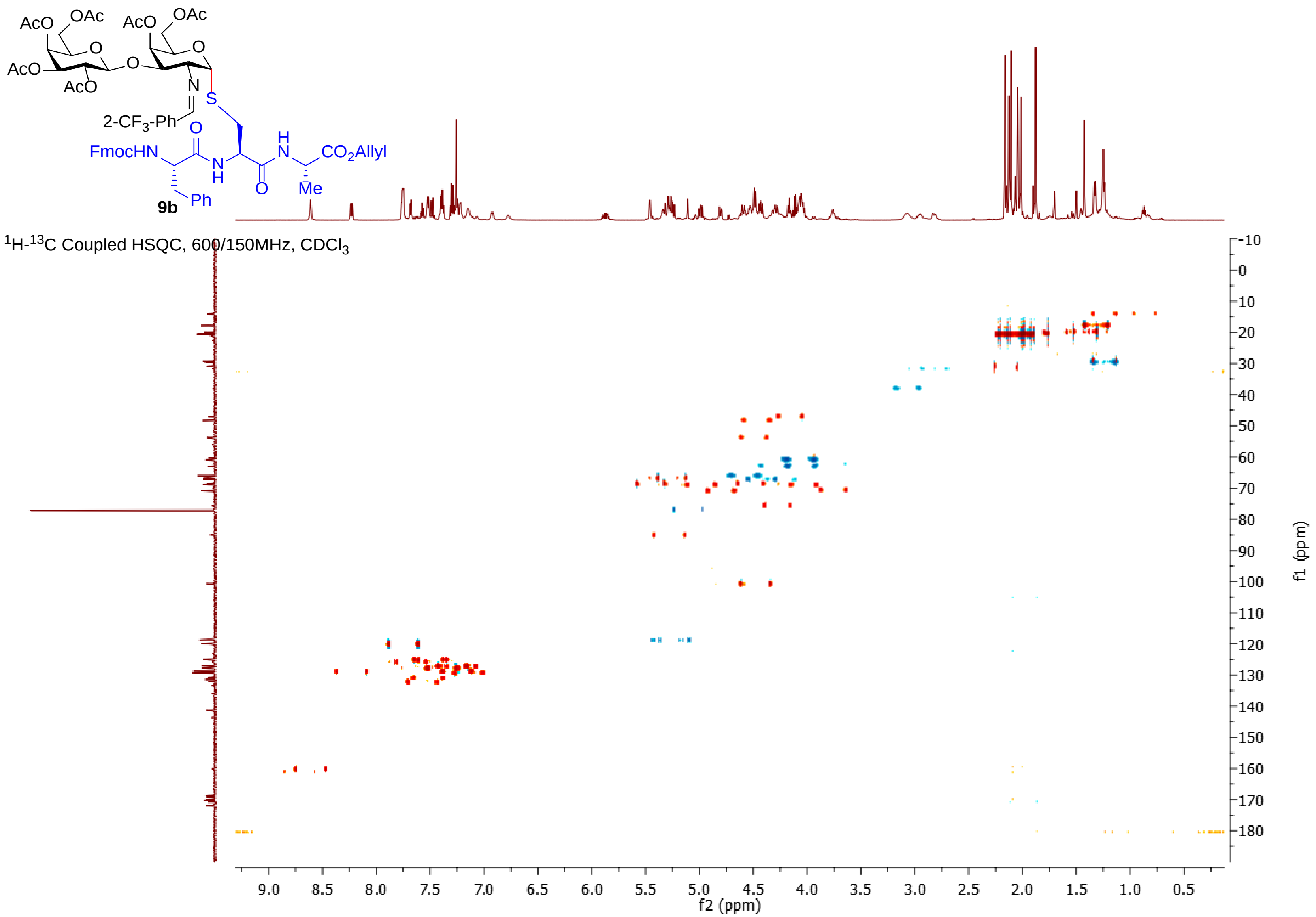
AcO
OAc
AcO
OAc
AcO
O
AcO
N
S
2-CF3-Ph
FmocHN
O
N
H
H
N
CO2Allyl
O
Me
Ph
9b
1H-13C Coupled HSQC, 600/150MHz, CDCl3
f2 (ppm)
f1 (ppm)

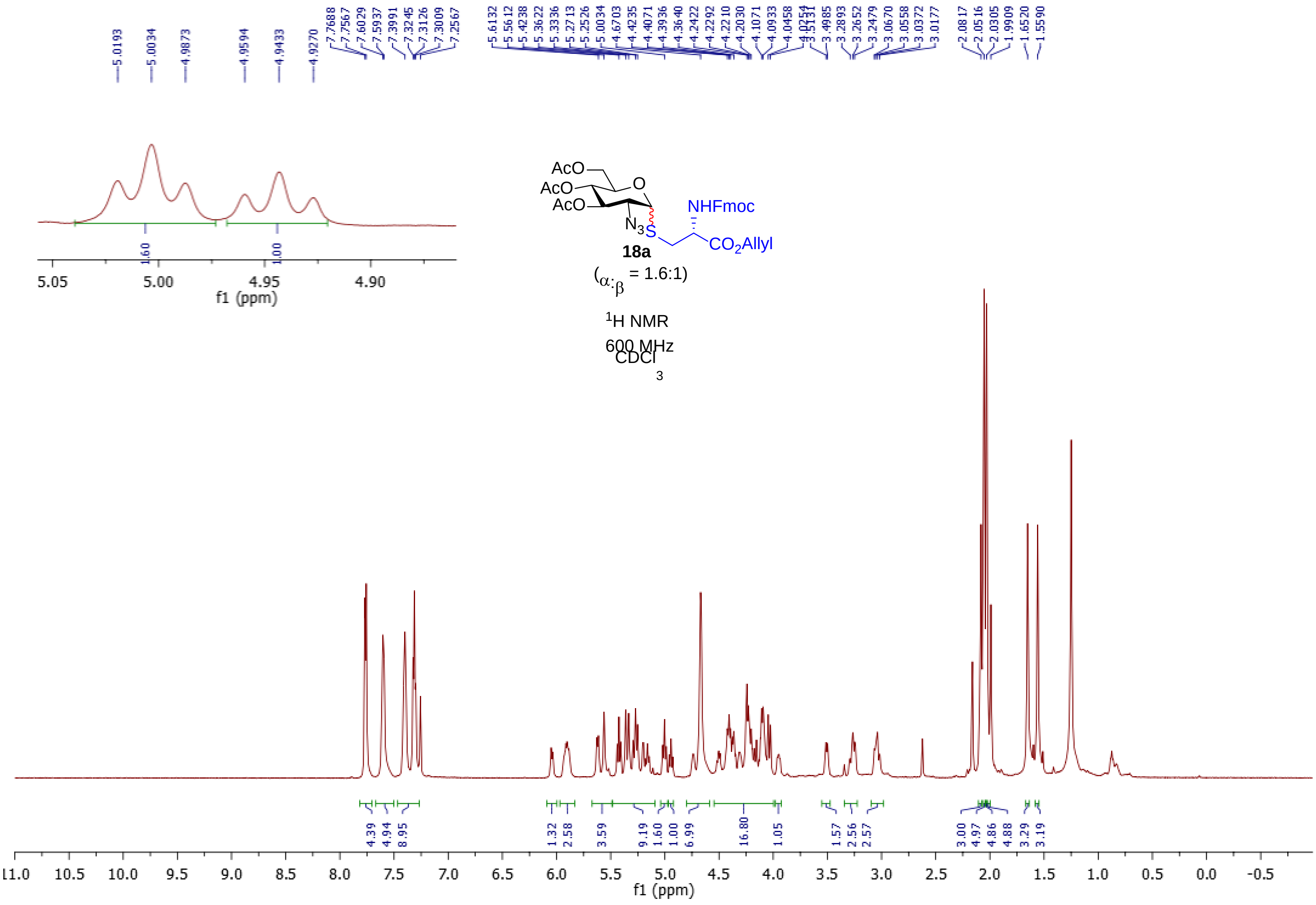

18a
(α:β = 1.6:1)
¹H NMR
600 MHz
CDCl3
f1 (ppm)

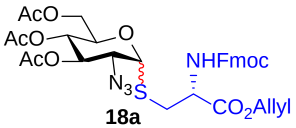

18a

(α:β = 1.6:1)

^{13}C NMR, 150 MHz, $CDCl_3$

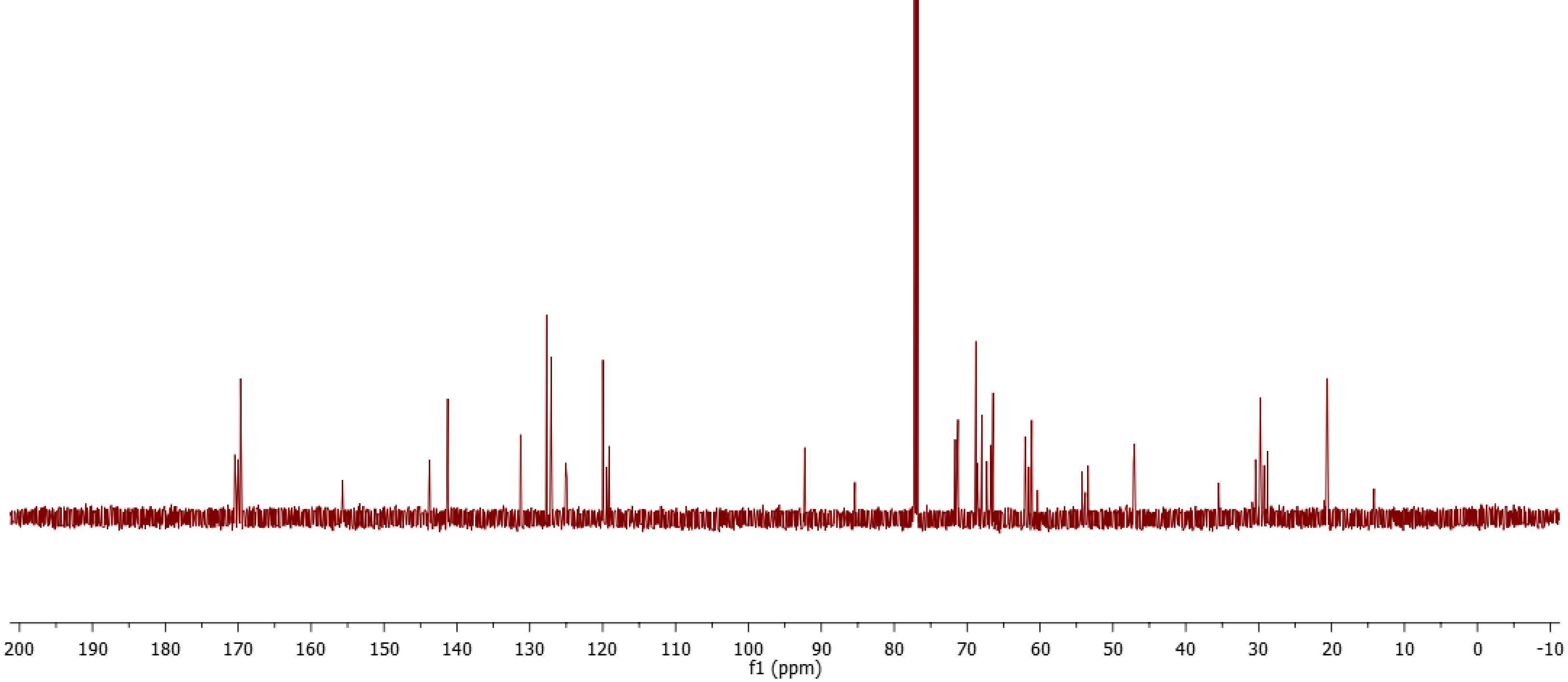

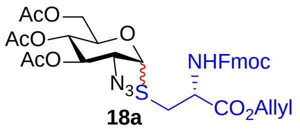

18a

(α:β = 1.6:1)

^{1}H-^{13}C HSQC, 600/150MHz, $CDCl_3$

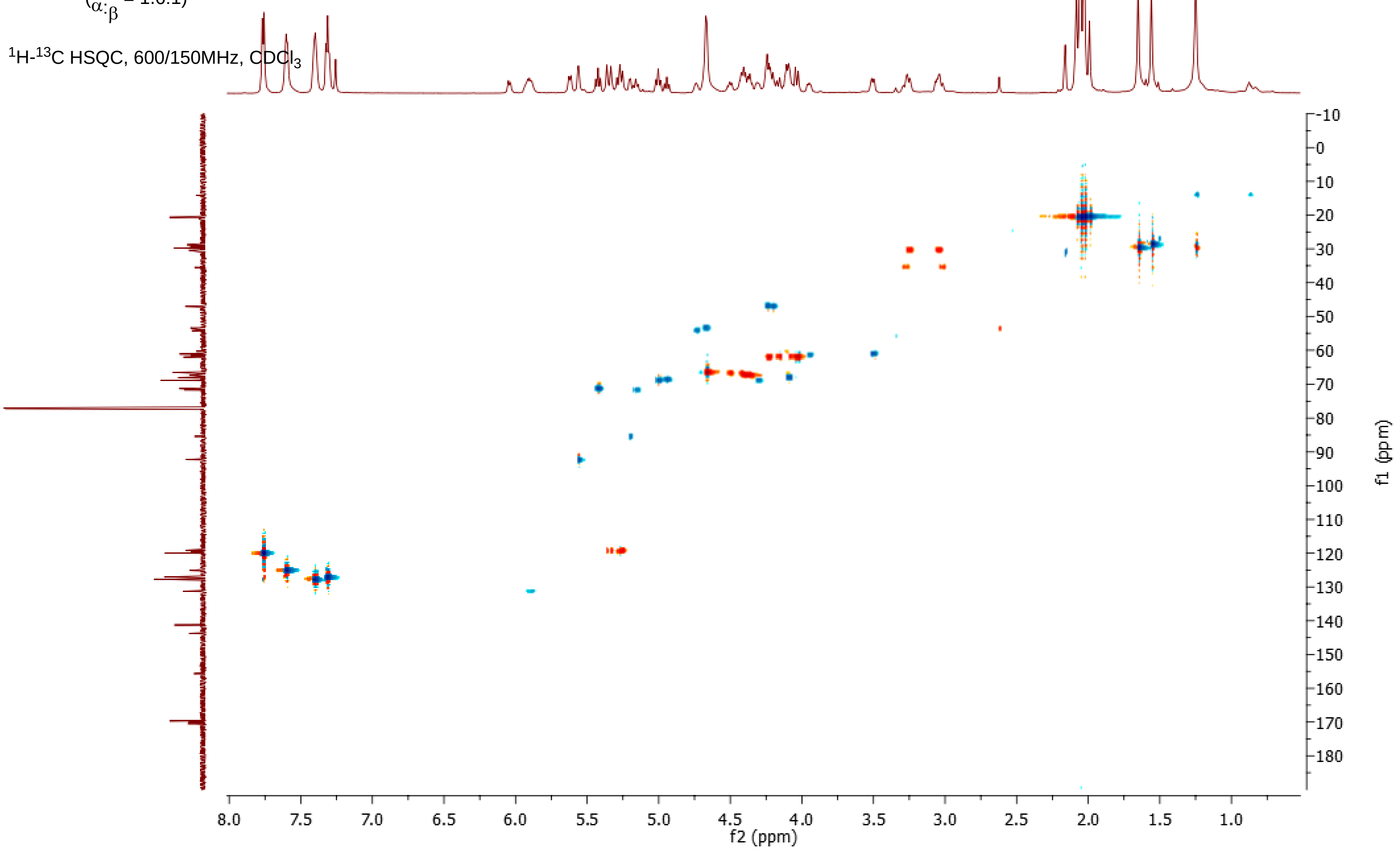

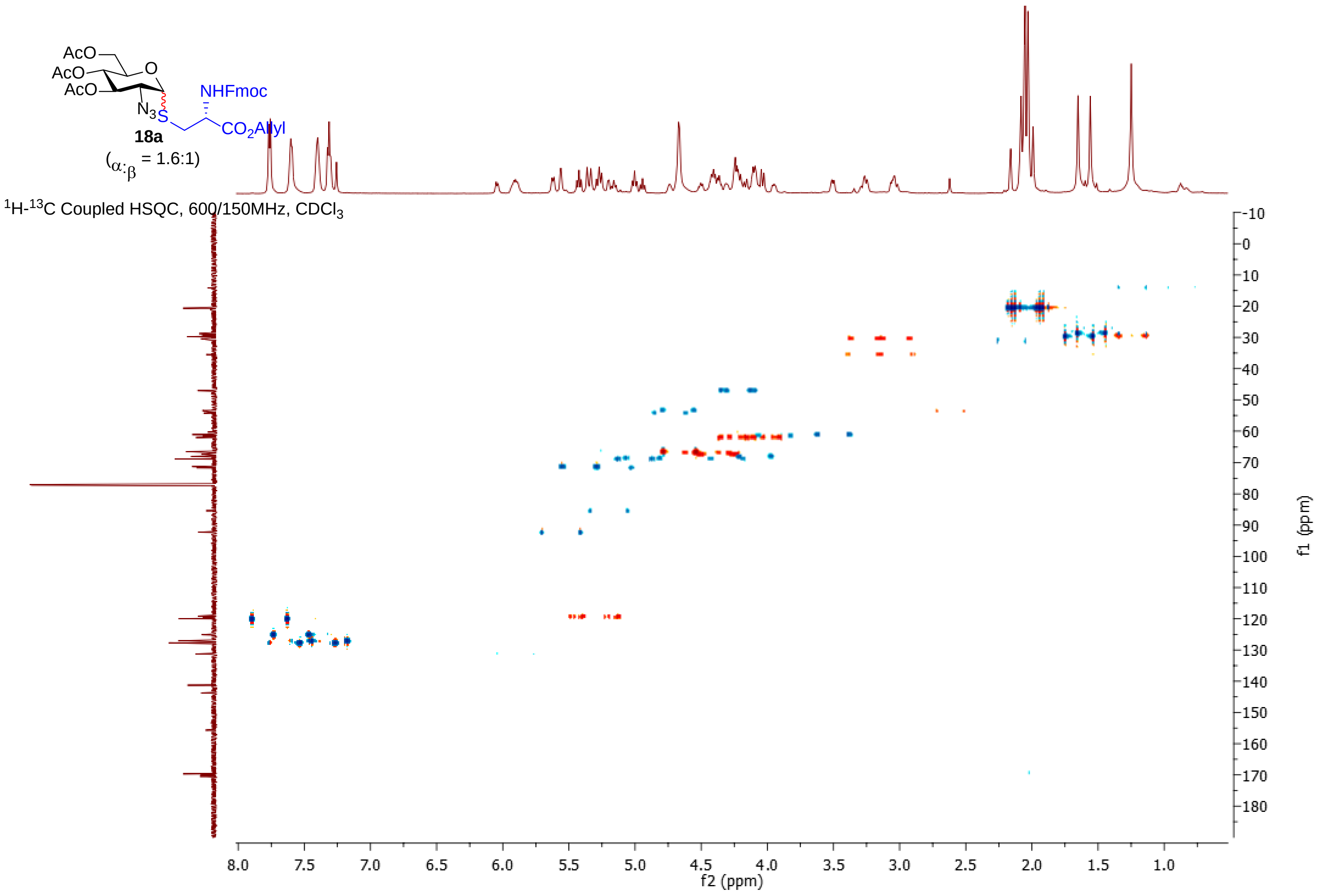

AcO
AcO
AcO
O
N3
S
NHFmoc
CO2Allyl
18a
(α:β = 1.6:1)
1H-13C Coupled HSQC, 600/150MHz, CDCl3
f2 (ppm)
f1 (ppm)

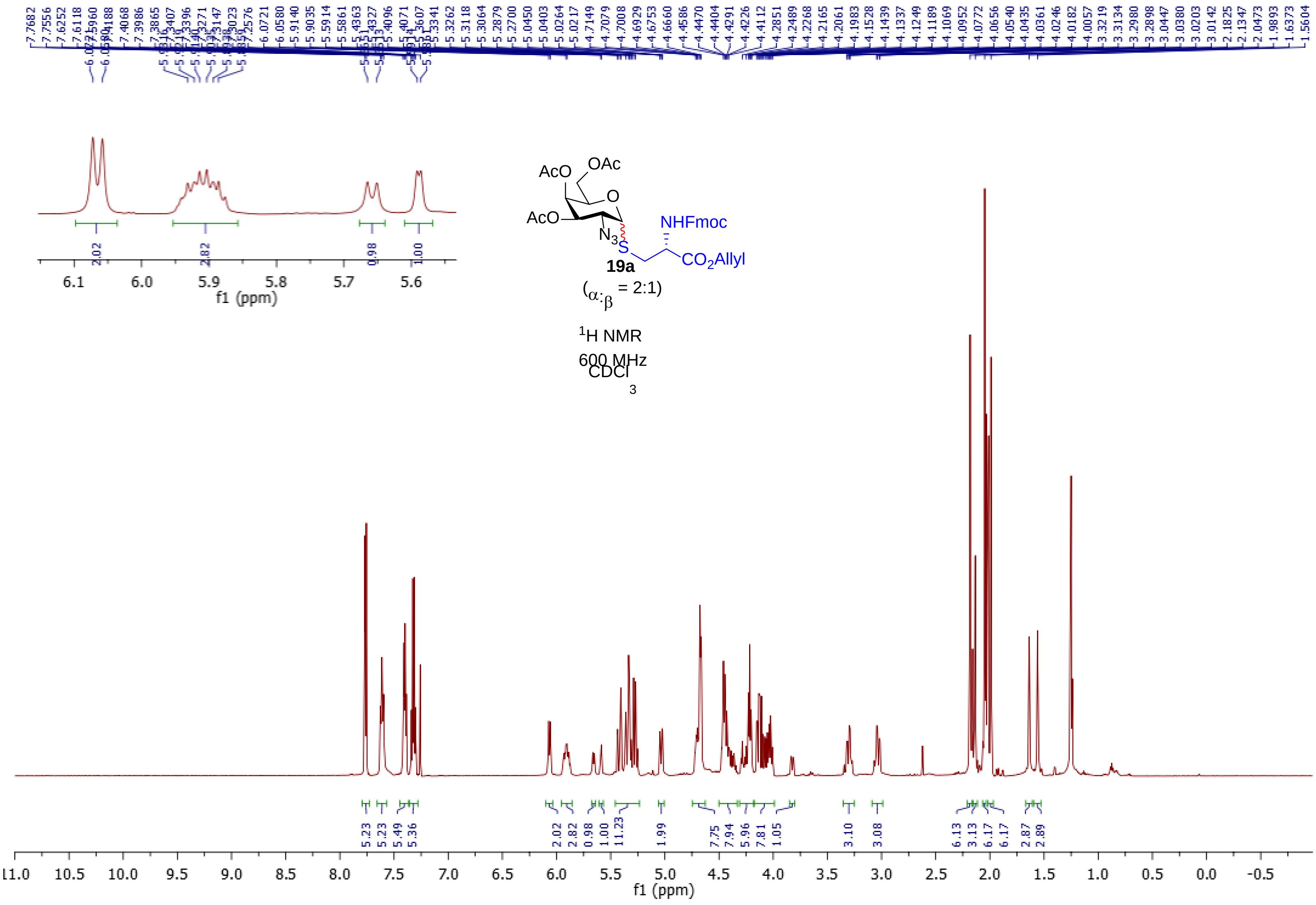

AcO
OAc
O
AcO
N3
S
NHFmoc
CO2Allyl
19a
(α:β = 2:1)
1H NMR
600 MHz
CDCl3
f1 (ppm)

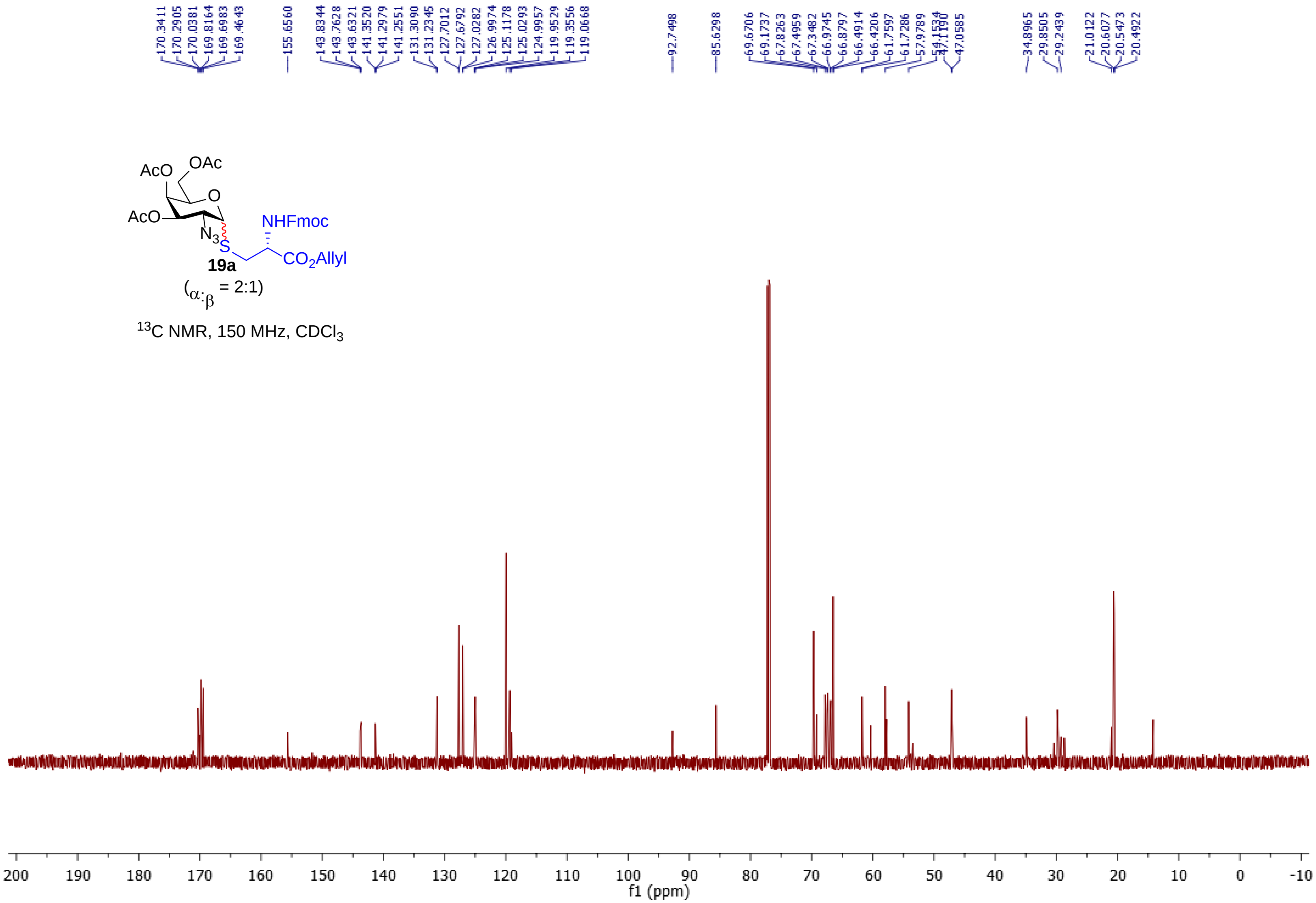

AcO
OAc
O
AcO
N3
S
NHFmoc
CO2Allyl
19a
(α:β = 2:1)
13C NMR, 150 MHz, CDCl3
170.3411
170.2905
170.0381
169.8164
169.6983
169.4643
155.6560
143.8344
143.7628
143.6321
141.3520
141.2979
141.2551
131.3090
131.2345
127.7012
127.6792
127.0282
126.9974
125.1178
125.0293
124.9957
119.9529
119.3556
119.0668
92.7498
85.6298
69.6706
69.1737
67.8263
67.4959
67.3482
66.9745
66.8797
66.4914
66.4206
61.7597
61.7286
57.9789
54.1534
47.1190
47.0585
34.8965
29.8505
29.2439
21.0122
20.6077
20.5473
20.4922
200
190
180
170
160
150
140
130
120
110
100
90
80
70
60
50
40
30
20
10
0
-10
f1 (ppm)

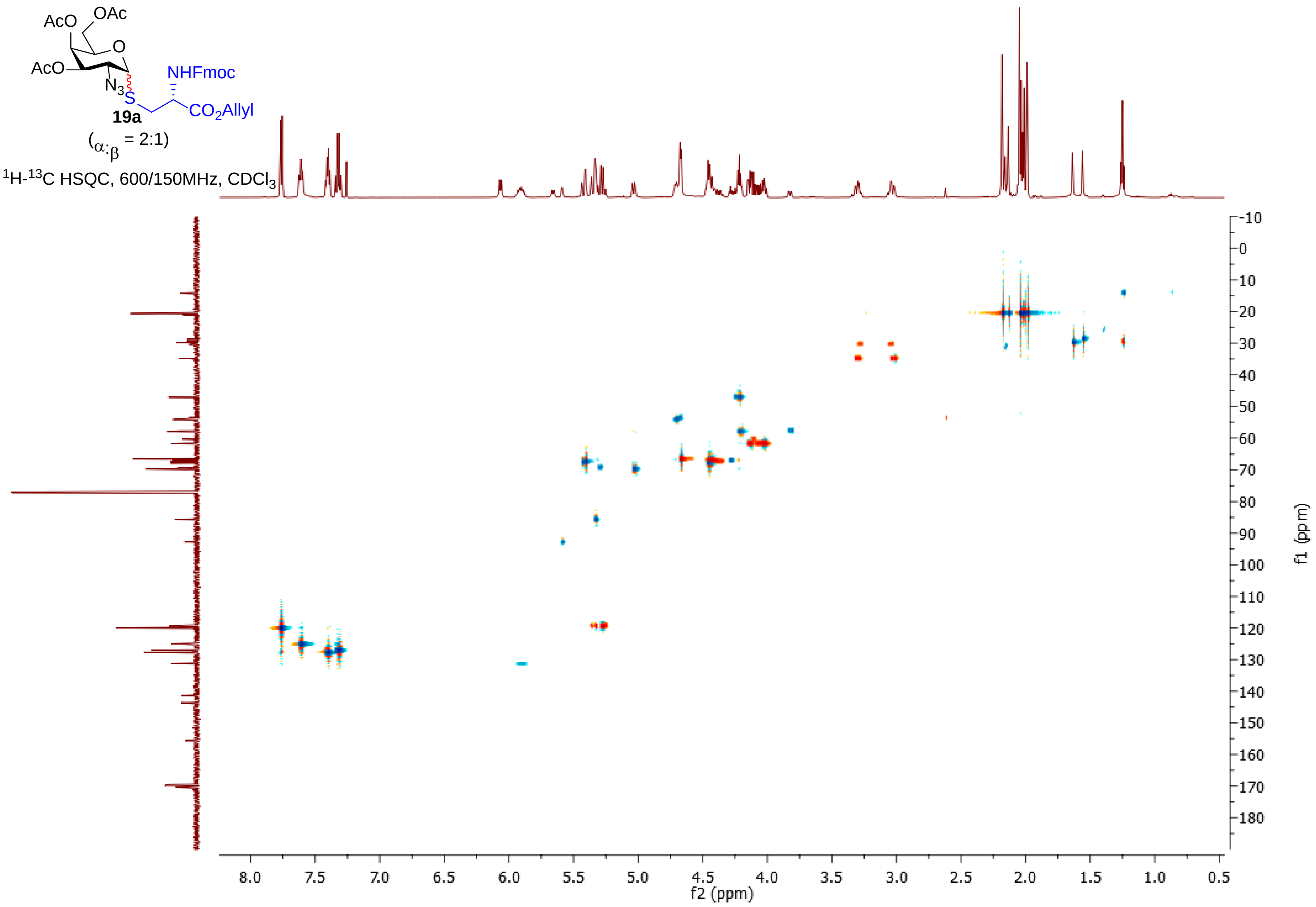
AcO
OAc
O
AcO
N3
S
NHFmoc
CO2Allyl
19a
(α:β = 2:1)
1H-13C HSQC, 600/150MHz, CDCl3
f2 (ppm)
f1 (ppm)

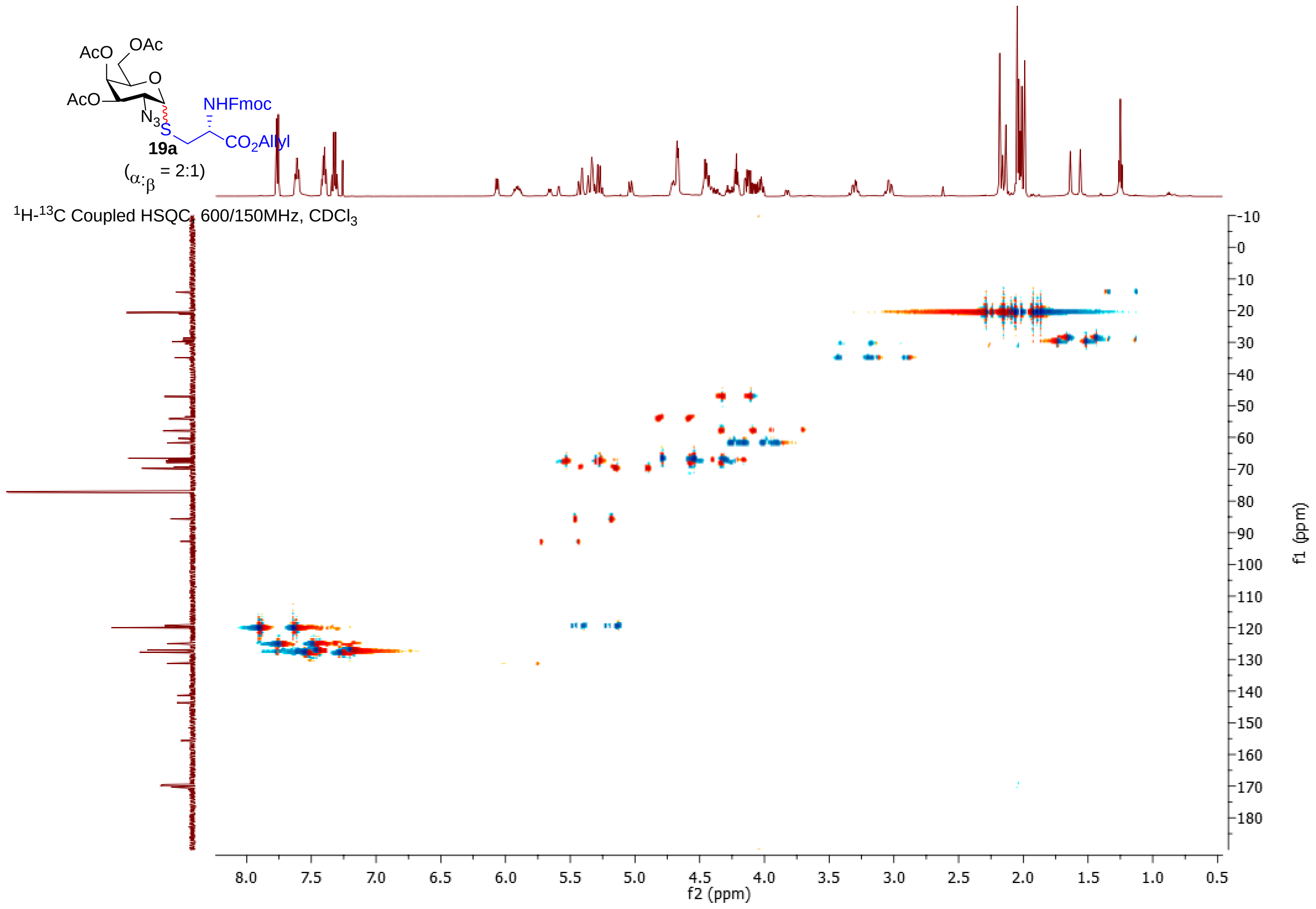
AcO
OAc
O
AcO
N3
S
NHFmoc
CO2Allyl
19a
(α:β = 2:1)
1H-13C Coupled HSQC, 600/150MHz, CDCl3
f1 (ppm)
f2 (ppm)

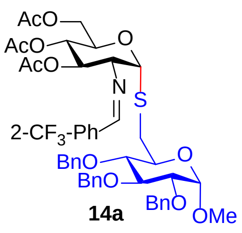

14a

^{1}H NMR
600 MHz
$CDCl_3$

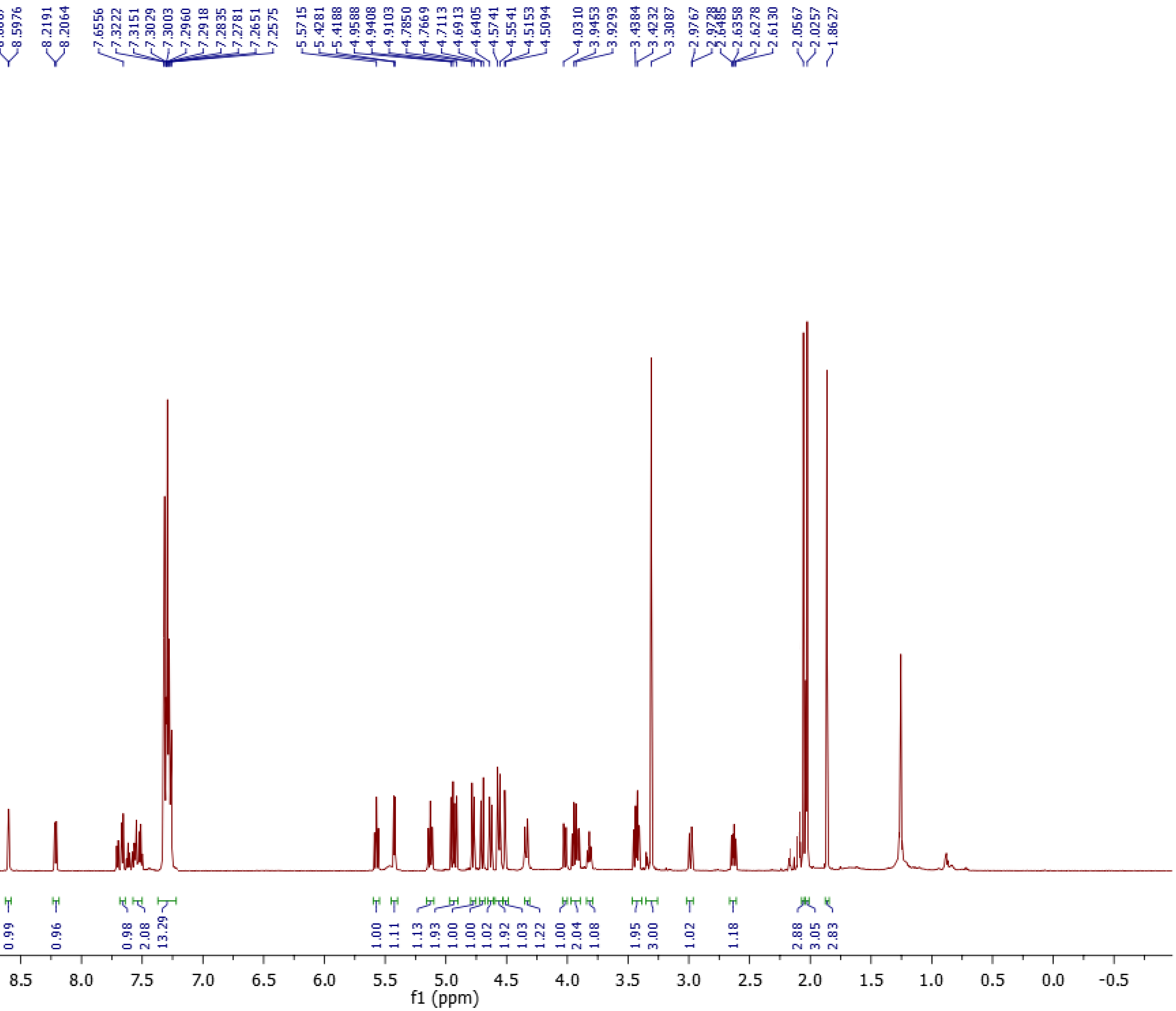

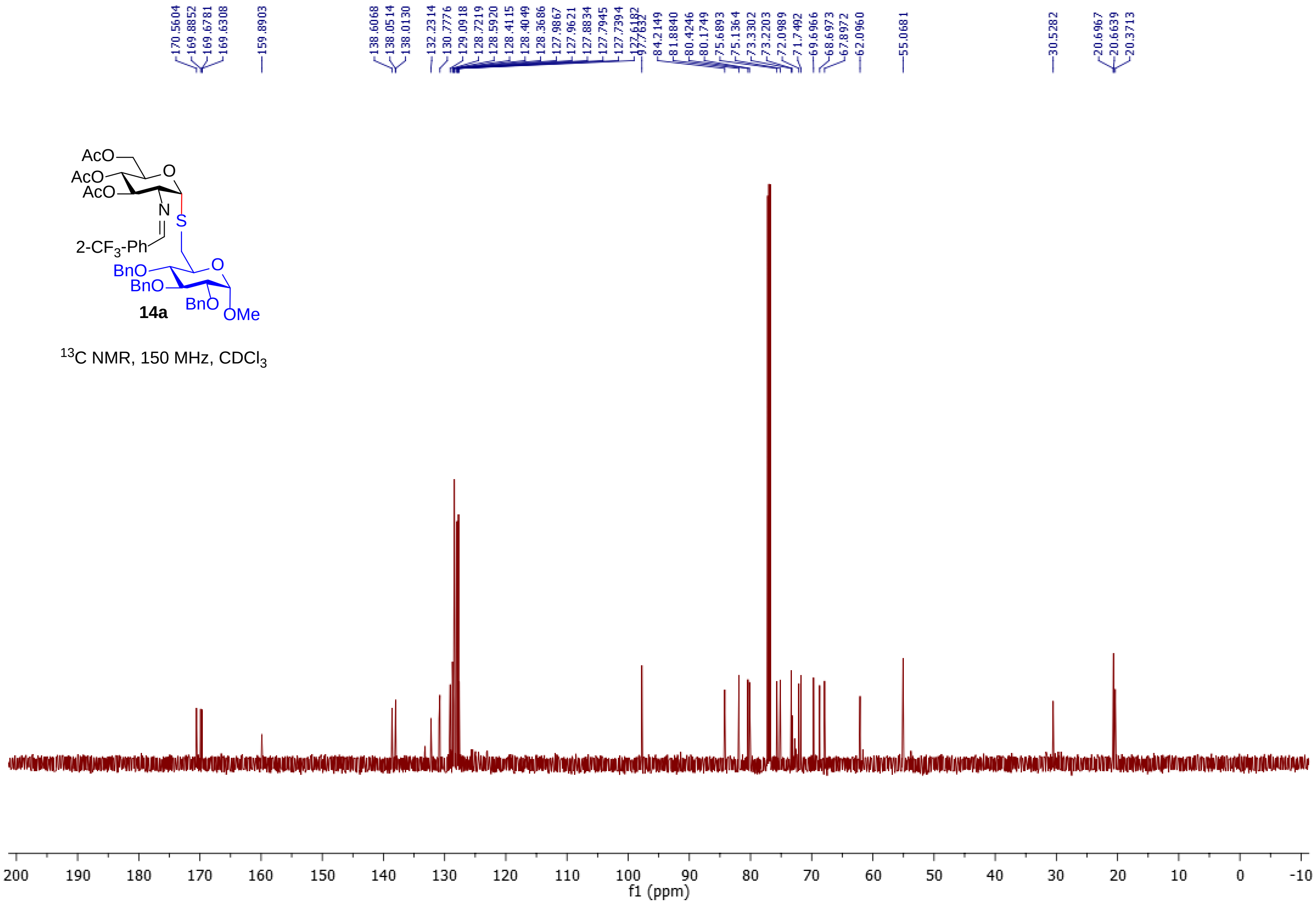

14a

^{13}C NMR, 150 MHz, $CDCl_3$

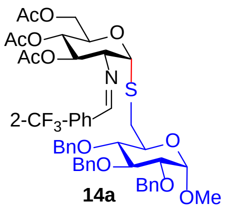

14a

^{1}H-^{13}C HSQC, 600/150MHz, $CDCl_3$

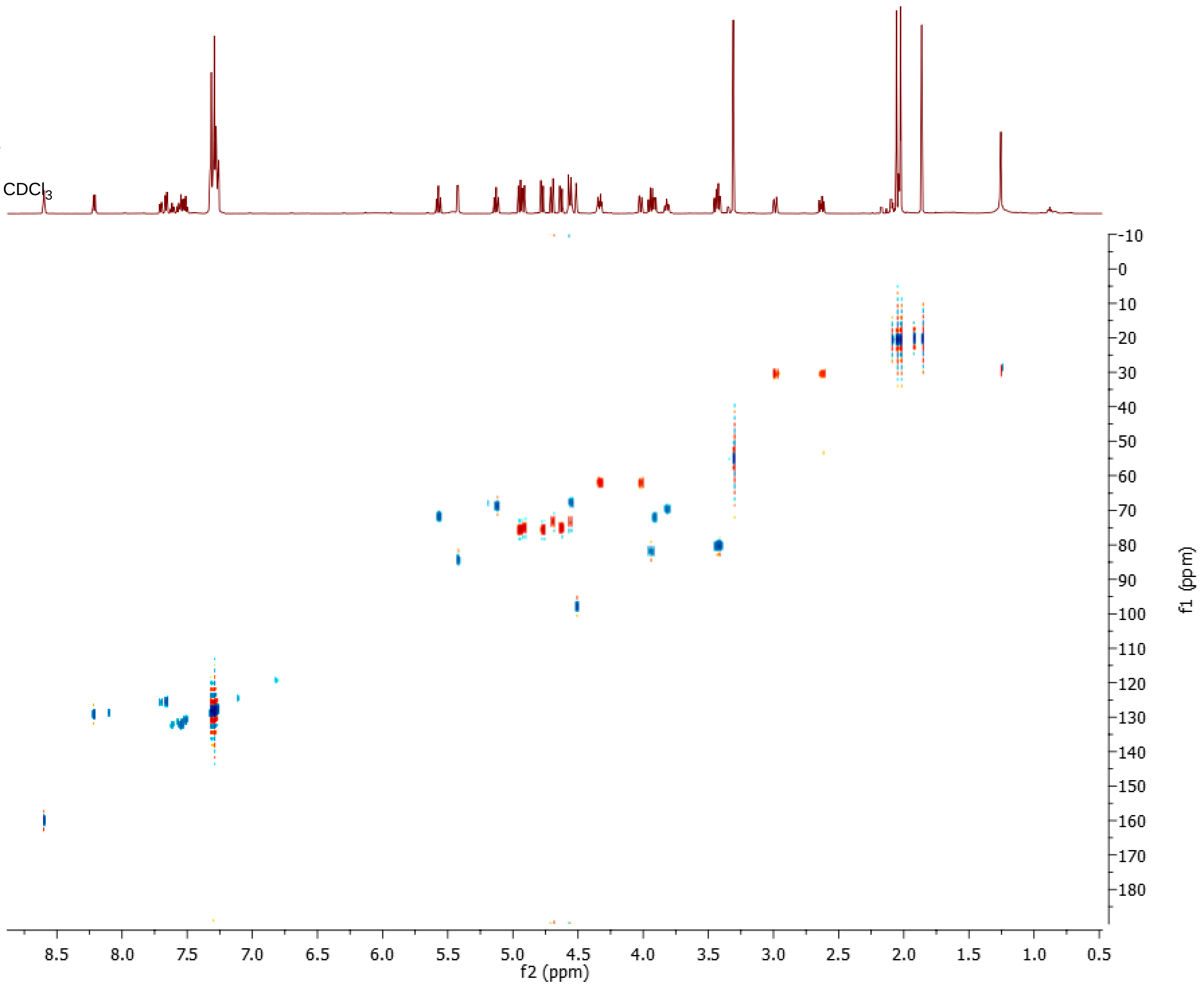

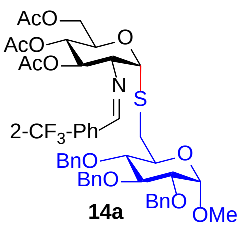

14a

^{1}H-^{13}C Coupled HSQC, 600/150MHz, $CDCl_3$

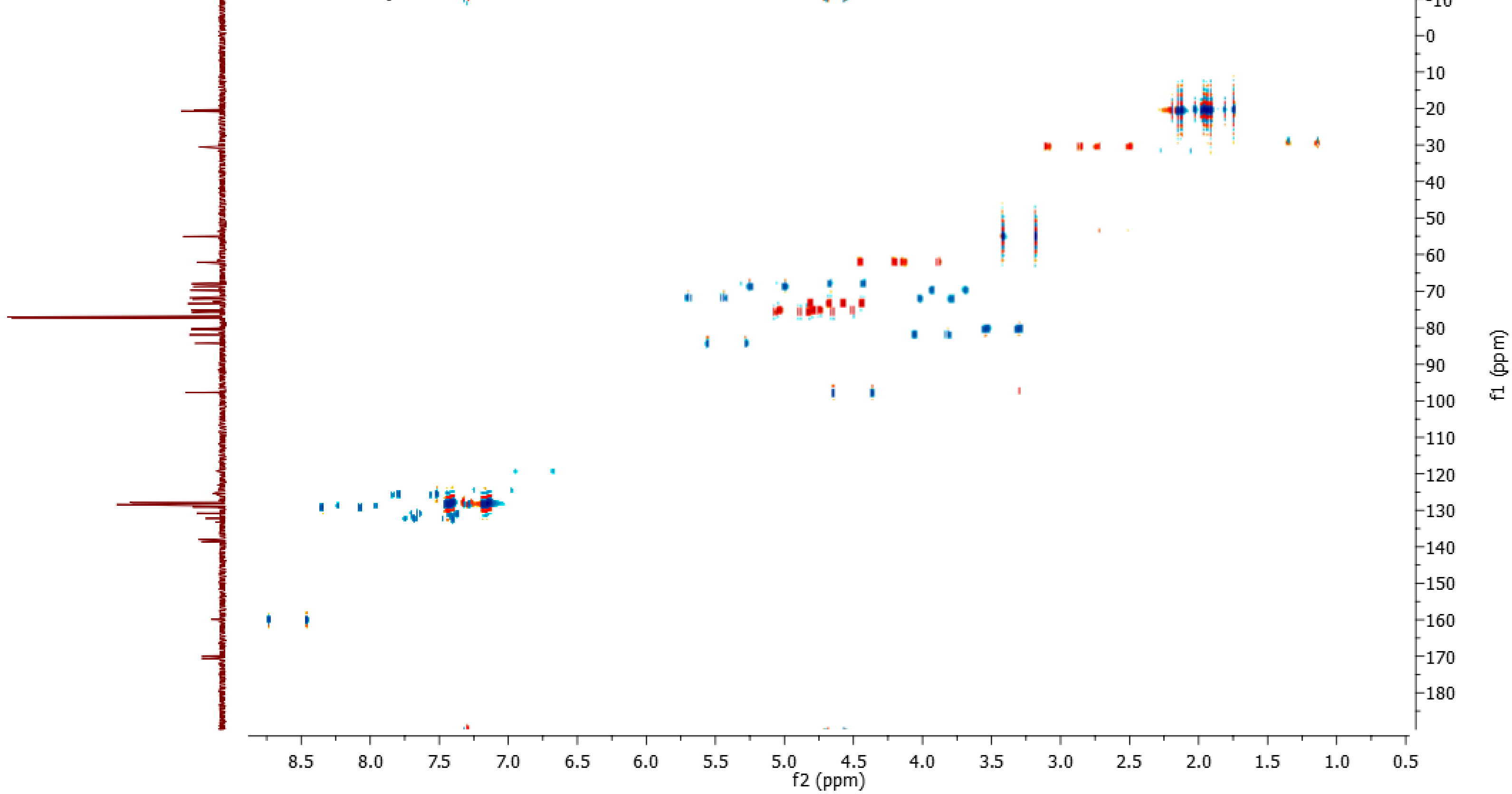

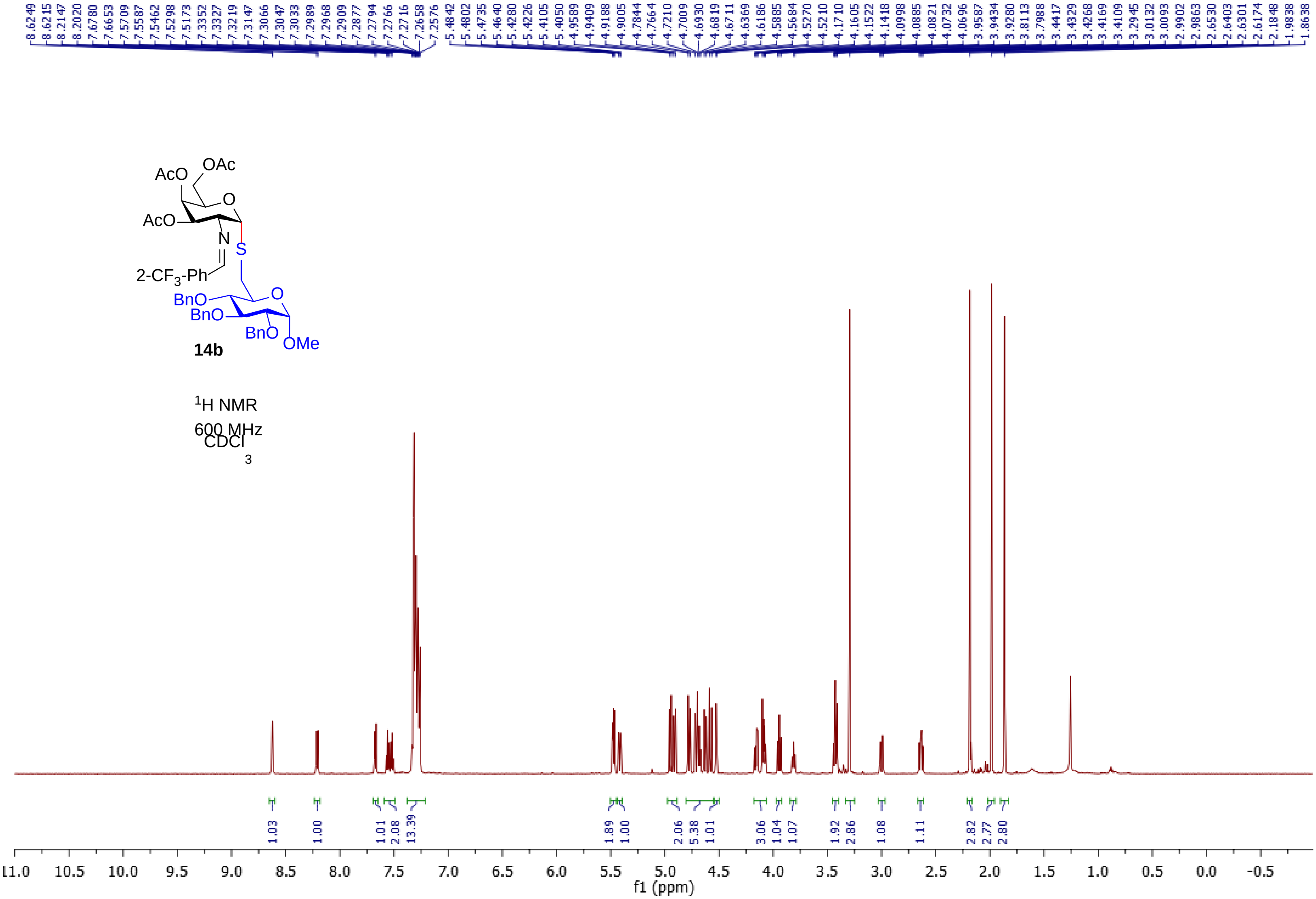

14b

^{1}H NMR
600 MHz
$CDCl_3$

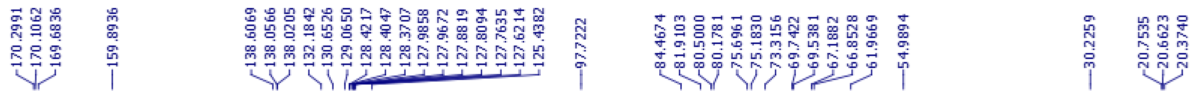

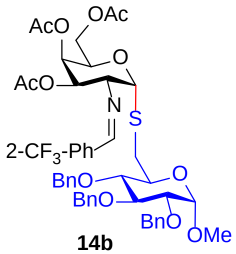

14b

^{13}C NMR, 150 MHz, $CDCl_3$

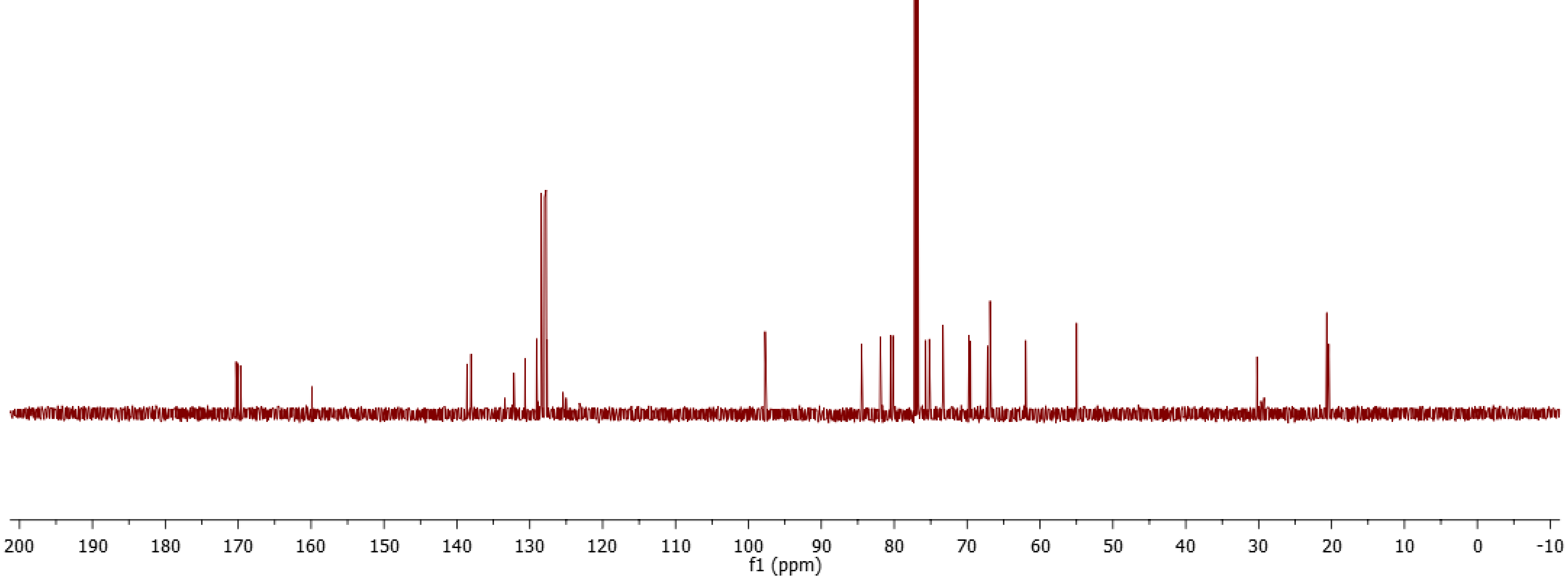

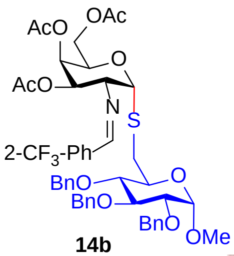
AcO
OAc
O
AcO
N
S
2-CF3-Ph
BnO
O
BnO
BnO
OMe

14b

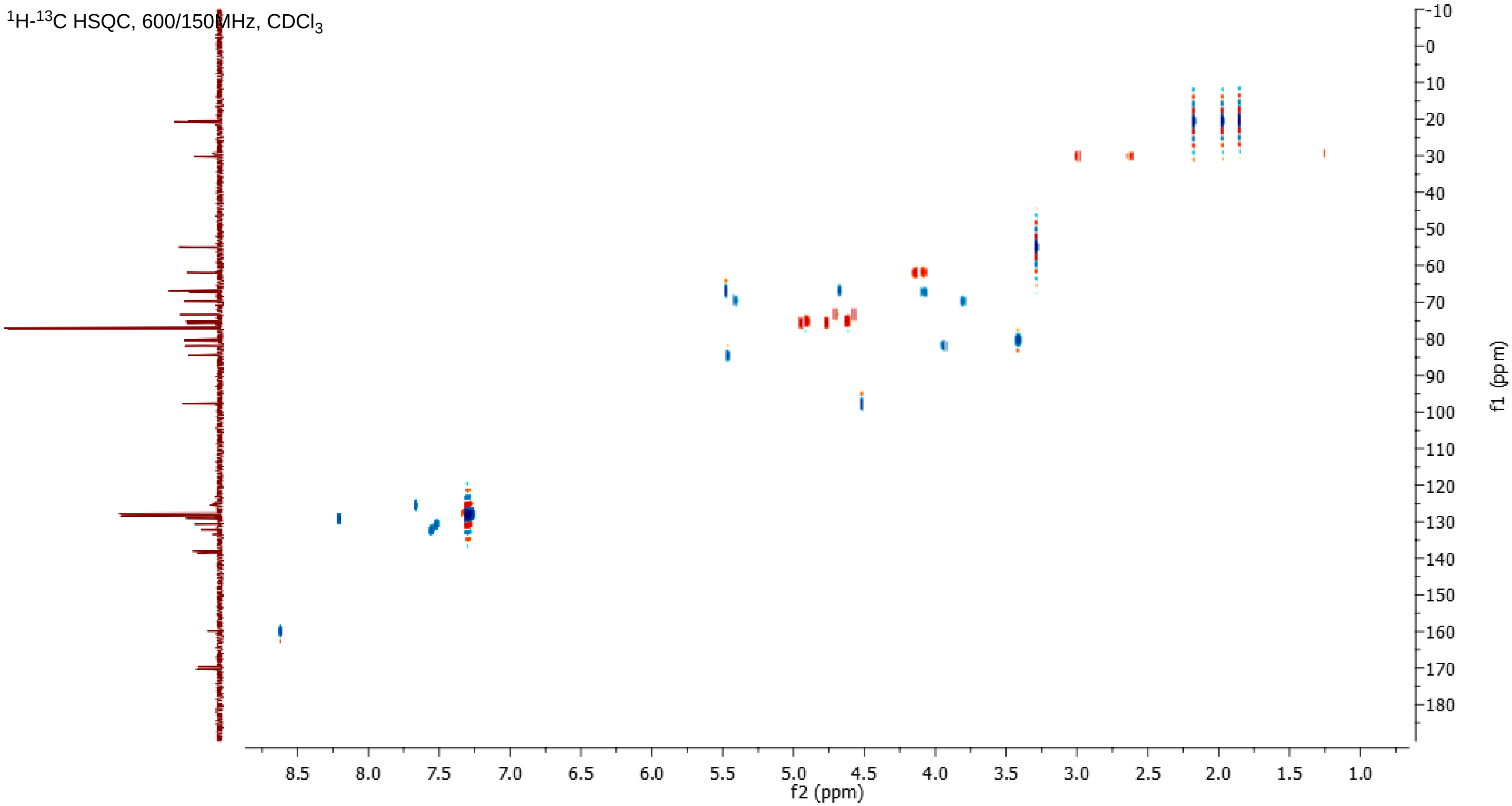
^{1}H-^{13}C HSQC, 600/150MHz, CDCl$_3$
f2 (ppm)
f1 (ppm)

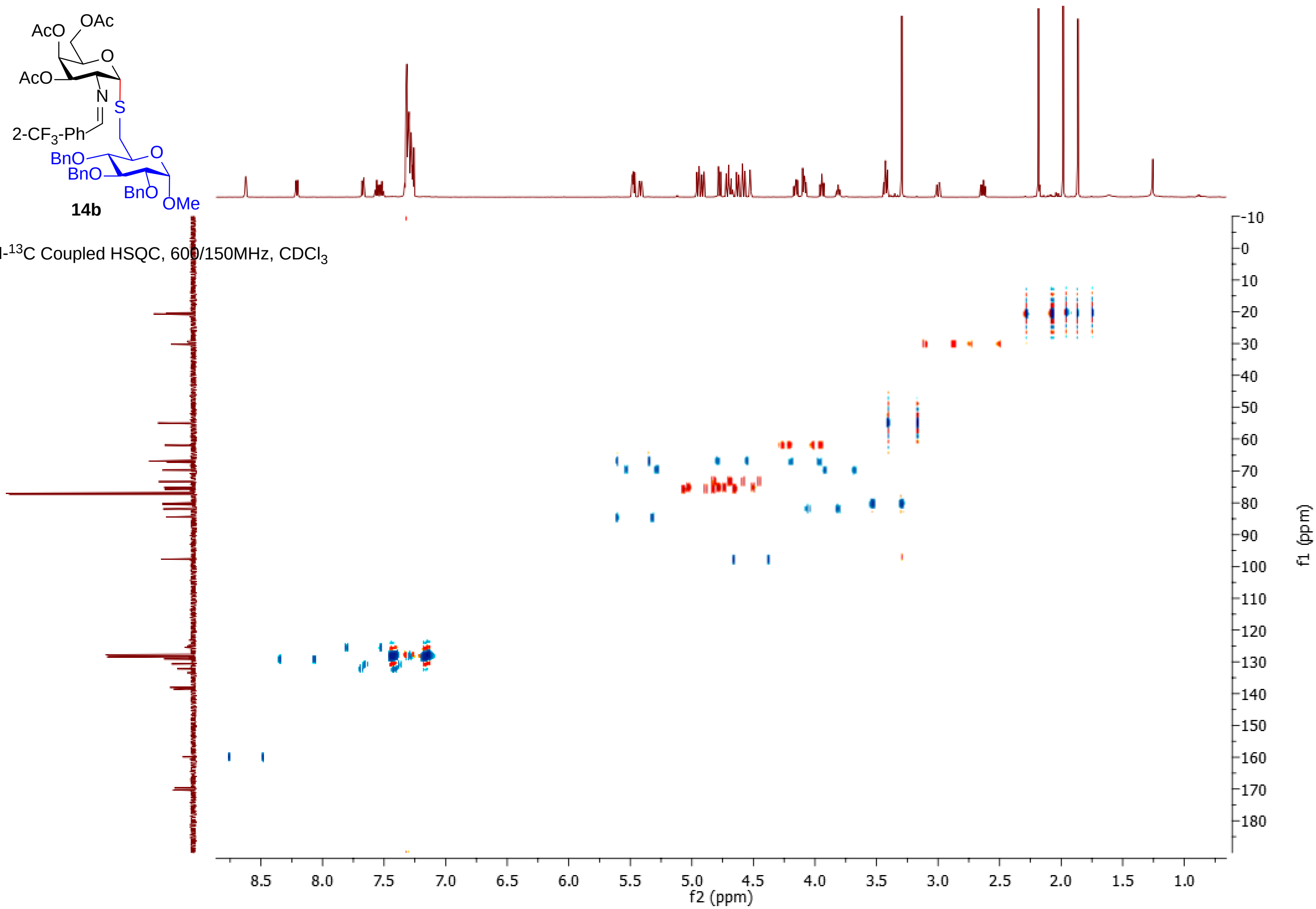

14b

^{1}H-^{13}C Coupled HSQC, 600/150MHz, $CDCl_3$

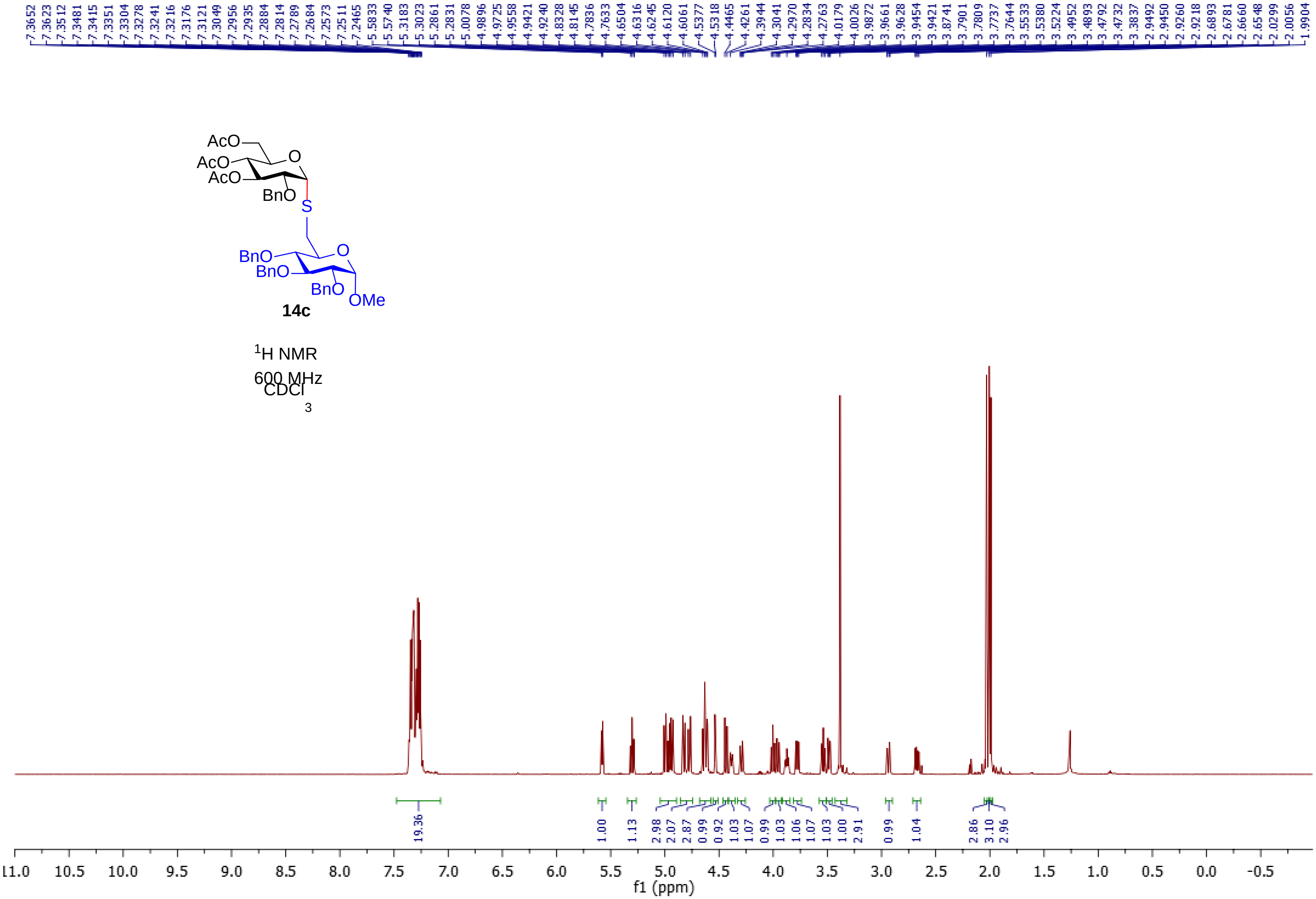

7.3652 7.3623 7.3512 7.3481 7.3415 7.3351 7.3304 7.3278 7.3241 7.3216 7.3176 7.3121 7.3049 7.2956 7.2935 7.2884 7.2814 7.2789 7.2684 7.2573 7.2511 7.2465 5.5833 5.5740 5.3183 5.3023 5.2861 5.2831 5.0078 4.9896 4.9725 4.9558 4.9421 4.9240 4.8328 4.8145 4.7836 4.7633 4.6504 4.6316 4.6245 4.6120 4.6061 4.5377 4.5318 4.4465 4.4261 4.3944 4.3041 4.2970 4.2834 4.2763 4.0179 4.0026 3.9872 3.9661 3.9628 3.9454 3.9421 3.8741 3.7901 3.7809 3.7737 3.7644 3.5533 3.5380 3.5224 3.4952 3.4893 3.4792 3.4732 3.3837 2.9492 2.9450 2.9260 2.9218 2.6893 2.6781 2.6660 2.6548 2.0299 2.0056 1.9904
AcO
AcO
AcO
O
BnO
S
BnO
BnO
BnO
O
OMe
14c
^{1}H NMR
600 MHz
CDCl$_3$
19.36 1.00 1.13 2.98 2.07 2.87 0.99 0.92 1.03 1.07 0.99 1.03 1.06 1.07 1.03 1.00 2.91 0.99 1.04 2.86 3.10 2.96
11.0 10.5 10.0 9.5 9.0 8.5 8.0 7.5 7.0 6.5 6.0 5.5 5.0 4.5 4.0 3.5 3.0 2.5 2.0 1.5 1.0 0.5 0.0 -0.5
f1 (ppm)

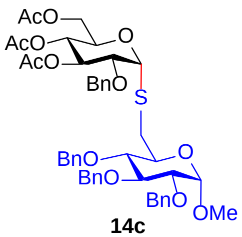

14c

^{13}C NMR, 150 MHz, $CDCl_3$

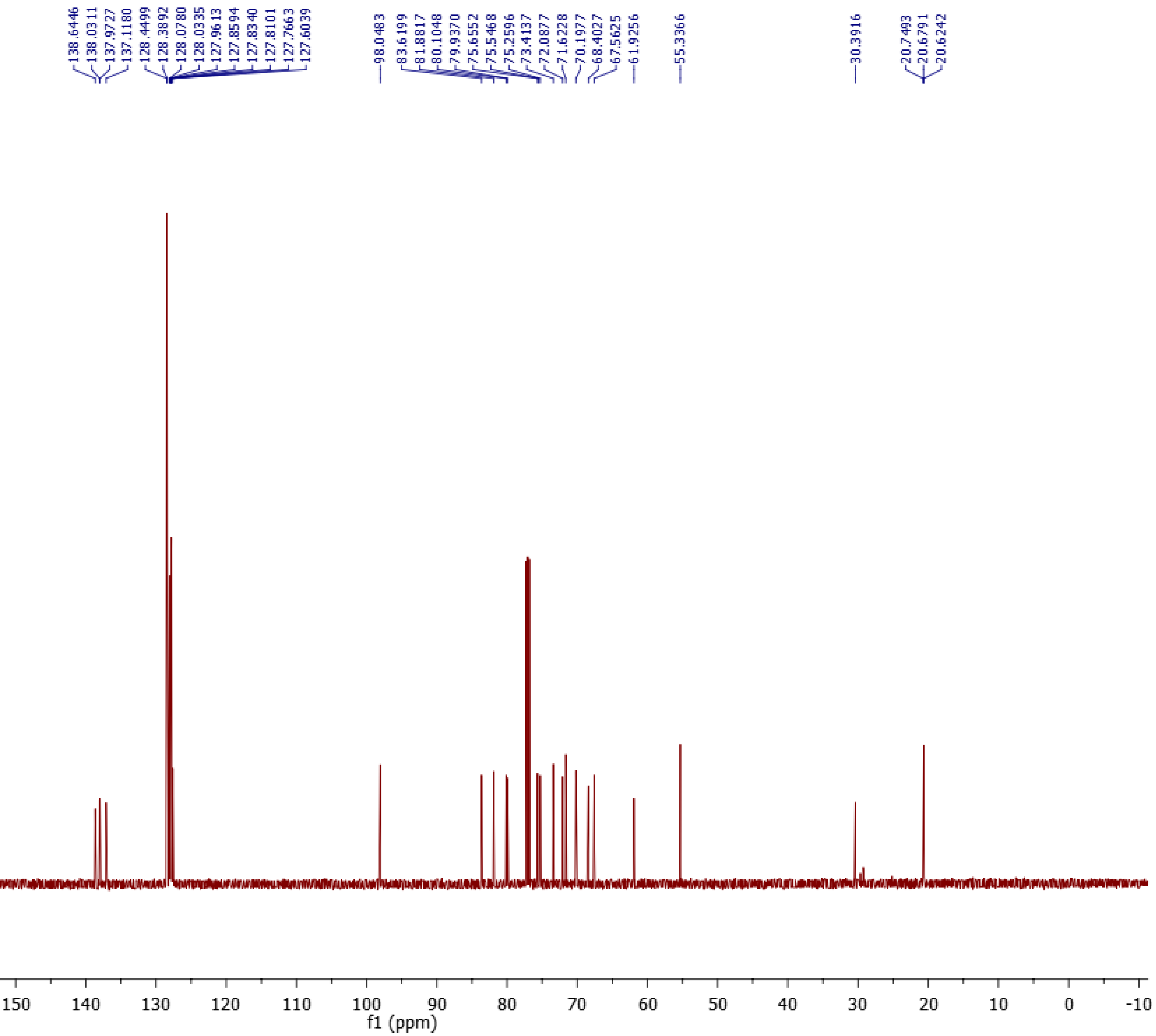

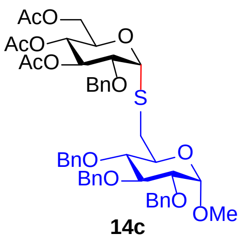

14c

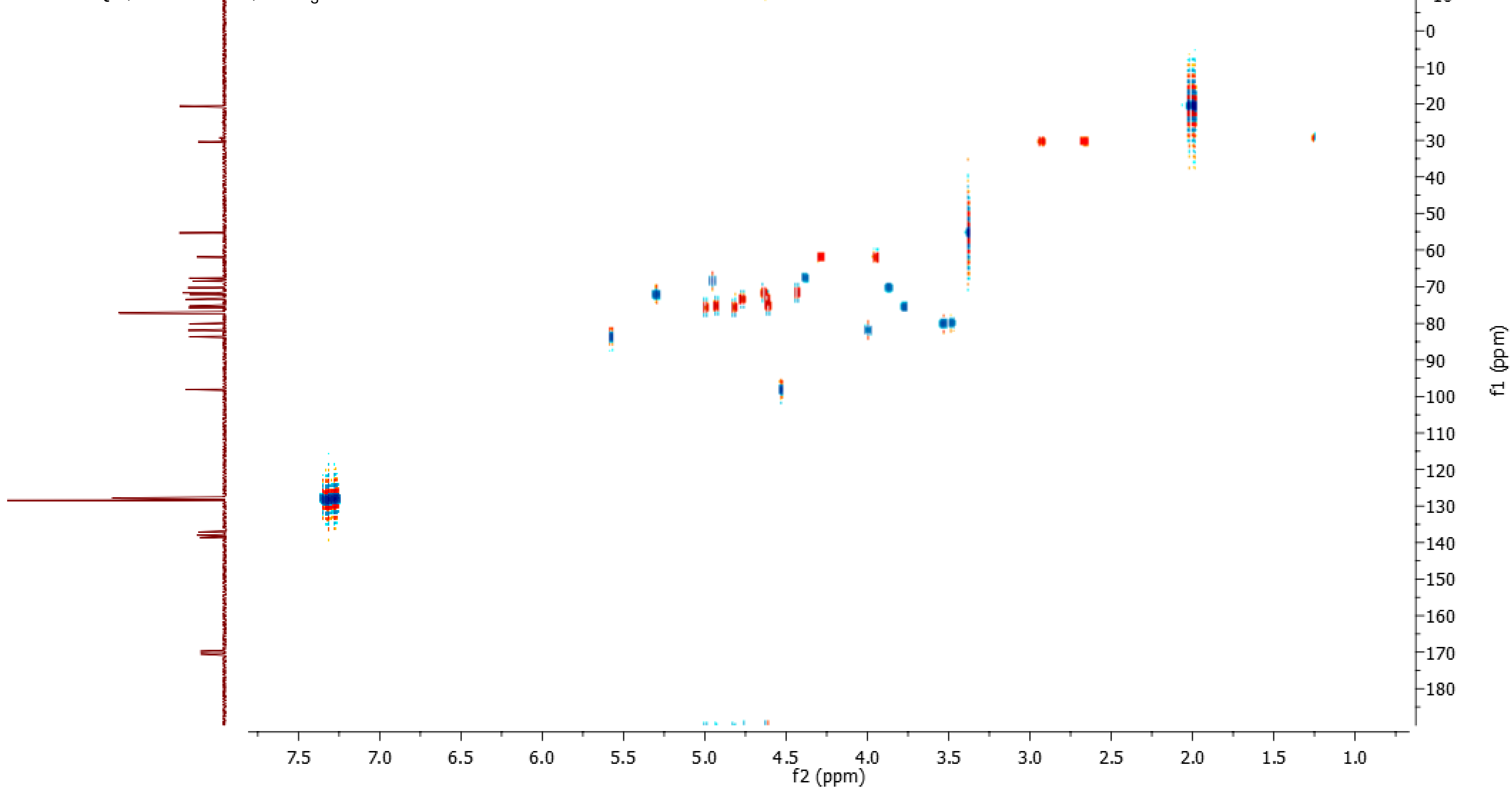

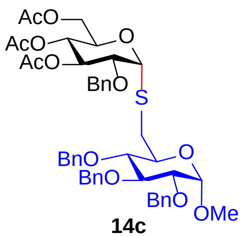

14c

^1H-^{13}C Coupled HSQC, 600/150MHz, $CDCl_3$

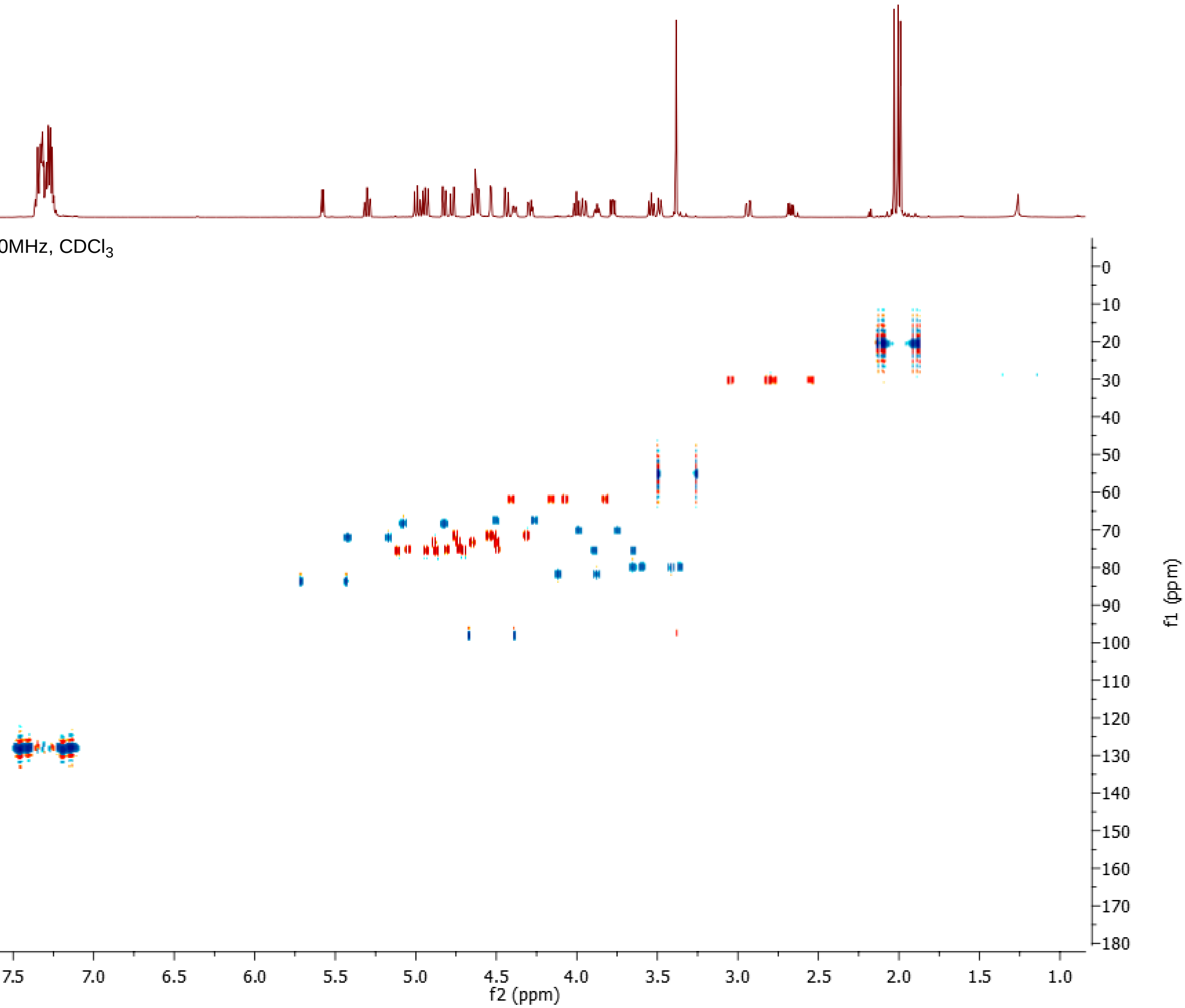

15a

^{1}H NMR

600 MHz

$CDCl_3$

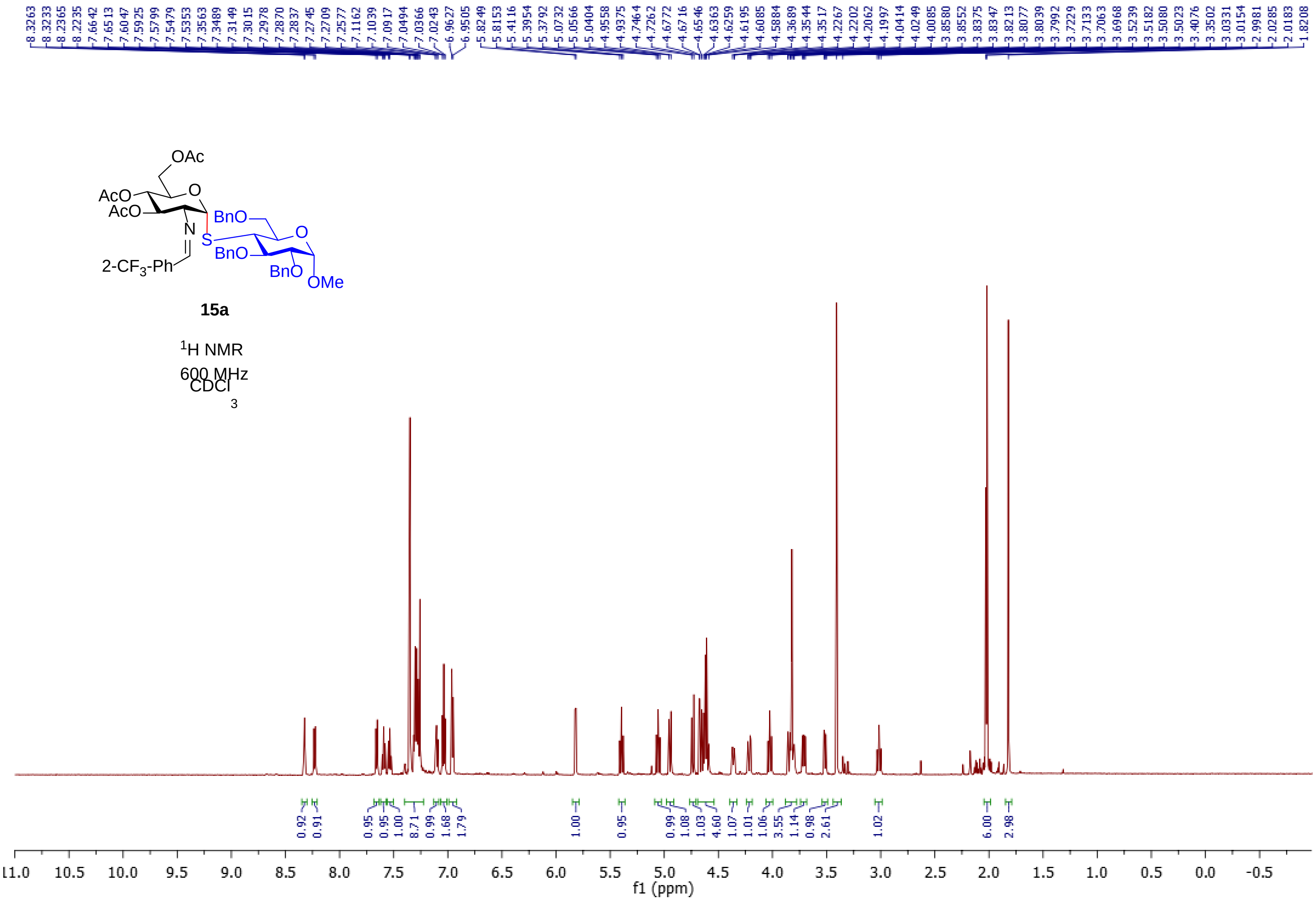

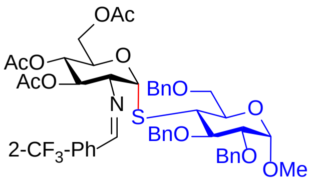

15a

^{13}C NMR, 150 MHz, $CDCl_3$

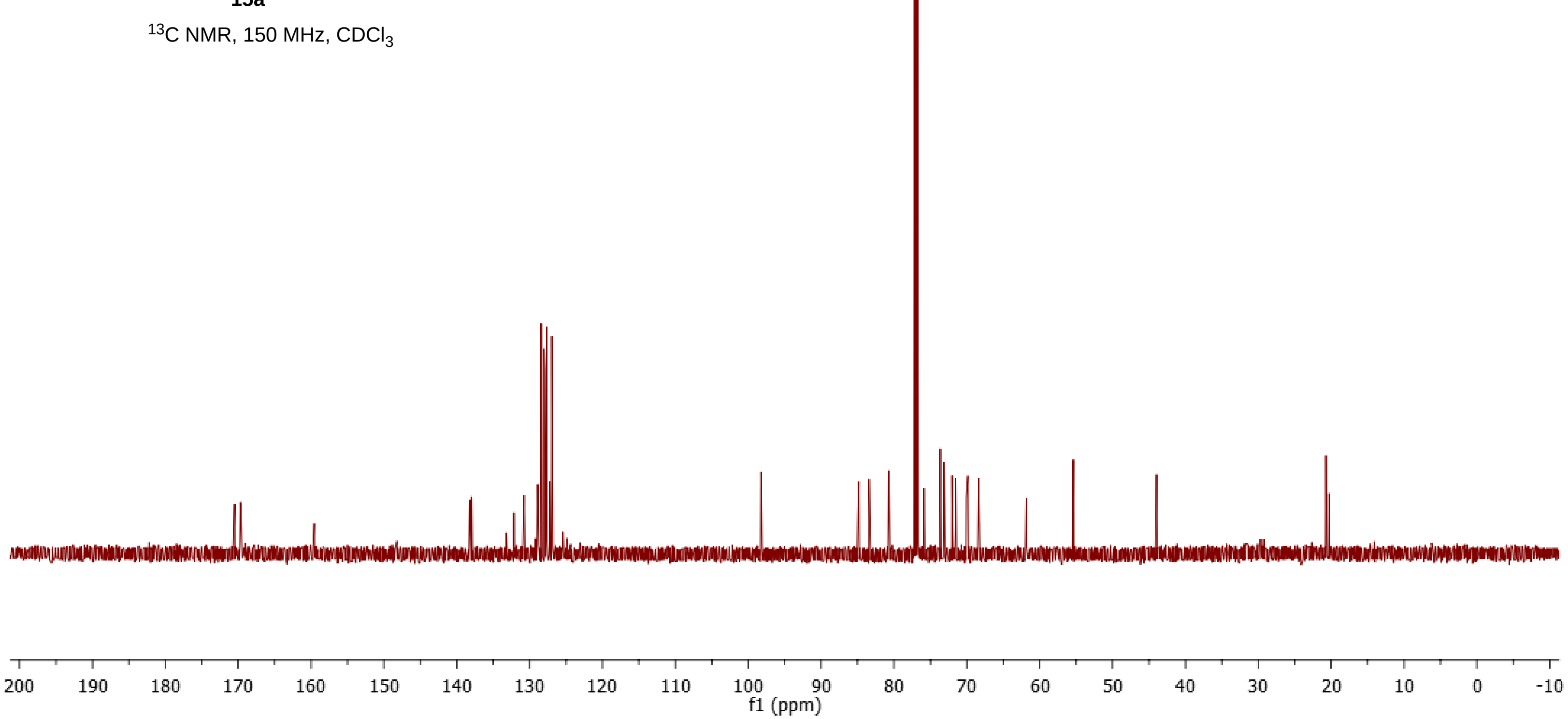

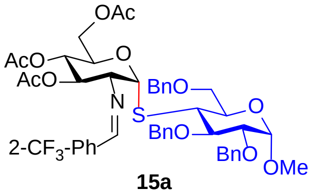

15a

^{1}H-^{13}C HSQC, 600/150MHz, $CDCl_3$

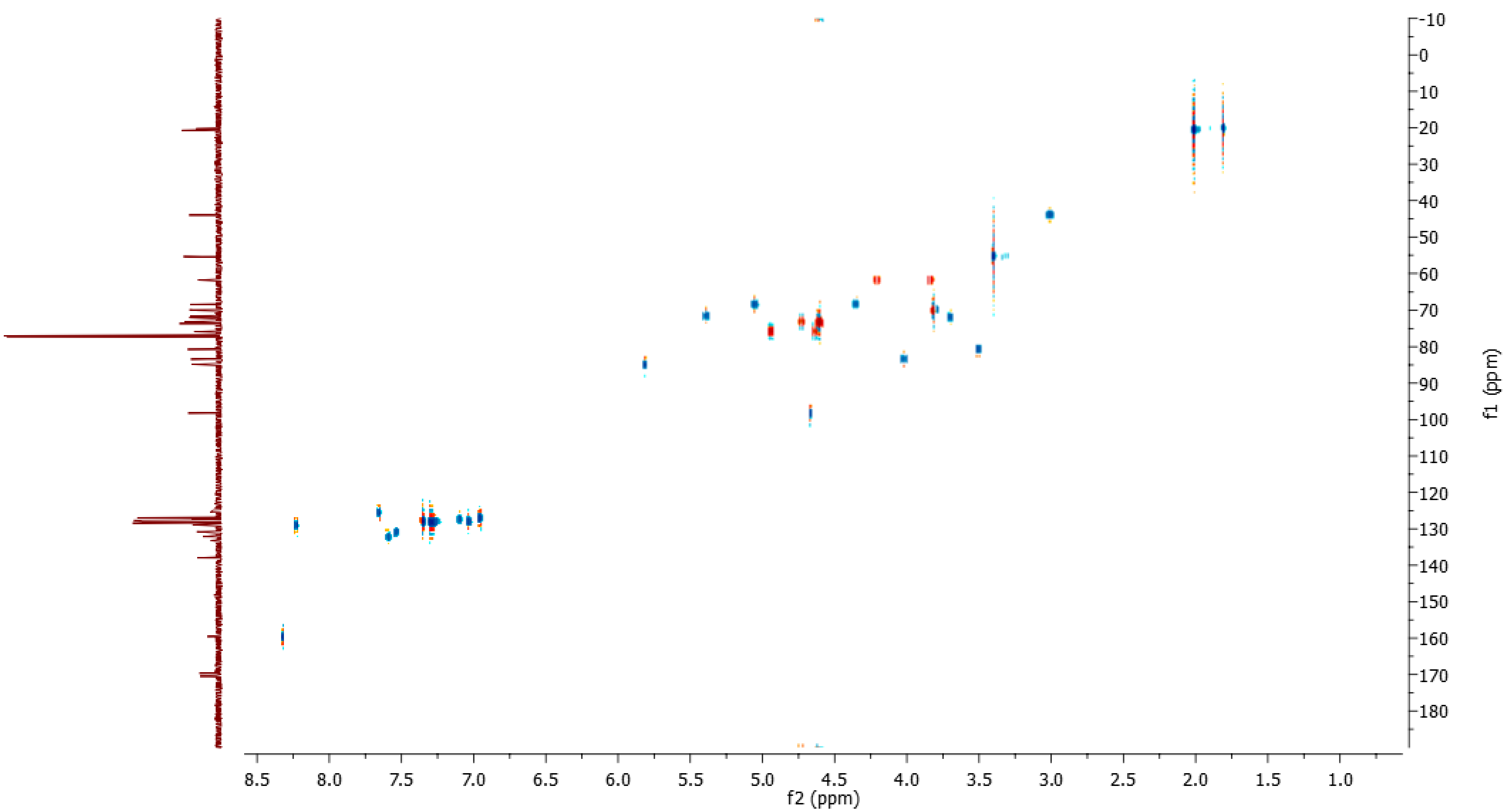

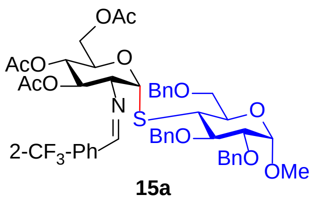

15a

^{1}H-^{13}C Coupled HSQC, 600/150MHz, $CDCl_3$

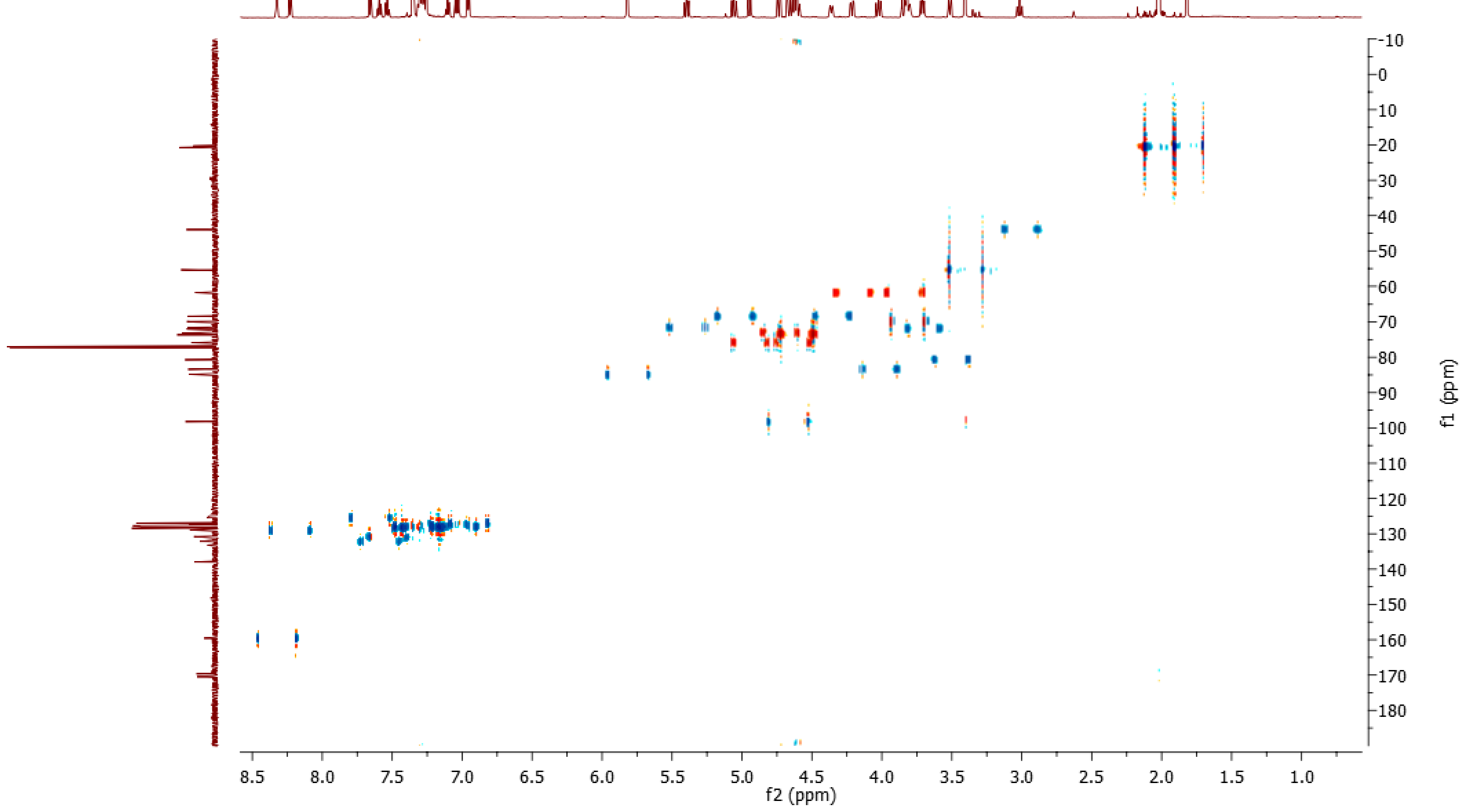

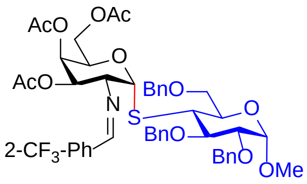

15b

^{1}H NMR
600 MHz
CDCl3

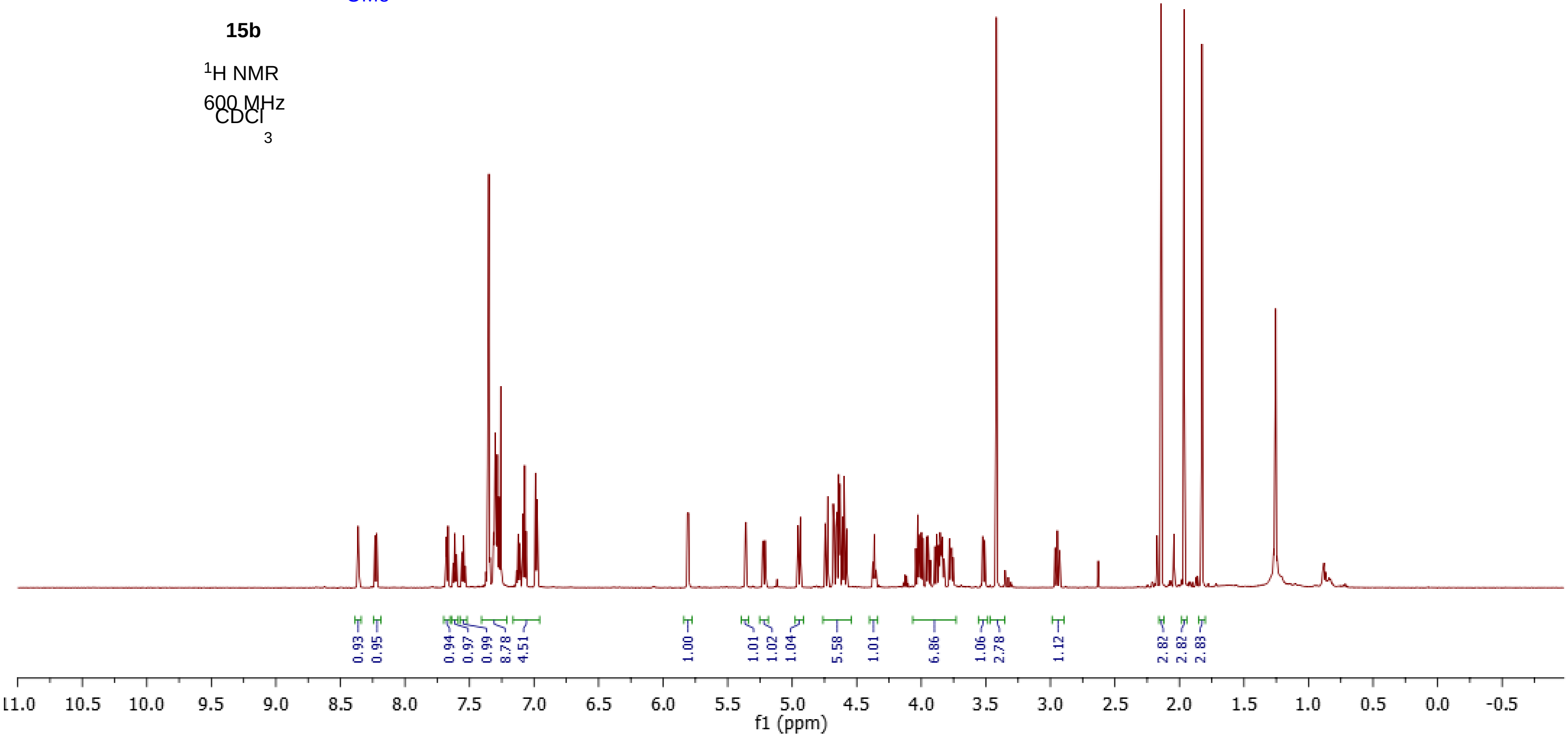

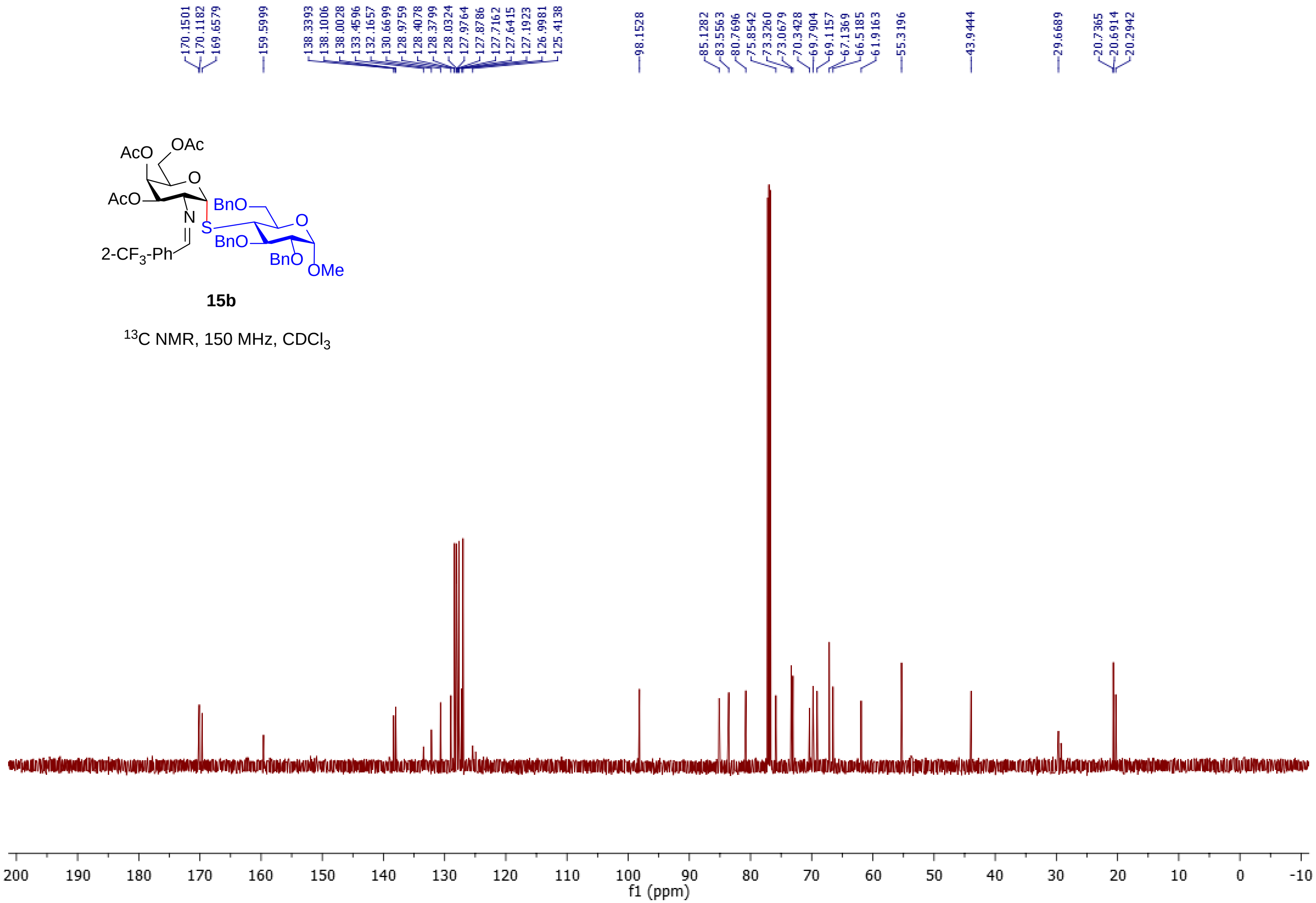
170.1501
170.1182
169.6579
159.5999
138.3393
138.1006
138.0028
133.4596
132.1657
130.6699
128.9759
128.4078
128.3799
128.0324
127.9764
127.8786
127.7162
127.6415
127.1923
126.9981
125.4138
98.1528
85.1282
83.5563
80.7696
75.8542
73.3260
73.0679
70.3428
69.7904
69.1157
67.1369
66.5185
61.9163
55.3196
43.9444
29.6689
20.7365
20.6914
20.2942
AcO
OAc
O
AcO
N
S
BnO
O
BnO
BnO
OMe
2-CF3-Ph
15b
13C NMR, 150 MHz, CDCl3
200 190 180 170 160 150 140 130 120 110 100 90 80 70 60 50 40 30 20 10 0 -10
f1 (ppm)

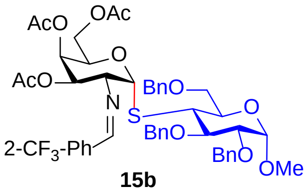

15b

^{1}H-^{13}C HSQC, 600/150MHz, $CDCl_3$

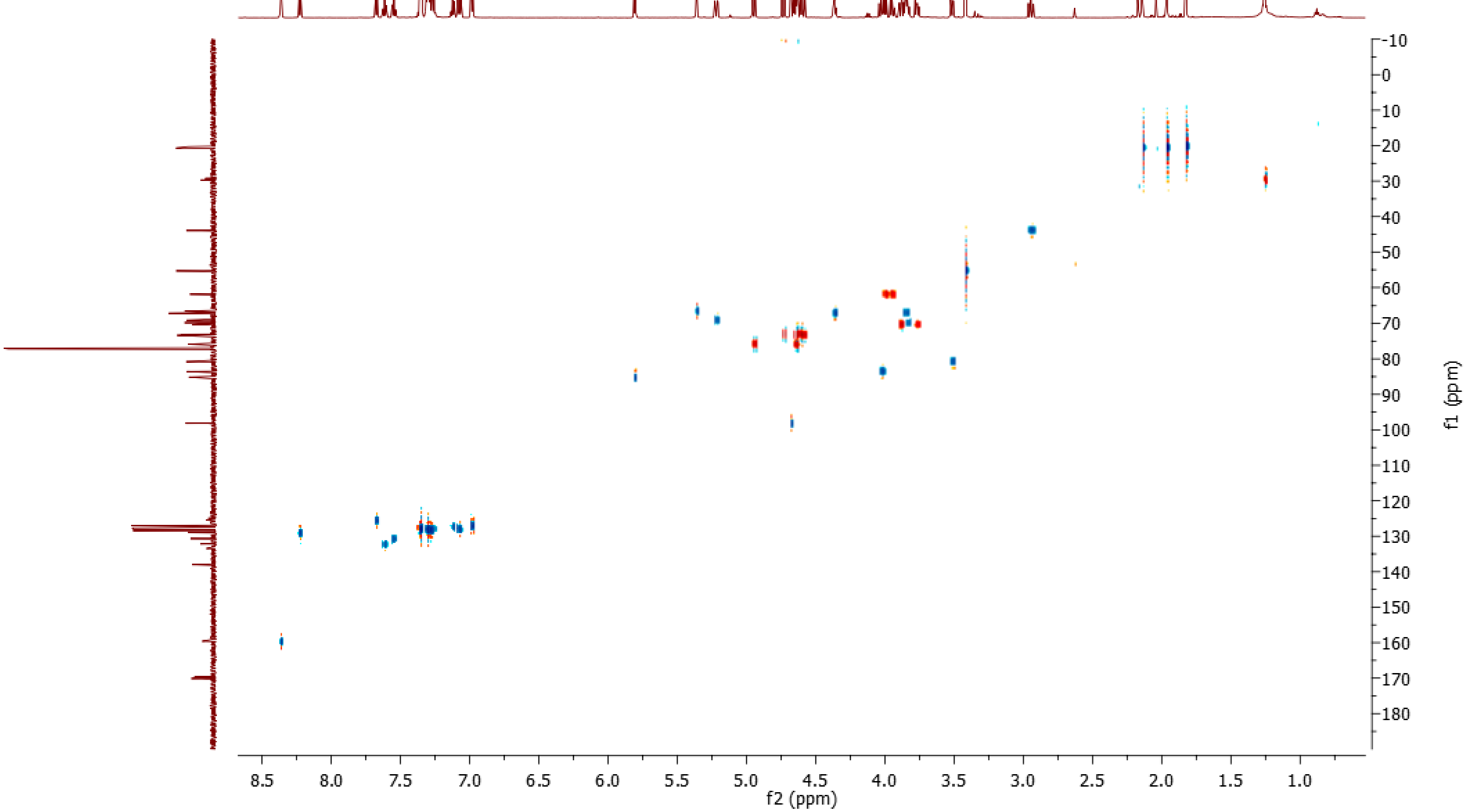

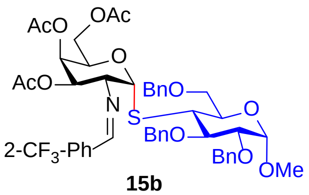

15b

^{1}H-^{13}C Coupled HSQC, 600/150MHz, $CDCl_3$

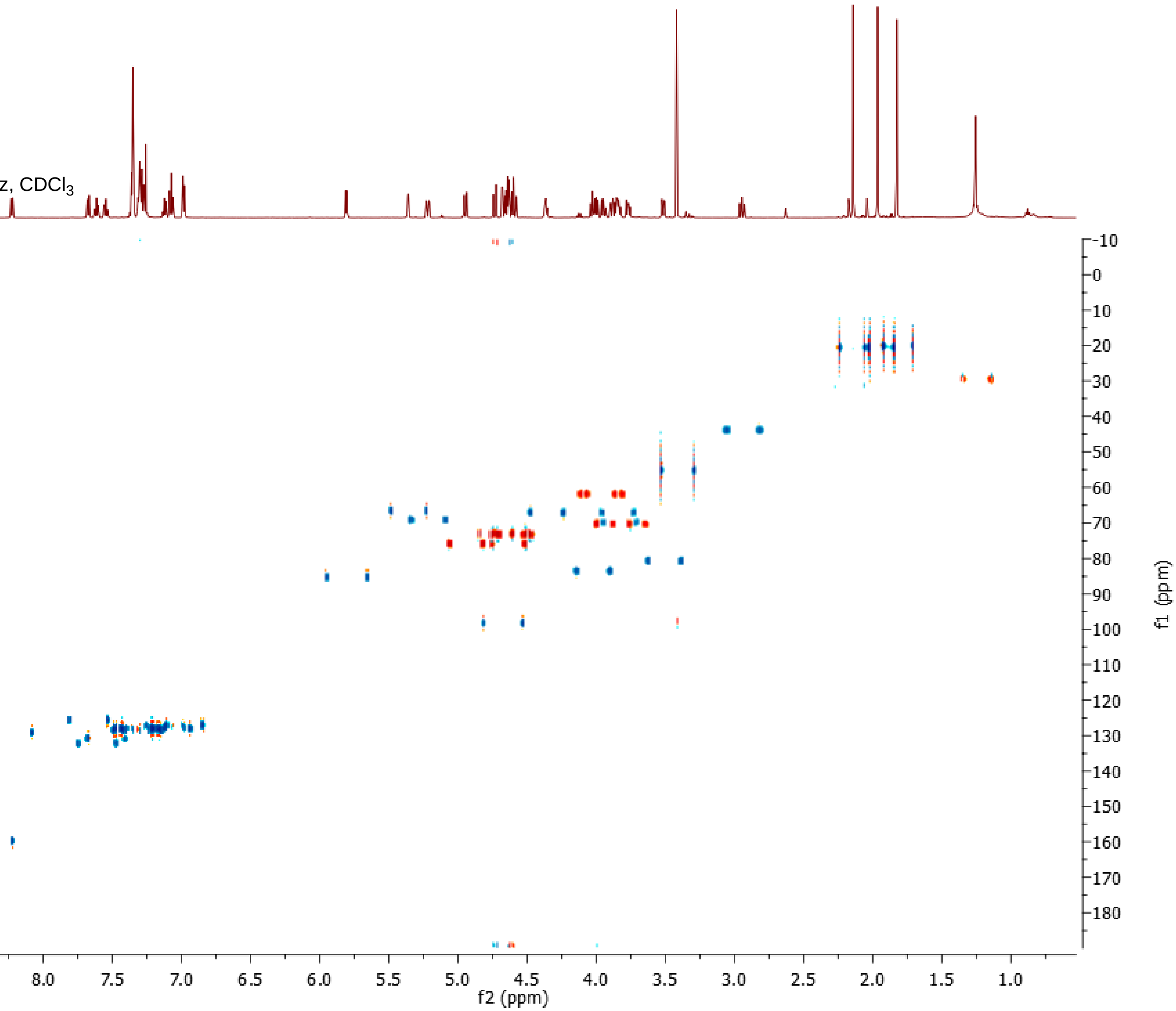

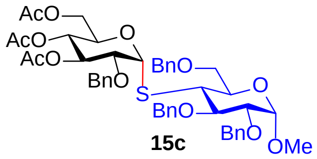

15c

^{1}H NMR
600 MHz
$CDCl_3$

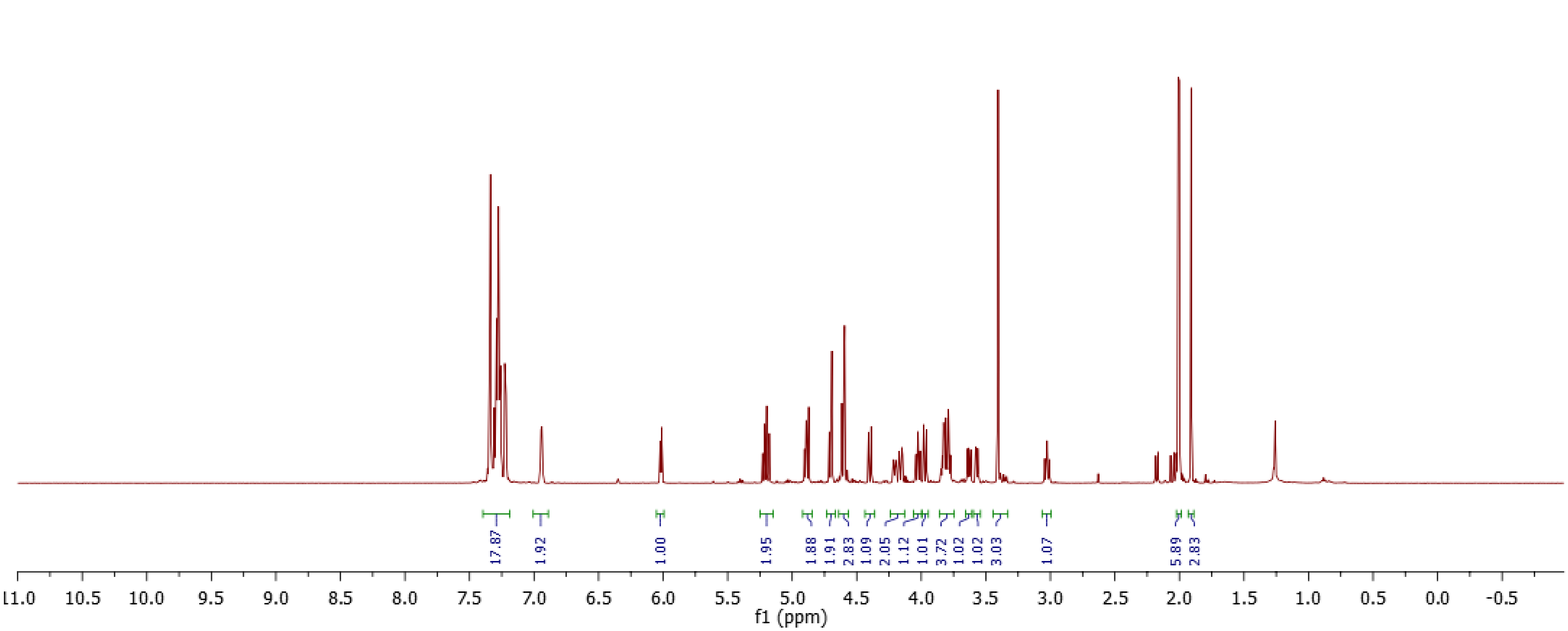

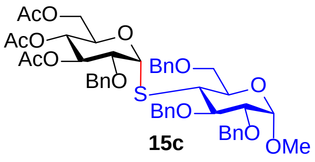

15c

^{13}C NMR, 150 MHz, $CDCl_3$

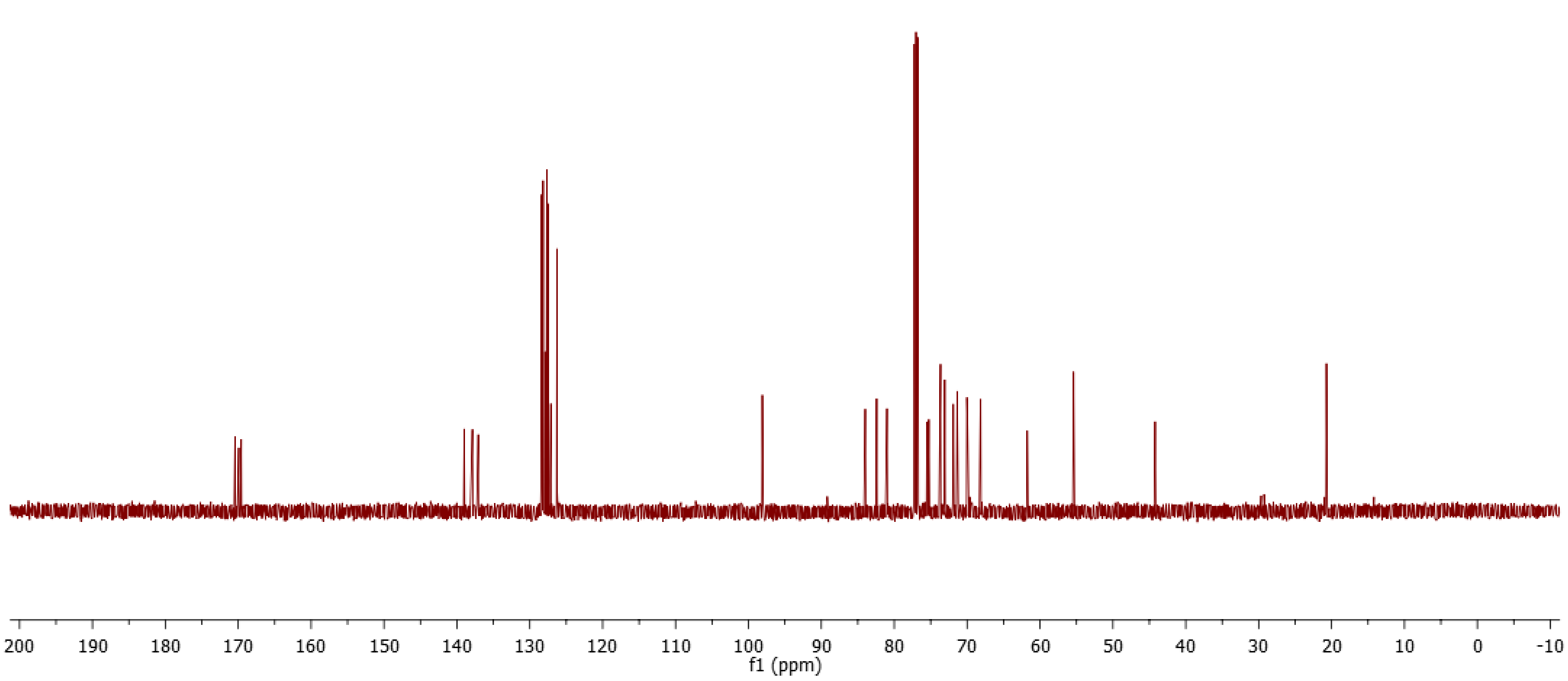

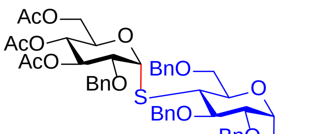

15c

^{1}H-^{13}C HSQC, 600/150MHz, $CDCl_3$

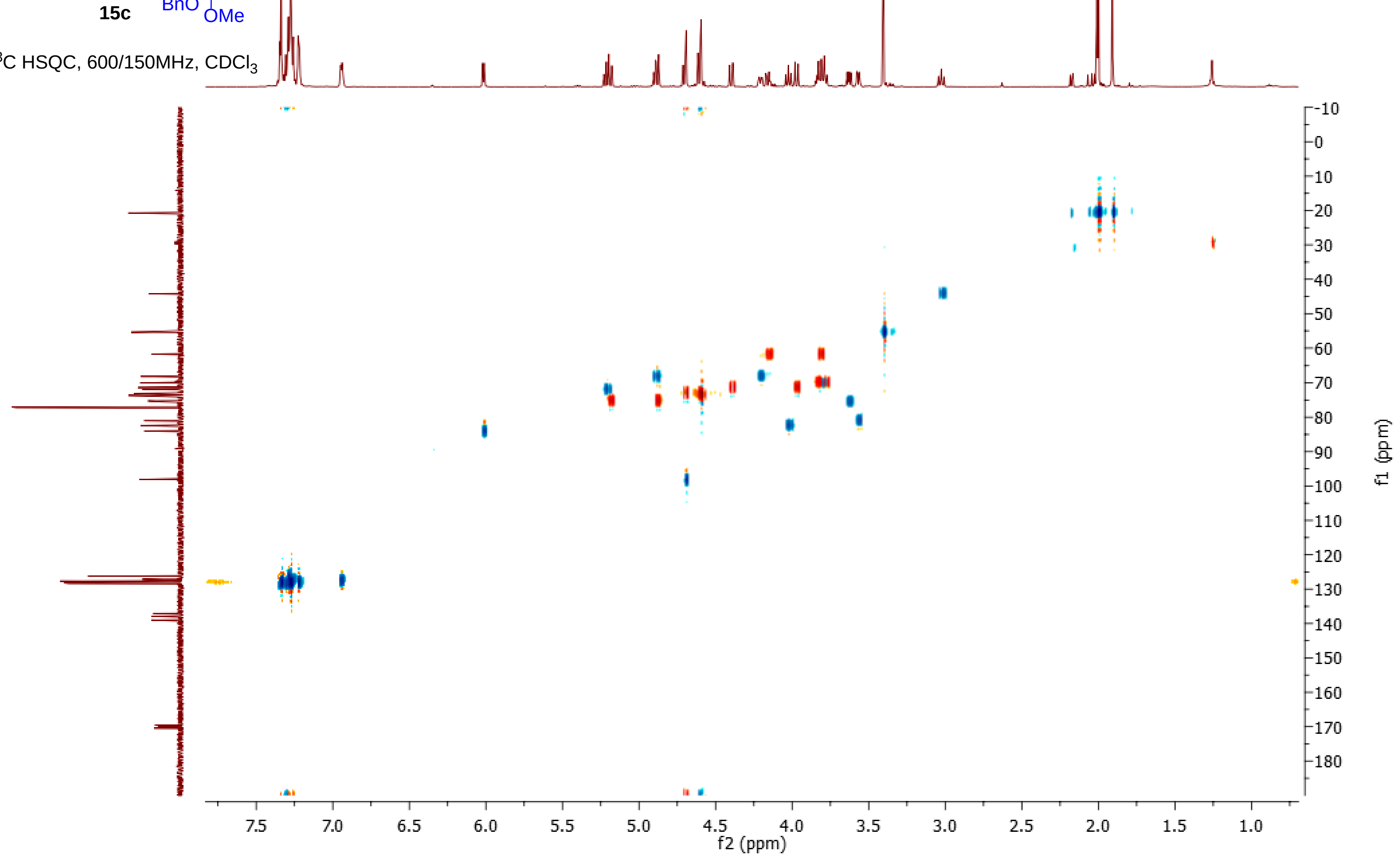

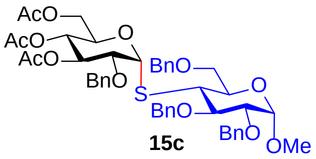

15c

^{1}H-^{13}C Coupled HSQC, 600/150MHz, $CDCl_3$

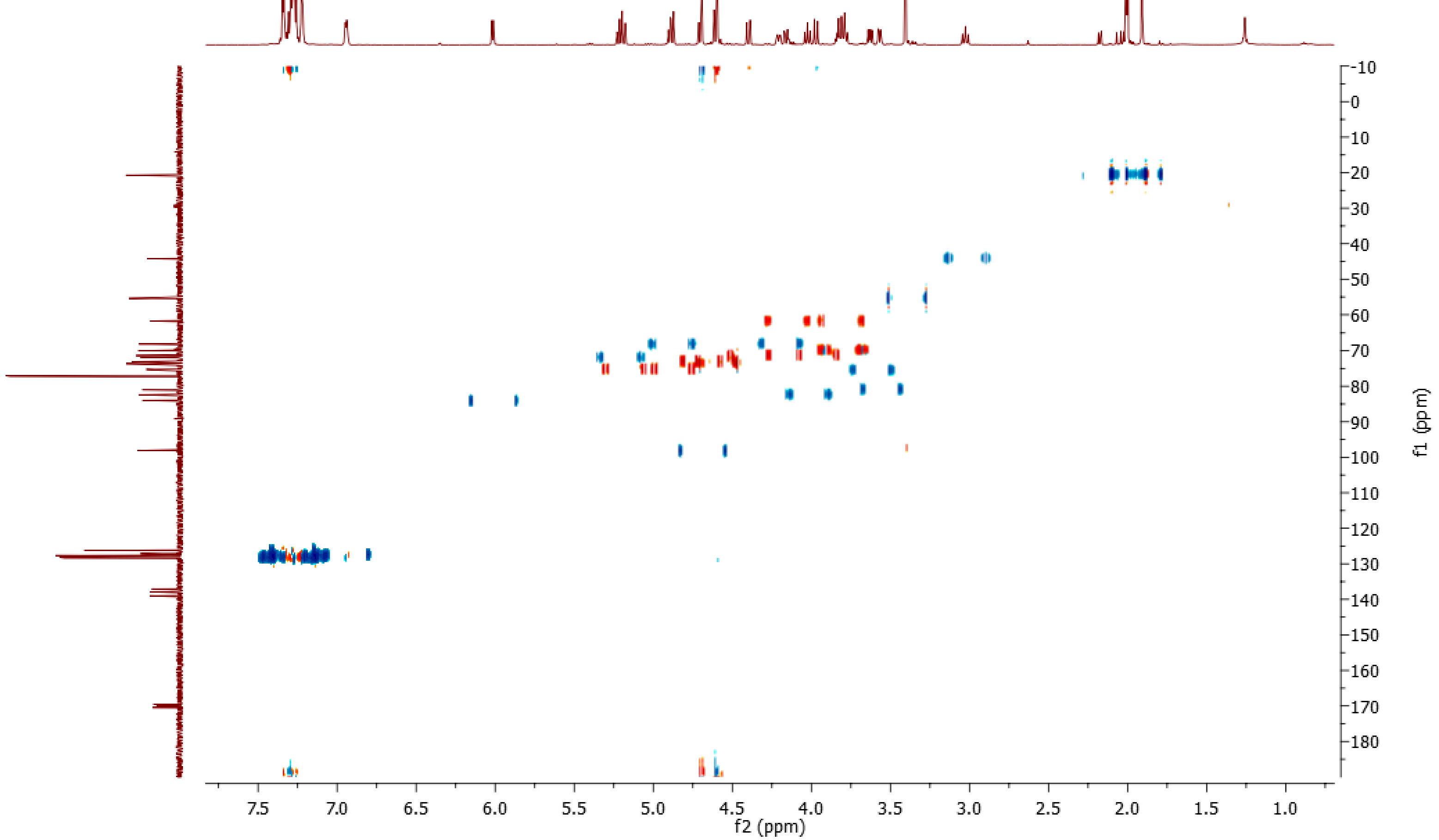

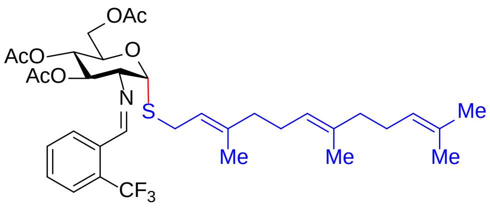

16a

^{1}H NMR

600 MHz

$CDCl_3$

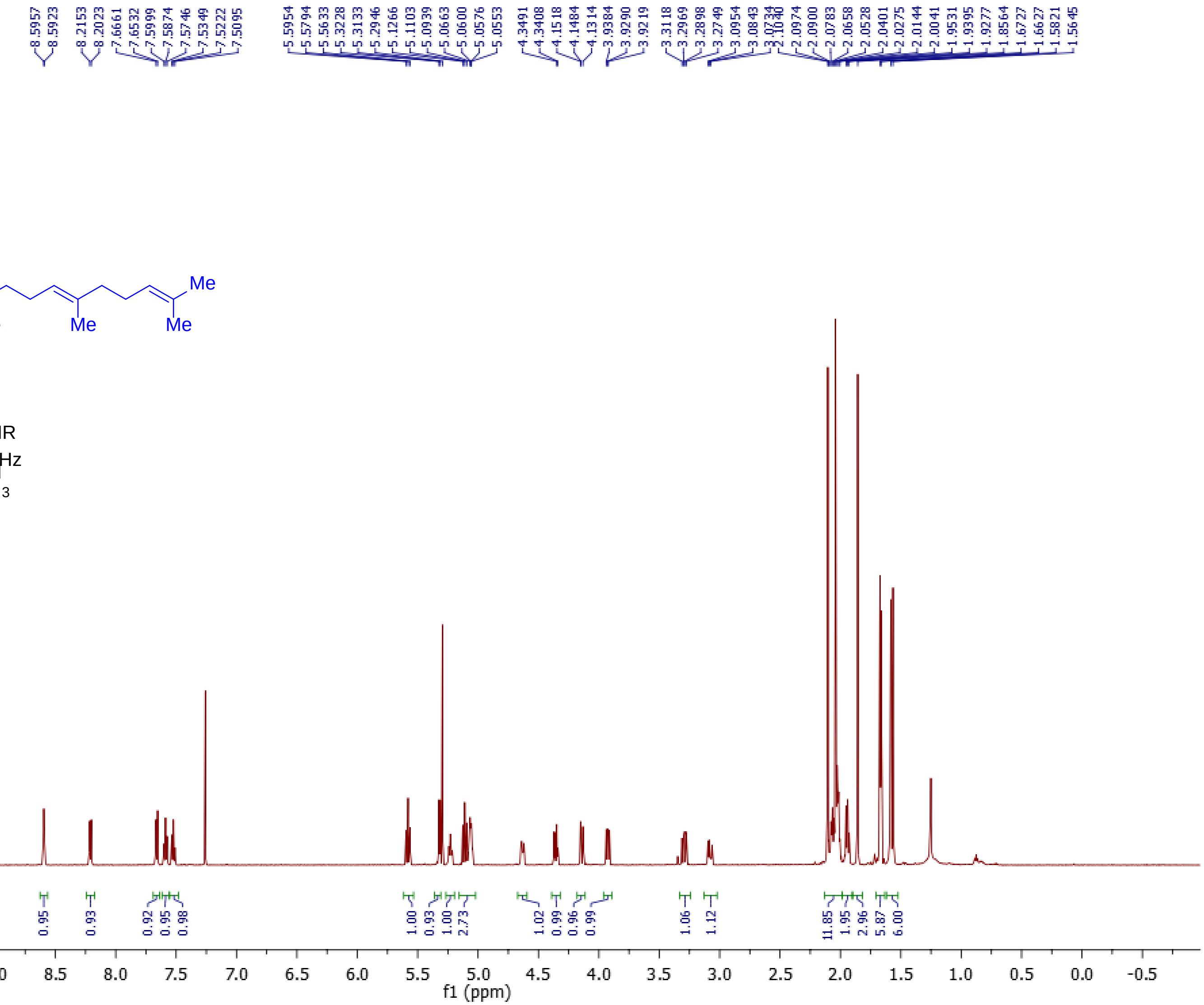

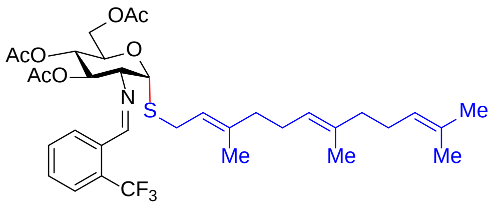

16a

^{13}C NMR, 150 MHz, $CDCl_3$

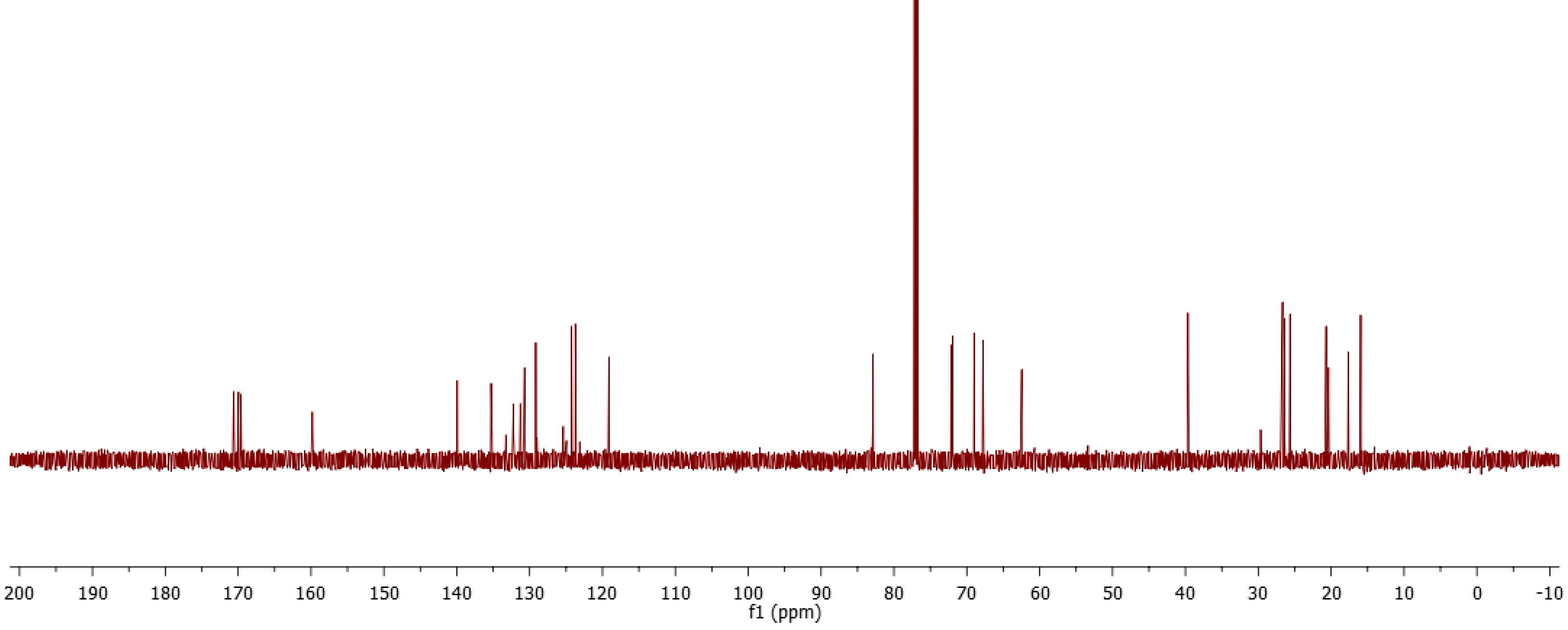

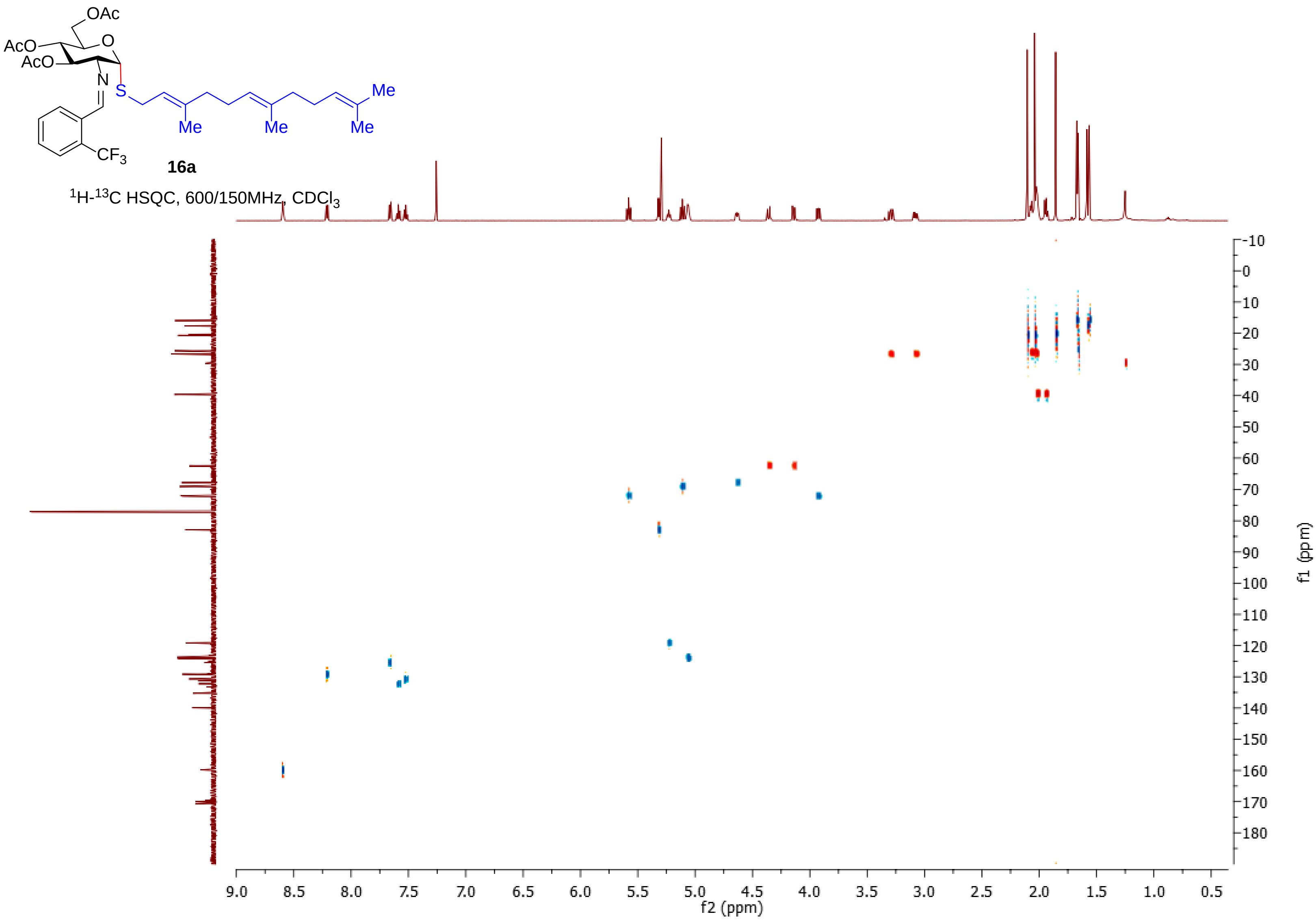
OAc
AcO
AcO
O
N
S
Me
Me
Me
Me
CF3
16a
1H-13C HSQC, 600/150MHz, CDCl3
f2 (ppm)
f1 (ppm)

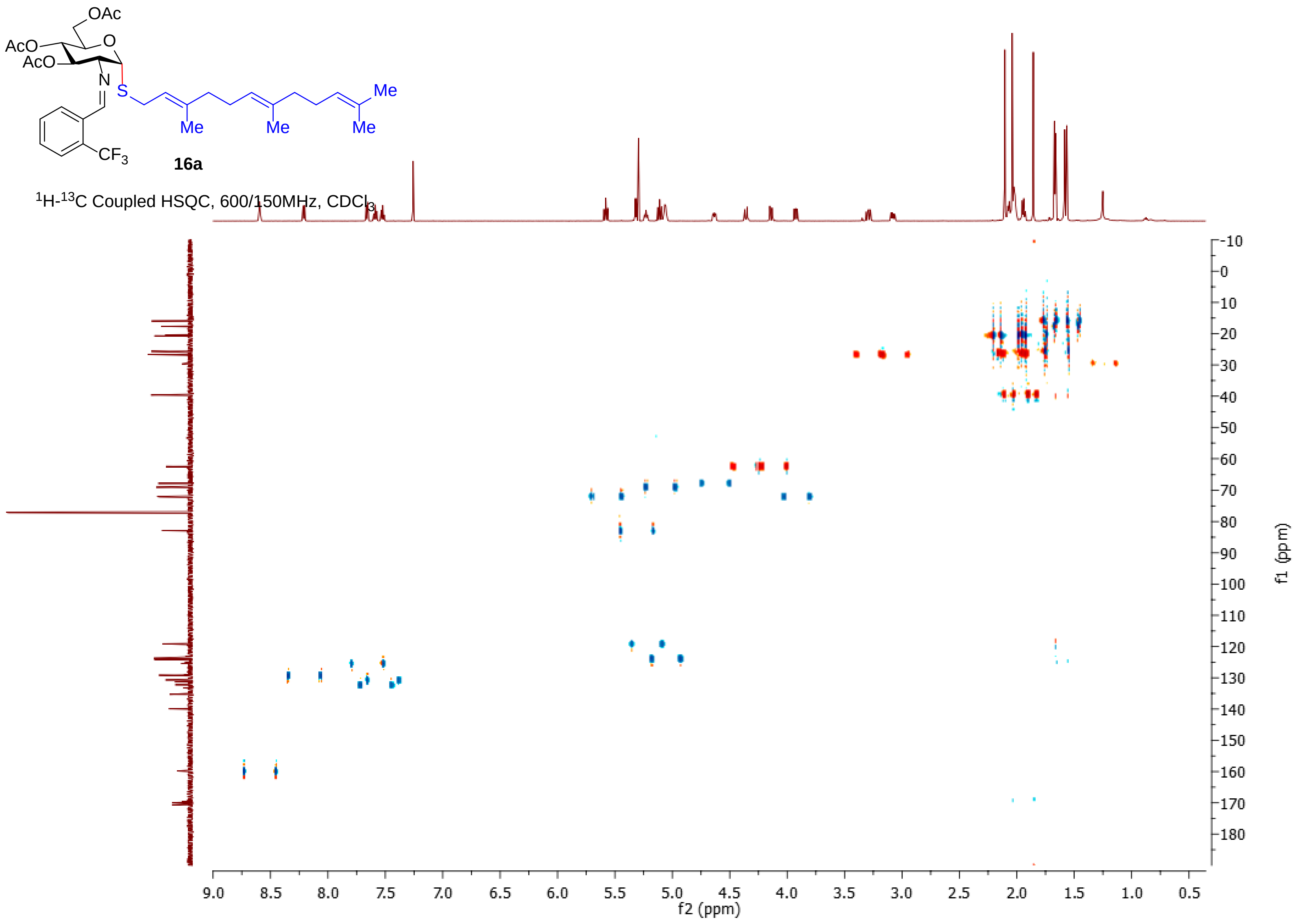

16a

^{1}H-^{13}C Coupled HSQC, 600/150MHz, $CDCl_3$

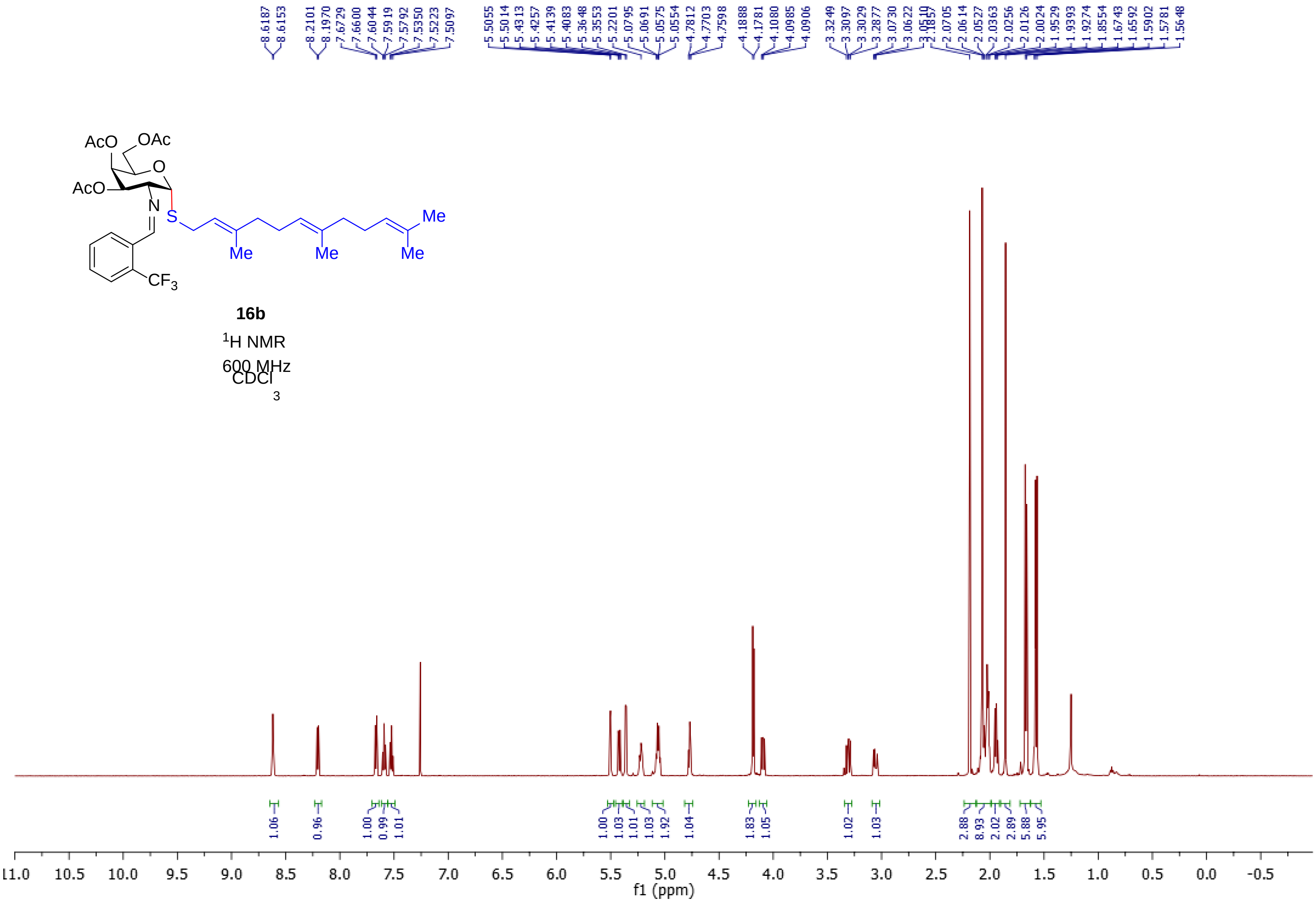
AcO
OAc
O
AcO
N
S
Me
Me
Me
Me
CF3
16b
1H NMR
600 MHz
CDCl3
f1 (ppm)

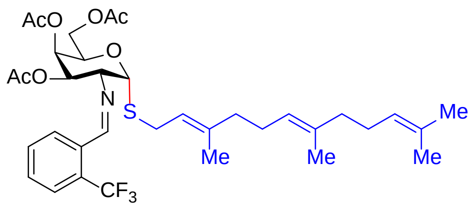

16b

^{13}C NMR, 150 MHz, $CDCl_3$

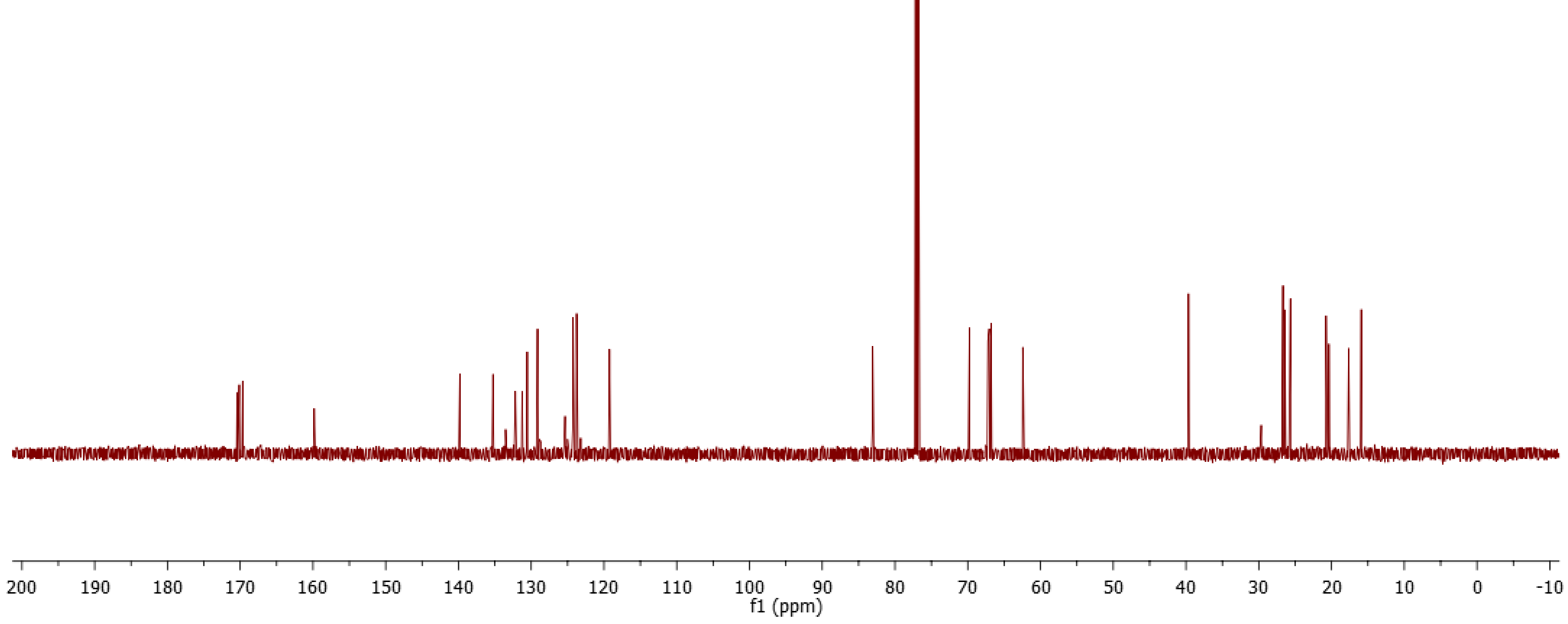

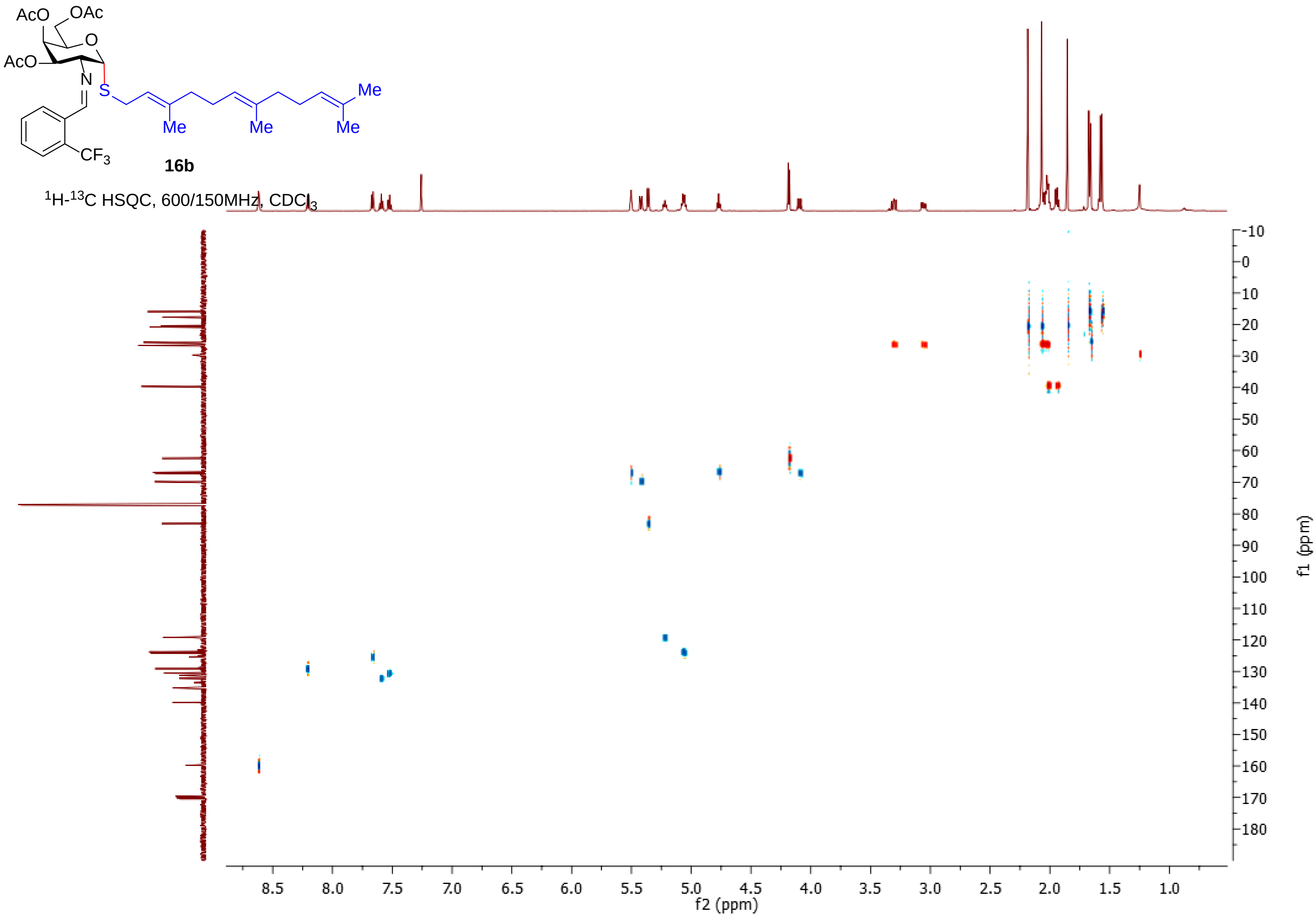
AcO
OAc
O
AcO
N
S
Me
Me
Me
Me
CF3
16b
1H-13C HSQC, 600/150MHz, CDCl3
f2 (ppm)
f1 (ppm)

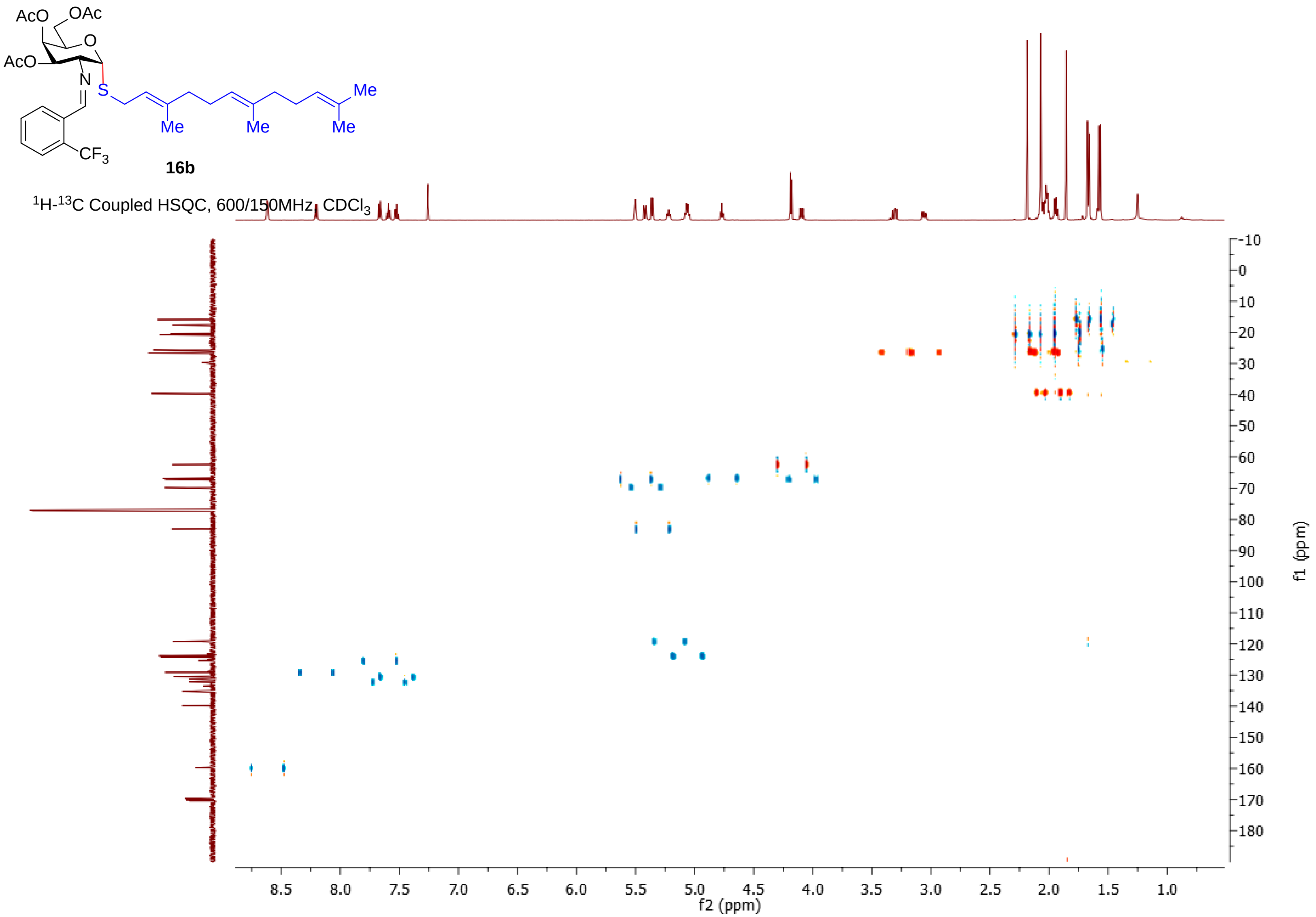

^{1}H-^{13}C Coupled HSQC, 600/150MHz, $CDCl_3$

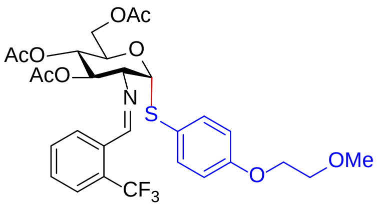

17a

^{1}H NMR
600 MHz
$CDCl_3$

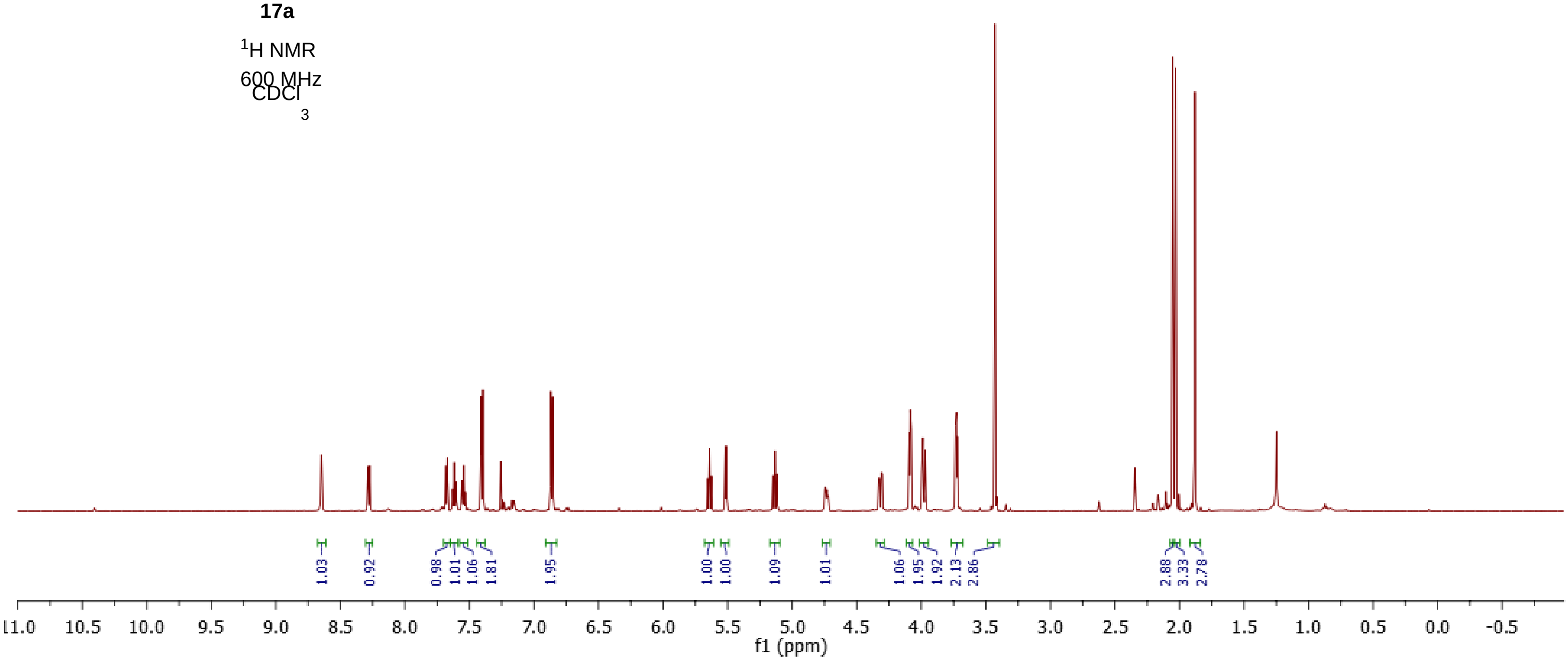

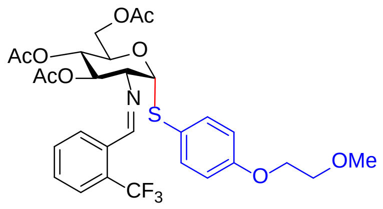

17a

^{13}C NMR, 150 MHz, $CDCl_3$

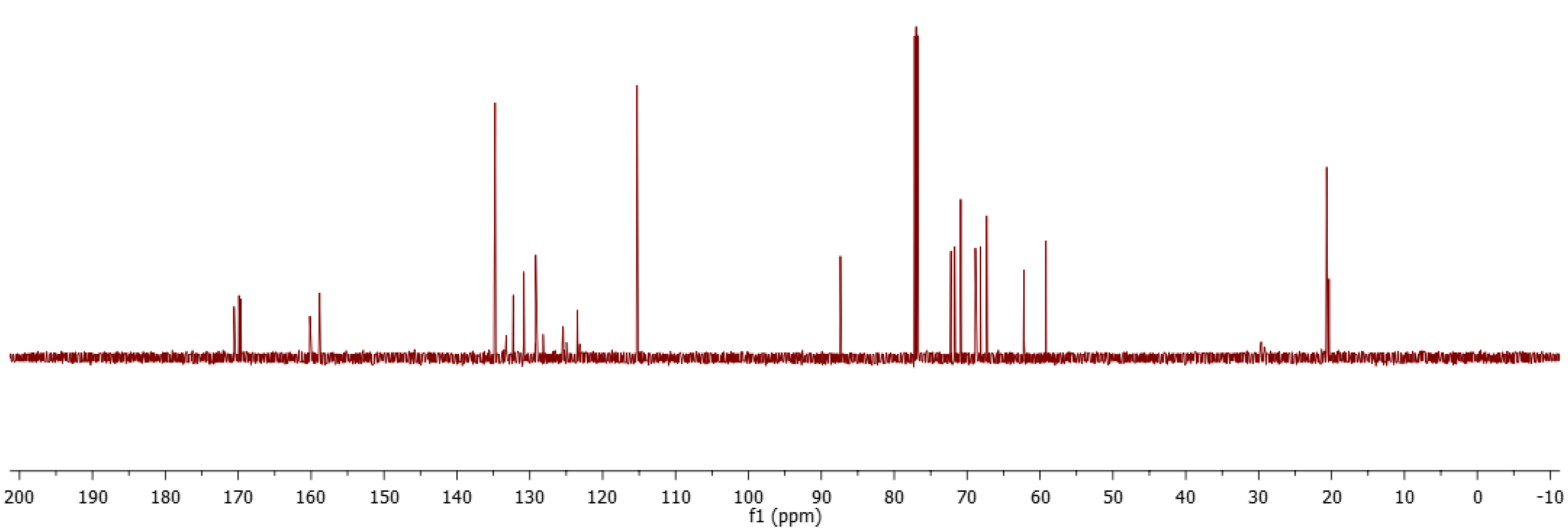

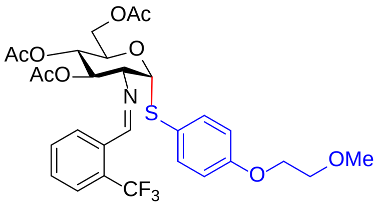

17a

^1H-^{13}C HSQC, 600/150MHz, $CDCl_3$

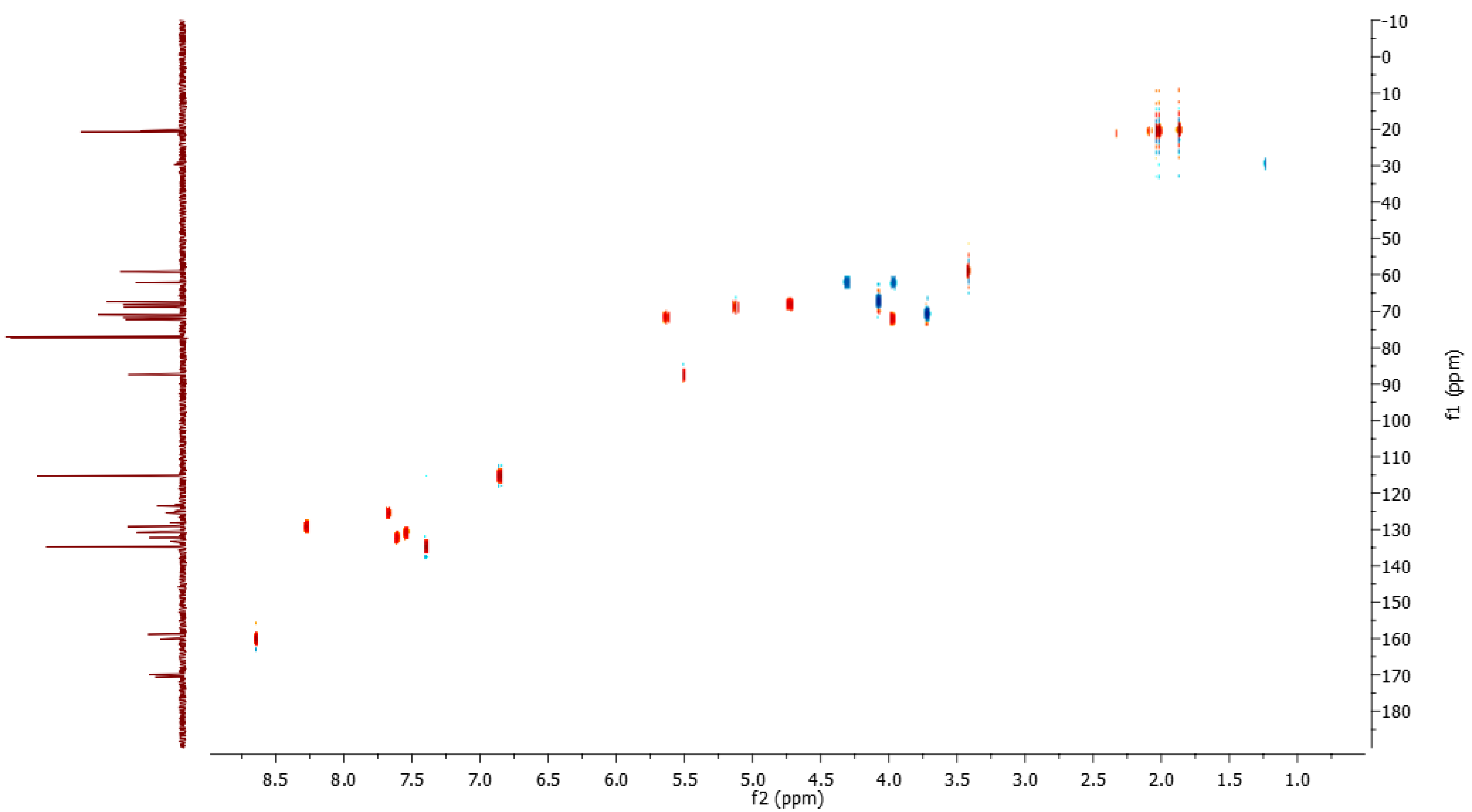

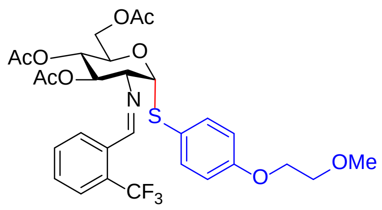

17a

^{1}H-^{13}C Coupled HSQC, 600/150MHz, $CDCl_3$

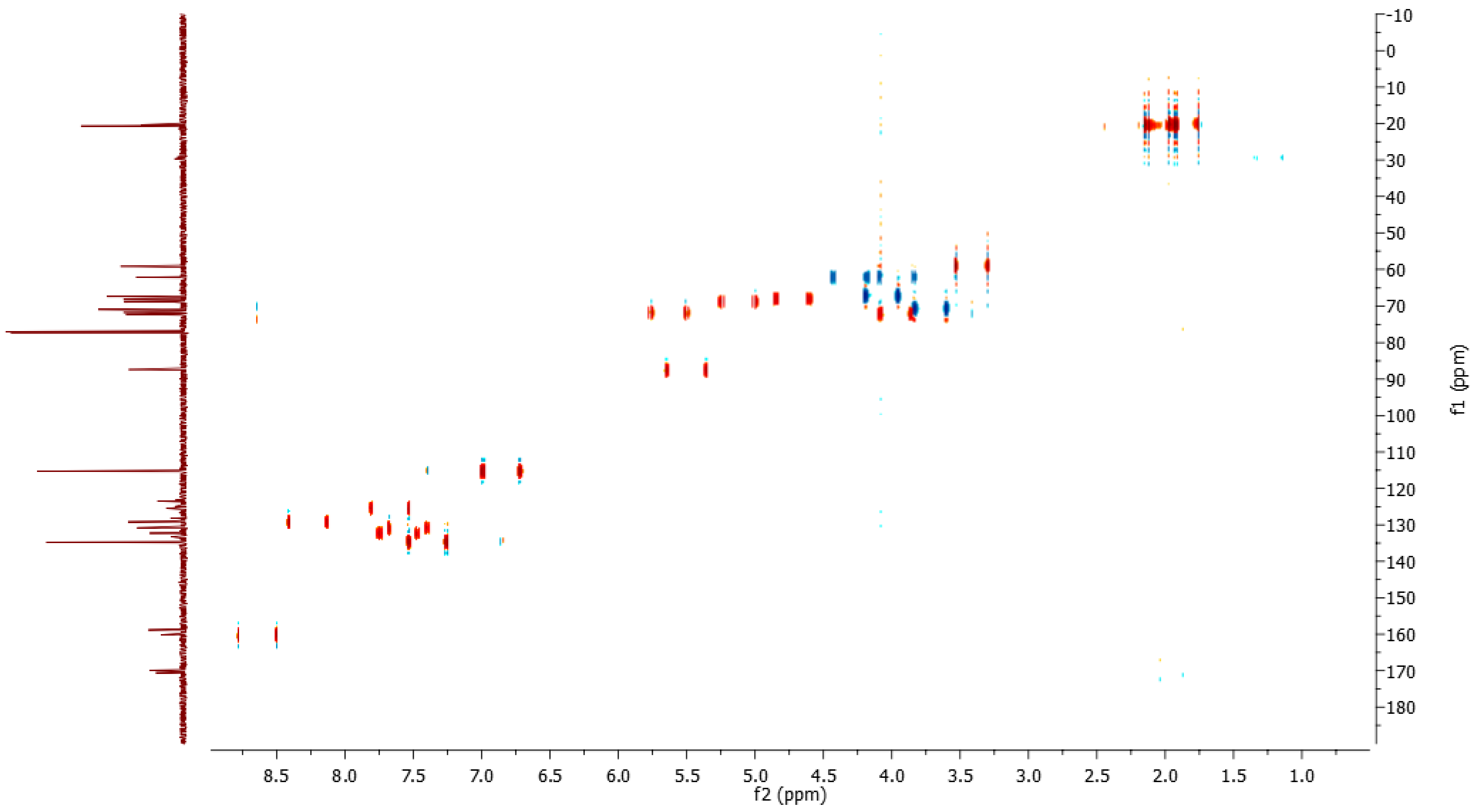

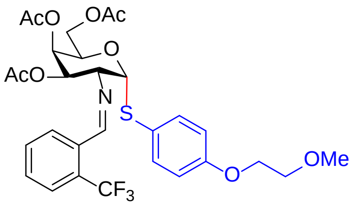

17b

^{1}H NMR

600 MHz

$CDCl_3$

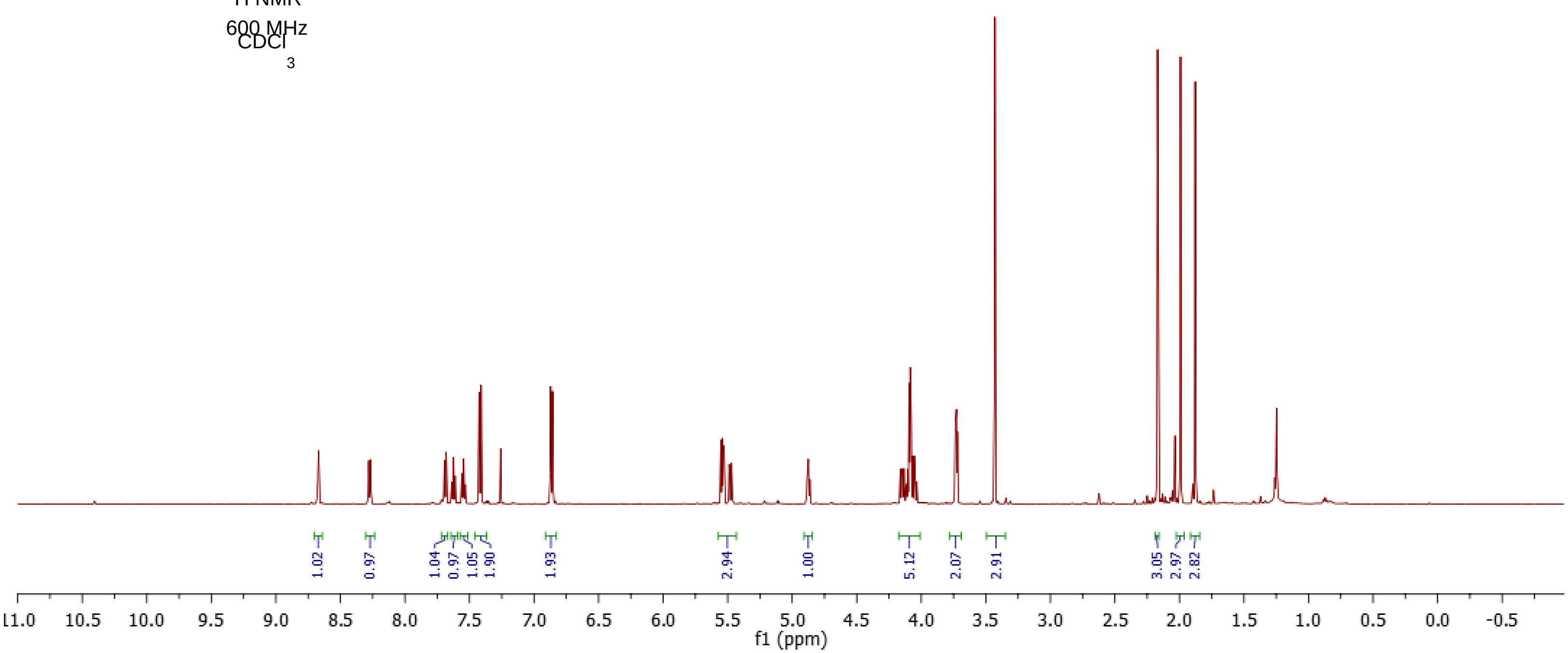

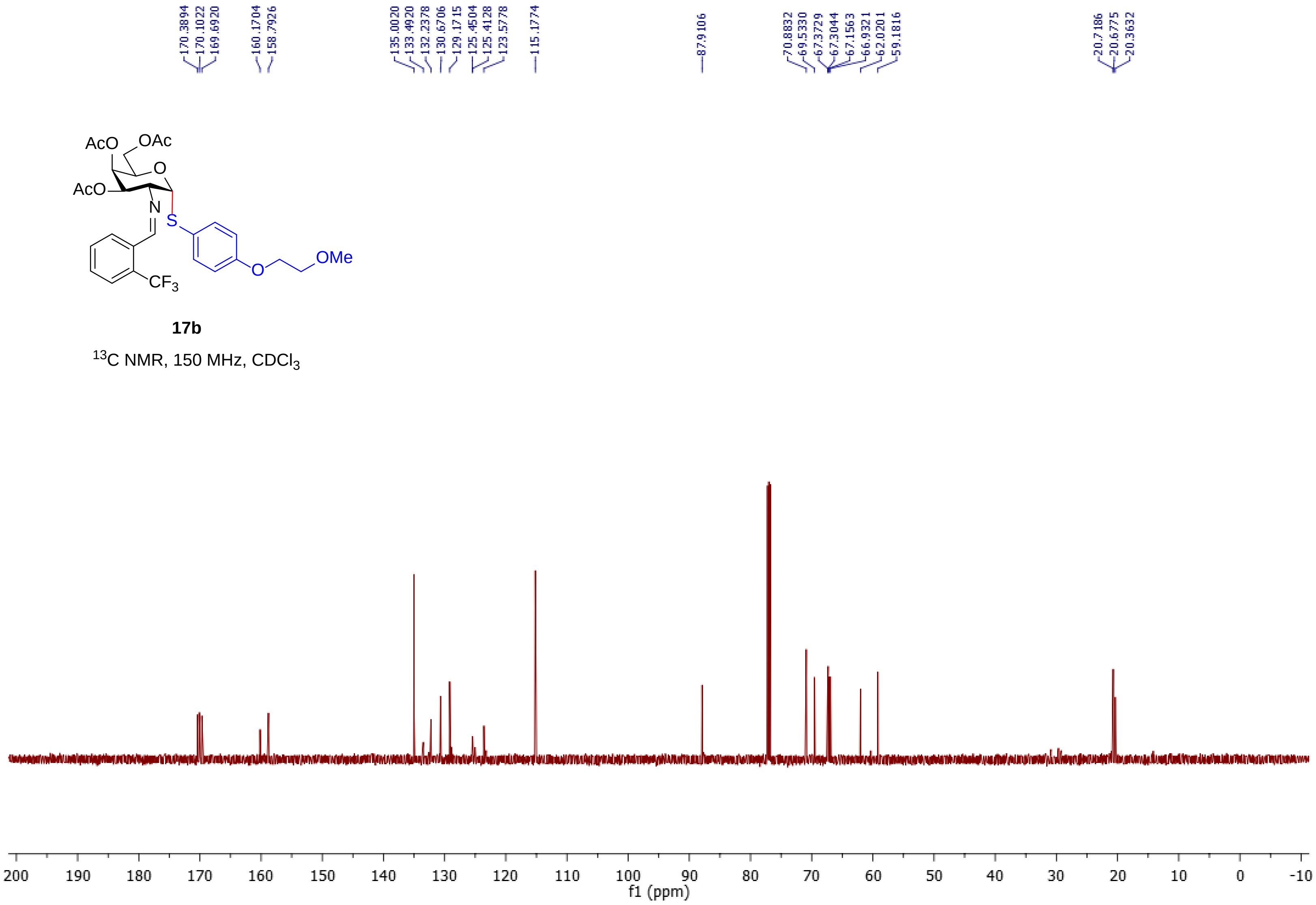

17b

^{13}C NMR, 150 MHz, $CDCl_3$

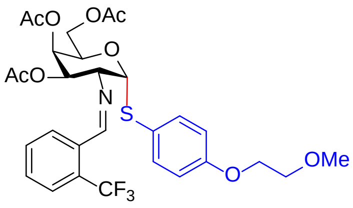

17b

^{1}H-^{13}C HSQC, 600/150MHz, CDCl$_3$

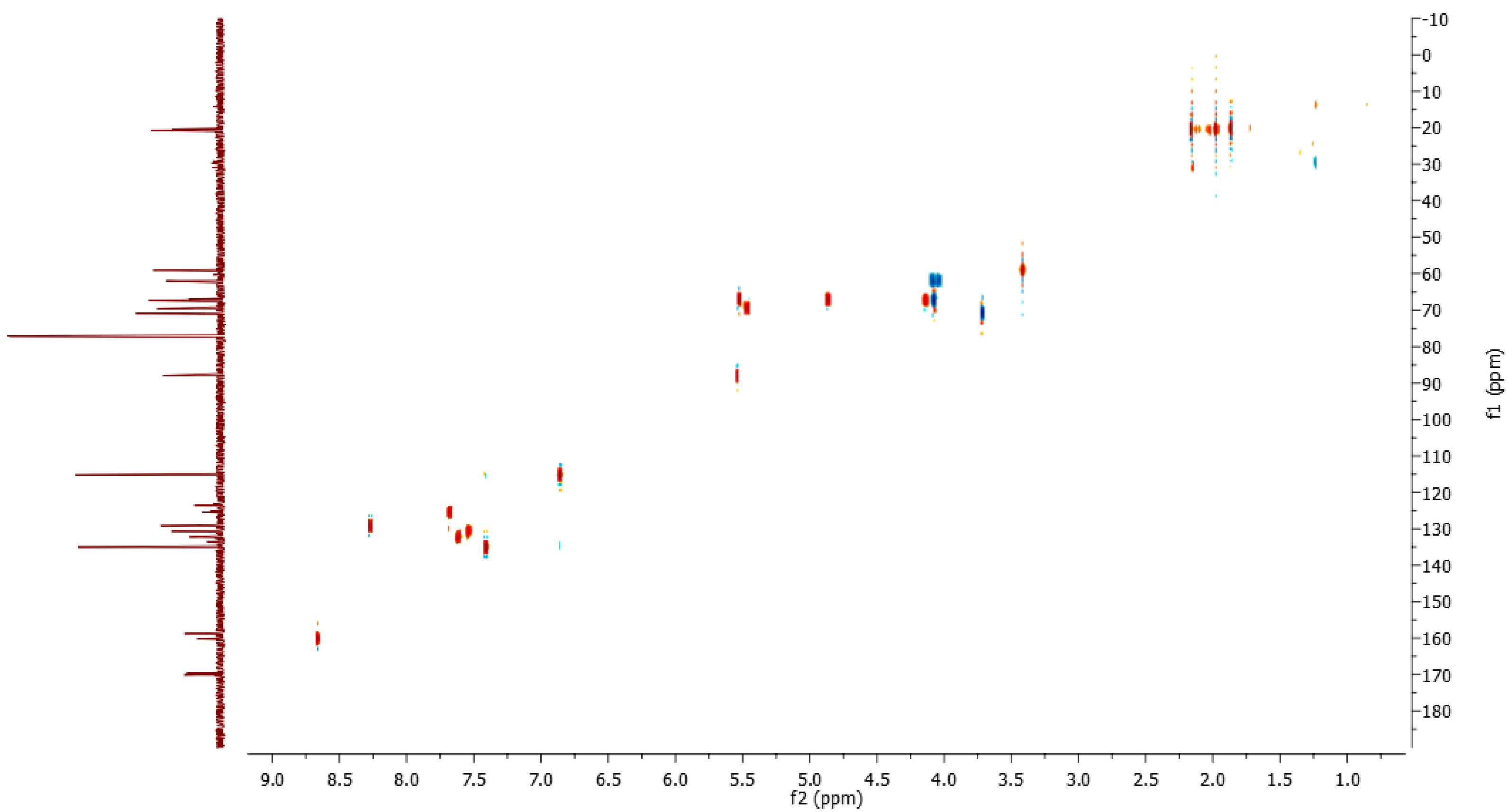

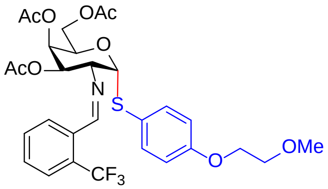

17b

^{1}H-^{13}C Coupled HSQC, 600/150MHz, $CDCl_3$

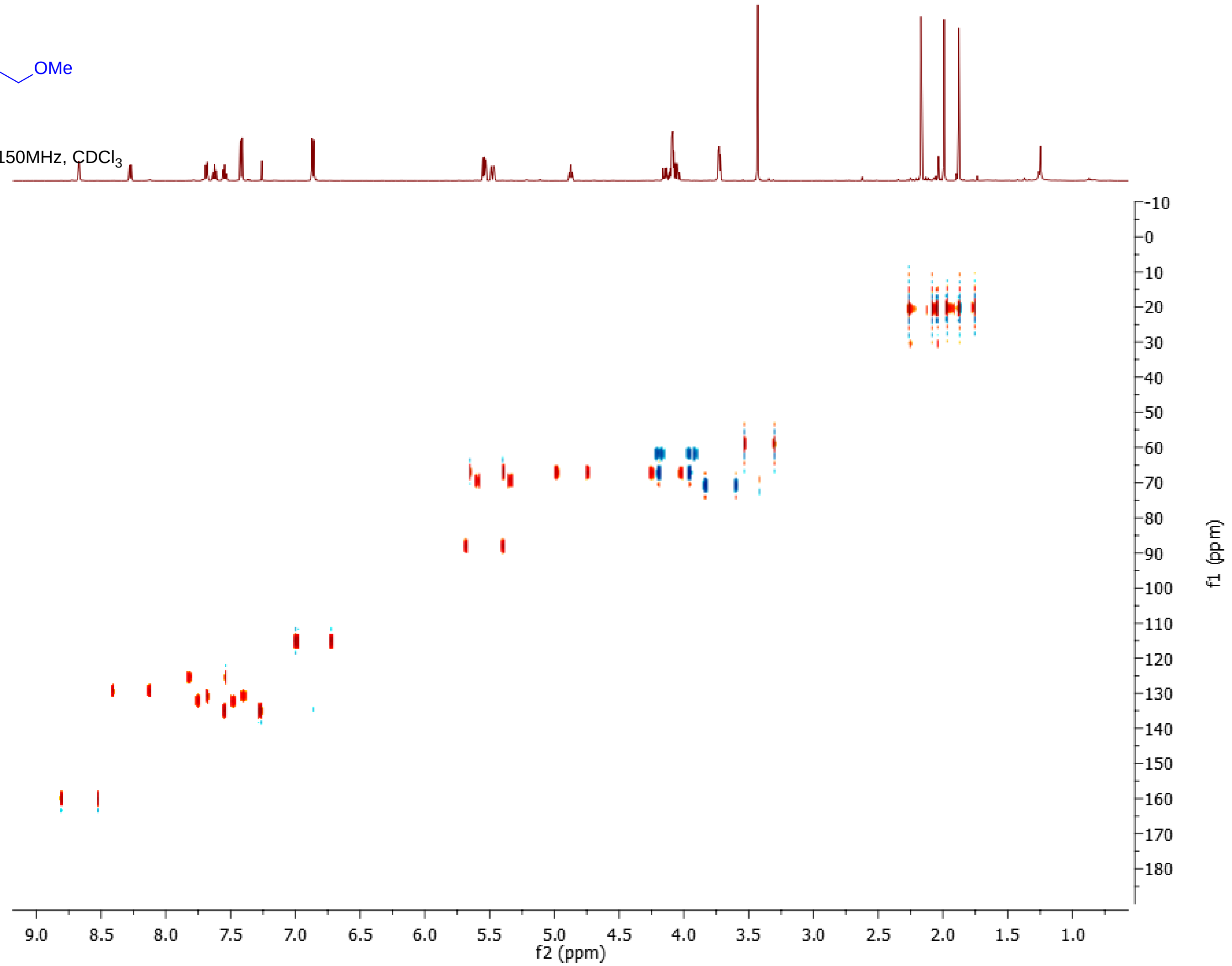

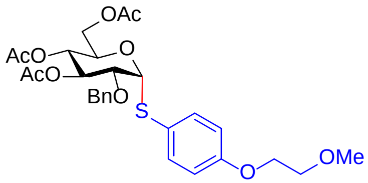

17c

^{1}H NMR
600 MHz
$CDCl_3$

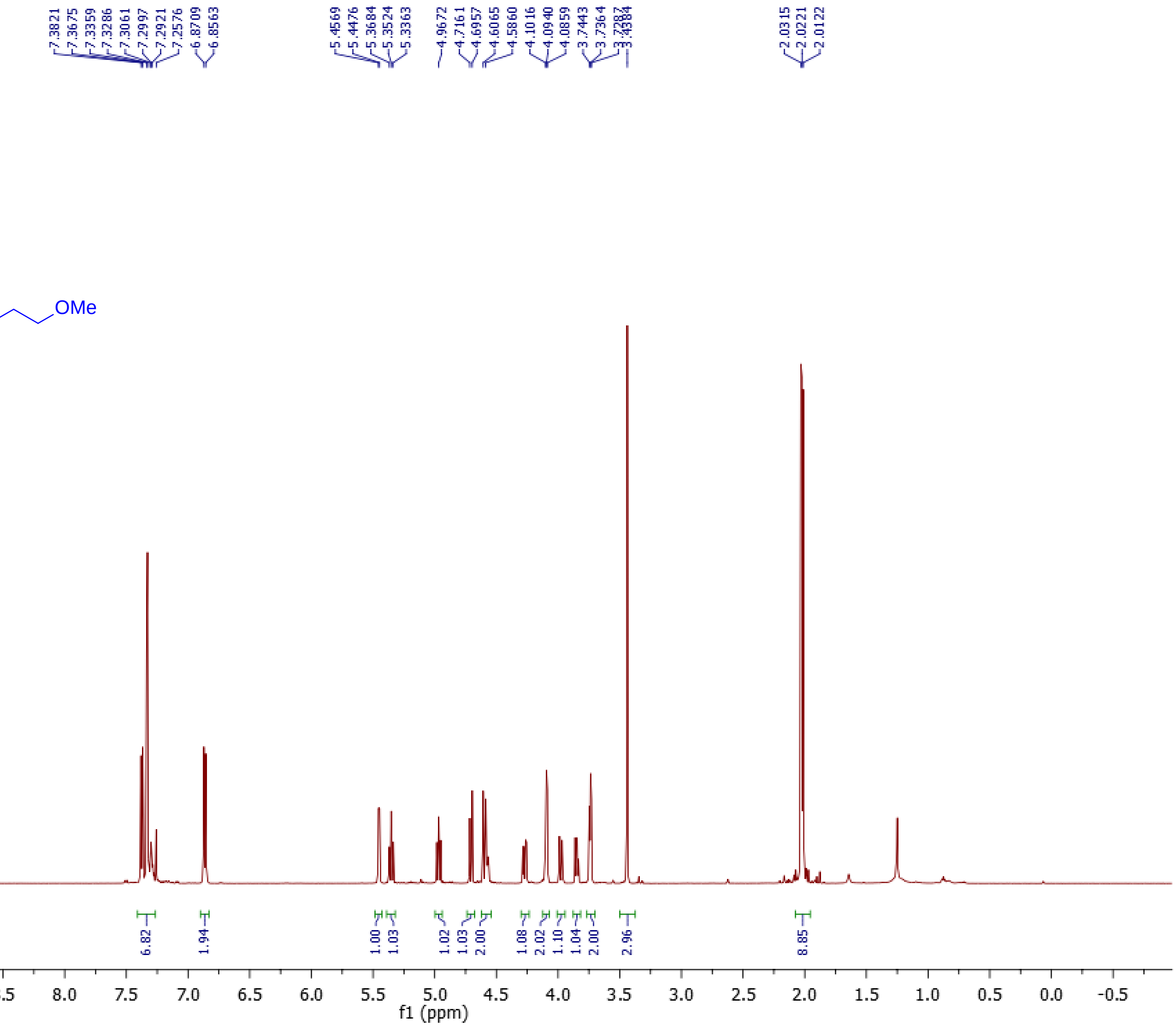

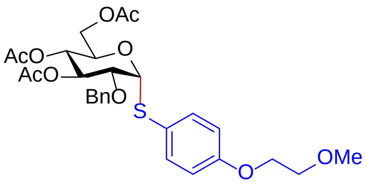

17c

^{13}C NMR, 150 MHz, $CDCl_3$

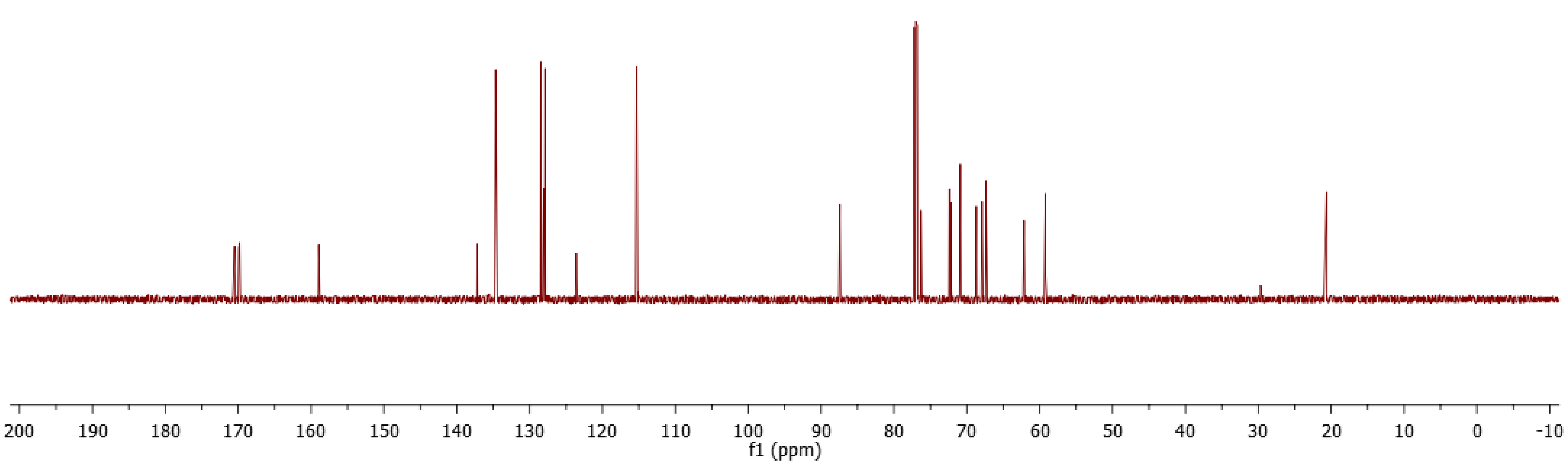

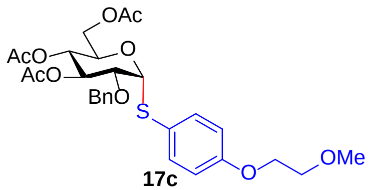

17c

^{1}H-^{13}C HSQC, 600/150MHz, $CDCl_3$

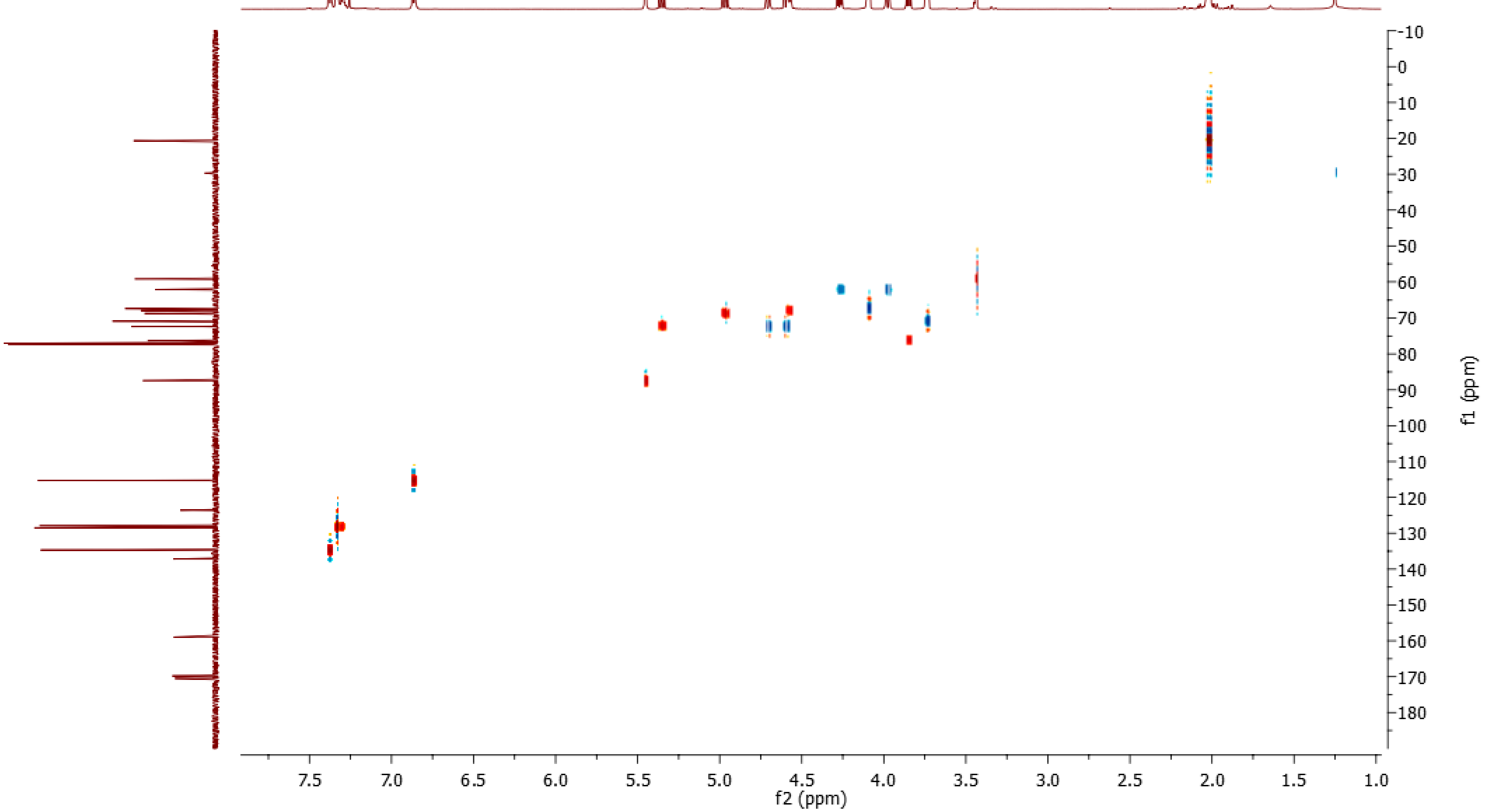

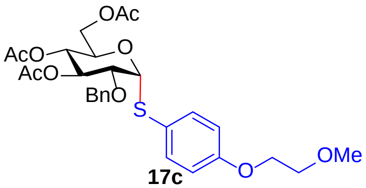

17c

^{1}H-^{13}C Coupled HSQC, 600/150MHz, $CDCl_3$

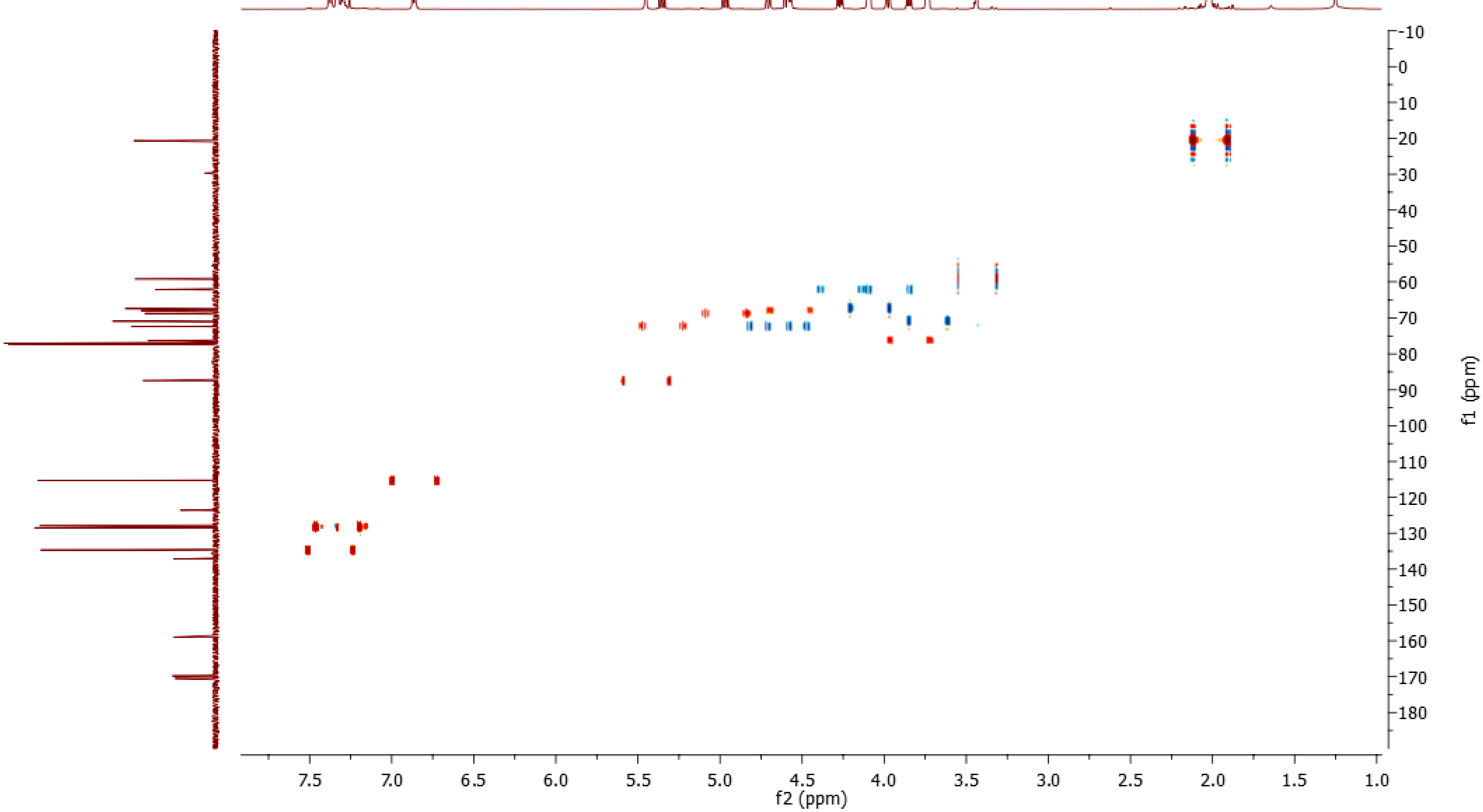

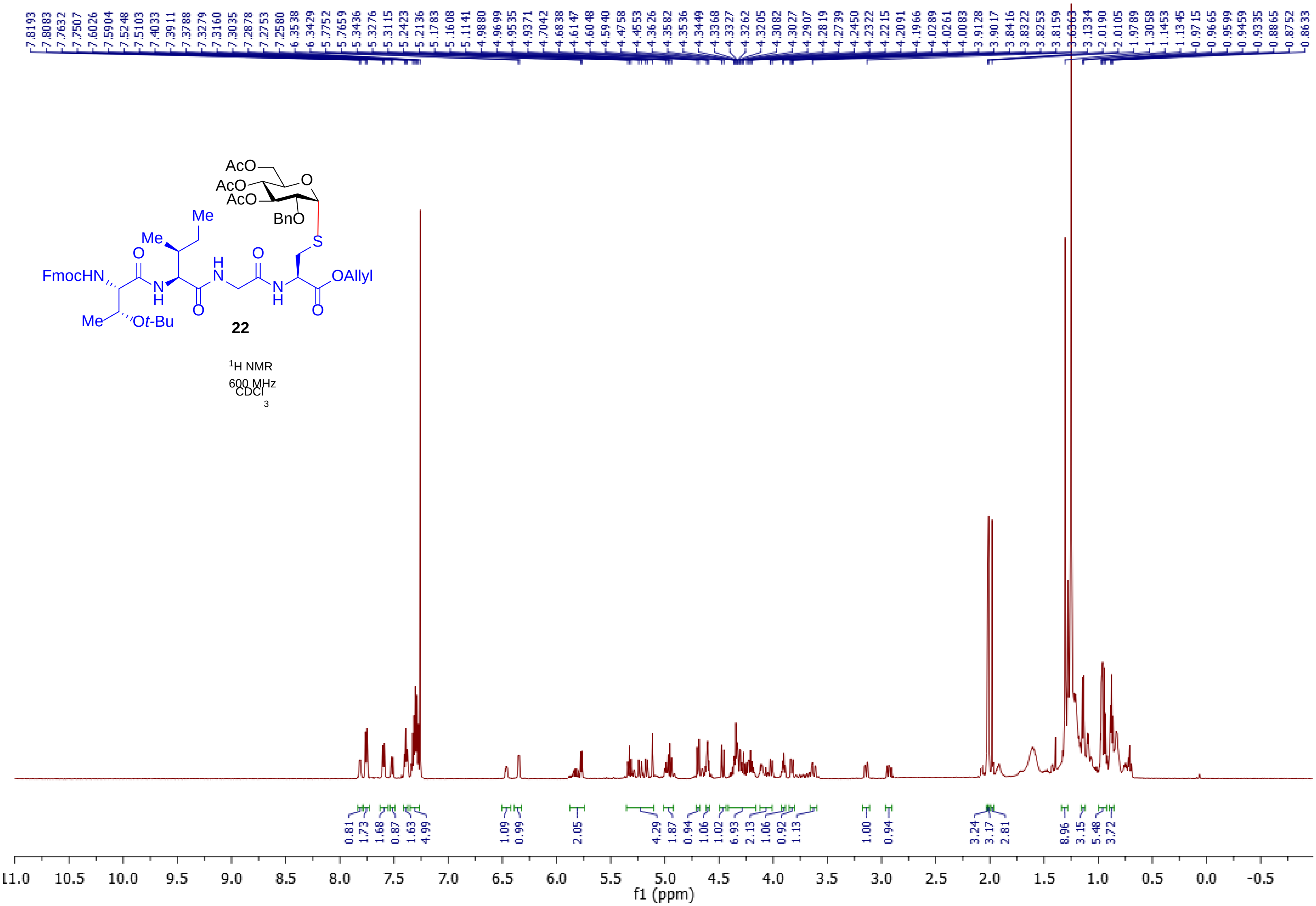

AcO
AcO
AcO
O
BnO
S
Me
Me
O
FmocHN
N
H
H
N
O
N
H
OAllyl
O
O
Me
Ot-Bu
22
^{1}H NMR
600 MHz
$CDCl_3$
f1 (ppm)

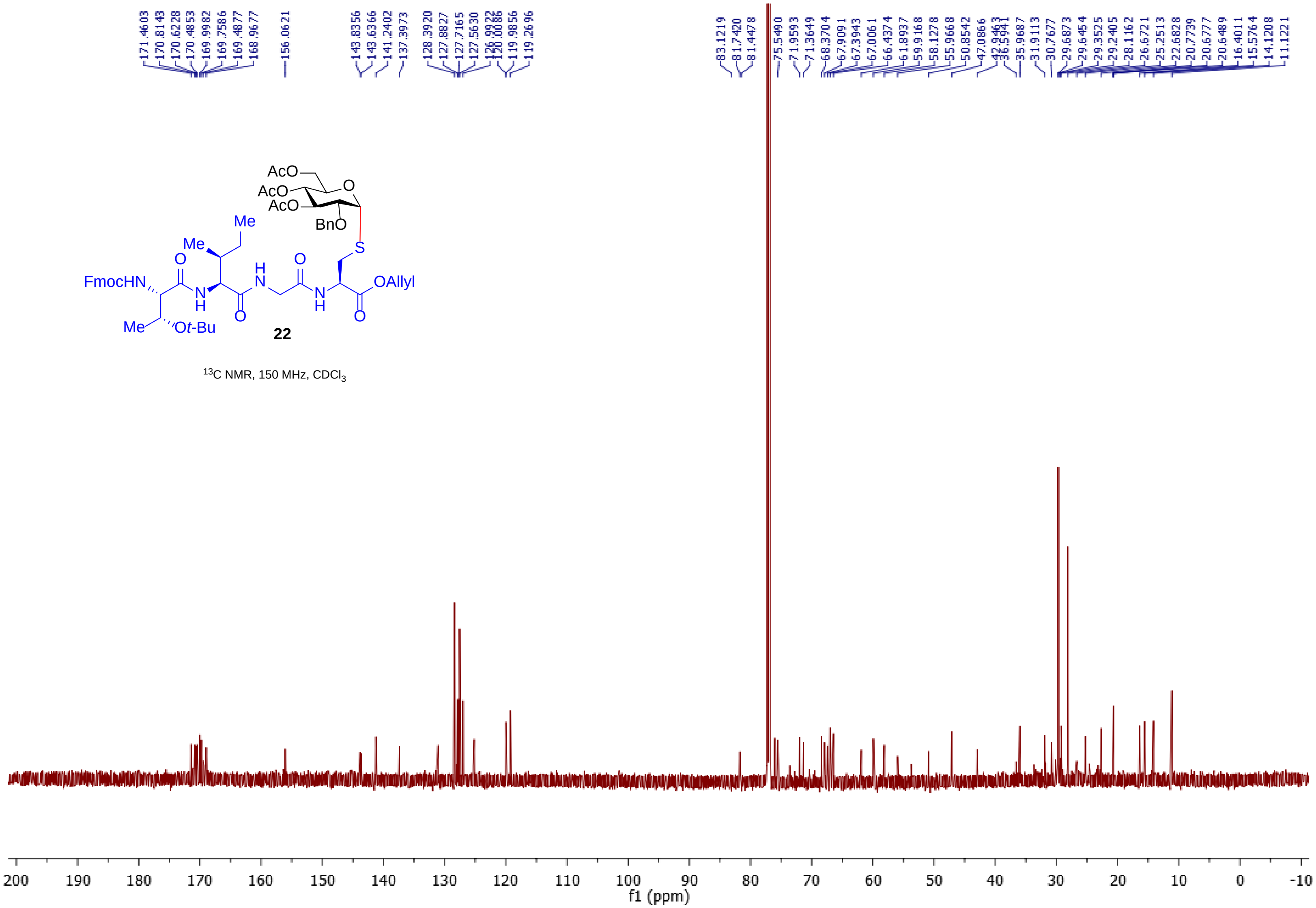

^{13}C NMR, 150 MHz, $CDCl_3$

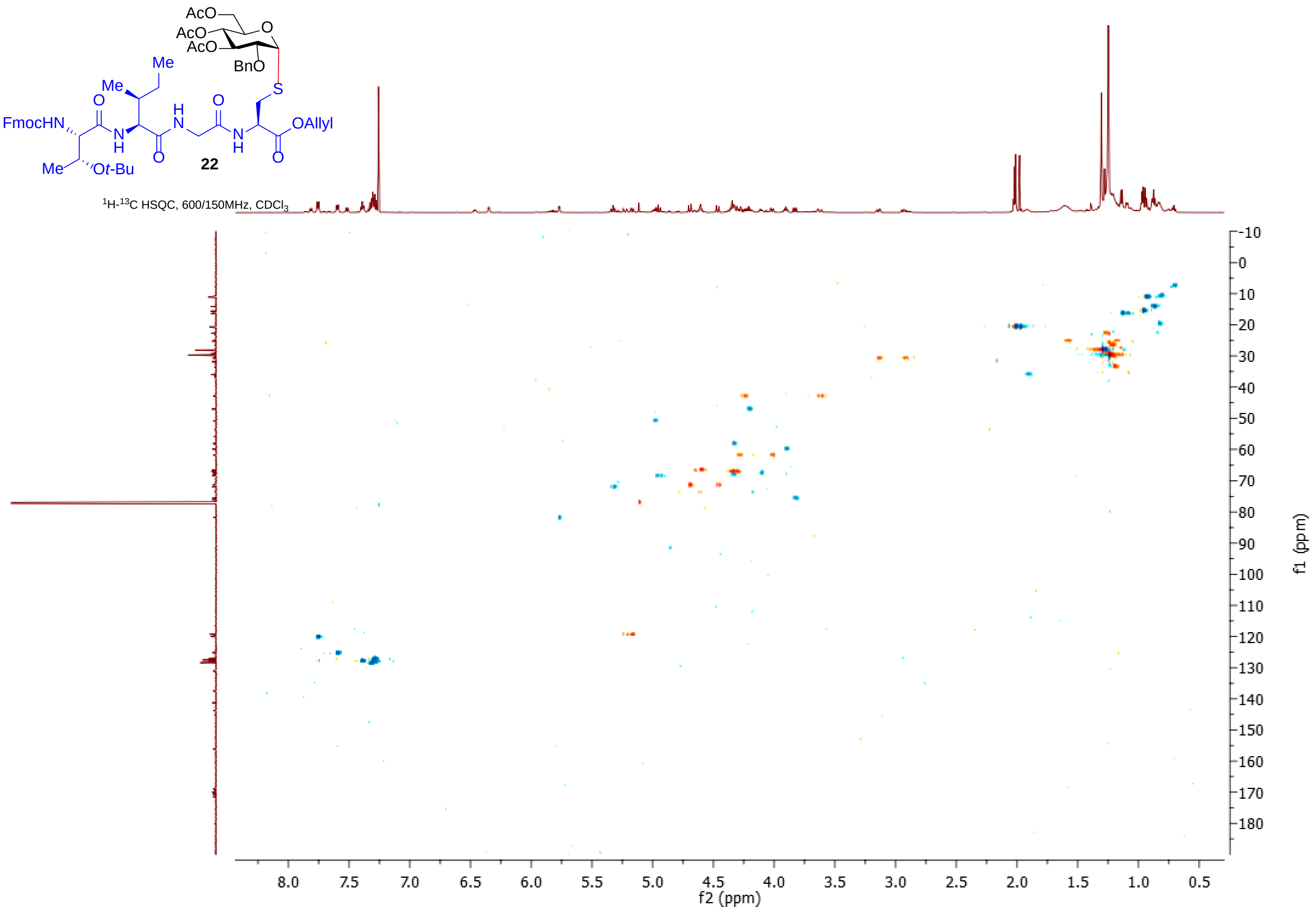
AcO
AcO
AcO
O
BnO
S
Me
Me
O
H
N
O
FmocHN
N
H
N
H
OAllyl
O
O
Me
Ot-Bu
22
^{1}H-^{13}C HSQC, 600/150MHz, $CDCl_3$
f2 (ppm)
f1 (ppm)

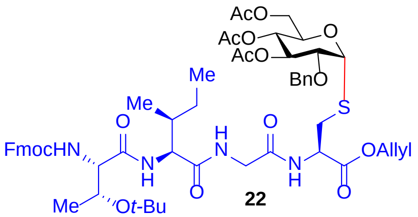

22

^{1}H-^{13}C Coupled HSQC, 600/150MHz, $CDCl_3$

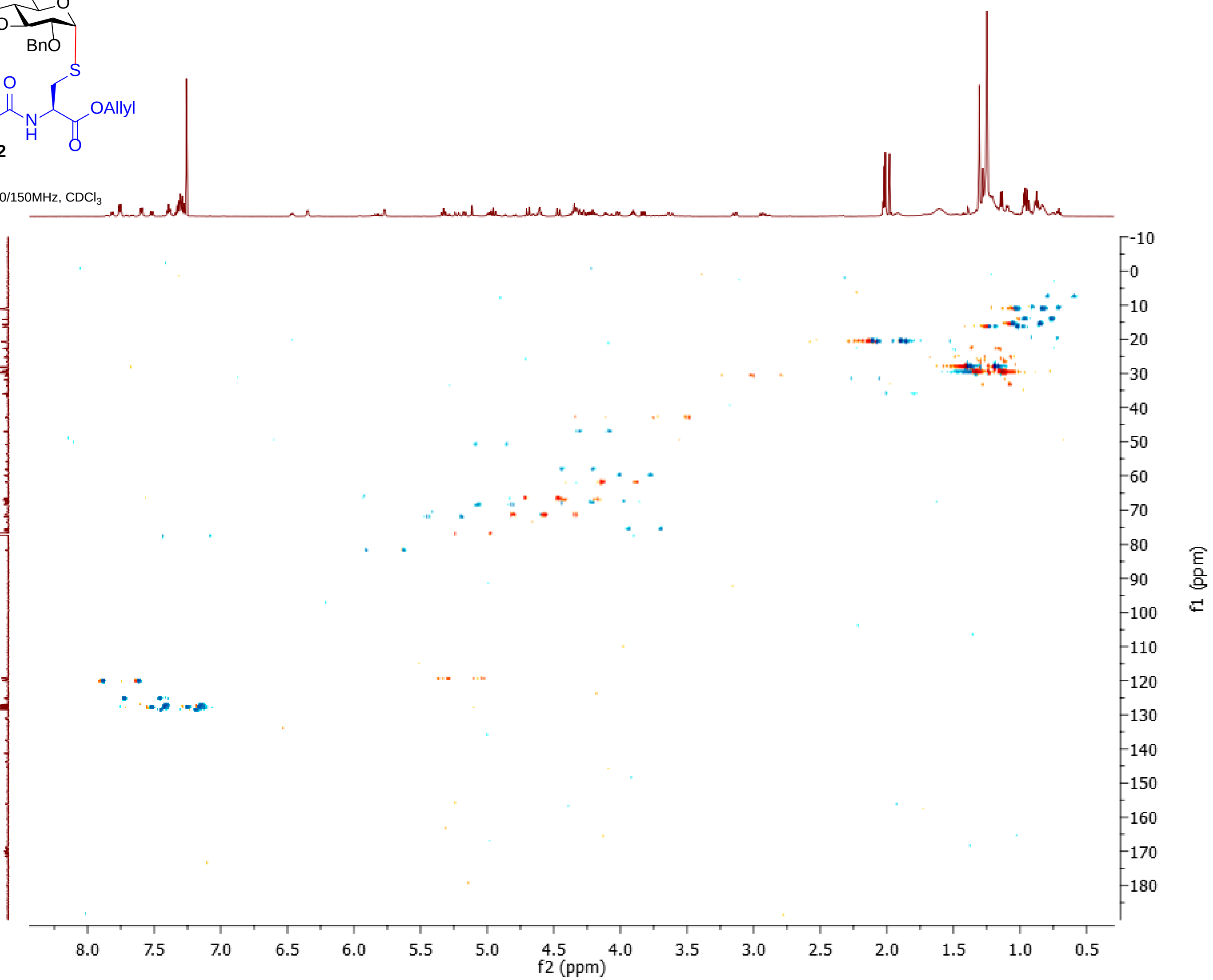

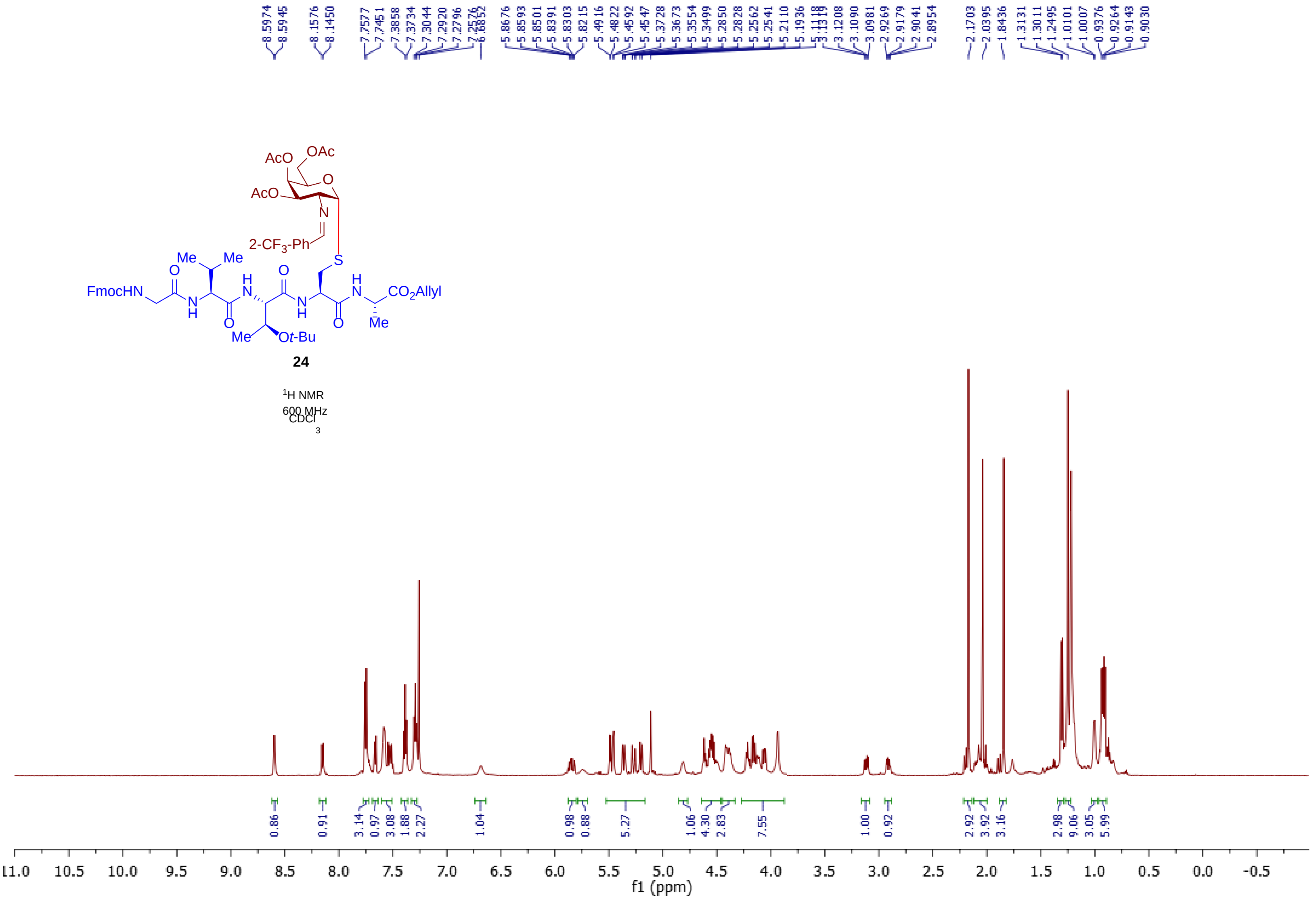
24
1H NMR
600 MHz
CDCl3
f1 (ppm)

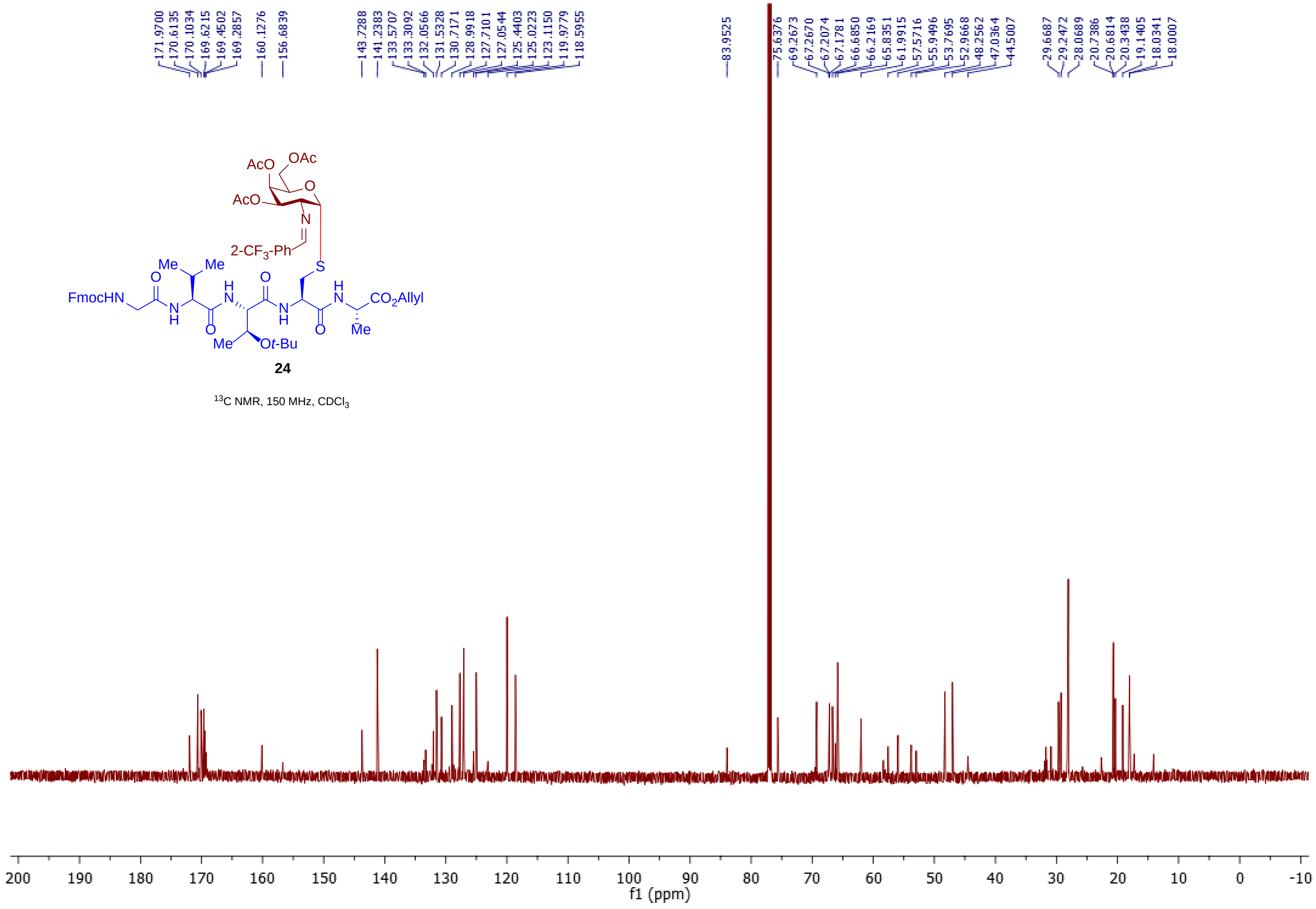

24

^{13}C NMR, 150 MHz, $CDCl_3$

24

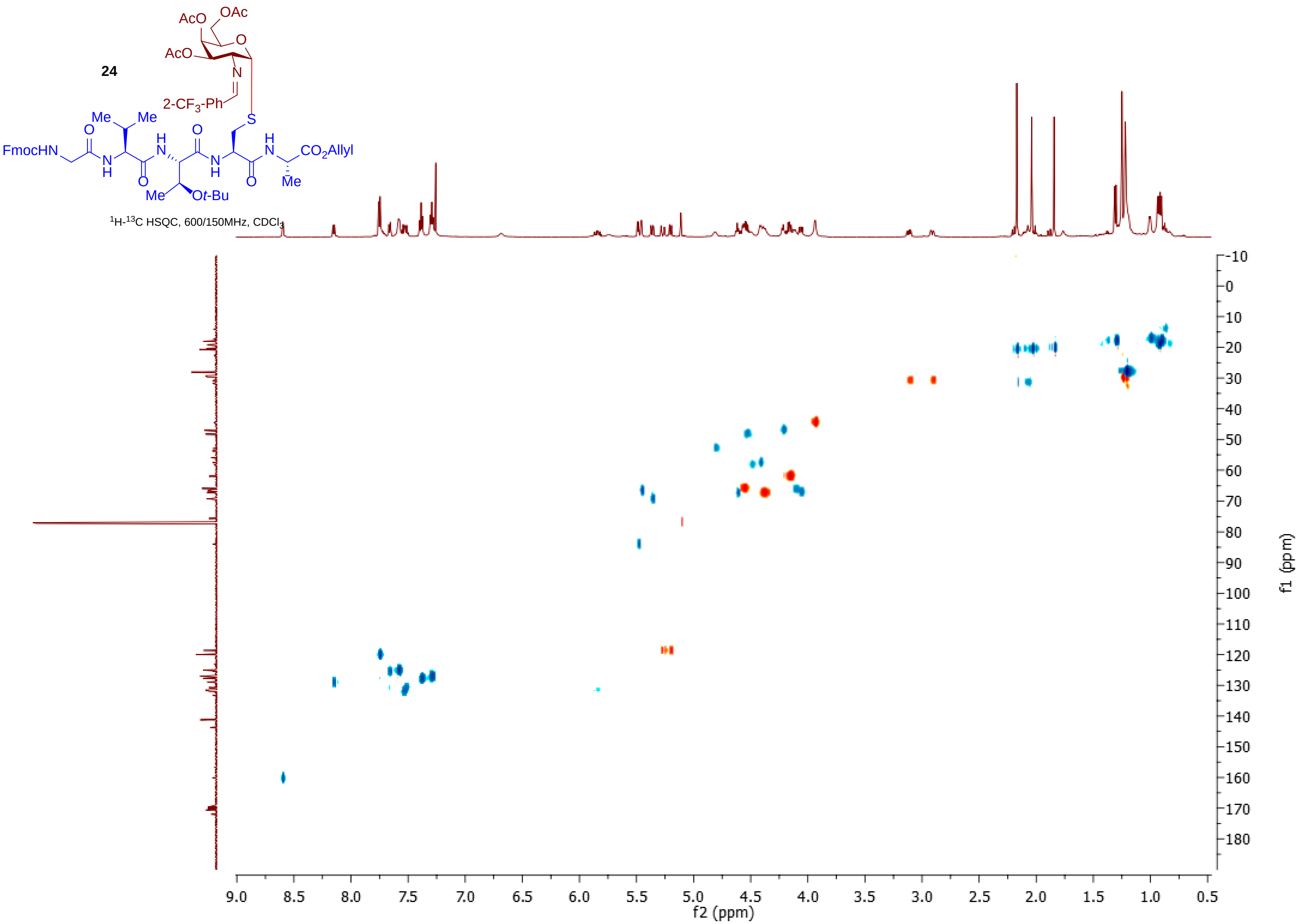

AcO
OAc
O
AcO
N
2-CF3-Ph
S
Me
Me
O
O
H
N
CO2Allyl
FmocHN
N
H
N
H
O
Me
Ot-Bu
N
H
O
Me
1H-13C HSQC, 600/150MHz, CDCl3
f2 (ppm)
f1 (ppm)

24

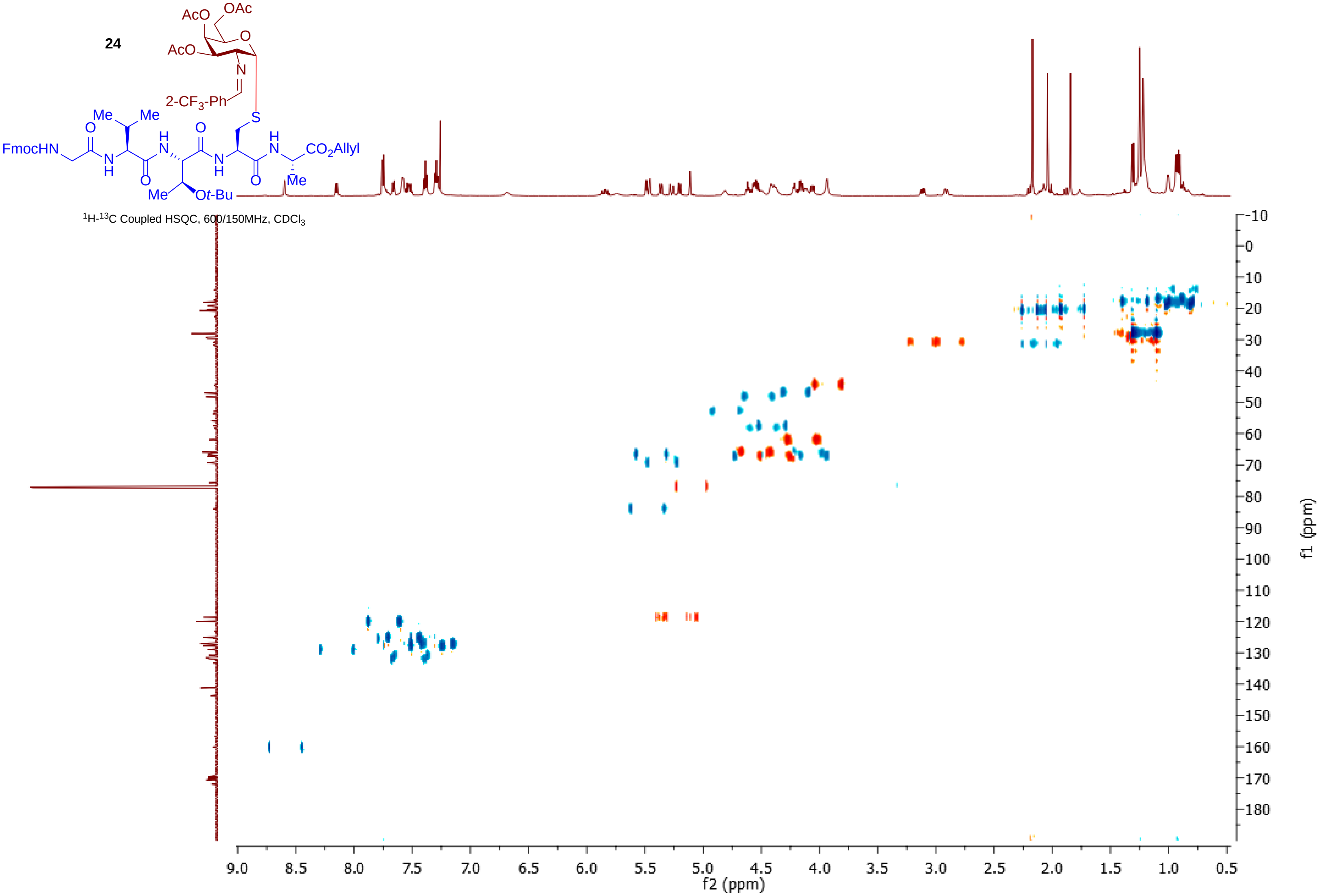

^{1}H-^{13}C Coupled HSQC, 600/150MHz, $CDCl_3$

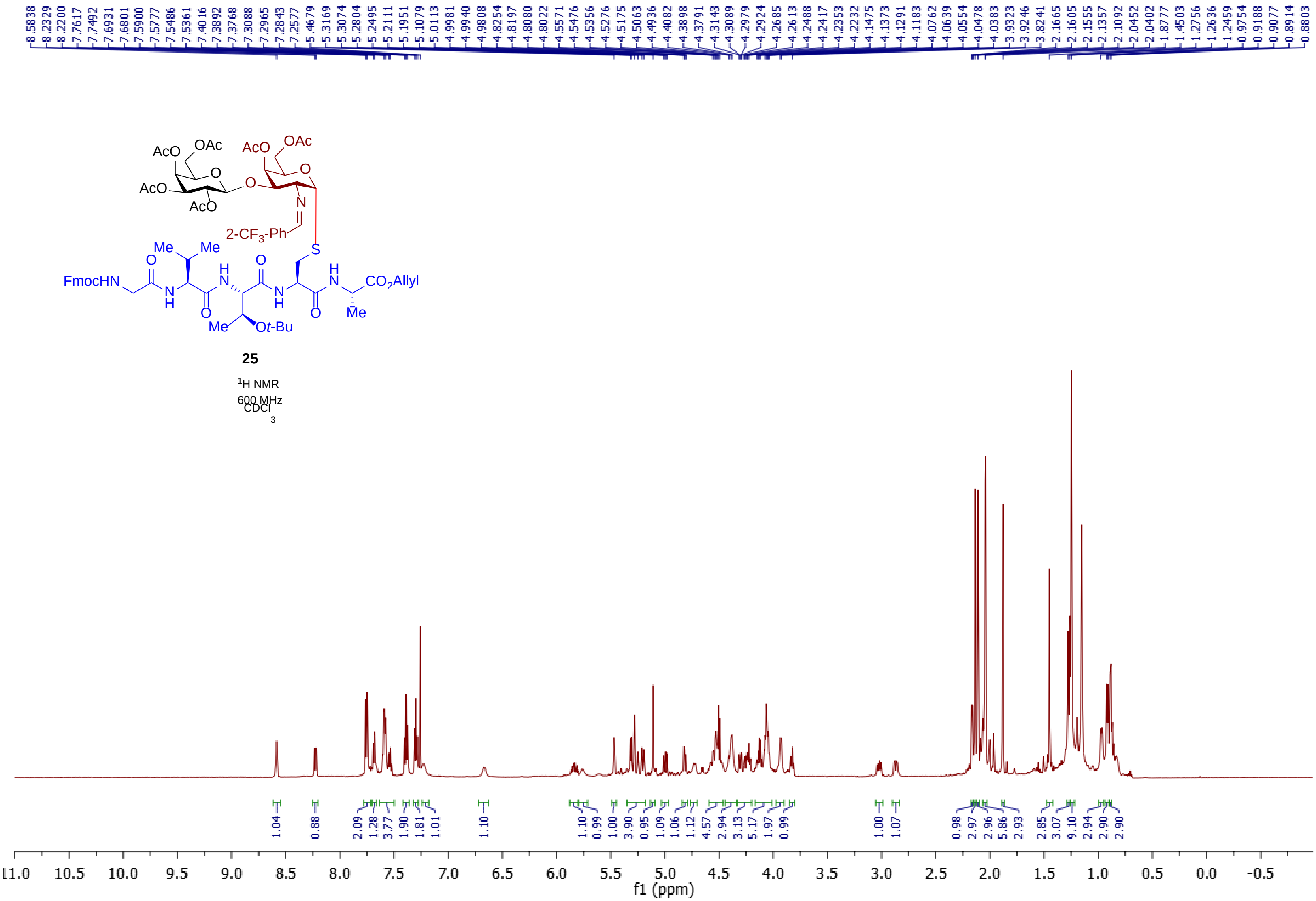
25
1H NMR
600 MHz
CDCl3
f1 (ppm)

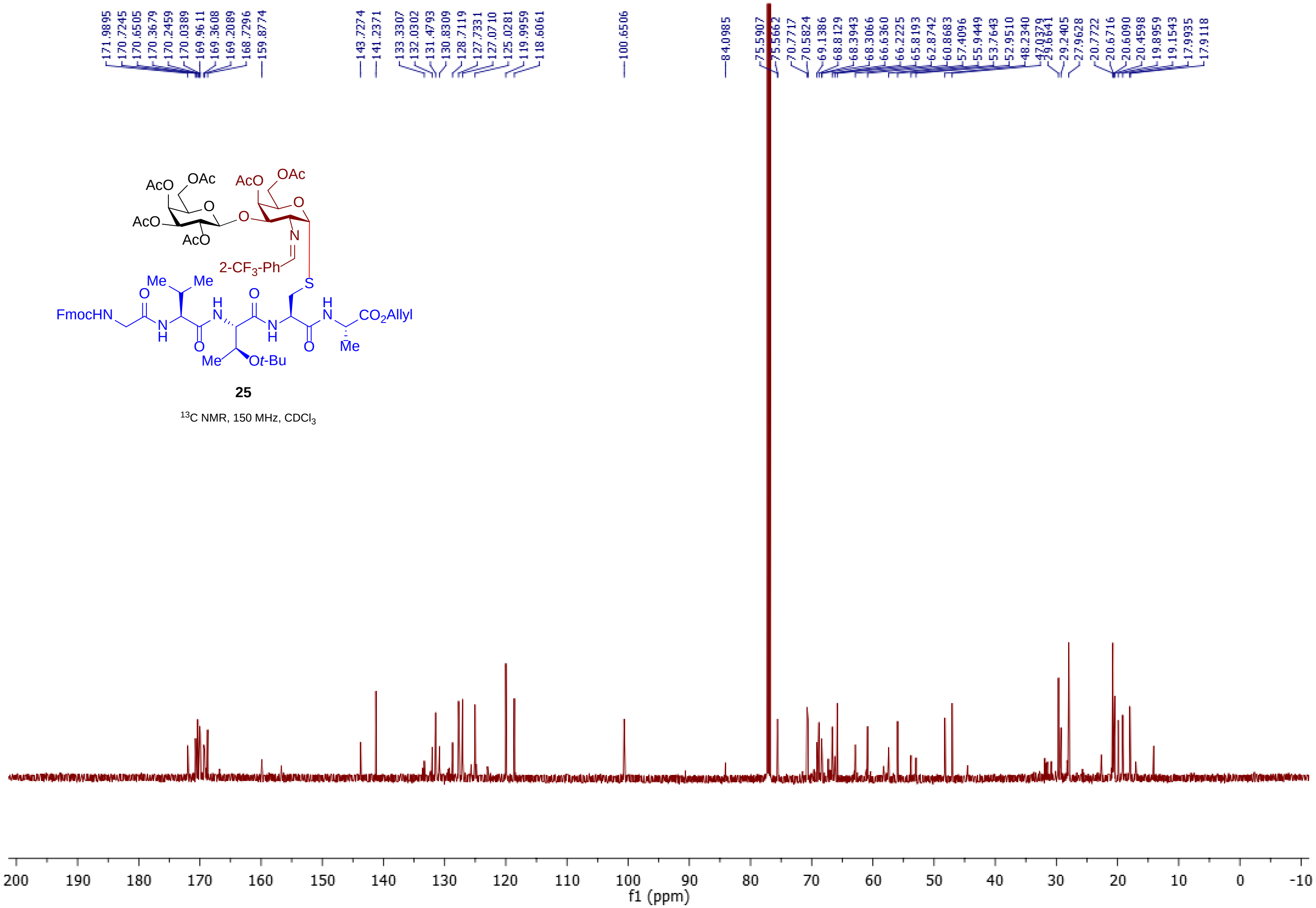

25

^{13}C NMR, 150 MHz, $CDCl_3$

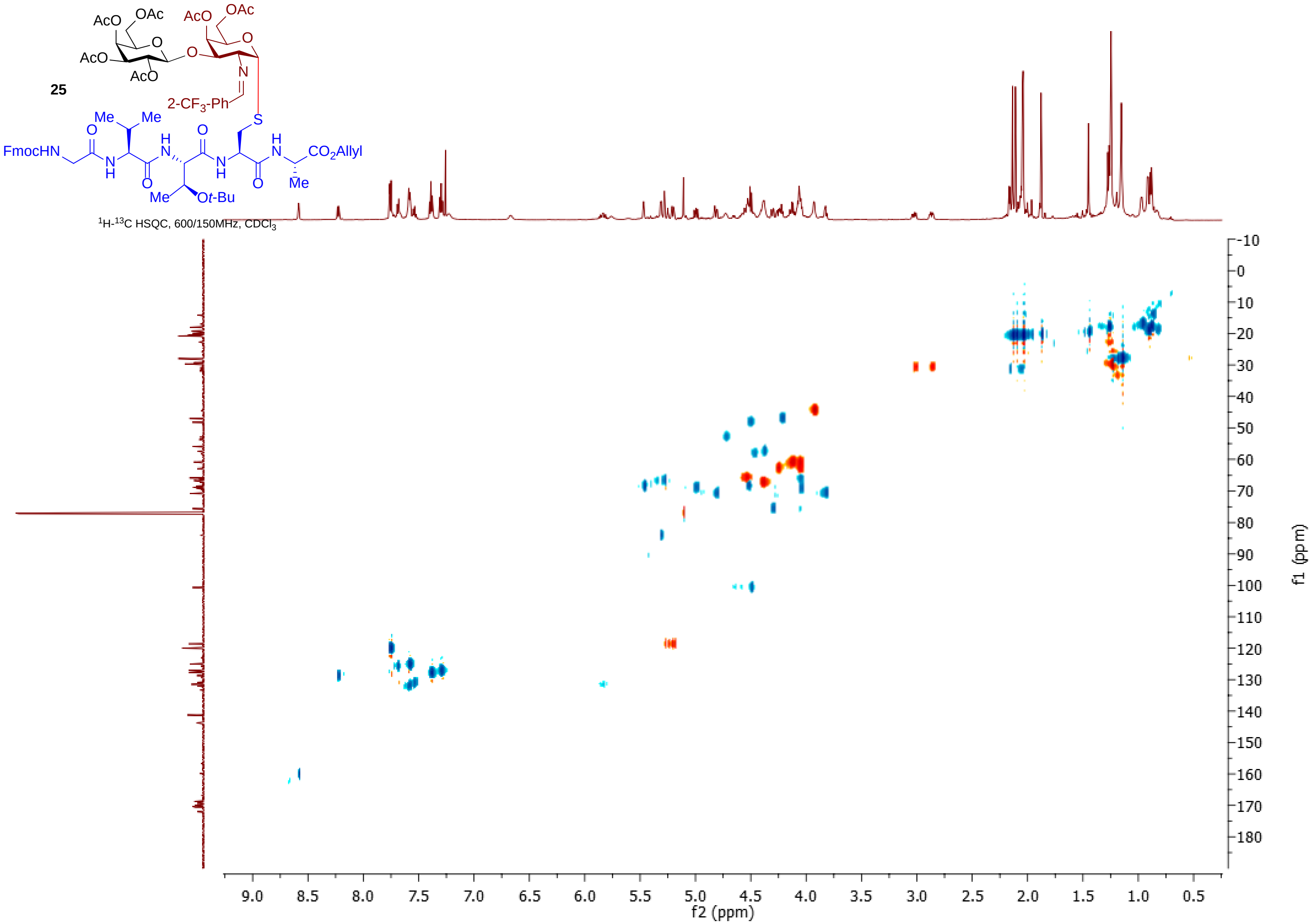

25
^{1}H-^{13}C HSQC, 600/150MHz, $CDCl_3$
f2 (ppm)
f1 (ppm)

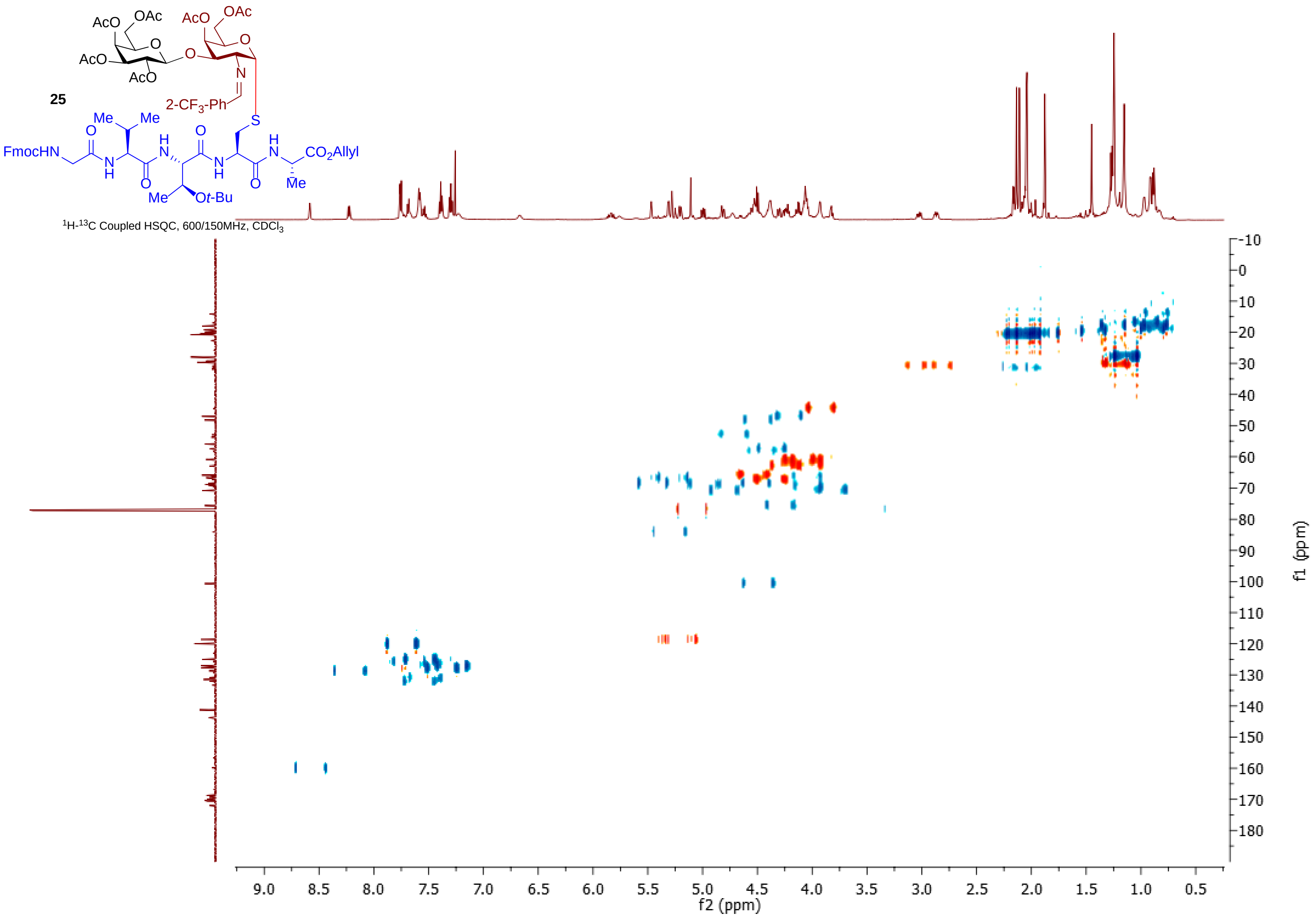
25
1H-13C Coupled HSQC, 600/150MHz, CDCl3
f2 (ppm)
f1 (ppm)
